# Antioxidant Chemistry of Phenolics in Chinese Herbal Medicines: A Holism‐Based Review

**DOI:** 10.1002/cbdv.71716

**Published:** 2026-09-20

**Authors:** Xican Li

**Affiliations:** ^1^ School of Chinese Herbal Medicine Guangzhou University of Chinese Medicine Guangzhou China

**Keywords:** antioxidant mechanism, antioxidation, Chinese materia medica, phytophenol, radical adduct formation

## Abstract

Chinese herbal medicines (CHMs) are used in traditional Chinese medicines (TCM) clinical practice under the guidance of the holism theory. Their clinical usages are basically related to their antioxidant effects; therefore, there have been accumulated a great deal of antioxidant studies in the past 40 years. The review systematically analyzes 769 available references, to find that the antioxidant capacity of CHMs overwhelmingly arises from phenolics (including polyene‐conjugated phenolics) rather than saponins or polysaccharides. The antioxidant mechanisms of phenolics, however, mainly include electron‐transfer (ET), proton‐transfer (PT), hydrogen atom transfer (HAT), ET–PT, PT–ET, and radical adduct formation (RAF). The RAF can occur at different binding sites to form different bonding types to cause various final products (including phenolic‐radical adducts, and phenolic‐phenolic dimers) and be affected by structural factors to show certain stereochemical characters. Further structure–activity relationships analysis indicates that the antioxidant effect of phenolics is seemingly attributed to the existence of specific antioxidant moieties (e.g., catechol); however, the high levels of these so‐called “specific antioxidant moieties” can be attributed to the stability of the oxidized products from their reactions with free radicals and, ultimately, to the degree of conjugation of the whole antioxidant molecule.

## Graphical abstract

Phenolic antioxidants in CHMs mainly include chalcone, stilbene, flavonoid, xanthone, and Diels–Alder‐type adducts. They undergo direct and indirect pathways to exert antioxidant action. The indirect pathway is transition metal‐chelating, while the direct pathway comprises several redox‐based reactions (e.g., SET and HAT) and RAF reactions. The direct pathway is affected by substituent group and governed by a holism‐based antioxidant rule.

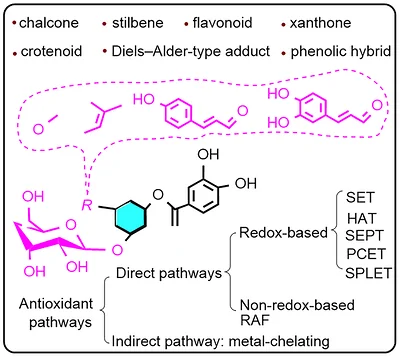

## 1 Introduction

Chinese herbal medicines (CHMs) are the drugs used in traditional Chinese medicines (TCM). Although most CHMs are extracted from plants, including marine algae [1, 2], the development of CHMs, however is based on the holism theory of TCM. TCM defines these CHMs various clinical efficacies, such as detoxification [3, 4], clearing away *heat* [4], and *Huoxue Huayu* (i.e., promoting blood circulation for reversing blood stasis) [5]. These clinical efficacies have been recently reported to be related to antioxidant effects. In particular, CHMs with *Huoxue Huayu* efficacies have been shown to be sources of natural antioxidants [6].

Antioxidant research concerning CHMs began in 1986 [7]. During the past 40 years, about 44 000 research articles papers involving antioxidant properties of CHMs have been published around the world. The review, however, collected 769 available references for literature analysis (Supporting Information‐1). Through analysis, the antioxidants in CHMs are found to mainly include phenolics [2], polyenes [8, 9], saponins [10, 11], and polysaccharides [12, 13]. However, our previous works concluded that the correlation coefficient between antioxidant levels and saponin or polysaccharide chemical content is very low [14, 15]. Furthermore, the antioxidant ability of saponins (or polysaccharides) themselves is much lower than that of phenolics [16, 17]. Thus, polysaccharides and saponins actually do not comply with the definition of an antioxidant, in that an antioxidant is a species that can effectively scavenge reactive oxygen species (ROS) at much lower concentrations than the substrate oxidized by ROS [18] (e.g., ^•^OH [19] and ^•^O_2_^−^ [20]). Hence, this paper will focus on phenolic antioxidants (including polyene‐conjugated phenolic antioxidants) and overlook polysaccharides and saponins.

Previously, phenolic antioxidants from CHMs have been simply compiled in two review papers, but their chemical mechanism has not been discussed [11]. Other review papers, however, focus on determination methods [21, 22, 23], structure–activity relationship [24, 25], chemical mechanisms, and chemical kinetic [26, 27, 28, 29]. Obviously, these are of little irrelevance to CHMs or TCM. The present work summarizes a basic database of structure and cross‐distribution of 477 phenolic antioxidants in 331 CHMs (1986 to 2026, Table S1), to outline the chemical characteristics of these antioxidants. Most notably, it focuses on antioxidant chemical mechanism based on holism. Our work involving radical adduct formation (RAF) studies based on ultra‐performance liquid chromatography coupled with electrospray ionization quadrupole time‐of‐flight tandem mass spectrometry (UPLC‐ESI‐Q‐TOF‐MS/MS) is also highlighted.

## 2 Chemistry of Antioxidant Mechanisms

Chemically, the antioxidant action of a phenolic is a free‐radical‐mediated chain reaction that proceeds via an initiation step (e.g., pyrogallol autoxidation [20]), a propagation step, and a termination step [18, 30, 31]. Cellular chain reactions, however are initiated through breath or ischemia‐reperfusion injury at the mitochondrion, where ROS are generated [32, 33]. Thus, the phenolic ROS‐scavenging reaction likely takes place mainly at the propagation and termination steps. At the propagation step, phenolic antioxidants can interact with ROS via multiple pathways, including electron‐transfer (ET), ET *plus* proton‐transfer (PT), and hydrogen‐atom‐transfer (HAT) pathways [28, 34, 35]. The termination step, however is fulfilled by RAF pathway [18, 28].

### 2.1 ET Pathway

One pathway in phenolic antioxidants from CHMs is ET (namely single electron transfer, SET) [36]. In 2010, two phenolics, verbascoside (acteoside) and quercetin from *Xuanshen* (*Scrophularia ningpoensis*), have been indicated to involve in ET pathway to quickly repair DNA radicals (e.g., the 2'‐deoxyguanosine‐5'‐monophosphate radical, dGMP^•^), the initiator source of gene mutations [37]. It suggested that ET pathway plays a possible role in the interaction of CHMs‐derived phenolics with biomolecules. The latest study stated that, in an aqueous environment, ET was the dominant pathway for many phenolic antioxidants [38].

However, phenolic ET pathway would cause unstable radical cations [39]; in biological system, (phenolic) ET pathway is always accompanied by a PT pathway [37, 39, 40, 41].

### 2.2 ET *plus* PT Pathways

The PT pathway is also termed as H^+^‐transfer pathway [40, 41] and its essence is a deprotonation reaction [42]. The deprotonation, however, has been reported to widely occur in phenolic antioxidants under physiological conditions and could be easily characterized by *p*Ka value [43].

According to the sequence and collaboration, the PT pathways accompanied by ET can be divided into three subtypes [44, 45]. (i) If PT precedes ET, this is called the PT‐ET pathway [46], or the proton loss single electron transfer (SPLET) pathway [21, 26, 28, 34, 47]. (ii) If ET precedes PT, this is known as the ET–PT pathway [28], or the sequential electron–proton transfer (SEPT) pathway [48]. (iii) If ET and PT occur simultaneously as two separate species, this is called the proton‐coupled electron transfer (PCET) pathway [21, 26, 28, 34, 49], or the concerted proton–electron transfer (CPET) pathway [50]. These pathways are collectively termed as ET *plus* PT pathways.

### 2.3 Hydrogen Atom Transfer (HAT) Pathway

However, if ET and PT occur simultaneously as a single unit, this is well‐known as the HAT pathway [21, 26, 28, 30, 34, 51]. It has also been documented as hydrogen‐donating reaction [18] or hydrogen‐abstraction reaction [28, 52, 53, 54]. The HAT potential of a phenolic antioxidant lies on the BDE (bond dissociation enthalpy or bond dissociation energy) value of ArO‐H [26, 28].

All these antioxidant pathways are competitive during the antioxidant process of one phenolic antioxidant. For example, the HAT pathway has been proven to initiate cellular oxidative damage, especially lipid oxidation [18], it may still compete with other antioxidant mechanisms [11, 48, 55]. A latest theoretical study suggested that, the aforementioned quercetin could also mediate HAT, SPLET, and ET–PT antioxidant pathways to play an antioxidant role [56].

Nevertheless, the net results of each antioxidant pathways are identical with a hydrogen‐abstraction reaction, that is, the phenolic antioxidant loses a hydrogen atom to form a phenolic radical (ArO^•^), and that radical acquires a hydrogen atom to transform into a stable radical‐H molecule [28, 52].

### 2.4 RAF Pathway

After hydrogen loss at the propagation step, the phenolic antioxidant molecule is converted into ArO^•^ radical. At the termination step, ArO^•^ may undergo an RAF reaction to yield the final products, which include the stable adduct molecules (or non‐radical products) [18, 57]. Thus, the reactivity of the free radical (or radical intermediate) quickly diminishes, and correspondingly, the chain reaction gets terminated. This is termed as the RAF antioxidant pathway [58, 59, 60]. Dissimilar to the four antioxidant mechanisms that cause an identical net result, RAF can yield a myriad of products, including phenolic–phenolic dimers and phenolic‐radical adducts [58, 61, 62]. The formation of these products, however, can further be affected by the bonding site, bond type, and reaction conditions [57, 63].

#### 2.4.1 Phenolic–Phenolic Dimerization

**SCHEME 1:**
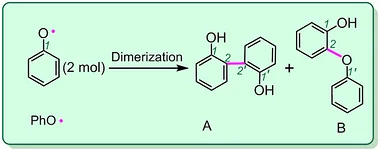
Dimerization reaction of phenol.

If the ArO^•^ species link with each other via covalent bonds at the termination step, this will yield a polymer (especially a dimer) [28]. The simplest example is that of phenoxy radical (PhO^•^), which produces two dimers [29] (Scheme 1). Hence, dimers can be regarded as one type of RAF product.

**SCHEME 2:**
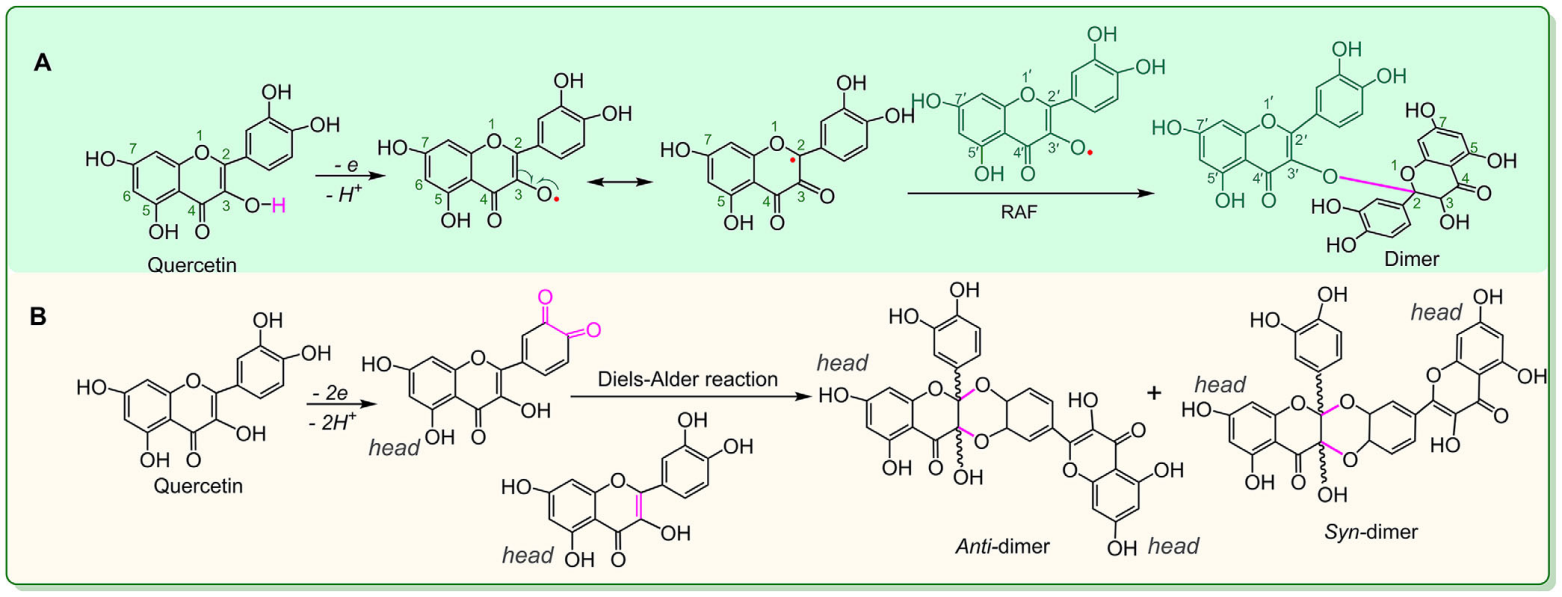
Generation of three dimers of quercetin.

For phenolics from CHMs, however, the dimerization reaction is more complicated. For example, quercetin, a phenolic commonly found in CHMs (Table S1), has been proposed to produce three dimers using theoretical chemistry [46] (Scheme 2).

#### 2.4.2 Phenolic‐Radical Addition

**SCHEME 3:**
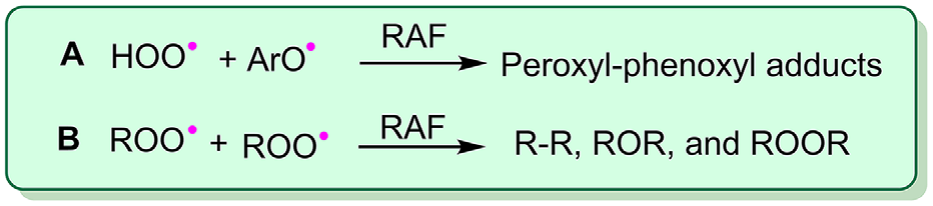
Production of radical adducts.

The aforementioned ArO^•^ can also covalently link to ROS to form a phenolic‐radical adduct [34]. For example, ArO^•^ can combine with ROO^•^ (alkylperoxyl radical) to yield radical adducts (Scheme 3A) [28, 31]. Furthermore, ROO^•^ species can also combine with each other to produce R–R, ROR, and ROOR via a lipid oxidation reaction (Scheme 3B) [18]. For example, tyrosine phenolic radical can combine with LOO^•^ (lipid peroxyl radical) to give LOO‐tyrosine adduct [57]. An echinatin‐DPPH^•^ adduct has also be found by cutting‐edge UPLC‐ESI‐Q‐TOF‐MS/MS technology (see Section 2.4.4.2).

#### 2.4.3 Effects of Structural Factors and Reaction Conditions

Like all chemical reactions, RAF can be affected by various structural factors and reaction conditions. Structural factors include, at the very least, deprotonation, intramolecular hydrogen bonds (IHBs), steric hindrance, and reactive site [64, 65]. Theoretical chemistry has shown that deprotonated *α*‐mangostin cannot effectively scavenge the ROO^•^ via RAF [66]; on the other hand, IHBs can stabilize the RAF product of silychristin with the LOO^•^ [67]. The experiments on authentic samples have suggested that steric hindrance can also affect the generation of dihydrochalcones via RAF [68] (Section 4.3). This explains why trimeric or tetrameric phenolics are so rare [69, 70], and why the synthesis of these phenolics is so challenging [63]. According to Pauling's resonance theory, a larger conjugative system provides more possible resonance structures. Hence, a conjugative antioxidant molecule was hypothesized to have different reactive sites for RAF linkage [63]. However, this hypothesis is supported by neither experimental nor theoretical evidence.

**SCHEME 4:**
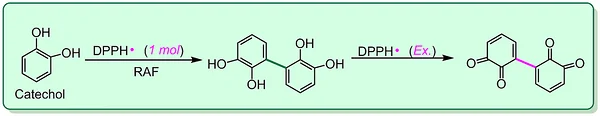
Reaction of catechol with DPPH•.

In addition, the RAF reaction is reportedly affected by the reaction conditions such as the stoichiometry and solvent [71]. For example, catechol (1,2‐dihydroxybenzene) reacts with DPPH^•^ at different molar ratios to yield different RAF products (Scheme 4) [52].

#### 2.4.4 RAF Study: A Potential New Field in Free Radical Chemistry

As discussed above, the RAF reaction is so complicated and variable that it can generate a variety of potential RAF products. On the other hand, some RAF products mediated by cellular ROS act as important biomarkers for disease screening or cytotoxins to induce cellular death [72, 73]. The complexity, variability, and importance are sufficient to establish an entirely new field in free radical chemistry. However, there are no reviews focusing on RAF studies [18, 21, 26, 28, 34, 37] this review, therefore, aims to provide both a broad and deep discussion.

##### 2.4.4.1 History and Limitations

In 1975, RAF was first identified in the ionizing radiation product of uracil, a primary base [74]. Structurally, uracil can also be considered a heterocyclic phenolic, which has been proven to be a good ^•^OH‐scavenger (IC_50_ 80.1 ± 2.89 µg/mL) [19]. In 1995, eugenol, a volatile phenol from CHMs, was hypothesized to yield two RAF products (i.e., eugenol dimer and eugenol‐radical adduct). However, there was neither experimental nor theoretical evidence to support the hypothesis [75].

Subsequently, a number of theoretical chemical studies tried to explore the RAF pathway of phenolic antioxidants [48, 58, 66, 76, 77, 78]. A recent study used density functional theory (DFT) calculations to exploit the RAF potential of chlorogenic acid, a phenolic acid widely occurring in CHMs [59].

However, these theoretical studies lacked authentic samples and reactional environments, and hence may have produced unreliable results. For example, the solvent effect has been frequently ignored [19]. The reaction solvent is prone to interaction with oxygen‐centered radicals (especially ^•^OH [19]) [34], nitrogen‐centered radicals (e.g., DPPH^•^) [21, 26], and even carbon‐centered radicals [30]. In our previous study, the ethanol solvent could bring about a 9‐fold greater ^•^OH‐scavenging percentage than the realistic value [19]. The DMSO solvent, however, could also disturb the ^•^OH‐scavenging assay; DMSO and ^•^OH can experience a RAF reaction to give a radical adduct (CH_3_)_2_S^•^‐OH, which can further release CH_4_ [19, 79].

RAF studies based on authentic samples were started in 2006. (+)‐Catechin treated with ABTS ^•+^ radicals was observed to yield several RAF products, with the aid of HPLC‐MS/MS technology [80]. However, this HPLC‐MS/MS technology was not equipped with peak‐extracting software. As such, the RAF products did not display distinctly separate chromatographic peaks, and thus could not be effectively elucidated using the MS spectra [81].

##### 2.4.4.2 UPLC‐ESI‐Q‐TOF‐MS/MS Strategy and Instances

To overcome the above limitations, the cutting‐edge UPLC‐ESI‐Q‐TOF‐MS/MS technology was equipped with peak‐extracting (or selected‐ion‐monitoring) software [57]. The software can well extract molecular ion peaks in MS spectra, and related fragment peaks in MS/MS spectra.

**FIGURE 1:**
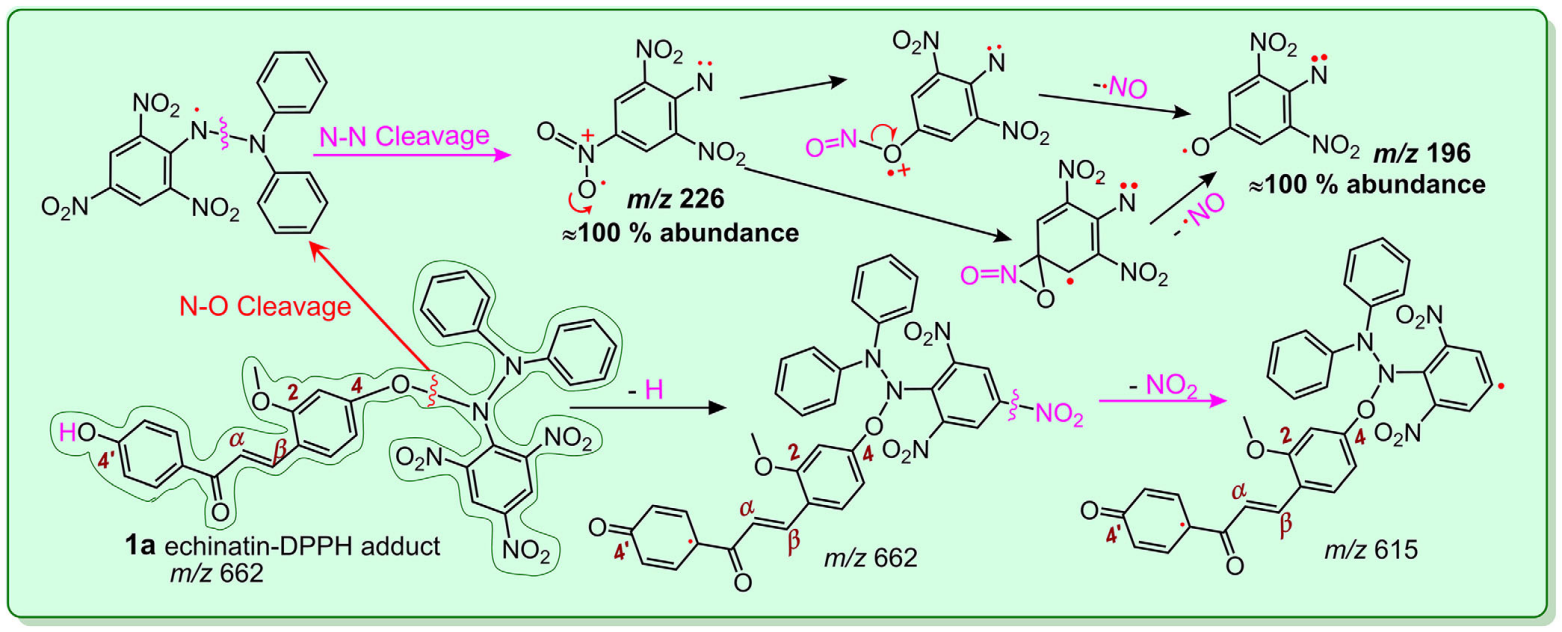
The proposed MS elucidation of echinatin‐DPPH adduct (*Note*: other possible covalent linkages should not be excluded).

**FIGURE 2:**
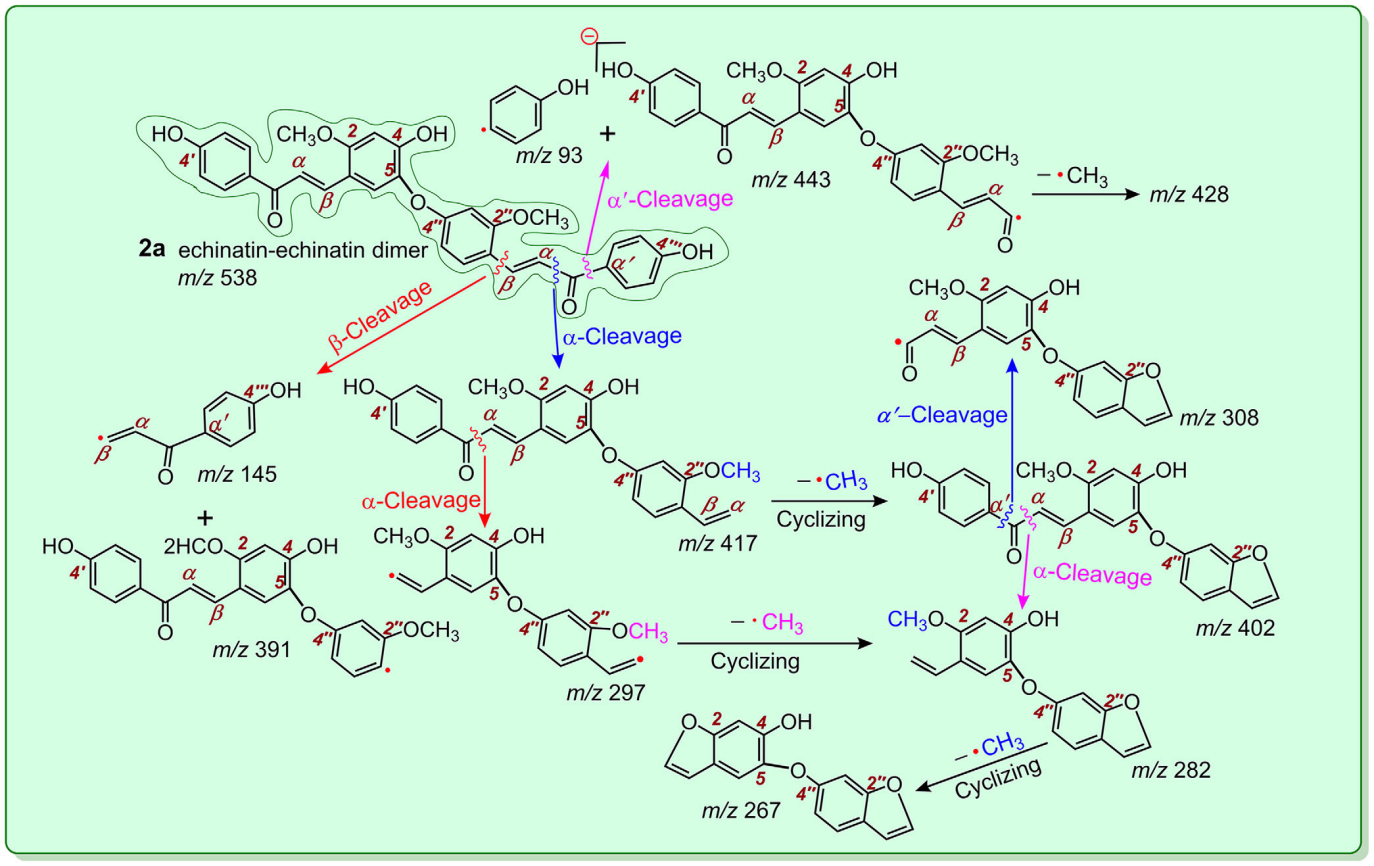
The proposed MS elucidation of echinatin–echinatin dimer (*Note*: other possible covalent linkages should not be excluded).

Using the cutting‐edge technology, the RAF pathway of echinatin, a chalcone occurring in the CHM *Gancao*, was investigated. As shown in the literature [61], echinatin generated relevant MS peaks and MS/MS peaks, after incubation with 2 mol DPPH^•^. The MS elucidation suggested that these MS peaks might have been from the echinatin‐DPPH adduct (**1a**) and echinatin‐echinatin dimer (**2a**) (Figures 1 and 2).

Simultaneous occurrence of both the echinatin‐DPPH adduct and echinatin–echinatin dimer clearly indicates that the interaction of echinatin with DPPH^•^ is a free radical chain reaction. This is because: (1) if the reaction is conducted under the condition of 2 mol DPPH^•^, 1 mol echinatin is synchronously coupled with DPPH^•^ to yield only the echinatin‐DPPH adduct (without the echinatin–echinatin dimer), similar to Diels–Alder adduct formation [57]; (2) if the reaction is ion‐driven (rather than radical‐driven), echinatin may transform into echinatin^+^ (or echinatin ^−^). Two echinatin ^+^ (or echinatin ^−^) ions, however, cannot covalently link with each other.

**FIGURE 3:**
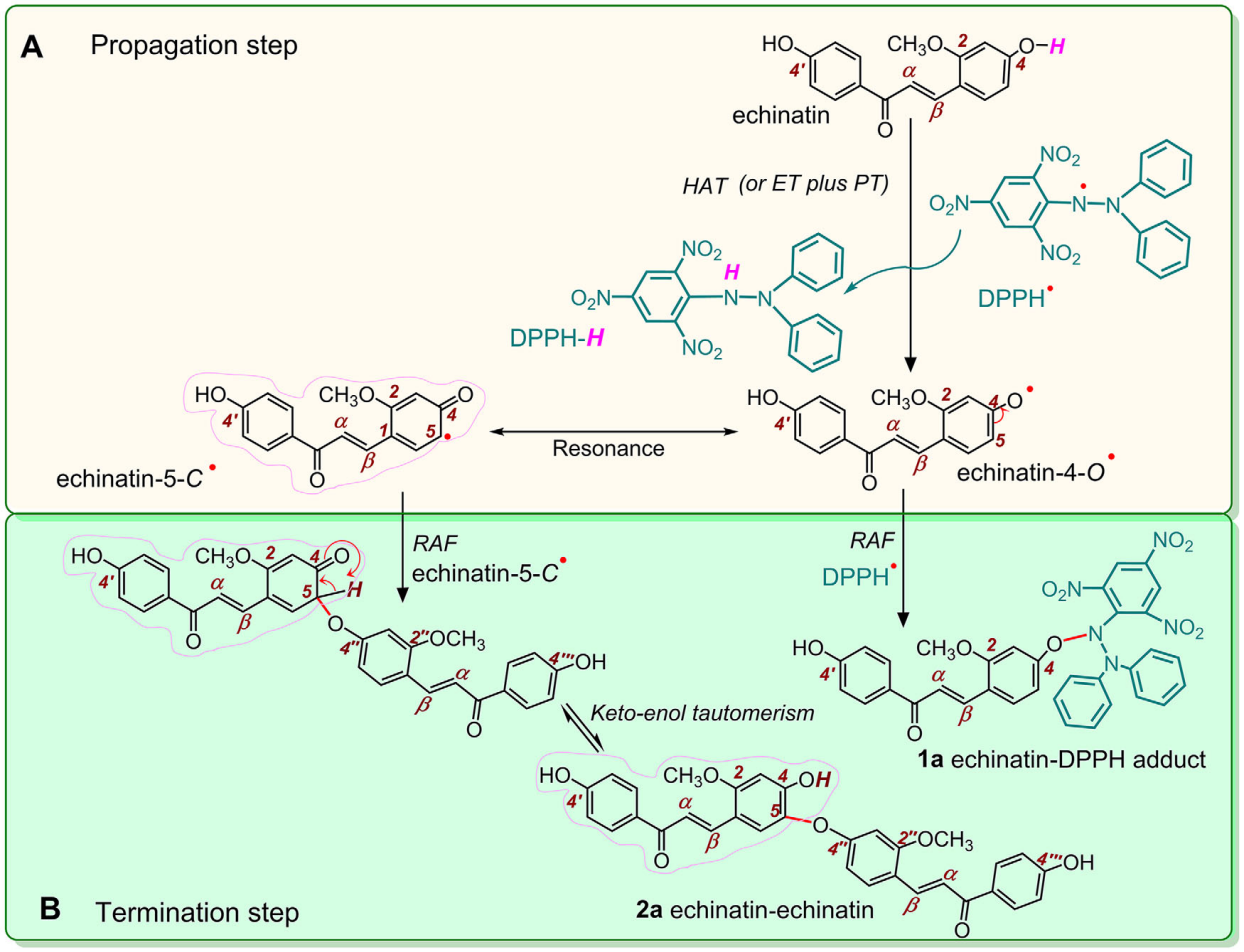
The proposed radical chain reactions of echinatin with DPPH^•^ radical: (A) termination step; (B) propagation step.

Based on the above MS spectra, deduction, and previous literature [82], the free radical chain reaction of echinatin with DPPH^•^ was proposed to include the propagation and termination steps (Figure 3). The RAF pathway may take place at the termination step. This relatively complete chain reaction proposal is basically consistent with the previous hypothesis [75].

In addition to echinatin, the UPLC‐ESI‐Q‐TOF‐MS/MS strategy has also been used to investigate other phenolic antioxidants from CHMs, including scutellarein [83], licochalcone A [61], galangin, 3,5,7‐trihydroxychromone [84], wedelolactone [85], maclurin [86], gallic acid, (+)‐catechin, epigallocatechin gallate (EGCG) [87], and five dihydrochalcones [68]. However, not all of them exhibited strong and distinct MS spectra, owing to their inherent molecular structures.

**FIGURE 4:**
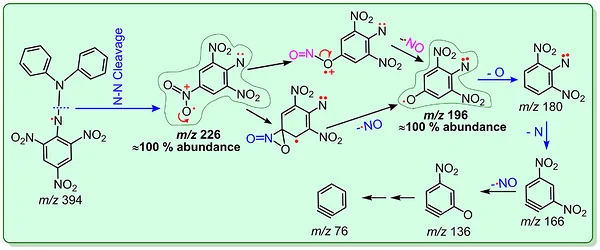
The proposed fragmentation of DPPH^•^ in ESI‐MS spectra (negative ion model).

Hence, it is strongly advised to select appropriate free radicals and phenolics as probes for such RAF studies. Ideal free radical probes, however, are required to present three advantages, that is, adequate reactivity, adequate molecular weight, and characteristic MS fragments. An adequate molecular weight can give rise to an adequate *m/z* value, distinguishable from those of common small molecules (e.g., CO_2_, *m/z* 44); whereas characteristic fragments can exhibit characteristic *m/z* values for further identification. The previous works suggested that DPPH^•^ is an ideal free radical probe. DPPH^•^ has adequate reactivity and molecular weight (M.W. 394); more importantly, it exhibits two characteristic fragment peaks (*m/z* 196 and 226) in the ESI‐MS field under negative ion model (Figure 4). These two peaks are so abundant that they can even act as basic peaks (100% abundance) [61, 88] (Figures 1 and 4). Given that an antioxidant mixed with DPPH^•^ displays an *m/z* 394 increment in MS spectra, and *m/z* 196 and 226 fragments in MS/MS spectra, it can be verified to undergo RAF with DPPH^•^. This is apparently more convincing than theoretical studies [48, 58, 59, 66, 76, 77, 78].

Other free radical probes may also be applicable to such investigations, e.g., 2‐phenyl‐4,4,5,5‐tetramethylimidazoline‐1‐oxyl‐3‐oxide radical (PTIO^•^) [35, 89], 2,2,6,6‐tetramethyl‐1‐piperidinyloxy radical (TEMPO^•^) [31], galvinoxyl radical [90] and diarylnitroxide radical (Ar_2_NO^•^) [31]. The PTIO^•^ has been recently used as a free radical probe for antioxidant assay in physiological aqueous solution by our team [35]; however, in RAF pathway, it is more inactive than DPPH^•^ [61, 89]. The TEMPO^•^, however has been recently to be inferior to Ar_2_NO^•^ in RAF potential. This is probably because both PTIO^•^ and TEMPO^•^ are aliphatic radicals [31]. Thus, it is advised to choose the aromatic radicals for probe in the RAF study.

Ideal phenolic probes should be a set of analogues or known authentic samples, because they can provide important information through structure–activity relationship analysis or comparison [91]. Alternatively, a series of synthetic compounds with bonding sites information act as the probes to identify bonding sites [57]. In other words, smart usage of the UPLC‐ESI‐Q‐TOF‐MS/MS strategy will provide reliable RAF evidence and important information (especially covalent bonding sites).

It is important to note that GC–MS could be used for RAF studies. However, it requires high temperature and volatility of the tested sample. Most phenolic antioxidants are known to be non‐volatile, and a high temperature can induce the cleavage of newly‐formed covalent bonds in RAF products. Therefore, the usage of GC–MS has been greatly limited and seldom reported [92].

##### 2.4.4.3 Implications and Perspectives

**FIGURE 5:**
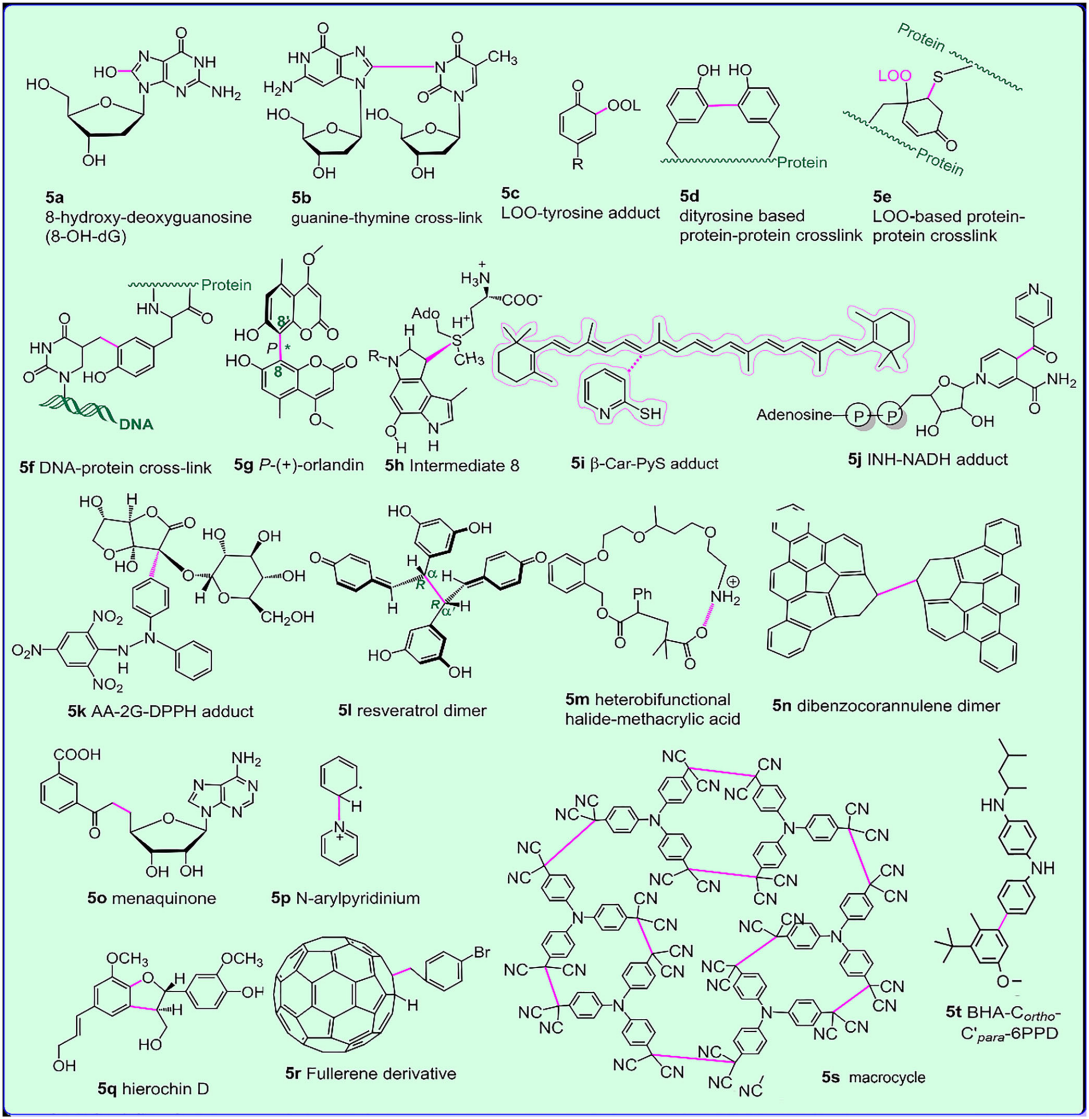
The structures of RAF instances **5a–5t** (LOO, lipid peroxide; β‐Car‐PyS, β‐carotenyl‐2‐pyridylthiyl; INH‐NADH, isonicotinoyl‐nicotinamide adenine dinucleotide; AA‐2G, 2‐*O*‐α‐D‐glucopyranosyl‐L‐ascorbic acid; BHA, butylated hydroxyanisole; N‐(1,3‐dimethylbutyl)‐*N*′‐phenyl‐*p*‐phenylenediamine).

The above findings concerning RAF are obviously based on phenolic antioxidants (especially those from CHMs). However, these main principles and technological approaches (especially the UPLC‐ESI‐Q‐TOF‐MS/MS strategy) may further shed light on a wide range of academic or applicable fields (Table 1 and Figure 5).

**TABLE 1:** The academic or applicable fields and instances relevant to RAF studies.

Academic or applicable fields	Instances
DNA‐radical adduct	8‐Hydro‐deoxyguanosine (8‐OH‐dG, **5a**) in DNA [93]
DNA crosslinking	Guanine‐thymine crosslink (**5b**) [94]
Protein‐radical adduct	LOO‐tyrosine (phenolic) adduct (**5c**) [57]
Protein crosslinking	Dityrosine‐based protein‐protein crosslink (**5d**) [95] LOO‐based protein‐protein crosslinking (**5e**) [57]
DNA‐protein interaction	DNA‐protein crosslink (**5f**) [72]
Natural product chemistry (CHM Chemistry)	*P‐*(+)‐orlandin (**5g**) [96, 97] Intermediate 8 (**5h**) [40]
Food, beverage, and nutrition	β‐Carotenyl‐2‐pyridylthiyl (β‐Car‐PyS) adduct (**5i**) [98]
Pharmacology	Isonicotinoyl‐nicotinamide adenine dinucleotide (INH‐NADH) adduct (**5j**) [99]
Medicinal chemistry	2‐*O*‐α‐D‐Glucopyranosyl‐L‐ascorbic acid‐DPPH (AA‐2G‐DPPH) adduct (**5k**) [100]
Synthetic chemistry	Resveratrol dimer (**5l**) [63] Heterobifunctional halide‐methacrylic acid (**5m**) [101] ]Dibenzocorannulene dimer (**5n**) [102]
Bioorganic chemistry	Menaquinone (**5o**) [103]
Electrochemistry	N‐arylpyridinium (**5p**) [104]; Hierochin D (**5q**) [105]
Nanomaterials	Fullerenes, C_60_ (**5r**) [106, 107]
Supramolecular chemistry	Macrocycle (**5s**) [108]
Environment chemistry	Phenol‐arylamine adduct (**5t**) [109]

Of the instances listed in Figure 5, 8‐OH‐dG (**5a**) from DNA oxidative damage can be used as a biomarker of genetic mutation and carcinogenesis (e.g., lung cancer); In fact, the relevant enzyme‐linked immunosorbent assay (ELISA) kit has been commercialized [73, 110].

The lipid peroxide‐tyrosine adduct (**5c**) and dityrosine‐based protein–protein crosslink (**5d**) are formed at the *ortho* position of phenolic ‐OH when protein interacts with LOO• [57, 95, 111]; If the adduct is linked at the *para* position, it may further generate a protein–protein crosslink via a S─C covalent bond (**5e**) [57]. These adducts can lead to cellular membrane nitration, and then induce atherosclerosis and neurodegenerative disorders (e.g., Parkinson's and Alzheimer's diseases) [57, 111].

Tyrosine with phenolic ‐OH however can couple with thymidine to form dT‐Tyr adduct to link DNA and protein (**5f**) [72], after oxidization by cellular ROS [112]. Ultimately, such DNA‐protein cross‐linking may be attributed to ischemia‐reperfusion injury [72]. However, it remains unknown whether the latest finding can be used for clinical diagnosis.

Cellular ROS may also participate C─C dimerization of phenolic coumarin 7‐demethylsiderin to *P*‐(+)‐orlandin (**5g**). This is a biosynthetic RAF reaction catalyzed by cytochrome P450 [96, 97]. Such biosynthesis has been shown to be stereoselective (see: Section 3.3).

The fullerene derivative (**5r**) has been mentioned to be synthesized via RAF reaction. Now it is expected to play a role in material, biological, and pharmacological applications [106, 107]. Dissimilar to **5r**, macrocyle (**5s**) however is a supramolecule. As seen in Figure 5, such supramolecule is self‐assembled through 9 new C─C covalent bonds. The formation of these covalent bonds also depends on RAF reactions [108]. Finally, RAF may also involve in other fields, such as plastics, lubricants, and cosmetics [26, 28]. All these instances have actually suggested the universality of RAF studies in human production and life.

The reason why RAF studies can involve so many academic or applicable fields is that, on earth, oxygen gas (O_2_) exists everywhere; hence, oxygen‐induced free radical chain reactions occur everywhere. Accordingly, free radical‐mediated antioxidant reactions (especially RAF) and relevant techniques are applied to various academic or applicable fields: from plants to animals; industry to preventive medicine; gene mutation to protein damage; small molecule to supramolecule, and synthetic drugs to CHMs.

### 2.5 Indirect Antioxidant Mechanism: Transition‐Metal Chelating

It has been documented that, in the initiation step of chain reaction, transition metals (e.g., Fe^2+^ and Cu^+^) often act as catalysts to facilitate free radical (particularly cellular ROS) generation [18, 21, 28]. Typically, Fe^2+^ can catalyze the transformation of H_2_O_2_ into ^•^OH radicals via the Fenton reaction or Haber–Weiss reaction in cells [19, 113, 114]. Thereby, iron overload can cause nervous diseases [115]. Likely for this reason, ancient traditional Chinese medicinalists discouraged the use of ironware to boil CHMs. Chelating these transition metals can effectively release oxidative stress [21]. Transition‐metal chelation reactions can be divided into two types in terms of stoichiometry—intramolecular and intermolecular [116].

If the transition‐metal (e.g., Fe^2+^) is in excess, a phenolic molecule is able to chelate the metal core to form an intramolecular complex [116]. On the other hand, if the phenolic ligand is in excess, two or more phenolic molecules may synergistically chelate a metal core to form intermolecular complexes [27, 116, 117]. For example, quercetin‐Fe^2+^ exists as at least seven complexes, including three intramolecular and four intermolecular complexes [116]. However, even with the intake of adequate flavanone‐enriched juice or food, the flavonoid level remains much lower than that of Fe^2+^ in human plasma [118]. Therefore, the discussion about intermolecular complexes has no biological relevance.

**SCHEME 5:**
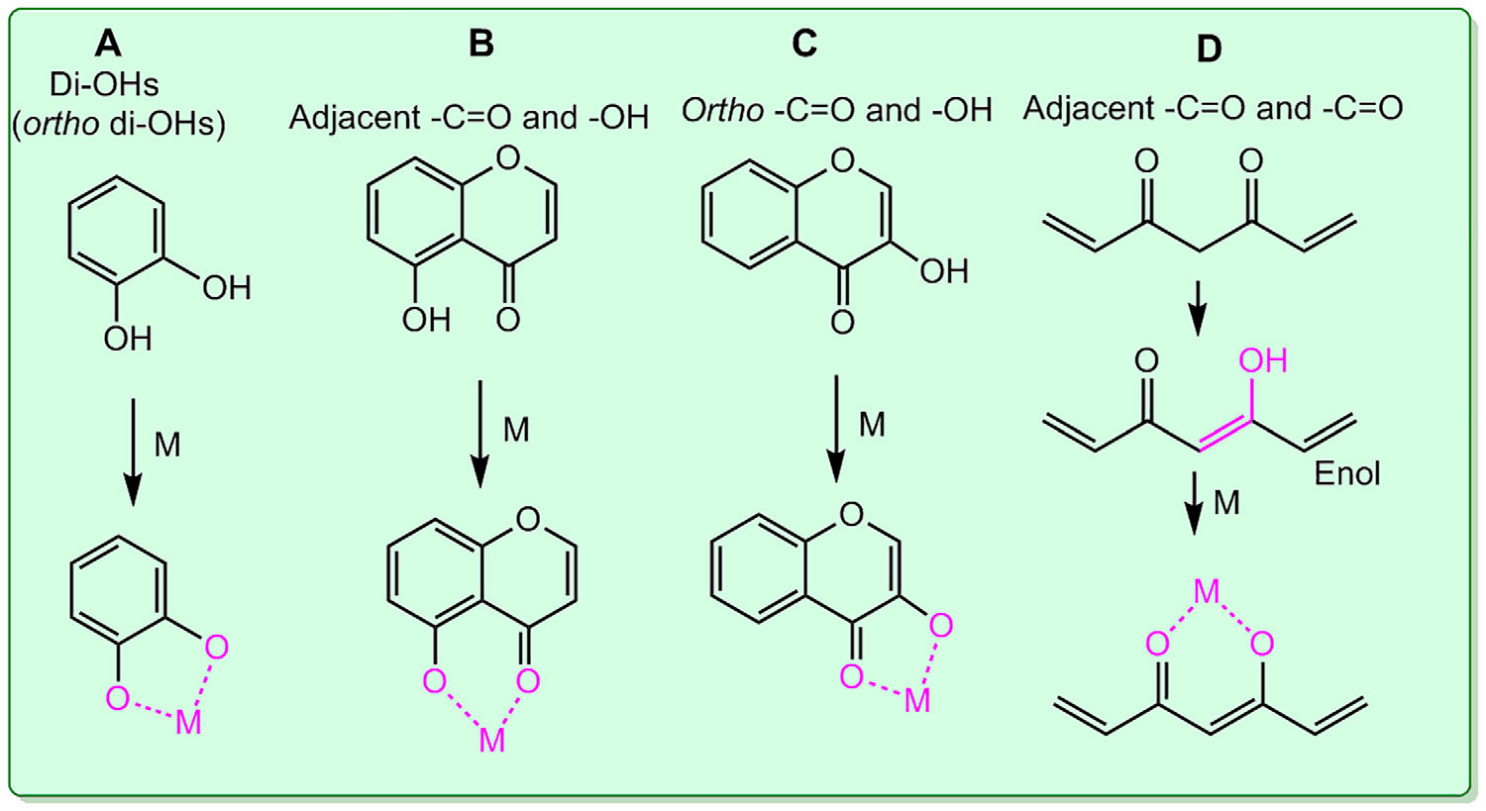
Possible metal‐chelating sites of phenolic antioxidant (M, metal).

Previous literature articles have demonstrated that a phenolic antioxidant molecule has three intramolecular chelating sites: (i) between di─OHs (Scheme 5A), (ii) between ─C═O and ─OH (Scheme 5B,C), and (iii) between di─C═O (Scheme 5D) [119, 120, 121, 122]. The isolated phenolic ─OH and ─C═O moieties have no chelating ability; likewise, the *ortho* methoxy‐hydroxy group also has no chelating ability [117, 121].

**FIGURE 6:**
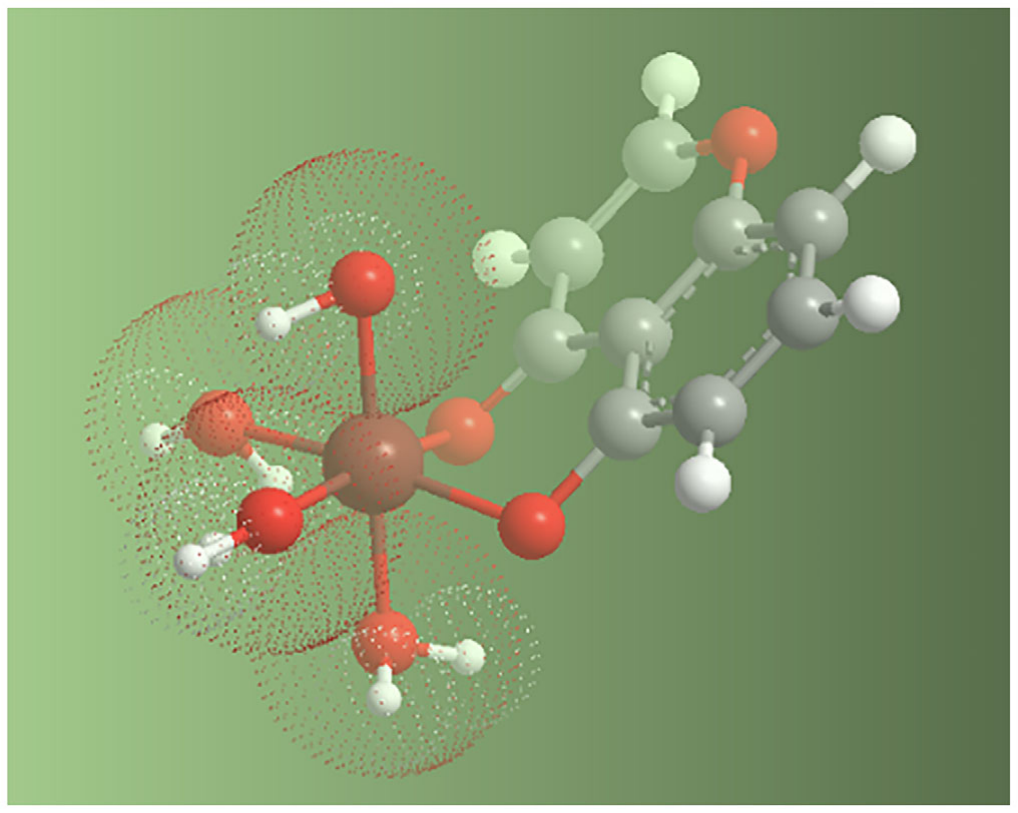
Molecular model of Fe^2+^‐complex at the adjacent ─C═O and ─OH site (including four H_2_O ligands).

- The site between di─OHs has one chelating possibility, that is, between the *ortho* di─OHs, which is also called a catechol moiety. In this moiety, the two OHs share a plane. Thus, catechols can easily chelate metals (e.g., Fe^2+^) to produce stable five‐membered rings (Scheme 5A). It is worth mentioning that the catecholic Fe^2+^‐chelating principle can also be generalized to the pyrogallol moiety (i.e., 1,2,3‐trihydroxybenzene). Owing to the popularity of the catechol (and pyrogallol) moiety, many phenolics present metal‐chelating potential in CHMs [17, 27, 83, 121, 123, 124, 125, 126, 127].
- The site between the ─C═O and ─OH moieties has two chelating possibilities—between adjacent ─C═O and ─OH (Scheme 5B), and between the *ortho* ─C═O and ─OH (Scheme 5C). The site between the adjacent ─C═O and ─OH is planar and can give rise to a six‐membered ring complex bearing Fe^2+^. However, the Fe^2+^ ion is further liganded by four water molecules to saturate its ligancy. Nevertheless, the six‐membered ring has little ring strain and displays a stable conformation (Figure 6). This may be responsible for the fact that some 5–OH flavonoids (e.g., naringenin [128] and 5–OH flavonoid itself [118]) have Fe^2+^‐chelating potential. Of course, a similar chelating site also emerges in xanthone and chalcone.
  The *ortho* ─C═O and ─OH site, however, principally occurs in flavonols [4, 117]. Unlike the adjacent ─C═O and ─OH site, the *ortho* ─C═O and ─OH site gives rise to a five‐membered ring with Fe^2+^. In this way, this site is similar to the catechol moiety [119].
- Adjacent ─C═O and ─C═O moieties may also have metal‐chelating potential. A typical example is curcumin, which can transfer one ─C═O to an enol [34, 121] (Scheme 5C and Section 4.7). Thus, the chelation of adjacent ─C═O and ─C═O is essentially that of adjacent ─C═O and ─OH.

It is worth mentioning that (1) Fe^2+^ can undergo solvation (particularly hydration) to achieve its saturated ligancy. Since the ligancy of Fe^2+^ is usually six, Fe^2+^‐complexes often display an octahedral configuration. When Fe^2+^ chelates at the adjacent ─C═O and ─OH site, the Fe^2+^‐core will be solvated by four H_2_O molecules to produce an octahedron (Figure 6).

**FIGURE 7:**
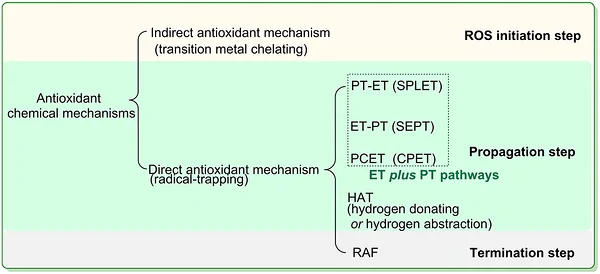
A brief summary of antioxidant pathways of CHMs‐derived phenolics.

(2) In these metal complexes, insertion of the transition metal expands the conjugative aromatic system. The expansion has decreased the energy gap between HOMO (highest occupied molecular orbital) and LUMO (lowest unoccupied molecular orbital). The metal complex molecules can absorb longer wavelengths with low energy, to easily excite the electrons, and thus present so‐called bathochromic‐shift effect [129, 130]. Therefore, the bathochromic‐shift effect in UV–vis spectra can be used to monitor the occurrence transition‐metal chelating pathway [17, 83, 85, 86, 123, 124, 125, 126, 127, 131].

(3) Despite that this transition‐metal chelating pathway has been indicated as one antioxidant pathway [1, 4], after all, it cannot mediate radical‐trapping to break the chain reaction, thus it is sometimes called the indirect antioxidant mechanism. Correspondingly, those radical‐trapping reactions are called the direct antioxidant mechanism [26, 28] (Figure 7).

## 3 Structural Factors in Phenolic Antioxidants in CHMs

Phenolics from CHMs are a biological consequence of plant metabolism. To a certain extent, their structures differ with the synthetics, for example, organometallic compound and fullerene derivatives. In other words, these phenolics are structurally characteristic and mostly include catechol and hydroxyquinone moieties, C═C bonds, stereochemical configurations, and various substituents (Table S1).

### 3.1 Hydroxyquinone and Catechol Moieties

Although the antioxidant activity comes from phenolic –OH, the phenolic –OH arrangement is more important than its quantitation. Previous studies have suggested that two arrangements basically dominate the antioxidant capacity of phenolics—the hydroxyquinone and catechol moieties [89, 91].

**SCHEME 6:**
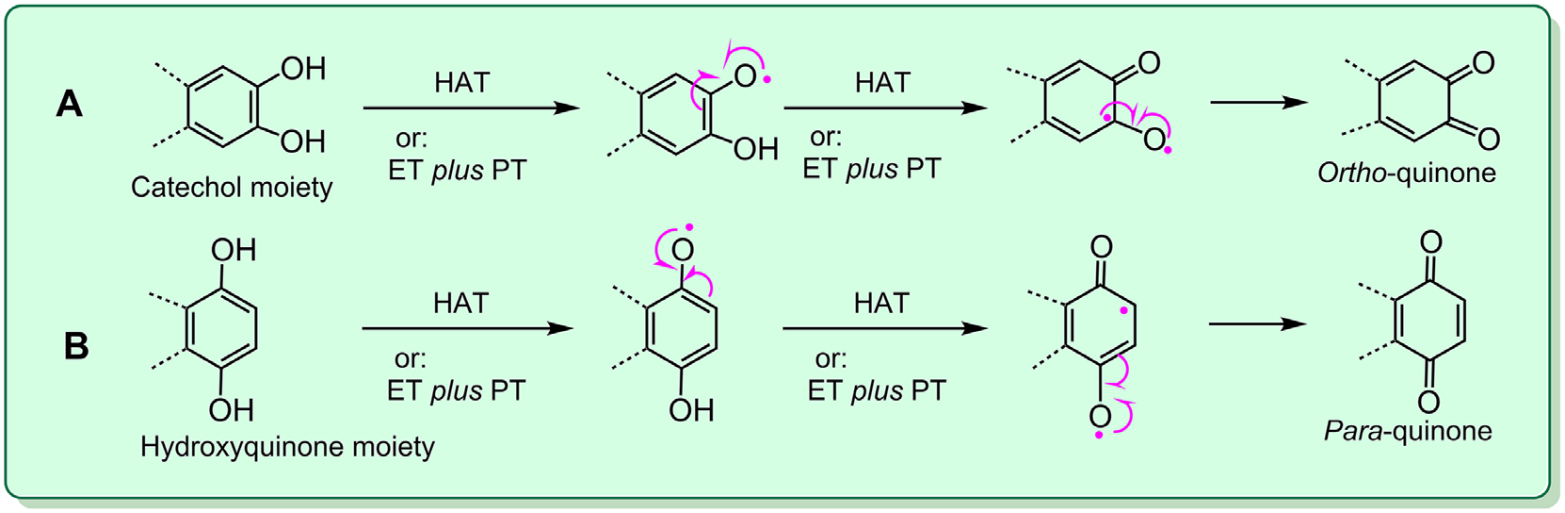
Oxidization reactions of hydroxyquinone and catechol moieties into quinones.

The catechol moiety is present in many various phenolics and has been extensively reported in the past decades. The reason the catechol moiety possesses strong antioxidant activity is that catechols can undergo HAT (or ET *plus* PT) pathways to give rise to *ortho*‐quinones, which are stable oxidation products (Scheme 6A) [86, 89].

Compared with the catechol moiety, however, hydroxyquinones have received much less interest from researchers [18, 21, 26, 28], possibly because of their rarity. For example, among thousands of flavonoids, there are a few that possess a hydroquinone moiety, for example, rhodionin, 5,8‐dihydroxy‐3,6,7‐trimethoxyflavone, 5,8‐dihydroxy‐6,7‐dimethoxyflavone, 5,8‐dihydroxy‐6,7,4′‐trimethoxyflavone, 5,8‐dihydroxy‐6,7,3′,4′,5′‐pentamethoxyflavone, and isothymusin [89, 132]. The previous work indicated that a hydroxyquinone moiety plays a similar role to that of a catechol moiety [89, 133]. This is likely because it can be oxidized to a stable quinone, that is, *para*‐quinone (Scheme 6B). Theoretical calculations suggest that catechol and hydroxyquinone moieties possess similar BDE values (81.8 and 81.9 kcal/mol, respectively) and inhibition rate constants (5.5 × 10^5^ M^−1^ s^−1^ and 4.0 × 10^5^ M^−1^ s^−1^, respectively) [26, 28]. Briefly, the stability of *ortho*‐quinone and *para*‐quinone may be responsible for the strong antioxidant activity of catechol and hydroxyquinone moieties, respectively.

### 3.2 C═C Bond and π–π Conjugation Effect

The C═C bond is also present in many phenolic antioxidants found in CHMs, particularly stilbenes, cinnamic acid derivatives, chalcones, flavonoids, and phenolic glucosides [2, 11, 134] (Table S1). Two oligo‐stilbenes α‐viniferin and caraphenol A are typical examples. The two coexist in the identical plant *Caragana sinica* [124]. When the α‐viniferin is introduced to a C═C bond, it converts into caraphenol A. Compared to α‐viniferin, caraphenol A has greater π–π conjugation and, accordingly, produces higher antioxidant levels (about 4.58‐fold) [124]. These comparisons base on coexisting analogues can provide a clue that, the introduction of C═C can sometimes bridge two large groups to form a more extensive conjugate system, to affect the antioxidant levels. This clue will further help to understand the possible antioxidant difference between 1‐*O*‐*p*‐hydroxybenzoyl‐β‐D‐apiofuranosyl‐(1→6)‐β‐D‐glucopyranoside and 1‐*O*‐caffeoyl‐β‐D‐apiofuranosyl‐(1→6)‐β‐D‐glucopyranoside, two coexisting analogues in the CHM *Danshen* [2].

Besides the introduction of C═C, the position of the C═C bond can also affect antioxidant ability. A typical example is the pair of moracin C and *iso*‐moracin C, which display different antioxidant potentials [88].

In a word, the C═C bond can mediate conjugation to influence phenolic antioxidant levels. Finally, the planar C═C is well known to present possible *Z*/*E* geometrical configuration. Whether the *Z*/*E* geometrical configuration influences antioxidant level, remains to be discussed in Section 4.2.

### 3.3 Chiral Carbon and Stereochemical Configuration

A chiral molecule is defined as one which is non‐superposable on its mirror image (i.e., enantiomer). Regularly, chiral molecule requires the presence of chiral carbon. However, the phenyl ring (or phenyl‐fused ring) possess planar configuration to be of no chiral carbon. Accordingly, some simple phenolics usually are not chiral, such as vanillic acid [20], mono‐stilbene resveratrol [63], and mono‐coumarin demethylsiderin [97].

**SCHEME 7:**
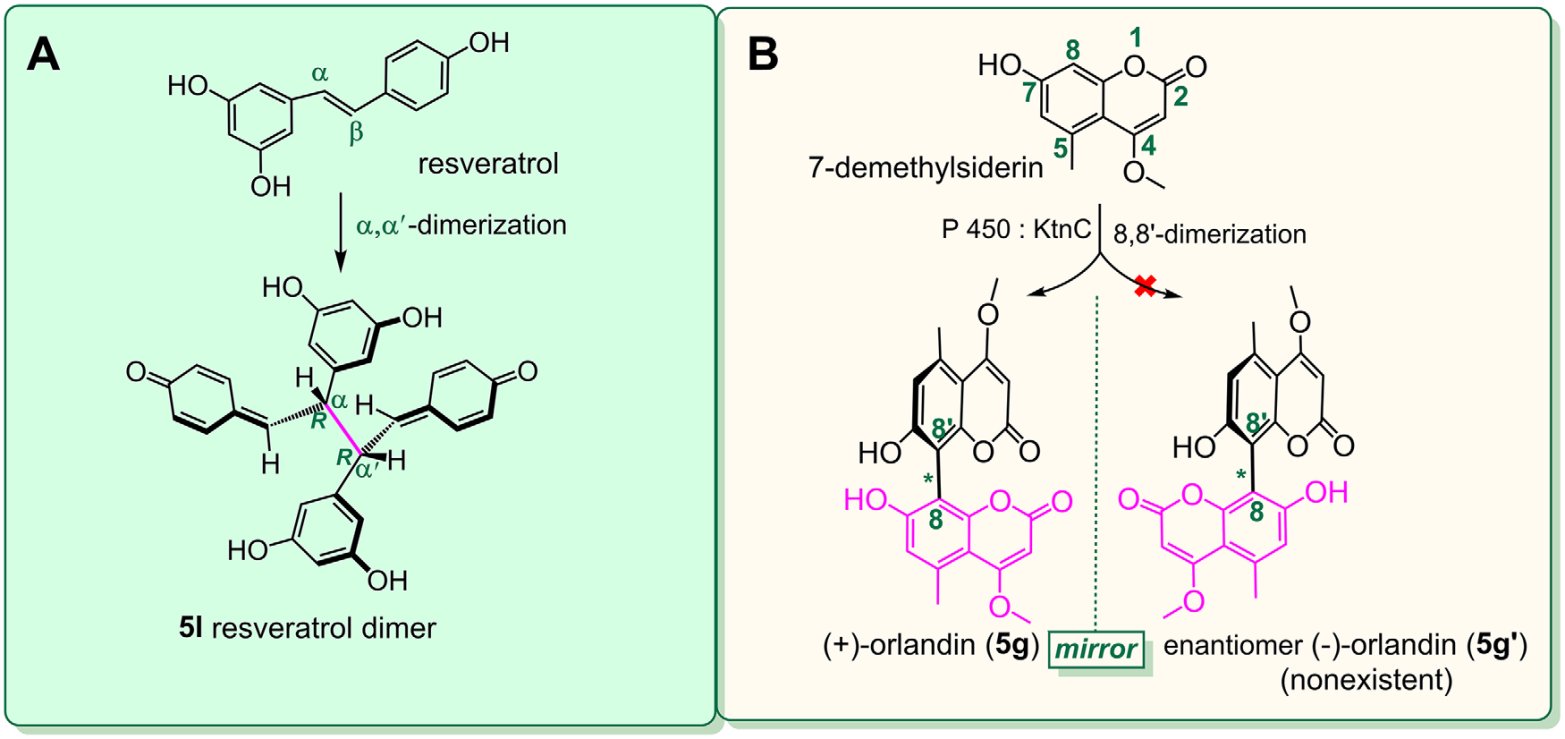
Production of chiral dimer via RAF pathway.

However, RAF‐mediated oligomerization (especially dimerization) may make their oligomers to be chiral. For example, resveratrol is a nonchiral phenolic from CHMs [135, 136, 137]. Once it dimerizes via a covalent bond, it produces a chiral dimer (**5l**) with (*αR,α’R*) absolute configuration (Figure 5 and Scheme 7A) [63]. Another example is dimerization of coumarin demethylsiderin. As seen in Scheme 7B, demethylsiderin molecule has no chiral carbon and is non‐chiral one. However, under the catalysis of cytochrome P450 enzyme, it can experience RAF pathway to form one chiral coumarin dimer (i.e., (+)‐orlandin **5g**). This dimer is non‐superposable on its enantiomer (‐)‐orlandin (**5g′**) and thus is chiral, regardless the absence of chiral carbon. Interestingly, such enzymatic biosynthesis has actually yielded no (‐)‐orlandin (**5g′**). Thus, this biosynthesis possesses high stereoselectivity (Scheme 7B) [96, 97].

In addition to oligomerization, exocyclic chiral carbon may also cause CHMs‐derived phenolics to be chiral. Typical examples are tea‐catechins [18, 37, 138]. The basic skeleton of tea‐catechins is catechin, which possesses two chiral carbons (i.e., 2‐carbon and 3‐carbon). When catechin displays the 2*R*,3*S* absolute configuration, it is conventionally called (+)‐catechin [139, 140]. Correspondingly, its diastereomer 2*R*,3*R*‐catechin is called (‐)‐epicatechin (or epicatechol, **EC**) [141]. In this study, the prefix “epi” means that the 2‐substituent and 3‐substituent are arranged on the same side of the fused ring. Apparently, the prefix “epi” in tea‐catechins indicates that a chiral carbon is responsible for increasing the amounts of phenolic antioxidants present.

It has been suggested that some tea‐catechins could coexist in Tibetan tea, an essential beverage of the Tibetan people. These catechins included (+)‐catechin, (‐)‐catechin gallate (**CG**), (‐)‐epicatechin gallate (**ECG**), and (‐)‐epigallocatechin gallate (**EGCG**). Such enrichment of tea‐catechins enhances the strong antioxidant activity of tea, thus allowing tea to effectively release cellular oxidative stress [87, 142]. This beneficial effect is termed *“Jiedu”* (meaning detoxification) in TCM. In fact, tea has already been documented as antitoxic in ancient Chinese books [1].

Taken together, oligomerization and exocyclic chiral carbon can make CHMs‐derived phenolics to be chiral. The chirality however may bring about enantiomers or diastereomers, and thus increase the phenolic amounts. The enrichment of chiral phenolics can further help CHMs to synergistically exert the antioxidant action or clinical effects. Of course, in the same chiral phenolic molecule, different stereochemical configurations may also affect the antioxidant action; this will be further discussed at Section 4.9.

### 3.4 Various Substituents

#### 3.4.1 Isoprenyl, Cyclized‐Isoprenyl, and 3‐Hydroxy‐3‐methylbutyl Groups

It is well known that isoprene (i.e., 2‐methyl‐1,3‐butadiene) is the basic skeleton of terpene and natural rubber. During plant metabolism, isoprenyl (also called prenyl [134]) can also attach to the skeletons of xanthone [64], flavonoid, chalcone [143], stilbene, lignanoid [144], Diels–Alder adducts [134, 145], 2‐phenyl‐benzofuran [146, 147], coumarin [148], and unusual phytophenols [149]. In particular, in the xanthone family, about 50% of the members contain an isoprenyl group [134]. Thus, isoprenyl can be considered a characteristic substituent of phenolic xanthones. A latest study revealed that isoprenylation can slightly promote both the ET potential and RAF potential [134]. Similarly, the cyclized‐isoprenyl group and 3‐hydroxy‐3‐methylbutyl group (also called the isopronyl group) can slightly affect the redox‐based antioxidant potentials (e.g., ET), unless the catechol (or hydroxyquinone) moiety is destroyed [89, 91].

#### 3.4.2 Sugar Residues

Glycosidation by sugar residues is also a biological consequence of plant metabolism and can be readily seen in phenolic antioxidants (except for volatile phytophenol and Diels–Alder type adducts) [134]. Glycosidation can introduce a large sugar residue, thereby hindering the RAF pathway, which can slightly enhance the metal‐chelating pathway [68, 123]. Sometimes glycosidation can break down the phenolic –OH to disrupt the catechol (or hydroquinone) moiety, which greatly reduces the antioxidant capacity [89].

Our previous work indicated that even the type of sugar residue can affect the antioxidant level. An available example is the pair of isoquercitrin and quercitrin. Isoquercitrin linked with glucoside has proven to be a stronger antioxidant than quercitrin with rhamnoside. This difference can be attributed to the 6″–OH group, which can enhance the ET and Fe^2+^‐chelating abilities of isoquercitrin [17].

#### 3.4.3 Methoxy and Alkoxy Groups

In many phenolic antioxidants from CHMs, a methoxy group is observed to be directly attached to the benzene ring [28, 134], to construct so‐called guaiacol moiety [35]. The methoxy group is able to donate an electron through *p*‐π conjugation, thereby improving the ET potential of phenolics [150]. On the other hand, an *ortho*‐methoxyl can lower the antioxidant ability via IHBs [28, 151]. On a whole, the enhancement effect of a methoxy group is slight. This can be further verified by the comparative experiments between subelliptenone G and bellidifolin [42], between hesperetin and naringenin [24, 152], and from the previous findings that guaiacol moiety exhibits a similar ^•^O_2_^−^ ‐scavenging rate to phenol [153].

Compared with the methoxyl, other alkoxy groups (e.g., ethoxyl and propoxyl) are quite rare in phenolics. Succinctly, the effect of methoxy or alkoxy groups on antioxidant ability is only slight, whether an enhancement or weakness.

#### 3.4.4 Galloyl, Caffeoyl, and *p*‐Coumaroyl Groups

Galloyl, caffeoyl, and *p‐*coumaroyl groups have been observed in phenolic antioxidants [2, 35, 154, 155, 156]. Recent studies suggested that they can improve antioxidant or cytoprotective effects to a great extent [123, 125, 157, 158], because these groups themselves possess high antioxidant activity. Obviously, this provides a structural modification approach for improving the antioxidant levels of phenolics isolated from CHMs. However, pyrogallolation may cause pro‐oxidation [159] because the pyrogallol moiety generates a superoxide anion radical (^•^O_2_^−^) at high pH values [20].

## 4 Characterization of Various Phenolic Antioxidants

Although volatile phytophenol, phenolic acid, and their glucoside in the CHMs were also reported to show antioxidant ability (e.g., [6]‐gingerol [160]), however, their structures were greatly different from each other, as shown in Table S1. Thus, their antioxidant structure–activity relationship could not be analyzed and summarized. Other antioxidants, however, would be further discussed for their characteristics, including stilbene, dihydrochalcone, flavonoid, 2‐phenyl‐benzofuran, xanthone, carotenoids, curcumin, phenolic alkaloids, Diels–Alder‐type adducts, and phenolic hybrids.

### 4.1 Stilbene and its *Z*/*E* Configuration Effect

**FIGURE 8:**
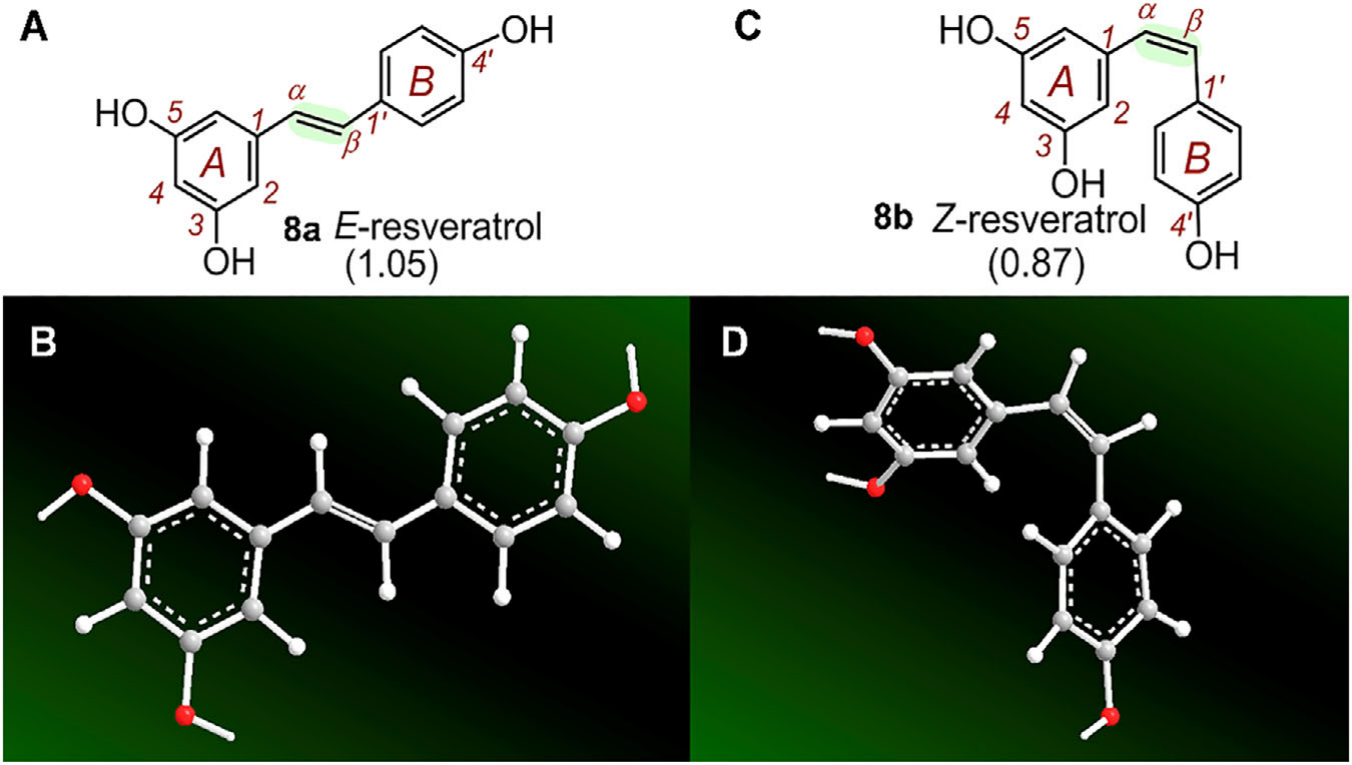
The configuration formula and molecular models of two resveratrols: (A) configuration formula of *E*‐resveratrol; (B) preferential conformation of *E*‐resveratrol; (C) configuration formula of *Z*‐resveratrol; (D) preferential conformation of *Z*‐resveratrol. The values in parentheses are the TEAC based on a DPPH•‐scavenging assay and obtained from the literature [163].

From CHMs, at least 400 stilbenes have been isolated, including mono‐stilbene and oligo‐stilbene [161, 162] Both stilbenes possess the same basic skeleton, that is, 1,2‐diphenylethene (green in Figure 8A,B) [124]. One well‐known stilbene is resveratrol (3,5,4′‐trihydroxy‐stilbene), which is found in many CHMs (Table S1) in the form of *E*‐resveratrol (**8a,** Figure 8A) [11, 35, 63]. An α,β‐substituted C═C bond can also yield the *Z*‐configuration (**8b,** Figure 8C). Thus, there are actually two resveratrols: *E*‐resveratrol and *Z*‐resveratrol.

Our study suggested that the two geometrically inequivalent configurations of resveratrol exhibit great differences in antioxidant ability. This difference arises from the alteration of functional group interactions. As illustrated in Figures 8A,B, E‐resveratrol positions its two rings at either side, effectively relieving the steric crowding; in this configuration, the two rings share a plane. In *Z*‐resveratrol, however, the two rings are positioned on the same side; this molecule is very crowded. To relieve the steric crowding, each of the two rings rotate slightly about the σ‐bond. Therefore, the two rings do not share a plane, and the *Z*‐resveratrol contains a dihedral angle (Figure 8D). Nonplanarity weakens the π–π conjugation degree and thereby reduces the molecule's antioxidant ability [163]. Thus, *Z*‐resveratrol (TEAC of 0.87) has a lower antioxidant activity than *E*‐resveratrol (TEAC of 1.05) (Figure 8A,C).

In summary, geometric configuration profoundly affects the antioxidant levels of phenolics bearing a C═C bond, despite the seemingly simple exchange of *Z*/*E* configurations. This likely explains why only *trans*‐piceid (i.e., *E*‐piceid) exists in nature, and why the antioxidant levels are so different between *trans*‐*ε*‐viniferin and *cis*‐*ε*‐viniferin and between maximol A and maximol B [82] (Table S1).

### 4.2 Dihydrochalcone and Glycosidation Effect on RAF Potential

Like stilbene, chalcone (including dihydrochalcone) can also be formed via biosynthesis in plants [164, 165, 166]. What is more, stilbene and chalcone usually stem from the same precursors (such as *p*‐coumaroyl‐CoA and malonyl‐CoA) [167]; thus, they occasionally coexist in the same plants [168].

**FIGURE 9:**
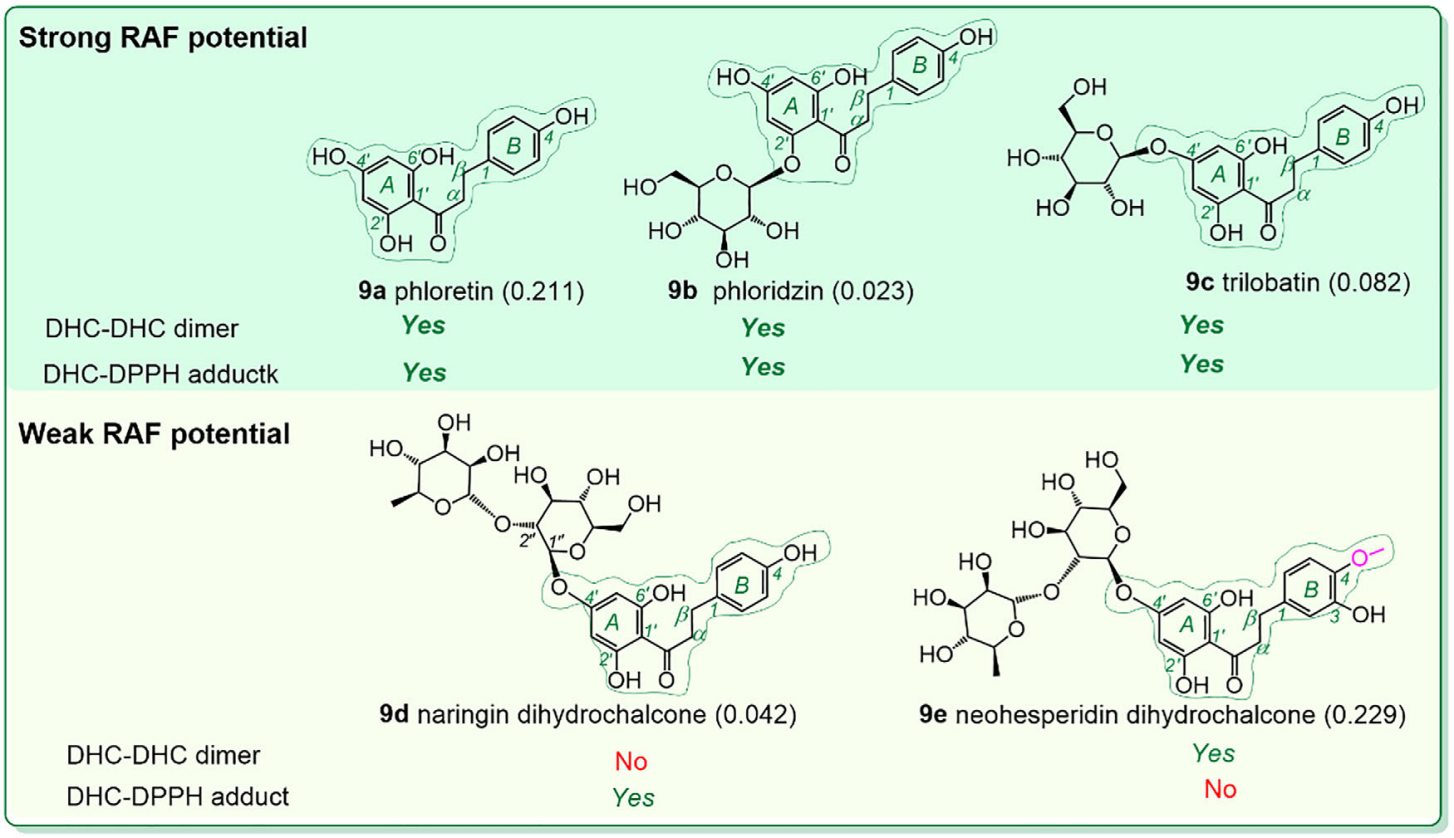
The RAF potentials and TEAC values of reference dihydrochalcones (DHC) from CHMs. The TEAC values are based on a DPPH^•^‐scavenging assay and obtained from the literature [68].

Antioxidant chalcones have been summarized in some literature articles [169, 170]. In the previous study, five dihydrochalcones (**9a–9e**, Figure 9) were selected as references and were shown to display relatively low antioxidant potentials (TEAC = 0.023–0.229) [68]. This may be because α,β‐hydrogenation blocks the conjugation between the *B‐*ring and the keto group (Figure 9).

Nonetheless, the RAF potentials of dihydrochalcones can be evidenced by UPLC‐ESI‐Q‐TOF‐MS/MS. As seen in Figure 9, the RAF potentials of the studied dihydrochalcones were found to be quite different. Each of the former three (**9a–9c**) can yield both dihydrochalcone–dihydrochalcone dimer and a dihydrochalcone‐radical adduct. By comparison, each of the two latter (**9d** and **9e**) can only yield either dihydrochalcone–dihydrochalcone dimer or the dihydrochalcone‐radical adduct [68]. This clearly suggests that glycosylation can reduce the RAF potential via steric hindrance and may explain the previous findings that three antioxidant phenylpropanoids (acteoside, poliumoside, and forsythoside B) have no RAF products [123]. These phenylpropanoids possess a great hindrance from the glucose, rhamnose, or apiose residues.

### 4.3 Flavonoids and Advances in Antioxidant Studies

**FIGURE 10:**
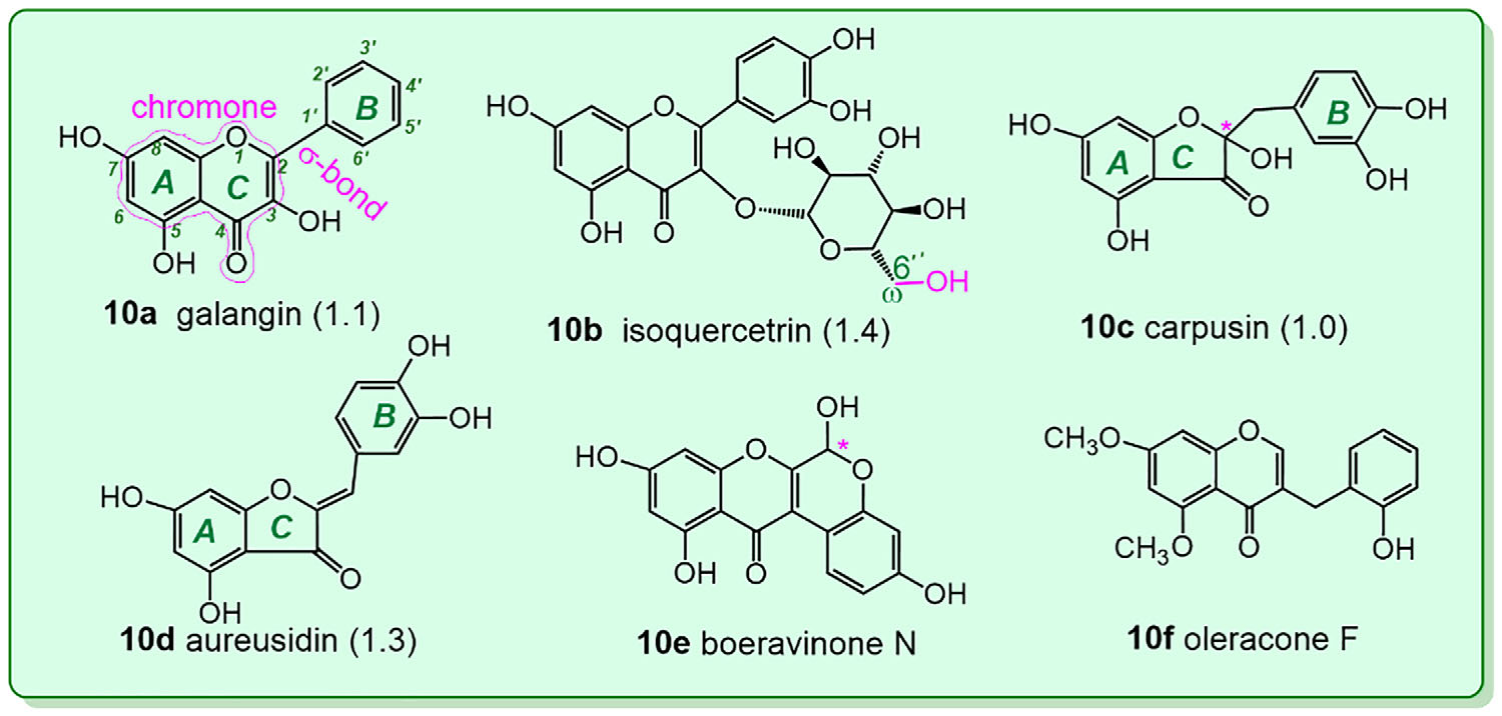
Flavonoids from CHMs. The drawing of the carpusin structure in the original literature is incorrect [175], and the absolute configuration of the chiral carbon (*) remains unclear. The TEAC values were obtained from the literature [17, 84, 175, 176, 177].

During plant metabolism, a chalcone skeleton may cyclize to form a new ring, which is conventionally numbered as the *C‐*ring. The *C‐*ring fused with the *A‐*ring constructs a chromone fused ring. Between the chromone fused‐ring and *B‐*ring, there is a *σ*‐bond to build up a typical flavonoid skeleton (e.g., **10a**, Figure 10) [171]. Such typical flavonoid skeleton however can derive several usual subtypes, including flavone, isoflavone, flavonol, isoflavonol, dihydroflavone, dihydroisoflavone, biflavonoid, and their corresponding glucosides (e.g., **10b**) [134, 172]. To date, various flavonoids have been successfully isolated and identified from CHMs or herbal medicines [162, 173, 174].

Most of these flavonoids can be attached by phenolic ‐OH and act as phenolic antioxidants [16, 18, 173, 178]. In the last few decades, extensive efforts have been devoted to understanding their antioxidant chemistry, especially the antioxidant mechanisms and antioxidant structure–activity relationships. The antioxidant mechanisms of phenolic flavonoids have been suggested to involve Fe^2+^‐chelation [116, 118, 126, 127], HAT [179], SPLET [179], PCET [180], SET‐PT (or SEPT) [179], and RAF [46].

Systematic structure–activity relationship analyses have demonstrated that the antioxidant levels of flavonoids can be affected by various functional groups or substituent groups [35, 87, 89, 181, 182, 183, 184, 185, 186, 187, 188, 189, 190, 191, 192, 193, 194, 195]. Even an ω‐OH (6″‐OH) group could influence the HAT, ET, and Fe^2+^‐chelating potentials of a flavonoid glycoside (**10b**) [17]. In addition to the functional group or substituent group, the flavonoid skeleton has also been investigated recently. It was observed that the null *B‐*ring could improve the antioxidant levels, and such improvement was associated with the π–π conjugation between the chromone fused‐ring and the *B‐*ring [17, 84].

If a five‐membered ring replaces the *C*‐ring in the typical flavonoid skeleton, such skeleton is called as aurone [175, 176]. Aurone however is an usual subtype in flavonoids family and can also function as phenolic antioxidants [196]. Previous study revealed that, carpusin (**10c**), an aurone from *Rheum emodi* (ZangbianDahuang, one kind of Himalayan Rhubarb), exhibited substantial antioxidant potential (TEAC 1.0) [175]. Given it proceeds a dehydration reaction, carpusin may transform into another aurone aureusidin (**10d**), a more powerful antioxidant [176] (TEAC 1.3, Figure 10). According to the above discussion, such improvement may be beneficial from the extension of π–πconjugation. This however is further supported by the evidence from synthetic aurones [196].

Similar to aurone, rotenoids are also an unusual subtype in flavonoids family. Recently, some rotenoids have also been isolated from the CHM *Laolaiqing (Boerhavia diffusa* L.) [177]. However, major of them contain only one phenolic ‐OH and thus are presumed to be weak antioxidants. Exceptionally, rotenoid boeravinone N (**10e**) possesses three phenolic ‐OH. However, the three do not construct either hydroxyquinone moiety or catechol moiety (Figure 10). It is thus presumed to be a weak antioxidant as well. These can partially explain why there has been no antioxidant report of rotenoids.

In addition to aurone and rotenoid, homoisoflavone is also unusual in flavonoids family. Recently, a new homoisoflavone, oleracone F (**10f**), was isolated from the CHM *Machixian* (*Portulaca oleracea* L.) and shown to possess antioxidant potential [197]. This suggests homoisoflavone subtype as a potential library of natural antioxidants.

### 4.4 2‐Phenyl‐benzofuran and Exocyclic C═C Bond Effect

**FIGURE 11:**
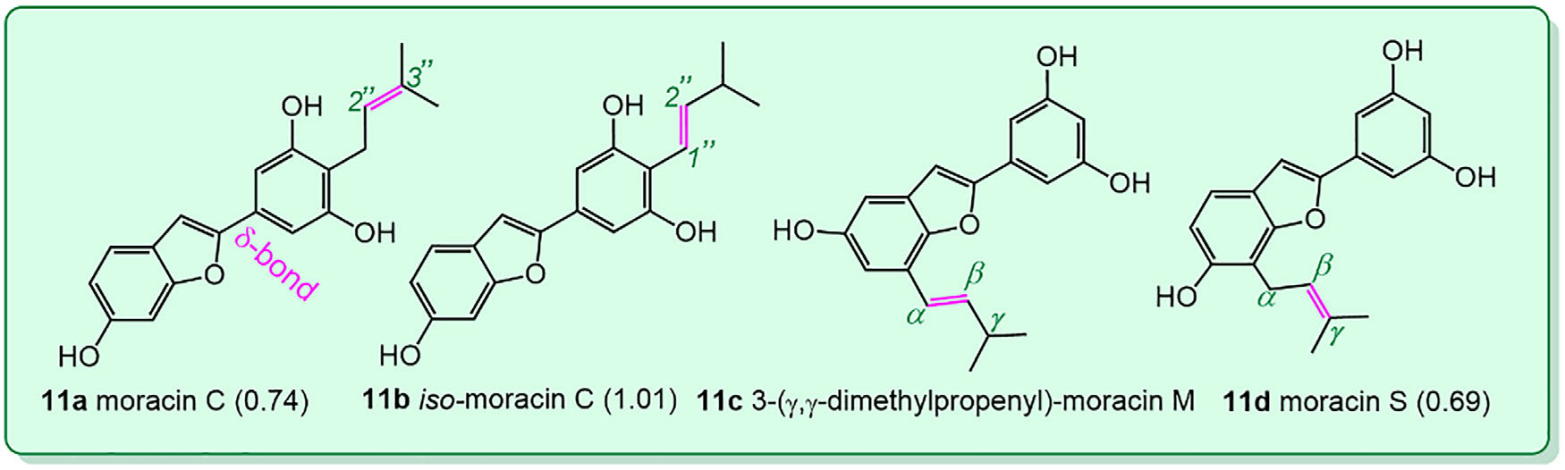
2‐Phenyl‐benzofuran derivatives from CHMs. The values in parentheses are the TEACs (based on a DPPH•‐scavenging assay) obtained from the literature [88, 199, 200, 201].

Similar to typical flavonoids, 2‐phenyl‐benzofuran also links a phenyl ring and a fused ring through a rotatable σ‐bond (e.g., **11a,** Figure 11). Most of 2‐phenyl‐benzofuran derivatives are found in CHMs from the *Moraceae* and *Gnetaceae* family (Table 1s). Moracin C (**11a**) and *iso*‐moracin C (**11b**) are two representatives [88, 198]. In the 2‐phenyl‐benzofuran derivatives, there are abundant exocyclic C═C bonds; some of them are conjugative (e.g., **11b** and 3‐(γ,γ‐dimethylpropenyl)‐moracin M, **11c**), whereas some are not (e.g., **11a** and moracin S, **11d**) (Figure 11) [199, 200, 201]. The previous study suggested that *iso*‐moracin C, with a conjugative exocyclic C═C bond, has a higher TEAC value than moracin C, with a non‐conjugative exocyclic C═C bond (Figure 11) [88].

The different roles of exocyclic C═C bonds however further emphasize the importance of conjugation (see: Section 3.2). Furthermore, their different roles may also explain why chalcones and stilbenes possess strong antioxidant abilities, and why (iso)prenyl substituents do not greatly affect the antioxidant properties of phenolics. In chalcones and stilbenes, the exocyclic C═C bond is conjugated with the aromatic skeleton, whereas the exocyclic C═C bond in an (iso)prenyl substituent is not.

### 4.5 Xanthone and Antioxidant Rule

Unlike a flavonoid, or 2‐phenyl‐benzofuran, a xanthone has no rotatable σ‐bond linking a phenyl ring; hence, the xanthone framework is rigid. In addition, the planar γ‐pyrone ring is symmetrically fused with two phenyl rings in the same plane (e.g., 1,3,5,8‐tetrahydroxyxanthone, **12a**). As such, the xanthone skeleton is planar, symmetrical, and rigid. Such structural characteristics impart a very definitive antioxidant structure–activity relationship.

**FIGURE 12:**
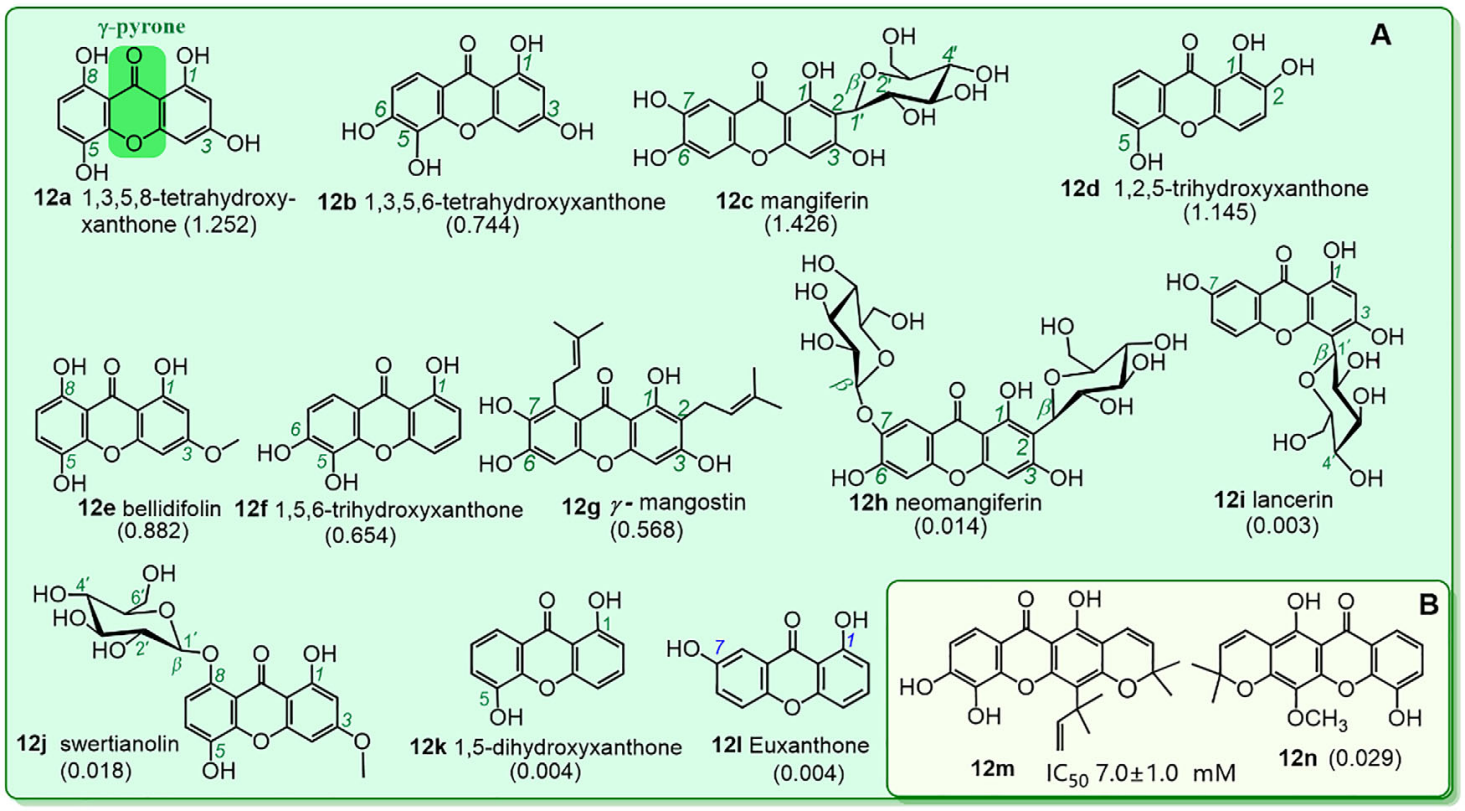
Xanthones from CHMs: (A) for structure–activity relationship analysis; (B) for prediction. The values in parentheses are the TEACs obtained from the literature [91, 202, 203].

Our study revealed that the hydroquinone moiety (e.g., 1,3,5,8‐tetrahydroxyxanthone, **12a**) and three catechol moieties—namely, the 5,6‐catechol (e.g., 1,3,5,6‐tetrahydroxyxanthone, **12b**), 6,7‐catechol (e.g., mangiferin, **12c**), and 1,2‐catechol (e.g., 1,2,5‐trihydroxyxanthone, **12d**) moieties—always govern the antioxidant capacity of the xanthone family [42, 89, 90]. Thus, once the hydroquinone (or catechol) moieties are disrupted, the antioxidant capacities greatly decrease. The governing role of the hydroquinone moiety, for example, can be found by comparison between 1,3,5,8‐tetrahydroxyxanthone (**12a**), bellidifolin (**12e**), and swertianolin (**12j**). As shown in Figure 12, **12e** is the 3‐*O*‐methyl ether of **12a**. This methylation eliminates the resorcinic 3‐OH. Hence, its TEAC value does not decrease significantly (1.252–0.882). However, if **12e** is further glycosided at the 8‐*O*‐position, the hydroquinone moiety is lost and the TEAC value remarkably decreases 49‐fold (from 0.882 to 0.018). Another example is the conversion of mangiferin (**12c**) to neomangiferin (**12h**). As shown in Figure 12, this structural change dramatically lowers the TEAC value 100‐fold (from 1.426 to 0.014); this is because the 6,7‐catechol moiety in **12c** is broken via 7‐*O*‐glycosidation. The above structure–activity relationship (especially the governing roles of the hydroquinone and three catechol moieties) was also observed in the ferric ion reducing antioxidant power (FRAP) assay based on the ET potential [42, 193].

These antioxidant structure–activity relationships can be used to directly predict the antioxidant level of any xanthone according to the drawn structural formula. For example, a newly found 5,6‐catecholic xanthone (**12m**) exhibits a higher antioxidant level (IC_50_ of 7.0 ± 0.1 µM, TEAC of ∼1.9) [203]. However, another newly found xanthone **12n** (Figure 12) should exhibit a much lower antioxidant level (TEAC of 0.029) [202] due to the lack of a hydroquinone or catechol moiety. Obviously, the accuracy of the prediction can be attributed to the planarity, symmetry, and rigidity of xanthone skeleton.

This type of accurate prediction should also be possible for transannular di‐OH moiety, a special antioxidant group. Di‐OH naphthalene derivatives (i.e., naphthalene diols [204]) are simple examples.

**FIGURE 13:**
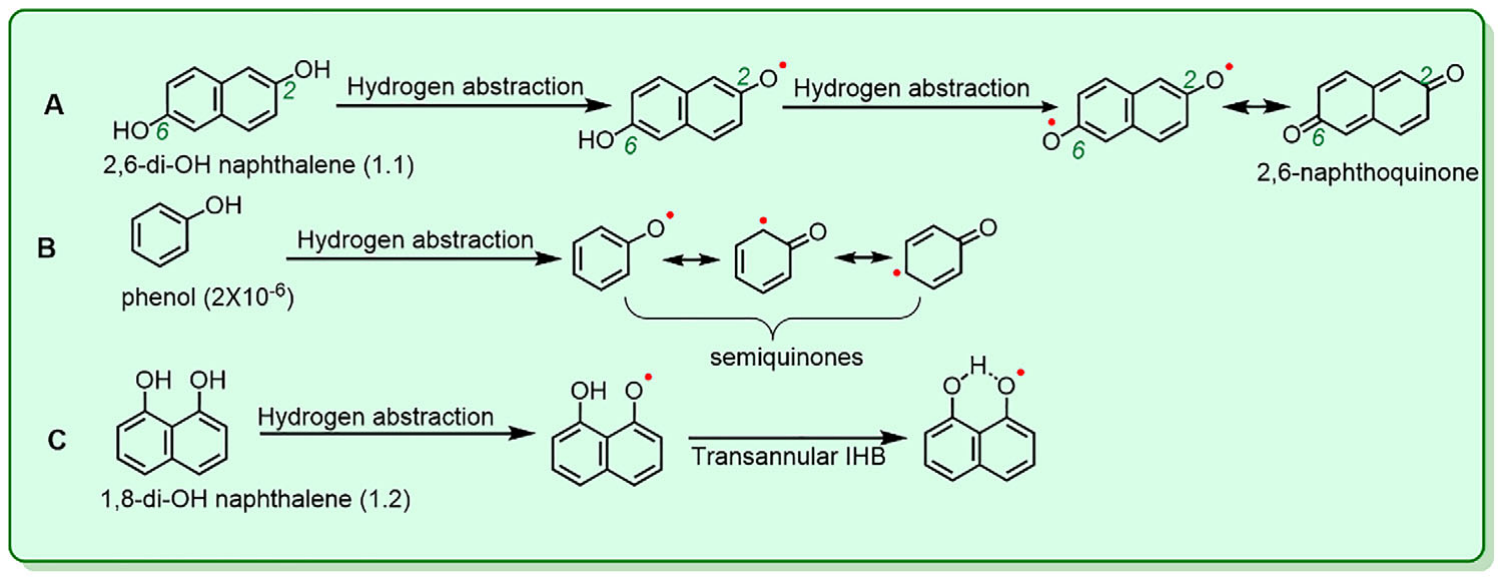
The hydrogen‐abstraction reactions of 2,6‐di‐OH naphthalene (A), phenol (B), and 1,8‐di‐OH naphthalene (C).

As shown in Figure 13A, 2,6‐di‐OH naphthalene (2,6‐naphthalenediol) possesses only two phenolic ‐OHs and two phenyl rings. On average, one phenyl ring contains one phenolic ‐OH. Seemingly, this is similar to phenol itself. However, the two phenolic ‐OHs can conjugate through the aromatic rings. Thus, if 2,6‐naphthalenediol undergoes hydrogen abstraction by free radicals, it would be converted into the 2,6‐naphthoquinone, a large conjugative system. Accordingly, 2,6‐naphthalenediol exhibits stronger antioxidant ability than that of phenol. The reason why phenol exhibits so less ability, is that it cannot be converted into stable quinone forms via hydrogen‐abstraction reactions. Its oxidized product is unstable semiquinone (Figure 13B).

Similarly, 1,8‐di‐OH naphthalene also exhibit strong antioxidant ability (Figure 13C). This has been reported to be attributed to its transannular IHB, which can stabilize the ArO^•^ radical [204].

**SCHEME 8:**
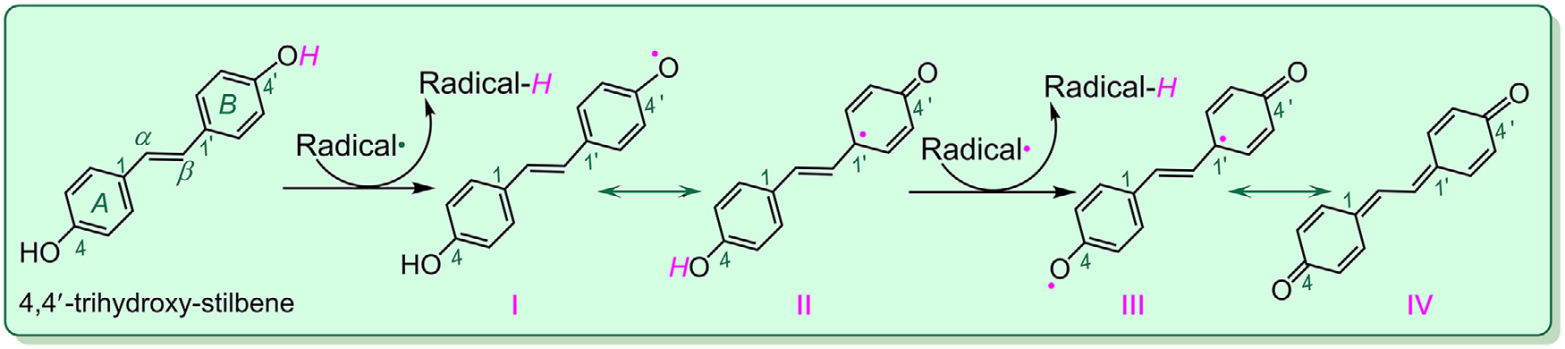
Reaction of 4,4′‐dihydroxy‐stilbene with excessive free radicals.

The third simple example is 4,4′‐dihydroxy‐stilbene. As shown in Scheme 8, 4,4′‐dihydroxy‐stilbene bears a transannular di‐OH moiety. Given that 4,4′‐dihydroxy‐stilbene losses two hydrogen atoms via free radical abstraction to give rise to the final product (i.e., quinone IV). Because the quinone (IV) molecule is virtually a large conjugative system, thus it presents high stability. Accordingly, its precursor 4,4′‐dihydroxy‐stilbene is assumed to be a strong antioxidant. This has been evidenced by a human erythrocyte experiment, where 4,4′‐dihydroxy‐stilbene exhibited remarkably stronger peroxidation‐inhibitory effect than resveratrol [205].

It is currently clear that (1) antioxidant understanding (or prediction) should not be merely based on the number of phenolic ‐OH groups, because their arrangement plays a more important role; (2) antioxidant understanding (or prediction) based on a certain arrangement (such as a hydroquinone or catechol moiety with strong antioxidant ability) is sometimes incomplete; and (3) complete and accurate antioxidant understanding (or prediction) must consider the whole molecule. If a molecule can give rise to a stable form after oxidization by a free radical, then it will exhibit strong antioxidant ability. In other words, the more stable the whole oxidized molecules become, the higher the levels of antioxidant properties will be. The stability of the whole molecule, of course, depends on the degree of conjugation, which, to some extent, is consistent with the holism theory of TCM. For the convenience of discussion, it is called as a qualitative antioxidant rule.

Using the qualitative rule based on holism theory, it can be understood why both **12k** and **12l** exhibited so low an antioxidant level (TEAC of 0.004, Figure 12) [90, 91, 99]: because the relative position of transannular di‐OH prevented the formation of the corresponding quinone with relative stability.

### 4.6 Carotenoids, Curcumin, and Antioxidant Rule Explanation Using BDE Values

**FIGURE 14:**
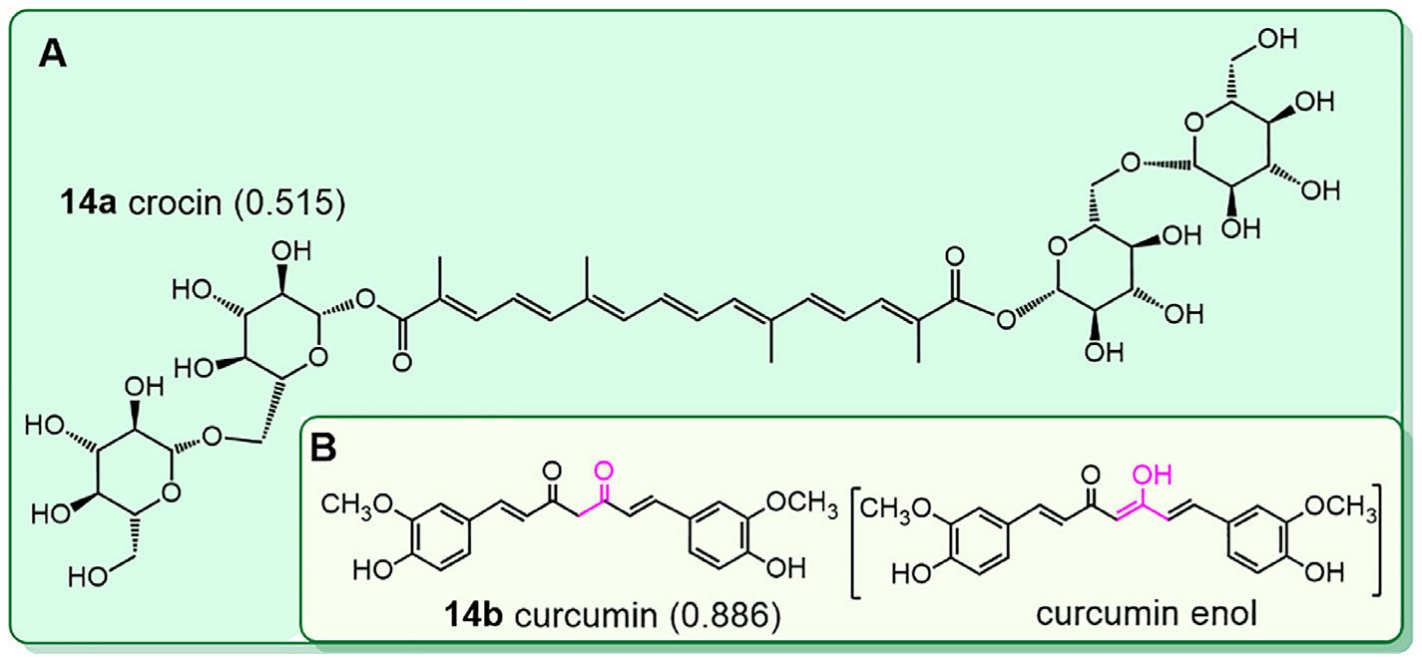
Structures and available TEAC values of crocin and curcumin. The TEAC values in parentheses were obtained from the literature [210, 211, 212, 213].

Several carotenoids with polyenes (but without phenolic ‐OHs), including crocin (**14a,** Figure 14), astaxanthin, crocetin, all‐*trans*‐retinol [206], lycopene [207], and anthoxanthin [208] (SI‐2 Table s1) have been isolated from some tonifying CHMs (e.g., *Gouqizi* and *Huluobo*). Similar to phenolic antioxidants, these carotenoids can also mediate HAT, or ET *plus* PT to exhibit antioxidant activity [8, 9, 209].

Particularly, crocin has appreciable antioxidant potential (TEAC of 0.515 [210, 211]) and can reduce ultraviolet B‐induced intracellular ROS [214]. This can be attributed to its low C─H BDE values (79.0–86.3 kcal/mol [209]), which are similar to phenolic O─H (ArO─H) BDE values (77.0–86.7 kcal/mol) [26, 28]. Its low C─H BDE value is due to its large conjugative system, which can stabilize the crocin^•^ radical.

A similar phenomenon was also observed for curcumin and other linear carotenoids with extensive conjugation [215]. As shown in Figure 14B, curcumin (especially its enol form, **14b**) contains not only conjugative polyenes, but phenolic ‐OHs as well, and can therefore mediate similar antioxidant pathways to display high antioxidant effects (TEAC of 0.886, Figure 14) [212, 213].

By comparison, the alcoholic O─H BDE values (88.3–91.6 kcal/mol) are significantly higher than those of crocin (a representative polyene) [209]. Thus, polyene C─H, phenolic O─H, and polyene‐conjugated phenolic O─H can similarly exhibit antioxidant ability by undergoing HAT; alcoholic O─H, however, has no similar antioxidant capacity.

In other words, conjugation effectively decreases the phenolic O─H BDE to below a threshold value (e.g., ∼88 kcal/mol for ROO‐H and 78.9 kcal/mol for DPPH‐H) [26, 65]. When one molecule shows a lower BDE value than the threshold value, it may easily donate a H atom to present antioxidant potential. Accordingly, the kinetic *k* value (rate constant) also improves [28, 64, 65]. Thus, a comparison of BDE values can be used to predict whether a molecule has antioxidant potential. This can be considered a quantitative antioxidant rule [28, 39].

This rule implies that there is no absolute gap in antioxidant potential between a phenolic moiety and polyene moiety. The difference lies merely in the BDE values. However, the BDE values are known to be affected by relevant structural factors (especially, larger conjugation) of the antioxidant molecule as a whole. In general, a molecule with a large conjugative system can be easily oxidized into a stable molecule, and thus would exhibit lower BDE values [26, 41, 196, 216, 217]. Therefore, the above antioxidant qualitative rule is apparently consistent with this quantitative rule.

**SCHEME 9:**
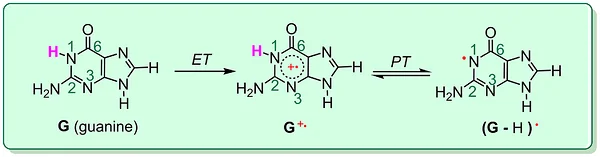
Generation of guanine radical (G‐H•) from guanine via ET *plus* PT pathway.

Under specified conditions, this quantitative rule is undoubtedly reasonable and can successfully predict antioxidant potential of compounds (especially phenolics). However, it depends on HAT antioxidant pathway, relevant BDE values [28, 39], and even special conditions (e.g., gas‐phase [39]). Other antioxidant pathways, unknown BDE values, or physiological conditions, may limit its application. For example, the quantitative rule cannot predict whether γ‐mangostin (**12g**), a xanthone from CHM *Shanzhu* (*Garcinia mangostana* L.), is able to scavenge guanine radical (**G‐H** ^•^), a DNA oxidative lesion [218]. This is because the generation of **G‐H** ^•^ radical is not mediated by HAT in either G‐quadruplex [218] or duplex DNA [219]. It however dependents on an ET *plus* PT pathway (Scheme 9). On the other hand, under physiological conditions, aqueous solution with alkalescence may firstly deprotonateγ‐mangostin into a monoanionic form. For such monoanionic form, ET is reported to be a thermochemically preferential antioxidant pathway [64]. Once monoanionic γ‐mangostin encounters the **G‐**H^•^ radical under physiological conditions, they may interact via ET (rather than HAT) pathway. The quantitative rule based on BDE value is not able to provide an antioxidant prediction.

The quantitative rule however, can easily give a prediction, that is, γ‐mangostin (**12g**) is capable scavenge **G‐H** ^•^ radical. This is because that the catecholic γ‐mangostin can be oxidized into a stable *ortho*‐quinone, regardless the antioxidant pathways (HAT, or ET *plus* PT). By comparison, the **G‐H** ^•^ radical cannot be further abstracted a hydrogen atom, because the whole radical now has only four hydrogen atoms. The two in the left ring can hardly be abstracted to yield a stable conjugation; The two in the right ring has the similar tendency. A possible situation is that γ‐mangostin devotes hydrogen atom through ET *plus* PT pathway to **G‐H** ^•^ radical to restore its stable form (i.e., guanine **G**).

The prediction means that, intake of γ‐mangostin may help to repair DNA oxidative lesions. In fact, γ‐mangostin and other xanthones are abundant in *Shanzhu* and have been known as its main bioactive component. Thus, the antioxidant effects of these xanthones may be partly responsible for the clearing away *heat* efficiency of *Shanzhu*.

**FIGURE 15:**
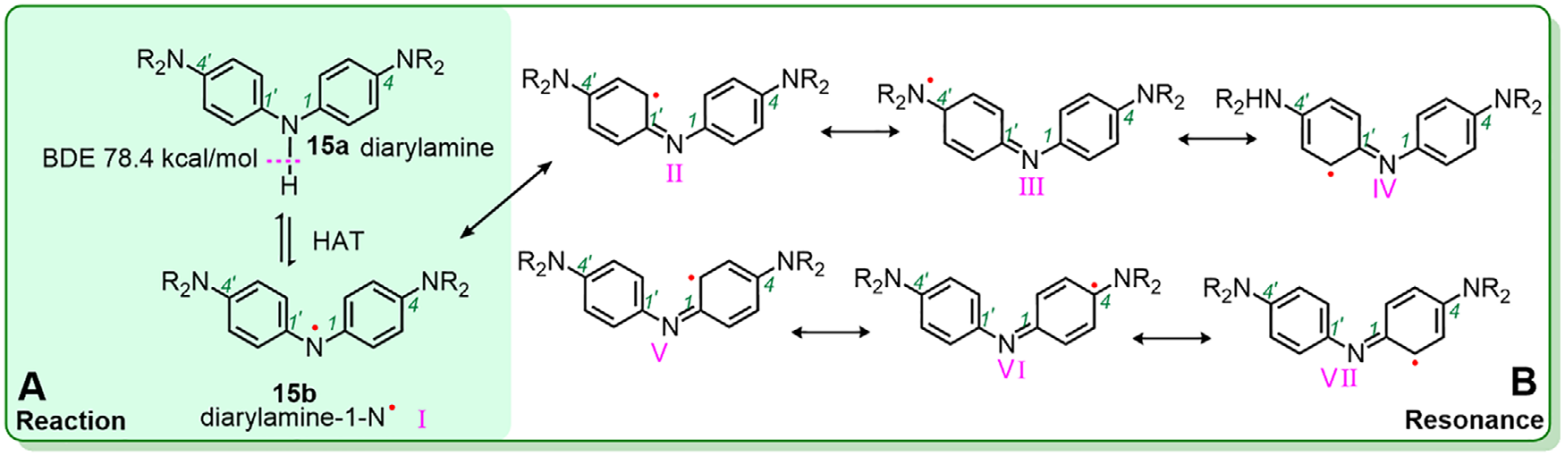
The HAT antioxidant reaction of diarylamine (A) and the resonance formulas of its product (B).

As such, some chemists pointed out that sometimes BDE requirement is not a sufficient factor for predicting the antioxidant potential (e.g., ROO^•^‐scavenging) [26]. A combination of quantitative and qualitative rules may be a good strategy for antioxidant prediction or understanding. Diarylamine (**15a,** Figure 15) is herein taken as an example. Diarylamine (**15a**) has been shown to be a strong antioxidant [26, 28]. Given that **15a** undergoes a HAT reaction, it will be converted into the diarylamine‐1‐N^•^ radical intermediate (**15b**). The presence of two phenyl ring makes the whole radical intermediate (**15b**) possess 7 resonance formulas (I–VII, Figure 15). Therefore, the whole diarylamine‐1‐N^•^ radical is relatively stable. In short, a combination of quantitative and qualitative antioxidant rules may help in precisely and completely understanding or predicting antioxidant capacity.

However, for an experienced free radical chemist, the qualitative antioxidant rule may not be so novel, because it can be deduced using the conjugation effect of orbital hybridization theory and Pauling's resonance theory. Nevertheless, this proposal purposely has four particular scientific significances: (1) it can help in direct prediction of the relative antioxidant levels, according to the drawn structural formula; (2) it reminds to pay close attention to the whole molecule (rather than a part) in understanding an antioxidant, especially, transannular di‐OHs, transannular IHBs, or diarylamine antioxidants; (3) the holism‐based rule can guide medicinal chemists to design more effective and reasonable antioxidant molecules; (4) the qualitative antioxidant rule can overcome the shortcomings of the quantitative rules.

### 4.7 Phenolic Alkaloids and pH Effect

**FIGURE 16:**
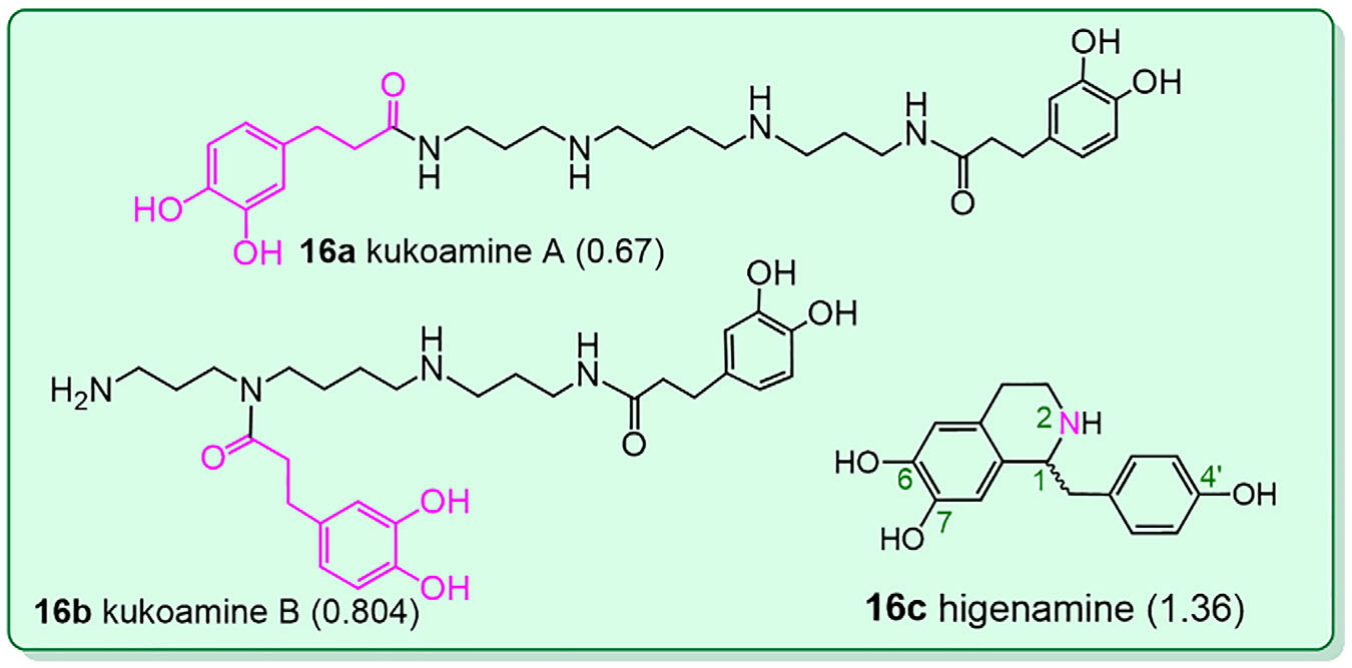
Phenolic alkaloids from CHMs. The values in parentheses are the TEAC obtained from the literature [131, 221].

Structurally, the most important feature of alkaloids is the presence of a nitrogen atom with alkalinity. However, phenolic –OHs, which are weakly acidic, are sometimes attached to the alkaloid skeletons (e.g., the isoquinoline, acridone, and lycopodine skeletons) to construct phenolic antioxidants [220]. Kukoamine A (**16a**), kukoamine B (**16b**), and higenamine (**16c**) are three typical examples (Figure 16).

**FIGURE 17:**
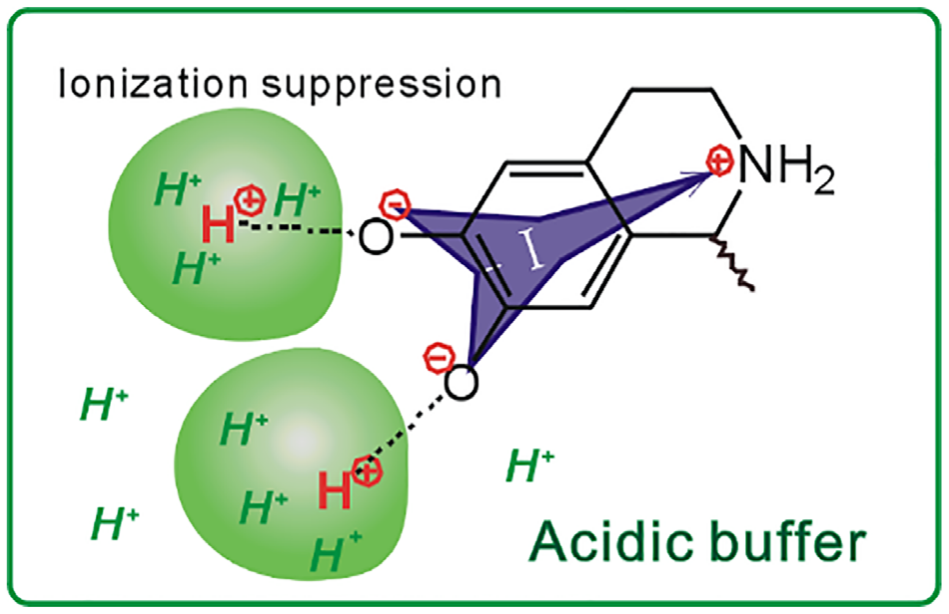
An isoquinoline‐type alkaloid undergoes ionization and the inductive effect (−I) to reduce antioxidant potential in an acidic buffer (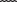, uncertain stereo configuration).

It has been suggested that, at low pH values, the N‐atom of higenamine will be protonated by H^+^ in solution to form a substituted ammonium ion with a net positive charge. The protonated N‐atom can bring about a strong electron‐withdrawing inductive effect (‐I), which can lower the electron density and hinder the ET pathway [131]. Meanwhile, H^+^ ions in an aqueous solution may further suppress the phenolic ionization (i.e., PT). Such synergistic action reduces both the ET and PT potentials (Figure 17); thus, the relative antioxidant ability of higenamine at pH 4.5 is only a third of that at pH 7.4 [221].

On the other hand, our previous study pointed out that the pH value can also affect the ROS generation (e.g., ^•^O_2_^−^ generation) [20]. It means pH value plays a critical role in antioxidant determination. The antioxidant determination (especially that involving biomolecules, e.g., DNA [222]) thus is strongly advised to be conducted in pH 7.4 aqueous buffer by our team [19, 20, 35].

### 4.8 Diels–Alder‐Type Adducts and Steric Effects

**FIGURE 18:**
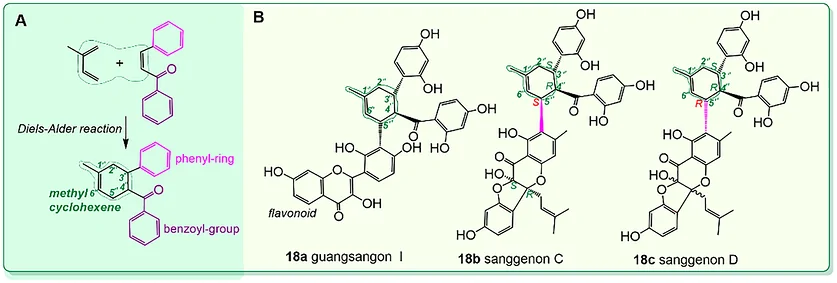
Formation of a Diels–Alder‐type adduct (A), and the reference Diels–Alder‐type adducts isolated from CHMs (B).

The Diels–Alder‐type adduct was first isolated from the CHM *Sangbaipi* (*Mori Cortex*) in 1981 [223]. It is formed through a [4+2] Diels–Alder adduction reaction between chalcone and isoprene during plant metabolism. Through the reaction, a methyl cyclohexene core is formed. Now the core has already connected with a phenyl group and a benzoyl group at 3″ and 4″‐positions, respectively (Figure 18A). Frequently, the 5″‐position can also link other large substituents, such as a flavonoid (**18a**, Figure 18B) [224], and a 2‐phenyl‐benzofuran (guangsangon E [225]). As such, the methyl cyclohexene core usually bears three chiral carbons, i.e., 3″‐carbone, 4″‐carbone, and 5″‐carbone. On the other hand, the above phenyl group, benzoyl group, and large substituent usually introduce phenolic ‐OH to the molecule. These make all Diels–Alder‐type adducts be chiral phenolics.

Of these, sanggenon C (**18b**) and sanggenon D (**18c**) are two typical examples. As shown in Figure 18, sanggenon C and sanggenon D present *(3*″*S,4*″*R,5*″*S)* and *(3*″*S,4*″*R,5*″*R)* absolute configurations, respectively. Hence, they are actually a pair of stereoisomers. The previous observations indicate that the stereoisomers present different antioxidant potentials. For instance, in the Fe^2+^‐chelating reaction, sanggenon C is more active than sanggenon D. The two large adjacent substituents at the 4″ and 5″‐positions in sanggenon C can jointly bind Fe^2+^ to form a big cyclic complex. Sanggenon D, however, has no similar advantage because its 4″,5″‐adjacent‐substituents do not array on the same side. Apparently, these differences can conclusively be attributed to the different steric configurations of the chiral carbons [226]. Nevertheless, the steric effect is much weaker than the aforementioned π–π conjugation effect towards antioxidant phenolics.

### 4.9 Phenolic Hybrids and Special Phenolics

**FIGURE 19:**
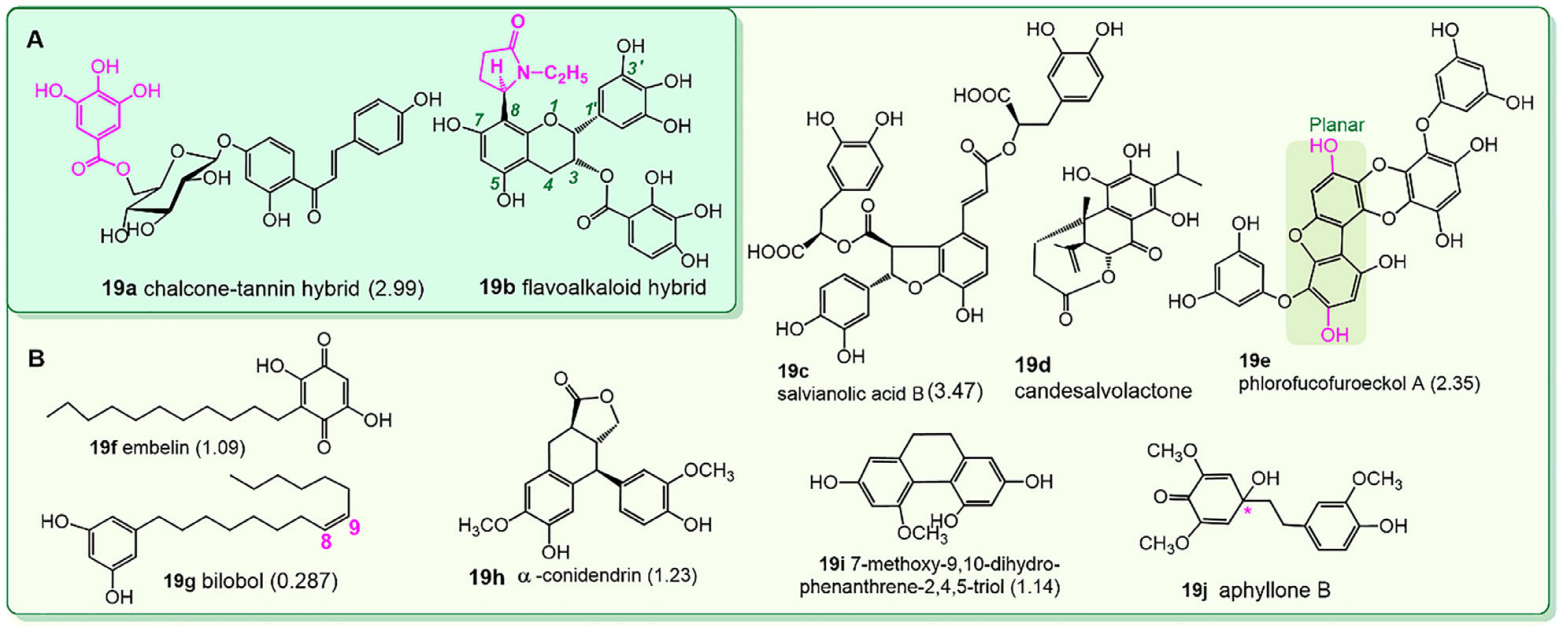
Some phenolic hybrids (A) and special phenolics (B) from CHMs. **19i**: The absolute configuration of the chiral carbon has not been indicated in the original literature [136]. The values in parentheses are the TEAC values obtained from the relevant literature [136, 158, 210, 230, 231, 232].

Besides the above usual skeletons of phenolics from CHMs, there are also some unusual skeletons including phenolic hybrids (**19a**‐**b**) [227] and so‐called special phenolics (**19c–19j**) (Figure 19). Nevertheless, their antioxidant structure–activity relationships comply with the aforementioned rules (Section 4.6). For example, embelin (**19f**) with hydroquinone moiety has a much greater antioxidant capacity than bilobol (**19g**) due to its *meta*‐di‐OH moiety [228, 229]. Phlorofucofuroeckol A (**19e**), with multiple *meta*‐di‐OHs, however, exhibits an even higher antioxidant level (TEAC of 2.35) [210, 230] (Figure 19) due to the transannular di‐OHs moiety in the sample plane (green in Figure 19). The reason its molecular structure looks so special (perhaps even strange) may be that it is derived from algae (*Kunbu* in TCM) and not from common plants. This implies that the aforementioned rules are applicable to all types of phenolic antioxidants, whether plant‐derived or algae‐derived, terricolous or marine.

In addition, chalcone‐tannin hybrid (**19a**) has also been demonstrated to possess high TEAC values and produce cytoprotective effects (Figure 19A). Its bioactivity may be due to the large conjugative system and galloylation, though its large substituents may hinder the molecules from freely crossing cell membranes.

The special phenolics salvianolic acid B (**19c**) and candesalvolactone (**19d**) are derived from *Danshen* (*Salvia* species) [2]. It has been shown that **19c** exhibits an extraordinarily beneficial effect on oxidative stressed mesenchymal stem cells (MSCs). This can be attributed to its strong antioxidant activity (TEAC = 3.47, Figure 19B) [231]. Its extraordinary bioactivity along with its high chemical content makes it the key compound used to characterize the quality of *Danshen* in the Chinese [5].

Finally, α‐conidendrin (**19h**) [210, 233], 7‐methoxy‐9,10‐dihydro‐phenanthrene‐2,4,5‐triol (**19i**) [232], and aphyllone B (**19j**) [136] are also structurally special phenolics. **19i** and **19j**, along with other unusual phenolics (e.g., gigantol and dendrophenol, SI‐2 Table s1) can be found in *Shihu* (*Dendrobium nobile* Lindl) [136, 234, 235]. *Shihu* is regarded as a magic drug in an ancient book of TCM [236]. Even today it is used to treat various diseases. However, it remains unclear whether its versatile effects are associated with these special phenolics. This interesting topic of course needs further work in the future.

## 5 Summary, Conclusion, and Significance

This review paper provides chemical insights into antioxidants from CHMs. It can be summarized that, (1) the antioxidant capacity of CHMs overwhelmingly arises from phenolics (including polyene‐conjugated phenolics) rather than from saponins or polysaccharides. (2) The antioxidant mechanisms of phenolics from CHMs mainly include HAT, ET *plus* PT, RAF, and transition metal chelation. Transition‐metal chelation is an indirect antioxidant mechanism and can occur between adjacent di‐OHs or between adjacent hydroxy‐keto groups. (3) The RAF pathway can lead to a variety of final products, including phenolic‐radical adducts and phenolic‐phenolic dimers. The RAF reaction can occur at different binding sites to form different bonding types and can be affected by structural factors and reaction conditions. The RAF products, however play a critical role in human life and health. Thus, the RAF pathway is not only complicated and variable, but also important and universal. (4) In terms of structure–activity relationships, the antioxidant effect of phenolics is seemingly attributed to the existence of specific antioxidant moieties, such as catechols and hydroxyquinones. However, the high levels of these so‐called “specific antioxidant moieties” can be attributed to the stability of the oxidized products from their reactions with free radicals and, ultimately, to the degree of conjugation of the whole antioxidant molecule. Thus, if a molecule can be oxidized into a larger conjugative system, the entire molecule may possess a strong antioxidant ability. In other words, the antioxidant capacity of one molecule can be judged based on the stability of the whole oxidized product, not just on mere phenolic ‐OH or “specific antioxidant moiety” quantification. (5) Other substituents and structural factors appear to play a minor role, including chiral carbons, (iso)prenylation, glycosidation, and geometrical configurations, unless they disrupt or affect the aforementioned conjugation or “specific antioxidant moieties.” (6) Finally, the pH value can also alter the strength of some antioxidants (especially phenolic alkaloids), which means antioxidant potential can also be affected by reaction conditions.

Of these, conclusion (3) regarding the RAF pathway will likely lead to an entirely new antioxidant field of study and thereby benefit several relevant academic or applicable fields. The cutting‐edge UPLC‐ESI‐Q‐TOF‐MS/MS strategy will also bring about a brilliant future in the field. Conclusion (4) provides a qualitative antioxidant rule which helps us to understand and even directly predict the relative antioxidant levels of molecules according to the drawn structural formula. Thus, it can overcome shortcomings of the quantitative rules. This qualitative rule is, to a great extent, consistent with the holism theory of TCM.

## Author Contributions

**Xican Li**: writing – original draft, conceptualization, formal analysis, writing, supervision, review, and editing .

## Conflicts of Interest

The authors declare no conflicts of interest.

## Supporting information



Supporting Information‐1: Total list of 769 references.
Supporting Information‐2: Table S1 ‐ Structure & cross‐distribution of 477 phenolics in 331 CHMs.
**Supporting File 1**: cbdv71716‐sup‐0001‐SuppMat.pdf.

**Supporting File 2**: cbdv71716‐sup‐0002‐SuppMat.pdf.

## Data Availability

The data that support the findings of this study are available from the corresponding author upon reasonable request.
