# Supplementary material for: Antioxidant Chemistry of Phenolics in Chinese Herbal Medicines: A Holism‐Based Review
Source: Chem Biodivers. 2026 Sep 20;23(9):e71716. doi: 10.1002/cbdv.71716 (PMC13590138; doi:10.1002/cbdv.71716)
Supplement: Supplementary file 1 [file CBDV-23-e71716-s001.pdf]

Total list of 769 references

(1) Li, S. Z. Compendium of Materia Medica (Bencao gangmu); Huaxia Publishing House, 2013.

(2) Wu, Y. B.; Ni, Z. Y.; Shi, Q. W.; Dong, M.; Kiyota, H.; Gu, Y. C.; Cong, B. Constituents from Salvia species and their biological activities. Chem Rev 2012, 112 (11), 5967-6026. DOI: 10.1021/cr200058f.

(3) Tu, Y. Artemisinin-A Gift from Traditional Chinese Medicine to the World (Nobel Lecture). Angew Chem Int Ed Engl 2016, 55 (35), 10210-10226. DOI: 10.1002/anie.201601967.

(4) Ge, H.; Wang, Y. F.; Xu, J.; Gu, Q.; Liu, H. B.; Xiao, P. G.; Zhou, J.; Liu, Y.; Yang, Z.; Su, H. Anti-influenza agents from Traditional Chinese Medicine. Nat Prod Rep 2010, 27 (12), 1758-1780. DOI: 10.1039/c0np00005a.

(5) Chinese-Pharmacopoeia-Commission. Chinese Pharmacopoeia (Part 1). Chinese Medical Science and Technology Press 2025, 1.

(6) Pan, K. Research progress on the antioxidant effect of traditional Chinese medicine for promoting blood circulation and resolving phlegm (Huoxue Huayu efficacies). Zhongguo Zhongyiaoo Zhinan 2013, 11 (30), 328-329.

(7) Niwa, Y.; Miyachi, Y. Antioxidant action of natural health products and Chinese herbs. Inflammation 1986, 10 (1), 79-91.

(8) Poliak, P.; Skorna, P.; Klein, E.; Lukes, V. Thermodynamics of radical scavenging of symmetric carotenoids and their charged species. Food Chem 2018, 268, 542-549. DOI: 10.1016/j.foodchem.2018.06.063.

(9) Milani, A.; Basirnejad, M.; Shahbazi, S.; Bolhassani, A. Carotenoids: biochemistry, pharmacology and treatment. Br J Pharmacol 2017, 174 (11), 1290-1324. DOI: 10.1111/bph.13625.

(10) Ko, K.; Wahyudi, L. D.; Kwon, Y. S.; Kim, J. H.; Yang, H. Nuclear Factor Erythroid 2-Related Factor 2 Activating Triterpenoid Saponins from Camellia japonica Roots. J Nat Prod 2018, 81 (11), 2399-2409. DOI: 10.1021/acs.jnatprod.8b00374.

(11) Matkowski, A.; Jamiokowska, W.; Nawrot, I. Chinese medicinal herbs as source of antioxidant compounds-where tradition meets the future. Curr. Med. Chem. 2013, 20, 984-1004.

(12) Chen, J.; Li, L.; Zhou, X.; Sun, P.; Li, B.; Zhang, X. Preliminary characterization and antioxidant and hypoglycemic activities in vivo of polysaccharides from Huidouba. Food Funct 2018, 9 (12), 6337-6348. DOI: 10.1039/c8fo01117f.

(13) Zhang, S.; Jiang, H.; Jiang, Q.; Zhao, G.; Liang, X.; Zhang, Y.; Liao, Z. A review of natural plant polysaccharides: the relationship between extraction methods, physicochemical properties, structures, and biological activity. J Pharm Pharmacol 2026, 78 (7). DOI: 10.1093/jpp/rgag080.

(14) Li, X. C.; Wu, X. T.; Ling, H. Correlation between antioxidant activities and phenolic contents of Radix Angelicae Sinensis (Danggui). Molecules 2009, 14 (12).

(15) Li, X. C.; Lin, J.; Gao, Y. X.; Han, W. J.; Chen, D. F. Antioxidant activity and mechanism of Rhizoma Cimicifugae. Chem. Cent. J. 2012, 6 (1). DOI: 10.1186/1752-153X-6-140.

(16) Xie, J. T.; Shao, Z. H.; Vanden Hoek, T. L.; Chang, W. T.; Li, J.; Mehendale, S.; Wang, C. Z.; Hsu, C. W.; Becker, L. B.; Yin, J. J.; et al. Antioxidant effects of ginsenoside Re in cardiomyocytes. Eur J Pharmacol 2006, 532 (3), 201-207. DOI: 10.1016/j.ejphar.2006.01.001.

(17) Li, X. C.; Jiang, Q.; Wang, T. T.; Liu, J. J.; Chen, D. F. Comparison of the Antioxidant Effects of Quercitrin and Isoquercitrin: Understanding the Role of the 6''-OH Group. Molecules 2016, 21 (9), 1246. DOI: doi:10.3390/molecules21091246.

(18) Shahidi, F.; Zhong, Y. Lipid oxidation and improving the oxidative stability. Chem Soc Rev 2010, 39 (11), 4067-4079. DOI: 10.1039/b922183m.

(19) Li, X. C. Solvent effects and improvements in the deoxyribose degradation assay for hydroxyl radical-scavenging. Food Chem. 2013, 141 (3), 2083-2088. DOI: 10.1016/j.foodchem.2013.05.084.

(20) Li, X. C. Improved pyrogallol autoxidation method: a reliable and cheap superoxide-scavenging assay suitable for all antioxidants. J. Agric. Food Chem. 2012, 60 (25), 6418-6424. DOI: 10.1021/jf204970r.

(21) Liu, Z. Q. Chemical methods to evaluate antioxidant ability. Chem Rev 2010, 110 (10), 5675-5691. DOI: 10.1021/cr900302x.

(22) Amorati, R.; Valgimigli, L. Advantages and limitations of common testing methods for antioxidants. Free Radic Res 2015, 49 (5), 633-649. DOI: 10.3109/10715762.2014.996146.

(23) Prior, R. L.; Wu, X.; Schaich, K. Standardized methods for the determination of antioxidant capacity and phenolics in foods and dietary supplements. J Agric Food Chem 2005, 53 (10), 4290-4302. DOI: 10.1021/jf0502698.

(24) Kim, D. O.; Lee, C. Y. Comprehensive Study on Vitamin C Equivalent Antioxidant Capacity (VCEAC) of Various Polyphenolics in Scavenging a Free Radical and its Structural Relationship Crit. Rev. Food Sci. 2004, 44.

(25) Birsa, M. L.; Sarbu, L. G. Novel Dithiocarbamic Flavanones with Antioxidant Properties—A Structure–Activity Relationship Study. International Journal of Molecular Sciences 2024, 25 (24). DOI: 10.3390/ijms252413698.

(26) Ingold, K. U.; Pratt, D. A. Advances in radical-trapping antioxidant chemistry in the 21st century: a kinetics and mechanisms perspective. Chem Rev 2014, 114 (18), 9022-9046. DOI: 10.1021/cr500226n.

(27) Perron, N. R.; Brumaghim, J. L. A Review of the Antioxidant Mechanisms of Polyphenol Compounds Related to Iron Binding. Cell Biochem. Biophys. 2009, 53 (2), 75-100. DOI: 10.1007/s12013-009-9043-x.

(28) Lucarini, M.; Pedulli, G. F. Free radical intermediates in the inhibition of the autoxidation reaction. Chem. Soc. Rev. 2010, 39 (6), 2106-2119.

(29) Foti, M. C. Antioxidant properties of phenols. J. Pharm. Pharmacol. 2007, 59 (12), 1673-1685. DOI: 10.1211/jpp.59.12.0010.

(30) Bagryanskaya, E. G.; Marque, S. R. Scavenging of organic C-centered radicals by nitroxides. Chem Rev 2014, 114 (9), 5011-5056. DOI: 10.1021/cr4000946.

(31) Harrison, K. A.; Haidasz, E. A.; Griesser, M.; Pratt, D. A. Inhibition of hydrocarbon autoxidation by nitroxide-catalyzed cross-dismutation of hydroperoxyl and alkylperoxyl radicals. Chem Sci 2018, 9 (28), 6068-6079. DOI: 10.1039/c8sc01575a.

(32) Weinberg, F.; Hamanaka, R.; Wheaton, W. W.; Weinberg, S.; Joseph, J.; Lopez, M.; Kalyanaraman, B.; Mutlu, G. M.; Budinger, G. R.; Chandel, N. S. Mitochondrial metabolism and ROS generation are essential for Kras-mediated tumorigenicity. Proc Natl Acad Sci U S A 2010, 107 (19), 8788-8793. DOI: 10.1073/pnas.1003428107.

(33) Saleem, R.; Robichaud, S. G.; Lamarre, S. G.; Scott, G. R. Mitochondrial physiology in cardiac muscle of deer mice native to high altitude. J Physiol 2025. DOI: 10.1113/jp289893.

(34) Litwinienko, G.; Ingold, K. U. Solvent effects on the rates and mechanisms of reaction of phenols with free radicals. Acc Chem Res 2007, 40 (3), 222-230. DOI: 10.1021/ar0682029.

(35) Li, X. C. 2-Phenyl-4,4,5,5-tetramethylimidazoline-1-oxyl 3-Oxide (PTIO(*)) Radical Scavenging: A New and Simple Antioxidant Assay In Vitro. J. Agric. Food Chem. 2017, 65 (30), 6288-6297. DOI: 10.1021/acs.jafc.7b02247.

(36) Bas, T. G. Dietary Polyphenols (Flavonoids) Derived from Plants for Use in Therapeutic Health: Antioxidant Performance, ROS, Molecular Mechanisms, and Bioavailability Limitations. Inter J Mol Sci 2026, 27 (3). DOI: 10.3390/ijms27031404.

(37) Zheng, R.; Shi, Y.; Jia, Z.; Zhao, C.; Zhang, Q.; Tan, X. Fast repair of DNA radicals. Chem Soc Rev 2010, 39 (8), 2827-2834. DOI: 10.1039/b924875g.

(38) Barisoff, A.; Walton-Raaby, M.; Jofily, P.; Mora-Diez, N. Computational Study of the Peroxyl Radical Scavenging Ability of Phenolic Antioxidants. Antioxidants 2026, 15 (7). DOI: 10.3390/antiox15070868.

(39) Wright, J. S.; Johnson, E. R.; DiLabio, G. A. Predicting the activity of phenolic antioxidants: theoretical method, analysis of substituent effects, and application to major families of antioxidants. J Am Chem Soc 2001, 123 (6), 1173-1183.

(40) Jin, W. B.; Wu, S.; Jian, X. H.; Yuan, H.; Tang, G. L. A radical S-adenosyl-L-methionine enzyme and a methyltransferase catalyze cyclopropane formation in natural product biosynthesis. Nat Commun 2018, 9 (1), 2771. DOI: 10.1038/s41467-018-05217-1.

(41) Zhuravlev, A. V.; Zakharov, G. A.; Shchegolev, B. F.; Savvateeva-Popova, E. V. Antioxidant Properties of Kynurenines: Density Functional Theory Calculations. PLoS Comput Biol 2016, 12 (11), e1005213. DOI: 10.1371/journal.pcbi.1005213.

(42) Li, X.; Jiang, Q.; Chen, B.; Luo, X.; Chen, D. Structure-Activity Relationship and Prediction of the Electron-Transfer Potential of the Xanthones Series. ChemistryOpen 2018, 7, 730 –736. DOI: 10.1002/open.201800108.

(43) Zeppilli, D.; Filippi, M.; Madabeni, A.; Orian, L. A Systematic Topological Picture of ROS Scavenging Activity via Formal Hydrogen Atom Transfer in Phenols and Polyphenols. J Org Chem 2026, 91 (20), 6938-6950. DOI: 10.1021/acs.joc.6c00415.

(44) Zhang, H.; Wu, W.; Mo, Y. Study of proton-coupled electron transfer (PCET) with four explicit diabatic states at the ab initio level. Comput. Theor. Chem. 2017, 1116, 50-58.

(45) Amorati, R.; Valgimigli, L. Methods To Measure the Antioxidant Activity of Phytochemicals and Plant Extracts. J Agric Food Chem 2018, 66 (13), 3324-3329. DOI: 10.1021/acs.jafc.8b01079.

(46) Fourre, I.; Di Meo, F.; Podloucka, P.; Otyepka, M.; Trouillas, P. Dimerization of quercetin, Diels-Alder vs. radical-coupling approach: a joint thermodynamics, kinetics, and topological study. J Mol Model 2016, 22 (8), 190. DOI: 10.1007/s00894-016-3051-8.

(47) Markovic, S.; Tosovic, J. Comparative study of the antioxidative activities of caffeoylquinic and caffeic acids. Food Chem. 2016, 210, 585-592. DOI: 10.1016/j.foodchem.2016.05.019.

(48) Iuga, C.; Alvarez-Idaboy, J. R.; Russo, N. Antioxidant activity of trans-resveratrol toward hydroxyl and hydroperoxyl radicals: a quantum chemical and computational kinetics study. J. Org. Chem. 2012, 77 (8), 3868-3877. DOI: 10.1021/jo3002134.

(49) Foti, M. C.; Daquino, C.; Mackie, I. D.; DiLabio, G. A.; Ingold, K. U. Reaction of phenols with the 2,2-diphenyl-1-picrylhydrazyl radical. Kinetics and DFT calculations applied to determine ArO-H bond dissociation enthalpies and reaction mechanism. J. Org. Chem. 2008, 73 (23), 9270-9282. DOI: 10.1021/jo8016555.

(50) Schrauben, J. N.; Cattaneo, M.; Day, T. C.; Tenderholt, A. L.; Mayer, J. M. Multiple-site concerted proton-electron transfer reactions of hydrogen-bonded phenols are nonadiabatic and well described by semiclassical Marcus theory. J Am Chem Soc 2012, 134 (40), 16635-16645. DOI: 10.1021/ja305668h.

(51) Usharani, D.; Lacy, D. C.; Borovik, A. S.; Shaik, S. Dichotomous hydrogen atom transfer vs proton-coupled electron transfer during activation of X-H bonds (X = C, N, O) by nonheme iron-oxo complexes of variable basicity. J Am Chem Soc 2013, 135 (45), 17090-17104. DOI: 10.1021/ja408073m.

(52) Guitard, R.; Paul, J. F.; Nardello-Rataj, V.; Aubry, J. M. Myricetin, rosmarinic and carnosic acids as superior natural antioxidant alternatives to alpha-tocopherol for the preservation of omega-3 oils. Food Chem 2016, 213, 284-295. DOI: 10.1016/j.foodchem.2016.06.038.

(53) Gutierrez-Oliva, S. Theoretical study of the hydrogen abstraction from vitamin-E analogues. The usefulness of DFT descriptors. J Mol Model 2011, 17 (3), 593-598. DOI: 10.1007/s00894-010-0759-8.

(54) Ohkubo, K.; Moro-oka, Y.; Fukuzumi, S. Hydrogen abstraction from neurotransmitters by active oxygen species facilitated by intramolecular hydrogen bonding in the radical intermediates. Org Biomol Chem 2006, 4 (6), 999-1001. DOI: 10.1039/b600111d.

(55) Medina, M. E.; Galano, A.; Alvarez-Idaboy, J. R. Theoretical study on the peroxyl radicals scavenging activity of esculetin and its regeneration in aqueous solution. Phys Chem Chem Phys 2014, 16 (3), 1197-1207. DOI: 10.1039/c3cp53889c.

(56) Zheng, Y. Z.; Deng, G.; Liang, Q.; Chen, D. F.; Guo, R.; Lai, R. C. Antioxidant Activity of Quercetin and Its Glucosides from Propolis: A Theoretical Study. Sci Rep 2017, 7 (1), 7543. DOI: 10.1038/s41598-017-08024-8.

(57) Shchepin, R.; Moller, M. N.; Kim, H. Y. H.; Hatch, D. M.; Bartesaghi, S.; Kalyanaraman, B.; Radi, R.; Porter, N. A. Tyrosine-Lipid Peroxide Adducts from Radical Termination: Para Coupling and Intramolecular Diels-Alder Cyclization. Journal of the American Chemical Society 2010, 132 (49), 17490-17500, Article. DOI: 10.1021/ja106503a.

(58) Li, D. D.; Han, R. M.; Liang, R.; Chen, C. H.; Lai, W.; Zhang, J. P.; Skibsted, L. H. Hydroxyl radical reaction with trans-resveratrol: initial carbon radical adduct formation followed by rearrangement to phenoxyl radical. J. Phys. Chem. B 2012, 116 (24), 7154-7161. DOI: 10.1021/jp3033337.

(59) Tosovic, J.; Markovic, S. Antioxidative activity of chlorogenic acid relative to trolox in aqueous solution - DFT study. Food Chem 2019, 278, 469-475. DOI: 10.1016/j.foodchem.2018.11.070 From NLM.

(60) Wang, B.; Fita, I.; Rovira, C. Theory Uncovers the Role of the Methionine-Tyrosine-Tryptophan Radical Adduct in the Catalase Reaction of KatGs: O2 Release Mediated by Proton-Coupled Electron Transfer. Chemistry 2018, 24 (20), 5388-5395. DOI: 10.1002/chem.201706076.

(61) Liang, M.; Li, X.; Ouyang, X.; Xie, H.; Chen, D. Antioxidant Mechanisms of Echinatin and Licochalcone A. Molecules 2019, 24 (3). DOI: 10.3390/molecules24010003.

(62) Li, X. C.; Han, L.; Li, Y. R.; Zhang, J.; Chen, J. M.; Lu, W. B.; Zhao, X. J.; Lai, Y. Y.; Chen, D. F.; Wei, G. Protective Effect of Sinapine against Hydroxyl Radical-Induced Damage to Mesenchymal Stem Cells and Possible Mechanisms. Chem. Pharm. Bull. (Tokyo) 2016, 64 (4), 319-325. DOI: 10.1248/cpb.c15-00850.

(63) Keylor, M. H.; Matsuura, B. S.; Griesser, M.; Chauvin, J. R.; Harding, R. A.; Kirillova, M. S.; Zhu, X.; Fischer, O. J.; Pratt, D. A.; Stephenson, C. R. Synthesis of resveratrol tetramers via a stereoconvergent radical equilibrium. Science 2016, 354 (6317), 1260-1265. DOI: 10.1126/science.aaj1597.

(64) Martinez, A.; Hernandez-Marin, E.; Galano, A. Xanthones as antioxidants: a theoretical study on the thermodynamics and kinetics of the single electron transfer mechanism. Food Funct. 2012, 3 (4), 442-450. DOI: 10.1039/c2fo10229c.

(65) Foti, M. C.; Amorati, R.; Pedulli, G. F.; Daquino, C.; Pratt, D. A.; Ingold, K. U. Influence of "remote" intramolecular hydrogen bonds on the stabilities of phenoxyl radicals and benzyl cations. J Org Chem 2010, 75 (13), 4434-4440. DOI: 10.1021/jo100491a.

(66) Martinez, A.; Galano, A.; Vargas, R. Free Radical Scavenger Properties of α-Mangostin: Thermodynamics and Kinetics of HAT and RAF Mechanisms. J. Phys. Chem. B 2011, 115, 12591–12598.

(67) Miguel Reina, M.; Martínez, A. Is Silybin the Best Free Radical Scavenger Compound in Silymarin? J. Phys. Chem. B 2016, 120, 4568-4578.

(68) Li, X.; Chen, B.; Xie, H.; He, Y.; Zhong, D.; Chen, D. Antioxidant Structure(-)Activity Relationship Analysis of Five Dihydrochalcones. Molecules 2018, 23 (5). DOI: 10.3390/molecules23051162.

(69) Jia, C. J.; Sun, X. M.; Zhang, X. Y.; Wei, Z. J.; Jia, B. X. Baeckeins L and M, two novel C-methylated triflavonoids from the roots of Baeckea frutescens L. Nat Prod Res 2018, 1-6. DOI: 10.1080/14786419.2018.1528590.

(70) Wang, L. W.; Su, H. J.; Yang, S. Z.; Won, S. J.; Lin, C. N. New alkaloids and a tetraflavonoid from Cephalotaxus wilsoniana. J Nat Prod 2004, 67 (7), 1182-1185. DOI: 10.1021/np0498657.

(71) Constantin, M. A.; Conrad, J.; Merisor, E.; Koschorreck, K.; Urlacher, V. B.; Beifuss, U. Oxidative dimerization of (E)- and (Z)-2-propenylsesamol with O2 in the presence and absence of laccases and other catalysts: selective

formation of carpanones and benzopyrans under different reaction conditions. J Org Chem 2012, 77 (10), 4528-4543. DOI: 10.1021/jo300263k.

(72) Groehler, A. t.; Kren, S.; Li, Q.; Robledo-Villafane, M.; Schmidt, J.; Garry, M.; Tretyakova, N. Oxidative cross-linking of proteins to DNA following ischemia-reperfusion injury. Free Radic Biol Med 2018, 120, 89-101. DOI: 10.1016/j.freeradbiomed.2018.03.010.

(73) Shen, J.; Deininger, P.; Hunt, J. D.; Zhao, H. 8-Hydroxy-2'-deoxyguanosine (8-OH-dG) as a potential survival biomarker in patients with nonsmall-cell lung cancer. Cancer 2007, 109 (3), 574-580. DOI: 10.1002/cncr.22417.

(74) Varghese, A. J. Sensitization of thymine and uracil to ionizing radiation by p-nitroacetophenone. Int J Radiat Biol Relat Stud Phys Chem Med 1975, 28 (5), 477-484.

(75) Brand-Williams, W.; Cuvelier, M. E.; Berset, C. Use of a Free Radical Method to Evaluate Antioxidant Activity. LWT-Food Sci. Technol. 1995, 28 (1), 25-30.

(76) Leon-Carmona, J. R.; Alvarez-Idaboy, J. R.; Galano, A. On the peroxyl scavenging activity of hydroxycinnamic acid derivatives: mechanisms, kinetics, and importance of the acid-base equilibrium. Phys Chem Chem Phys 2012, 14 (36), 12534-12543. DOI: 10.1039/c2cp40651a.

(77) Mikulski, D.; Gorniak, R.; Molski, M. A theoretical study of the structure-radical scavenging activity of trans-resveratrol analogues and cis-resveratrol in gas phase and water environment. Eur. J. Med. Chem. 2010, 45 (3), 1015-1027. DOI: 10.1016/j.ejmech.2009.11.044.

(78) Moreno-Vasquez, M. J.; Valenzuela-Buitimea, E. L.; Plascencia-Jatomea, M.; Encinas-Encinas, J. C.; Rodriguez-Felix, F.; Sanchez-Valdes, S.; Rosas-Burgos, E. C.; Ocano-Higuera, V. M.; Graciano-Verdugo, A. Z. Functionalization of chitosan by a free radical reaction: Characterization, antioxidant and antibacterial potential. Carbohydr Polym 2017, 155, 117-127. DOI: 10.1016/j.carbpol.2016.08.056.

(79) Koulkes-Pujo, A. M.; Moreau, M.; Sutton, J. Methane formation from the reactions of hydroxyl radicals and hydrogen atoms with dimethyl sulfoxide (DMSO). FEBS Lett 1981, 129 (1), 52-54.

(80) Osman, A. M.; Wong, K. K.; Fernyhough, A. ABTS radical-driven oxidation of polyphenols: isolation and structural elucidation of covalent adducts. Biochem Biophys Res Commun 2006, 346 (1), 321-329. DOI: 10.1016/j.bbrc.2006.05.118.

(81) Osman, A. M. Multiple pathways of the reaction of 2,2-diphenyl-1-picrylhydrazyl radical (DPPH.) with (+)-catechin: evidence for the formation of a covalent adduct between DPPH. and the oxidized form of the polyphenol. Biochem. Biophys. Res. Commun. 2011, 412 (3), 473-478. DOI: 10.1016/j.bbrc.2011.07.123.

(82) Keylor, M. H.; Matsuura, B. S.; Stephenson, C. R. Chemistry and Biology of Resveratrol-Derived Natural Products. Chem Rev 2015, 115 (17), 8976-9027. DOI: 10.1021/cr500689b.

(83) Liu, Q.; Li, X.; Ouyang, X.; Chen, D. Dual Effect of Glucuronidation of a Pyrogallol-Type Phytophenol Antioxidant: A Comparison between Scutellarein and Scutellarin. Molecules 2018, 23 (12). DOI: 10.3390/molecules23123225.

(84) Ouyang, X.; Li, X.; Lu, W.; Zhao, X.; Chen, D. A Null B-Ring Improves the Antioxidant Levels of Flavonol: A Comparative Study between Galangin and 3,5,7-Trihydroxychromone. Molecules 2018, 23 (12), 3083. DOI: 10.3390/molecules23123083.

(85) Li, X. C.; Wang, T. T.; Liu, J. J.; Liu, Y. L.; Zhang, J.; Lin, J.; Zhao, Z. X.; Chen, D. F. Effect and mechanism of wedelolactone as antioxidant-coumestan on •OH-treated mesenchymal stem cells. Arab. J. Chem. 2017. DOI: org/10.1016/j.arabjc.2017.03.008.

(86) Li, X. C.; Gao, Y. X.; Li, F.; Liang, A. F.; Xu, Z. M.; Bai, Y.; Mai, W. Q.; Han, L.; Chen, D. F. Maclurin protects against hydroxyl radical-induced damages to mesenchymal stem cells: antioxidant evaluation and mechanistic insight. Chem. Biol. Interact. 2014, 219, 221-228. DOI: 10.1016/j.cbi.2014.06.014.

(87) Xie, H.; Li, X. C.; Ren, Z. X.; Qiu, W. M.; Chen, J. L.; Jiang, Q.; Chen, B.; Chen, D. F. Antioxidant and Cytoprotective Effects of Tibetan Tea and Its Phenolic Components. Molecules 2018, 23 (2). DOI: 10.3390/molecules23020179.

(88) Li, X.; Xie, H.; Zhan, R.; Chen, D. Effect of Double Bond Position on 2-Phenyl-benzofuran Antioxidants: A Comparative Study of Moracin C and Iso-Moracin C. Molecules 2018, 23 (4), 754. DOI: 10.3390/molecules23040754.

(89) Li, X.; Chen, B.; Zhao, X.; Chen, D. 2-Phenyl-4,4,5,5-tetramethylimidazoline-1-oxyl 3-oxide Radical (PTIO*) Trapping Activity and Mechanisms of 16 Phenolic Xanthones. Molecules 2018, 23 (7). DOI: 10.3390/molecules23071692.

(90) Ingleson, M. J.; Pink, M.; Fan, H.; Caulton, K. G. Redox chemistry of the triplet complex (PNP)Co(I). J Am Chem Soc 2008, 130 (13), 4262-4276. DOI: 10.1021/ja074378+.

(91) Li, X. Comparative Study of 1,1-Diphenyl-2-picryl-hydrazyl Radical (DPPH•) Scavenging Capacity of the Antioxidant Xanthones Family. ChemistrySelect 2018, 3, 13081-13086.

(92) Hassan, I.; Pinto, S.; Weisbecker, C.; Attygalle, A. B. Competitive Deprotonation and Superoxide [O(2)(-)*)] Radical-Anion Adduct Formation Reactions of Carboxamides under Negative-Ion Atmospheric-Pressure Helium-Plasma Ionization (HePI) Conditions. J. Am. Soc. Mass Spectrom. 2016, 27 (3), 394-401. DOI: 10.1007/s13361-015-1296-6.

(93) Dawoud, A. A.; Kawaguchi, T.; Jankowiak, R. Integrated microfluidic device with an electroplated palladium decoupler for more sensitive amperometric detection of the 8-hydroxy-deoxyguanosine (8-OH-dG) DNA adduct. Anal Bioanal Chem 2007, 388 (1), 245-252. DOI: 10.1007/s00216-007-1203-5.

(94) Crean, C.; Uvaydov, Y.; Geacintov, N. E.; Shafirovich, V. Oxidation of single-stranded oligonucleotides by carbonate radical anions: generating intrastrand cross-links between guanine and thymine bases separated by cytosines. Nucleic Acids Res 2008, 36 (3), 742-755. DOI: 10.1093/nar/gkm1092.

(95) Abeyawardhane, D. L.; Fernandez, R. D.; Heitger, D. R.; Crozier, M. K.; Wolver, J. C.; Lucas, H. R. Copper Induced Radical Dimerization of alpha-Synuclein Requires Histidine. J Am Chem Soc 2018. DOI: 10.1021/jacs.8b08947 From NLM.

(96) Mazzaferro, L. S.; Huttel, W.; Fries, A.; Muller, M. Cytochrome P450-catalyzed regio- and stereoselective phenol coupling of fungal natural products. J Am Chem Soc 2015, 137 (38), 12289-12295. DOI: 10.1021/jacs.5b06776.

(97) Gil Girol, C.; Fisch, K. M.; Heinekamp, T.; Gunther, S.; Huttel, W.; Piel, J.; Brakhage, A. A.; Muller, M. Regio- and stereoselective oxidative phenol coupling in Aspergillus niger. Angew Chem Int Ed Engl 2012, 51 (39), 9788-9791. DOI: 10.1002/anie.201203603.

(98) Chen, C. H.; Han, R. M.; Liang, R.; Fu, L. M.; Wang, P.; Ai, X. C.; Zhang, J. P.; Skibsted, L. H. Direct observation of the beta-carotene reaction with hydroxyl radical. J Phys Chem B 2011, 115 (9), 2082-2089. DOI: 10.1021/jp1100889 From NLM.

(99) Ghiladi, R. A.; Medzihradszky, K. F.; Rusnak, F. M.; Ortiz de Montellano, P. R. Correlation between isoniazid resistance and superoxide reactivity in mycobacterium tuberculosis KatG. J Am Chem Soc 2005, 127 (38), 13428-13442. DOI: 10.1021/ja054366t From NLM.

(100) Tai, A.; Iomori, A.; Ito, H. Structural evidence for the DPPH radical-scavenging mechanism of 2-O-alpha-d-glucopyranosyl-l-ascorbic acid. Bioorg Med Chem 2017, 25 (20), 5303-5310. DOI: 10.1016/j.bmc.2017.07.044 From NLM.

(101) Ida, S.; Terashima, T.; Ouchi, M.; Sawamoto, M. Selective radical addition with a designed heterobifunctional halide: a primary study toward sequence-controlled polymerization upon template effect. J Am Chem Soc 2009, 131 (31), 10808-10809. DOI: 10.1021/ja9031314 From NLM.

(102) Spisak, S. N.; Zabula, A. V.; Alkan, M.; Filatov, A. S.; Rogachev, A. Y.; Petrukhina, M. A. Site-Directed Dimerization of Bowl-Shaped Radical Anions to Form a sigma-Bonded Dibenzocorannulene Dimer. Angew Chem Int Ed Engl 2018, 57 (21), 6171-6175. DOI: 10.1002/anie.201801537 From NLM.

(103) Ji, X.; Li, Y.; Xie, L.; Lu, H.; Ding, W.; Zhang, Q. Expanding Radical SAM Chemistry by Using Radical Addition Reactions and SAM Analogues. Angew Chem Int Ed Engl 2016, 55 (39), 11845-11848. DOI: 10.1002/anie.201605917 From NLM.

(104) Rossler, S. L.; Jelier, B. J.; Tripet, P. F.; Shemet, A.; Jeschke, G.; Togni, A.; Carreira, E. M. Pyridyl Radical Cation for C-H Amination of Arenes. Angew Chem Int Ed Engl 2019, 58 (2), 526-531. DOI: 10.1002/anie.201810261 From NLM.

(105) Romero, K. J.; Galliher, M. S.; Raycroft, M. A. R.; Chauvin, J. R.; Bosque, I.; Pratt, D. A.; Stephenson, C. R. J. Electrochemical Dimerization of Phenylpropenoids and the Surprising Antioxidant Activity of the Resultant Quinone Methide Dimers. Angew Chem Int Ed Engl 2018, 57 (52), 17125-17129. DOI: 10.1002/anie.201810870.

(106) Tzirakis, M. D.; Orfanopoulos, M. Radical reactions of fullerenes: from synthetic organic chemistry to materials science and biology. Chem Rev 2013, 113 (7), 5262-5321. DOI: 10.1021/cr300475r.

(107) Karakoti, A.; Singh, S.; Dowding, J. M.; Seal, S.; Self, W. T. Redox-active radical scavenging nanomaterials. Chem Soc Rev 2010, 39 (11), 4422-4432. DOI: 10.1039/b919677n.

(108) Yuan, L.; Han, Y.; Tao, T.; Phan, H.; Chi, C. Formation of a Macrocycles-in-a-Macrocycle Superstructure with All-gauche Conformation by Reversible Radical Association. Angew Chem Int Ed Engl 2018, 57 (29), 9023-9027. DOI: 10.1002/anie.201804720.

(109) Cheng, S.; Jin, L.; Ma, J.; Liu, S.; Zhang, S.; Guan, D.-X.; de Visser, S. P.; Schymanski, E. L.; Ji, L. Discovery of Metabolic Cross-Coupling in Phenol-Arylamine Mixtures by Cytochrome P450 via Combined Computational and Experimental Approaches. Environmental Science & Technology 2025, 59 (42), 22874-22886. DOI: 10.1021/acs.est.5c08819.

(110) Song, M. F.; Li, Y. S.; Ootsuyama, Y.; Kasai, H.; Kawai, K.; Ohta, M.; Eguchi, Y.; Yamato, H.; Matsumoto, Y.; Yoshida, R.; et al. Urea, the most abundant component in urine, cross-reacts with a commercial 8-OH-dG ELISA kit and contributes to overestimation of urinary 8-OH-dG. Free Radic Biol Med 2009, 47 (1), 41-46. DOI: 10.1016/j.freeradbiomed.2009.02.017.

(111) Bartesaghi, S.; Wenzel, J.; Trujillo, M.; Lopez, M.; Joseph, J.; Kalyanaraman, B.; Radi, R. Lipid peroxyl radicals mediate tyrosine dimerization and nitration in membranes. Chem Res Toxicol 2010, 23 (4), 821-835. DOI: 10.1021/tx900446r.

(112) Cadenas, S. ROS and redox signaling in myocardial ischemia-reperfusion injury and cardioprotection. Free Radic Biol Med 2018, 117, 76-89. DOI: 10.1016/j.freeradbiomed.2018.01.024.

(113) Kehrer, J. P. The Haber-Weiss reaction and mechanisms of toxicity. Toxicology 2000, 149 (1), 43-50.

(114) Sun, H.; Zhang, C.; Cao, S.; Sheng, T.; Dong, N.; Xu, Y. Fenton reactions drive nucleotide and ATP syntheses in cancer. J Mol Cell Biol 2018, 10 (5), 448-459. DOI: 10.1093/jmcb/mjy039.

(115) Barnham, K. J.; Bush, A. I. Biological metals and metal-targeting compounds in major neurodegenerative diseases. Chem Soc Rev 2014, 43 (19), 6727-6749. DOI: 10.1039/c4cs00138a.

(116) Ren, J.; Meng, S.; Lekka Ch, E.; Kaxiras, E. Complexation of flavonoids with iron: structure and optical signatures. J. Phys. Chem. B 2008, 112 (6), 1845-1850. DOI: 10.1021/jp076881e.

(117) Mladenka, P.; Macakova, K.; Filipsky, T.; Zatloukalova, L.; Jahodar, L.; Bovicelli, P.; Silvestri, I. P.; Hrdina, R.; Saso, L. In vitro analysis of iron chelating activity of flavonoids. J Inorg Biochem 2011, 105 (5), 693-701. DOI: 10.1016/j.jinorgbio.2011.02.003.

(118) Tian, Y.; Li, X.; Xie, H.; Wang, X.; Xie, Y.; Chen, C.; Chen, D. Protective Mechanism of the Antioxidant Baicalein toward Hydroxyl Radical-Treated Bone Marrow-Derived Mesenchymal Stem Cells. Molecules 2018, 23 (1), 223. DOI: 10.3390/molecules23010223.

(119) Jiang, Q.; Li, X. C.; Tian, Y. G.; Lin, Q. Q.; Xie, H.; Lu, W. B.; Chi, Y. G.; Chen, D. F. Lyophilized aqueous extracts of Mori Fructus and Mori Ramulus protect Mesenchymal stem cells from *OH-treated damage: bioassay and antioxidant mechanism. BMC Complement. Altern. Med. 2017, 16 (1), 423. DOI: 10.1186/s12906-017-1730-3.

(120) Liu, J. J.; Li, X. C.; Lin, J.; Li, Y. R.; Wang, T. T.; Jiang, Q.; Chen, D. F. Sarcandra glabra (Caoshanhu) protects mesenchymal stem cells from oxidative stress: a bioevaluation and mechanistic chemistry. BMC Complement. Altern. Med. 2016, 16 (1), 423. DOI: 10.1186/s12906-016-1383-7.

(121) Ferrari, E. B., R.; Sacchi, S.; Pignedoli, F.; Asti, M.; Saladini, M. Curcumin derivatives as metal-chelating agents with potential multifunctional activity for pharmaceutical applications. J. Inorg. Biochem. 2014, 139, 38-48.

(122) Maria M. Kasprzak, M.; Erxleben, A.; Ochocki, J. Properties and applications of flavonoid metal complexes. RSC Adv. 2015, 5, 45853-45877

(123) Li, X.; Xie, Y.; Li, K.; Wu, A.; Xie, H.; Guo, Q.; Xue, P.; Maleshibek, Y.; Zhao, W.; Guo, J.; et al. Antioxidation and Cytoprotection of Acteoside and Its Derivatives: Comparison and Mechanistic Chemistry. Molecules 2018, 23 (2). DOI: 10.3390/molecules23020498.

(124) Li, X.; Xie, Y.; Xie, H.; Yang, J.; Chen, D. π -π Conjugation Enhances Oligostilbene's Antioxidant Capacity: Evidence from α-Viniferin and Caraphenol A. Molecules 2018, 23 (3), 694. DOI: 10.3390/molecules23030694.

(125) Li, X. C.; Li, K.; Xie, H.; Xie, Y. L.; Li, Y. Y.; Zhao, X. J.; Jiang, X. H.; Chen, D. F. Antioxidant and Cytoprotective Effects of the Di-O-Caffeoylquinic Acid Family: The Mechanism, Structure-Activity Relationship, and Conformational Effect. Molecules 2018, 23 (1), 222. DOI: 10.3390/molecules23010222.

(126) Li, X.; Liu, J.; Lin, J.; Wang, T.; Huang, J.; Lin, Y.; Chen, D. Protective Effects of Dihydromyricetin against *OH-Induced Mesenchymal Stem Cells Damage and Mechanistic Chemistry. Molecules 2016, 21 (5), 604. DOI: 10.3390/molecules21050604.

(127) Li, X.; Xie, H.; Jiang, Q.; Wei, G.; Lin, L.; Li, C.; Ou, X.; Yang, L.; Xie, Y.; Fu, Z.; et al. The mechanism of (+) taxifolin's protective antioxidant effect for •OH-treated bone marrow-derived mesenchymal stem cells. Cell Mol Biol Lett. 2017, 22, 231.

(128) Song, J. Y.; Liu, Y.; Zhao, H. Y.; Han, H. T.; Li, Z. F.; Guo, W. H.; Chu, W. Y.; Sun, Z. Z. Efficient nickel(II) naringenin-oxime complex catalyzed Mizoroki-Heck cross-coupling reaction in the presence of hydrazine hydrate. New J Chem 2017, 41 (20), 12288-12292. DOI: 10.1039/c7nj03148c.

(129) Lugovik, K. I.; Eltyshev, A. K.; Suntsova, P. O.; Smoluk, L., T,; Belousova, A. V.; Ulitko, M. V.; Minin, A. S.; Slepukhin, P. A.; Benassi, E.; Belskaya, N. P. Fluorescent boron complexes based on new N,O-chelates as promising candidates for flow cytometry. Org Biomol Chem. 2018, 16 (28), 5150-5162.

(130) Niskanen, M.; Hirva, P.; Haukka, M. The effect of N-ligands on the geometry, bonding, and electronic absorption properties of ruthenium carbonyl chains. Phys Chem Chem Phys 2010, 12 (33), 9777-9782. DOI: 10.1039/c0cp00189a.

(131) Li, X.; Lin, J.; Chen, B.; Xie, H.; Chen, D. Antioxidant and Cytoprotective Effects of Kukoamines A and B: Comparison and Positional Isomeric Effect. Molecules 2018, 23 (4). DOI: 10.3390/molecules23040973.

(132) Grayer, R. J.; Veitch, N. C.; Kite, G. C.; Price, A. M.; Kokubun, T. Distribution of 8-oxygenated leaf-surface flavones in the genus Ocimum. Phytochemistry 2001, 56 (6), 559-567.

(133) Yang, J. S. 13C-NMR Spectroscopic Databook of Natural Products; Chemical Industry Press., 2011.

(134) Qin, H. L.; Yu, D. Q. 1H-NMR Spectroscopic Databook of Natural Products; Chemical Industry Press., 2011.

(135) Li, Y. R.; Li, S.; Lin, C. C. Effect of resveratrol and pterostilbene on aging and longevity. Biofactors 2018, 44 (1), 69-82. DOI: 10.1002/biof.1400.

(136) Yang, D.; Liu, L. Y.; Cheng, Z. Q.; Xu, F. Q.; Fan, W. W.; Zi, C. T.; Dong, F. W.; Zhou, J.; Ding, Z. T.; Hu, J. M. Five new phenolic compounds from Dendrobium aphyllum. Fitoterapia 2015, 100, 11-18. DOI: 10.1016/j.fitote.2014.11.004.

(137) Hu, Y.; Wang, S.; Wu, X.; Zhang, J.; Chen, R.; Chen, M.; Wang, Y. Chinese herbal medicine-derived compounds for cancer therapy: a focus on hepatocellular carcinoma. J. Ethnopharmacol. 2013, 149 (3), 601-612. DOI: 10.1016/j.jep.2013.07.030.

(138) Yang, H.; Xue, X.; Li, H.; Apandi, S. N.; Tay-Chan, S. C.; Ong, S. P.; Tian, E. F. The relative antioxidant activity and steric structure of green tea catechins - A kinetic approach. Food Chem 2018, 257, 399-405. DOI: 10.1016/j.foodchem.2018.03.043.

(139) Zhang, S.; Cui, Y.; Li, L.; Li, Y.; Zhou, P.; Luo, L.; Sun, B. Preparative HSCCC isolation of phloroglucinolysis products from grape seed polymeric proanthocyanidins as new powerful antioxidants. Food Chem 2015, 188, 422-429. DOI: 10.1016/j.foodchem.2015.05.030.

(140) Wang, S.; Wu, S.; Liu, S. Integration of (+)-catechin and β-sitosterol to achieve excellent radical-scavenging activity in emulsions. Food Chem 2019, 272, 596-603. DOI: 10.1016/j.foodchem.2018.08.098.

(141) Plumb, G. W.; de Pascual-Teresa, S.; Santos-Buelga, C.; Rivas-Gonzalo, J. C.; Williamson, G. Antioxidant properties of gallocatechin and prodelphinidins from pomegranate peel. Redox Rep 2002, 7 (1), 41-46. DOI: 10.1179/135100002125000172.

(142) Gao, X.; Lin, X.; Li, X.; Zhang, Y.; Chen, Z.; Li, B. Cellular antioxidant, methylglyoxal trapping, and anti-inflammatory activities of cocoa tea (Camellia ptilophylla Chang). Food Funct 2017, 8 (8), 2836-2846. DOI: 10.1039/c7fo00368d.

(143) Qin, H., L.; Yu, D. Q. 1H-NMR Spectroscopic Databook of Natural Products; Chemical Industry Press, 2011.

(144) Le, H. T. T.; Nguyen, L. H.; Nguyen, T. H.; Nguyen, V.-K.; Danova, A.; Truong, T. N.; Chavasiri, W. Gagones A–F: Six prenylated chalcones from the heartwood of Mansonia gagei. Phytochemistry 2023, 206. DOI: 10.1016/j.phytochem.2022.113516.

(145) Zhang, X.; Huang, S.; Li, Z.; Gao, Y.; Li, Z.; Wang, Q. Study on chemical constituents and their biological activities from the stems of Syringaoblata Lindl. Fitoterapia 2026, 188. DOI: 10.1016/j.fitote.2025.107017.

(146) Lu, Y.; Xue, Y.; Chen, S.; Zhu, H.; Zhang, J.; Li, X. N.; Wang, J.; Liu, J.; Qi, C.; Du, G.; et al. Antioxidant Lignans and Neolignans from Acorus tatarinowii. Sci Rep 2016, 6, 22909. DOI: 10.1038/srep22909.

(147) Tanjung, M.; Rachmadiarti, F.; Prameswari, A.; Ultha Wustha Agyani, V.; Dewi Saputri, R.; Srie Tjahjandarie, T.; Maolana Syah, Y. Airlanggins A-B, two new isoprenylated benzofuran-3-ones from the stem bark of Calophyllum soulattri. Nat Prod Res 2018, 32 (13), 1493-1498. DOI: 10.1080/14786419.2017.1380027.

(148) Kapche, G. D.; Amadou, D.; Waffo-Teguo, P.; Donfack, J. H.; Fozing, C. D.; Harakat, D.; Tchana, A. N.; Merillon, J. M.; Moundipa, P. F.; Ngadjui, B. T.; et al. Hepatoprotective and antioxidant arylbenzofurans and flavonoids from the twigs of Morus mesozygia. Planta Med 2011, 77 (10), 1044-1047. DOI: 10.1055/s-0030-1270745.

(149) Du, L.; Mahdi, F.; Jekabsons, M. B.; Nagle, D. G.; Zhou, Y. D. Natural and semisynthetic mammea-type isoprenylated dihydroxycoumarins uncouple cellular respiration. J Nat Prod 2011, 74 (2), 240-248. DOI: 10.1021/np100762s.

(150) Peng, X.; Liu, J.; Wang, C.; Han, Z.; Shu, Y.; Li, X.; Zhou, L.; Qiu, M. Unusual prenylated phenols with antioxidant activities from Ganoderma cochlear. Food Chem 2015, 171, 251-257. DOI: 10.1016/j.foodchem.2014.08.127.

(151) Jia, Y.; He, Y.; Lu, F. The structure-antioxidant activity relationship of dehydrodiferulates. Food Chem 2018, 269, 480-485. DOI: 10.1016/j.foodchem.2018.07.038.

(152) Baschieri, A.; Pulvirenti, L.; Muccilli, V.; Amorati, R.; Tringali, C. Chain-breaking antioxidant activity of hydroxylated and methoxylated magnolol derivatives: the role of H-bonds. Org Biomol Chem 2017, 15 (29), 6177-6184. DOI: 10.1039/c7ob01195d.

(153) Rice-Evans, C. A.; Miller, N. J.; Paganga, G. Structure-antioxidant activity relationships of flavonoids and phenolic acids. Free Radic Biol Med 1996, 20 (7), 933-956.

(154) Fujisawa, S.; Atsumi, T.; Kadoma, Y.; Sakagami, H. Antioxidant and prooxidant action of eugenol-related compounds and their cytotoxicity. Toxicology 2002, 177 (1), 39-54.

(155) Karas, D. U., J.; Valentová, K. Galloylation of polyphenols alters their biological activity. Food Chem. Toxi. 2017, 105, 223-240.

(156) Chyba, A.; Mastihuba, V.; Mastihubova, M. Effective enzymatic caffeoylation of natural glucopyranosides. Bioorg Med Chem Lett 2016, 26 (6), 1567-1570. DOI: 10.1016/j.bmcl.2016.02.010.

(157) Tang, Y.; Lou, F.; Wang, J.; Li, Y.; Zhuang, S. Coumaroyl flavonol glycosides from the leaves of Ginkgo biloba. Phytochemistry 2001, 58 (8), 1251-1256.

(158) Li, X.; Tian, Y.; Wang, T.; Lin, Q.; Feng, X.; Jiang, Q.; Liu, Y.; Chen, D. Role of the p-Coumaroyl Moiety in the Antioxidant and Cytoprotective Effects of Flavonoid Glycosides: Comparison of Astragalin and Tiliroside. Molecules 2017, 22 (7). DOI: 10.3390/molecules22071165.

(159) Li, X. C.; Liu , J. J.; Zhao, Z. X.; Wang, T. T.; Lin, J.; Chen, D. F. Effects of Natural ChalconeTannin Hybrids Protecting Mesenchymal Stem Cells against ROS-mediated Oxidative Damage and Indexes for Antioxidant Mechanisms. Chem. Lett. 2016, 45 (7), 743-745.

(160) Mitjans, M.; Ugartondo, V.; Martinez, V.; Tourino, S.; Torres, J. L.; Vinardell, M. P. Role of galloylation and polymerization in cytoprotective effects of polyphenolic fractions against hydrogen peroxide insult. J Agric Food Chem 2011, 59 (5), 2113-2119. DOI: 10.1021/jf1025532.

(161) Lin, J.; Li, X. C.; Chen, L.; Lu, W. Z.; Chen, X. W.; Han, L.; Chen, D. F. Protective effect against hydroxyl radical-induced DNA damage and antioxidant mechanism of [6]-gingerol: A Chemical Study. B Korean Chem Soc 2014, 35, 1633-1638. DOI: 10.5681/apb.2013.028.

(162) Lin, J.; Li, X.; Chen, B.; Wei, G.; Chen, D. E-Configuration Improves Antioxidant and Cytoprotective Capacities of Resveratrols. Molecules 2018, 23 (7). DOI: 10.3390/molecules23071790.

(163) Esatbeyoglu, T.; Ewald, P.; Yasui, Y.; Yokokawa, H.; Wagner, A. E.; Matsugo, S.; Winterhalter, P.; Rimbach, G. Chemical Characterization, Free Radical Scavenging, and Cellular Antioxidant and Anti-Inflammatory Properties of a Stilbenoid-Rich Root Extract of Vitis vinifera. Oxid Med Cell Longev 2016, 2016, 8591286. DOI: 10.1155/2016/8591286.

(164) Holl, J.; Vannozzi, A.; Czemmel, S.; D'Onofrio, C.; Walker, A. R.; Rausch, T.; Lucchin, M.; Boss, P. K.; Dry, I. B.; Bogs, J. The R2R3-MYB transcription factors MYB14 and MYB15 regulate stilbene biosynthesis in Vitis vinifera. Plant Cell 2013, 25 (10), 4135-4149. DOI: 10.1105/tpc.113.117127.

(165) Watts, K. T.; Lee, P. C.; Schmidt-Dannert, C. Biosynthesis of plant-specific stilbene polyketides in metabolically engineered Escherichia coli. BMC Biotechnol 2006, 6, 22. DOI: 10.1186/1472-6750-6-22.

(166) Shi, S. P.; Wanibuchi, K.; Morita, H.; Endo, K.; Noguchi, H.; Abe, I. Enzymatic formation of unnatural novel chalcone, stilbene, and benzophenone scaffolds by plant type III polyketide synthase. Org Lett 2009, 11 (3), 551-554. DOI: 10.1021/ol802606w.

(167) Ruhmann, S.; Treutter, D.; Fritsche, S.; Briviba, K.; Szankowski, I. Piceid (resveratrol glucoside) synthesis in stilbene synthase transgenic apple fruit. J Agric Food Chem 2006, 54 (13), 4633-4640. DOI: 10.1021/jf060249l.

(168) Cuendet, M.; Potterat, O.; Salvi, A.; Testa, B.; Hostettmann, K. A stilbene and dihydrochalcones with radical scavenging activities from Loiseleuria procumbens. Phytochemistry 2000, 54 (8), 871-874.

(169) Sahu, N. K.; Balbhadra, S. S.; Choudhary, J.; Kohli, D. V. Exploring pharmacological significance of chalcone scaffold: a review. Curr Med Chem 2012, 19 (2), 209-225.

(170) Wang, G.; Xue, Y.; An, L.; Zheng, Y.; Dou, Y.; Zhang, L.; Liu, Y. Theoretical study on the structural and antioxidant properties of some recently synthesised 2,4,5-trimethoxy chalcones. Food Chem 2015, 171, 89-97. DOI: 10.1016/j.foodchem.2014.08.106.

(171) Zhao, Q.; Zhang, Y.; Wang, G.; Hill, L.; Weng, J.; Chen, X.; Xue, H.; Martin, C. A specialized flavone biosynthetic pathway has evolved in the medicinal plant, Scutellaria baicalensis. Sci. Adv. 2016, 2, e1501780. DOI: 10.1126/sciadv.1501780.

(172) Li, X.; Han, W.; Mai, W.; Wang, L. Antioxidant activity and mechanism of Tetrahydroamentoflavone in vitro. Nat Prod Commun 2013, 8, 787-789.

(173) Wang, K.; Chen, X. Protective effect of flavonoids on oxidative stress injury in Alzheimer’s disease. Nat Prod Res 2024, 39 (5), 1272-1299. DOI: 10.1080/14786419.2024.2345760.

(174) Zainuddin, M.-S.; Pamidi, N.; Azman, A. S.; Bai Magalingam, K.; Bhuvanendran, S. Oxidative stress biomarkers and flavonoids in Alzheimer's disease: current clinical evidence and therapeutic perspectives. Redox Rep 2026, 31 (1). DOI: 10.1080/13510002.2026.2677396.

(175) Nguyen, N. H.; Pham, T. H. T.; Nguyen, N. T. T.; Bui, V. K. H.; Van Vo, G. Herbal Medicine in Diabetes Treatment: An Updated Strategy With Flavonoid Compounds in Preclinical and Clinical Studies. Chem Biodivers 2025. DOI: 10.1002/cbdv.202402806.

(176) Li, S.; Huang, Z.; Lv, L.; Wang, S.; Yang, G.; Zhang, L.; Du, M.; Zhang, Y.; Ma, J.; Hong, M.; et al. Bioactive polyphenols from Isodon serra with antioxidant and hepatoprotective activities in vitro and in vivo. RSC Advances 2026, 16 (10), 8487-8498. DOI: 10.1039/d5ra08382f.

(177) Stepanic, V.; Gall Troselj, K.; Lucic, B.; Markovic, Z.; Amic, D. Bond dissociation free energy as a general parameter for flavonoid radical scavenging activity. Food Chem 2013, 141 (2), 1562-1570. DOI: 10.1016/j.foodchem.2013.03.072.

(178) Musialik, M.; Kuzmicz, R.; Pawlowski, T. S.; Litwinienko, G. Acidity of hydroxyl groups: an overlooked influence on antiradical properties of flavonoids. J. Org. Chem. 2009, 74 (7), 2699-2709. DOI: 10.1021/jo802716v.

(179) Seyoum, A.; Asres, K.; El-Fiky, F. K. Structure-radical scavenging activity relationships of flavonoids. Phytochemistry 2006, 67 (18), 2058-2070. DOI: 10.1016/j.phytochem.2006.07.002.

(180) Woodman, O.; Meeker, W.; Boujaoude, M. Vasorelaxant and antioxidant activity of flavonols and flavones: Structure-activity relationships. J. Cardiovasc. Pharm. 2005, 46 (3), 302-309.

(181) Rice-Evans, C. A.; Miller, N. J.; Paganga, G. Structure-antioxidant activity relationships of flavonoids and phenolic acids. Free Radic. Biol. Med. 1996, 20 (7), 933-956.

(182) Zuvela, P.; David, J.; Wong, M. W. Interpretation of ANN-based QSAR models for prediction of antioxidant activity of flavonoids. J. Comput. Chem. 2018. DOI: 10.1002/jcc.25168.

(183) Cherrak, S. A.; Mokhtari-Soulimane, N.; Berroukeche, F.; Bensenane, B.; Cherbonnel, A.; Merzouk, H.; Elhabiri, M. In Vitro Antioxidant versus Metal Ion Chelating Properties of Flavonoids: A Structure-Activity Investigation. PLoS One 2016, 11 (10), e0165575. DOI: 10.1371/journal.pone.0165575.

(184) Choi, J. S.; Chung, H. Y.; Kang, S. S.; Jung, M. J.; Kim, J. W.; No, J. K.; Jung, H. A. The Structure–Activity Relationship of Flavonoids as Scavengers of Peroxynitrite. Phytother. Res. 2002, 16, 232-235.

(185) Rahman, M. M.; Ichiyanagi, T.; Komiyama, T.; Hatano, Y.; Konishi, T. Superoxide radical- and peroxynitrite-scavenging activity of anthocyanins; structure-activity relationship and their synergism. Free Radic. Res. 2006, 40 (9), 993-1002. DOI: 10.1080/10715760600815322.

(186) Ali, H. M.; Almagribi, W.; Al-Rashidi, M. N. Antiradical and reductant activities of anthocyanidins and anthocyanins, structure-activity relationship and synthesis. Food Chem. 2016, 194, 1275-1282. DOI: 10.1016/j.foodchem.2015.09.003.

(187) Singh, M.; Kaur, M.; Silakari, O. Flavones: an important scaffold for medicinal chemistry. Eur. J. Med. Chem. 2014, 84, 206-239. DOI: 10.1016/j.ejmech.2014.07.013.

(188) Mendes, A. P.; Borges, R. S.; Neto, A. M.; de Macedo, L. G.; da Silva, A. B. The basic antioxidant structure for flavonoid derivatives. J. Mol. Model. 2012, 18 (9), 4073-4080. DOI: 10.1007/s00894-012-1397-0.

(189) Hu, J. P.; Calomme, M.; Lasure, A.; De Bruyne, T.; Pieters, L.; Vlietinck, A.; Vanden Berghe, D. A. Structure-activity relationship of flavonoids with superoxide scavenging activity. Biol. Trace. Elem. Res. 1995, 47 (1-3), 327-331. DOI: 10.1007/BF02790134.

(190) Cao, G.; Sofic, E.; Prior, R. L. Antioxidant and prooxidant behavior of flavonoids: structure-activity relationships. Free Radic. Biol. Med. 1997, 22 (5), 749-760.

(191) Omidreza Firuzia, A. L., Rita Petruccib, Giancarlo Marrosub, Luciano Saso. Evaluation of the antioxidant activity of flavonoids by bferric reducing antioxidant powerQ assay and cyclic voltammetry. Biochimica et Biophysica Acta 2005, 1721, 174-184.

(192) Tournaire, C.; Croux, S.; Maurette, M. T.; Beck, I.; Hocquaux, M.; Braun, A. M.; Oliveros, E. Antioxidant activity of flavonoids: efficiency of singlet oxygen (1 delta g) quenching. J. Photochem. Photobiol. B 1993, 19 (3), 205-215.

(193) Cos, P.; Ying, L.; Calomme, M.; Hu, J. P.; Cimanga, K.; Poel, B. V.; Pieters, L.; Vlietinck, A. J.; Berghe, D. V. Structure-Activity Relationship and Classification of Flavonoids as Inhibitors of Xanthine Oxidase and Superoxide Scavengers. J. Nat. Prod. 1998, 61, 71-76.

(194) Krenn, L.; Presser, A.; Pradhan, R.; Bahr, B.; Paper, D. H.; Mayer, K. K.; Kopp, B. Sulfemodin 8-O-β-D-glucoside, a new sulfated anthraquinone glycoside, and antioxidant phenolic compounds from Rheum emodi. J Nat Prod 2003, 66 (8), 1107-1109. DOI: 10.1021/np0301442.

(195) Boucherle, B.; Peuchmaur, M.; Boumendjel, A.; Haudecoeur, R. Occurrences, biosynthesis and properties of aurones as high-end evolutionary products. Phytochemistry 2017, 142, 92-111. DOI: 10.1016/j.phytochem.2017.06.017.

(196) Bairwa, K.; Singh, I. N.; Roy, S. K.; Grover, J.; Srivastava, A.; Jachak, S. M. Rotenoids from Boerhaavia diffusa as potential anti-inflammatory agents. J Nat Prod 2013, 76 (8), 1393-1398. DOI: 10.1021/np300899w.

(197) Nenadis, N.; Sigalas, M. P. A DFT study on the radical scavenging activity of maritimetin and related aurones. J Phys Chem A 2008, 112 (47), 12196-12202. DOI: 10.1021/jp8058905.

(198) Yang, X.; Ying, Z.; Liu, H.; Ying, X.; Yang, G. A new homoisoflavone from Portulaca oleracea L. and its antioxidant activity. Nat Prod Res 2018, 1-7. DOI: 10.1080/14786419.2018.1484465.

(199) Su, B. N.; Cuendet, M.; Hawthorne, M. E.; Kardono, L. B.; Riswan, S.; Fong, H. H.; Mehta, R. G.; Pezzuto, J. M.; Kinghorn, A. D. Constituents of the bark and twigs of Artocarpus dadah with cyclooxygenase inhibitory activity. J Nat Prod 2002, 65 (2), 163-169.

(200) Lee, D.; Bhat, K. P.; Fong, H. H.; Farnsworth, N. R.; Pezzuto, J. M.; Kinghorn, A. D. Aromatase inhibitors from Broussonetia papyrifera. J Nat Prod 2001, 64 (10), 1286-1293.

(201) Kapche, G. D.; Fozing, C. D.; Donfack, J. H.; Fotso, G. W.; Amadou, D.; Tchana, A. N.; Bezabih, M.; Moundipa, P. F.; Ngadjui, B. T.; Abegaz, B. M. Prenylated arylbenzofuran derivatives from Morus mesozygia with antioxidant activity. Phytochemistry 2009, 70 (2), 216-221. DOI: 10.1016/j.phytochem.2008.12.014.

(202) Yao, C. S.; Lin, M.; Liu, X.; Wang, Y. H. Stilbene derivatives from Gnetum cleistostachyum. J Asian Nat Prod Res 2005, 7 (2), 131-137. DOI: 10.1080/10286020310001625102.

(203) Tan, W. N.; Khairuddean, M.; Wong, K. C.; Tong, W. Y.; Ibrahim, D. Antioxidant compounds from the stem bark of Garcinia atroviridis. J Asian Nat Prod Res 2016, 18 (8), 804-811. DOI: 10.1080/10286020.2016.1160071.

(204) Tantapakul, C.; Maneerat, W.; Sripisut, T.; Ritthiwigrom, T.; Andersen, R. J.; Cheng, P.; Cheenpracha, S.; Raksat, A.; Laphookhieo, S. New Benzophenones and Xanthones from Cratoxylum sumatranum ssp. neriifolium and Their Antibacterial and Antioxidant Activities. J Agric Food Chem 2016, 64 (46), 8755-8762. DOI: 10.1021/acs.jafc.6b03643.

(205) Foti, M. C.; Johnson, E. R.; Vinqvist, M. R.; Wright, J. S.; Barclay, L. R.; Ingold, K. U. Naphthalene diols: a new class of antioxidants intramolecular hydrogen bonding in catechols, naphthalene diols, and their aryloxyl radicals. J Org Chem 2002, 67 (15), 5190-5196.

(206) Fan, G. J.; Liu, X. D.; Qian, Y. P.; Shang, Y. J.; Li, X. Z.; Dai, F.; Fang, J. G.; Jin, X. L.; Zhou, B. 4,4'-Dihydroxy-trans-stilbene, a resveratrol analogue, exhibited enhanced antioxidant activity and cytotoxicity. Bioorg Med Chem 2009, 17 (6), 2360-2365. DOI: 10.1016/j.bmc.2009.02.014.

(207) Nenadis, N.; Lazaridou, O.; Tsimidou, M. Z. Use of reference compounds in antioxidant activity assessment. J Agric Food Chem 2007, 55 (14), 5452-5460. DOI: 10.1021/jf070473q.

(208) Assimopoulou, A. N.; Sinakos, Z.; Papageorgiou, V. P. Radical scavenging activity of Crocus sativus L. extract and its bioactive constituents. Phytother Res 2005, 19 (11), 997-1000. DOI: 10.1002/ptr.1749.

(209) Somparn, P.; Phisalaphong, C.; Nakornchai, S.; Unchern, S.; Morales, N. P. Comparative antioxidant activities of curcumin and its demethoxy and hydrogenated derivatives. Biol Pharm Bull 2007, 30 (1), 74-78.

(210) Foti, M. C.; Slavova-Kazakova, A.; Rocco, C.; Kancheva, V. D. Kinetics of curcumin oxidation by 2,2-diphenyl-1-picrylhydrazyl (DPPH): an interesting case of separated coupled proton-electron transfer. Org Biomol Chem 2016, 14 (35), 8331-8337. DOI: 10.1039/c6ob01439a.

(211) Tesoriere, L.; Ciaccio, M.; Bongiorno, A.; Riccio, A.; Pintaudi, A. M.; Livrea, M. A. Antioxidant activity of all-trans-retinol in homogeneous solution and in phosphatidylcholine liposomes. Arch Biochem Biophys 1993, 307 (1), 217-223. DOI: 10.1006/abbi.1993.1581.

(212) Gajowik, A.; Dobrzynska, M. M. Lycopene - antioxidant with radioprotective and anticancer properties. A review. Rocz Panstw Zakl Hig 2014, 65 (4), 263-271.

(213) Esatbeyoglu, T.; Rimbach, G. Canthaxanthin: From molecule to function. Mol Nutr Food Res 2017, 61 (6). DOI: 10.1002/mnfr.201600469.

(214) Najafi, H.; Yarijani, Z. M.; Najafi, M. Theoretical and Experimental in vivo Study of Antioxidant Activity of Crocin in Order to Propose Novel Derivatives with Higher Antioxidant Activity and Their Delivery via Nanotubes and Nanocones. Inflammation 2017, 40 (5), 1794-1802. DOI: 10.1007/s10753-017-0623-4.

(215) Deng, M.; Li, D.; Zhang, Y.; Zhou, G.; Liu, W.; Cao, Y.; Zhang, W. Protective effect of crocin on ultraviolet B induced dermal fibroblast photoaging. Mol Med Rep 2018, 18 (2), 1439-1446. DOI: 10.3892/mmr.2018.9150.

(216) Focsan, A. L.; Bowman, M. K.; Molnar, P.; Deli, J.; Kispert, L. D. Carotenoid radical formation: dependence on conjugation length. J Phys Chem B 2011, 115 (30), 9495-9506. DOI: 10.1021/jp204787b.

(217) Mazzone, G.; Malaj, N.; Russo, N.; Toscano, M. Density functional study of the antioxidant activity of some recently synthesized resveratrol analogues. Food Chem. 2013, 141, 2017–2024.

(218) Zhang, H. Y.; Sun, Y. M.; Wang, X. L. Substituent effects on O--H bond dissociation enthalpies and ionization potentials of catechols: a DFT study and its implications in the rational design of phenolic antioxidants and elucidation of structure-activity relationships for flavonoid antioxidants. Chemistry 2003, 9 (2), 502-508. DOI: 10.1002/chem.200390052.

(219) Wu, L.; Liu, K.; Jie, J.; Song, D.; Su, H. Direct observation of guanine radical cation deprotonation in G-quadruplex DNA. J Am Chem Soc 2015, 137 (1), 259-266. DOI: 10.1021/ja510285t.

(220) Kobayashi, K.; Tagawa, S. Direct observation of guanine radical cation deprotonation in duplex DNA using pulse radiolysis. J Am Chem Soc 2003, 125 (34), 10213-10218. DOI: 10.1021/ja036211w.

(221) Xie, Y.; Li, X.; Chen, J.; Deng, Y.; Lu, W.; Chen, D. pH Effect and Chemical Mechanisms of Antioxidant Higenamine. Molecules 2018, 23, 2176. DOI: 10.3390/molecules23092176

(222) Qin, H., L.; Yu, D. Q. 1H-NMR Spectroscopic Databook of Natural Products-alkaloid; Chemical Industry Press, 2011.

(223) Li, X. C.; Mai, W. Q.; Wang, L.; Han, W. J. A hydroxyl-scavenging assay based on DNA damage in vitro. Anal. Biochem. 2013, 438 (1), 29-31. DOI: 10.1016/j.ab.2013.03.014.

(224) Nomura, T.; Fukai, T.; Hano, Y.; Uzawa, J. Structure of sanggenon C, a natural hypotensive Diels-Alder adduct from Chinese crude drug Sang-bai-pi. Heterocycles 1981, 16, 2141-2148.

(225) Dai, S. J.; Ma, Z. B.; Wu, Y.; Chen, R. Y.; Yu, D. Q. Guangsangons F-J, anti-oxidant and anti-inflammatory Diels-Alder type adducts, from Morus macroura Miq. Phytochemistry 2004, 65 (23), 3135-3141. DOI: 10.1016/j.phytochem.2004.07.010.

(226) Dai, S. J.; Mi, Z. M.; Ma, Z. B.; Li, S.; Chen, R. Y.; Yu, D. Q. Bioactive diels-alder type adducts from the stem bark of Morus macroura. Planta Med 2004, 70 (8), 758-763. DOI: 10.1055/s-2004-827208.

(227) Li, X.; Ren, Z.; Wu , Z.; Fu, Z.; Xie, H.; Deng, L.; Jiang, X.; Chen, D. Steric Effect of Antioxidant Diels-Alder-Type Adducts: A Comparison of Sanggenon C with Sanggenon D. Molecules 2018, 23 (10), 2610. DOI: 10.3390/molecules23102610

(228) Kang, H. S.; Chung, H. Y.; Jung, J. H.; Son, B. W.; Choi, J. S. A new phlorotannin from the brown alga Ecklonia stolonifera. Chem Pharm Bull (Tokyo) 2003, 51 (8), 1012-1014.

(229) Wang, T.; Li, L.; Li, Y.; Wu, J.; Huang, Y.; Wei, G.; Chen, D. Mechanistic Chemistry of Extraordinary Capacity of Salvianolic Acid B on Oxidativelydamaged Mesenchymal Stem Cells. J. Chin. Chem. Soc. 2016, 63, 924–929. DOI: 10.1002/jccs.201600112

(230) Sritularak, B.; Anuwat, M.; Likhitwitayawuid, K. A new phenanthrenequinone from Dendrobium draconis. J Asian Nat Prod Res 2011, 13 (3), 251-255. DOI: 10.1080/10286020.2010.546354.

(231) Cheng, J.; Wu, F. H.; Wang, P.; Ke, J. P.; Wan, X. C.; Qiu, M. H.; Bao, G. H. Flavoalkaloids with a Pyrrolidinone Ring from Chinese Ancient Cultivated Tea Xi-Gui. J Agric Food Chem 2018, 66 (30), 7948-7957. DOI: 10.1021/acs.jafc.8b02266.

(232) Sumino, M.; Sekine, T.; Ruangrungsi, N.; Igarashi, K.; Ikegami, F. Ardisiphenols and other antioxidant principles from the fruits of Ardisia colorata. Chem Pharm Bull (Tokyo) 2002, 50 (11), 1484-1487.

(233) Tanaka, A.; Arai, Y.; Kim, S. N.; Ham, J.; Usuki, T. Synthesis and biological evaluation of bilobol and adipostatin A. J Asian Nat Prod Res 2011, 13 (4), 290-296. DOI: 10.1080/10286020.2011.554828.

(234) Banskota, A. H.; Tezuka, Y.; Nguyen, N. T.; Awale, S.; Nobukawa, T.; Kadota, S. DPPH radical scavenging and nitric oxide inhibitory activities of the constituents from the wood of Taxus yunnanensis. Planta Med 2003, 69 (6), 500-505. DOI: 10.1055/s-2003-40641.

(235) Fernandes, D. C.; Regasini, L. O.; Vellosa, J. C.; Pauletti, P. M.; Castro-Gamboa, I.; Bolzani, V. S.; Oliveira, O. M.; Silva, D. H. Myeloperoxidase inhibitory and radical scavenging activities of flavones from Pterogyne nitens. Chem Pharm Bull (Tokyo) 2008, 56 (5), 723-726.

(236) Klongkumnuankarn, P.; Busaranon, K.; Chanvorachote, P.; Sritularak, B.; Jongbunprasert, V.; Likhitwitayawuid, K. Cytotoxic and Antimigratory Activities of Phenolic Compounds from Dendrobium brymerianum. Evid Based Complement Alternat Med 2015, 2015, 350410. DOI: 10.1155/2015/350410.

(237) Tao, H. J. Variorum of Shennong's Classic of Materia Medica; People's Medical Publishing House; , 1995.

(238) Essa, H. A.; El-Metwally, A. E. 6-Gingerol ameliorates high-fat, high-sucrose diet-induced metabolic dysfunction and depressive-like behaviors by attenuating neuroinflammation and oxidative stress. Metabolic Brain Disease 2026, 41 (1). DOI: 10.1007/s11011-025-01782-9.

(239) Zhang, F.; Zhang, J. G.; Yang, W.; Xu, P.; Xiao, Y. L.; Zhang, H. T. 6-Gingerol attenuates LPS-induced neuroinflammation and cognitive impairment partially via suppressing astrocyte overactivation. Biomed. Pharmacother. 2018, 107, 1523-1529, Article. DOI: 10.1016/j.biopha.2018.08.136.

(240) Zuo, A. R.; Dong, H. H.; Yu, Y. Y.; Shu, Q. L.; Zheng, L. X.; Yu, X. Y.; Cao, S. W. The antityrosinase and antioxidant activities of flavonoids dominated by the number and location of phenolic hydroxyl groups. Chin. Med. 2018, 13, 12, Article. DOI: 10.1186/s13020-018-0206-9.

(241) Marteau, C.; Guitard, R.; Penverne, C.; Favier, D.; Nardello-Rataj, V.; Aubry, J. M. Boosting effect of ortho-propenyl substituent on the antioxidant activity of natural phenols. Food Chem. 2016, 196, 418-427, Article. DOI: 10.1016/j.foodchem.2015.09.007.

(242) Mateen, S.; Rehman, M. T.; Shahzad, S.; Naeem, S. S.; Faizy, A. F.; Khan, A. Q.; Khan, M. S.; Husain, F. M.; Moin, S. Anti-oxidant and anti-inflammatory effects of cinnamaldehyde and eugenol on mononuclear cells of rheumatoid arthritis patients. European journal of pharmacology 2019, 852, 14-24. DOI: 10.1016/j.ejphar.2019.02.031.

(243) Sharma, U. K.; Kumar, R.; Gupta, A.; Ganguly, R.; Singh, A. K.; Ojha, A. K.; Pandey, A. K. Ameliorating efficacy of eugenol against metanil yellow induced toxicity in albino Wistar rats. Food and chemical toxicology : an international journal published for the British Industrial Biological Research Association 2019, 126, 34-40. DOI: 10.1016/j.fct.2019.01.032.

(244) Cheng, J.; Dong, S. Q.; Yi, L. T.; Geng, D.; Liu, Q. Magnolol abrogates chronic mild stress-induced depressive-like behaviors by inhibiting neuroinflammation and oxidative stress in the prefrontal cortex of mice. International Immunopharmacology 2018, 59, 61-67, Article. DOI: 10.1016/j.intimp.2018.03.031.

(245) Gursul, S.; Karabulut, I.; Durmaz, G. Antioxidant efficacy of thymol and carvacrol in microencapsulated walnut oil triacylglycerols. Food Chem. 2019, 278, 805-810, Article. DOI: 10.1016/j.foodchem.2018.11.134.

(246) Dairi, N.; Ferfera-Harrar, H.; Ramos, M.; Garrigos, M. C. Cellulose acetate/AgNPs-organoclay and/or thymol nano-biocomposite films with combined antimicrobial/antioxidant properties for active food packaging use. International journal of biological macromolecules 2018, 121, 508-523. DOI: 10.1016/j.ijbiomac.2018.10.042.

(247) Hernandez, D. A.; Tenorio, F. J. Reactivity indexes of antioxidant molecules from Rosmarinus officinalis. Structural Chemistry 2018, 29 (3), 741-751, Article. DOI: 10.1007/s11224-017-1066-0.

(248) Loussouarn, M.; Krieger-Liszkay, A.; Svilar, L.; Bily, A.; Birtic, S.; Havaux, M. Carnosic Acid and Carnosol, Two Major Antioxidants of Rosemary, Act through Different Mechanisms. Plant Physiology 2017, 175 (3), 1381-1394, Article. DOI: 10.1104/pp.17.01183.

(249) Ghasemi, S.; Koohi, D. E.; Emmamzadehhashemi, M. S. B.; Khamas, S. S.; Moazen, M.; Hashemi, A. K.; Amin, G.; Golfakhrabadi, F.; Yousefi, Z.; Yousefbeyk, F. Investigation of phenolic compounds and antioxidant activity of leaves extracts from seventeen cultivars of Iranian olive (Olea europaea L.). Journal of Food Science and Technology-Mysore 2018, 55 (11), 4600-4607, Article. DOI: 10.1007/s13197-018-3398-1.

(250) Yildiz, Z. I.; Celebioglu, A.; Kilic, M. E.; Durgun, E.; Uyar, T. Fast-dissolving carvacrol/cyclodextrin inclusion complex electrospun fibers with enhanced thermal stability, water solubility, and antioxidant activity. Journal of Materials Science 2018, 53 (23), 15837-15849, Article. DOI: 10.1007/s10853-018-2750-1.

(251) Adeyemi, O. S.; Atolani, O.; Banerjee, P.; Arolasafe, G.; Preissner, R.; Etukudoh, P.; Ibraheem, O. Computational and experimental validation of antioxidant properties of synthesized bioactive ferulic acid derivatives. International Journal of Food Properties 2018, 21 (1), 101-113, Article. DOI: 10.1080/10942912.2018.1439958.
(252) Shirazi, M. T.; Gholami, H.; Kavoosi, G.; Rowshan, V.; Tafsiry, A. Chemical composition, antioxidant, antimicrobial and cytotoxic activities of Tagetes minuta and Ocimum basilicum essential oils. Food science & nutrition 2014, 2 (2), 146-155. DOI: 10.1002/fsn3.85.
(253) Teissedre, P. L.; Waterhouse, A. L. Inhibition of oxidation of human low-density lipoproteins by phenolic substances in different essential oils varieties. Journal of Agricultural and Food Chemistry 2000, 48 (9), 3801-3805, Article. DOI: 10.1021/jf990921x.
(254) Zhang, R. F.; Khan, S. A.; Lin, Y. S.; Guo, D. L.; Pan, X. W.; Liu, L.; Wei, Z. C.; Zhang, Y.; Deng, Y. Y.; Zhang, M. W. Phenolic profiles and cellular antioxidant activity of longan pulp of 24 representative Chinese cultivars. International Journal of Food Properties 2018, 21 (1), 746-759, Article. DOI: 10.1080/10942912.2018.1425705.
(255) Jung, M. J.; Kang, S. S.; Jung, Y. J.; Choi, J. S. Phenolic glycosides from the stem bark of Albizzia julibrissin. Chemical & pharmaceutical bulletin 2004, 52 (12), 1501-1503. DOI: 10.1248/cpb.52.1501.
(256) Wu, Q.; Fu, D. X.; Hou, A. J.; Lei, G. Q.; Llu, Z. J.; Chen, J. K.; Zhou, T. S. Antioxidative phenols and phenolic glycosides from Curculigo orchioides. Chemical & pharmaceutical bulletin 2005, 53 (8), 1065-1067, Article. DOI: 10.1248/cpb.53.1065.
(257) Chen, L. H.; Chen, C.; Zheng, Z. Z.; Liu, S. S.; Tong, Q. X.; Xiao, J. B.; Lin, H. T.; Ming, Y. L. Cytotoxic and antioxidant activities of Macfadyena unguis-cati L. aerial parts and bioguided isolation of the antitumor active components. Industrial Crops and Products 2017, 107, 531-538, Article; Proceedings Paper. DOI: 10.1016/j.indcrop.2017.05.018.
(258) Aminah, N. S.; Yulvia, A.; Tanjung, M. Methyl-3,4-Dihydroxybenzoate and 9-10-Dihydrophenanthrene-2,4,7-Triol Two Phenolic Compounds from Dioscorea alata L. and Their Antioxidant Activity. In 6th International Conference and Workshops on Basic and Applies Sciences, Abdullah, H. Y. Ed.; AIP Conference Proceedings, Vol. 1888; Amer Inst Physics, 2017.
(259) Demirci, M. A.; Ipek, Y.; Gul, F.; Ozen, T.; Demirtas, I. Extraction, isolation of heat-resistance phenolic compounds, antioxidant properties, characterization and purification of 5-hydroxymaltol from Turkish apple pulps. Food Chemistry 2018, 269, 111-117, Article. DOI: 10.1016/j.foodchem.2018.06.147.
(260) He, Z. D.; Lau, K. M.; But, P. P. H.; Jiang, R. W.; Dong, H.; Ma, S. C.; Fung, K. P.; Ye, W. C.; Sun, H. D. Antioxidative glycosides from the leaves of Ligustrum robustum. Journal of Natural Products 2003, 66 (6), 851-854, Article. DOI: 10.1021/np020568g.
(261) Qiong, W., et al. Antioxidative Phenols and Phenolic Glycosides from Curculigo orchioides. Chem. Pharm. Bull 2005.
(262) Liu, C.-M.; Yang, H.-X.; Ma, J.-Q.; Yang, W.; Feng, Z.-J.; Sun, J.-M.; Cheng, C.; Li, J.; Jiang, H. Role of AMPK pathway in lead-induced endoplasmic reticulum stress in kidney and in paeonol-induced protection in mice. Food and chemical toxicology : an international journal published for the British Industrial Biological Research Association 2018, 122, 87-94. DOI: 10.1016/j.fct.2018.10.024.
(263) Wang, H.; Sun, Y.; Ye, W. C.; Xiong, F.; Wu, H. J.; Yang, C. H.; Zhao, S. X. Antioxidative phenylethanoid and phenolic glycosides from Picrorhiza scrophulariiflora. Chem. Pharm. Bull. 2004, 52 (5), 615-617, Article. DOI: 10.1248/cpb.52.615.
(264) Zhang, H. Y.; Ge, N.; Zhang, Z. Y. Theoretical elucidation of activity differences of five phenolic antioxidants. Acta Pharmacologica Sinica 1999, 20 (4), 363-366, Article.

(265) Schwartner, C.; Bors, W.; Michel, C.; Franck, U.; MullerJakic, B.; Nenninger, A.; Asakawa, Y.; Wagner, H. Effect of Marchantins and related compounds on 5-lipoxygenase and cyclooxygenase and their antioxidant properties: A structure activity relationship study. Phytomedicine 1995, 2 (2), 113-117, Article. DOI: 10.1016/s0944-7113(11)80055-3.

(266) Xie, S. Y.; Yang, X. Y.; Ding, Z. G.; Li, M. G.; Zhao, J. Y. Chemical Constituents and Pharmacological Effects of Taraxacum mongolicum Hand.-Mazz. Natural Product Research and Development 2012, 24 (S1), 141-151.

(267) Zhou, J.; Ma, Y.; Jia, Y.; Pang, M.; Cheng, G.; Cai, S. Phenolic profiles, antioxidant activities and cytoprotective effects of different phenolic fractions from oil palm (Elaeis guineensis Jacq.) fruits treated by ultra-high pressure. Food Chem. 2019, 288, 68-77. DOI: 10.1016/j.foodchem.2019.03.002.

(268) Wang, X. Z.; Li, X. C.; Chen, D. F. Evaluation of antioxidant activity of isoferulic acid in vitro. Nat. Prod. Commun. 2011, 6 (9), 1285-1288.

(269) Alsanie, W. F.; El-Hallous, E. I.; Dessoky, E. S.; Ismail, I. A. Viper's Bugloss (Echium vulgare L) Extract as A Natural Antioxidant and Its Effect on Hyperlipidemia. Int. J. Pharm. Phytopharm. Res. 2018, 8 (1), 81-89, Article.

(270) Li, X. C.; Hu, Q. P.; Jiang, S. X.; Li, F.; Lin, J.; Han, L.; Hong, Y. L.; Lu, W. B.; Gao, Y. X.; Chen, D. F. Flos Chrysanthemi Indici protects against hydroxyl-induced damages to DNA and MSCs via antioxidant mechanism. J. Saudi Chem. Soc. 2015, 19 (4), 454-460. DOI: 10.1016/j.jscs.2014.06.004.

(271) Wang, Z. Y.; Xia, Q.; Liu, X.; Liu, W. X.; Huang, W. Z.; Mei, X.; Luo, J.; Shon, M. X.; Lin, R. C.; Zou, D. X.; et al. Phytochemistry, pharmacology, quality control and future research of Forsythia suspensa (Thunb.) Vahl: A review. Journal of Ethnopharmacology 2018, 210, 318-339. DOI: 10.1016/j.jep.2017.08.040.

(272) Jiang, Q.; Li, X. C.; Tian, Y. G.; Lin, Q. Q.; Xie, H.; Lu, W. B. A.; Chi, Y. G.; Chen, D. F. Lyophilized aqueous extracts of Mori Fructus and Mori Ramulus protect Mesenchymal stem cells from center dot OH-treated damage: bioassay and antioxidant mechanism. Bmc Complementary and Alternative Medicine 2017, 17. DOI: ARTN 242

10.1186/s12906-017-1730-3.

(273) Shin, H. Y.; Kim, S. M.; Lee, J. H.; Lim, S. T. Solid-state fermentation of black rice bran with Aspergillus awamori and Aspergillus oryzae: Effects on phenolic acid composition and antioxidant activity of bran extracts. Food Chem. 2019, 272, 235-241, Article. DOI: 10.1016/j.foodchem.2018.07.174.

(274) Li, X. C.; Wang, X. Z.; Chen, D. F.; Chen, S. Z. Antioxidant of Protocatechuic Acid. Functional Foods in Health and Disease 2011, 7, 232-244.

(275) Kawabata, J.; Okamoto, Y.; Kodama, A.; Makimoto, T.; Kasai, T. Oxidative dimers produced from protocatechuic and gallic esters in the DPPH radical scavenging reaction. J Agric Food Chem 2002, 50 (19), 5468-5471.

(276) Samsonowicz, M.; Regulska, E.; Karpowicz, D.; Lesniewska, B. Antioxidant properties of coffee substitutes rich in polyphenols and minerals. Food Chem. 2019, 278, 101-109, Article. DOI: 10.1016/j.foodchem.2018.11.057.

(277) Xie, Y.; Li, X.; Xu, J.; Jiang, Q.; Xie, H.; He, J.; Chen, D. Two phenolic antioxidants in Suoyang enhance viability of *OH-damaged mesenchymal stem cells: comparison and mechanistic chemistry. Chem. Cent. J. 2017, 11 (1). DOI: 10.1186/s13065-017-0313-1.

(278) Zhang, J. H.; Tian, H. L.; Zhan, P.; Du, F. L.; Zong, A. Z.; Xu, T. C. Isolation and identification of phenolic compounds in Chinese purple yam and evaluation of antioxidant activity. Lwt-Food Science and Technology 2018, 96, 161-165, Article. DOI: 10.1016/j.lwt.2018.05.019.

(279) Nakilcioglu-Tas, E. The Effects of Sugar Addition and Degree of Roast on the Bioactive Compounds and Antioxidant Activity of Turkish-Style Coffee Brews. Indian Journal of Pharmaceutical Education and Research 2018, 52 (3), 456-466, Article. DOI: 10.5530/ijper.52.3.53.

(280) Cheng, F. F.; Chen, W. J.; Huang, Y. L.; Zhang, C. M.; Shen, Y.; Hai, H.; Zheng, Y. J.; Tang, M. M.; Zhao, S. L.; Liu, J. K. Protective effect of areca inflorescence extract on hydrogen peroxide-induced oxidative damage to human serum albumin. Food Res. Int. 2011, 44 (1), 98-102, Article. DOI: 10.1016/j.foodres.2010.11.005.
(281) Rao, Y. Y.; Zhao, X. X.; Li, Z. L.; Huang, J. H. Phenolic acids induced growth of 3D ordered gold nanoshell composite array as sensitive SERS nanosensor for antioxidant capacity assay. Talanta 2018, 190, 174-181, Article. DOI: 10.1016/j.talanta.2018.07.069.
(282) Irakli, M.; Chatzopoulou, P.; Ekateriniadou, L. Optimization of ultrasound-assisted extraction of phenolic compounds: Oleuropein, phenolic acids, phenolic alcohols and flavonoids from olive leaves and evaluation of its antioxidant activities. Industrial Crops and Products 2018, 124, 382-388, Article. DOI: 10.1016/j.indcrop.2018.07.070.
(283) Denk, B.; Kırıkkulak, M.; Güngör, Ş.; Gülhan, M. F.; İnanç, M. E.; Avdatek, F.; Yeni, D.; Taşdemir, U. Can Vanillic Acid, a Phenolic Substance, Be a Useful Tool to Prevent Oxidative Stress Induced by Freezing Ram Sperm? Reproduction in Domestic Animals 2025, 60 (12). DOI: 10.1111/rda.70152.
(284) Al Omairi, N. E.; Radwan, O. K.; Alzahrani, Y. A.; Kassab, R. B. Neuroprotective efficiency of Mangifera indica leaves extract on cadmium-induced cortical damage in rats. Metabolic Brain Disease 2018, 33 (4), 1121-1130, Article. DOI: 10.1007/s11011-018-0222-6.
(285) Rouphael, Y.; Colla, G.; Graziani, G.; Ritieni, A.; Cardarelli, M.; De Pascale, S. Phenolic composition, antioxidant activity and mineral profile in two seed-propagated artichoke cultivars as affected by microbial inoculants and planting time. Food Chemistry 2017, 234, 10-19, Article. DOI: 10.1016/j.foodchem.2017.04.175.
(286) Anupama, N.; Rani, M. R. P.; Shyni, G. L.; Raghu, K. G. Glucotoxicity results in apoptosis in H9c2 cells via alteration in redox homeostasis linked mitochondrial dynamics and polyol pathway and possible reversal with cinnamic acid. Toxicology in Vitro 2018, 53, 178-192, Article. DOI: 10.1016/j.tiv.2018.08.010.
(287) Suriano, S.; Iannucci, A.; Codianni, P.; Fares, C.; Russo, M.; Pecchioni, N.; Marciello, U.; Savino, M. Phenolic acids profile, nutritional and phytochemical compounds, antioxidant properties in colored barley grown in southern Italy. Food Research International 2018, 113, 221-233, Article. DOI: 10.1016/j.foodres.2018.06.072.
(288) Marquez, K.; Contreras, D.; Salgado, P.; Mardones, C. Production of hydroxyl radicals and their relationship with phenolic compounds in white wines. Food Chemistry 2019, 271, 80-86, Article. DOI: 10.1016/j.foodchem.2018.07.165.
(289) Landete, J. M. Ellagitannins, ellagic acid and their derived metabolites: A review about source, metabolism, functions and health. Food Research International 2011, 44 (5), 1150-1160, Review. DOI: 10.1016/j.foodres.2011.04.027.
(290) Yang, T.; Wang, L.; Zhu, M.; Zhang, L.; Yan, L. Properties and molecular mechanisms of resveratrol: a review. Pharmazie 2015, 70 (8), 501-506.
(291) Li, X. K.; LI, H. Y.; Li, S.; Song, N. N.; Hou, X. D.; Zhou, W. H.; Cui, Y. H.; Ma, L. Advances in study on resveratrol. Chinese Traditional and Herbal Drugs 2016, 47 (14), 2568-2577.
(292) Jaa, A.; de Moura, P. H. B.; Ruiz-Larrea, M. B.; Ruiz Sanz, J. I.; Richard, T. Potential Transformation of Food Resveratrol: Mechanisms and Biological Impact. Molecules 2025, 30 (3). DOI: 10.3390/molecules30030536.
(293) Tang, C. C.; Zhang, W. W.; Cai, H. B.; Ye, Z. Y.; Zhang, X.; Tan, W. S. Resveratrol improves ex vivo expansion of CB-CD34(+) cells via downregulating intracellular reactive oxygen species level. J. Cell. Biochem. 2019, 120 (5), 7778-7787, Article. DOI: 10.1002/jcb.28052.
(294) Morikawa, T.; Xu, F. M.; Matsuda, H.; Yoshikawa, M. Structures of Novel Norstilbene Dimer, Longusone A, and Three New Stilbene Dimers, Longusols A, B, and C, with Antiallergic and Radical Scavenging Activities

from Egyptian Natural Medicine Cyperus longus. Chemical & pharmaceutical bulletin 2010, 58 (10), 1379-1385, Article. DOI: 10.1248/cpb.58.1379.

(295) Xiang, T.; Uno, T.; Ogino, F.; Ai, C. Q.; Duo, J.; Sankawa, U. Antioxidant constituents of Caragana tibetica. Chemical & pharmaceutical bulletin 2005, 53 (9), 1204-1206, Article. DOI: 10.1248/cpb.53.1204.

(296) Lu, Y.; Wang, A. H.; Shi, P.; Zhang, H. A Theoretical Study on the Antioxidant Activity of Piceatannol and Isorhapontigenin Scavenging Nitric Oxide and Nitrogen Dioxide Radicals. PloS one 2017, 12 (1), 16, Article. DOI: 10.1371/journal.pone.0169773.

(297) Liu, J.; Li, W. J.; Jiang, S. J.; Guo, Y.; Wang, G. L.; Lin, R. C. Phenolic Acids and Antioxidant Activities of Danshen Injection. Chemistry of Natural Compounds 2014, 50 (1), 83-87, Article. DOI: 10.1007/s10600-014-0872-5.

(298) Perez-Gonzalez, A.; Rebollar-Zepeda, A. M.; Leon-Carmona, J. R.; Galano, A. Reactivity Indexes and O-H Bond Dissociation Energies of a Large Series of Polyphenols: Implications for their Free Radical Scavenging Activity. Journal of the Mexican Chemical Society 2012, 56 (3), 241-249, Article.

(299) Lam, F.; Bradshaw, T. D.; Mao, H.; Roberts, S.; Pan, Y. J.; Wang, S. D. ZJU-6, a novel derivative of Erianin, shows potent anti-tubulin polymerisation and anti-angiogenic activities. Investigational New Drugs 2012, 30 (5), 1899-1907, Article. DOI: 10.1007/s10637-011-9755-9.

(300) Zhang, X. D.; Yu, Y. E.; Cen, Y. S.; Yang, D. F.; Qi, Z. C.; Hou, Z. N.; Han, S. L.; Cai, Z. X.; Liu, K. C. Bivariate Correlation Analysis of the Chemometric Profiles of Chinese Wild Salvia miltiorrhiza Based on UPLC-Qqq-MS and Antioxidant Activities. Molecules 2018, 23 (3), 13, Article. DOI: 10.3390/molecules23030538.

(301) Wang, Y. Q.; Zhuang, G.; Li, S. J. Multiple on-line screening and identification methods for hydroxyl radical scavengers in Yudanshen. Journal of Pharmaceutical and Biomedical Analysis 2018, 156, 278-283, Article. DOI: 10.1016/j.jpba.2018.04.043.

(302) Saez, V.; Pastene, E.; Vergara, C.; Mardones, C.; Hermosin-Gutierrez, I.; Gomez-Alonso, S.; Gomez, M. V.; Theoduloz, C.; Riquelme, S.; von Baer, D. Oligostilbenoids in Vitis vinifera L. Pinot Noir grape cane extract: Isolation, characterization, in vitro antioxidant capacity and anti-proliferative effect on cancer cells. Food Chemistry 2018, 265, 101-110, Article; Proceedings Paper. DOI: 10.1016/j.foodchem.2018.05.050.

(303) Sano, S.; Sugiyama, K.; Ito, T.; Katano, Y.; Ishihata, A. Identification of the Strong Vasorelaxing Substance Scirpusin B, a Dimer of Piceatannol, from Passion Fruit (Passiflora edulis) Seeds. Journal of Agricultural and Food Chemistry 2011, 59 (11), 6209-6213, Article. DOI: 10.1021/jf104959t.

(304) Lin, Y. L.; Chang, Y. Y.; Kuo, Y. H.; Shiao, M. S. Anti-lipid-peroxidative principles from Tournefortia sarmentosa. Journal of Natural Products 2002, 65 (5), 745-747, Article. DOI: 10.1021/np010538y.

(305) Chen, C. C.; Huang, Y. L.; Yeh, P. Y.; Ou, J. C. Cyclized geranyl stilbenes from the rhizomes of Helminthostachys zeylanica. Planta medica 2003, 69 (10), 964-967, Article.

(306) Zhang, R.; Kang, K. A.; Piao, M. J.; Lee, K. H.; Jang, H. S.; Park, M. J.; Kim, B. J.; Kim, J. S.; Kim, Y. S.; Ryu, S. Y.; et al. Rhapontigenin from Rheum undulatum protects against oxidative-stress-induced cell damage through antioxidant activity. Journal of Toxicology and Environmental Health-Part a-Current Issues 2007, 70 (13-14), 1155-1166, Article. DOI: 10.1080/15287390701252766.

(307) Yu, W. Z.; Tao, M. R.; Zhao, Y. L.; Hu, X. Q.; Wang, M. F. 4 '- Methoxyresveratrol Alleviated AGE-Induced Inflammation via RAGE-Mediated NF-kappa B and NLRP3 Inflammasome Pathway. Molecules 2018, 23 (6), 13, Article. DOI: 10.3390/molecules23061447.

(308) Tung, N. H.; Kwon, H. J.; Kim, J. H.; Ra, J. C.; Ding, Y.; Kim, J. A.; Kim, Y. H. Anti-influenza diarylheptanoids from the bark of Alnus japonica. Bioorganic & Medicinal Chemistry Letters 2010, 20 (3), 1000-1003, Article. DOI: 10.1016/j.bmcl.2009.12.057.

(309) Huang, K. S.; Zhou, S.; Lin, M.; Wang, Y. H. An isorhapontigenin tetramer and a novel stilbene dimer from Gnetum hainanense. Planta Med 2002, 68 (10), 916-920. DOI: 10.1055/s-2002-34951.

(310) Iliya, I.; Ali, Z.; Tanaka, T.; Iinuma, M.; Furasawa, M.; Nakaya, K.; Shirataki, Y.; Murata, J.; Darnaedi, D.; Matsuura, N.; et al. Three new trimeric stilbenes from Gnetum gnemon. Chemical & pharmaceutical bulletin 2003, 51 (1), 85-88, Article. DOI: 10.1248/cpb.51.85.

(311) Yao, C. S.; Lin, M.; Wang, L. Isolation and biomimetic synthesis of anti-inflammatory stilbenolignans from Gnetum cleistostachyum. Chem. Pharm. Bull. 2006, 54 (7), 1053-1057, Article. DOI: 10.1248/cpb.54.1053.

(312) Dai, S. J.; Mi, Z. M.; Ma, Z. B.; Li, S.; Chen, R. Y.; Yu, D. Q. Bioactive Diels-Alder type adducts from the stem bark of Morus macroura. Planta medica 2004, 70 (8), 758-763, Article. DOI: 10.1055/s-2004-827208.

(313) Cheng, K. J.; Liu, X.; Chen, L.; Lv, J. M.; Qu, F. J.; Pan, X. W.; Li, L.; Cui, X. G.; Gao, Y.; Xu, D. F. alpha-Viniferin activates autophagic apoptosis and cell death by reducing glucocorticoid receptor expression in castration-resistant prostate cancer cells. Medical Oncology 2018, 35 (7), 11, Article. DOI: 10.1007/s12032-018-1163-y.

(314) Ren, X. Y.; Yu, J.; Yin, X. F.; Tu, L. D.; Kong, Q. J. Isolation and purification of five phenolic compounds from the Xinjiang wine grape (Vitis Vinifera) and determination of their antioxidant mechanism at cellular level. European Food Research and Technology 2018, 244 (9), 1569-1579, Article. DOI: 10.1007/s00217-018-3070-z.

(315) He, C.-N.; Bi, W.; Shen, J.; Peng, Y.; Xiao, P.-G. Determination of ten stilbenes and their antioxidant activity of peony seed coat, seed kernel and seed coat extracts. Zhongguo Zhong yao za zhi = Zhongguo zhongyao zazhi = China journal of Chinese materia medica 2016, 41 (6), 1081-1086. DOI: 10.4268/cjcmm20160618.

(316) Ge, H. M.; Yang, W. H.; Zhang, J.; Tan, R. X. Antioxidant Oligostilbenoids from the Stem Wood of Hopea hainanensis. Journal of Agricultural and Food Chemistry 2009, 57 (13), 5756-5761, Article. DOI: 10.1021/jf900756d.

(317) Dai, J. R.; Hallock, Y. F.; Cardellina, J. H.; Boyd, M. R. HIV-inhibitory and cytotoxic oligostilbenes from the leaves of Hopea malibato. J. Nat. Prod. 1998, 61 (3), 351-353, Article. DOI: 10.1021/np970519h.

(318) Li, X. C.; Xie, Y. L.; Xie, H.; Yang, J.; Chen, D. F. pi-pi Conjugation Enhances Oligostilbene's Antioxidant Capacity: Evidence from alpha-Viniferin and Caraphenol A. Molecules 2018, 23 (3), 10, Article. DOI: 10.3390/molecules23030694.

(319) Jin, Q.; Han, X. H.; Hong, S. S.; Lee, C.; Choe, S.; Lee, D.; Kim, Y.; Hong, J. T.; Lee, M. K.; Hwang, B. Y. Antioxidative oligostilbenes from Caragana sinica. Bioorg Med Chem Lett 2012, 22 (2), 973-976. DOI: 10.1016/j.bmcl.2011.12.012.

(320) Wang, G. W.; Wang, H. L.; Capretto, D. A.; Han, Q.; Hu, R. B.; Yang, S. D. Tf2O-catalyzed Friedel-Crafts alkylation to synthesize dibenzo a,d cycloheptene cores and application in the total synthesis of Diptoindonesin D, Pauciflorial F, and (+/-)-Ampelopsin B. Tetrahedron 2012, 68 (26), 5216-5222, Article. DOI: 10.1016/j.tet.2012.03.073.

(321) Matsuura, B. S.; Keylor, M. H.; Li, B.; Lin, Y. X.; Allison, S.; Pratt, D. A.; Stephenson, C. R. J. A Scalable Biomimetic Synthesis of Resveratrol Dimers and Systematic Evaluation of their Antioxidant Activities. Angewandte Chemie-International Edition 2015, 54 (12), 3754-3757, Article. DOI: 10.1002/anie.201409773.

(322) Li, C.; Xu, X. F.; Tab, Z. H.; Wang, X. J.; Pan, Y. J. Resveratrol dimers, nutritional components in grape wine, are selective ROS scavengers and weak Nrf2 activators. Food Chemistry 2015, 173, 218-223, Article. DOI: 10.1016/j.foodchem.2014.09.165.

(323) Chen, C. J.; Jiang, R.; Wang, G.; Jiao, R. H.; Tancharoen, C.; Sudto, K.; Vajarothai, S.; Hannongbua, S.; Ge, H. M.; Tan, R. X. Oligostilbenoids with Acetylcholinesterase Inhibitory Activity from Dipterocarpus alatus. Planta medica 2014, 80 (17), 1641-1646, Article. DOI: 10.1055/s-0034-1383194.

(324) Chen, J.; Ma, M.; Lu, Y.; Wang, L.; Wu, C.; Duan, H. Rhaponticin from rhubarb rhizomes alleviates liver steatosis and improves blood glucose and lipid profiles in KK/Ay diabetic mice. Planta Med 2009, 75 (5), 472-477. DOI: 10.1055/s-0029-1185304.

(325) Yao, C. S.; Lin, M.; Yang, Q. Y. A new resveratrol trimer derivative from Gnetum brunonianum. Journal of Asian Natural Products Research 2012, 14 (9), 918-922, Article. DOI: 10.1080/10286020.2012.695350.

(326) Erasalo, H.; Hamalainen, M.; Leppanen, T.; Maki-Opas, I.; Laavola, M.; Haavikko, R.; Yli-Kauhaluoma, J.; Moilanen, E. Natural Stilbenoids Have Anti-Inflammatory Properties in Vivo and Down-Regulate the Production of Inflammatory Mediators NO, IL6, and MCP1 Possibly in a PI3K/Akt-Dependent Manner. Journal of Natural Products 2018, 81 (5), 1131-1142, Article. DOI: 10.1021/acs.jnatprod.7b00384.

(327) Lai, Y.; Zhou, C.; Huang, P.; Dong, Z.; Mo, C.; Xie, L.; Lin, H.; Zhou, Z.; Deng, G.; Liu, Y.; et al. Polydatin alleviated alcoholic liver injury in zebrafish larvae through ameliorating lipid metabolism and oxidative stress. Journal of pharmacological sciences 2018, 138 (1), 46-53. DOI: 10.1016/j.jphs.2018.08.007.

(328) Lai, Y. L.; Zhou, C. Y.; Huang, P.; Dong, Z. Y.; Mo, C.; Xie, L. P.; Lin, H. Y.; Zhou, Z. T.; Deng, G. H.; Liu, Y.; et al. Polydatin alleviated alcoholic liver injury in zebrafish larvae through ameliorating lipid metabolism and oxidative stress. Journal of Pharmacological Sciences 2018, 138 (1), 46-53, Article. DOI: 10.1016/j.jphs.2018.08.007.

(329) Park, S. Y.; Jin, M. L.; Wang, Z.; Park, G.; Choi, Y. W. 2,3,4 ',5-tetrahydroxystilbene-2-O-beta-D-glucoside exerts anti-inflammatory effects on lipopolysaccharide-stimulated microglia by inhibiting NF-kappa B and activating AMPK/Nrf2 pathways. Food and Chemical Toxicology 2016, 97, 159-167, Article. DOI: 10.1016/j.fct.2016.09.010.

(330) Xu, S. F.; Liu, J.; Shi, J. S.; Wang, Z. T.; Ji, L. L. 2,3,4 ',5-tetrahydroxystilbene-2-O-beta-D-glucoside exacerbates acetaminophen-induced hepatotoxicity by inducing hepatic expression of CYP2E1, CYP3A4 and CYP1A2. Scientific Reports 2017, 7, 13, Article. DOI: 10.1038/s41598-017-16688-5.

(331) Lee, J. P.; Min, B. S.; An, R. B.; Na, M. K.; Lee, S. M.; Lee, H. K.; Kim, J. G.; Bae, K. H.; Kang, S. S. Stilbenes from the roots of Pleuropterus ciliinervis and their antioxidant activities. Phytochemistry 2003, 64 (3), 759-763, Article. DOI: 10.1016/s0031-9422(03)00417-5.

(332) Park, D.; Kim, H. J.; Jung, S. Y.; Yook, C. S.; Jin, C.; Lee, Y. S. A New Diarylheptanoid Glycoside from the Stem Bark of Alnus hirsuta and Protective Effects of Diarylheptanoid Derivatives in Human HepG2 Cells. Chemical & pharmaceutical bulletin 2010, 58 (2), 238-241, Article. DOI: 10.1248/cpb.58.238.

(333) Lee, M.; Song, J. Y.; Chin, Y. W.; Sung, S. H. Anti-adipogenic diarylheptanoids from Alnus hirsuta f. sibirica on 3T3-L1 cells. Bioorganic & Medicinal Chemistry Letters 2013, 23 (7), 2069-2073, Article. DOI: 10.1016/j.bmcl.2013.01.127.

(334) Wang, L.; Cui, X. Q.; Gong, T.; Yan, R. Y.; Tan, Y. X.; Chen, R. Y. Three new compounds from the barks of Morus nigra. Journal of Asian Natural Products Research 2008, 10 (9), 897-902.

(335) Takasugi, M.; Ishikawa, S. I.; Masamune, T. Albafurans A and B, geranyl 2-phenylbenzofurans from mulberry. Chemistry Letters 1982, (8), 1221-1222.

(336) Rong, Y. Z.; Wang, Z. W.; Zhao, B. DFT-Based Quantum Chemical Studies on Conformational, Electronic and Antioxidant Properties of Isobavachalcone and 4-Hydroxyderricin. Food Biophys. 2013, 8 (4), 250-255, Article. DOI: 10.1007/s11483-013-9296-1.

(337) Jung, H. A.; Jeong, D. M.; Chung, H. Y.; Lim, H. A.; Kim, J. Y.; Yoon, N. Y.; Choi, J. S. Re-evaluation of the antioxidant prenylated flavonoids from the roots of Sophora flavescens. Biol Pharm Bull 2008, 31 (5), 908-915.

(338) Gaur, R.; Yadav, K. S.; Verma, R. K.; Yadav, N. P.; Bhakuni, R. S. In vivo anti-diabetic activity of derivatives of isoliquiritigenin and liquiritigenin. Phytomedicine 2014, 21 (4), 415-422.

(339) Kim, Y. M.; Kim, T. H.; Kim, Y. W.; Yang, Y. M.; Ryu, D. H.; Hwang, S. J.; Lee, J. R.; Kim, S. C.; Kim, S. G. Inhibition of liver X receptor-Î±-dependent hepatic steatosis by isoliquiritigenin, a licorice antioxidant flavonoid, as mediated by JNK1 inhibition. Free Radic Biol Med 2010, 49 (11), 1722-1734.

(340) Zhan, C.; Yang, J. Protective effects of isoliquiritigenin in transient middle cerebral artery occlusion-induced focal cerebral ischemia in rats. Pharmacological Research 2006, 53 (3), 303-309.

(341) Zhou, Y. Z.; Li, X.; Gong, W. X.; Tian, J. S.; Gao, X. X.; Gao, L.; Zhang, X.; Du, G. H.; Qin, X. M. Protective effect of isoliquiritin against corticosterone-induced neurotoxicity in PC12 cells. Food & Function 2017, 8 (3), 1235.

(342) Chen, J. J.; Cheng, M. J.; Shu, C. W.; Sung, P. J.; Lim, Y. P.; Cheng, L. Y.; Wang, S. L.; Chen, L. C. A New Chalcone and Antioxidant Constituents of Glycyrrhiza glabra. Chemistry of Natural Compounds 2017, 53 (4), 632-634.

(343) Cheng, Z. J.; Kuo, S. C.; Chan, S. C.; Ko, F. N.; Teng, C. M. Antioxidant properties of butein isolated from Dalbergia odorifera. Biochim Biophys Acta 1998, 1392 (2–3), 291-299.

(344) Nakanishi, N.; Fukuoh, A.; Kang, D.; Iwai, S.; Kuraoka, I. Effects of DNA lesions on the transcription reaction of mitochondrial RNA polymerase: implications for bypass RNA synthesis on oxidative DNA lesions. Mutagenesis 2013, 28 (1), 117-123. DOI: 10.1093/mutage/ges060.

(345) Tian, X.; Liu, C.; Jiang, H. L.; Yan, Z.; Han, J.; Ju, L.; Meng, C. Cardioprotection provided by Echinatin against ischemia/reperfusion in isolated rat hearts. Bmc Cardiovasc Disord 2016, 16 (1), 119.

(346) Lv, H.; Xiao, Q.; Zhou, J.; Feng, H.; Liu, G.; Ci, X. Licochalcone A Upregulates Nrf2 Antioxidant Pathway and Thereby Alleviates Acetaminophen-Induced Hepatotoxicity. Frontiers in Pharmacology 2018, 9, 147.

(347) Han, J.; Wang, D.; Yu, B.; Wang, Y.; Ren, H.; Zhang, B.; Wang, Y.; Zheng, Q. Cardioprotection against ischemia/reperfusion by licochalcone B in isolated rat hearts. Oxidative Medicine & Cellular Longevity 2014, 2014, 134862.

(348) Zhou, M.; Liu, L.; Wang, W.; Han, J.; Ren, H.; Zheng, Q.; Wang, D. Role of licochalcone C in cardioprotection against ischemia/reperfusion injury of isolated rat heart via antioxidant, anti-inflammatory, and anti-apoptotic activities. Life Sciences 2015, 132, 27-33.

(349) Yuan, X.; Niu, H.; Wang, P.; Lu, J.; Zhao, H.; Liu, S.; Zheng, Q.; Li, C. Cardioprotective Effect of Licochalcone D against Myocardial Ischemia/Reperfusion Injury in Langendorff-Perfused Rat Hearts. PloS one 2015, 10 (6), e0128375.

(350) Kim, S. S.; Lim, J.; Bang, Y.; Gal, J.; Lee, S. U.; Cho, Y. C.; Yoon, G.; Kang, B. Y.; Cheon, S. H.; Choi, H. J. Licochalcone E activates Nrf2/antioxidant response element signaling pathway in both neuronal and microglial cells: therapeutic relevance to neurodegenerative disease. Journal of Nutritional Biochemistry 2012, 23 (10), 1314-1323.

(351) Victor; Kuete; Jaures; Noumedem; Frederic; Nana. Chemistry and Pharmacology of 4-Hydroxylonchocarpin: A Review. Chinese Journal of Integrative Medicine 2013, 19 (6), 475-480.

(352) Adem, F. A.; Kuete, V.; Mbaveng, A. T.; Heydenreich, M.; Koch, A.; Ndakala, A.; Irungu, B.; Yenesew, A.; Efferth, T. Cytotoxic flavonoids from two Lonchocarpus species. Natural product research 2018, 1-9.

(353) Dang, Y.; Ling, S.; Duan, J.; Ma, J.; Ni, R.; Xu, J. W. Bavachalcone-induced manganese superoxide dismutase expression through the AMP-activated protein kinase pathway in human endothelial cells. Pharmacology 2015, 95 (3-4), 105.

(354) Shakya, R.; Ye, J.; Rommens, C. M. Altered leaf colour is associated with increased superoxide-scavenging activity in aureusidin-producing transgenic plants. Plant Biotechnology Journal 2012, 10 (9), 1046-1055.

(355) Calis, I.; Kuruüzüm, A.; Demirezer, L. O.; Sticher, O.; Ganci, W.; Rüedi, P. Phenylvaleric acid and flavonoid glycosides from Polygonum salicifolium. Journal of Natural Products 1999, 62 (8), 1101.

(356) Cioffi, G.; Escobar, L. A.; De, T. N. Antioxidant chalcone glycosides and flavanones from Maclura (Chlorophora) tinctoria. Journal of Natural Products 2003, 66 (8), 1061-1064.

(357) Cheng, Z.; Lin, C.; Hwang, T.; Teng, C. Broussochalcone A, a potent antioxidant and effective suppressor of inducible nitric oxide synthase in lipopolysaccharide-activated macrophages. Biochemical Pharmacology 2001, 61 (8), 939-946.

(358) Padhye, S.; Ahmad, A.; Oswal, N.; Sarkar, F. H. Emerging role of Garcinol, the antioxidant chalcone from Garcinia indica Choisy and its synthetic analogs. Journal of Hematology & Oncology 2009, 2 (1), 38.

(359) Isa, N. M.; Abdelwahab, S. I.; Mohan, S.; Abdul, A. B.; Sukari, M. A.; Taha, M. M.; Syam, S.; Narrima, P.; Sch, C.; Ahmad, S. In vitro anti-inflammatory, cytotoxic and antioxidant activities of boesenbergin A, a chalcone isolated from Boesenbergia rotunda (L.) (fingerroot). Brazilian Journal of Medical & Biological Research 2012, 45 (6), 524-530.

(360) Wang, C.; Ma, H.; Zhang, S.; Wang, Y.; Liu, J.; Xiao, X. Safflor yellow B suppresses pheochromocytoma cell (PC12) injury induced by oxidative stress via antioxidant system and Bcl-2 /Bax pathway. Naunyn-Schmiedeberg's Archives of Pharmacology 2009, 380 (2), 135-142.

(361) Li, Y.; Piao, D.; Zhang, H.; Kim, T.; Lee, S. H.; Chang, H. W.; Woo, M. H.; Son, J. K. Quality evaluation of Carthami Flos by HPLC-UV. Archives of Pharmacal Research 2015, 38 (5), 1-9.

(362) Yue, S.; Tang, Y.; Xu, C.; Li, S.; Zhu, Y.; Duan, J. A. Two New Quinochalcone C-Glycosides from the Florets of Carthamus tinctorius. International Journal of Molecular Sciences 2014, 15 (9), 16760-16771.

(363) Yoon, H. R.; Paik, Y. S. Radical-Scavenging Activities of Four Quinochalcones of Safflower. Journal of the Korean Society for Applied Biological Chemistry 2008, 51 (4), 346-348.

(364) Pinner, K. D.; Wales, C. T. K.; Gristock, R. A.; Vo, H. T.; So, N.; Jacobs, A. T. Flavokawains A and B from kava (Piper methysticum) activate heat shock and antioxidant responses and protect against hydrogen peroxide-induced cell death in HepG2 hepatocytes. Pharmaceutical Biology 2016, 54 (9), 1.

(365) Li, N.; Liu, J. H.; Zhang, J.; Yu, B. Y. Comparative Evaluation of Cytotoxicity and Antioxidative Activity of 20 Flavonoids. J Agric Food Chem 2008, 56 (10), 3876-3883.

(366) Voon, F.-L.; Sulaiman, M. R.; Akhtar, M. N.; Idris, M. F.; Akira, A.; Perimal, E. K.; Israf, D. A.; Ming-Tatt, L. Cardamonin (2′,4′-dihydroxy-6′-methoxychalcone) isolated from Boesenbergia rotunda (L.) Mansf. inhibits CFA-induced rheumatoid arthritis in rats. European Journal of Pharmacology 2017, 794, 127-134.

(367) De, S. S.; Eckers, A.; Wehrend, C.; Micoogullari, M.; Sies, H.; Stahl, W.; Steinbrenner, H. Interplay between the chalcone cardamonin and selenium in the biosynthesis of Nrf2-regulated antioxidant enzymes in intestinal Caco-2 cells. Free radical biology & medicine 2016, 91 (1-2), 164-171.

(368) Wen, R.; Lv, H. N.; Jiang, Y.; Tu, P. F. Anti-inflammatory flavone and chalcone derivatives from the roots of Pongamia pinnata (L.) Pierre. Phytochemistry 2018, 149, 56.

(369) Lima, N. M.; Andrade, J. I.; Lima, K. C.; dos Santos, F. N.; Barison, A.; Salomé, K. S.; Matsuura, T.; Nunez, C. V. Chemical profile and biological activities of Deguelia duckeana A.M.G. Azevedo (Fabaceae). Natural product research 2013, 27 (4-5), 425-432.

(370) Saha, M. M.; Mallik, U. K.; Mallik, A. K. A chromenoflavanone and two caffeic esters from Pongamia glabra. Phytochemistry 1991, 30 (11), 3834-3836.

(371) Magalhães, A. F.; Tozzi, A. M. G. A.; Sales, B. H. L. N.; Magalhães, E. G. Twenty-three flavonoids from Lonchocarpus subglaucescens. Phytochemistry 1996, 42 (5), 1459-1471.

(372) Sritularak, B.; Likhitwitayawuid, K. Flavonoids from the pods of Millettia erythrocalyx. Phytochemistry 2006, 67 (8), 812-817.

(373) Chang, L. C.; Gerhäuser, C.; Song, L.; Farnsworth, N. R.; Pezzuto, J. M.; Kinghorn, A. D. Activity-guided isolation of constituents of Tephrosia purpurea with the potential to induce the phase II enzyme, quinone reductase. Journal of Natural Products 1997, 60 (9), 869.

(374) Caamalfuentes, E. E.; Perazasánchez, S. R.; Torrestapia, L. W.; Moopuc, R. E. Isolation and Identification of Cytotoxic Compounds from Aeschynomene fascicularis, a Mayan Medicinal Plant. Molecules 2015, 20 (8), 13563-13574.

(375) Kim, Y. E.; Choi, H. C.; Lee, I. C.; Yuk, D. Y.; Lee, H.; Choi, B. Y. 3-Deoxysappanchalcone Promotes Proliferation of Human Hair Follicle Dermal Papilla Cells and Hair Growth in C57BL/6 Mice by Modulating WNT/β-Catenin and STAT Signaling. Biomolecules & Therapeutics 2016, 24 (6), 572-580.

(376) Seo, H.; Kim, S.; Mahmud, H. A.; Islam, M. I.; Nam, K. W.; Lee, B. E.; Lee, H.; Cho, M. L.; Shin, H. M.; Song, H. Y. In vitro Antitubercular Activity of 3-Deoxysappanchalcone Isolated From the Heartwood of Caesalpinia sappan Linn. Phytotherapy Research Ptr 2017, 31 (1).

(377) Seo, K. H.; Lee, D. Y.; Jeong, R. H.; Lee, D. S.; Kim, Y. E.; Hong, E. K.; Kim, Y. C.; Baek, N. I. Neuroprotective effect of prenylated arylbenzofuran and flavonoids from morus alba fruits on glutamate-induced oxidative injury in HT22 hippocampal cells. Journal of medicinal food 2015, 18 (4), 403.

(378) Jiang, Y.; Zhang, G.; Yan, D.; Yang, H.; Ye, Z.; Ma, T. Bioactivity-Guided Fractionation of the Traditional Chinese Medicine Resina Draconis Reveals Loureirin B as a PAI-1 Inhibitor. Evid Based Complement Alternat Med 2017, 2017, 9425963. DOI: 10.1155/2017/9425963.

(379) Wang, X. H. Production of dragon's blood in Dracaena cochinchinensis plants by inoculation of Fusarium proliferatum. Plant Science 2011, 180 (2), 292-299.

(380) Tang, N.; Yan, W. Solubilities of Naringin Dihydrochalcone in Pure Solvents and Mixed Solvents at Different Temperatures. 2016.

(381) Rezk, B. M.; Haenen, G. R.; Wj, V. D. V.; Bast, A. The antioxidant activity of phloretin: the disclosure of a new antioxidant pharmacophore in flavonoids. Biochemical & Biophysical Research Communications 2002, 295 (1), 9-13.

(382) Zhao, Y. M.; Wang, J.; Song, G. M.; Song, X. B.; Liu, D. L.; Xie, W. L. Study on the Dynamic Changs of Phlordzin and Phloretin in Leave and Branches of Apple Tree. Food Research & Development 2013.

(383) Shindo, K.; Kato, M.; Kinoshita, A.; Kobayashi, A.; Koike, Y. Analysis of Antioxidant Activities Contained in theBoesenbergia pandurataSchult. Rhizome. Bioscience, Biotechnology, and Biochemistry 2014, 70 (9), 2281-2284. DOI: 10.1271/bbb.60086.

(384) Sun, Y.; Li, W.; Liu, Z. Preparative isolation, quantification and antioxidant activity of dihydrochalcones from Sweet Tea (Lithocarpus polystachyus Rehd.). Journal of Chromatography B 2015, 1002, 372-378. DOI: 10.1016/j.jchromb.2015.08.045.

(385) Tian, Y.; Li, X.; Xie, H.; Wang, X.; Xie, Y.; Chen, C.; Chen, D. Protective Mechanism of the Antioxidant Baicalein toward Hydroxyl Radical-Treated Bone Marrow-Derived Mesenchymal Stem Cells. Molecules 2018, 23 (1). DOI: 10.3390/molecules23010223.

(386) Ahmed, S. I.; Hayat, M. Q.; Tahir, M.; Mansoor, Q.; Ismail, M.; Keck, K.; Bates, R. B. Pharmacologically active flavonoids from the anticancer, antioxidant and antimicrobial extracts of Cassia angustifolia Vahl. BMC Complement. Altern. Med. 2016, 16.

(387) Fonseca, S. F.; Lima, D. B.; Alves, D.; Jacob, R. G.; Perin, G.; Lenardao, E. J.; Savegnago, L. Synthesis, characterization and antioxidant activity of organoselenium and organotellurium compound derivatives of chrysin. New Journal of Chemistry 2015, 39 (4), 3043-3050.

(388) Wang CZ; Yuan HH; Bao XL; MB., L. In vitro antioxidant and cytotoxic properties of ethanol extract of Alpinia oxyphylla fruits. Pharm Biol 2013, 51 (11), 1419–1425.

(389) Shieh, D. E.; Liu, L. T.; Lin, C. C. Antioxidant and free radical scavenging effects of baicalein, baicalin and wogonin. Anticancer Research 2000, 20 (5a), 2861-2865.

(390) Dong, L. L.; Fu, Y. J.; Zu, Y. G.; Luo, M.; Wang, W.; Li, C. Y.; Mu, P. S. An enhanced preparation and purification of the major antioxidants baicalein and wogonin from Scutellariae radix. Food Chem. 2012, 133 (2), 430-436.

(391) Gao, Y.; Zhao, J. T.; Zu, Y. G.; Fu, Y. J.; Liang, L.; Luo, M.; Wang, W.; Efferth, T. Antioxidant properties, superoxide dismutase and glutathione reductase activities in HepG2 cells with a fungal endophyte producing apigenin from pigeon pea [Cajanus cajan (L.) Millsp.]. Food Res Int 2012, 49 (1), 147-152.

(392) Zheng, R. L.; Xing, G. X.; Ju, Y.; Jia, Z. J. Flavonoids from Saussurea stella Maxim as superoxide scavengers and antioxidants. Indian Journal of Chemistry Section B-Organic Chemistry Including Medicinal Chemistry 1997, 36 (12), 1201-1203.

(393) Hu, J.; Ma, W.; Li, N.; Wang, K. J. Antioxidant and Anti-Inflammatory Flavonoids from the Flowers of Chuju, a Medical Cultivar of Chrysanthemum Morifolim Ramat. Journal of the Mexican Chemical Society 2017, 61 (4), 282-289, Article.

(394) Chen, Z. J.; Kong, S. S.; Song, F. F.; Li, L. P.; Jiang, H. D. Pharmacokinetic study of luteolin, apigenin, chrysoeriol and diosmetin after oral administration of Flos Chrysanthemi extract in rats. Fitoterapia 2012, 83 (8), 1616-1622.

(395) Panuccio, M. R.; Fazio, A.; Papalia, T.; Barreca, D. Antioxidant Properties and Flavonoid Profile in Leaves of CalabrianLavandula multifidaL., an Autochthon Plant of Mediterranean Southern Regions. Chemistry & Biodiversity 2016, 13 (4), 416-421. DOI: 10.1002/cbdv.201500115.

(396) Liao, W. Z.; Ning, Z. X.; Chen, L. Y.; Wei, Q. Y.; Yuan, E. D.; Yang, J. G.; Ren, J. Y. Intracellular Antioxidant Detoxifying Effects of Diosmetin on 2,2-Azobis(2-amidinopropane) Dihydrochloride (AAPH)-Induced Oxidative Stress through Inhibition of Reactive Oxygen Species Generation. Journal of Agricultural and Food Chemistry 2014, 62 (34), 8648-8654.

(397) Zhang, C.-F.; Zhang, S.-L.; He, X.; Yang, X.-L.; Wu, H.-T.; Lin, B.-Q.; Jiang, C.-P.; Wang, J.; Yu, C.-H.; Yang, Z.-L.; et al. Antioxidant effects of Genkwa flos flavonoids on Freund′s adjuvant-induced rheumatoid arthritis in rats. Journal of Ethnopharmacology 2014, 153 (3), 793-800. DOI: 10.1016/j.jep.2014.03.046.

(398) Yang, J.; Kim, J. S.; Jeong, H. J.; Kang, H. H.; Cho, J. C.; Yeom, H. M.; Kim, M. J. Determination of antioxidant and alpha-glucosidase inhibitory activities and luteolin contents of Chrysanthemum morifolium Ramat extracts. African Journal of Biotechnology 2011, 10 (82), 19197-19202.

(399) Gokbulut, A.; Satilmis, B.; Batcioglu, K.; Cetin, B.; Sarer, E. Antioxidant activity and luteolin content of Marchantia polymorpha L. Turkish Journal of Biology 2012, 36 (4), 381-385.

(400) An, F.; Yang, G. D.; Tian, J. M.; Wang, S. H. Antioxidant effects of the orientin and vitexin in Trollius chinensis Bunge in D-galactose-aged mice. Neural Regeneration Research 2012, 7 (33), 2565-2575.

(401) Gu, C. B.; Ma, H.; Ning, W. J.; Niu, L. L.; Han, H. Y.; Yuan, X. H.; Fu, Y. J. Characterization, culture medium optimization and antioxidant activity of an endophytic vitexin-producing fungus Dichotomopilus funicola Y3 from pigeon pea [Cajanus cajan (L.) Millsp.]. Journal of Applied Microbiology 2018, 125 (4), 1054-1065.

(402) Yang, P.; Kong, X. Z.; Cheng, C. M.; Li, C. Y.; Yang, X. M.; Zhao, X. E. Synthesis and biological evaluation of 8-substituted and deglucuronidated scutellarin and baicalin analogues as antioxidant responsive element activators. Science China-Chemistry 2011, 54 (10), 1565-1575.

(403) Rehecho, S.; Hidalgo, O.; de Cirano, M. G. I.; Navarro, I.; Astiasaran, I.; Ansorena, D.; Cavero, R. Y.; Calvo, M. I. Chemical composition, mineral content and antioxidant activity of Verbena officinalis L. Lwt-Food Science and Technology 2011, 44 (4), 875-882.

(404) Tuzun, B. S.; Hajdu, Z.; Orban-Gyapai, O.; Zomborszki, Z. P.; Jedlinszki, N.; Forgo, P.; Kivcak, B.; Hohmann, J. Isolation of Chemical Constituents of Centaurea virgata Lam. and Xanthine Oxidase Inhibitory Activity of the Plant Extract and Compounds. Medicinal Chemistry 2017, 13 (5), 498-502, Article. DOI: 10.2174/1573406413666161219161946.

(405) Peng, Z. F.; Strack, D.; Baumert, A.; Subramaniam, R.; Goh, N. K.; Chia, T. F.; Tan, S. N.; Chia, L. S. Antioxidant flavonoids from leaves of Polygonum hydropiper L. Phytochemistry 2003, 62 (2), 219-228, Article. DOI: 10.1016/s0031-9422(02)00504-6.

(406) Venditti, A.; Frezza, C.; Bianco, A.; Serafini, M.; Cianfaglione, K.; Nagy, D. U.; Iannarelli, R.; Caprioli, G.; Maggi, F. Polar Constituents, Essential Oil and Antioxidant Activity of Marsh Woundwort (Stachys palustris L.). Chemistry & Biodiversity 2017, 14 (3), 8, Article. DOI: 10.1002/cbdv.201600401.

(407) Fu, M. Q.; Xu, Y. J.; Chen, Y. L.; Wu, J. J.; Yu, Y. S.; Zou, B.; An, K. J.; Xiao, G. S. Evaluation of bioactive flavonoids and antioxidant activity in Pericarpium Citri Reticulatae (Citrus reticulata 'Chachi') during storage. Food Chemistry 2017, 230, 649-656, Article. DOI: 10.1016/j.foodchem.2017.03.098.

(408) Mehmood, T.; Khan, M. R.; Shabbir, M. A.; Zia, M. A. Phytochemical profiling and HPLC quantification of citrus peel from different varieties. Progress in Nutrition 2018, 20, 279-288, Article. DOI: 10.23751/pn.v20i1-S.6357.

(409) Wu, C.; Zhao, J.; Chen, Y.; Li, T.; Zhu, R.; Zhu, B.; Zhang, Y. Tangeretin protects human brain microvascular endothelial cells against oxygen-glucose deprivation-induced injury. Journal of cellular biochemistry 2018. DOI: 10.1002/jcb.27762.

(410) Debnath, T.; Park, P.-J.; Deb Nath, N. C.; Samad, N. B.; Park, H. W.; Lim, B. O. Antioxidant activity of Gardenia jasminoides Ellis fruit extracts. Food Chemistry 2011, 128 (3), 697-703. DOI: 10.1016/j.foodchem.2011.03.090.

(411) Jian, S.; Xue-Mei, H.; Mou-Ming, Z.; Li, L.; Chang-Bao, L.; Yi, D. Antioxidant and nitrite-scavenging capacities of phenolic compounds from sugarcane (Saccharum officinarum L.) tops. Molecules 2014, 19 (9), 13147-13160.

(412) Suksamrarn, A.; Poomsing, P.; Aroonrerk, N.; Punjanon, T.; Suksamrarn, S.; Kongkun, S. Antimycobacterial and antioxidant flavones fromLimnophila geoffrayi. Archives of Pharmacal Research 2003, 26 (10), 816-820. DOI: 10.1007/bf02980026.

(413) Cypriani, B.; Limasset, B.; Carrie, M. L.; Doucen, C. L.; Roussie, M.; Paulet, A. D.; Damon, M. Antioxidant activity of micronized diosmin on oxygen species from stimulated human neutrophils. Biochemical Pharmacology 1993, 45 (7), 1531-1535.

(414) Dwiecki, K.; Neunert, G.; Polewski, P.; Polewski, K. Antioxidant activity of daidzein, a natural antioxidant, and its spectroscopic properties in organic solvents and phosphatidylcholine liposomes. J Photoch Photobio B 2009, 96 (3), 242-248.

(415) Zhang, D. Y.; Zu, Y. G.; Fu, Y. J.; Luo, M.; Gu, C. B.; Wang, W.; Yao, X. H. Negative pressure cavitation extraction and antioxidant activity of biochanin A and genistein from the leaves of Dalbergia odorifera T. Chen. Sep Purif Technol 2011, 83, 91-99.

(416) Chen, C. Y.; Zu, Y. G.; Fu, Y. J.; Luo, M.; Zhao, C. J.; Wang, W.; Zhao, B. S.; Li, J.; Efferth, T. Preparation and antioxidant activity of Radix Astragali residues extracts rich in calycosin and formononetin. Biochem Eng J 2011, 56 (1-2), 84-93.

(417) Chen, Y.; Chang, S. K. C. Macronutrients, Phytochemicals, and Antioxidant Activity of Soybean Sprout Germinated with or without Light Exposure. Journal of Food Science 2015, 80 (6), S1391-S1398. DOI: 10.1111/1750-3841.12868.

(418) Jung, H. A.; Kim, A. R.; Chung, H. Y.; Choi, J. S. In vitro antioxidant activity of some selectedprunus species in Korea. Archives of Pharmacal Research 2002, 25 (6), 865-872. DOI: 10.1007/bf02977006.

(419) Shi, S. Y.; Ma, Y. J.; Zhang, Y. P.; Liu, L. L.; Liu, Q.; Peng, M. J.; Xiong, X. Systematic separation and purification of 18 antioxidants from Pueraria lobata flower using HSCCC target-guided by DPPH-HPLC experiment. Separation and Purification Technology 2012, 89, 225-233, Article. DOI: 10.1016/j.seppur.2012.01.041.

(420) Xu, H. Y.; Zheng, H. C.; Zhang, H. W.; Zhang, J. Y.; Ma, C. M. Comparison of Antioxidant Constituents of Agriophyllum squarrosum Seed with Conventional Crop Seeds. Journal of Food Science 2018, 83 (7), 1823-1831, Article. DOI: 10.1111/1750-3841.14159.

(421) Fu, Y.; Chen, J.; Li, Y. J.; Zheng, Y. F.; Li, P. Antioxidant and anti-inflammatory activities of six flavonoids separated from licorice. Food Chem. 2013, 141 (2), 1063-1071.

(422) Ji, S.; Li, Z.; Song, W.; Wang, Y.; Liang, W.; Li, K.; Tang, S.; Wang, Q.; Qiao, X.; Zhou, D. Bioactive Constituents of Glycyrrhiza uralensis (Licorice): Discovery of the Effective Components of a Traditional Herbal Medicine. Journal of Natural Products 2017, 79 (2), 281.

(423) Qiu, D.; Guo, J.; Yu, H.; Yan, J.; Yang, S.; Li, X.; Zhang, Y.; Sun, J.; Cong, J.; He, S.; et al. Antioxidant phenolic compounds isolated from wild Pyrus ussuriensis Maxim. fruit peels and leaves. Food Chemistry 2018, 241, 182-187. DOI: 10.1016/j.foodchem.2017.08.072.

(424) Meng, Z.; Zhao, J.; Duan, H.; Guan, Y.; Zhao, L. Green and efficient extraction of four bioactive flavonoids from Pollen Typhae by ultrasound-assisted deep eutectic solvents extraction. J Pharm Biomed Anal 2018, 161, 246-253. DOI: 10.1016/j.jpba.2018.08.048.

(425) Zhang, X. F.; Chen, J.; Yang, J. L.; Shi, Y. P. UPLC-MS/MS analysis for antioxidant components of Lycii Fructus based on spectrum-effect relationship. Talanta 2018, 180, 389-395. DOI: 10.1016/j.talanta.2017.12.078.

(426) Yuan, Q. X.; Zhao, L. Y. The Mulberry (Morus alba L.) Fruit-A Review of Characteristic Components and Health Benefits. Journal of Agricultural and Food Chemistry 2017, 65 (48), 10383-10394. DOI: 10.1021/acs.jafc.7b03614.

(427) Mocan, A.; Schafberg, M.; Crișan, G.; Rohn, S. Determination of lignans and phenolic components of Schisandra chinensis (Turcz.) Baill. using HPLC-ESI-ToF-MS and HPLC-online TEAC: Contribution of individual components to overall antioxidant activity and comparison with traditional antioxidant assays. Journal of Functional Foods 2016, 24, 579-594. DOI: 10.1016/j.jff.2016.05.007.

(428) Quispe, C.; Villalobos, M.; Bórquez, J.; Simirgiotis, M. Chemical Composition and Antioxidant Activity of Aloe vera from the Pica Oasis (Tarapacá, Chile) by UHPLC-Q/Orbitrap/MS/MS. Journal of Chemistry 2018, 2018, 1-12. DOI: 10.1155/2018/6123850.

(429) Divakaran, S. A.; Hema, P. S.; Nair, M. S.; Nair, C. K. K. Antioxidant capacity and radioprotective properties of the flavonoids galangin and kaempferide isolated from Alpinia galanga L. (Zingiberaceae) against radiation induced cellular DNA damage. International Journal of Radiation Research 2013, 11 (2), 81-89.

(430) Liu, P. F.; Deng, T. S.; Hou, X. L.; Wang, J. G. Antioxidant Properties of Isolated Isorhamnetin from the Sea Buckthorn Marc. Plant Foods for Human Nutrition 2009, 64 (2), 141-145.

(431) Yao, Y. S.; Lin, G. B.; Xie, Y.; Ma, P.; Li, G. W.; Meng, Q. C.; Wu, T. Preformulation studies of myricetin: a natural antioxidant flavonoid. Pharmazie 2014, 69 (1), 19-26.

(432) Yang, J. Y.; Lee, H. S. Evaluation of Antioxidant and Antibacterial Activities of Morin Isolated from Mulberry Fruits (Morus alba L.). Journal of the Korean Society for Applied Biological Chemistry 2012, 55 (4), 485-489.

(433) Hatano, T.; Takagi, M.; Ito, H.; Yoshida, T. Phenolic constituents of liquorice .7. A new chalcone with a potent radical scavenging activity and accompanying phenolics from liquorice. Chemical & Pharmaceutical Bulletin 1997, 45 (9), 1485-1492, Article.

(434) GuzmÃ n-GutiÃ©rrez, S. L.; Nieto-Camacho, A.; Castillo-Arellano, J. I.; Huerta-Salazar, E.; Hernã, n.-P. G.; Silva-Miranda, M.; Argã¼Ello-Nã, j. O.; Sepãºlveda-Robles, O.; Espitia, C. I.; Reyes-Chilpa, R. Mexican Propolis: A Source of Antioxidants and Anti-Inflammatory Compounds, and Isolation of a Novel Chalcone and Îµ-Caprolactone Derivative. Molecules 2018, 23 (2), 334.

(435) Bucar, F., .; Resch, M., .; Bauer, R., .; Burits, M., .; Knauder, E., .; Schubert-Zsilavecz, M., . 5-Methylflavasperone and rhamnetin from Guiera senegalensis and their antioxidative and 5-lipoxygenase inhibitory activity. Pharmazie 2010, 30 (13), no-no.

(436) Zhao, L.-j.; Liu, W.; Xiong, S.-h.; Tang, J.; Lou, Z.-h.; Xie, M.-x.; Xia, B.-h.; Lin, L.-m.; Liao, D.-f. Determination of Total Flavonoids Contents and Antioxidant Activity of Ginkgo biloba Leaf by Near-Infrared Reflectance Method. International Journal of Analytical Chemistry 2018, 2018, 1-7. DOI: 10.1155/2018/8195784.

(437) Li, X. C.; Wang, L.; Han, W. J.; Mai, W. Q.; Han, L.; Chen, D. F. Amentoflavone protects against hydroxyl radical-induced DNA damage via antioxidant mechanism. Turkish Journal of Biochemistry-Turk Biyokimya Dergisi 2014, 39 (1), 30-U58.

(438) Zheng, X.-k.; Wang, W.-w.; Zhang, L.; Su, C.-f.; Wu, Y.-y.; Ke, Y.-y.; Hou, Q.-w.; Liu, Z.-y.; Gao, A.-s.; Feng, W.-s. Antihyperlipidaemic and antioxidant effect of the total flavonoids inSelaginella tamariscina(Beauv.) Spring in diabetic mice. Journal of Pharmacy and Pharmacology 2013, 65 (5), 757-766. DOI: 10.1111/jphp.12035.

(439) Li, X.; Huang, Y.; Chen, D. Protective Effect against Hydroxyl-induced DNA Damage and Antioxidant Activity of Citri reticulatae Pericarpium. Adv Pharm Bull 2013, 3 (1), 175-181. DOI: 10.5681/apb.2013.029.

(440) Barreca, D.; Bisignano, C.; Ginestra, G.; Bisignano, G.; Bellocco, E.; Leuzzi, U.; Gattuso, G. Polymethoxylated, C- and O-glycosyl flavonoids in tangelo (Citrus reticulate Citrus paradisi) juice and their influence on antioxidant properties. Food Chem 2013, 141, 1481-1488.

(441) Huang, Y.; Zhou, L. S.; Yan, L.; Ren, J.; Zhou, D. X.; Li, S. S. Alpinetin inhibits lipopolysaccharide-induced acute kidney injury in mice. International Immunopharmacology 2015, 28 (2), 1003-1008.

(442) Miler, M.; Zivanovic, J.; Ajdzanovic, V.; Orescanin-Dusic, Z.; Milenkovic, D.; Konic-Ristic, A.; Blagojevic, D.; Milosevic, V.; Sosic-Jurjevic, B. Citrus flavanones naringenin and hesperetin improve antioxidant status and membrane lipid compositions in the liver of old-aged Wistar rats. Experimental Gerontology 2016, 84, 49-60.

(443) Liu, R. X.; Wang, Q.; Guo, H. Z.; Li, L.; Bi, K. S.; Guo, D. A. Simultaneous determination of 10 major flavonoids in Dalbergia odorifera by high performance liquid chromatography. Journal of Pharmaceutical and Biomedical Analysis 2005, 39 (3-4), 469-476.

(444) Johnson, J.; Maher, P.; Hanneken, A. The Flavonoid, Eriodictyol, Induces Long-term Protection in ARPE-19 Cells through Its Effects on Nrf2 Activation and Phase 2 Gene Expression. Investigative Ophthalmology & Visual Science 2009, 50 (5), 2398-2406.

(445) Martins, N.; Barros, L.; Dueñas, M.; Santos-Buelga, C.; Ferreira, I. C. F. R. Characterization of phenolic compounds and antioxidant properties of Glycyrrhiza glabra L. rhizomes and roots. RSC Advances 2015, 5 (34), 26991-26997. DOI: 10.1039/c5ra03963k.

(446) Aiello, F.; Armentano, B.; Polera, N.; Carullo, G.; Loizzo, M. R.; Bonesi, M.; Cappello, M. S.; Capobianco, L.; Tundis, R. From Vegetable Waste to New Agents for Potential Health Applications: Antioxidant Properties and Effects of Extracts, Fractions and Pinocembrin from Glycyrrhiza glabra L. Aerial Parts on Viability of Five Human Cancer Cell Lines. Journal of Agricultural and Food Chemistry 2017, 65 (36), 7944-7954.

(447) Zhang, H.; Yang, Y.-f.; Zhou, Z.-q. Phenolic and flavonoid contents of mandarin (Citrus reticulata Blanco) fruit tissues and their antioxidant capacity as evaluated by DPPH and ABTS methods. Journal of Integrative Agriculture 2018, 17 (1), 256-263. DOI: 10.1016/s2095-3119(17)61664-2.

(448) Patel, N. K.; Bhutani, K. K. Pinostrobin and Cajanus lactone isolated from Cajanus cajan (L.) leaves inhibits TNF-α and IL-1β production: in vitro and in vivo experimentation. Phytomedicine 2014, 21 (7), 946-953.

(449) Dongmei, W.; Liang, Z.; Dengwu, L.; Wenjun, P. Antioxidant activities of different extracts and homoisoflavanones isolated from the Polygonatum odoratum. Natural product research 2013, 27 (12), 1111-1114.

(450) Dieudonné, N.; Joseph Tanyi, M.; Zacharias Tanee, F.; Albert, K.; Jean-Claude, M.; M Carmen, R.; Giner, R. M.; Salvador, M. E.; José Luis, R. Anti-inflammatory activities of two flavanones, sigmoidin A and sigmoidin B, from Erythrina sigmoidea. Planta medica 2004, 70 (02), 104-107.

(451) Igal, E.; M Paula, I.; Casanova, M. B.; Celentano, A. M.; Cristina, P.; José Luis, C.; Diez, R. A.; Marta, D. Mitochondrial toxicity and antioxidant activity of a prenylated flavonoid isolated from Dalea elegans. Chemico-biological interactions 2008, 171 (3), 294-305.

(452) Lu, C.-l.; Zhu, W.; Wang, M.; Xu, X.-j.; Lu, C.-j. Antioxidant and Anti-Inflammatory Activities of Phenolic-Enriched Extracts ofSmilax glabra. Evidence-Based Complementary and Alternative Medicine 2014, 2014, 1-8. DOI: 10.1155/2014/910438.

(453) Valenzuela A; Guerra R; LA., V. Antioxidant properties of the flavonoids silybin and (+)-cyanidanol-3: comparison with butylated hydroxyanisole and butylated hydroxytoluene. Planta Med 1986, 6, 438-440.

(454) Lucini, L.; Kane, D.; Pellizzoni, M.; Ferrari, A.; Trevisi, E.; Ruzickova, G.; Arslan, D. Phenolic profile and in vitro antioxidant power of different milk thistle [Silybum marianum (L.) Gaertn.] cultivars. Industrial Crops and Products 2016, 83, 11-16. DOI: 10.1016/j.indcrop.2015.12.023.

(455) Ouyang, H.; Hou, K.; Peng, W.; Liu, Z.; Deng, H. Antioxidant and xanthine oxidase inhibitory activities of total polyphenols from onion. Saudi Journal of Biological Sciences 2018, 25 (7), 1509-1513. DOI: 10.1016/j.sjbs.2017.08.005.

(456) Chang, S. K.; Alasalvar, C.; Shahidi, F. Review of dried fruits: Phytochemicals, antioxidant efficacies, and health benefits. Journal of Functional Foods 2016, 21, 113-132. DOI: 10.1016/j.jff.2015.11.034.

(457) Lu, C. L.; Zhu, W.; Wang, M.; Xu, X. J.; Lu, C. J. Antioxidant and Anti-Inflammatory Activities of Phenolic-Enriched Extracts of Smilax glabra. Evidence-Based Complementary and Alternative Medicine 2014.

(458) Zhao, Z. W.; Dong, Z. F.; Ming, J.; Liu, Y. Cedrin identified from Cedrus deodara (Roxb.) G. Don protects PC12 cells against neurotoxicity induced by A beta(1-42). Natural Product Research 2018, 32 (12), 1455-1458.

(459) Shao, B.; Guo, H. Z.; Cui, Y. J.; Liu, A. H.; Yu, H. L.; Guo, H.; Xu, M.; Guo, D. A. Simultaneous determination of six major stilbenes and flavonoids in Smilax china by high performance liquid chromatography. Journal of Pharmaceutical and Biomedical Analysis 2007, 44 (3), 737-742.

(460) Chen, S. D.; Lu, C. J.; Zhao, R. Z. Qualitative and Quantitative Analysis of Rhizoma Smilacis glabrae by Ultra High Performance Liquid Chromatography Coupled with LTQ Orbitrap(XL) Hybrid Mass Spectrometry. Molecules 2014, 19 (7), 10427-10439.

(461) Kwon, D.; Yoon, S.; Carter, O.; Bailey, G. S.; Dashwood, R. H. Antioxidant and antigenotoxic activities ofAngelica keiskei, Oenanthe javanicaandBrassica oleraceain the Salmonella mutagenicity assay and in HCT116 human colon cancer cells. BioFactors 2006, 26 (4), 231-244. DOI: 10.1002/biof.5520260402.

(462) Choi, E. J.; Kwon, H. C.; Sohn, Y. C.; Nam, C. W.; Park, H. B.; Kim, C. Y.; Yang, H. O. Four flavonoids from Echinosophora koreensis and their effects on alcohol metabolizing enzymes. Arch Pharm Res 2009, 32 (6), 851-855. DOI: 10.1007/s12272-009-1606-2.

(463) Qian, Z. M.; Lu, J.; Gao, Q. P.; Li, S. P. Rapid method for simultaneous determination of flavonoid, saponins and polyacetylenes in folium ginseng and radix ginseng by pressurized liquid extraction and high-performance liquid chromatography coupled with diode array detection and mass spectrometry. J Chromatogr A 2009, 1216 (18), 3825-3830. DOI: 10.1016/j.chroma.2009.02.065.

(464) He, F. Y.; Li, D. W.; Wang, D. M.; Deng, M. Extraction and Purification of Quercitrin, Hyperoside, Rutin, and Afzelin from Zanthoxylum Bungeanum Maxim Leaves Using an Aqueous Two-Phase System. J. Food Sci. 2016, 81 (7), C1593-C1602.

(465) Bezerra, I. C. F.; Ramos, R. T. D.; Ferreira, M. R. A.; Soares, L. A. L. Chromatographic profiles of extractives from leaves of Eugenia uniflora. Revista Brasileira De Farmacognosia-Brazilian Journal of Pharmacognosy 2018, 28 (1), 92-101.

(466) Cichewicz, R. H.; Nair, M. G. Isolation and Characterization of Stelladerol, a New Antioxidant Naphthalene Glycoside, and Other Antioxidant Glycosides from Edible Daylily (Hemerocallis) Flowers. Journal of Agricultural and Food Chemistry 2002, 50 (1), 87-91. DOI: 10.1021/jf010914k.

(467) Granica, S.; Czerwińska, M. E.; Żyżyńska-Granica, B.; Kiss, A. K. Antioxidant and anti-inflammatory flavonol glucuronides from Polygonum aviculare L. Fitoterapia 2013, 91, 180-188. DOI: 10.1016/j.fitote.2013.08.026.

(468) Qin, Y.; Chen, J.-P.; Li, C.-Y.; Zhu, L.-J.; Zhang, X.; Wang, J.-H.; Yao, X.-S. Flavonoid glycosides from the fruits of Embelia ribes and their anti-oxidant and α-glucosidase inhibitory activities. Journal of Asian Natural Products Research 2020, 23 (8), 724-730. DOI: 10.1080/10286020.2020.1776266.

(469) Ferreres, F.; Pereira, D. M.; Valentão, P. c.; Andrade, P. B.; Seabra, R. M.; Sottomayor, M. New Phenolic Compounds and Antioxidant Potential ofCatharanthus roseus. Journal of Agricultural and Food Chemistry 2008, 56 (21), 9967-9974. DOI: 10.1021/jf8022723.

(470) Nishida, Y.; Wada, K.; Toyohisa, D.; Tanaka, T.; Ono, M.; Yasuda, S. Homoisoflavones as the antioxidants responsible from bulbs of Scilla scilloides. Nat Prod Res 2013, 27 (24), 2360-2362. DOI: 10.1080/14786419.2013.830218.

(471) Tepe, B. In vitro antioxidant activities of the methanol extracts of four Helichrysum species from Turkey. Food Chemistry 2005, 90 (4), 685-689. DOI: 10.1016/j.foodchem.2004.04.030.

(472) Chen, H.; Wang, C.; Ye, J.; Zhou, H.; Yuan, J. Phenolic extracts fromRhus verniciflua Stokes bark by decompressing inner ebullition and their antioxidant activities. Natural Product Research 2014, 28 (7), 496-499. DOI: 10.1080/14786419.2013.872105.

(473) Li, X. C.; Xie, H.; Zhan, R. C.; Chen, D. F. Effect of Double Bond Position on 2-Phenyl-benzofuran Antioxidants: A Comparative Study of Moracin C and Iso-Moracin C. Molecules 2018, 23 (4). DOI: ARTN 754
10.3390/molecules23040754.

(474) Chen, Y.; Wei, X. H.; Deng, H. Antioxidant 2-phenylbenzofurans and a coumestan from Lespedeza virgata. Journal of Natural Products 2008, 71 (6), 929.

(475) Li, M.; Zhang, L.; Liu, W.; Lu, W. DFT Study on Molecular Structures and ROS Scavenging Mechanisms of Novel Antioxidants from Lespedeza Virgata. Chinese Journal of Chemical Physics 2011, 24 (2), 173-180.

(476) Qi, J.; Deng, H.; Cao, D. Total synthesis of lespedezavirgatol. Science China Chemistry 2010, 53 (12), 2547-2550.

(477) Ju, H. L.; Ko, H. J.; Woo, E. R.; Sang, K. L.; Moon, B. S.; Chan, W. L.; Mandava, S.; Samala, M.; Lee, J.; Kim, H. P. Moracin M inhibits airway inflammation by interrupting the JNK/c-Jun and NF-κB pathways in vitro and in vivo. European Journal of Pharmacology 2016, 783, 64-72.

(478) Sun, J.; He, X. M.; Zhao, M. M.; Li, L.; Li, C. B.; Dong, Y. Antioxidant and nitrite-scavenging capacities of phenolic compounds from sugarcane (Saccharum officinarum L.) tops. Molecules 2014, 19 (9), 13147-13160.

(479) Kapche, G. D. W. F.; Fozing, C. D.; Donfack, J. H.; Fotso, G. W.; Amadou, D.; Tchana, A. N.; Bezabih, M.; Moundipa, P. F.; Ngadjui, B. T.; Abegaz, B. M. Prenylated arylbenzofuran derivatives from Morus mesozygia with antioxidant activity. Phytochemistry 2009, 70 (2), 216-221.

(480) Seong, S. H.; Ha, M. T.; Min, B. S.; Jung, H. A.; Choi, J. S. Moracin derivatives from Morus Radix as dual BACE1 and cholinesterase inhibitors with antioxidant and anti-glycation capacities. Life Sciences 2018.

(481) Hwang, S. M.; Lee, H. J.; Jung, J. H.; Sim, D. Y.; Hwang, J.; Park, J. E.; Shim, B. S.; Kim, S. H. Inhibition of Wnt3a/FOXM1/beta-Catenin Axis and Activation of GSK3beta and Caspases are Critically Involved in Apoptotic Effect of Moracin D in Breast Cancers. Int J Mol Sci 2018, 19 (9). DOI: 10.3390/ijms19092681 From NLM.

(482) Han, S. J.; Ryu, S. N.; Kang, S. S. A new 2-arylbenzofuran with antioxidant activity from the black colored rice (Oryza sativa L.) bran. Cheminform 2005, 36 (18), 1365.

(483) Kapche, G. D.; Amadou, D.; Waffo-Teguo, P.; Donfack, J. H.; Fozing, C. D.; Harakat, D.; Tchana, A. N.; Mérillon, J. M.; Moundipa, P. F.; Ngadjui, B. T. Hepatoprotective and antioxidant arylbenzofurans and flavonoids from the twigs of Morus mesozygia. Planta medica 2011, 77 (10), 1044-1047.

(484) Kyekyeku, J. O.; Kusari, S.; Adosraku, R. K.; Zühlke, S.; Spiteller, M. Prenylated 2-arylbenzofuran derivatives with potent antioxidant properties from Chlorophora regia (Moraceae). Fitoterapia 2016, 108 (1), 41-47.

(485) Meneses, M. A.; Caputo, G.; Scognamiglio, M.; Reverchon, E.; Adami, R. Antioxidant phenolic compounds recovery from Mangifera indica L. by-products by supercritical antisolvent extraction. Journal of Food Engineering 2015, 163, 45-53. DOI: 10.1016/j.jfoodeng.2015.04.025.

(486) Olennikov, D. N.; Chirikova, N. K.; Vennos, C. Chemical Composition, Antioxidant and Anticholinesterase Activities of Gentianella azurea from Russian Federation. Nat. Prod. Commun. 2017, 12 (1), 55-56.

(487) Singh, P. P.; Ambika; Chauhan, S. M. S. Activity-guided isolation of antioxidant xanthones from Swertia chirayita (Roxb.) H. Karsten (Gentianaceae). Natural product research 2012, 26 (18), 1682-1686. DOI: 10.1080/14786419.2011.592836.

(488) Urbain, A.; Marston, A.; Grilo, L. S.; Bravo, J.; Purev, O.; Purevsuren, B.; Batsuren, D.; Reist, M.; Carrupt, P. A.; Hostettmann, K. Xanthones from Gentianella amarella ssp. acuta with acetylcholinesterase and monoamine oxidase inhibitory activities. J Nat Prod 2008, 71 (5), 895-897. DOI: 10.1021/np070690l.

(489) Li, X.; Chen, B.; Zhao, X.; Chen, D. 2-Phenyl-4,4,5,5-tetramethylimidazoline-1-oxyl 3-oxide Radical (PTIO center dot) Trapping Activity and Mechanisms of 16 Phenolic Xanthones. Molecules 2018, 23 (7). DOI: 10.3390/molecules23071692.

(490) Matkowski, A.; Kus, P.; Goralska, E.; Wozniak, D. Mangiferin - a Bioactive Xanthonoid, not only from Mango and not just Antioxidant. Mini-Reviews in Medicinal Chemistry 2013, 13 (3), 439-455, Review.

(491) Li, X.; Jiang, Q.; Chen, B.; Luo, X.; Chen, D. Structure-Activity Relationship and Prediction of the Electron-Transfer Potential of the Xanthones Series. Chemistryopen 2018, 7 (9), 730-736. DOI: 10.1002/open.201800108.

(492) Zheng, H.-H.; Luo, C.-T.; Chen, H.; Lin, J.-N.; Ye, C.-L.; Mao, S.-S.; Li, Y.-L. Xanthones from Swertia mussotii as Multitarget-Directed Antidiabetic Agents. Chemmedchem 2014, 9 (7), 1374-1377. DOI: 10.1002/cmdc.201300507.

(493) Xia, Y.; Liu, X.; Zou, C.; Feng, S.; Guo, H.; Yang, Y.; Lei, Y.; Zhang, J.; Lu, Y. Garcinone C exerts antitumor activity by modulating the expression of ATR/Stat3/4E-BP1 in nasopharyngeal carcinoma cells. Oncology reports 2018, 39 (3), 1485-1493. DOI: 10.3892/or.2018.6218.

(494) Wang, S. N.; Li, Q.; Jing, M. H.; Alba, E.; Yang, X. H.; Sabate, R.; Han, Y. F.; Pi, R. B.; Lan, W. J.; Yang, X. B.; et al. Natural Xanthones from Garcinia mangostana with Multifunctional Activities for the Therapy of Alzheimer's Disease. Neurochem. Res. 2016, 41 (7), 1806-1817, Article. DOI: 10.1007/s11064-016-1896-y.

(495) Gu, C.; Yang, M.; Zhou, Z.; Khan, A.; Cao, J.; Cheng, G. Purification and characterization of four benzophenone derivatives from Mangifera indica L. leaves and their antioxidant, immunosuppressive and alpha-glucosidase inhibitory activities. Journal of Functional Foods 2019, 52, 709-714. DOI: 10.1016/j.jff.2018.11.045.
(496) Hou, C. Theoretical study of antioxidative ability and antioxidative mechanism of norathyriol in solution. Computational and Theoretical Chemistry 2014, 1028, 87-91. DOI: 10.1016/j.comptc.2013.11.023.
(497) Gao, X.-y.; Wang, S.-n.; Yang, X.-h.; Lan, W.-j.; Chen, Z.-w.; Chen, J.-k.; Xie, J.-h.; Han, Y.-f.; Pi, R.-b.; Yang, X.-b. Gartanin Protects Neurons against Glutamate-Induced Cell Death in HT22 Cells: Independence of Nrf-2 but Involvement of HO-1 and AMPK. Neurochemical Research 2016, 41 (9), 2267-2277. DOI: 10.1007/s11064-016-1941-x.
(498) Luo, M.; Liu, Q.; He, M.; Yu, Z.; Pi, R.; Li, M.; Yang, X.; Wang, S.; Liu, A. Gartanin induces cell cycle arrest and autophagy and suppresses migration involving PI3K/Akt/mTOR and MAPK signalling pathway in human glioma cells. Journal of Cellular and Molecular Medicine 2017, 21 (1), 46-57. DOI: 10.1111/jcmm.12937.
(499) Kim, M.-O.; Lee, H.-S.; Chin, Y.-W.; Moon, D.-O.; Ahn, J.-S. Gartanin induces autophagy through JNK activation which extenuates caspase-dependent apoptosis. Oncology Reports 2015, 34 (1), 139-146. DOI: 10.3892/or.2015.3948.
(500) Wang, S.-n.; Li, Q.; Jing, M.-h.; Alba, E.; Yang, X.-h.; Sabate, R.; Han, Y.-f.; Pi, R.-b.; Lan, W.-j.; Yang, X.-b.; et al. Natural Xanthones from Garcinia mangostana with Multifunctional Activities for the Therapy of Alzheimer's Disease. Neurochemical Research 2016, 41 (7), 1806-1817. DOI: 10.1007/s11064-016-1896-y.
(501) Blanco-Ayala, T.; Lugo-Huitron, R.; Serrano-Lopez, E. M.; Reyes-Chilpa, R.; Rangel-Lopez, E.; Pineda, B.; Noel Medina-Campos, O.; Sanchez-Chapul, L.; Pinzon, E.; Cristina, T.-S.; et al. Antioxidant properties of xanthones from Calophyllum brasiliense: prevention of oxidative damage induced by FeSO4. Bmc Complementary and Alternative Medicine 2013, 13. DOI: 10.1186/1472-6882-13-262.
(502) Chen, L.; Zhou, Q.; Li, B.; Liu, S.-J.; Dong, J.-X. A new flavonoid from Cudrania cochinchinensis. Natural product research 2015, 29 (13), 1217-1221. DOI: 10.1080/14786419.2014.997234.
(503) Li, W.; Sun, Y. N.; Yan, X. T.; Yang, S. Y.; Choi, C. W.; Hyun, J. W.; Kang, H. K.; Paek, K. Y.; Kim, Y. H. Isolation of xanthones from adventitious roots of St. John's Wort (Hypericum perforatum L.) and their antioxidant and cytotoxic activities. Food Science and Biotechnology 2013, 22 (4), 945-949. DOI: 10.1007/s10068-013-0168-8.
(504) Minami, H.; Kinoshita, M.; Fukuyama, Y.; Kodama, M.; Yoshizawa, T.; Sugiura, M.; Nakagawa, K.; Tago, H. ANTIOXIDANT XANTHONES FROM GARCINIA-SUBELLIPTICA. Phytochemistry 1994, 36 (2), 501-506. DOI: 10.1016/s0031-9422(00)97103-6.
(505) Wang, L.-L.; Li, Z.-L.; Hua, H.-M.; Liu, X.-Q. Chemical constituents from barks of Garcinia tetralata. Zhongguo Zhong yao za zhi = Zhongguo zhongyao zazhi = China journal of Chinese materia medica 2008, 33 (20), 2350-2352.
(506) Iinuma, M.; Tosa, H.; Tanaka, T.; Asai, F.; Shimano, R. 3 XANTHONES FROM ROOT BARK OF GARCINIA-SUBELLIPTICA. Phytochemistry 1995, 38 (1), 247-249, Article. DOI: 10.1016/0031-9422(94)00595-k.
(507) Chukaew, A.; Saithong, S.; Chusri, S.; Limsuwan, S.; Watanapokasin, R.; Voravuthikunchai, S. P.; Chakthong, S. Cytotoxic xanthones from the roots of Mesua ferrea L. Phytochemistry 2019, 157, 64-70. DOI: 10.1016/j.phytochem.2018.10.008.
(508) Zuo, G.-Y.; An, J.; Han, J.; Zhang, Y.-L.; Wang, G.-C.; Hao, X.-Y.; Bian, Z.-Q. Isojacareubin from the Chinese Herb Hypericum japonicum: Potent Antibacterial and Synergistic Effects on Clinical Methicillin-Resistant Staphylococcus aureus (MRSA). International Journal of Molecular Sciences 2012, 13 (7), 8210-8218. DOI: 10.3390/ijms13078210.

(509) Lee, E. J. fun609α, g-Mangostins Induce Autophagy and Show Synergistic Effect with Gemcitabine in Pancreatic Cancer Cell Lines. Biomolecules & Therapeutics 2017, 25 (6), 609-617.

(510) Stark, T. D.; Salger, M.; Frank, O.; Balemba, O. B.; Wakamatsu, J.; Hofmann, T. Antioxidative Compounds from Garcinia buchananii Stem Bark. Journal of Natural Products 2015, 78 (2), 234-240. DOI: 10.1021/np5007873.

(511) Yuan, H.; Jiang, C.; Zhao, J.; Zhao, Y.; Zhang, Y.; Xu, Y.; Gao, X.; Guo, L.; Liu, Y.; Liu, K.; et al. Euxanthone Attenuates A(1-42)-Induced Oxidative Stress and Apoptosis by Triggering Autophagy. Journal of Molecular Neuroscience 2018, 66 (4), 512-523. DOI: 10.1007/s12031-018-1175-2.

(512) Li, Y.-z.; Li, Z.-l.; Hua, H.-m.; Li, Z.-g.; Liu, M.-s. Studies on flavonoids from stems and leaves of Calophyllum inophyllum. Zhongguo Zhong yao za zhi = Zhongguo zhongyao zazhi = China journal of Chinese materia medica 2007, 32 (8), 692-694.

(513) Lannang, A. M.; Komguem, J.; Ngninzeko, F. N.; Tangmouo, J. G.; Lontsi, D.; Ajaz, A.; Choudhary, M. I.; Ranjit, R.; Devkota, K. P.; Sondengam, B. L. Bangangxanthone A and B, two xanthones from the stem bark of Garcinia polyantha Oliv. Phytochemistry 2005, 66 (19), 2351-2355. DOI: 10.1016/j.phytochem.2005.06.016.

(514) Buelna-Chontal, M.; Correa, F.; Hernandez-Resendiz, S.; Zazueta, C.; Pedraza-Chaverri, J. Protective Effect of alpha-Mangostin on Cardiac Reperfusion Damage by Attenuation of Oxidative Stress. Journal of medicinal food 2011, 14 (11), 1370-1374. DOI: 10.1089/jmf.2010.0238.

(515) Trisuwan, K.; Ritthiwigrom, T. Benzophenone and xanthone derivatives from the inflorescences of Garcinia cowa. Archives of Pharmacal Research 2012, 35 (10), 1733-1738. DOI: 10.1007/s12272-012-1004-z.

(516) Panthong, K.; Hutadilok-Towatana, N.; Panthong, A. Cowaxanthone F, a new tetraoxygenated xanthone, and other anti-inflammatory and antioxidant compounds from Garcinia cowa. Canadian Journal of Chemistry-Revue Canadienne De Chimie 2009, 87 (11), 1636-1640. DOI: 10.1139/v09-123.

(517) Panthong, K.; Pongcharoen, W.; Phongpaichit, S.; Taylor, W. C. Tetraoxygenated xanthones from the fruits of Garcinia cowa. Phytochemistry 2006, 67 (10), 999-1004. DOI: 10.1016/j.phytochem.2006.02.027.

(518) Ponguschariyagul, S.; Sichaem, J.; Khumkratok, S.; Siripong, P.; Lugsanangarm, K.; Tip-pyang, S. Caloinophyllin A, a new chromanone derivative from Calophyllum inophyllum roots. Natural product research 2018, 32 (21), 2535-2541. DOI: 10.1080/14786419.2018.1425845.

(519) Jung, H. A.; Su, B. N.; Keller, W. J.; Mehta, R. G.; Kinghorn, A. D. Antioxidant xanthones from the pericarp of Garcinia mangostana (mangosteen). Journal of Agricultural and Food Chemistry 2006, 54 (6), 2077-2082. DOI: 10.1021/jf052649z.

(520) Li, J.; Chen, L.; Wu, H.; Lu, Y.; Hu, Z.; Lu, B.; Zhang, L.; Chai, Y.; Zhang, J. The Mixture of Salvianolic Acids from Salvia miltiorrhiza and Total Flavonoids from Anemarrhena asphodeloides Attenuate Sulfur Mustard-Induced Injury. International Journal of Molecular Sciences 2015, 16 (10), 24555-24573. DOI: 10.3390/ijms161024555.

(521) Ji, X.; Avula, B.; Khan, I. A. Quantitative and qualitative determination of six xanthones in Garcinia mangostana L. by LC-PDA and LC-ESI-MS. Journal of Pharmaceutical and Biomedical Analysis 2007, 43 (4), 1270-1276. DOI: 10.1016/j.jpba.2006.10.018.

(522) Suvarnakuta, P.; Chaweerungrat, C.; Devahastin, S. Effects of drying methods on assay and antioxidant activity of xanthones in mangosteen rind. Food Chemistry 2011, 125 (1), 240-247. DOI: 10.1016/j.foodchem.2010.09.015.

(523) Li, J.; Li, W. Z. M.; Huang, W.; Cheung, A. W. H.; Bi, C. W. C.; Duan, R.; Guo, A. J. Y.; Dong, T. T. X.; Tsim, K. W. K. Quality evaluation of Rhizoma Belamcandae (Belamcanda chinensis (L.) DC.) by using high-performance liquid chromatography coupled with diode array detector and mass spectrometry. Journal of Chromatography A 2009, 1216 (11), 2071-2078. DOI: 10.1016/j.chroma.2008.05.082.

(524) Yu, H. Y.; Jin, S. L.; Zhang, X.; Liu, Y.; Ou, Y. F.; Wang, N. L.; Yao, X. S. Two new benzophenone glucosides from Cratoxylon cochinchinensis. Chinese Chemical Letters 2009, 20 (4), 459-461. DOI: 10.1016/j.cclet.2008.12.038.

(525) Taher, M.; Susanti, D.; Rezali, M. F.; Zohri, F. S. A.; Ichwan, S. J. A.; Alkhamaiseh, S. I.; Ahmad, F. Apoptosis, antimicrobial and antioxidant activities phytochemicals from Garcinia malaccensis Hk.f. Asian Pacific Journal of Tropical Medicine 2012, 5 (2), 136-141. DOI: 10.1016/s1995-7645(12)60012-1.

(526) Chang, C. H.; Lin, C. C.; Hattori, M.; Namba, T. EFFECTS ON ANTI-LIPID PEROXIDATION OF CUDRANIA-COCHINCHINENSIS VAR GERONTOGEA. Journal of Ethnopharmacology 1994, 44 (2), 79-85.

(527) Zhou, Y.-H.; Zhang, S.-Y.; Guo, Q.; Chai, X.-Y.; Jiang, Y.; Tu, P.-F. Chemical investigation of the roots of Polygala sibirica L. Chinese journal of natural medicines 2014, 12 (3), 225-228. DOI: 10.3724/sp.j.1009.2014.00225.

(528) Wang, J.; Jiang, Z.; Wei, G.; Wang, Y.; Liu, Y. Protective Effects of Chemical Constituents from the Roots of Polygala sibirica L. on PC12 Cells Damage Induced by H2O2. Latin American Journal of Pharmacy 2014, 33 (7), 1120-1124.

(529) Yin, C.; Xie, L.; Guo, Y. Phytochemical analysis and antibacterial activity of Gentiana macrophylla extract against bacteria isolated from burn wound infections. Microbial Pathogenesis 2018, 114, 25-28. DOI: 10.1016/j.micpath.2017.10.049.

(530) Wani, B. A.; Ramamoorthy, D.; Rather, M. A.; Arumugam, N.; Qazi, A. K.; Majeed, R.; Hamid, A.; Ganie, S. A.; Ganai, B. A.; Anand, R.; et al. Induction of apoptosis in human pancreatic MiaPaCa-2 cells through the loss of mitochondrial membrane potential (Delta psi(m)) by Gentiana kurroo root extract and LC-ESI-MS analysis of its principal constituents. Phytomedicine 2013, 20 (8-9), 723-733. DOI: 10.1016/j.phymed.2013.01.011.

(531) Lee, B. W.; Lee, J. H.; Lee, S. T.; Lee, H. S.; Lee, W. S.; Jeong, T. S.; Park, K. H. Antioxidant and cytotoxic activities of xanthones from Cudrania tricuspidata. Bioorganic & Medicinal Chemistry Letters 2005, 15 (24), 5548-5552. DOI: 10.1016/j.bmcl.2005.08.099.

(532) Lin, L.-L.; Huang, F.; Chen, S.-B.; Yang, D.-J.; Chen, S.-L.; Yang, J.-S.; Xiao, P.-G. Chemical constituents in roots of Polygala fallax and their anti-oxidation activities in vitro. Zhongguo Zhong yao za zhi = Zhongguo zhongyao zazhi = China journal of Chinese materia medica 2005, 30 (11), 827-830.

(533) Wu, J.-f.; Tu, P.-f.; Zhan, H.-t.; Gao, J.-c. Dioxyxanthones from Polygala hongkongensis and Their Cytotoxicity. Chemical Research in Chinese Universities 2011, 27 (5), 777-779.

(534) Ashida, S.; Noguchi, S. F.; Suzuki, T. ANTIOXIDATIVE COMPONENTS, XANTHONE DERIVATIVES, IN SWERTIA-JAPONICA MAKINO. Journal of the American Oil Chemists Society 1994, 71 (10), 1095-1099. DOI: 10.1007/bf02675902.

(535) Tian, L. Y.; Bai, X.; Chen, X. H.; Fang, J. B.; Liu, S. H.; Chen, J. C. Anti-diabetic effect of methylswertianin and bellidifolin from Swertia punicea Hemsl. and its potential mechanism. Phytomedicine 2010, 17 (7), 533-539. DOI: 10.1016/j.phymed.2009.10.007.

(536) Mahendran, G.; Bai, V. N. Plant regeneration through direct somatic embryogenesis, antioxidant properties, and metabolite profiles of Swertia corymbosa (Griseb.) Wight ex CB Clarke. Plant Biosystems 2017, 151 (1), 39-49. DOI: 10.1080/11263504.2015.1064043.

(537) Li, H.; Lu, Q.; Bian, K. Ultrasonic-Assisted Extraction and Evaluation of Biological Activities of Flavonoids from Flemingia philippinensis Merr et Rolfe. Tropical Journal of Pharmaceutical Research 2015, 14 (8), 1365-U1213. DOI: 10.4314/tjpr.v14i8.7.

(538) Shi, Q.; Chen, J.; Zhou, Q.; Lei, H.; Luan, L.; Liu, X.; Wu, Y. Indirect identification of antioxidants in Polygalae Radix through their reaction with 2,2-dipheny1-1-picrylhydrazyl and subsequent HPLC-ESI-Q-TOF-MS/MS. Talanta 2015, 144, 830-835. DOI: 10.1016/j.talanta.2015.07.032.

(539) Xu, R.; Mao, F.; Zhao, Y.; Wang, W.; Fan, L.; Gao, X.; Zhao, J.; Tian, H. UPLC Quantitative Analysis of Multi-Components by Single Marker and Quality Evaluation of Polygala tenuifolia Wild. Extracts. Molecules 2017, 22 (12). DOI: 10.3390/molecules22122276.
(540) Azad, F. R.; Shafaghat, A.; Salimi, F. Chemical Constituents, Antioxidant and Antimicrobial Activities of Essential Oil and Hexane Extract from Arnebia linearifolia DC. Seeds. Journal of Essential Oil Bearing Plants 2014, 17 (1), 87-94. DOI: 10.1080/0972060x.2014.884813.
(541) Mohamed, T. K. Chemical constituents and antioxidant activity of Citrus paradisi (star-ruby red grapefruit) and Citrus sinensis (blood sweet orange) Egyptian cultivars. Asian Journal of Chemistry 2004, 16 (3-4), 1753-1764.
(542) Marques, E. d. S.; Salles, D. B.; Maistro, E. L. Assessment of the genotoxic/clastogenic potential of coumarin derivative 6,7-dihydroxycoumarin (aesculetin) in multiple mouse organs. Toxicology reports 2015, 2, 268-274. DOI: 10.1016/j.toxrep.2015.01.005.
(543) Wang, K.; Zhang, Y.; Ekunwe, S. I. N.; Yi, X.; Liu, X.; Wang, H.; Pan, Y. Antioxidant activity and inhibition effect on the growth of human colon carcinoma (HT-29) cells of esculetin from Cortex Fraxini. Medicinal Chemistry Research 2011, 20 (7), 968-974. DOI: 10.1007/s00044-010-9426-y.
(544) Tang, Y. P.; Hu, J.; Wang, J. H.; Lou, F. C. A new coumaronochromone from Sophora japonica. Journal of Asian Natural Products Research 2002, 4 (1), 1-5. DOI: 10.1080/10286020290019622.
(545) Wadhwa, D.; Arora, V.; Arora, L.; Arora, P.; Parkash, O. Synthesis of Naphthalene Functionalized Trans-2,3-dihydrofuro 3,2-c coumarins as Antioxidant and Anthelmintic Agents. Journal of Heterocyclic Chemistry 2016, 53 (4), 1030-1035. DOI: 10.1002/jhet.2431.
(546) Dehghan, H.; Sarrafi, Y.; Salehi, P.; Ebrahimi, S. N. alpha-Glucosidase inhibitory and antioxidant activity of furanocoumarins from Heracleum persicum. Medicinal Chemistry Research 2017, 26 (4), 849-855. DOI: 10.1007/s00044-017-1796-y.
(547) Li, D.; Wu, L. Coumarins from the roots of Angelica dahurica cause anti-allergic inflammation. Experimental and Therapeutic Medicine 2017, 14 (1), 874-880. DOI: 10.3892/etm.2017.4569.
(548) Kumar, V.; Sharma, K.; Ahmed, B.; Al-Abbasi, F. A.; Anwar, F.; Verma, A. Deconvoluting the dual hypoglycemic effect of wedelolactone isolated from Wedelia calendulacea: investigation via experimental validation and molecular docking. Rsc Advances 2018, 8 (32), 18180-18196. DOI: 10.1039/c7ra12568b.
(549) Nie, Y.; Yao, W. A Comprehensive Quality Evaluation Method Based on C-30-HPLC and an Analytic Hierarchy Process for the Chinese Herbal Formula, Erzhiwan. Molecules 2018, 23 (8). DOI: 10.3390/molecules23082045.
(550) Ding, H.; Wang, Y.; Gao, Y.; Han, X.; Liu, S.; Tang, G.; Li, J.; Zhao, D. Purification of wedelolactone from Eclipta alba and evaluation of antioxidant activity. Separation Science and Technology 2017, 52 (17), 2732-2741. DOI: 10.1080/01496395.2017.1374973.
(551) Han, L.; Sun, C.; Lin, J.; Li, F.; Li, X.; Wu, Z.; Liu, C.; Bai, Y.; Mai, W.; Chen, D. Herba Ecliptae Protects against Hydroxyl Radical-induced Damages to DNA and Mesenchymal Stem Cells via Antioxidant Mechanism. Journal of the Chinese Chemical Society 2014, 61 (10), 1161-1167. DOI: 10.1002/jccs.201400082.
(552) Kadhum, A. A. H.; Al-Amiery, A. A.; Musa, A. Y.; Mohamad, A. B. The Antioxidant Activity of New Coumarin Derivatives. International Journal of Molecular Sciences 2011, 12 (9), 5747-5761. DOI: 10.3390/ijms12095747.
(553) Oliva, A.; Meepagala, K. M.; Wedge, D. E.; Harries, D.; Hale, A. L.; Aliotta, G.; Duke, S. O. Natural fungicides from Ruta graveolens L. leaves, including a new quinolone alkaloid. Journal of Agricultural and Food Chemistry 2003, 51 (4), 890-896. DOI: 10.1021/jf0259361.
(554) Hisamoto, M.; Kikuzaki, H.; Ohigashi, H.; Nakatani, N. Antioxidant compounds from the leaves of Peucedanum japonicum Thunb. Journal of Agricultural and Food Chemistry 2003, 51 (18), 5255-5261. DOI: 10.1021/jf0262458.

(555) Yang, Y.-F.; Xu, W.; Song, W.; Ye, M.; Yang, X.-W. Transport of Twelve Coumarins from Angelicae Pubescentis Radix across a MDCK-pHaMDR Cell Monolayer-An in Vitro Model for Blood-Brain Barrier Permeability. Molecules 2015, 20 (7), 11719-11732. DOI: 10.3390/molecules200711719.

(556) Sourivong, P.; Schronerova, K.; Babincova, M. Scoparone inhibits ultraviolet radiation-induced lipid peroxidation. Zeitschrift Fur Naturforschung Section C-a Journal of Biosciences 2007, 62 (1-2), 61-64.

(557) Doppalapudi, S.; Mahira, S.; Khan, W. Development and in vitro assessment of psoralen and resveratrol co-loaded ultradeformable liposomes for the treatment of vitiligo. Journal of Photochemistry and Photobiology B-Biology 2017, 174, 44-57. DOI: 10.1016/j.jphotobiol.2017.07.007.

(558) Jan, S.; Parween, T.; Siddiqi, T. O.; Mahmooduzzafar. Anti-oxidant modulation in response to gamma radiation induced oxidative stress in developing seedlings of Psoralea corylifolia L. Journal of Environmental Radioactivity 2012, 113, 142-149. DOI: 10.1016/j.jenvrad.2012.05.019.

(559) Guo, J. M.; Weng, X. C.; Wu, H.; Li, Q. H.; Bi, K. S. Antioxidants from a Chinese medicinal herb - Psoralea corylifolia L. Food Chemistry 2005, 91 (2), 287-292. DOI: 10.1016/j.foodchem.2004.04.029.

(560) Chen, C.-H.; Hwang, T.-L.; Chen, L.-C.; Chang, T.-H.; Wei, C.-S.; Chen, J.-J. Isoflavones and anti-inflammatory constituents from the fruits of Psoralea corylifolia. Phytochemistry 2017, 143, 186-193. DOI: 10.1016/j.phytochem.2017.08.004.

(561) Orhan, I. E.; Tosun, F.; Skalicka-Wozniak, K. Cholinesterase and tyrosinase inhibitory, and antioxidant potential of randomly selected Umbelliferous plant species and the chromatographic profile of Heracleum platytaenium Boiss. and Angelica sylvestris L. var. sylvestris. Journal of the Serbian Chemical Society 2016, 81 (4), 357-368. DOI: 10.2298/jsc150902017o.

(562) Bai, Y.; Li, D.; Zhou, T.; Qin, N.; Li, Z.; Yu, Z.; Hua, H. Coumarins from the roots of Angelica dahurica with antioxidant and antiproliferative activities. Journal of Functional Foods 2016, 20, 453-462. DOI: 10.1016/j.jff.2015.11.018.

(563) Wang, Z.; Zhu, W.; Liu, H.; Wu, G.; Song, M.; Yang, B.; Yang, D.; Wang, Q.; Kuang, H. Simultaneous Determination of Aesculin, Aesculetin, Fraxetin, Fraxin and Polydatin in Beagle Dog Plasma by UPLC-ESI-MS/MS and Its Application in a Pharmacokinetic Study after Oral Administration Extracts of Ledum palustre L. Molecules 2018, 23 (9). DOI: 10.3390/molecules23092285.

(564) Yao, Y.; Zhao, X.; Xin, J.; Wu, Y.; Li, H. Coumarins improved type 2 diabetes induced by high-fat diet and streptozotocin in mice via antioxidation. Canadian Journal of Physiology and Pharmacology 2018, 96 (8), 765-771. DOI: 10.1139/cjpp-2017-0612.

(565) Naseri, M.; Monsef-Esfehani, H. R.; Saeidnia, S.; Dastan, D.; Gohari, A. R. Antioxidative Coumarins from the Roots of Ferulago subvelutina. Asian Journal of Chemistry 2013, 25 (4), 1875-1878. DOI: 10.14233/ajchem.2013.13208.

(566) Wu, C.-R.; Huang, M.-Y.; Lin, Y.-T.; Ju, H.-Y.; Ching, H. Antioxidant properties of Cortex Fraxini and its simple coumarins. Food Chemistry 2007, 104 (4), 1464-1471. DOI: 10.1016/j.foodchem.2007.02.023.

(567) Chen, Y.; Zhang, Q.; Liu, H.; Lu, C.; Liang, C.-L.; Qiu, F.; Han, L.; Dai, Z. Esculetin Ameliorates Psoriasis-Like Skin Disease in Mice by Inducing CD4(+) Foxp3(+) Regulatory T Cells. Frontiers in Immunology 2018, 9. DOI: 10.3389/fimmu.2018.02092.

(568) He, Y.; Li, C.; Ma, Q.; Chen, S. Esculetin inhibits oxidative stress and apoptosis in H9c2 cardiomyocytes following hypoxiaireoxygenation injury. Biochemical and Biophysical Research Communications 2018, 501 (1), 139-144. DOI: 10.1016/j.bbrc.2018.04.195.

(569) Kim, N. M.; Kim, J.; Chung, H. Y.; Choi, J. S. Isolation of luteolin 7-O-rutinoside and esculetin with potential antioxidant activity from the aerial parts of Artemisia montana. Archives of Pharmacal Research 2000, 23 (3), 237-239. DOI: 10.1007/bf02976451.

(570) Pokharel, Y. R.; Jeong, J.-E.; Oh, S. J.; Kim, S. K.; Woo, E.-R.; Kang, K. W. Screening of potential chemopreventive compounds from Poncirus trifoliata Raf. Pharmazie 2006, 61 (9), 796-798.

(571) Al-Rifai, A. Identification and evaluation of in-vitro antioxidant phenolic compounds from the Calendula tripterocarpa Rupr. South African Journal of Botany 2018, 116, 238-244. DOI: 10.1016/j.sajb.2018.04.007.

(572) Osman, W. N. W.; Mohamed, S. Standardized Morinda citrifolia L. and Morinda elliptica L. leaf extracts alleviated fatigue by improving glycogen storage and lipid/carbohydrate metabolism. Phytotherapy Research 2018, 32 (10), 2078-2085. DOI: 10.1002/ptr.6151.

(573) Shaw, C. Y.; Chen, C. H.; Hsu, C. C.; Chen, C. C.; Tsai, Y. C. Antioxidant properties of scopoletin isolated from Sinomonium acutum. Phytotherapy Research 2003, 17 (7), 823-825. DOI: 10.1002/ptr.1170.

(574) Dakah, A.; Zaid, S.; Suleiman, M.; Abbas, S.; Wink, M. In vitro propagation of the medicinal plant Ziziphora tenuior L. and evaluation of its antioxidant activity. Saudi Journal of Biological Sciences 2014, 21 (4), 317-323. DOI: 10.1016/j.sjbs.2013.12.002.

(575) Jung, H. A.; Park, J. J.; Islam, M. N.; Jin, S. E.; Min, B.-S.; Lee, J.-H.; Sohn, H. S.; Choi, J. S. Inhibitory Activity of Coumarins from Artemisia capillaris against Advanced Glycation Endproduct Formation. Archives of Pharmacal Research 2012, 35 (6), 1021-1035. DOI: 10.1007/s12272-012-0610-0.

(576) Kang, J. I.; Hong, J.-Y.; Choi, J. S.; Lee, S. K. Columbianadin Inhibits Cell Proliferation by Inducing Apoptosis and Necroptosis in HCT116 Colon Cancer Cells. Biomolecules & Therapeutics 2016, 24 (3), 320-327. DOI: 10.4062/biomolther.2015.145.

(577) Ng, T. B.; Liu, F.; Wang, Z. T. Antioxidative activity of natural products from plants. Life Sciences 2000, 66 (8), 709-723. DOI: 10.1016/s0024-3205(99)00642-6.

(578) Yang, Y.-F.; Zhang, L.; Yang, X.-W. Distribution Assessments of Coumarins from Angelicae Pubescentis Radix in Rat Cerebrospinal Fluid and Brain by Liquid Chromatography Tandem Mass Spectrometry Analysis. Molecules (Basel, Switzerland) 2018, 23 (1). DOI: 10.3390/molecules23010225.

(579) Girennavar, B.; Poulose, S. M.; Jayaprakasha, G. K.; Bhat, N. G.; Patil, B. S. Furocoumarins from grapefruit juice and their effect on human CYP 3A4 and CYP1B1 isoenzymes. Bioorganic & Medicinal Chemistry 2006, 14 (8), 2606-2612. DOI: 10.1016/j.bmc.2005.11.039.

(580) Miyake, Y.; Murakami, A.; Sugiyama, Y.; Isobe, M.; Koskimizu, K.; Ohigashi, H. Identification of coumarins from lemon fruit (Citrus limon) as inhibitors of in vitro tumor promotion and superoxide and nitric oxide generation. Journal of Agricultural and Food Chemistry 1999, 47 (8), 3151-3157. DOI: 10.1021/jf980999y.

(581) Okuyama, S.; Nakashima, T.; Nakamura, K.; Shinoka, W.; Kotani, M.; Sawamoto, A.; Nakajima, M.; Furukawa, Y. Inhibitory Effects of Auraptene and Naringin on Astroglial Activation, Tau Hyperphosphorylation, and Suppression of Neurogenesis in the Hippocampus of Streptozotocin-Induced Hyperglycemic Mice. Antioxidants 2018, 7 (8). DOI: 10.3390/antiox7080109.

(582) Soltani, F.; Mosaffa, F.; Iranshahi, M.; Karimi, G.; Malekaneh, M.; Haghighi, F.; Behravan, J. Auraptene from Ferula szowitsiana Protects Human Peripheral Lymphocytes Against Oxidative Stress. Phytotherapy Research 2010, 24 (1), 85-89. DOI: 10.1002/ptr.2874.

(583) Karakaya, S.; Koca, M.; Kilic, C. S.; Coskun, M. Antioxidant and anticholinesterase activities of Ferulago syriaca Boiss. and F-isaurica Pesmen growing in Turkey. Medicinal Chemistry Research 2018, 27 (7), 1843-1850. DOI: 10.1007/s00044-018-2196-7.

(584) Liang, W.-H.; Chang, T.-W.; Charng, Y.-C. Effects of drying methods on contents of bioactive compounds and antioxidant activities of Angelica dahurica. Food Science and Biotechnology 2018, 27 (4), 1085-1092. DOI: 10.1007/s10068-018-0359-4.

(585) Biljali, S.; Hadjimitova, V. A.; Topashka-Ancheva, M. N.; Momekova, D. B.; Traykov, T. T.; Karaivanova, M. H. Antioxidant and antiradical properties of esculin, and its effect in a model of epirubicin-induced bone marrow toxicity. Folia medica 2012, 54 (3), 42-49.

(586) Hu, H.-B.; Zheng, X.-D.; Jian, Y.-F.; Liu, J.-X.; Zhu, J.-H. Constituents of the root of Anemone tomentosa. Archives of Pharmacal Research 2011, 34 (7), 1097-1105. DOI: 10.1007/s12272-011-0707-x.

(587) Zhang, B.; Chen, J.; Jiang, Q.; Wang, X.; Lu, Y.; Gong, L.; Chen, D. Simultaneous determination of three active components in rat plasma by UPLC-MS/MS: Application to pharmacokinetic study after oral administration of Herba Sarcandrae extract. Biomedical Chromatography 2017, 31 (4). DOI: 10.1002/bmc.3834.

(588) Huang, H.-Y.; Ko, H.-H.; Jin, Y.-J.; Yang, S.-Z.; Shih, Y.-A.; Chen, I.-S. Dihydrochalcone glucosides and antioxidant activity from the roots of Anneslea fragrans var. lanceolata. Phytochemistry 2012, 78, 120-125. DOI: 10.1016/j.phytochem.2012.02.023.

(589) Deng, G.-g.; Gui, Z.-j.; Yang, X.-w. Chemical constituents from polarity part in roots of Angelica dahurica var. formosana cv. Chuanbaizhi. China journal of Chinese materia medica 2015, 40 (19), 3805-3810.

(590) Phan Thi Anh, D.; Tran Le, Q.; Nguyen Thi Thanh, M. Constituents of the Stem of Nauclea orientalis. Natural Product Communications 2015, 10 (11), 1901-1903.

(591) Doi, K.; Kojima, T.; Makino, M.; Kimura, Y.; Fujimoto, Y. Studies on the constituents of the leaves of Morus alba L. Chemical & Pharmaceutical Bulletin 2001, 49 (2), 151-153. DOI: 10.1248/cpb.49.151.

(592) Huawu, Z.; Yiming, L.; Xiang, H.; Yuanying, J.; Shanhao, J.; Dayuan, Z. J. A. J. O. S. M. M. U. Antiinflammatory and antioxidant effects of the extracts from Scrophularia ningpoensis Hemsl.[J]. 1999, 9.

(593) Kapoor, I. P. S.; Singh, B.; Singh, G.; De Heluani, C. S.; De Lampasona, M. P.; Catalan, C. A. N. Chemistry and antioxidant activity of essential oil and oleoresins of black caraway (Carum bulbocastanum) fruits: Part 69. Journal of the Science of Food and Agriculture 2010, 90 (3), 385-390. DOI: 10.1002/jsfa.3824.

(594) Singh, G.; Marimuthu, P.; De Heluani, C. S.; Catalan, C. A. N. Antioxidant and biocidal activities of Carum nigrum (seed) essential oil, oleoresin, and their selected components. Journal of Agricultural and Food Chemistry 2006, 54 (1), 174-181. DOI: 10.1021/jf0518610.

(595) Kikuzaki, H.; Hara, S.; Kawai, Y.; Nakatani, N. Antioxidative phenylpropanoids from berries of Pimenta dioica. Phytochemistry 1999, 52 (7), 1307-1312. DOI: 10.1016/s0031-9422(99)00406-9.

(596) Leu, Y. L.; Wang, Y. L.; Huang, S. C.; Shi, L. S. Chemical constituents from roots of Taraxacum formosanum. Chemical & pharmaceutical bulletin 2005, 53 (7), 853-855. DOI: 10.1248/cpb.53.853.

(597) Genovese, S.; Curini, M.; Epifano, F.; Menghini, L.; Ricci, D.; Fraternale, D.; Giamperi, L.; Bucchini, A.; Bellacchio, E. Antioxidant and lipoxygenase inhibitory activities of boropinic acid, active principle of Boronia pinnata. Planta medica 2006, 72 (11), 987-987.

(598) Delazar, A.; Biglari, F.; Esnaashari, S.; Nazemiyeh, H.; Talebpour, A.-H.; Nahar, L.; Sarker, S. D. GC-MS analysis of the essential oils, and the isolation of phenylpropanoid derivatives from the aerial parts of Pimpinella aurea. Phytochemistry 2006, 67 (19), 2176-2181. DOI: 10.1016/j.phytochem.2006.06.012.

(599) Tai, Z.; Chen, A.; Qin, B.; Cai, L.; Xu, Y. Chemical constituents and antioxidant activity of the Musa basjoo flower. European Food Research and Technology 2014, 239 (3), 501-508. DOI: 10.1007/s00217-014-2244-6.

(600) Zhou, W.; Xie, H.; Xu, X.; Liang, Y.; Wei, X. Phenolic constituents from Isodon lophanthoides var. graciliflorus and their antioxidant and antibacterial activities. Journal of Functional Foods 2014, 6, 492-498. DOI: 10.1016/j.jff.2013.11.015.

(601) Al-Qudah, M. A.; Al-Jaber, H. I.; Abu Zarga, M. H.; Abu Orabi, S. T. Flavonoid and phenolic compounds from Salvia palaestina L. growing wild in Jordan and their antioxidant activities. Phytochemistry 2014, 99, 115-120. DOI: 10.1016/j.phytochem.2014.01.001.

(602) Yousuf, S.; Choudhary, M. I.; Rahman, A. U. Separation of phenylpropanoids and evaluation of their antioxidant activity. In Advanced Protocols in Oxidative Stress II, Springer, 2010; pp 357-377.

(603) Nićiforović, N.; Mihailović, V.; Mašković, P.; Solujić, S.; Stojković, A.; Muratspahić, D. P. J. F.; Toxicology, C. Antioxidant activity of selected plant species; potential new sources of natural antioxidants. 2010, 48 (11), 3125-3130.

(604) Shin, S.-L.; Lee, C.-H. J. J. o. L. S. Antioxidant activities of ostrich fern by different extraction methods and solvents. 2011, 21 (1), 56-61.

(605) Daels-Rakotoarison, D. A.; Seidel, V.; Gressier, B.; Brunet, C.; Tillequin, F.; Bailleul, F.; Luyckx, M.; Dine, T.; Cazin, M.; Cazin, J.-C. J. A. Neurosedative and antioxidant activities of phenylpropanoids from Ballota nigra. 2000, 50 (01), 16-23.

(606) Hennebelle, T.; Sahpaz, S.; Gressier, B.; Joseph, H.; Bailleul, F. J. P. R. A. I. J. D. t. P.; Derivatives, T. E. o. N. P. Antioxidant and neurosedative properties of polyphenols and iridoids from Lippia alba. 2008, 22 (2), 256-258.

(607) da Silva, F. R.; Rodrigues, F. E.; Gomes, A. R.; Arriaga, A.; Mafezoli, J.; Lemos, T. L.; Almeida, M.; Santiago, G. M.; Braz-Filho, R.; da Costa, J. G. J. Q. N. Phytochemical study, antioxidant and antibacterial activities of Stemodia maritima. 2014, 37 (9), 1474-1478.

(608) Saracoglu, I.; Harput, U. S.; Inoue, M.; Ogihara, Y. New phenylethanoid glycosides from Veronica pectinata var. glandulosa and their free radical scavenging activities. Chemical and pharmaceutical bulletin 2002, 50 (5), 665-668.

(609) Choudhary, M. I.; Begum, A.; Abbaskhan, A.; Musharraf, S. G.; Ejaz, A. J. C.; biodiversity. Two new antioxidant phenylpropanoids from Lindelofia stylosa. 2008, 5 (12), 2676-2683.

(610) Waisundara, V. Y.; Huang, M.; Hsu, A.; Huang, D.; Tan, B. K.-H. J. T. A. j. o. C. m. Characterization of the anti-diabetic and antioxidant effects of Rehmannia glutinosa in streptozotocin-induced diabetic Wistar rats. 2008, 36 (06), 1083-1104.

(611) He, Z.-D.; Lau, K.-M.; But, P. P.-H.; Jiang, R.-W.; Dong, H.; Ma, S.-C.; Fung, K.-P.; Ye, W.-C.; Sun, H.-D. Antioxidative Glycosides from the Leaves of Ligustrum r obustum. Journal of natural products 2003, 66 (6), 851-854.

(612) Lin, Y.-L.; Chang, Y.-Y.; Kuo, Y.-H.; Shiao, M.-S. Anti-Lipid-Peroxidative Principles from Tournefortia s armentosa. Journal of natural products 2002, 65 (5), 745-747.

(613) WANG, J.; LI, L.; LIU, C.-m.; LIU, Z.-j.; BAI, H.-l. J. L. J. o. T. C. M. Studies on the determination and antioxidant activities of total phenolic from different Chirita longgangensis var. hongyao extract [J]. 2011, 3.

(614) Wang, H.; Sun, Y.; Ye, W.-C.; Xiong, F.; Wu, J.-J.; Yang, C.-H.; Zhao, S.-X. Antioxidative phenylethanoid and phenolic glycosides from Picrorhiza scrophulariiflora. Chemical and pharmaceutical bulletin 2004, 52 (5), 615-617.

(615) Chen, H.; Li, M.; Zhang, C.; Du, W.; Shao, H.; Feng, Y.; Zhang, W.; Yang, S. Isolation and Identification of the Anti-Oxidant Constituents from Loropetalum chinense (R-Brown) Oliv. Based on UHPLC-Q-TOF-MS/MS. Molecules 2018, 23 (7). DOI: 10.3390/molecules23071720.

(616) Shi, J.; Gong, J.; Liu, J. e.; Wu, X.; Zhang, Y. Antioxidant capacity of extract from edible flowers of Prunus mume in China and its active components. Lwt-Food Science and Technology 2009, 42 (2), 477-482. DOI: 10.1016/j.lwt.2008.09.008.

(617) Ganzon, J. G.; Chen, L. G.; Wang, C. C. 4-O-Caffeoylquinic acid as an antioxidant marker for mulberry leaves rich in phenolic compounds. Journal of Food and Drug Analysis 2018, 26 (3), 985-993, Article. DOI: 10.1016/j.jfda.2017.11.011.

(618) Hamauzu, Y.; Yasui, H.; Inno, T.; Kume, C.; Omanyuda, M. Phenolic profile, antioxidant property, and anti-influenza viral activity of Chinese quince (Pseudocydonia sinensis Schneid.), quince (Cydonia oblonga Mill.), and apple (Malus domestica Mill.) fruits. Journal of Agricultural and Food Chemistry 2005, 53 (4), 928-934. DOI: 10.1021/jf0494635.

(619) Sun, Y.; Li, H.; Hu, J.; Li, J.; Fan, Y.-w.; Liu, X.-r.; Deng, Z.-y. Qualitative and Quantitative Analysis of Phenolics in Tetrastigma hemsleyanum and Their Antioxidant and Antiproliferative Activities. Journal of Agricultural and Food Chemistry 2013, 61 (44), 10507-10515. DOI: 10.1021/jf4037547.

(620) Xiang, Z.; Ning, Z. Scavenging and antioxidant properties of compound derived from chlorogenic acid in South-China honeysuckle. Lwt-Food Science and Technology 2008, 41 (7), 1189-1203. DOI: 10.1016/j.lwt.2007.08.006.

(621) Oliveira, I.; Valentao, P.; Lopes, R.; Andrade, P. B.; Bento, A.; Pereira, J. A. Phytochemical characterization and radical scavenging activity of Portulaca oleraceae L. leaves and stems. Microchemical Journal 2009, 92 (2), 129-134. DOI: 10.1016/j.microc.2009.02.006.

(622) Dan, T.; Hui-Jun, L.; Jun, C.; Chao-Wei, G.; Ping, L. Rapid and simple method for screening of natural antioxidants from Chinese herb Flos Lonicerae Japonicae by DPPH-HPLC-DAD-TOF/MS. Journal of separation science 2015, 31 (20), 3519-3526.

(623) Lee, W.-C.; Peng, C.-C.; Chang, C.-H.; Huang, S.-H.; Chyau, C.-C. Extraction of Antioxidant Components from Bidens pilosa Flowers and Their Uptake by Human Intestinal Caco-2 Cells. Molecules 2013, 18 (2), 1582-1601. DOI: 10.3390/molecules18021582.

(624) Liu, L.; Sun, Y.; Laura, T.; Liang, X.; Ye, H.; Zeng, X. Determination of polyphenolic content and antioxidant activity of kudingcha made from Ilex kudingcha C.J. Tseng. Food Chemistry 2009, 112 (1), 35-41. DOI: 10.1016/j.foodchem.2008.05.038.

(625) Zheleva-Dimitrova, D.; Gevrenova, R.; Zaharieva, M. M.; Najdenski, H.; Ruseva, S.; Lozanov, V.; Balabanova, V.; Yagi, S.; Momekov, G.; Mitev, V. HPLC-UV and LC–MS Analyses of Acylquinic Acids in Geigeria alata (DC) Oliv. & Hiern. and their Contribution to Antioxidant and Antimicrobial Capacity. Phytochemical Analysis Pca 2016, 28.

(626) da Silva, J. B.; Mendes, R. d. F.; Tomasco, V.; Campos Pinto, N. d. C.; de Oliveira, L. G.; Rodrigues, M. N.; de Oliveira Aragdoa, D. M.; Kopke de Aguiar, J. A.; Alves, M. S.; Nogueira Marques Castanon, M. C.; et al. New aspects on the hepatoprotective potential associated with the antioxidant, hypocholesterolemic and anti-inflammatory activities of Vernonia condensata Baker. Journal of Ethnopharmacology 2017, 198, 399-406. DOI: 10.1016/j.jep.2017.01.039.

(627) Pagano, I.; Piccinelli, A. L.; Celano, R.; Campone, L.; Gazzerro, P.; De Falco, E.; Rastrelli, L. Chemical profile and cellular antioxidant activity of artichoke by-products. Food & Function 2016, 7 (12), 4841-4850. DOI: 10.1039/c6fo01443g.

(628) Salami, M.; Rahimmalek, M.; Ehtemam, M. H. Inhibitory effect of different fennel (Foeniculum vulgare) samples and their phenolic compounds on formation of advanced glycation products and comparison of antimicrobial and antioxidant activities. Food Chemistry 2016, 213, 196-205. DOI: 10.1016/j.foodchem.2016.06.070.

(629) Younsi, F.; Trimech, R.; Boulila, A.; Ezzine, O.; Dhahri, S.; Boussaid, M.; Messaoud, C. Essential Oil and Phenolic Compounds of Artemisia herba-alba (Asso.): Composition, Antioxidant, Antiacetylcholinesterase, and

Antibacterial Activities. International Journal of Food Properties 2016, 19 (7), 1425-1438. DOI: 10.1080/10942912.2015.1079789.

(630) Kobayashi, M.; Kakizono, T.; Nishio, N.; Nagai, S.; Kurimura, Y.; Tsuji, Y. J. A. M.; Biotechnology. Antioxidant role of astaxanthin in the green alga Haematococcus pluvialis. 1997, 48 (3), 351-356.

(631) Kobayashi, M. J. A. m.; biotechnology. In vivo antioxidant role of astaxanthin under oxidative stress in the green alga Haematococcus pluvialis. 2000, 54 (4), 550-555.

(632) Kuroyanagi, M.; Shimomae, M.; Nagashima, Y.; Muto, N.; Okuda, T.; Kawahara, N.; Nakane, T.; Sano, T. New diarylheptanoids from Alnus japonica and their antioxidative activity. Chemical & pharmaceutical bulletin 2005, 53 (12), 1519-1523. DOI: 10.1248/cpb.53.1519.

(633) Wicha, P.; Tocharus, J.; Janyou, A.; Jittiwat, J.; Changtam, C.; Suksamrarn, A.; Tocharus, C. Hexahydrocurcumin protects against cerebral ischemia/reperfusion injury, attenuates inflammation, and improves antioxidant defenses in a rat stroke model. PloS one 2017, 12 (12). DOI: 10.1371/journal.pone.0189211.

(634) Guo, X.; Li, W. F.; Wang, H. P.; Fan, Y. Y.; Wang, H. F.; Gao, X. H.; Niu, B. L.; Gong, X. C. Preparation, characterization, release and antioxidant activity of curcumin-loaded amorphous calcium phosphate nanoparticles. Journal of Non-Crystalline Solids 2018, 500, 317-325, Article. DOI: 10.1016/j.jnoncrysol.2018.08.015.

(635) Serqueira, D. S.; Dornellas, R. M.; Silva, L. G.; de Melo, P. G.; Castellan, A.; Ruggiero, R.; Richter, E. M.; Munoz, R. A. A. Tetrahydrocurcuminoids as potential antioxidants for biodiesels. Fuel 2015, 160, 490-494, Article. DOI: 10.1016/j.fuel.2015.07.104.

(636) Yu, Q. Q.; Liu, Y. Y.; Wu, Y. F.; Chen, Y. Dihydrocurcumin ameliorates the lipid accumulation, oxidative stress and insulin resistance in oleic acid-induced L02 and HepG2 cells. Biomedicine & Pharmacotherapy 2018, 103, 1327-1336, Article. DOI: 10.1016/j.biopha.2018.04.143.

(637) Akter, J.; Hossain, M. A.; Takara, K.; Islam, M. Z.; Hou, D.-X. Antioxidant activity of different species and varieties of turmeric (Curcuma spp): Isolation of active compounds. Comparative biochemistry and physiology. Toxicology & pharmacology : CBP 2018, 215, 9-17. DOI: 10.1016/j.cbpc.2018.09.002.

(638) dos Santos, P. D. F.; Francisco, C. R. L.; Coqueiro, A.; Leimann, F. V.; Pinela, J.; Calhelha, R. C.; Ineu, R. P.; Ferreira, I.; Bona, E.; Goncalves, O. H. The nanoencapsulation of curcuminoids extracted from Curcuma longa L. and an evaluation of their cytotoxic, enzymatic, antioxidant and anti-inflammatory activities. Food & Function 2019, 10 (2), 573-582, Article. DOI: 10.1039/c8fo02431f.

(639) Puglia, C.; Santonocito, D.; Musumeci, T.; Cardile, V.; Graziano, A. C. E.; Salerno, L.; Raciti, G.; Crasci, L.; Panico, A. M.; Puglisi, G. Nanotechnological Approach to Increase the Antioxidant and Cytotoxic Efficacy of Crocin and Crocetin. Planta Medica 2019, 85 (3), 258-265, Article. DOI: 10.1055/a-0732-5757.

(640) Watanabe, M.; Ayugase, J. Improvement in Lipid Metabolism by Carotenoid Extracts from Pink Colored and Orange Colored Tomatoes in Mice Subjected to Restraint Stress. Journal of the Japanese Society for Food Science and Technology-Nippon Shokuhin Kagaku Kogaku Kaishi 2013, 60 (7), 332-338, Article.

(641) Can-Cauich, C. A.; Sauri-Duch, E.; Moo-Huchin, V. M.; Betancur-Ancona, D.; Cuevas-Glory, L. F. Effect of extraction method and specie on the content of bioactive compounds and antioxidant activity of pumpkin oil from Yucatan, Mexico. Food Chemistry 2019, 285, 186-193, Article. DOI: 10.1016/j.foodchem.2019.01.153.

(642) Galano, A.; Francisco-Marquez, M.; Alvarez-Idaboy, J. R. Mechanism and kinetics studies on the antioxidant activity of sinapinic acid. Phys Chem Chem Phys 2011, 13 (23), 11199-11205. DOI: 10.1039/c1cp20722a.

(643) Zhang, N.; Lian, Z.; Peng, X.; Li, Z.; Zhu, H. Applications of Higenamine in pharmacology and medicine. J Ethnopharmacol 2017, 196, 242-252. DOI: 10.1016/j.jep.2016.12.033.

(644) Zhao, Q.; Zhao, Y.; Wang, K. Antinociceptive and free radical scavenging activities of alkaloids isolated from Lindera angustifolia Chen. Journal of Ethnopharmacology 2006, 106 (3), 408-413. DOI: 10.1016/j.jep.2006.01.019.

(645) Costa, E. V.; Oliveira da Cruz, P. E.; de Lourenco, C. C.; de Souza Moraes, V. R.; de Lima Nogueira, P. C.; Salvador, M. J. Antioxidant and antimicrobial activities of aporphinoids and other alkaloids from the bark of Annona salzmannii A. DC. (Annonaceae). Natural product research 2013, 27 (11), 1002-1006. DOI: 10.1080/14786419.2012.688044.

(646) Bezakova, L.; Misik, V.; Malekova, L.; Svajdlenka, E.; Kostalova, D. Lipoxygenase inhibition and antioxidant properties of bisbenzylisoquinoline alkaloids isolated from Mahonia aquifolium. Pharmazie 1996, 51 (10), 758-761.

(647) Jia, F.; Ruan, S.; Liu, N.; Fu, L. Synergistic Antitumor Effects of Berbamine and Paclitaxel through ROS/Akt Pathway in Glioma Cells. Evidence-based complementary and alternative medicine : eCAM 2017, 2017, 8152526-8152526. DOI: 10.1155/2017/8152526.

(648) Zarei, A.; Changizi-Ashtiyani, S.; Taheri, S.; Ramezani, M. A quick overview on some aspects of endocrinological and therapeutic effects of Berberis vulgaris L. Avicenna journal of phytomedicine 2015, 5 (6), 485-497.

(649) Khan, A.; Bai, H.; Liu, E.; Chen, M.; Yu, C.; Wang, R.; Khan, A.; Bai, Z. Protective effect of neferine against UV-B-mediated oxidative damage in human epidermal keratinocytes. Journal of Dermatological Treatment 2018, 29 (7), 733-741. DOI: 10.1080/09546634.2018.1441490.

(650) Xie, Y.; Zhang, Y.; Guo, Z.; Zeng, H.; Zheng, B. EFFECTS OF NEFERINE FROM NELUMBINIS PLUMULA ON OLEIC ACID-INDUCED HEPATIC STEATOSIS IN VITRO VIA ITS ANTIOXIDANT ACTIVITY. Current Topics in Nutraceutical Research 2017, 15 (3-4), 195-204.

(651) Gulcin, I.; Elias, R.; Gepdiremen, A.; Chea, A.; Topal, F. Antioxidant activity of bisbenzylisoquinoline alkaloids from Stephania rotunda: cepharanthine and fangchinoline. Journal of Enzyme Inhibition and Medicinal Chemistry 2010, 25 (1), 44-53. DOI: 10.3109/14756360902932792.

(652) Gomez, G. I.; Velarde, V. Boldine Improves Kidney Damage in the Goldblatt 2K1C Model Avoiding the Increase in TGF-beta. International Journal of Molecular Sciences 2018, 19 (7). DOI: 10.3390/ijms19071864.

(653) Moezi, L.; Yahosseini, S.; Jamshizadeh, A.; Pirsalami, F. Acute Boldine Treatment Induces Anti-convulsant Effects in Mice through its Antioxidant Activity. Drug research 2018. DOI: 10.1055/a-0659-2478.

(654) Zahari, A.; Ablat, A.; Siyasothy, Y.; Mohamad, J.; Choudhary, M. I.; Awang, K. In vitro antiplasmodial and antioxidant activities of bisbenzylisoquinoline alkaloids from Alseodaphne corneri Kosterm. Asian Pacific Journal of Tropical Medicine 2016, 9 (4), 320-324. DOI: 10.1016/j.apjtm.2016.03.008.

(655) Li, C.; Wang, M.-H. Potential Biological Activities of Magnoflorine: A Compound from Aristolochia debilis Sieb. et Zucc. Korean Journal of Plant Reources. 2014, 27 (3), 223-228. DOI: 10.7732/kjpr.2014.27.3.223.

(656) Luo, T.; Shen, X.-Y.; Li, S.; Ouyang, T.; Mai, Q.-A.; Wang, H.-Q. The Protective Effect of Jatrorrhizine Against Oxidative Stress in Primary Rat Cortical Neurons. Cns & Neurological Disorders-Drug Targets 2017, 16 (5), 617-623. DOI: 10.2174/1871527315666160711101210.

(657) Ubeda, A.; Montesinos, C.; Paya, M.; Terencio, C.; Alcaraz, M. J. Antioxidant action of benzylisoquinoline alkaloids. Free radical research communications 1993, 18 (3), 167-175. DOI: 10.3109/10715769309147490.

(658) Fan, H.; Shu, Q.; Guan, X.; Zhao, J.; Yan, J.; Li, X.; Liu, J.; Jia, Z.; Shi, J.; Li, J. Sinomenine Protects PC12 Neuronal Cells against H2O2-induced Cytotoxicity and Oxidative Stress via a ROS-dependent Up-regulation of Endogenous Antioxidant System. Cellular and Molecular Neurobiology 2017, 37 (8), 1387-1398. DOI: 10.1007/s10571-017-0469-1.

(659) Zhou, Y.; Liu, H.; Song, J.; Cao, L.; Tang, L.; Qi, C. Sinomenine alleviates dextran sulfate sodium-induced colitis via the Nrf2/NQO-1 signaling pathway. Molecular Medicine Reports 2018, 18 (4), 3691-3698. DOI: 10.3892/mmr.2018.9378.

(660) Kiplimo, J. J.; Islam, M. S.; Koorbanally, N. A. A Novel Flavonoid and Furoquinoline Alkaloids from Vepris glomerata and their Antioxidant Activity. Natural Product Communications 2011, 6 (12), 1847-1850.

(661) Kim, D. H.; Jang, Y. Y.; Han, E. S.; Lee, C. S. Protective effect of harmaline and harmalol against dopamine- and 6-hydroxydopamine-induced oxidative damage of brain mitochondria and synaptosomes, and viability loss of PC12 cells. European Journal of Neuroscience 2001, 13 (10), 1861-1872. DOI: 10.1046/j.0953-816x.2001.01563.x.

(662) Su, S.; Cao, M.; Wu, G.; Long, Z.; Cheng, X.; Fan, J.; Xu, Z.; Su, H.; Hao, Y.; Li, G.; et al. Hordenine protects against hyperglycemia-associated renal complications in streptozotocin-induced diabetic mice. Biomedicine & Pharmacotherapy 2018, 104, 315-324. DOI: 10.1016/j.biopha.2018.05.036.

(663) Li, X.; Ren, Z.; Wu, Z.; Fu, Z.; Xie, H.; Deng, L.; Jiang, X.; Chen, D. Steric Effect of Antioxidant Diels-Alder-Type Adducts: A Comparison of Sanggenon C with Sanggenon D. Molecules (Basel, Switzerland) 2018, 23 (10). DOI: 10.3390/molecules23102610.

(664) Chen, L.-D.; Liu, Z.-H.; Zhang, L.-F.; Yao, J.-N.; Wang, C.-F. Sanggenon C induces apoptosis of colon cancer cells via inhibition of NO production, iNOS expression and ROS activation of the mitochondrial pathway. Oncology reports 2017, 38 (4), 2123-2131. DOI: 10.3892/or.2017.5912.

(665) Zhang, Q.-J.; Ni, G.; Wang, Y.-H.; Chen, R.-Y.; Yu, D.-Q. Three new Diels-Alder type adducts from the stem bark of Morus cathayana. Journal of Asian Natural Products Research 2009, 11 (3), 267-273. DOI: 10.1080/10286020902767690.

(666) Cui, X.-Q.; Wang, H.-Q.; Liu, C.; Chen, R.-Y. Study of anti-oxidant phenolic compounds from stem barks of Morus yunanensis. Zhongguo Zhong yao za zhi = Zhongguo zhongyao zazhi = China journal of Chinese materia medica 2008, 33 (13), 1569-1572.

(667) Dai, S. J.; Wu, Y.; Wang, Y. H.; He, W. Y.; Chen, R. Y.; Yu, D. Q. New Diels-Alder type adducts from Morus macroura and their anti-oxidant activities. Chemical & pharmaceutical bulletin 2004, 52 (10), 1190-1193. DOI: 10.1248/cpb.52.1190.

(668) Hong, S.; Kwon, J.; Kim, D.-W.; Lee, H. J.; Lee, D.; Mar, W. Mulberrofuran G Protects Ischemic Injury-induced Cell Death via Inhibition of NOX4-mediated ROS Generation and ER Stress. Phytotherapy Research 2017, 31 (2), 321-329. DOI: 10.1002/ptr.5754.

(669) Abbas, G. M.; Bar, F. M. A.; Baraka, H. N.; Gohar, A. A.; Lahloub, M.-F. A new antioxidant stilbene and other constituents from the stem bark of Morus nigra L. Natural product research 2014, 28 (13), 952-959. DOI: 10.1080/14786419.2014.900770.

(670) Dai, S. J.; Ma, Z. B.; Wu, Y.; Chen, R. Y.; Yu, D. Q. Guangsangons F-J, anti-oxidant and anti-inflammatory Diels-Alder type adducts, from Morus macroura Miq. Phytochemistry 2004, 65 (23), 3135-3141. DOI: 10.1016/j.phytochem.2004.07.010.

(671) Tan, Y.-X.; Yan, R.-Y.; Wang, H.-Q.; Chen, R.-Y.; Yu, D.-Q. Wittiorumins A-F, Antioxidant Diels-Alder-Type Adducts from Morus wittiorum. Planta medica 2009, 75 (3), 249-255. DOI: 10.1055/s-0028-1112202.

(672) Zhou, R. F.; Long, H. S.; Zhang, B.; Lao, Z. Z.; Zheng, Q. Y.; Wang, T. C.; Zhang, Y. X.; Wu, Q. G.; Lai, X. P.; Li, G.; et al. Salvianolic acid B, an antioxidant derived from Salvia militarize, protects mice against -radiation-induced damage through Nrf2/Bach1. Molecular Medicine Reports 2019, 19 (2), 1309-1317, Article. DOI: 10.3892/mmr.2018.9718.

(673) Chan, P. C.; Xia, Q. S.; Fu, P. P. Ginkgo biloba leave extract: Biological, medicinal, and toxicological effects. Journal of Environmental Science and Health Part C-Environmental Carcinogenesis & Ecotoxicology Reviews 2007, 25 (3), 211-244, Review. DOI: 10.1080/10590500701569414.

(674) Lee, M. S.; Shin, T.; Utsuki, T.; Choi, J. S.; Byun, D. S.; Kim, H. R. Isolation and Identification of Phlorotannins from Ecklonia stolonifera with Antioxidant and Hepatoprotective Properties in Tacrine-Treated HepG2 Cells. Journal of Agricultural and Food Chemistry 2012, 60 (21), 5340-5349, Article. DOI: 10.1021/jf300157w.

(675) Shibata, T.; Ishimaru, K.; Kawaguchi, S.; Yoshikawa, H.; Hama, Y. Antioxidant activities of phlorotannins isolated from Japanese Laminariaceae. Journal of Applied Phycology 2008, 20 (5), 705-711, Article. DOI: 10.1007/s10811-007-9254-8.

(676) Gupta, R.; Sharma, A. K.; Sharma, M. C.; Gupta, R. S. Antioxidant activity and protection of pancreatic ss-cells by embelin in streptozotocin-induced diabetes. Journal of Diabetes 2012, 4 (3), 248-256. DOI: 10.1111/j.1753-0407.2012.00187.x.

(677) Mahendran, S.; Badami, S.; Ravi, S.; Thippeswamy, B. S.; Veerapur, V. P. ANTIOXIDANT, ANALGESIC AND ANTI-INFLAMMATORY PROPERTIES OF NEW NINHYDRIN ADDUCT OF EMBELIN. Pharmaceutical Chemistry Journal 2011, 45 (9), 547-551. DOI: 10.1007/s11094-011-0676-x.

(678) Lee, M.-S.; Kim, J.-I.; Utsuki, T.; Park, N.-G.; Kim, H.-R. Cytoprotective effects of phlorofucofuroeckol A isolated from Ecklonia stolonifera against tacrine-treated HepG2 cells. Fitoterapia 2012, 83 (6), 1060-1067. DOI: 10.1016/j.fitote.2012.05.007.

(679) Masaki, H.; Atsumi, T.; Sakurai, H. HAMAMELITANNIN AS A NEW POTENT ACTIVE OXYGEN SCAVENGER. Phytochemistry 1994, 37 (2), 337-343. DOI: 10.1016/0031-9422(94)85057-7.

(680) Sanchez-Tena, S.; Fernandez-Cachon, M. L.; Carreras, A.; Luisa Mateos-Martin, M.; Costoya, N.; Moyer, M. P.; Nunez, M. J.; Torres, J. L.; Cascante, M. Hamamelitannin from Witch Hazel (Hamamelis virginiana) Displays Specific Cytotoxic Activity against Colon Cancer Cells. Journal of Natural Products 2012, 75 (1), 26-33. DOI: 10.1021/np200426k.

(681) Aree, T.; Jongrungruangchok, S. Structure-antioxidant activity relationship of beta-cyclodextrin inclusion complexes with olive tyrosol, hydroxytyrosol and oleuropein: Deep insights from X-ray analysis, DFT calculation and DPPH assay. Carbohydrate polymers 2018, 199, 661-669. DOI: 10.1016/j.carbpol.2018.07.019.

(682) Liu, M.; Yong, Q.; Yu, S. Efficient bioconversion of oleuropein from olive leaf extract to antioxidant hydroxytyrosol by enzymatic hydrolysis and high-temperature degradation. Biotechnology and Applied Biochemistry 2018, 65 (5), 680-689. DOI: 10.1002/bab.1651.

(683) Andrade, M. A.; Ribeiro-Santos, R.; Conceicao Costa Bonito, M.; Saraiva, M.; Sanches-Silva, A. Characterization of rosemary and thyme extracts for incorporation into a whey protein based film. Lwt-Food Science and Technology 2018, 92, 497-508. DOI: 10.1016/j.lwt.2018.02.041.

(684) Alejandro Hernandez, D.; Tenorio, F. J. Reactivity indexes of antioxidant molecules from Rosmarinus officinalis. Structural Chemistry 2018, 29 (3), 741-751. DOI: 10.1007/s11224-017-1066-0.

(685) Tripathi, Y. B.; Sharma, M. Comparison of the antioxidant action of the alcoholic extract of Rubia cordifolia with rubiadin. Indian Journal of Biochemistry & Biophysics 1998, 35 (5), 313-316.

(686) Tripathi, Y. B.; Sharma, M.; Manickam, M. Rubiadin, a new antioxidant from Rubia cordifolia. Indian Journal of Biochemistry & Biophysics 1997, 34 (3), 302-306.

(687) Han, H.; Weng, X.; Bi, K. Antioxidants from a Chinese medicinal herb - Lithospermum erythrorhizon. Food Chemistry 2008, 106 (1), 2-10. DOI: 10.1016/j.foodchem.2007.01.031.

(688) Janicsak, G.; Hohmann, J.; Zupko, I.; Forgo, P.; Redei, D.; Falkay, G.; Mathe, I. Diterpenes from the aerial parts of Salvia candelabrum and their protective effects against lipid peroxidation. Planta medica 2003, 69 (12), 1156-1159.

(689) Mathe, I.; Hohmann, J.; Janicsak, G.; Nagy, G.; Dora, R. Chemical diversity of the biological active ingredients of salvia officinalis and some closely related species. Acta pharmaceutica Hungarica 2007, 77 (1), 37-45.

(690) Ozarowski, M.; Piasecka, A.; Gryszczynska, A.; Sawikowska, A.; Pietrowiak, A.; Opala, B.; Mikolajczak, P. L.; Kujawski, R.; Kachlicki, P.; Buchwald, W.; et al. Determination of phenolic compounds and diterpenes in roots of Salvia miltiorrhiza and Salvia przewalskii by two LC-MS tools: Multi-stage and high resolution tandem mass spectrometry with assessment of antioxidant capacity. Phytochemistry Letters 2017, 20, 331-338. DOI: 10.1016/j.phytol.2016.12.001.

(691) Christopher, R.; Nyandoro, S. S.; Chacha, M.; de Koning, C. B. A new cinnamoylglycoflavonoid, antimycobacterial and antioxidant constituents from Heritiera littoralis leaf extracts. Natural product research 2014, 28 (6), 351-358. DOI: 10.1080/14786419.2013.863202.

(692) Khalil, A. A.; Khan, M. R.; Shabbir, M. A.; Khalil ur, R. IN VITRO ANTIOXIDANT ACTIVITY AND PUNICALAGIN CONTENT QUANTIFICATION OF POMEGRANATE PEEL OBTAINED AS AGRO-WASTE AFTER JUICE EXTRACTION. Pakistan Journal of Agricultural Sciences 2018, 55 (1), 197-201. DOI: 10.21162/pakjas/18.5663.

(693) Oudane, B.; Boudemagh, D.; Bounekhel, M.; Sobhi, W.; Vidal, M.; Broussy, S. Isolation, characterization, antioxidant activity, and protein-precipitating capacity of the hydrolyzable tannin punicalagin from pomegranate yellow peel (Punica granatum). Journal of Molecular Structure 2018, 1156, 390-396. DOI: 10.1016/j.molstruc.2017.11.129.

(694) Bhattacharjee, P.; Bera, I.; Chakraborty, S.; Ghoshal, N.; Bhattacharyya, D. Aristolochic acid and its derivatives as inhibitors of snake venom L-amino acid oxidase. Toxicon 2017, 138, 1-17. DOI: 10.1016/j.toxicon.2017.08.003.

(695) Aree, T.; Jongrungruangchok, S. beta-Cyclodextrin encapsulation elevates antioxidant capacity of tea: A closing chapter on non-epicatechins, atomistic insights from X-ray analysis, DFT calculation and DPPH assay. Carbohydrate polymers 2018, 194, 24-33. DOI: 10.1016/j.carbpol.2018.04.016.

(696) Martini, S.; Conte, A.; Tagliazucchi, D. Bioaccessibility, bioactivity and cell metabolism of dark chocolate phenolic compounds after in vitro gastro-intestinal digestion. Journal of Functional Foods 2018, 49, 424-436. DOI: 10.1016/j.jff.2018.09.005.

(697) Lad, H.; Bhatnagar, D. Amelioration of oxidative and inflammatory changes by Swertia chirayita leaves in experimental arthritis. Inflammopharmacology 2016, 24 (6), 363-375. DOI: 10.1007/s10787-016-0290-3.

(698) He, Z. D.; But, P. P. H.; Chan, T. W. D.; Dong, H.; Xu, H. X.; Lau, C. P.; Sun, H. D. Antioxidative glucosides from the fruits of Ligustrum lucidum. Chemical & pharmaceutical bulletin 2001, 49 (6), 780-784. DOI: 10.1248/cpb.49.780.

(699) Ma, S. C.; He, Z. D.; Deng, X. L.; But, P. P. H.; Ooi, V. E. C.; Xu, H. X.; Lee, S. H. S.; Lee, S. F. In vitro evaluation of secoiridoid glucosides from the fruits of Ligustrum lucidum as antiviral agents. Chemical & pharmaceutical bulletin 2001, 49 (11), 1471-1473. DOI: 10.1248/cpb.49.1471.

(700) Tada, M.; Ohkanda, T.; Kurabe, J. Syntheses of Carnosic Acid and Carnosol, Anti-oxidants in Rosemary, from Pisiferic Acid, the Major Constituent of Sawara. Chemical & pharmaceutical bulletin 2010, 58 (1), 27-29. DOI: 10.1248/cpb.58.27.

(701) Yatagai, M.; Nakatani, N. ANTIMITE, ANTIFLY, ANTIOXIDATIVE, AND ANTIBACTERIAL ACTIVITIES OF PISIFERIC ACID AND ITS CONGENERS. Mokuzai Gakkaishi 1994, 40 (12), 1355-1362.

(702) Narukawa, Y.; Shimizu, N.; Shimotohno, K.; Takeda, T. Two new diterpenoids from Plectranthus nummularius BRIQ. Chemical & pharmaceutical bulletin 2001, 49 (9), 1182-1184. DOI: 10.1248/cpb.49.1182.

(703) Quintero, C.; Vera, R.; Dario Perez, L. alpha-Tocopherol loaded thermosensitive polymer nanoparticles: preparation, in vitro release and antioxidant properties. Polimeros-Ciencia E Tecnologia 2016, 26 (4), 304-312. DOI: 10.1590/0104-1428.2324.

(704) Pantoja-Chamorro, A. L.; Hurtado-Benavides, A. M.; Martínez-Correa, H. A. Evaluación del Rendimiento, Composición y Actividad Antioxidante de Aceite de Semillas de Mora (Rubus glaucus) Extraído con CO2 Supercrítico. Información tecnológica 2017, 28 (1), 35-46.

(705) Iwatake, M.; Okamoto, K.; Tanaka, T.; Tsukuba, T. Castalagin Exerts Inhibitory Effects on Osteoclastogenesis Through Blocking a Broad Range of Signaling Pathways with Low Cytotoxicity. Phytotherapy Research 2015, 29 (6), 917-924. DOI: 10.1002/ptr.5333.

(706) Kaneshima, T.; Myoda, T.; Nakata, M.; Fujimori, T.; Toeda, K.; Nishizawa, M. Antioxidant activity of C-Glycosidic ellagitannins from the seeds and peel of camu-camu (Myrciaria dubia). Lwt-Food Science and Technology 2016, 69, 76-81. DOI: 10.1016/j.lwt.2016.01.024.

(707) Aroso, I. M.; Araujo, A. R.; Fernandes, J. P.; Santos, T.; Batista, M. T.; Pires, R. A.; Mano, J. F.; Reis, R. L. Hydroalcoholic extracts from the bark of Quercus suber L. (Cork): optimization of extraction conditions, chemical composition and antioxidant potential. Wood Science and Technology 2017, 51 (4), 855-872. DOI: 10.1007/s00226-017-0904-y.

(708) Muccilli, V.; Cardullo, N.; Spatafora, C.; Cunsolo, V.; Tringali, C. alpha-Glucosidase inhibition and antioxidant activity of an oenological commercial tannin. Extraction, fractionation and analysis by HPLC/ESI-MS/MS and H-1 NMR. Food Chemistry 2017, 215, 50-60. DOI: 10.1016/j.foodchem.2016.07.136.

(709) Kahkonen, M.; Kylli, P.; Ollilainen, V.; Salminen, J.-P.; Heinonen, M. Antioxidant Activity of Isolated Ellagitannins from Red Raspberries and Cloudberries. Journal of Agricultural and Food Chemistry 2012, 60 (5), 1167-1174. DOI: 10.1021/jf203431g.

(710) Zamurrad, S.; Parrino, S.; Daccueil, F.; Roemer, E. J.; Simon, S. R. In vitro investigation of antioxidant and antiproteolytic properties of the clove extracts: Tellimagrandin I and Casuarictin. In Vitro Cellular & Developmental Biology-Animal 2008, 44, S47-S47.

(711) Luo, W.; Zhao, M.; Yang, B.; Ren, J.; Shen, G.; Rao, G. Antioxidant and antiproliferative capacities of phenolics purified from Phyllanthus emblica L. fruit. Food Chemistry 2011, 126 (1), 277-282. DOI: 10.1016/j.foodchem.2010.11.018.

(712) Reddy, D. B.; Reddanna, P. Chebulagic acid (CA) attenuates LPS-induced inflammation by suppressing NF-kappa B and MAPK activation in RAW 264.7 macrophages. Biochemical and Biophysical Research Communications 2009, 381 (1), 112-117. DOI: 10.1016/j.bbrc.2009.02.022.

(713) Klika, K. D.; Saleem, A.; Sinkkonen, J.; Kahkonen, M.; Loponen, J.; Tahtinen, P.; Pihlaja, K. The structural and conformational analyses and antioxidant activities of chebulinic acid and its thrice-hydrolyzed derivative, 2,4-chebuloyl-beta-D-glucopyranoside, isolated from the fruit of Terminalia chebula. Arkivoc 2004, 83-105.

(714) Upadhyay, A.; Agrahari, P.; Singh, D. K. A Review on the Pharmacological Aspects of Terminalia chebula. International Journal of Pharmacology 2014, 10 (6), 289-298. DOI: 10.3923/ijp.2014.289.298.

(715) Ding, Y.; Ren, D.; Xu, H.; Liu, W.; Liu, T.; Li, L.; Li, J.; Li, Y.; Wen, A. Antioxidant and pro-angiogenic effects of corilagin in rat cerebral ischemia via Nrf2 activation. Oncotarget 2017, 8 (70), 114816-114828. DOI: 10.18632/oncotarget.22023.

(716) Reddy, B. U.; Mullick, R.; Kumar, A.; Sharma, G.; Bag, P.; Roy, C. L.; Sudha, G.; Tandon, H.; Dave, P.; Shukla, A.; et al. A natural small molecule inhibitor corilagin blocks HCV replication and modulates oxidative stress to reduce liver damage. Antiviral Research 2018, 150, 47-59. DOI: 10.1016/j.antiviral.2017.12.004.

(717) Aayadi, H.; Mittal, S. P. K.; Deshpande, A.; Gore, M.; Ghaskadbi, S. S. Cytoprotective effect exerted by geraniin in HepG2 cells is through microRNA mediated regulation of BACH-1 and HO-1. Bmb Reports 2017, 50 (11), 560-565. DOI: 10.5483/BMBRep.2017.50.11.060.

(718) Chung, A. P. Y. S.; Gurtu, S.; Chakravarthi, S.; Moorthy, M.; Palanisamy, U. D. Geraniin Protects High-Fat Diet-Induced Oxidative Stress in Sprague Dawley Rats. Frontiers in nutrition 2018, 5, 17-17. DOI: 10.3389/fnut.2018.00017.

(719) Tabata, H.; Katsube, T.; Tsuma, T.; Ohta, Y.; Imawaka, N.; Utsumi, T. Isolation and evaluation of the radical-scavenging activity of the antioxidants in the leaves of an edible plant, Mallotus japonicus. Food Chemistry 2008, 109 (1), 64-71. DOI: 10.1016/j.foodchem.2007.12.017.

(720) Yang, C.; Li, F.; Zhang, X.; Wang, L.; Zhou, Z.; Wang, M. Phenolic antioxidants from Rosa soulieana flowers. Natural product research 2013, 27 (21), 2055-2058. DOI: 10.1080/14786419.2013.811660.

(721) Zhou, B.; Yang, L.; Liu, Z. L. Strictinin as an efficient antioxidant in lipid peroxidation. Chemistry and Physics of Lipids 2004, 131 (1), 15-25. DOI: 10.1016/j.chemphyslip.2004.03.007.

(722) Yin, T.-P.; Cai, L.; Chen, Y.; Li, Y.; Wang, Y.-R.; Liu, C.-S.; Ding, Z.-T. Tannins and Antioxidant Activities of the Walnut (Juglans regia) Pellicle. Natural Product Communications 2015, 10 (12), 2141-2144.

(723) Eyles, A.; Davies, N. W.; Mitsunaga, T.; Mihara, R.; Mohammed, C. Role of Eucalyptus globulus wound wood extractives: evidence of superoxide dismutase-like activity. Forest Pathology 2004, 34 (4), 225-232. DOI: 10.1111/j.1439-0329.2004.00361.x.

(724) Chen, Y.; Wang, J.; Ou, Y.; Chen, H.; Xiao, S.; Liu, G.; Cao, Y.; Huang, Q. Cellular antioxidant activities of polyphenols isolated from Eucalyptus leaves (Eucalyptus grandis x Eucalyptus urophylla GL9). Journal of Functional Foods 2014, 7, 737-745. DOI: 10.1016/j.jff.2013.12.003.

(725) Ganesan, D.; Al-Sayed, E.; Albert, A.; Paul, E.; Singab, A. N. B.; Sadasivam, S. G.; Saso, L. Antioxidant activity of phenolic compounds from extracts of Eucalyptus globulus and Melaleuca styphelioides and their protective role on D-glucose-induced hyperglycemic stress and oxalate stress in NRK-49Fcells. Natural product research 2018, 32 (11), 1274-1280. DOI: 10.1080/14786419.2017.1343324.

(726) Chang, M. Y.; Byon, S.-H.; Shin, H.-C.; Han, S. E.; Kim, J. Y.; Byun, J. Y.; Lee, J. D.; Park, M. K. Protective effects of the seaweed phlorotannin polyphenolic compound dieckol on gentamicin-induced damage in auditory hair cells. International Journal of Pediatric Otorhinolaryngology 2016, 83 (4), 31-36. DOI: 10.1016/j.ijporl.2016.01.014.

(727) Lee, M.-S.; Lee, B.; Park, K.-E.; Utsuki, T.; Shin, T.; Oh, C. W.; Kim, H.-R. Dieckol enhances the expression of antioxidant and detoxifying enzymes by the activation of Nrf2-MAPK signaling pathway in HepG2 cells (vol 174, pg 538, 2015). Food Chemistry 2016, 202, 521-521. DOI: 10.1016/j.foodchem.2016.02.084.

(728) Kang, H. S.; Chung, H. Y.; Jung, J. H.; Son, B. W.; Choi, J. S. A new phlorotannin from the brown alga Ecklonia stolonifera. Chemical & pharmaceutical bulletin 2003, 51 (8), 1012-1014.

(729) Jang, J.; Ye, B.-R.; Heo, S.-J.; Oh, C.; Kang, D.-H.; Kim, J. H.; Affan, A.; Yoon, K.-T.; Choi, Y.-U.; Park, S. C.; et al. Photo-oxidative stress by ultraviolet-B radiation and antioxidative defense of eckstolonol in human keratinocytes. Environmental Toxicology and Pharmacology 2012, 34 (3), 926-934. DOI: 10.1016/j.etap.2012.08.003.

(730) Kang, H. S.; Chung, H. Y.; Kim, J. Y.; Son, B. W.; Jung, H. A.; Choi, J. S. Inhibitory phlorotannins from the edible brown alga Ecklonia stolonifera on total reactive oxygen species (ROS) generation. Archives of Pharmacal Research 2004, 27 (2), 194-198. DOI: 10.1007/bf02980106.

(731) Ooi, D. J.; Chan, K. W.; Sarega, N.; Alitheen, N. B.; Ithnin, H.; Ismail, M. Bioprospecting the Curculigoside-Cinnamic Acid-Rich Fraction from Molineria latifolia Rhizome as a Potential Antioxidant Therapeutic Agent. Molecules 2016, 21 (6). DOI: 10.3390/molecules21060682.

(732) Wang, Y.; Zhao, L.; Wang, Y.; Xu, J.; Nie, Y.; Guo, Y.; Tong, Y.; Qin, L.; Zhang, Q. Curculigoside isolated from Curculigo orchioides prevents hydrogen peroxide-induced dysfunction and oxidative damage in calvarial osteoblasts. Acta Biochimica Et Biophysica Sinica 2012, 44 (5), 431-441. DOI: 10.1093/abbs/gms014.

(733) Ono, M.; Masuoka, C.; Koto, M.; Tateishi, M.; Komatsu, H.; Kobayashi, H.; Igoshi, K.; Ito, Y.; Okawa, M.; Nohara, T. Antioxidant ortho-benzoyloxyphenyl acetic acid ester, vaccihein A, from the fruit of rabbiteye blueberry (Vaccinium ashei). Chemical & pharmaceutical bulletin 2002, 50 (10), 1416-1417. DOI: 10.1248/cpb.50.1416.

(734) Ye, L.; Gong, J.; Wang, Y.; Sun, X. Pharmacological Activities of Sijunzi Decoction Which Are Related to Its Antioxidant Properties. Journal of Chemistry 2014. DOI: 10.1155/2014/278318.

(735) Pang, L.; Zhu, K.; Feng, X.; Liu, W.; Peng, D.; Qiu, L.; Gao, X.; Deng, J.; Li, Y.; Zhao, X. Protective effect of liensinine on periodontitis through its antioxidant effect in mice. Journal of the Korean Society for Applied Biological Chemistry 2015, 58 (6), 927-936. DOI: 10.1007/s13765-015-0122-y.

(736) Tsai, J. Y.; Chou, C. J.; Chen, C. F.; Chiou, W. F. Antioxidant activity of piperlactam S: Prevention of copper-induced LDL peroxidation and amelioration of free radical-induced oxidative stress of endothelial cells. Planta medica 2003, 69 (1), 3-8. DOI: 10.1055/s-2003-37040.

(737) Riaz, N.; Malik, A.; Rehman, A.; Ahmed, Z.; Muhammad, P.; Nawaz, S. A.; Siddiqui, J.; Choudhary, M. I. Lipoxygenase inhibiting and antioxidant oligostilbene and monoterpene galactoside from Paeonia emodi. Phytochemistry 2004, 65 (8), 1129-1135. DOI: 10.1016/j.phytochem.2003.10.012.

(738) Jun, H. U.; Yan, X.; Wei, W.; Hao, W. U.; Lei, H.; Lijun, D. U. Antioxidant Activity In Vitro of Three Constituents from Caesalpinia sappan L. Tsinghua Science & Technology 2008, 13 (4), 474-479.

(739) Sasaki, Y.; Hosokawa, T.; Nagai, M.; Nagumo, S. In vitro study for inhibition of NO production about constituents of Sappan Lignum. Biological & Pharmaceutical Bulletin 2007, 30 (1), 193-196, Article. DOI: 10.1248/bpb.30.193.

(740) Singh, S.; Khatoon, S.; Joshi, Y.; Prgyadeep, S.; Upreti, D. K.; Rawat, A. K. S. A Validated HPTLC Densitometric Method for Simultaneous Determination of Evernic and Usnic Acids in Four Usnea Species and Comparison of Their Antioxidant Potential. Journal of chromatographic science 2016, 54 (9), 1670-1677. DOI: 10.1093/chromsci/bmw118.

(741) Popovici, V.; Bucur, L.; Popescu, A.; Caraiane, A.; Badea, V. DETERMINATION OF THE CONTENT IN USNIC ACID AND POLYPHENOLS FROM THE EXTRACTS OF USNEA BARBATA L. AND THE EVALUATION OF THEIR ANTIOXIDANT ACTIVITY. Farmacia 2018, 66 (2), 337-341.

(742) Youns, M.; ElKhoely, A.; Kamel, R. The growth inhibitory effect of gambogic acid on pancreatic cancer cells. Naunyn-Schmiedebergs Archives of Pharmacology 2018, 391 (5), 551-560. DOI: 10.1007/s00210-018-1485-5.

(743) Nabi, F.; Li, K.; Shahzad, M.; Han, Z.; Zhang, D.; Liu, J.; Li, J. Gambogic Acid Inhibits Hsp90 Expressions in Thiram-Induced Tibial Dyschondroplasia. Pakistan Veterinary Journal 2016, 36 (2), 224-226.

(744) da Silva Padilha, C. V.; Telles Biasoto, A. C.; Correa, L. C.; Lima, M. d. S.; Pereira, G. E. Phenolic compounds profile and antioxidant activity of commercial tropical red wines (Vitis vinifera L.) from Sao Francisco Valley, Brazil. Journal of Food Biochemistry 2017, 41 (3). DOI: 10.1111/jfbc.12346.

(745) Li, S.; Yang, Y.; Li, J.; Zhu, Z.; Lorenzo, J. M.; Barba, F. J. Increasing Yield and Antioxidative Performance of Litchi Pericarp Procyanidins in Baked Food by Ultrasound-Assisted Extraction Coupled with Enzymatic Treatment. Molecules 2018, 23 (9). DOI: 10.3390/molecules23092089.

(746) Mizuno, M.; Nakanishi, I.; Matsubayashi, S.; Imai, K.; Arai, T.; Matsumoto, K.-i.; Fukuhara, K. Synthesis and antioxidant activity of a procyanidin B3 analogue. Bioorganic & Medicinal Chemistry Letters 2017, 27 (4), 1041-1044. DOI: 10.1016/j.bmcl.2016.12.067.

(747) Mizuno, M.; Nakanishi, I.; Matsumoto, K.-i.; Fukuhara, K. Enhanced radical scavenging activity of a procyanidin B3 analogue comprised of a dimer of planar catechin. Bioorganic & Medicinal Chemistry Letters 2017, 27 (22), 5010-5013. DOI: 10.1016/j.bmcl.2017.10.007.

(748) Raudone, L.; Raudonis, R.; Liaudanskas, M.; Viskelis, J.; Pukalskas, A.; Janulis, V. Phenolic Profiles and Contribution of Individual Compounds to Antioxidant Activity of Apple Powders. Journal of Food Science 2016, 81 (5), C1055-C1061. DOI: 10.1111/1750-3841.13277.

(749) Justino, A. B.; Miranda, N. C.; Franco, R. R.; Martins, M. M.; da Silva, N. M.; Espindola, F. S. Annona muricata Linn. leaf as a source of antioxidant compounds with in vitro antidiabetic and inhibitory potential against alpha-amylase, alpha-glucosidase, lipase, non-enzymatic glycation and lipid peroxidation. Biomedicine & Pharmacotherapy 2018, 100, 83-92. DOI: 10.1016/j.biopha.2018.01.172.

(750) Xiao, Y.; Dong, J.; Yin, Z.; Wu, Q.; Zhou, Y.; Zhou, X. Procyanidin B2 protects against D-galactose-induced mimetic aging in mice: Metabolites and microbiome analysis. Food and Chemical Toxicology 2018, 119, 141-149. DOI: 10.1016/j.fct.2018.05.017.

(751) Yang, Q.-N.; Deng, W.; Wu, D.-T.; Li, J.; Liu, H.-Y.; Yan, H.-L.; Du, K.; Hu, Y.-C.; Zou, L.; Huang, J.-W. Characterization, Antioxidant Capacity, and Anti-Inflammatory Activity of Polyphenol-Enriched Extracts Obtained from Unripe, Mature, and Overripe Fruits of Red-Fleshed Kiwifruit Cultivars. Foods 2024, 13 (18). DOI: 10.3390/foods13182860.

(752) Fatima, M.; Rizvi, S. I. Anti Oxidative Effect of Black Tea Theaflavin on Erythrocytes Subjected to Oxidative Stress. National Academy Science Letters-India 2015, 38 (1), 25-28. DOI: 10.1007/s40009-014-0285-9.

(753) Bhuyan, L. P.; Sabhapondit, S.; Baruah, B. D.; Bordoloi, C.; Gogoi, R.; Bhattacharyya, P. Polyphenolic compounds and antioxidant activity of CTC black tea of North-East India. Food Chemistry 2013, 141 (4), 3744-3751. DOI: 10.1016/j.foodchem.2013.06.086.

(754) Gao, Y.; Li, W.; Jia, L.; Li, B.; Chen, Y. C.; Tu, Y. Enhancement of (-)-epigallocatechin-3-gallate and theaflavin-3-3 '-digallate induced apoptosis by ascorbic acid in human lung adenocarcinoma SPC-A-1 cells and esophageal carcinoma Eca-109 cells via MAPK pathways. Biochemical and Biophysical Research Communications 2013, 438 (2), 370-374. DOI: 10.1016/j.bbrc.2013.07.078.

(755) Sang, S. M.; Tian, S. Y.; Jhoo, J. W.; Wang, H.; Stark, R. E.; Rosen, R. T.; Yang, C. S.; Ho, C. T. Chemical studies of the antioxidant mechanism of theaflavins: radical reaction products of theaflavin 3,3 '-digallate with hydrogen peroxide. Tetrahedron Letters 2003, 44 (30), 5583-5587. DOI: 10.1016/s0040-4039(03)01382-0.

(756) Blaszczak, W.; Amarowicz, R.; Gorecki, A. R. Antioxidant capacity, phenolic composition and microbial stability of aronia juice subjected to high hydrostatic pressure processing. Innovative Food Science & Emerging Technologies 2017, 39, 141-147. DOI: 10.1016/j.ifset.2016.12.005.

(757) Dugasani, S.; Pichika, M. R.; Nadarajah, V. D.; Balijepalli, M. K.; Tandra, S.; Korlakunta, J. N. Comparative antioxidant and anti-inflammatory effects of 6 -gingerol, 8 -gingerol, 10 -gingerol and 6 -shogaol. Journal of Ethnopharmacology 2010, 127 (2), 515-520. DOI: 10.1016/j.jep.2009.10.004.

(758) Ionov, M.; Gordiyenko, N. V.; Zukowska, I.; Tokhtaeva, E.; Mareninova, O. A.; Baram, N.; Ziyaev, K.; Rezhepov, K.; Zamaraeva, M. Stability and antioxidant activity of gossypol derivative immobilized on N-polyvinylpyrrolidone. International journal of biological macromolecules 2012, 51 (5), 908-914. DOI: 10.1016/j.ijbiomac.2012.08.005.

(759) Moser, B. R. Efficacy of gossypol as an antioxidant additive in biodiesel. Renewable Energy 2012, 40 (1), 65-70. DOI: 10.1016/j.renene.2011.09.022.

(760) Zhang, D.; Lu, C.; Yu, Z.; Wang, X.; Yan, L.; Zhang, J.; Li, H.; Wang, J.; Wen, A. Echinacoside Alleviates UVB Irradiation-Mediated Skin Damage via Inhibition of Oxidative Stress, DNA Damage, and Apoptosis. Oxidative Medicine and Cellular Longevity 2017. DOI: 10.1155/2017/6851464.

(761) Jia, C.; Shi, H.; Jin, W.; Zhang, K.; Jiang, Y.; Zhao, M.; Tu, P. Metabolism of Echinacoside, a Good Antioxidant, in Rats: Isolation and Identification of Its Biliary Metabolites. Drug Metabolism and Disposition 2009, 37 (2), 431-438. DOI: 10.1124/dmd.108.023697.

(762) Liu, X.; Pan, L.; Wang, X.; Gong, Q.; Zhu, Y. Z. Leonurine protects against tumor necrosis factor-alpha-mediated inflammation in human umbilical vein endothelial cells. Atherosclerosis 2012, 222 (1), 34-42. DOI: 10.1016/j.atherosclerosis.2011.04.027.

(763) Qi, J.; Hong, Z. Y.; Xin, H.; Zhu, Y. Z. Neuroprotective Effects of Leonurine on Ischemia/Reperfusion-Induced Mitochondrial Dysfunctions in Rat Cerebral Cortex. Biological & pharmaceutical bulletin 2010, 33 (12), 1958-1964. DOI: 10.1248/bpb.33.1958.

(764) Suga, A.; Takaishi, Y.; Goto, S.; Munakata, T.; Yamauchi, I.; Kogure, K. Two lignan dimers from bamboo stems (Phyllostachys edulis). Phytochemistry 2003, 64 (5), 991-996. DOI: 10.1016/50031-9422(03)00422-9.

(765) Lee, Y. J.; Jung, O.; Lee, J.; Son, J.; Cho, J. Y.; Ryou, C.; Lee, S. Y. Maclurin exerts anti-cancer effects on PC3 human prostate cancer cells via activation of p38 and inhibitions of JNK, FAK, AKT, and c-Myc signaling pathways. Nutrition research (New York, N.Y.) 2018, 58, 62-71. DOI: 10.1016/j.nutres.2018.07.003.

(766) Li, X.; Gao, Y.; Li, F.; Liang, A.; Xu, Z.; Bai, Y.; Mai, W.; Han, L.; Chen, D. Maclurin protects against hydroxyl radical-induced damages to mesenchymal stem cells: Antioxidant evaluation and mechanistic insight. Chemico-biological interactions 2014, 219, 221-228. DOI: 10.1016/j.cbi.2014.06.014.

(767) Kouam, S. F.; Ngadjui, B. T.; Krohn, K.; Wafo, P.; Ajaz, A.; Choudhary, M. I. Prenylated anthronold antioxidants from the stem bark of Harungana madagascariensis. Phytochemistry 2005, 66 (10), 1174-1179. DOI: 10.1016/j.phytochem.2005.03.022.

(768) Cakir, A.; Mavi, A.; Yildirim, A.; Duru, M. E.; Harmandar, H.; Kazaz, C. Isolation and characterization of antioxidant phenolic compounds from the aerial parts of Hypericum hyssopifolium L. by activity-guided fractionation. Journal of Ethnopharmacology 2003, 87 (1), 73-83. DOI: 10.1016/s0378-8741(03)00112-0.

(769) Conforti, F.; Statti, G. A.; Tundis, R.; Menichini, F.; Houghton, P. Antioxidant activity of methanolic extract of Hypericum triquetrifolium Turra aerial part. Fitoterapia 2002, 73 (6), 479-483. DOI: 10.1016/s0367-326x(02)00162-4.
