# Supplementary material for: Antioxidant Chemistry of Phenolics in Chinese Herbal Medicines: A Holism‐Based Review
Source: Chem Biodivers. 2026 Sep 20;23(9):e71716. doi: 10.1002/cbdv.71716 (PMC13590138; doi:10.1002/cbdv.71716)
Supplement: Supplementary file 2 [file CBDV-23-e71716-s002.pdf]

Note: To support the discussion of main text, this review performed a preliminary statistics of these Chinese and English articles was based on the “title/abstract” retrieval in respectively CNKI and Pubmed, spanning 40 years (1986-2026). The preliminary statistics involves in 36,000 Chinese research articles and 8,000 English research articles involving antioxidant properties of CHMs. Furthermore, the articles were optimized by manually. Articles without chemical structure were abodoned while those with chemical structure were reserved. The reserved articles were further screened, according to the phenolic structure and attribute of CHM. Finally, 769 available references have been colleded for the literature analysis (SI-1). Some of them are used for the discussion of main text while most of them (541 references) are collected in Table s1. It is found that Table s1 includes 477 phenolic antioxidants which are cross-distributed in 331 CHMs.

Table s1 The structure and cross-distribution of 477 phenolic antioxidants in 331 CHMs

No	Type	Subtype	Name	Structure	Cross-distribution in CHMs		
					Chinese name	English name	Plant
1	Volatile phytophenol, phenolic acid, and their glucoside	Volatile phytophenol and glucoside	[6]-Gingerol	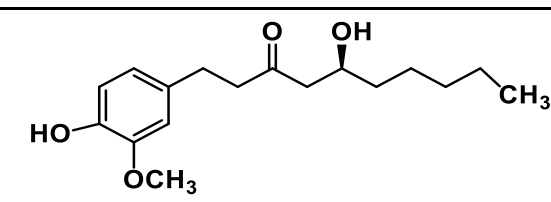	Shengjiang	*Zingiberis Rhizoma Recens*	*Zingiber officinale Rose* [1]
					Ganjiang	*Zingiberis Rhizoma*	*Zingiber officinale Rose.* [2]
			Isoeugenol	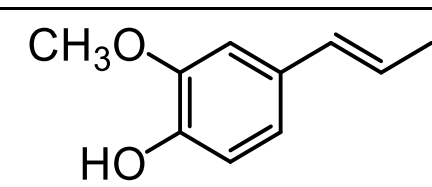	Roudoukou	*Semen Myristicae*	*Myristica fragrans Houtt* [3]
					Dingxiang	*Caryophylli Flos*	*Ewgewia caryophyllata Thunb.* [4]
			Eugenol	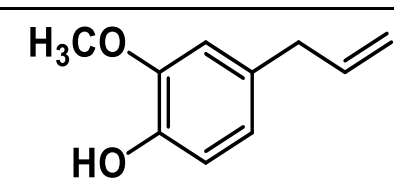	Dingxiang	*Caryophylli Flos*	*Eugenia caryophllata Thunb.* [5]
					Rougui	*Cinnamon*	*Cinnamomum verum J. Presl* [6]
			Magnolol	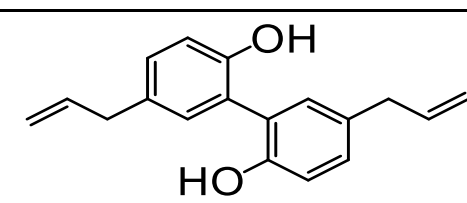	Houpo	*Magnoliae Officinalis Cortex*	*Magnoliae Officinalis Cortex*[7]
			Thymol	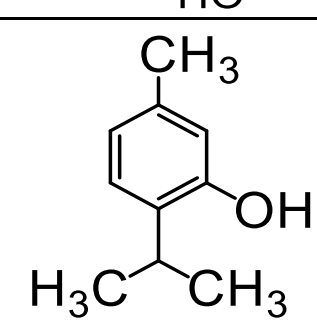	Bailixiang	*Thymus mongolicus Ronn*	*Thymus mongolicus Ronn*[8]
					Niuzhi	*Origanum vulgare* L	*Origanum vulgare L*[9]
			Carnosol		Shuweicaofen	*Salvia japonica Thunb.*	*Salvia japonica Thunb.* [10]

				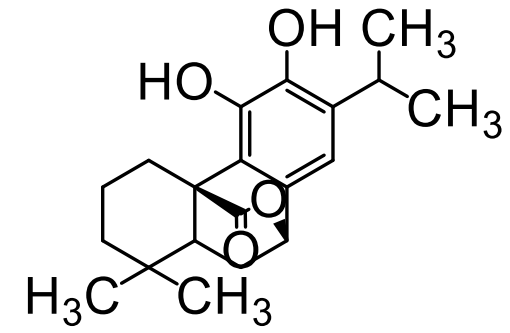	Midiexiang	*Rosmarinus officinalis*	*Rosmarinus officinalis* [11]
			Vanillin<br>Carvacrol	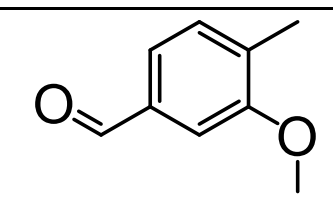<br>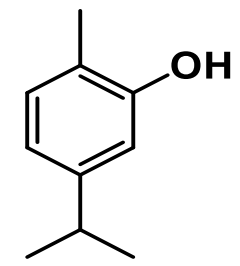	Xiangjialan	*Vanilla fragrans (Salisb.) Ames*	*Vanilla fragrans Salisb* [12]
					Shixiangru	*Mosla chinensis Maxim*	*Mosla chinensis Maxim* [13]
			Ferulic acid ethyl ester<br>Methylchavicol	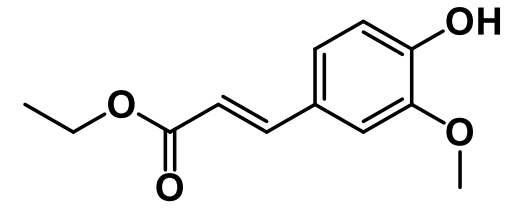<br>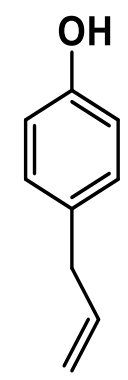	Chuangxiong	*Ligusticum chuanxiong hort*	*Ligusticum chuanxiong hort* [14]
			Ferulic acid ethyl ester<br>Methylchavicol<br>Ferulic acid ethyl ester	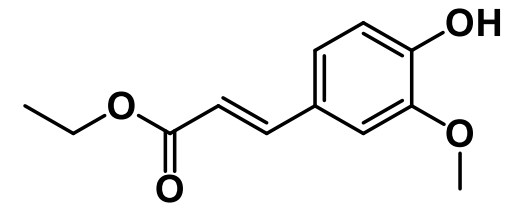<br>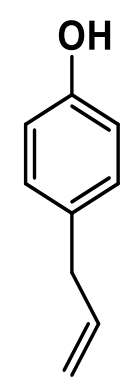	Bajiaohuixiang	*Lllicium verum*	*Lllicium verum hook. f* [15]
					Xinyi	*Magnolia kokus*	*Magnolia kokus DC* [16]

			4-methylcatechol<br>Albibrissinoside B	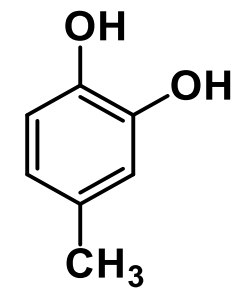<br>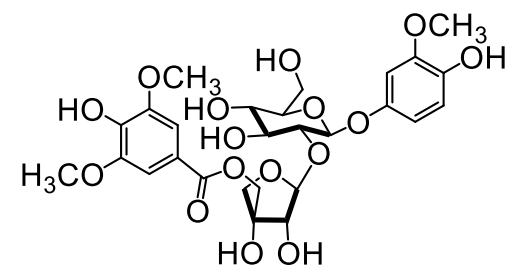	Pingguo	*Malus domestica*	*Malus domestica*[17]
			4-methylcatechol<br>Albibrissinoside B<br>Curculigoside	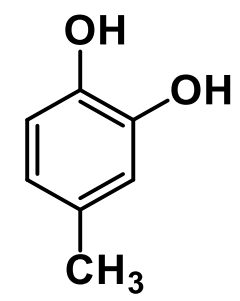<br>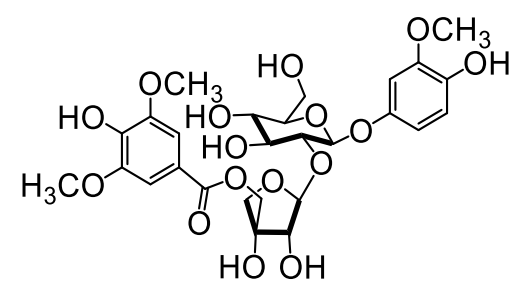<br>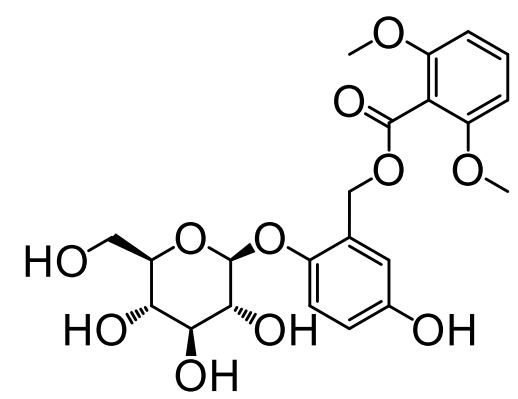	Hehuanpi	*Silktree Albizia Bark*	*Silktree Albizia Bark*[18]
					Xianmao	*Curculigo orchioides*	*Curculigo orchioides Gaertn*[19]
			Decaffeoylacteoside	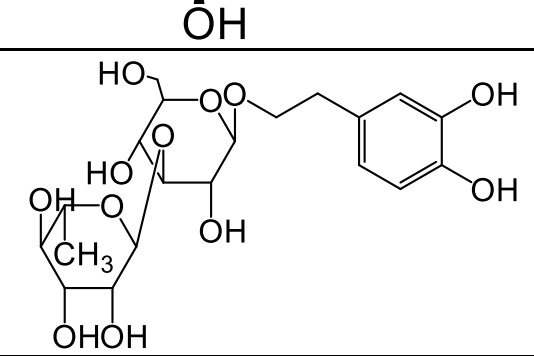	Xuanshen	*Scrophularia ningpoensis Hemsl*	*Scrophularia ningpoensis Hemsl*[20]
			Ethyl 3,4-Dihydroxybenzoate	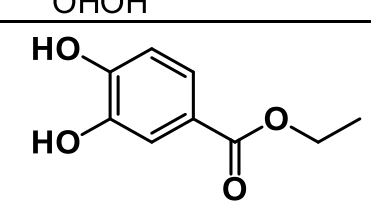	Xianrenzhang	*Opuntia stricta*	*Opuntia stricta (Haw.) Haw. var. dilleniid (Ker-Gawl.) Benson* [21]

			P-Hydroxybenzoic acid	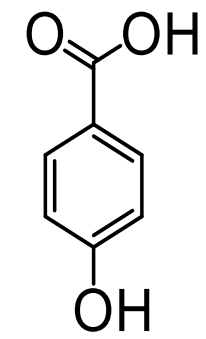	Banbianlian	*Lobelia chinensis Lour.*	*Lobelia chinensis Lour* [22]
			Ligurobustoside N	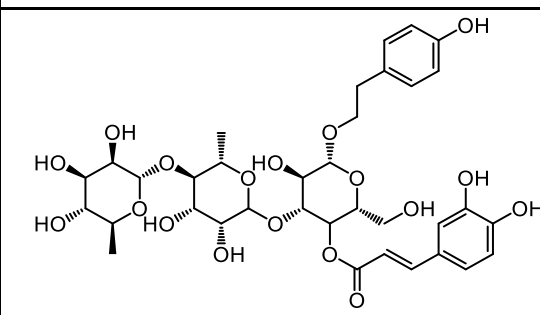	Cuzhuangnvzhen	*Ligustrum robustum (Roxburgh) Blume*	*Ligustrum robustum*[23]
			Orcinol glucoside	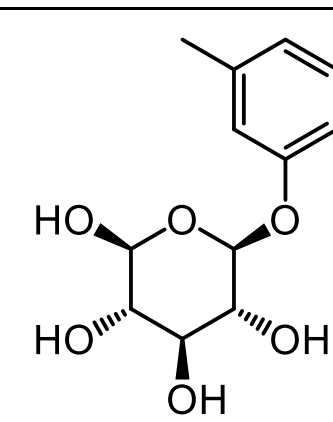	Xianmao	*Curculigo orchioides Gaertn.*	*Curculigo orchioides Gaertn.* [24]
			Paeonol	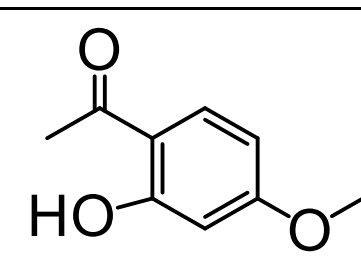	Mudanpi	*Paeonia moutan Sim.*	*Paeonia moutan Sim.* [25]
			Scroside A	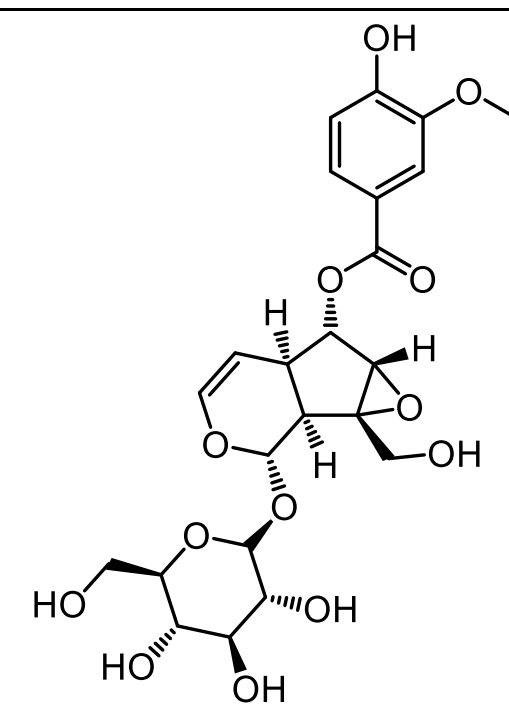	Huhuanglian	*Picrorhiza scrophulariiflora*	*Picrorhiza scrophulariiflora Pennell* [26]
			Salvianic aid A	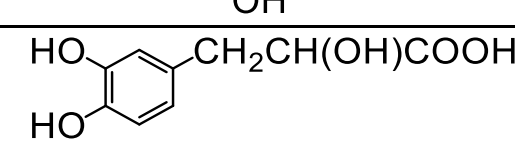	Danshen	*Salvia miltiorrhiza Bge.*	*Salvia miltiorrhiza Bge.* [27]

			Perrottetin D	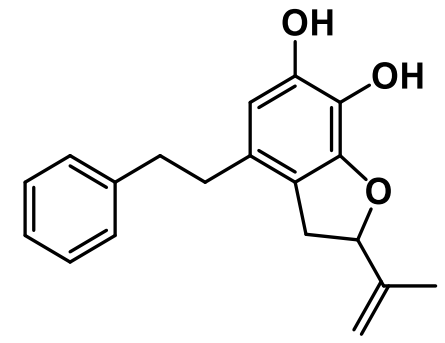	Ningbianetai	*Radula Perrottetii*	*Radula complanata (linn.) dum.* [28]
			Caffeic acid	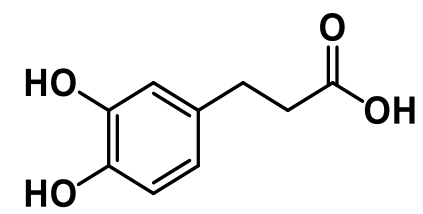	Pugongying	*Taraxaci Herba*	*TaraxacMm mongolicum Hand.-Mazz.* [29]
			Perrottetin D	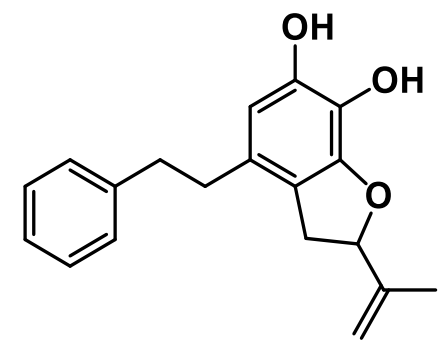	Shengma	*Cimicifugae Rhizoma*[30]	*Cimicifuga foetida L.*
			Ferulic acid	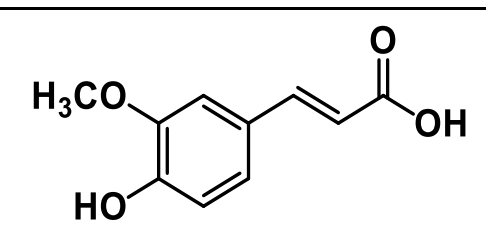	Danggui	Radix Angelicae Sinensisi[31]	*Angelica sinensis (Oliv.) Diels*
		Phenolic acid and glucoside	Ferulic acid<br>Isoferulic acid [32]	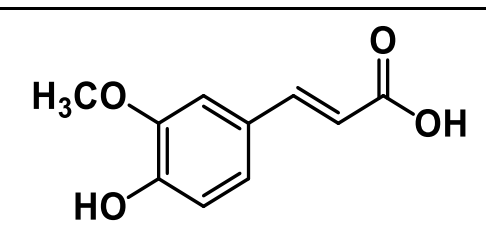<br>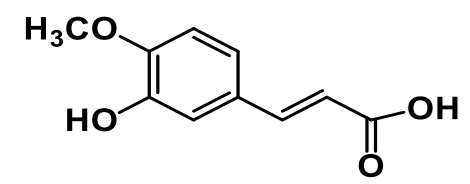	Shengma	*Cimicifugae Rhizoma*[31]	*Cimicifuga foetida L .*
					Shengma	*Cimicifugae Rhizoma* [31]	*Cimicifuga foetida L .*
			Isoferulic acid [32]<br>Chlorogenic acid	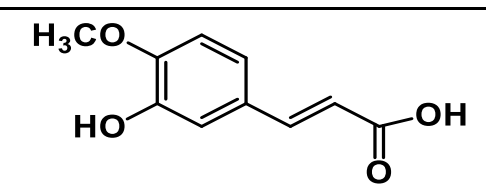<br>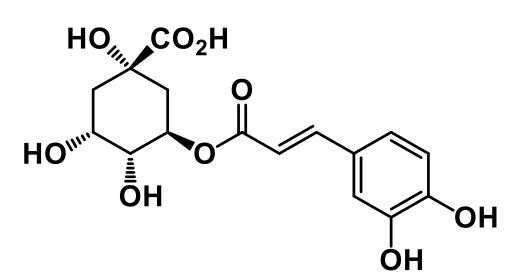	Banbianlian	*Lobelia chinensis Herba*[33]	*Lobelia chinensis Lour.*
					Juhua	*Flos Chrysanthemi Indici*[34]	*Chrysanthemum morifolium Ramat.*
			Chlorogenic acid<br>Chlorogenic acid		Lianqiao	*Forsythiae Fructus*[35]	*Forsythia suspensa (Thunb.) Vahl*

			Protocatechuic acid	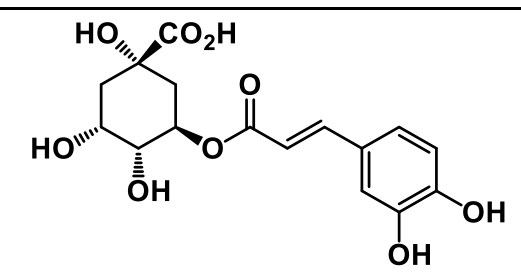 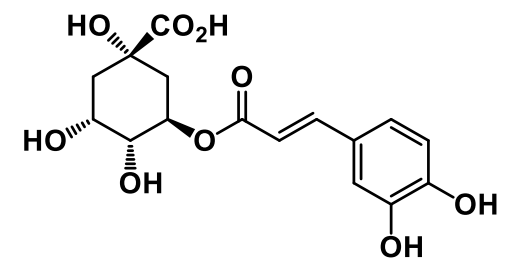 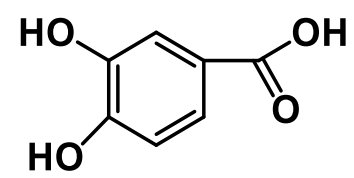	Sangshen	*Mori Fructus*[36]	*Morus alba L.*
			Chlorogenic acid<br>Protocatechuic acid<br>Gallic acid	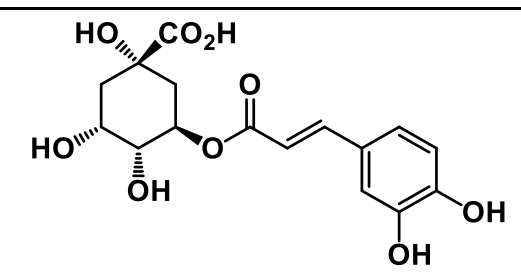 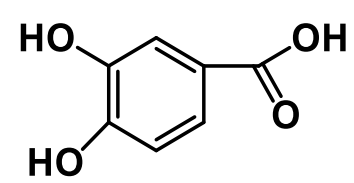 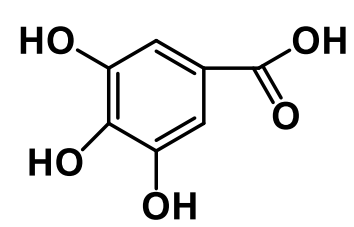	Touhualiao	*Polygonum Capitatum*[37]	*Polygonum capitatum Buch.-Ham. ex D. Don*
					Gouji	*Cibotii Rhizoma*	*Cibcnium barometz(L.) J .Sm*[38]
					Wuweizi	*Schisandrae Chinensis Fructus* [39, 40]	*Schisandra chinensis(Turcz.) Baill*
			Gallic acid<br><br>Sinapic acid	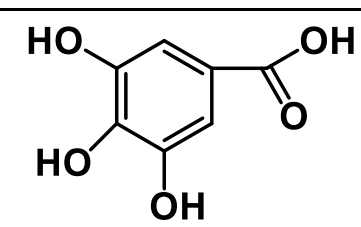 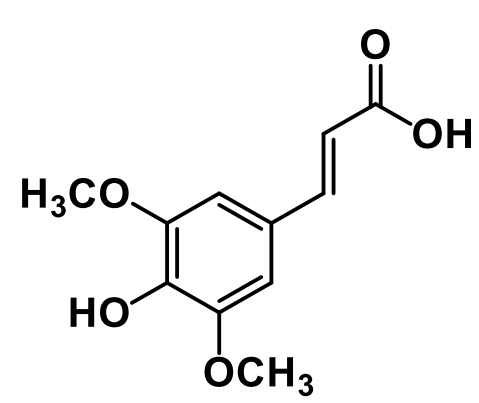	Suoyang [41]	*Cynomorii Herba*	*Cynomorium songaricum Rupr.*
					Huangjiezi	*Brassica juncea(L.)Czern. et Coss.*	*Brassica juncea(L.)Czern. et Coss.* [42]
			Sinapic acid		Baijianzi	*Semen sinapis*	*Sinapis alba L*[43]

			p-coumalic acid<br>vanillic acid	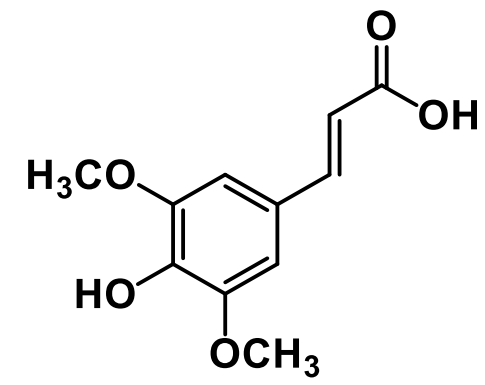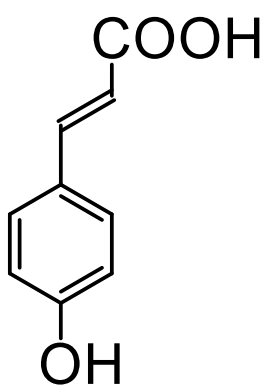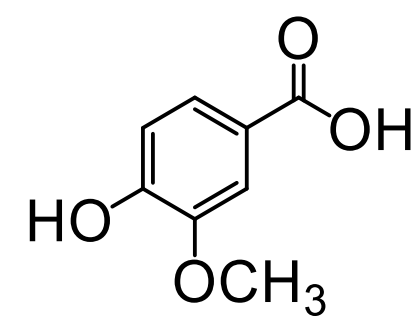	Baihuasheshecao	*Oldenlandia diffusa （Willd.）Roxb*	*Oldenlandia diffusa Willd.* [44]
			p-coumalic acid<br>vanillic acid<br>p-coumalic acid	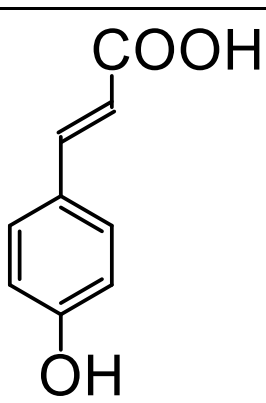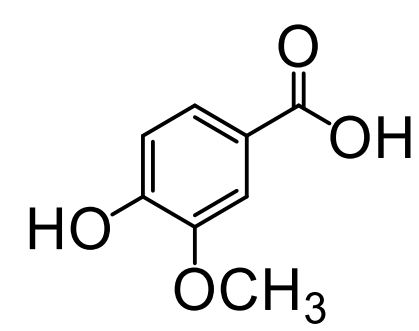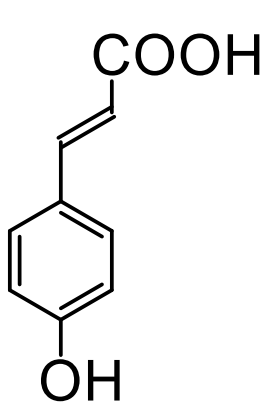	Chuanlianzi	*MeLia toosendan Sieb.et Zucc.*	*MeLia toosendan Sieb.et Zucc.* [45]
					Xiangzilan	*Vanilla fragrans*	*Vanilla fragrans* [46,47]

			homovanillic acid<br>Cynarine	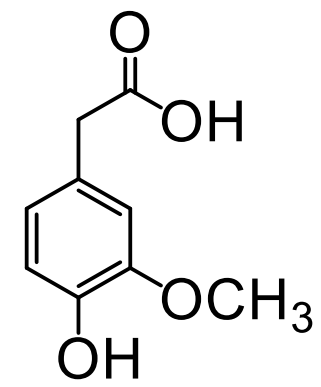<br>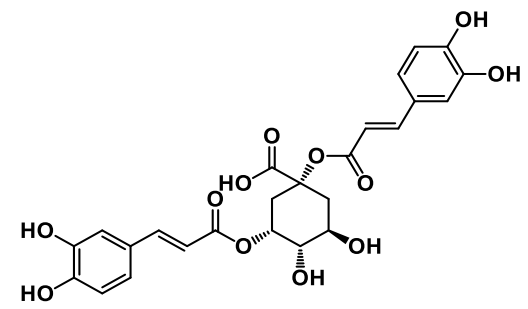	Shizhu	*Dianthus chinensis*	*Dianthus chinensis* L[48]
			homovanillic acid<br>Cynarine<br>Cinnamic acid	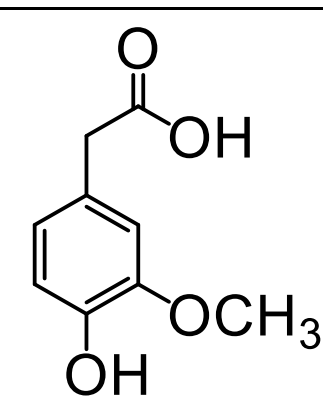<br>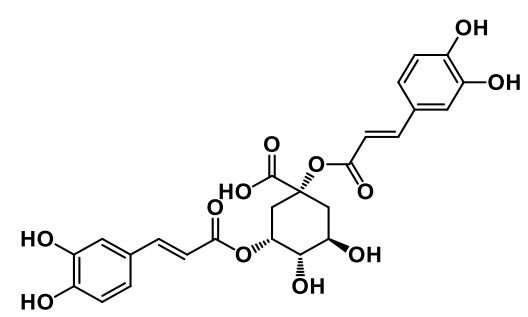<br>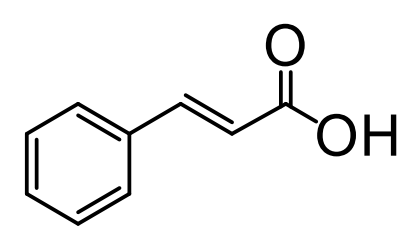	Yangji	*Cynara scolymus*	*Cynara scolymus* L[49]
					Rouguipi	*Cinnamomum cassia Presl*	*Cinnamomum cassia* Presl [50]
			Cynarine	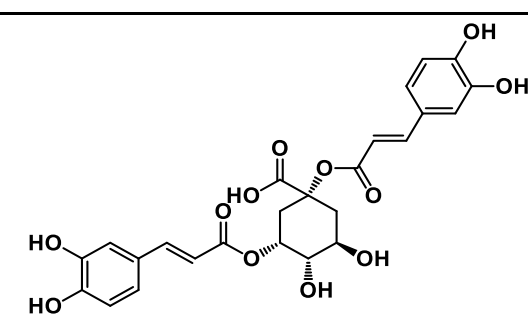	Anxixiang	*Benzoin*	*Styrax formosanus*[51]
			Syringc acid<br>Ellagic acid	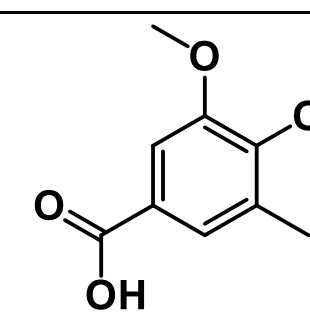	Banlangen	*Isatis indigotica Fortune*	*Isatis indigotica Fortune* [52]

				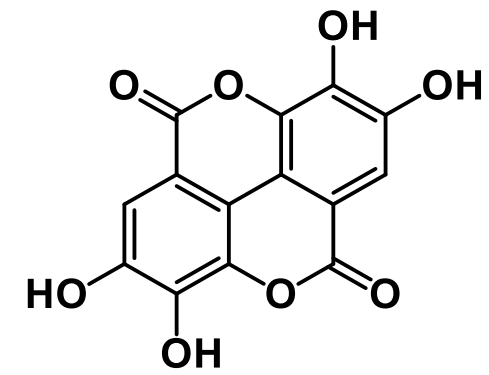			
			Syringc acid<br>Ellagic acid<br>Resveratrol	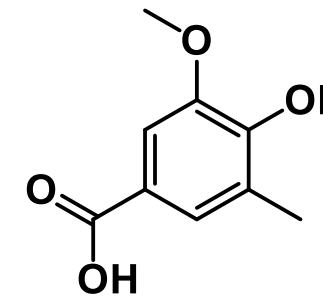<br>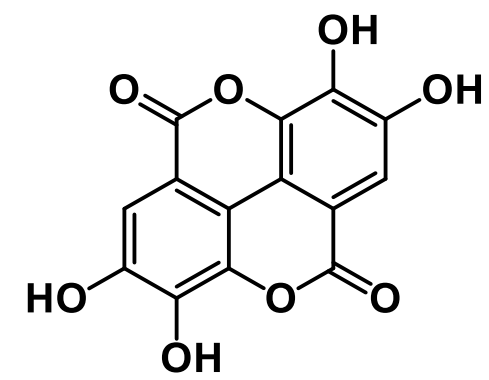<br>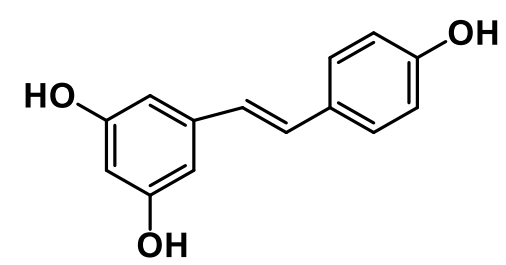	Hutao<br>Huzhang	*Juglans regia L*<br>*Polygoni Cuspidati Rhizoma et Radix*	*Juglans regia L*[53]<br>*Polygonum cuspidatum* [54]
			Ellagic acid	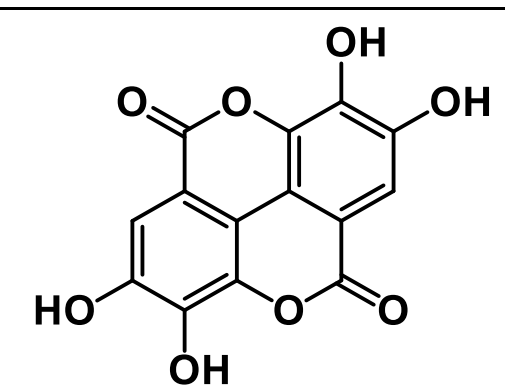	Sangshen	*Mori Fructus*[55]	*Morus alba L.*[56]
			Resveratrol<br>Cassigarol E<br>Cassigarol G	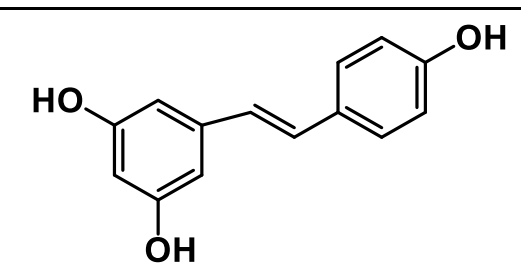	Tufuling	*Smilacis Glabrae Rhizoma*	*Smilax glabra Roxb.* [57]

				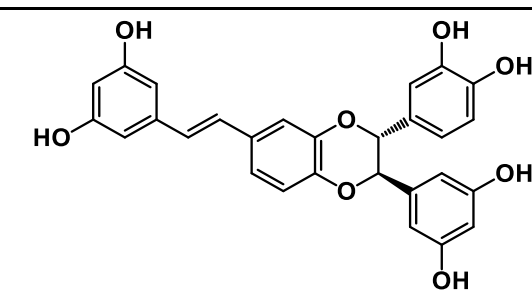 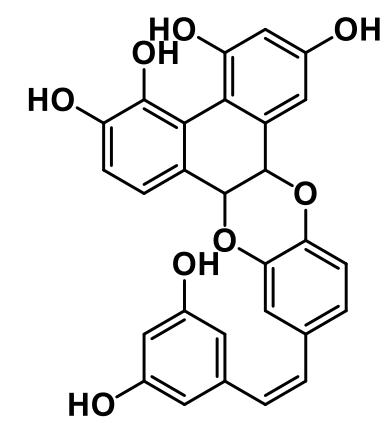			
2	Stilbenes	Monostilbene	Resveratrol<br>Cassigarol E<br>Cassigarol G<br>Isorhapontigenin	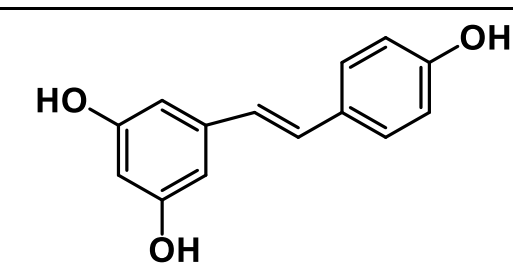 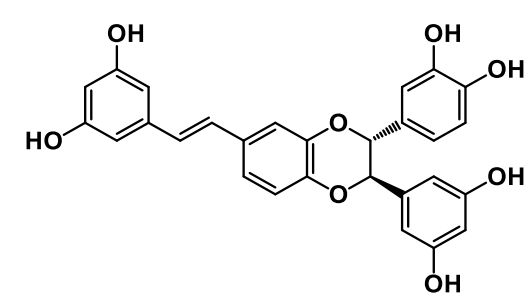 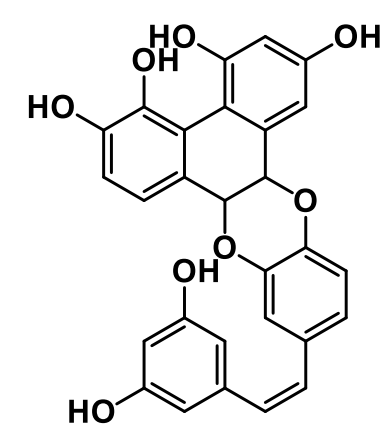 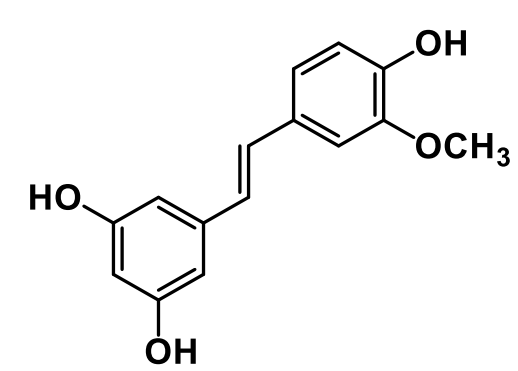	Zangjinjier	*Caragana tibetica*	*Caragana tibetica*[58]
					Maocijinjier	*Caragana tibetica*	*Caragana tibetica*[59]
					Xiaoyemaimateng	*Gnetum parvifolium*	*Gnetum parvifolium (Warb.) C. Y. Cheng ex Chun*[60]

			Isosalvianolic acid C	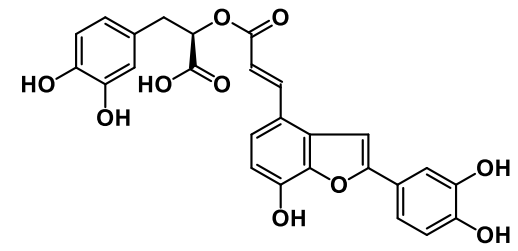	Shijianchuang	*Salvia chinensis Benth*	*Salvia chinensis* Benth[61]
			Piceatannol	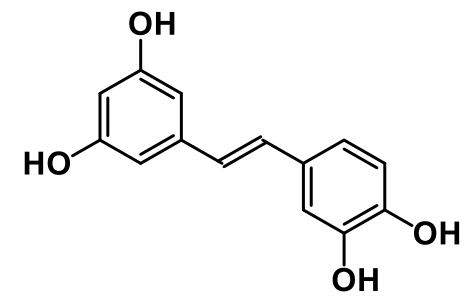	Lanmei	*Blueberry*	*Semen Trigonellae* [62]
			Erianin	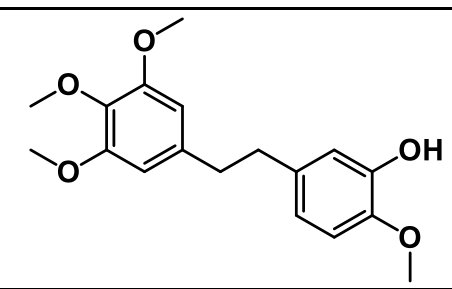	Shihu	*Dendrobium loddigesii Rolfe*	*Dendrobium loddigesii* Rolfe [63]
			Salvianolic acid A	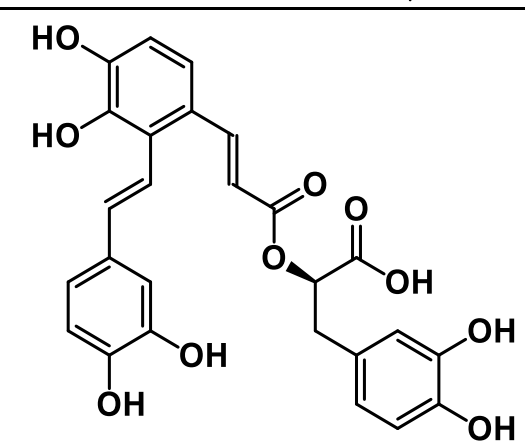	Danshen	Salvia miltiorrhiza Bunge	*Salvia miltiorrhiza* Bunge [64]
			Salvianolic acid F	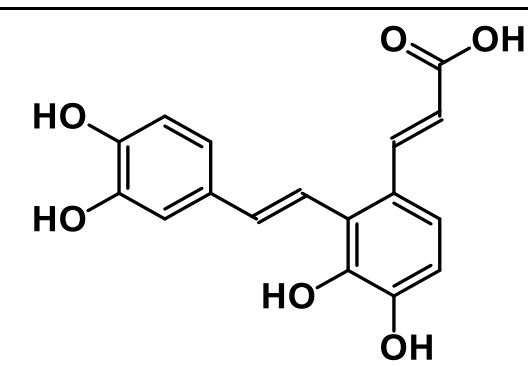	Zidanteng	*Tournefortia sarmentosa*	*Tournefortia sarmentosa Lam.* [65]
			Scirpusin A	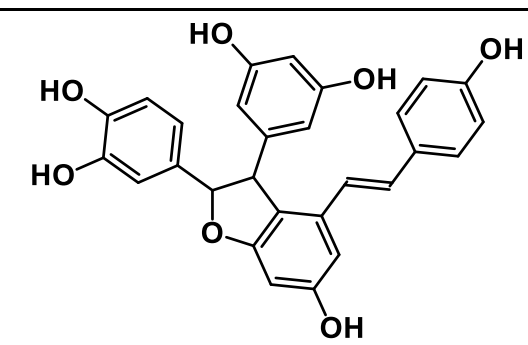	Maocijinjier	*Caragana tibetica*	*Caragana tibetica*[66]

			Scirpusin B	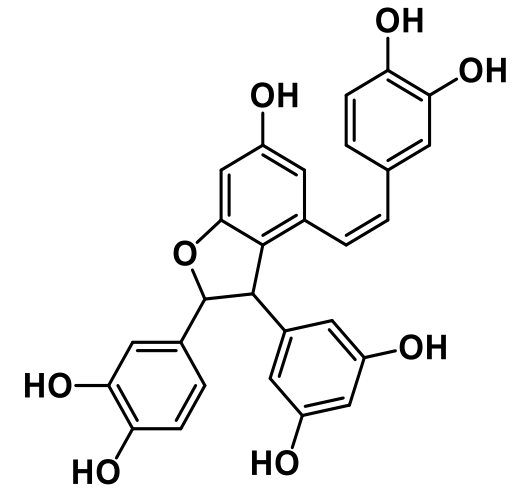	Maocijinjier	*Caragana tibetica*	*Caragana tibetica*[67]
			Tibeticanol	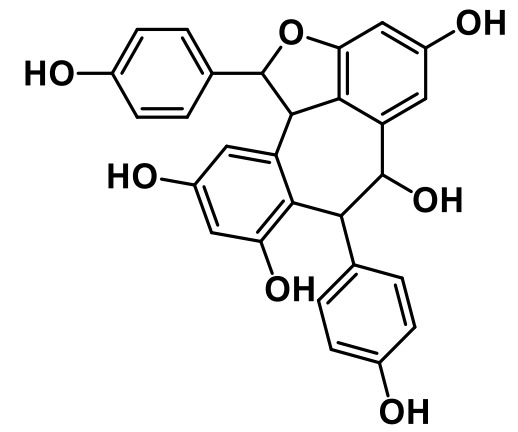	Maocijinjier	*Caragana tibetica*	*Caragana tibetica*[59]
			Tournefolic acid B	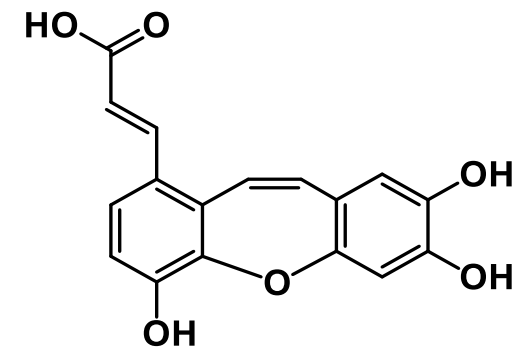	Zidanteng	*Tournefortia sarmentosa*	*Tournefortia sarmentosa* [61]
			Tournefolic acid B ethyl ester	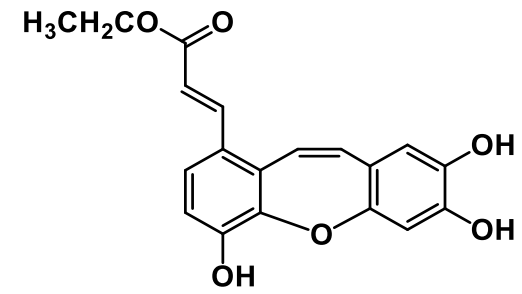	Zidanteng	*Tournefortia sarmentosa*	*Tournefortia sarmentosa* [68]
			Ugonstilbene A	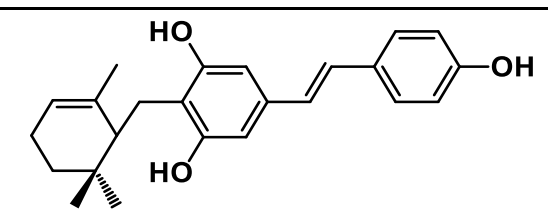	Rudiwugong	*Helminthostachys zeylanica*	*Helminthostachys zeylanica*[69]
			Ugonstilbene B	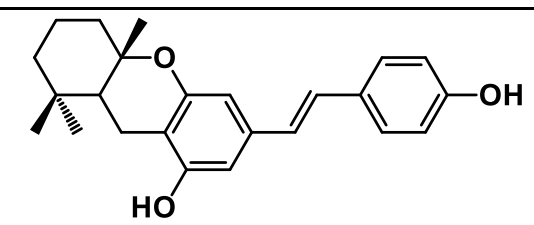	Rudiwugong	*Helminthostachys zeylanica*	*Helminthostachys zeylanica*[69]

			Ugonstilbene C	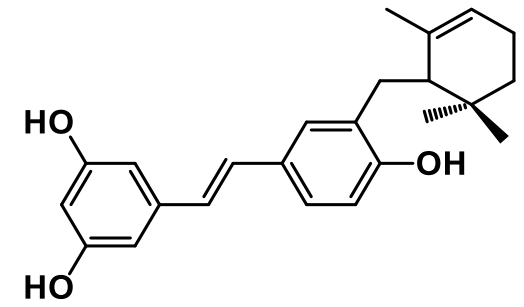	Rudiwugong	*Helminthostachys zeylanica*	*Helminthostachys zeylanica*[69]
			Rhapontigenin	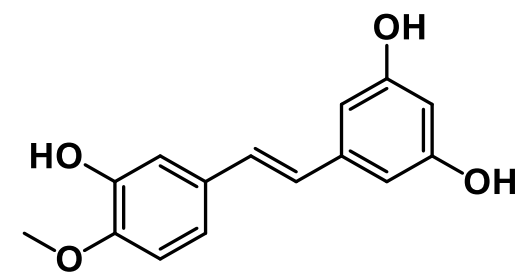	Dahuang	*Rhizome rhubarb*	*Rheum palmatum L*[70]
			4'-Methoxyresveratrol	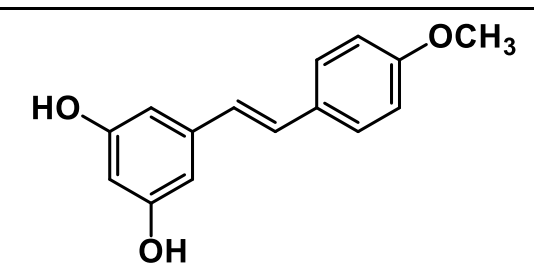	Dahuang	*Rhizome rhubarb*	*Rheum palmatum L*[71]
			Platyphyllol	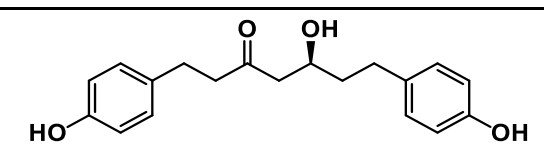	Baiqianceng	*Punktree*	*Melaleuca cajuputi subsp. cumingiana* [72]
			Gnetuhainin S	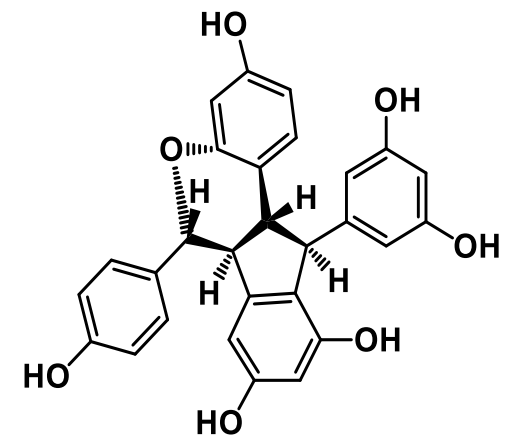	Maimateng	*Gnetum hainanense*[73]	*Gnetum montanum Markgr.*
			Gnemonol C	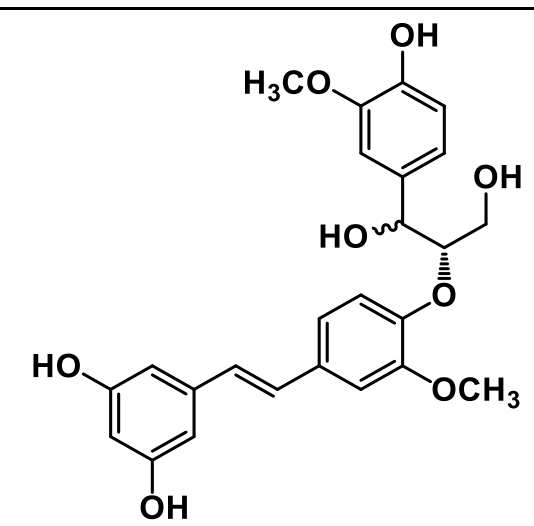	Xianzhoumaimateng	*Gnetum gnemon*	*Gnetum gnemon* [74]

			Gnetucleistol F	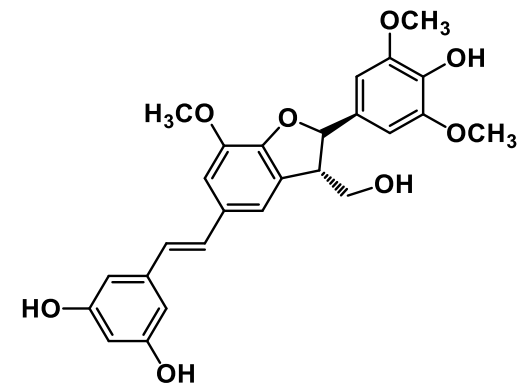	Xianzhoumaimateng	*Gnetum gnemon*	*Gnetum gnemon*[75]
		Oligostilbene	Gnemonol E	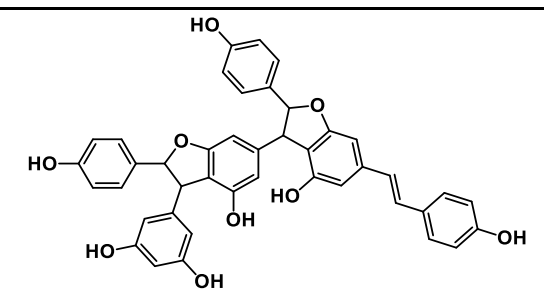	Xianzhoumaimateng	*Gnetum gnemon*	*Gnetum gnemon*[74]
			Kuwanon X	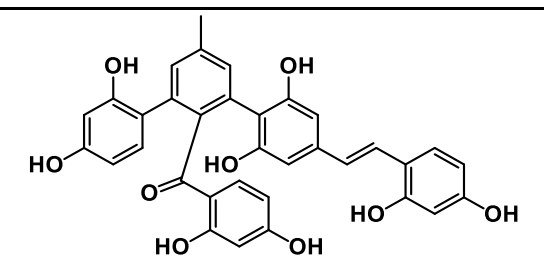	Naisang	*Morus macroura*	*Morus macroura*[76]
			α-viniferin	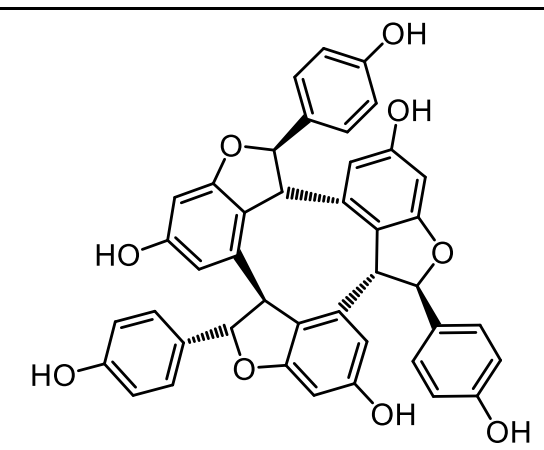	Putao	*Grape*	*Vitis vinifera L.* [77,56]
			Trans-ε-viniferin	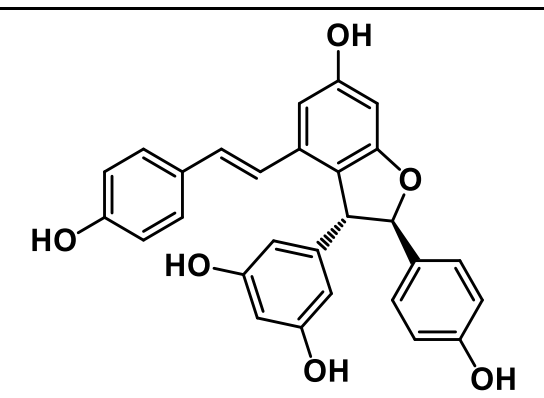	Qingmei	*Vatica mangachapoi Blanco*	*Vatica mangachapoi Blanco* [78]
			Cis-ε-viniferin	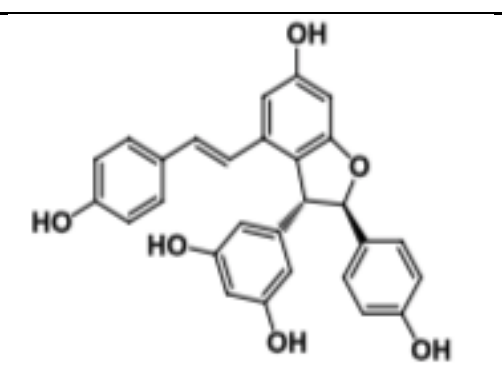	Shanputao	Vitis amurensis	Vitis amurensis[66, 79]

			Maximal A	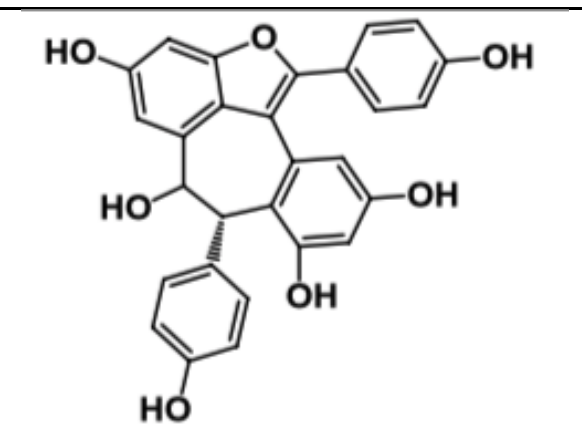	Malibatuoshu	Hopea malibato	Hopea malibato [80]
			Maximal B	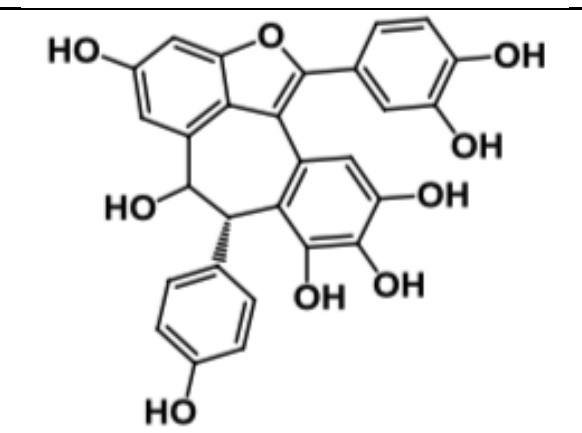	Malibatuoshu	Hopea malibato	Hopea malibato [81]
			Caraphenol A	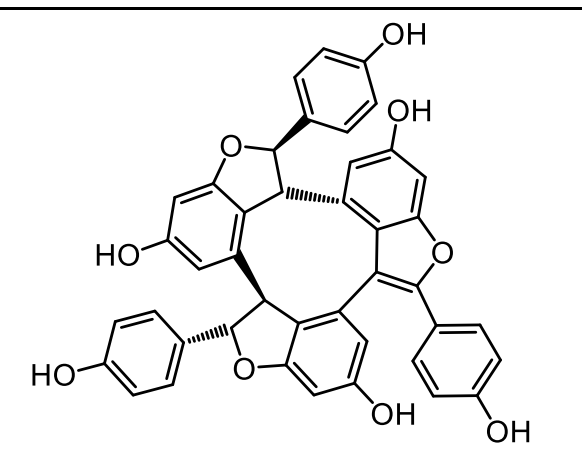	Jinquegen	*Caragana sinica Rehd*	*Caragana sinica Rehd*[82, 83]
			Diptoindonesin D	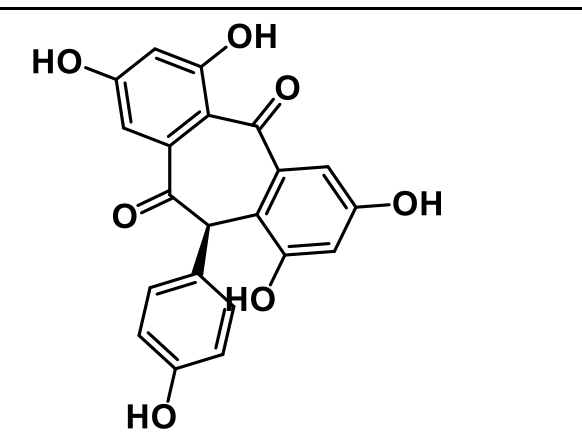	Zhougaijiazhi	*Amauroderma*	*Amauroderma* [84]
			Quadrangularin A	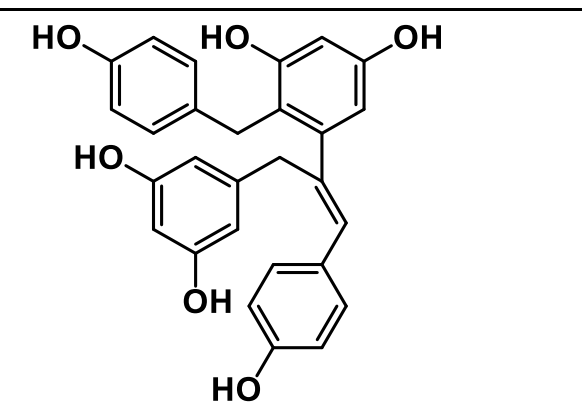	Sanyedijin	*parthenocissns tricuspidata*	*parthenocissns tricuspidata* [85]

			Pallidol	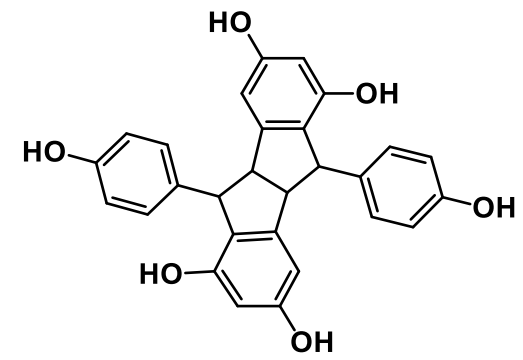	Lvyedijin	*Parthenocissus laetevirens*	*Parthenocissus laetevirens* [86]
			Hopeahainol D	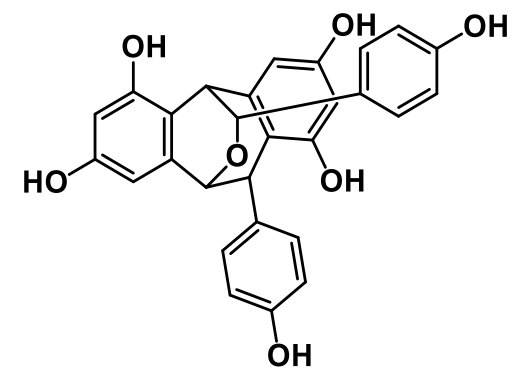	Polei	*Hopea hainanensis Merr. et Chun*	*Hopea hainanensis Merr. et Chun* [87]
			Hopeafuran	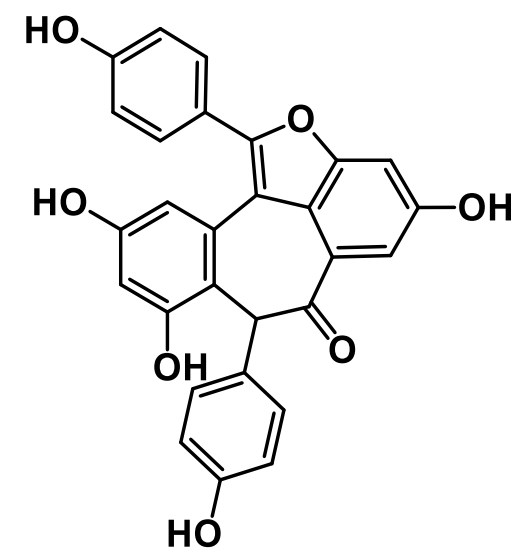	Pijiuhua	*Hopea utilis*	*Hopea utilis* [87]
			Rhaponticin	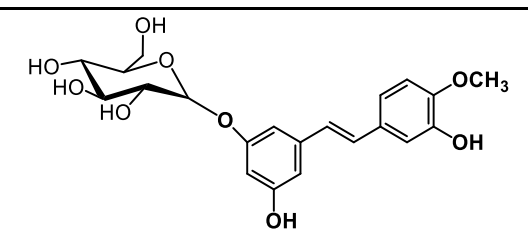	Dahuang	*Rhizome rhubarb*	*Rheum palmatum L.* [88]
			isorhapontigenin-11-O-β-D-glucopyranoside	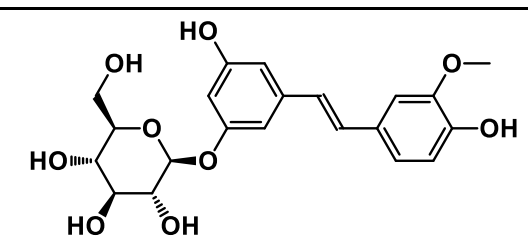	Xiaoyemaimateng	*Gnetum parvifolium*	*Gnetum parvifolium* [89]
			Piceatannol-3'-O-β-D-glucopyranoside	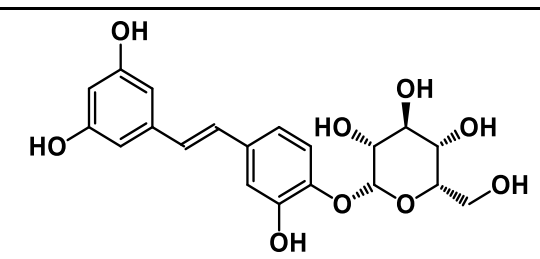	Baipishan	*Astringin*	*Picea aurantiaca Mast.* [90]

		Stilbene glucoside	Polydatin	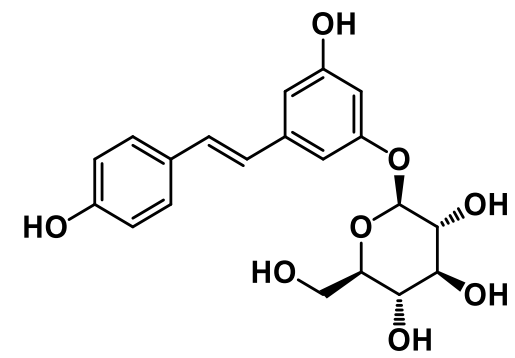	Huzhang	*Rhizoma Polygoni Cuspidati*	*Rhizoma Polygoni Cuspidati*[91]
			Piceatannol-3'-O-β-D-glucopyranoside	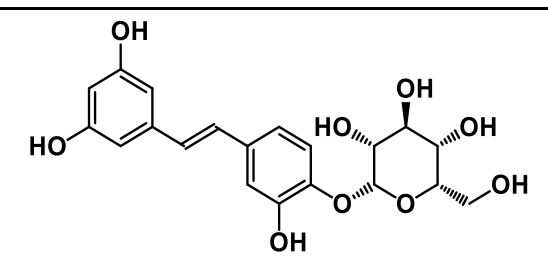	Anshu	*Eucalyptus robusta Smith*	*Eucalyptus robusta Smith*[92]
			2,3,5,4'-Tetrahydroxystilbene-2-O-D-glucoside 2,3,5,4’-tetrahydroxystilbene-2-O-β-D-glucopyrano-side-3″-monogalloyl ester	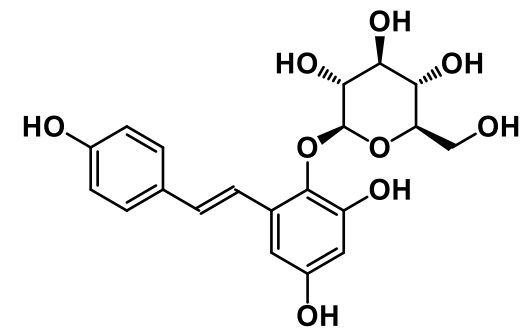 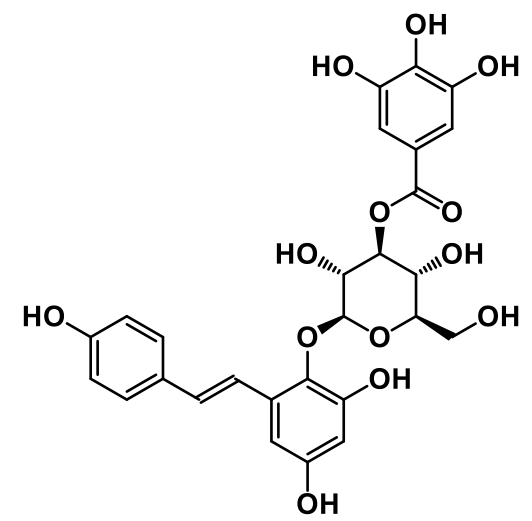	Heshouwu	*Fallopia multiflora (Thunb.) Harald*	*Fallopia multiflora (Thunb.) Harald*[93]
			2,3,5,4'-Tetrahydroxystilbene-2-O-D-glucoside 2,3,5,4’-tetrahydroxystilbene-2-O-β-D-glucopyrano-side-3″-monogalloyl ester Pieceid-2"-O-gallate	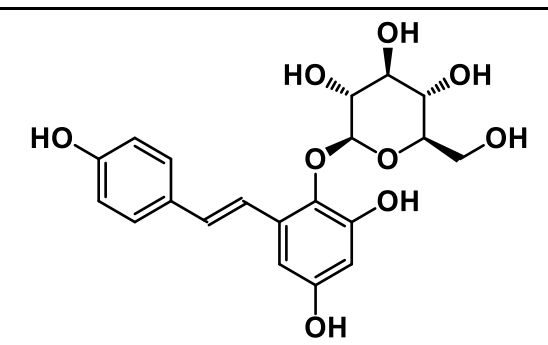	Heshouwu	*Fallopia multiflora (Thunb.) Harald*	*Fallopia multiflora (Thunb.) Harald* [94]
					Maomailiao	*Fallopia multiflora var. ciliinervis*	*Fallopia multiflora var. ciliinervis*[95]

				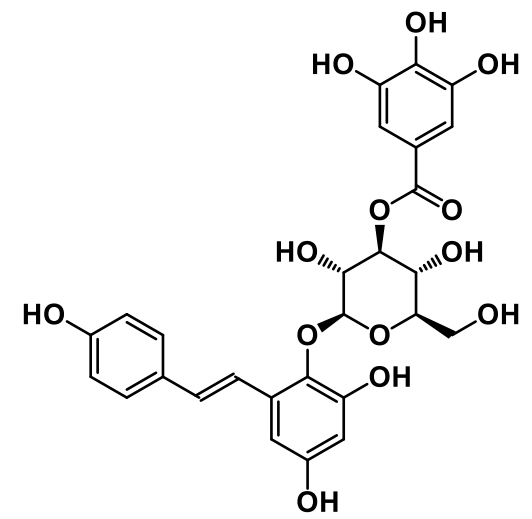 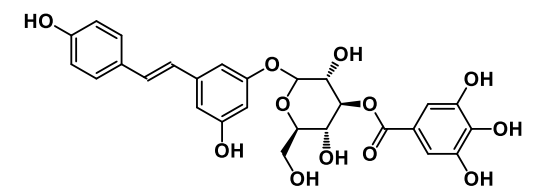			
			Pieceid-2"-O-coumarate	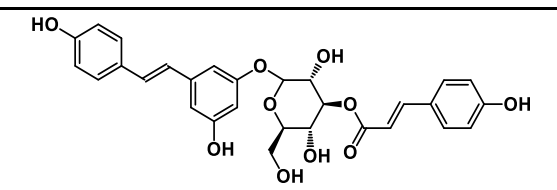	Maomailiao	*Fallopia multiflora var. ciliinervis*	*Fallopia multiflora var. ciliinervis*[95]
			Platyphylloside	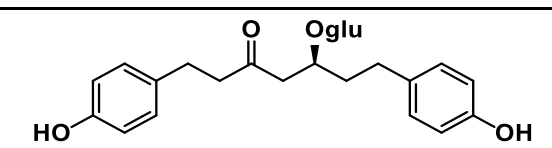	Baihualin	*Betula Pendula*	*Betula Pendula var* [96]
			Platyphyllonol-5-O- β -D-xylopyranoside	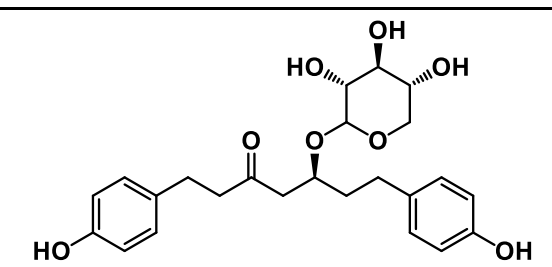	Maochiyang	*Alnus hirsuta*	*Alnus hirsuta var*[97]
			albafurans B	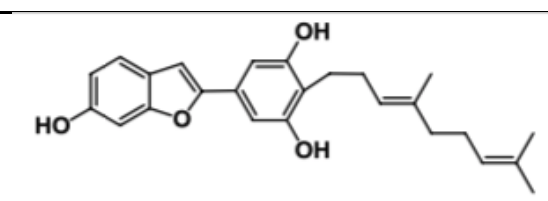	Heisangpi	Dry bark of black mulberry	Morus nigra L. .[98, 99]
			Isobavachalcone	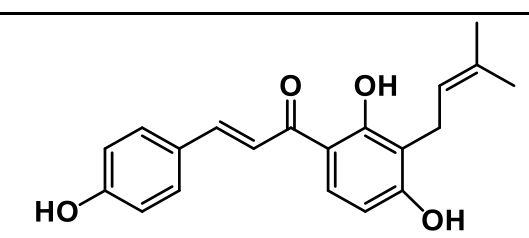	Buguzhi	*Fructus Psoraleae*	*Posoralea corylifolia L.* [100]

			Kuraridin	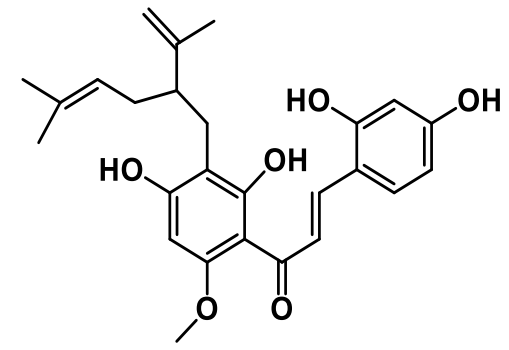	Kusen	*Sophorae Flavescentis Radix*	*Sophora flavescens Ait.* [101]
			Kuraridinol	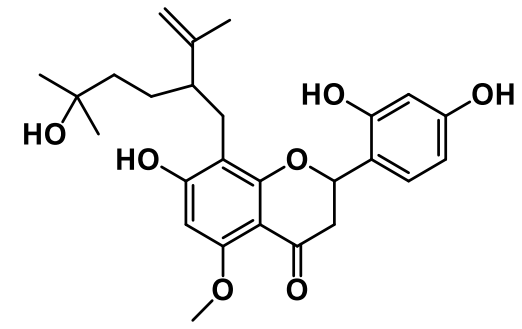	Kusen	*Sophorae Flavescentis Radix*	*Sophora flavescens Ait.* [101]
3	Chalcones	Chalcone	Isoliquiritigenin	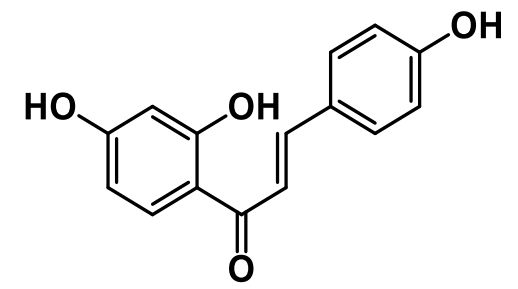	Gancao	*Licorice*	*Glycyrrhiza uralensis Fisch* [102-104]
			Isoliquiritin	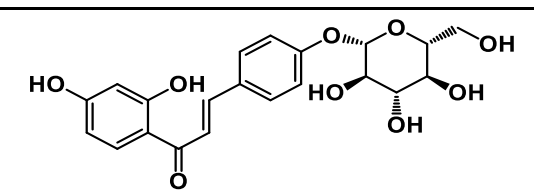	Gancao	*Licorice*	*Glycyrrhiza uralensis Fisch* [102, 105]
			Glycyglabrone	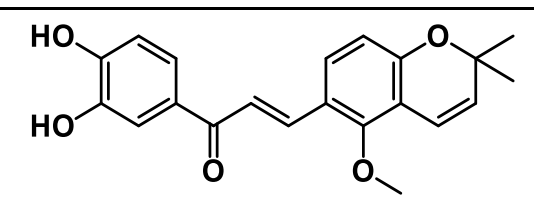	Gancao	*Licorice*	*Glycyrrhiza glabra L.* [106]
			Licoagrochalcone B	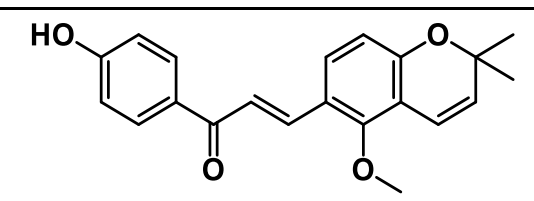	Gancao	*Licorice*	*Glycyrrhiza glabra L.* [106]
			Butein	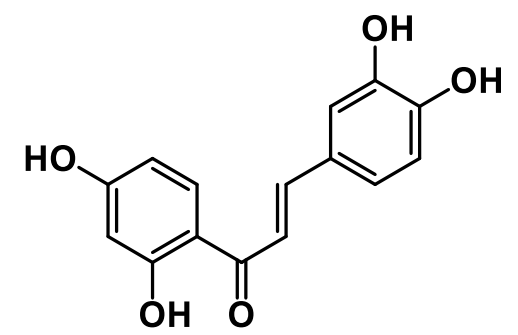	Jiangxiang	*Lignum Dalbergiae Odoriferae*	*Dalbergia odorifera T. Chen*[107]
			5-(1,1-dimethylallyl)-3,4,4'-trihydroxy-2-methoxychalcone	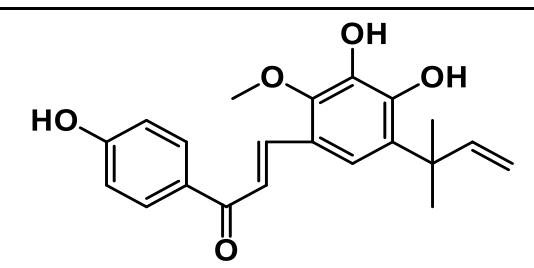	Gancao	*Licorice*	*Glycyrrhiza uralensis Fisch*[108]

			Echinatin	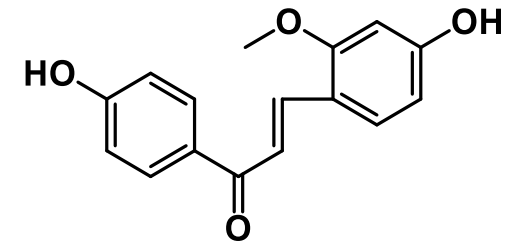	Gancao	*Licorice*	*Glycyrrhiza uralensis Fisch*[109]
			Licochalcone A	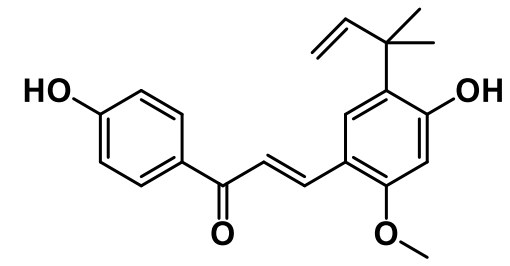	Gancao	*Licorice*	*Glycyrrhiza uralensis Fisch*[110]
			Licochalcone B	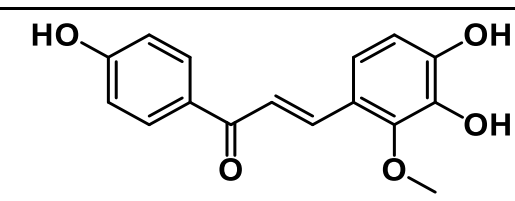	Gancao	*Licorice*	*Glycyrrhiza uralensis Fisch*[111]
			Licochalcone C	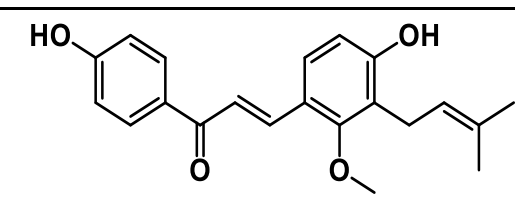	Gancao	*Licorice*	*Glycyrrhiza uralensis Fisch*[106, 112]
			Licochalcone D	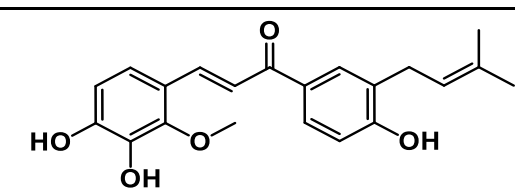	Gancao	*Licorice*	*Glycyrrhiza uralensis Fisch*[113]
			Licochalcone E	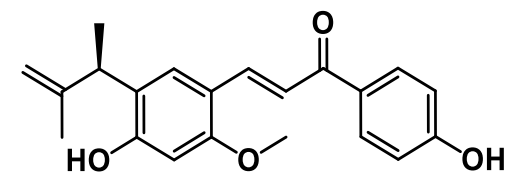	Gancao	*Licorice*	*Glycyrrhiza uralensis Fisch*[114]
			4-Hydroxylonchocarpin	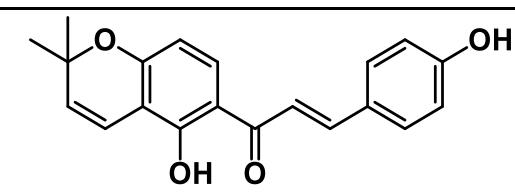	Dianlongdan	*Lonchocarpus eriocalyx*	*Gentiana rigescens Franch. ex Hemsl.* [115, 116]
			Bavachalcone	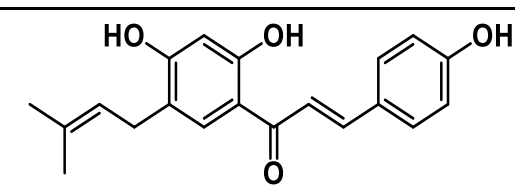	Buguzhi	*Psoralen*	*Psoralea corylifolia Linn.* [117]
			Naringenin chalcone	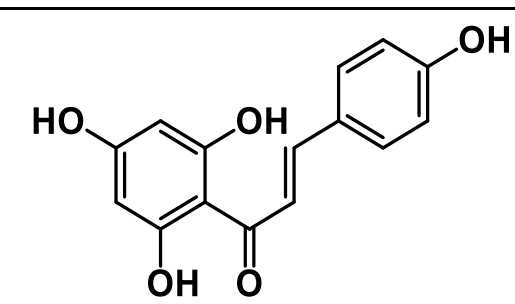	Zhishi	*Citrus aurantium L.*	*Citrus aurantium L.* [118]
			4'-O-[-D-glucopyranosyl-(1→6)-glucopyranosyl]- oxy-2'-hydroxy-3',6'-	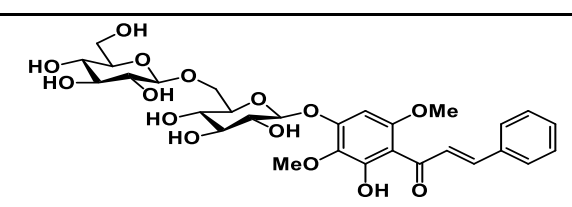	Mianmaosuanmoyeliao	*Woolly Dock Leaf*	*Polygonum lapathifolium var. salicifolium*[119]

			Dimethoxydihydrochalcone				
			4’-O- -D-(2’’-p-coumaroyl)glucopyranosyl-4,2’,3’-trihydroxychalcone	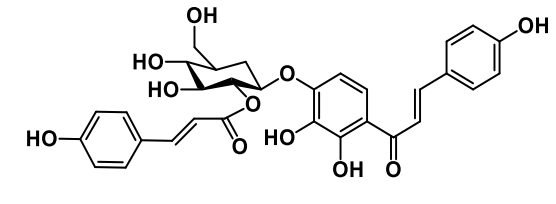	Banmao	*Maclura tinctoria*	*Saccharum arundinaceum Retz.* [120]
			4’-O- -D-(2’’-p-coumaroyl-6’’-acetyl)glucopyranosyl-4,2’,3’-trihydroxychalcone	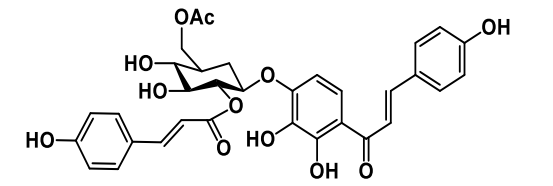	Banmao	*Maclura tinctoria*	*Saccharum arundinaceum Retz.* [120]
			3’-(3-methyl-2-butenyl)-4’-O- -D-glucopyranosyl-4,2’-dihydroxychalcone	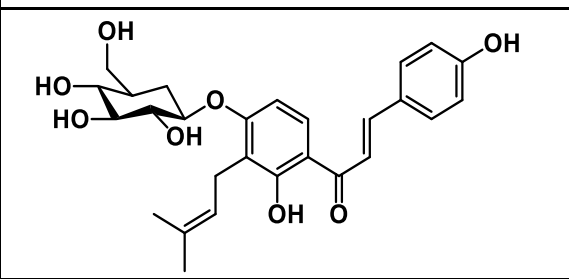	Banmao	*Maclura tinctoria*	*Saccharum arundinaceum Retz.* [120]
			4’-O-D-(2’’-acetyl-6’’-cinnamoyl)glucopyranosyl-4,2’,3’-trihydroxychalcone	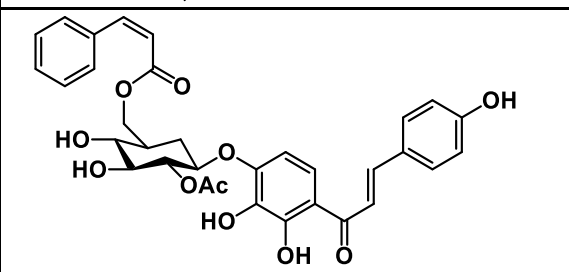	Banmao	*Maclura tinctoria*	*Saccharum arundinaceum Retz.* [120]
			Broussochalcone A	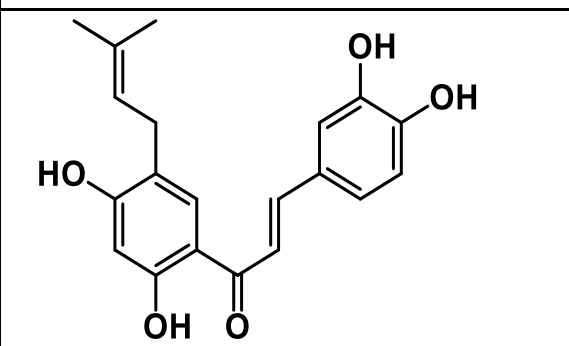	Goushu	*Broussonetia papyrifera*	*Broussonetia papyrifera Vent.* [121]
			Garcinol	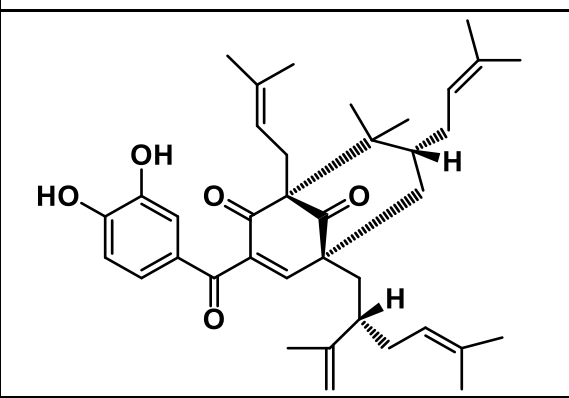	Tenghuang	*Garcinia indica*	*Garcinia indica*[122]

			Isogarcinol	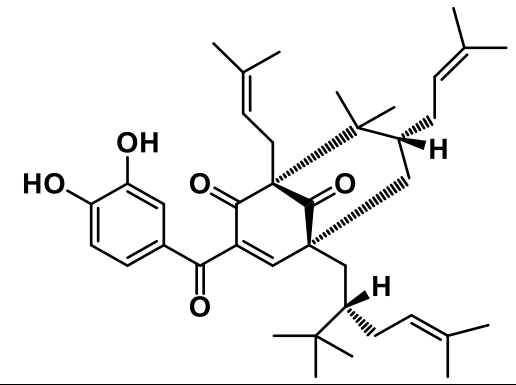	Tenghuang	*Garcinia indica*	*Garcinia indica*[122]
			Boesenbergin A	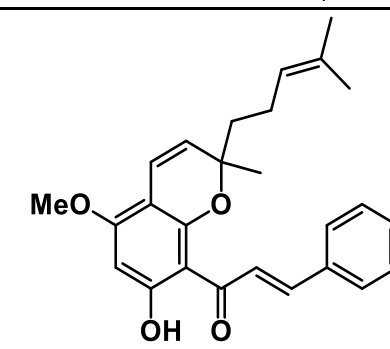	Aochunjiang	*Fingerroot*	*Boesenbergia rotunda (L.)* [123]
			Safflor yellow B	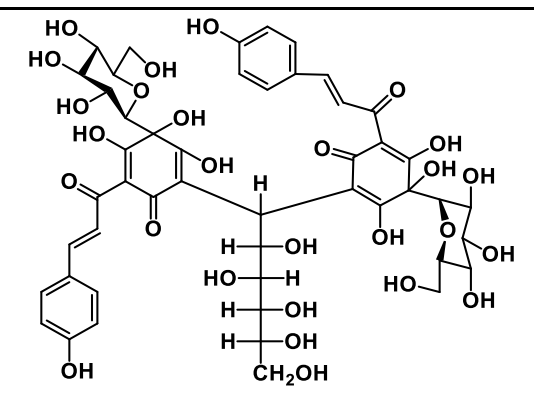	Honghua	*safflower*	*Carthamus tinctorius L.* [124, 125]
			Safflomin A;	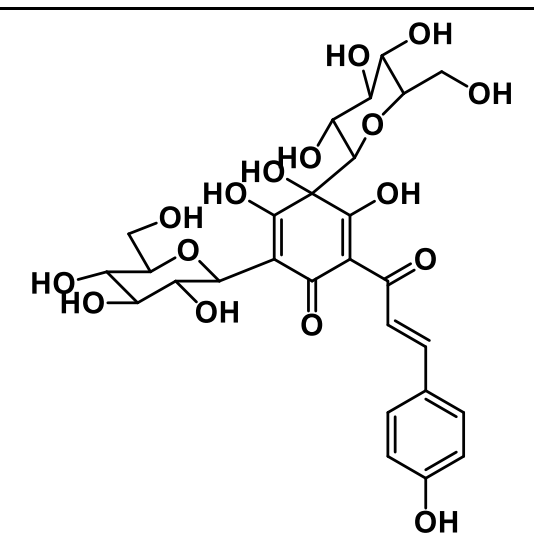	Honghua	*safflower*	*Carthamus tinctorius L.* [125, 126]
			Anhydroxysafflor Yellow B	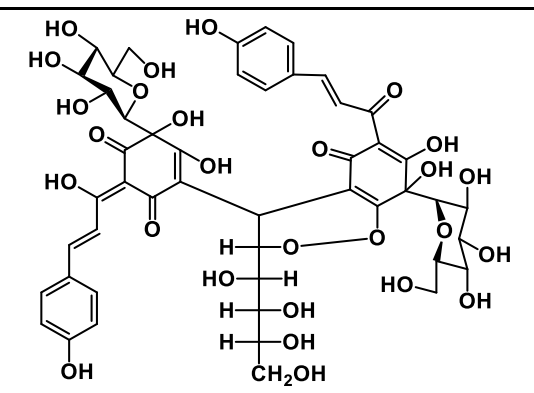	Honghua	*safflower*	*Carthamus tinctorius L.* [126]

			Hydroxysafflor yellow B	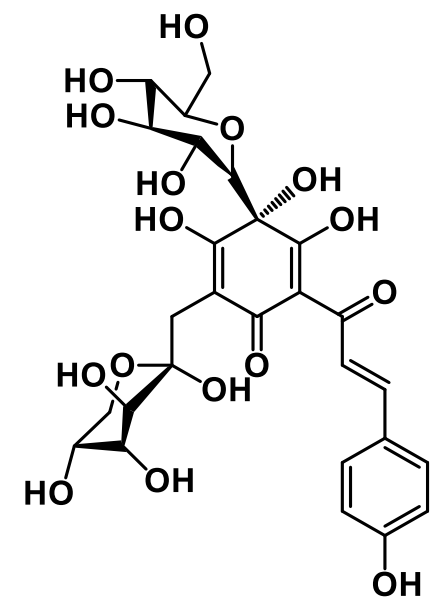	Honghua	*safflower*	*Carthamus tinctorius L.* [126]
			Hydroxysafflor yellow C	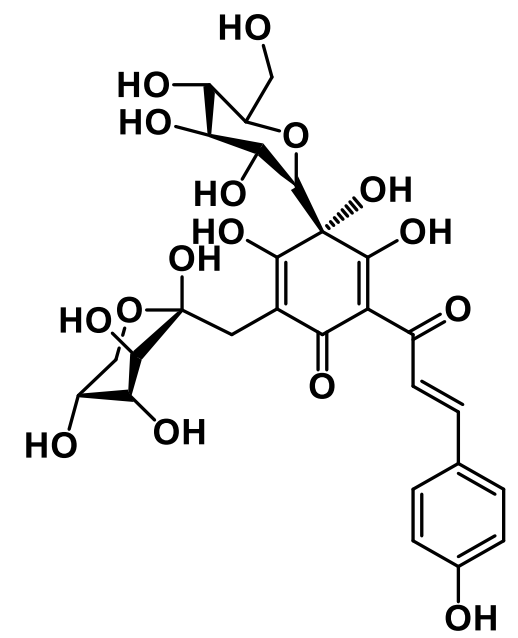	Honghua	*safflower*	*Carthamus tinctorius L.* [126]
			Safflomin C	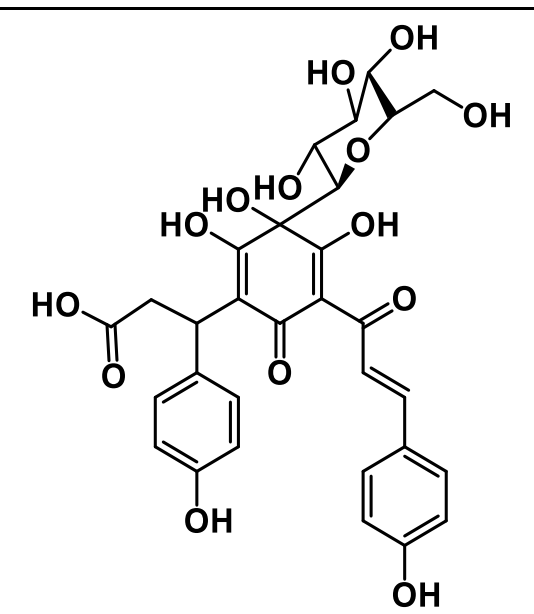	Honghua	*safflower*	*Carthamus tinctorius L.* [126, 127]

			Saffloquinoside C	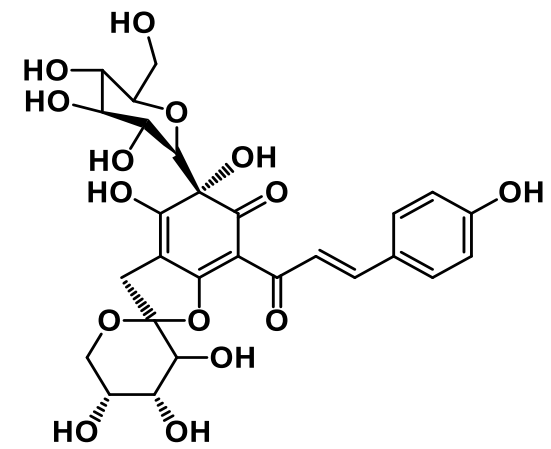	Honghua	*safflower*	*Carthamus tinctorius L.* [126]
			Flavokawains A	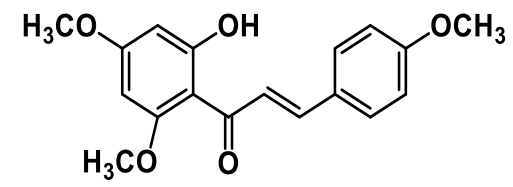	Hujiao	*kava*	*Piper methysticum Forst. f.* [128, 129]
			Flavokawains B	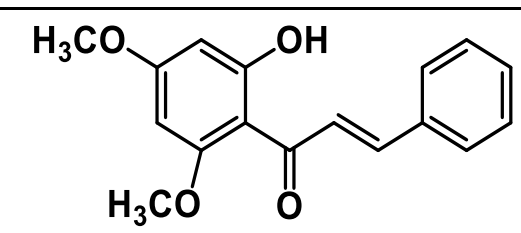	Hujiao	*kava*	*Piper methysticum Forst. f.* [128, 129]
			Flavokawains C	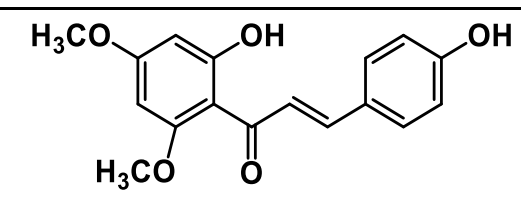	Hujiao	*kava*	*Piper methysticum Forst. f.* [129]
			Cardamonin	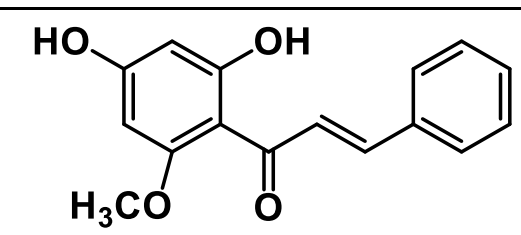	Aochunjiang	*Fingerroot*	*Boesenbergia rotunda (L.)* [129-131]
			2’,4’-dihydroxy-3’-(g,g-dimethylallyl)-3,4-methylenedioxychalcone	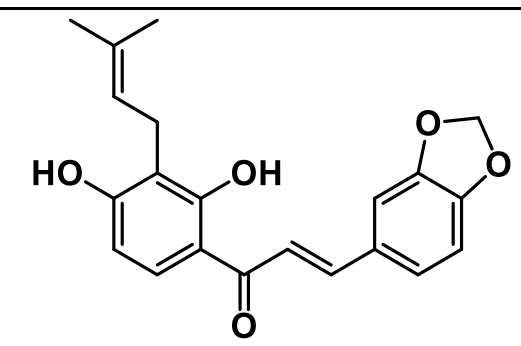	Shuihuangpi	*Pongamia*	*Pongamia pinnata (L.) Pierre*[132]
			lonchocarpin	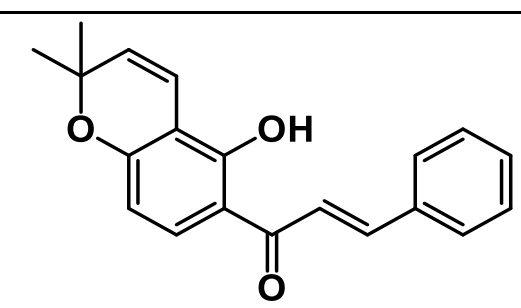	**S**huihuangpi	*Pongamia*	*Pongamia pinnata (L.) Pierre*[132, 133]
			Glabrachromene I	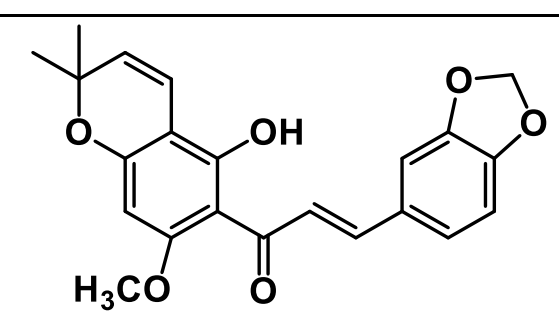	**S**huihuangpi	*Pongamia*	*Pongamia pinnata (L.) Pierre*[132, 134]

			3,4-dimethoxy-2-hydroxy-6'',6''-dimethylchromeno-[2'',3'':4',3']-chalcone	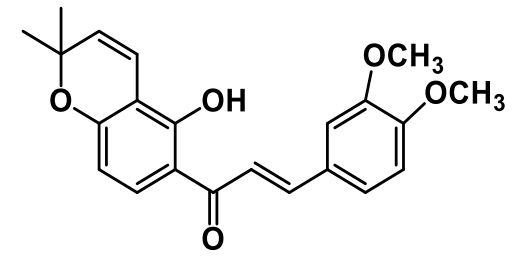	**S**huihuangpi	*Pongamia*	*Pongamia pinnata (L.) Pierre*[132, 135]
			2'-hydroxy-3,4-dimethoxy-[2'',3'':4',3']-furanochalcone	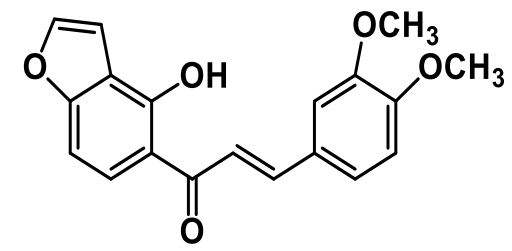	**S**huihuangpi	*Pongamia*	*Pongamia pinnata (L.) Pierre*[132, 136]
			Pongamol	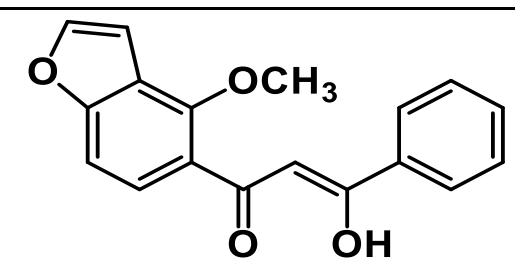	**S**huihuangpi	*Pongamia*	*Pongamia pinnata (L.) Pierre*[132, 137]
			Isocordoin	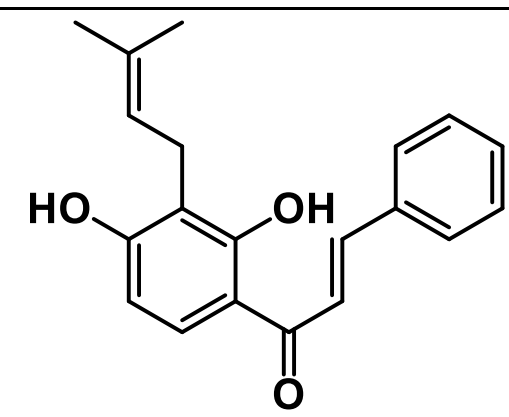	**S**huihuangpi	*Pongamia*	*Pongamia pinnata (L.) Pierre*[132, 138]
			Sappanchalcone	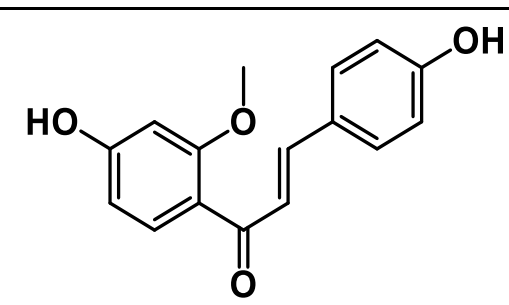	Sumu	*Sapanwood*	*Caesalpinia sappan Linn.* [139, 140]
			Morachalcone A	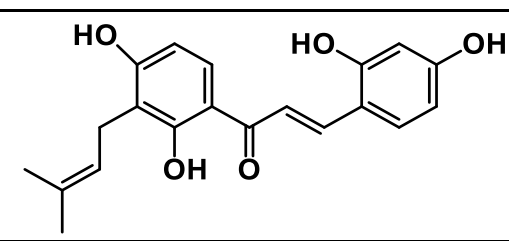	Sangshen	*mulberry;*	*Morus alba L.* [141]
			Loureirin B	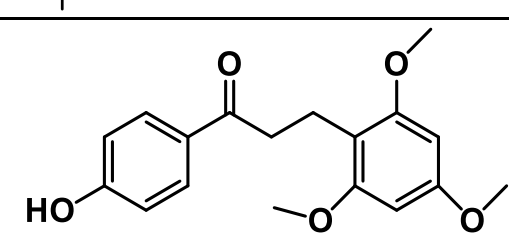	Longxuejie	*Resina Draconis*[142]	*Daemonorops draco Bl.* [143]

			Naringin dihydrochalcone	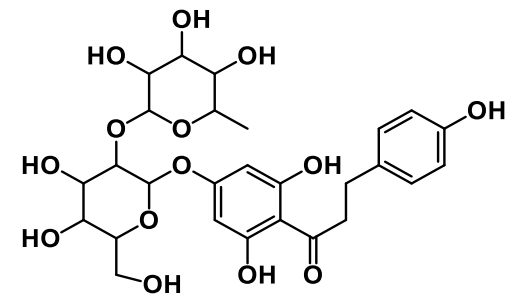	Zhishi	*Citrus aurantium L.*	*Citrus aurantium L.* [144]
			Kanzonol Y	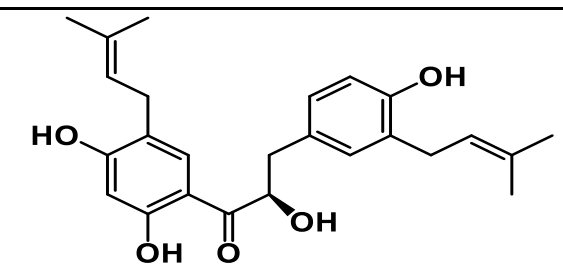	Gancao	*Glycyrrhiza glabra*	*Glycyrrhiza glabra L.* [106]
		Dihydrochalcone	Phloridzin	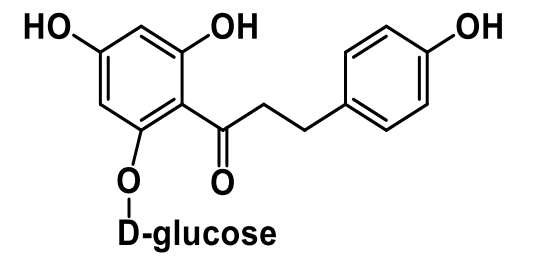	Pingguo	*Apple*	*Malus domestica* [145, 146]
			Phloretin	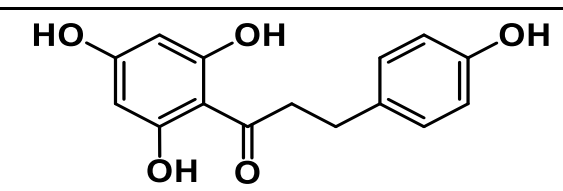	Pingguo	*Apple*	*Malus domestica* [145, 146]
			Uvangoletin	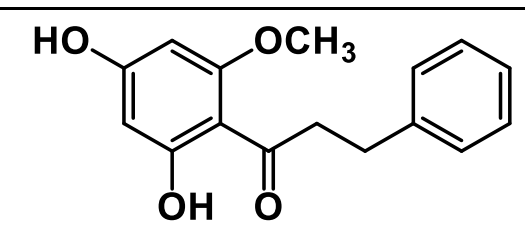	Aochunjiang	*Pandurate fingerroot*	*Boesenbergia pandurata*[147]
			loureirin A	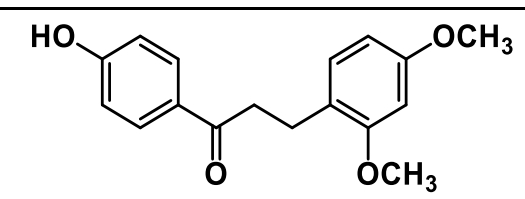	Longxue	*Dragon's blood*	*Dracaena cochinchinensis (Lour.) S. C. Chen*[143]
			Trilobatin	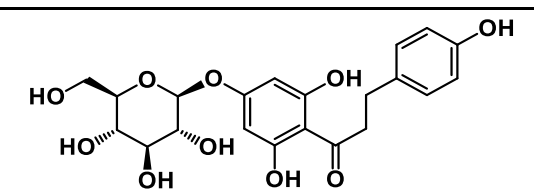	Duosuishike	*Lithocarpus polystachyus Rehd*[148]	*Lithocarpus polystachyus （Wall. ） Rehd.*
			Baicalein	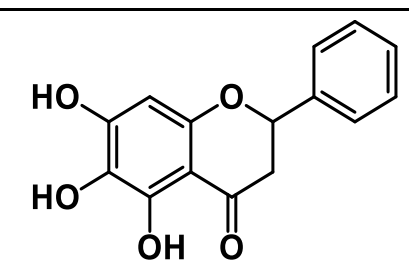	Huangqin	*Scutellariae Radix*	*Scutellaria baicalensis G eorgi* [149]
			Scutellarein	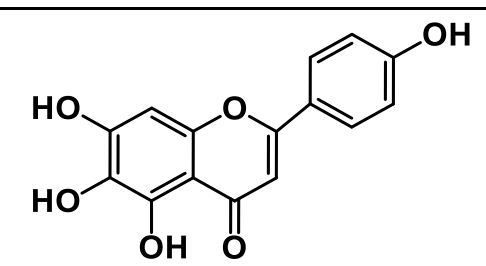	Fanxieye	*Sennae folium*	*Cassia angustifolia Vahl*[150]

			Baicalein	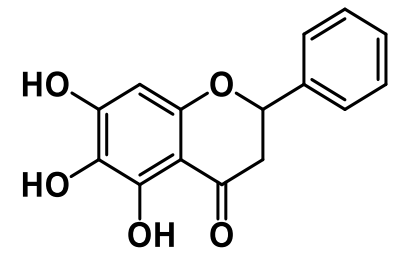	Huangqin	*Scutellariae Radix*	*Scutellaria baicalensis G eorgi* [149]
	Flavonoids	Flavone	Chrysin	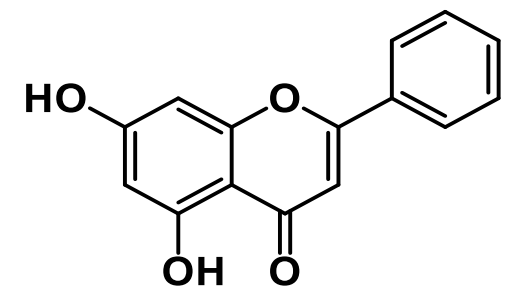	Fengjiao	Propolis[151]	*Apis mellifera L .*
			Chrysin<br>Chrysin<br>wogonin	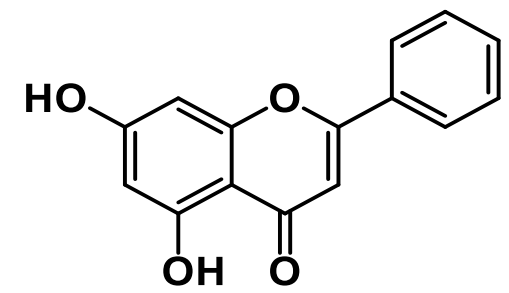<br>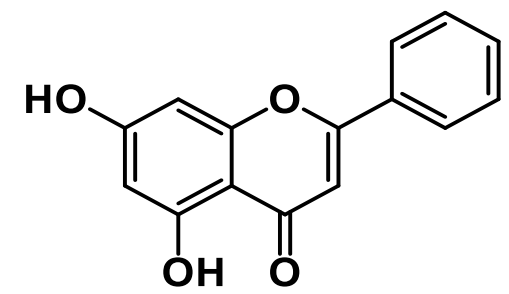<br>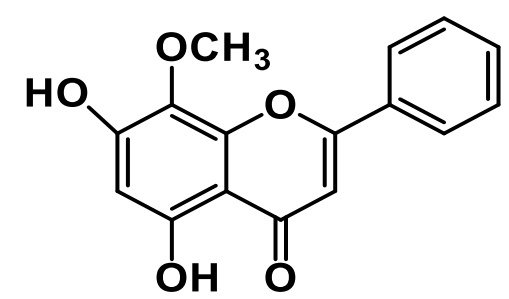	Baixiangguo	Passion fruit	*Passiflora edulis*[151]
					Yizhi	Alpinia oxyphyllae Fructus	*Alpinia oxyphylla Miquel*[152]
			Chrysin<br>wogonin<br>Apigenin	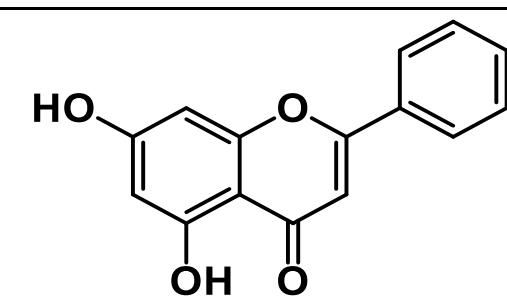<br>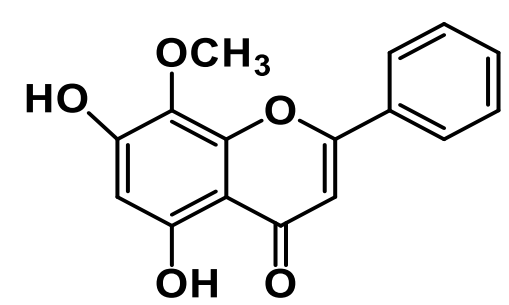	Banzhilian	*Scutellaria rivularis*[153]	*Scutellaria barbata D. Don*
					Huangqin	*Scutellariae radix*[154]	*Scutellaria baicalensis Georgi*
					Mudou	*pigeon pea*[155]	*Cajanus cajan (L.) Millsp.*

				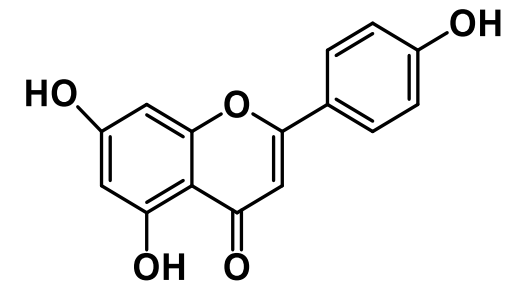			
			Apigenin<br>Acacetin<br>Chrysoeriol	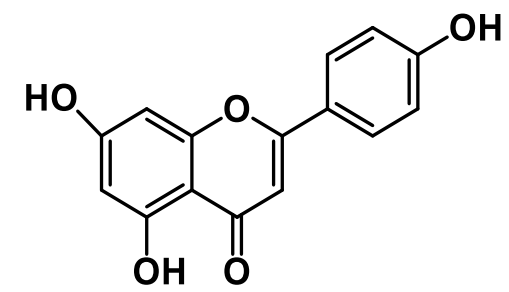<br>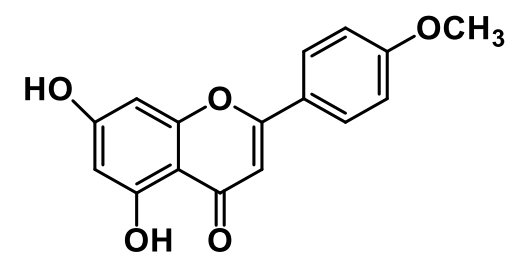<br>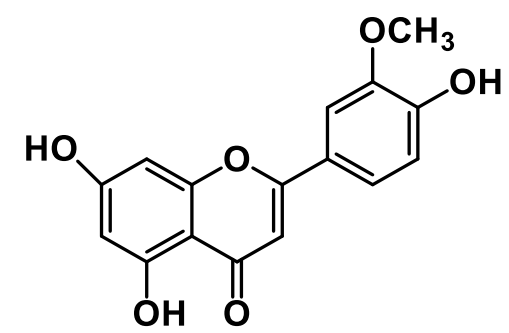	Xingzhuangfengmaoju	*Saussurea stella Maxim*[156]	*Saussurea stella Maxim*
					Chuju	*Chrysanthemum morifolim cv.*	*Dendranthema morifolium (Ramat.) Tzvel.*[157]
			Acacetin<br>Chrysoeriol<br>Acacetin	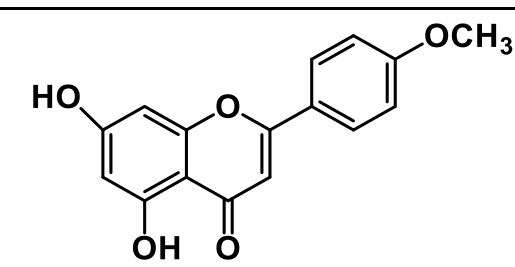<br>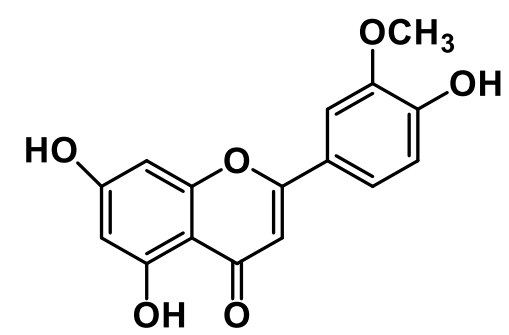<br>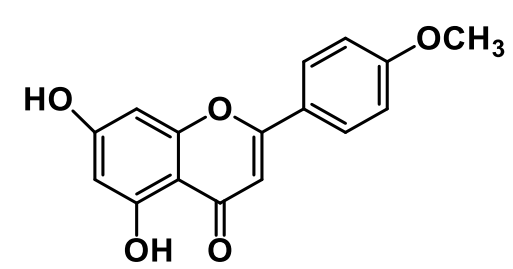	Fengmaoju	*Flos Chtysanthemi*[158]	*Saussurea japonica (Thunb.) DC.*
					Muxu	Alfalfa	*Medicago sativa L.*[159]

			Diosmetin	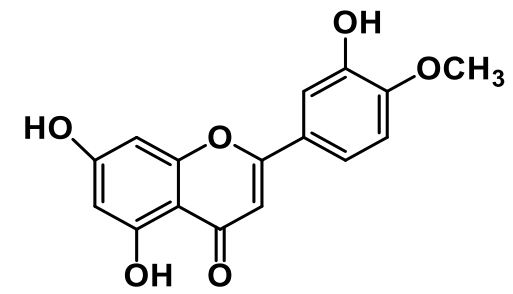	Liulanxiang	Spearmint[160]	*Mentha spicata Linn.*
			Diosmetin<br>Genkwanin<br>Luteolin	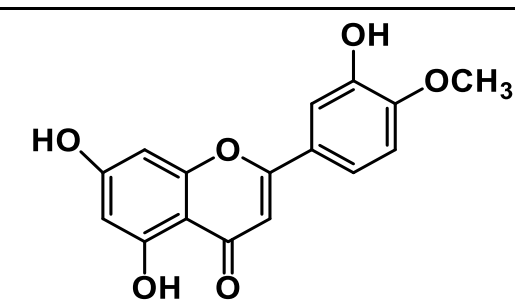<br>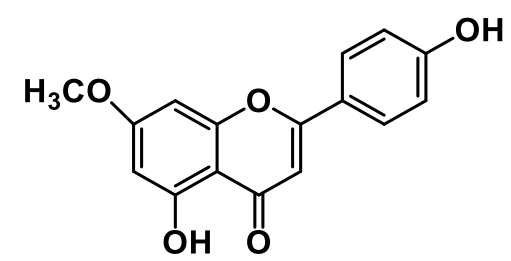<br>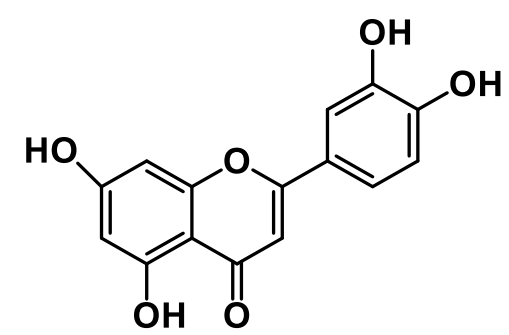	Chuanlongshuyu	Discorea nipponica Makino	*Discorea nipponica Makino*[160]
					Yuanhua	Flos Genkwa[161]	*Daphne genkwa Sieb. et Zucc.*
			Genkwanin<br>Luteolin<br>Genkwanin	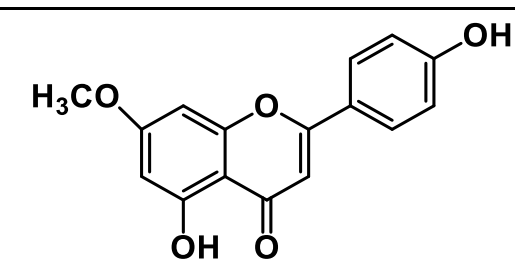<br>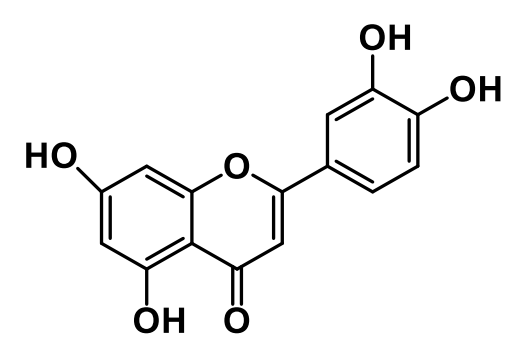<br>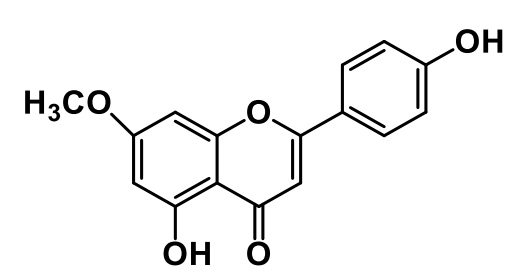	Juhua	*Chrysanthemi Flos*	*Chrysanthemum morifolium Ramat*[162]
					Diqian	*Marchantia polymorpha L.*	*Marchantia polymorpha L.*[163]

			Vitexin	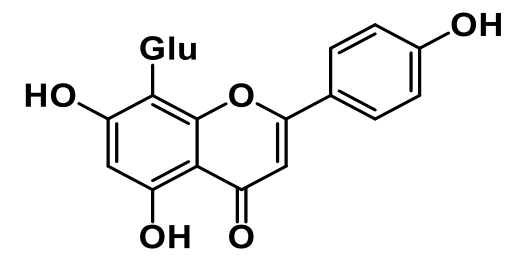	Jinlianhua	*Trollius chinensis Bunge*	*Trollius chinensis Bunge*[164]
			Vitexin<br>Scutellarin	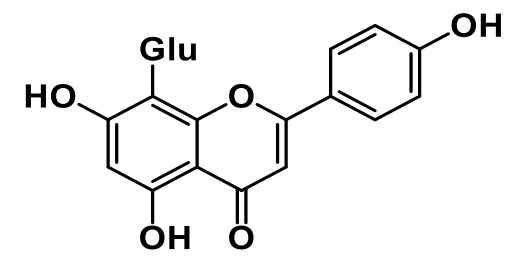<br>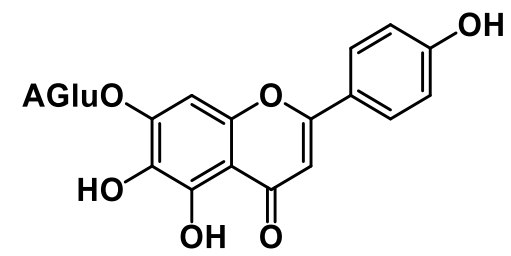	Mudou	Pigeon pea[165]	*Cajanus cajan (L.) Millsp.*
					Huangqin	*Scutellariae Radix*	*Scutellaria baicalensis Georigi*[166]
			Scutellarin<br>Scutellarin<br>Orientin<br>Cirsimaritin	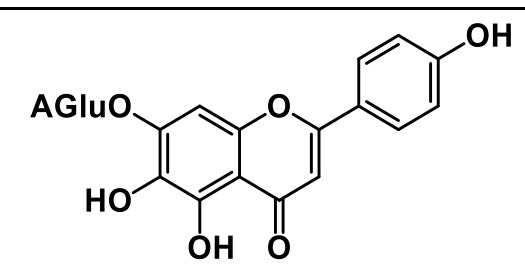<br>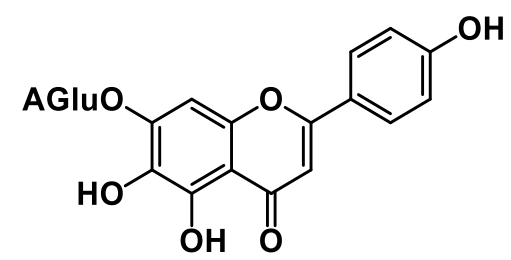<br>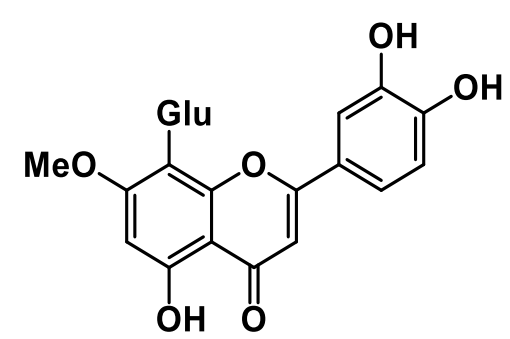<br>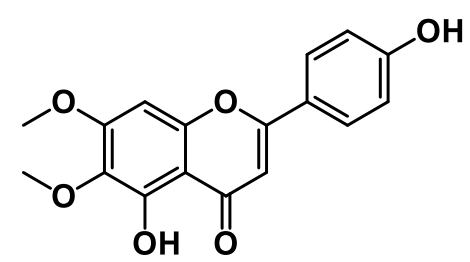	Dengzhanhua	*Erigerontis herba*	*Erigeron breviscapus (vant) Hand-Mazz*[166]
					Mabiancao	*Verbenae herba*	*Verbena officinalis L.*[167]
			Scutellarin<br>Orientin<br>Cirsimaritin<br>Eupatorin	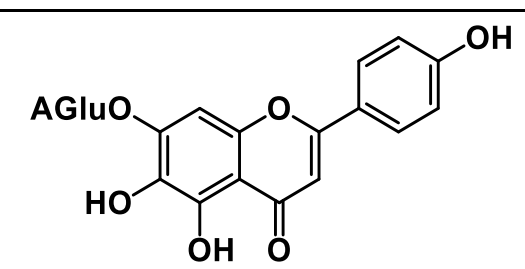	Jinlianhua	*Trollius chinensis Bunge*	*Trollius chinensis Bunge*[164]
					Maozhuateng	*Macfadyena unguis-cati*	*Macfadyena unguis-cati L.*[20]
					Huweicao	*Centaurea virgata*	*Centaurea virgata Lam.*[168]

				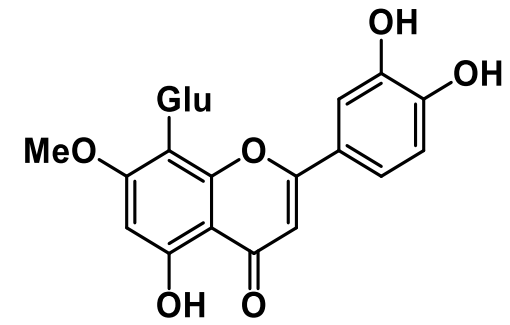 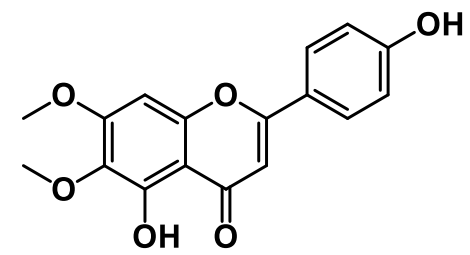 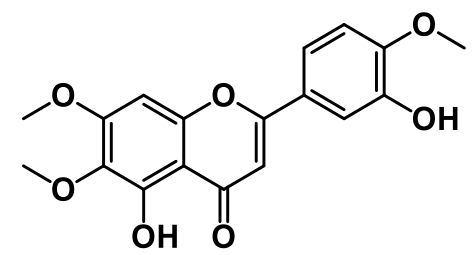			
			6- Hydroxyluteolin	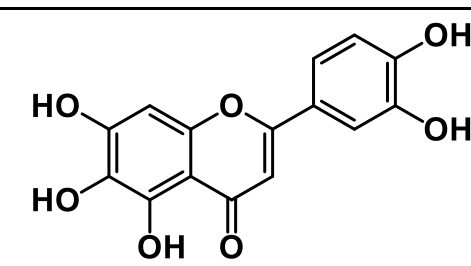	Shuiliao	*Laksa*	*Polygonum hydropiper L.*[169]
			Isoscutellarein	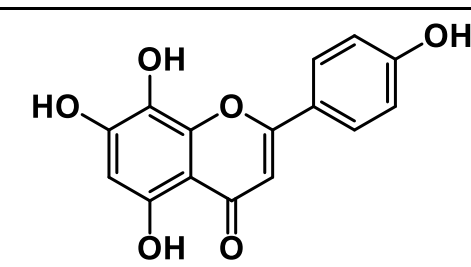	Zhaoshengshuisu	*Stachys palustris*	*Stachys palustris L.*[170]
			Sinensetin	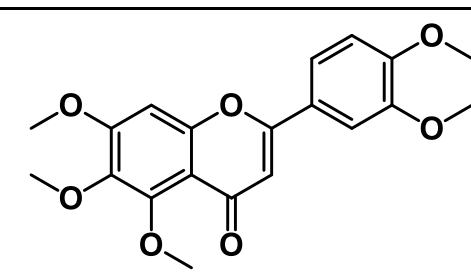	Chenpi	*Pericarpium Citri Reticulata*[171]	*Citrus reticulata Blanco*
			Nobiletin	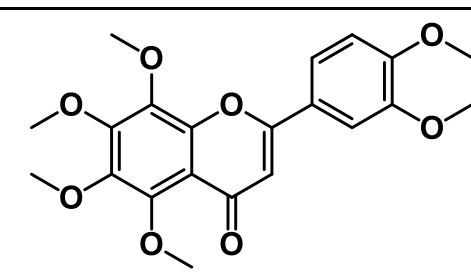	Chenpi	*citrus*[172]	*Citrus reticulata Blanco*
			Tangeretin	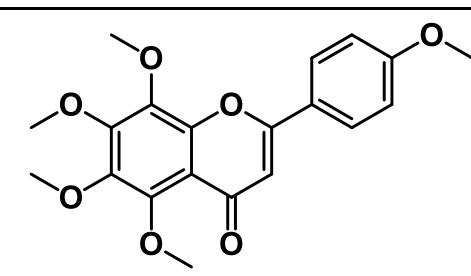	Chenpi	*citrus*[173]	*Citrus reticulata Blanco*

			Gardenin	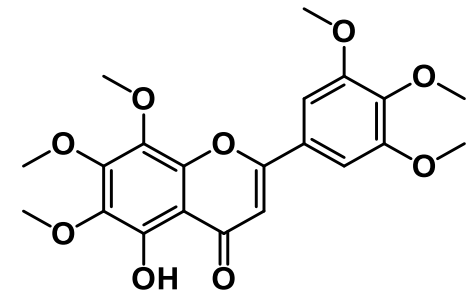	Zhizi	*Gardenia lucida*[174]	*Gardenia jasminoides Ellis*
			Australone A	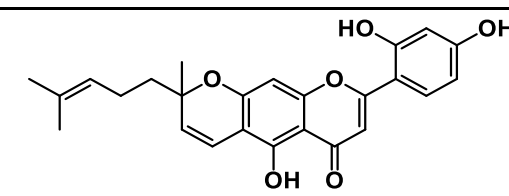	Ganzhe	*Sugarcane*	*Saccharum officinarum L.*[175]
			Albanin A	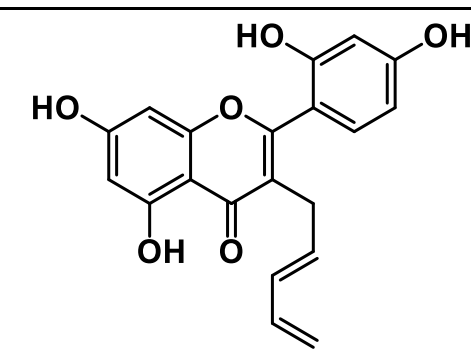	Ganzhe	*Sugarcane*	*Saccharum officinarum L.*[175]
			5'-Geranyl-5,7,2',4'-tetrahydroxy-flavone	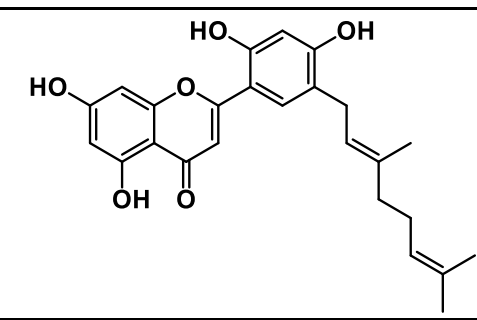	Ganzhe	*Sugarcane*	*Saccharum officinarum L.*[175]
			nevadensin	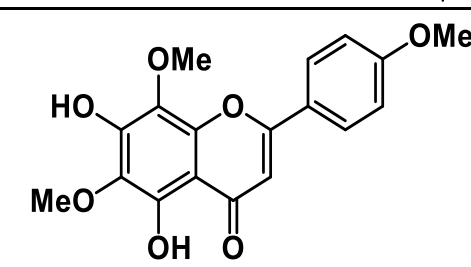	Yiyeqinglan	*Limnophila geoffrayi*	*Limnophila geoffrayi*[176]
			diosmin	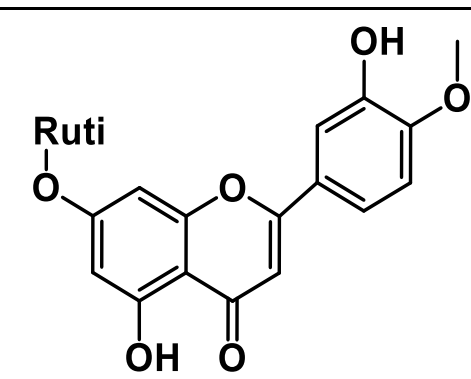	Zhangyetuowu	*Ligularia przewalskii*	*Ligularia przewalskii (Maxim.) Diels* [177]
			apigenin-4′,7-dimethyl ether	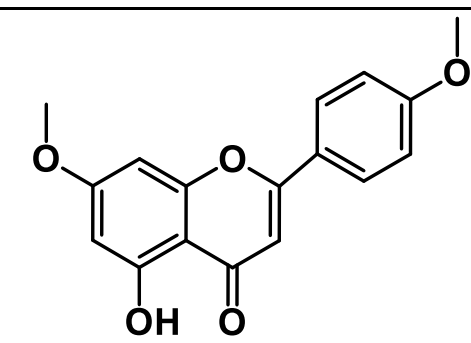	Baimao	*cogongrass*	*TEUCRIUM POLIUM L.*[129]

			luteolin-3′,4′,7-trimethyl ether	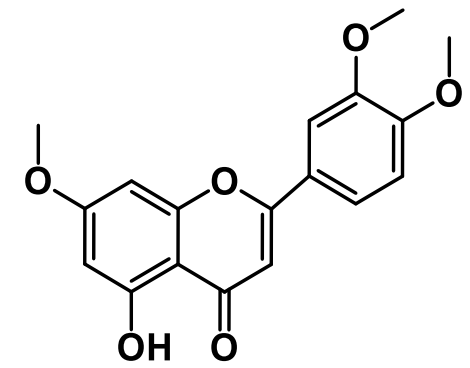	Shanfeng	*Blumea aromatica*	*Blumea aromatica* （*Wall.* ）*DC.*[129]
			hydroxygenkwanin	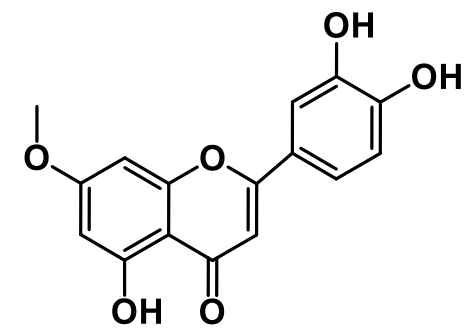	Yuanhua	*Daphne*	*Daphne genkwa Sieb. et Zucc.*[129]
			Daidzein	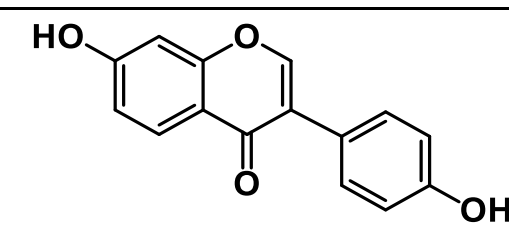	Dadou	Soybeans[178]	*Glycine max (Linn.) Merr.*
			Biochanin A	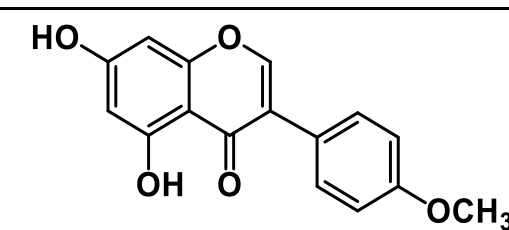	Jiangxiangye	*Dalbergiae odoriferae leaves*	*Dalbergia odorifera T. Chen leaves*[179]
			Genistein	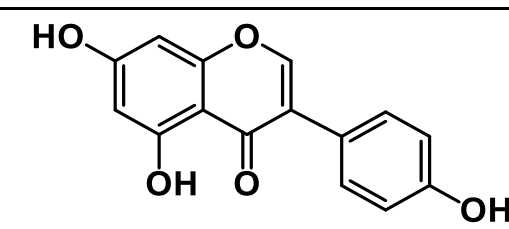	Jiangxiangye	*Dalbergiae odoriferae leaves*	*Dalbergia odorifera T. Chen leaves*[179]
		Isoflavone	Cajanin	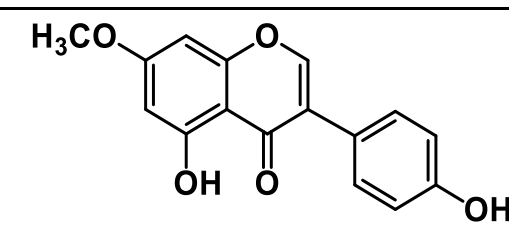	Mudou	Pigeon pea[155]	*Cajanus cajan (L.) Millsp.*
			Calycosin	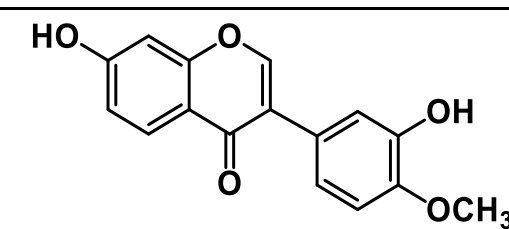	Huangqi	*Radix Astragali*[180]	*Astragalus membranaceus (Fisch.) Bge. var. mongholicus (Bge.) Hsiao*
			Formononetin	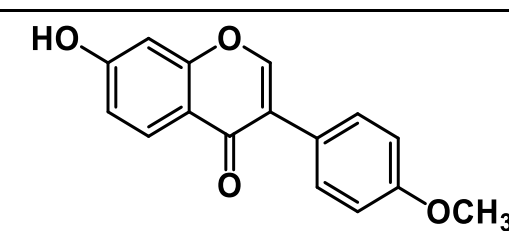	Huangqi	*Radix Astragali*[180]	*Astragalus membranaceus (Fisch.) Bge. var. mongholicus (Bge.) Hsiao*

			Glycitein	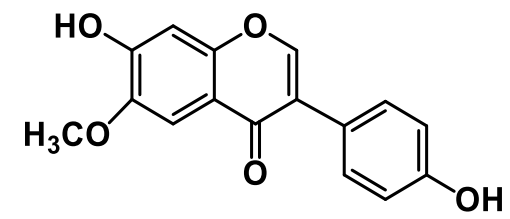	Dadou	Soybeans[181]	*Glycine max (Linn.) Merr.*
			Prunetin	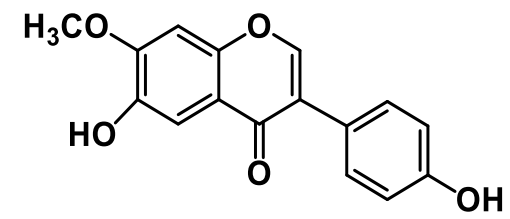	Kuyingtao	*Bitter cherry*	*Prunus emarginata*[182]
			Tectoridin	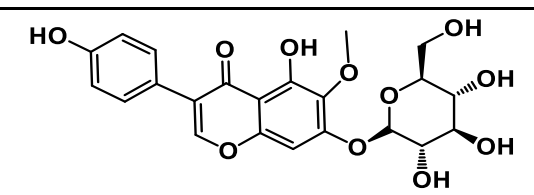	Gegenhua	*Pueraria lobata flower*[183]	*Pueraria lobata (Willd.) Ohwi*
			Genistin	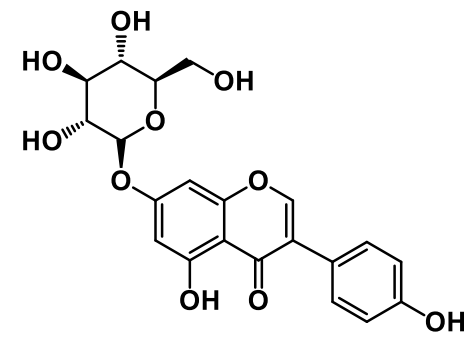	Shapeng	*Agriophyllum squarrosum seed*[184]	*Agriophyllum squarrosum (L.) Moq.*
			Glyurallin B	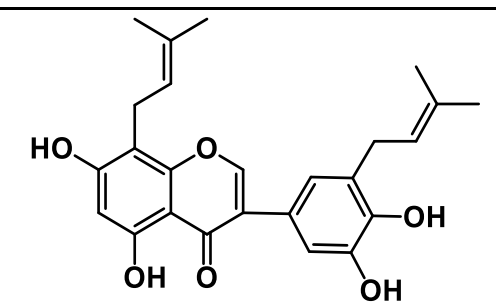	Gancao	*Licorice*	*Glycyrrhiza uralensis Fisch.*[185]
			Glycyuralin F	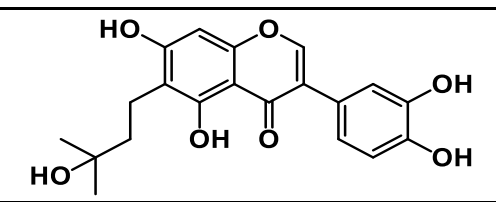	Gancao	*Licorice*	*Glycyrrhiza uralensis* [186]
			Orobol	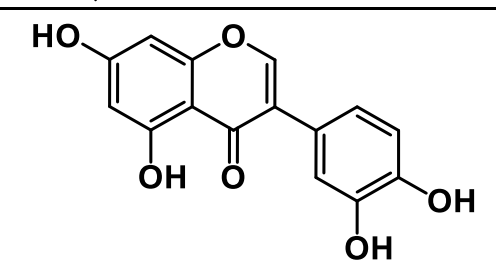	Lizi	*Pear*	*Pyrus ussuriensis Maxim.*[187]
			quercetin	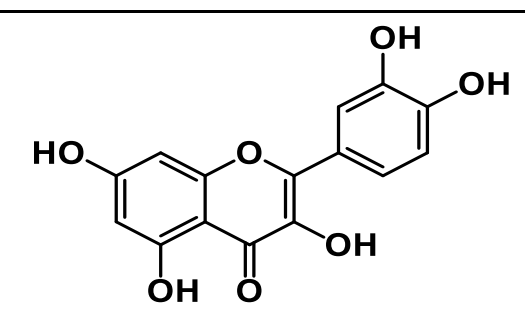	Puhuang	*Typhae Pollen*[188]	*Typha angustifolia L*

			Orobol	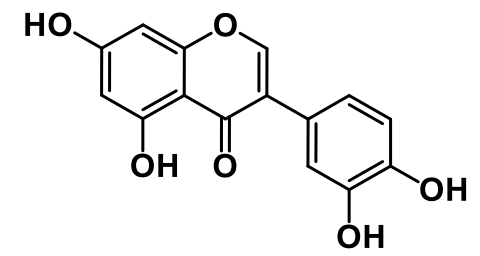	Gouqizi	*Lycii Fructus*[189]	*Lycium barbarum L.*
			quercetin<br>Kaempferol	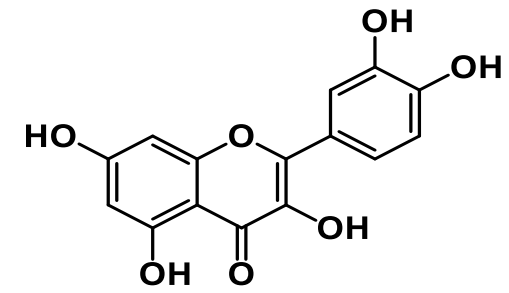<br>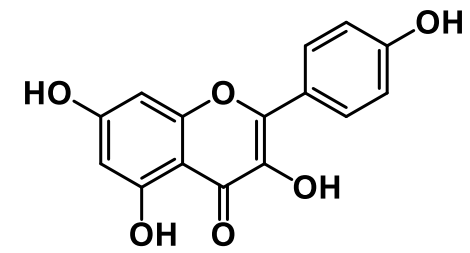	Sangshen	*Mori Fructus*[190]	*Morus alba L.*
		Flavonol	quercetin<br>Kaempferol<br>Kaempferol<br>Kushenol C<br>Galangin	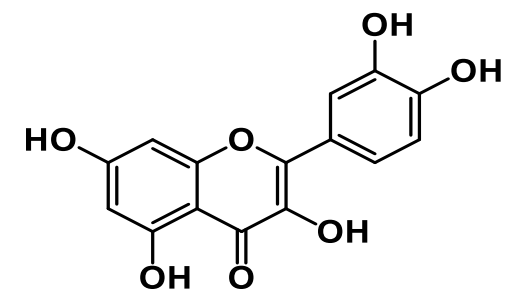<br>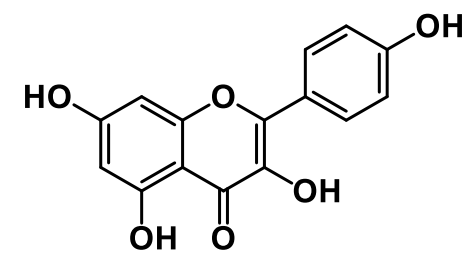<br>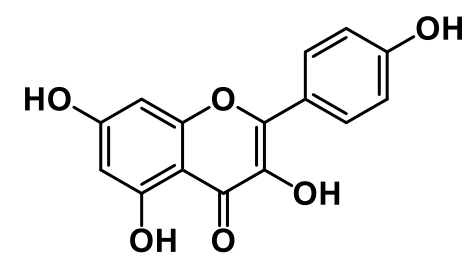<br>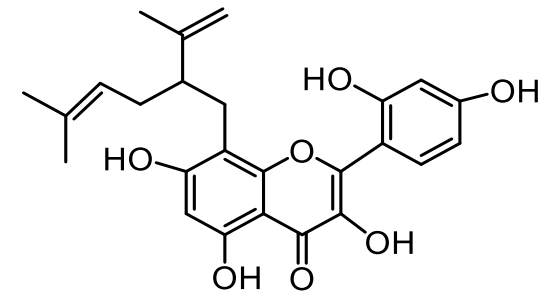	Wuweizi	*Schisandrae Chinensis Fructus*	*Schisandra chinensis (Turcz.)Baill*[191]
					Puhuang	*Typhae Pollen*[188]	*Typha angustifolia L*
					Luhui	Aloe[192]	*Aloe barbadmsis Miller*

				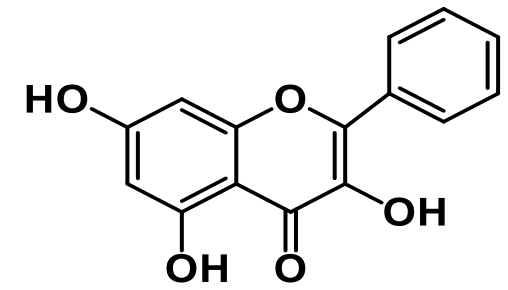			
			Kaempferol<br>Kushenol C<br>Galangin<br>Kaempferide	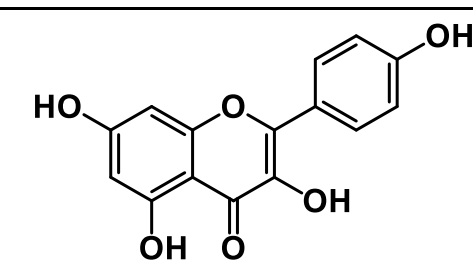<br>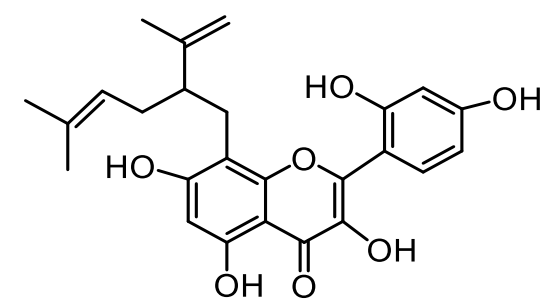<br>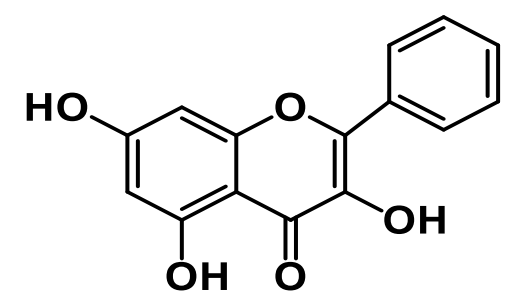<br>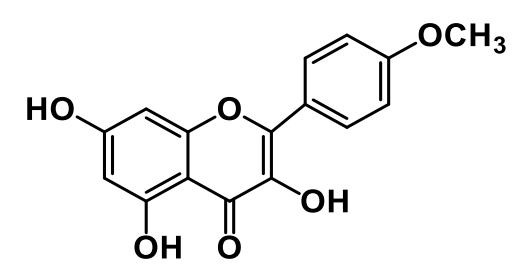	Kusen	*Sophorae flavescentis radix*	*Sophora flavescens Ait.*[101]
					Gaoliangjiang	*Alpinia galanga L.*[193]	*Alpinia officinarum Hance*
					Gaoliangjiang	*Alpinia galanga L.*[193]	*Alpinia officinarum Hance*
			Isorhamnetin	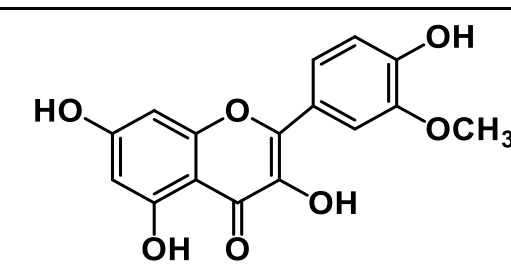	Shajiguozha	*Sea Buckthorn Marc*[194]	*Sea Buckthorn Marc*
			Myricetin	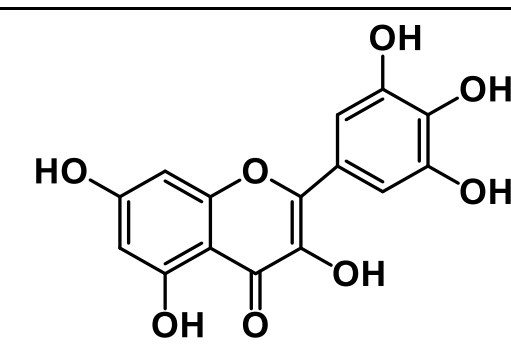	Yangmei	*Myrica rubra Zucc*[195]	*Myrica rubra (Lour.) Sieb. et Zucc.*

			Morin	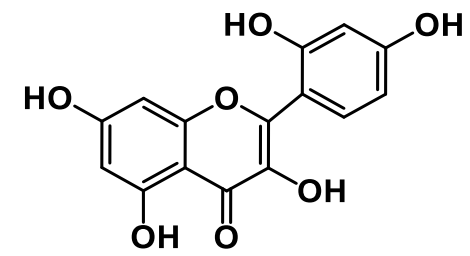	Sangshen	*Mulberry Fruits*	*Morus alba L.*[196]
			Glycyrrhiza-flavonol A	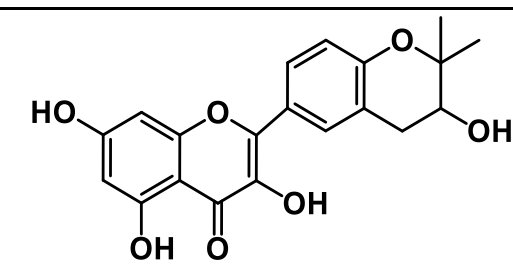	Gancao	*liquorice*[197]	*Glycyrrhiza uralensis Fisch.*
			Izalpinin	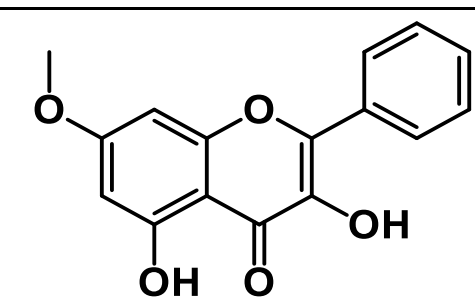	Yizhiren	*Fructus Alpiniae Oxyphyllae*	*Alpinia oxyphylla Miq.*[198]
			Rhamnetin	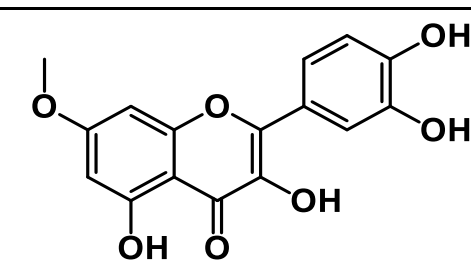	Shijunzi	*Combretaceae*	*Guiera senegalensis J. F. Gmelin* [198, 199]
			Isoginkgetin	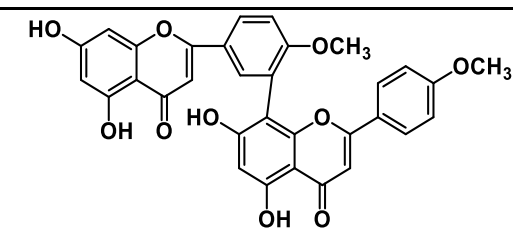	Yinxingye	*Ginkgo Folium*	*Ginkgo biloba L.*[200]
			Amentoflavone	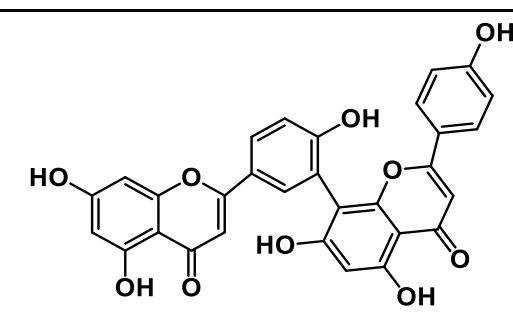	Juanbai	*Selaginellar herba*[201]	*Selaginella tamariscina (Beauv.) Spring*
			Ginkgetin	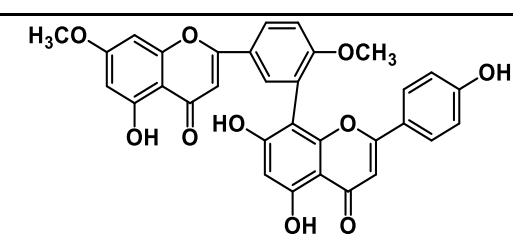	Yinxingye	*Ginkgo Folium*	*Ginkgo biloba L.* [200]
		Biflavone	Bilobetin	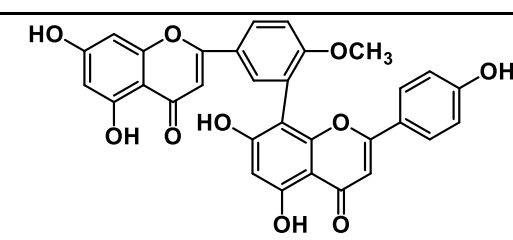	Yinxingye	*Ginkgo Folium*	*Ginkgo biloba L.*[200]

			Cupresuflavone	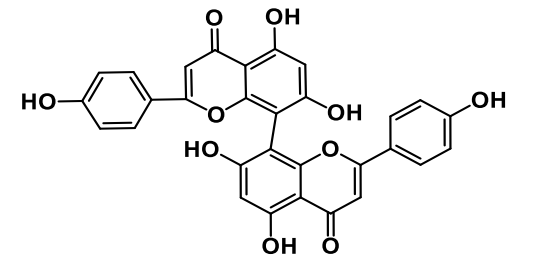	Juanbai	*Selaginellar herba*[201]	*Selaginella tamariscina (Beauv.) Spring*
			Hinokiflavone	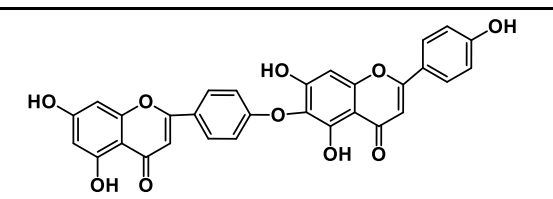	Juanbai	*Selaginellar herba*	*Selaginella tamariscina (Beauv.) Spring*[202]
			Hesperetin	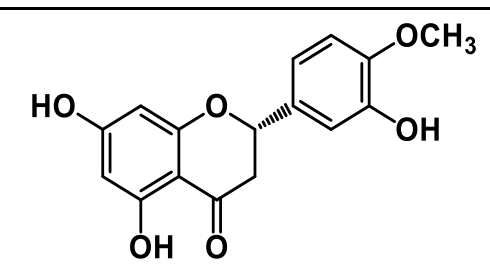	Chenpi	*Citri reticulatae Pericarpium*[203]	*Citrus reticulata Blanco*
			Hinokiflavone	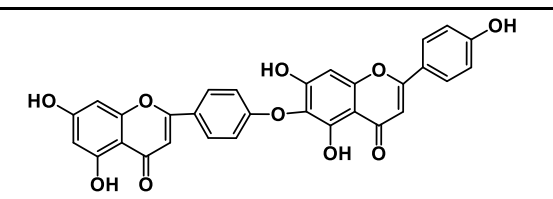	Zhiqiao	*Aurantii Fructus*[204]	*Citrus aurantium L.*
			Sophoraflavanone G<br>Alpinetin	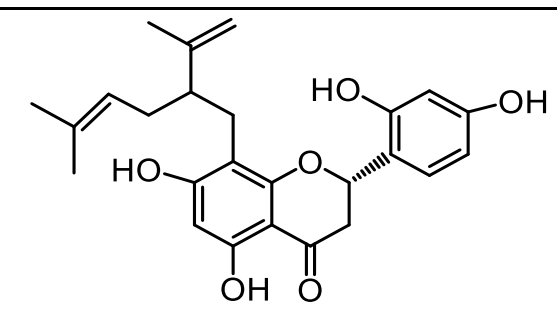 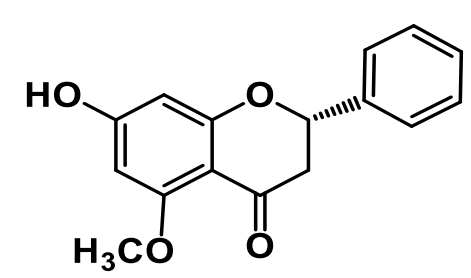	Kusen	*Sophorae flavescentis radix*	*Sophora flavescens Ait.*[101]
		Dihydroflavone	Sophoraflavanone G<br>Alpinetin<br>Naringenin	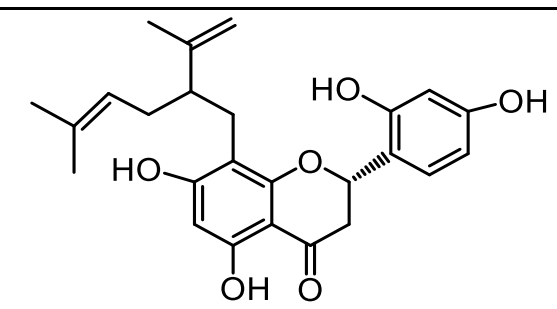 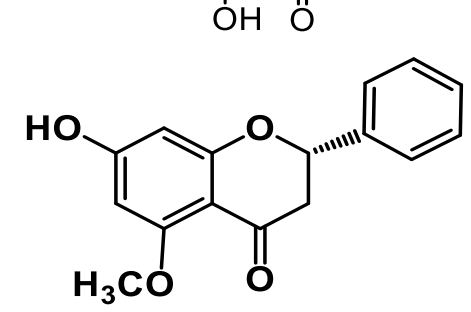	Caodoukou	*Alpiniae katsumadai Semen*	*Alpinia katsumadai Hayata*[205]
					Juhong	*Citri exocarpium rubrum*[206]	*Citrus reticulata Blanco*

				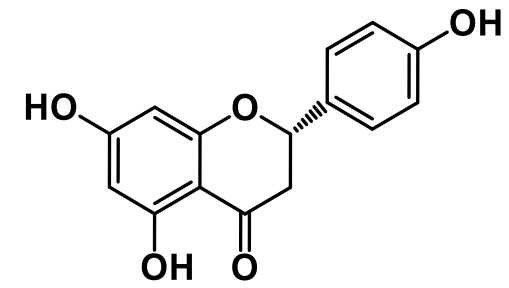			
			Alpinetin	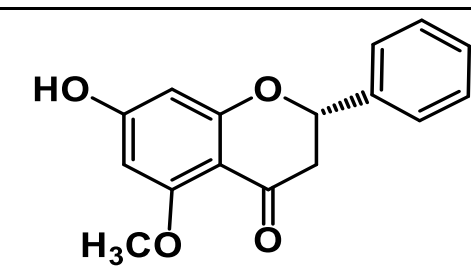	Zhiqiao	*Aurantii Fructus*	*Citrus aurantium L.*[206]
			Butin<br>Eriodictyol	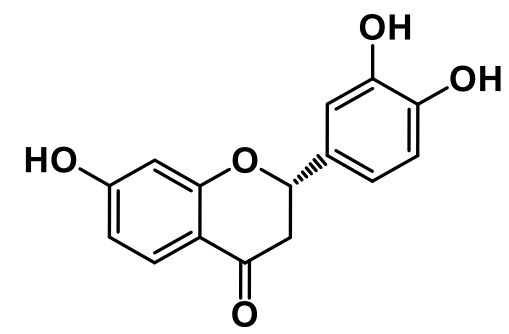<br>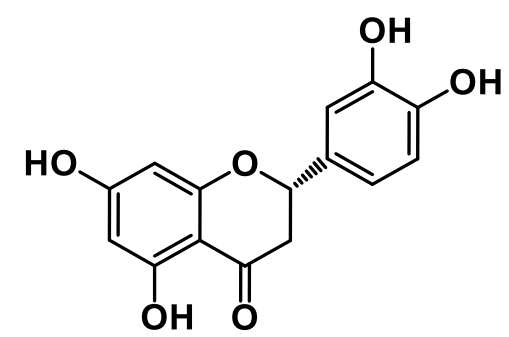	Jiangxiang[207]	*Dalbergiae odoriferae*	*Dalbergia odorifera T. Chen*
			Butin<br>Eriodictyol<br>Butin	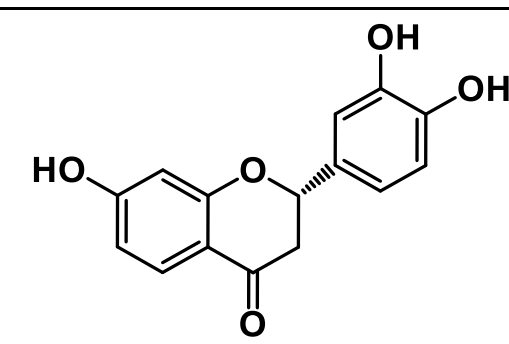<br>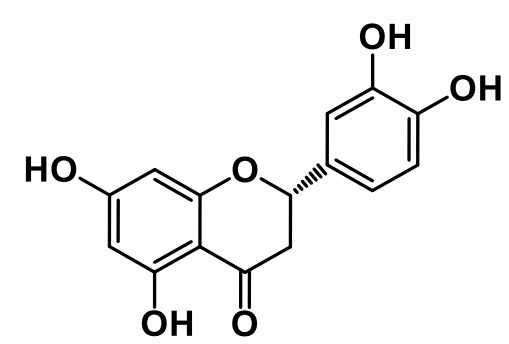	Juhong	*Citri exocarpium rubrum*[208]	*Citrus reticulata Blanco*
					Chenpi[208]	*Citri reticulatae pericarpium*	*Citrus reticulata Blanco*

				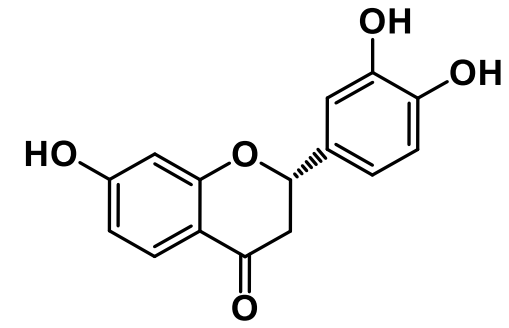			
			Liquiritigenin<br>Pinocembrin	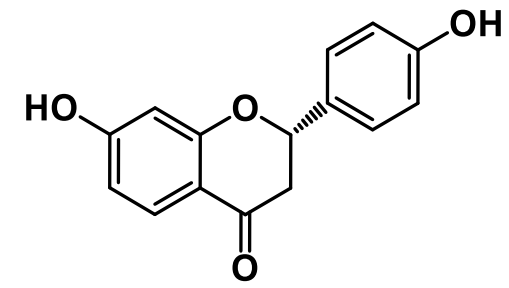<br>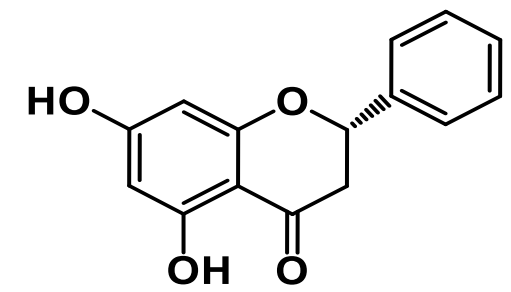	Gancao	*Glycyrrhiza glabra*[209]	*Glycyrrhiza glabra L.*
			Liquiritigenin<br>Pinocembrin<br>Hesperidin	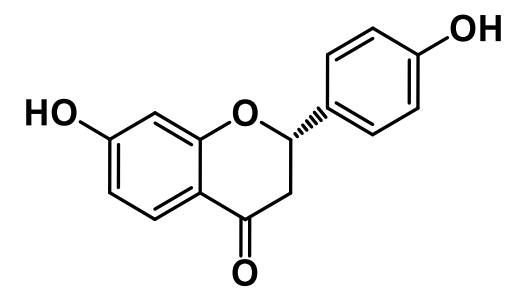<br>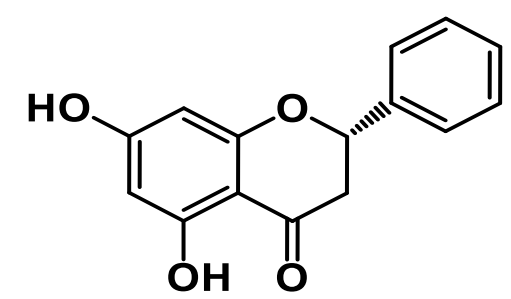<br>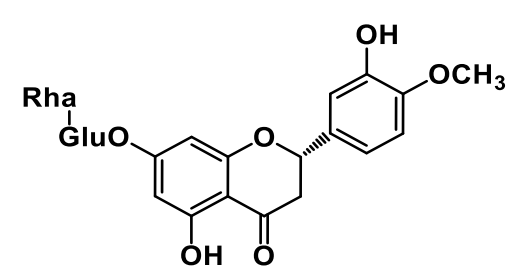	Gancao	*Glycyrrhiza glabra*	*Glycyrrhiza glabra L.*[198, 210]
					Chenpi[211]	*Citri reticulatae pericarpium*	*Citrus reticulata Blanco*
			Pinocembrin	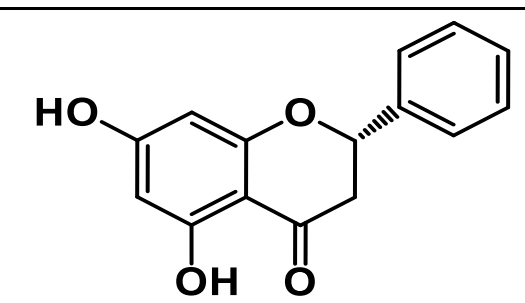	Zhiqiao	*Aurantii Fructus*	*Citrus aurantium L.*[211]

			Pinostrobin<br>3-(4'-hydroxy-benzyl)-5,7-dihydroxy-6-methyl-8-methoxy-chroman-4-one	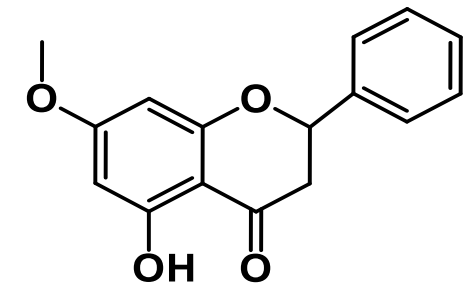<br>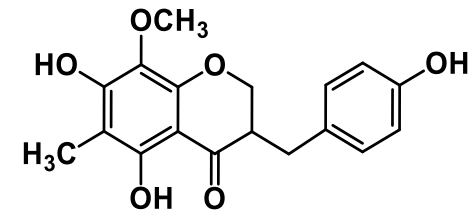	Mudou	*pigeon pea*	*Cajanus cajan (Linn.) Millsp.*[198, 212]
			Pinostrobin<br>3-(4'-hydroxy-benzyl)-5,7-dihydroxy-6-methyl-8-methoxy-chroman-4-one<br>3-(4'-hydroxyl-benzyl)-5,7-dihydroxy-6-methyl-chroman-4-one	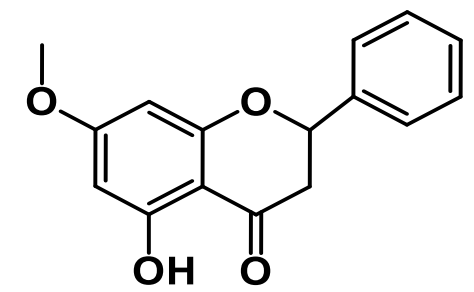<br>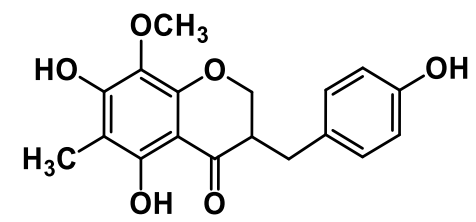<br>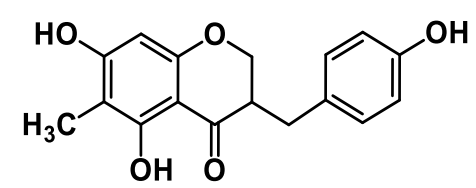	Yuzhu	*Polygonatum*	*Polygonatum odoratum （Mill. ）Druce*[213]
					Yuzhu	*Polygonatum*	*Polygonatum odoratum （Mill. ）Druce*[213]
			Sigmoidin A	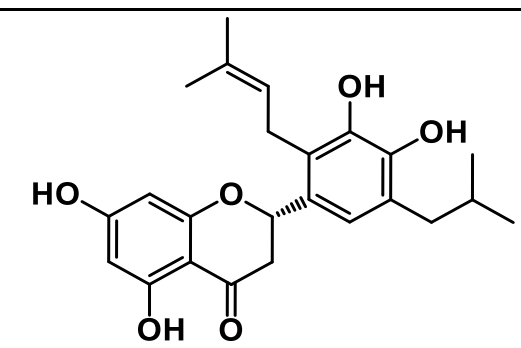	Honghuacitong	*Erythrina variegata*	*Erythrina sigmoidea Hua*[214]
			Sigmoidin B	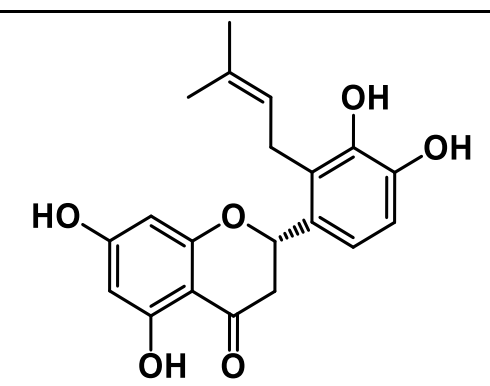	Honghuacitong	*Erythrina variegata*	*Erythrina sigmoidea Hua*[214]
			2'-4'-dihidroxy-5'-(1'''-dimethylallyl)-6-prenylpinocembrin)	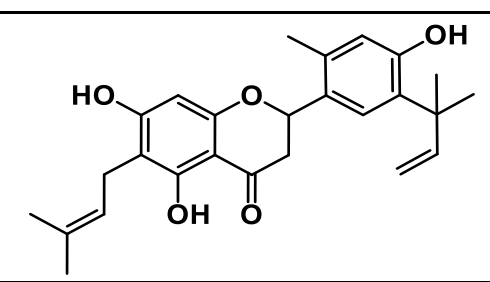	Daliju	*Dalea*	*Dalea elegans*[215]

			dihydroquercetin (taxifolin)	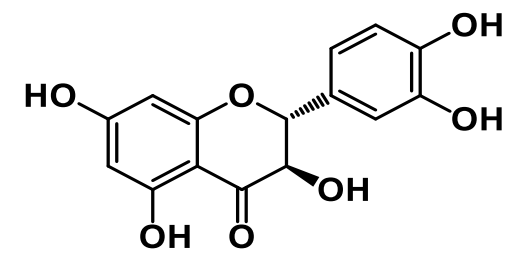	Tufuling	*Smilacis Glabra*	*Smilax glabra Roxb.*[216]
			Silybin	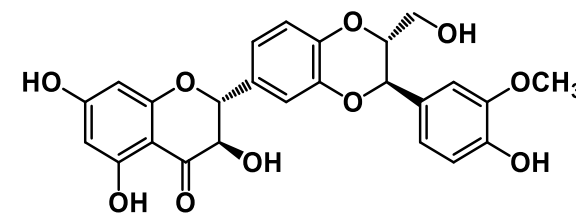	Shuifeiji	*Silybi Fructus*	*Silybum marianum(L.) Gaertn* [217]
			Silibinin A	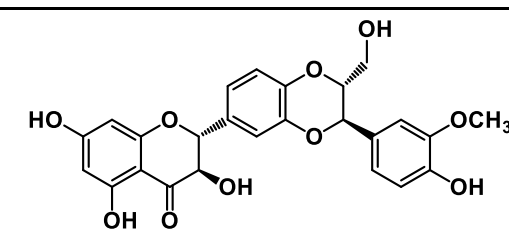	Shuifeiji	*Silybi Fructus*	*Silybum marianum(L.) Gaertn*[218]
		Dihydroflavonol	Dihydrokaempferol	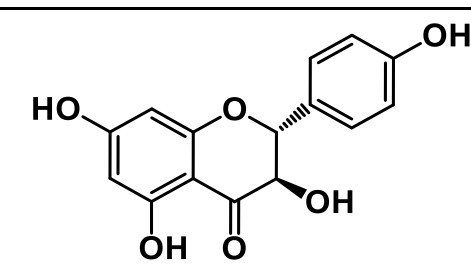	Yangchong	*scaly leaf of shallot*	*Allium cepa L.*[219]
			Dihydrokaempferide	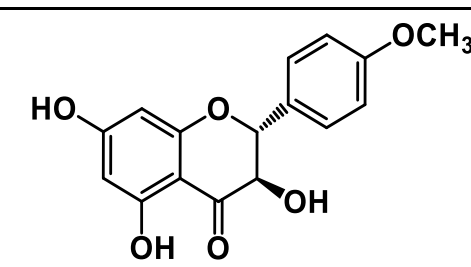	Suanmei	*Prunus domestica*	*Prunus domestica L.*[220]
			Dihydromyricetin	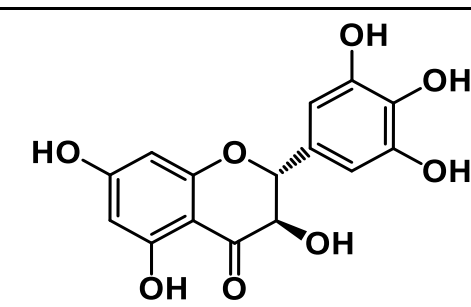	Tengcha [221]	*Ampelopsis grossedentata*	*Ampelopsis grossedentata W.T.wang*
			Astilbin	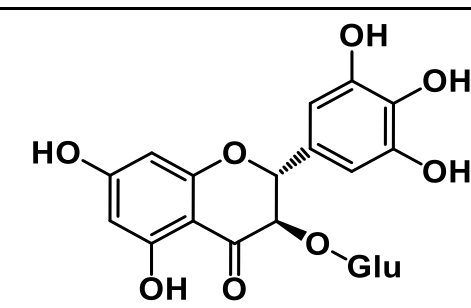	Tufuling	*Smilax glabra*[222]	*Smilax glabra Roxb.*
			Dihydromyricetin	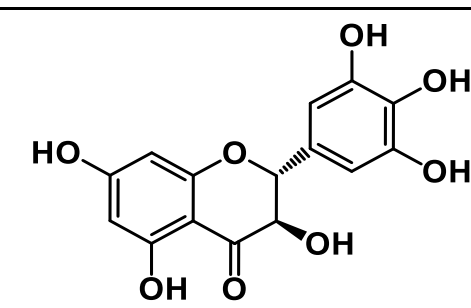	Caoshanhu[223]	*Sarcandra glabra*	*Sarcandra glabra (Thunb.) Nakai*

			Cedrin	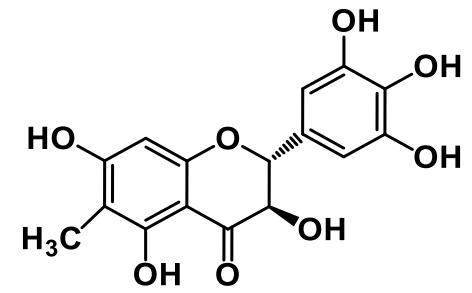	Xuesong	Cedar	*Cedrus deodara (Roxb.) G. Don*[224]
			Cedrin	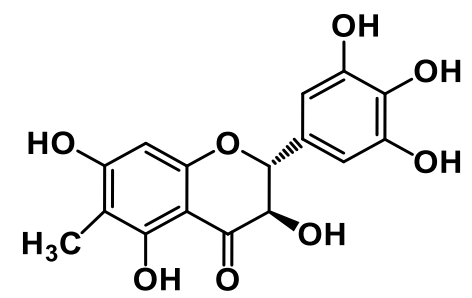	Baqia	*Smilax china*	*Smilax china L.*[225]
			Isoengeletin	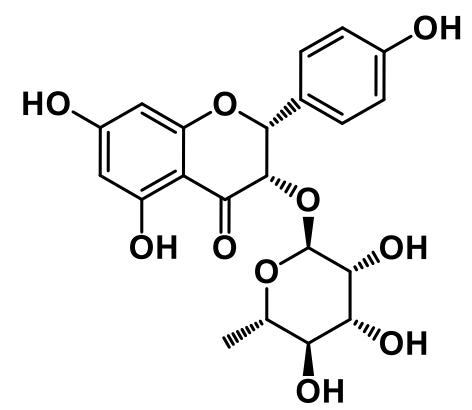	Baqia	*Smilax china*	*Smilax china L.* [226]
			7-methylaromadendrin	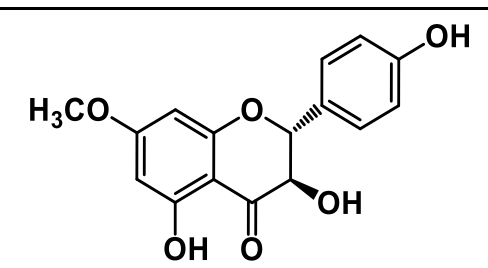	Yinbaiyang	*Populus alba*[227]	*Populus alba L.*
			Isoengeletin	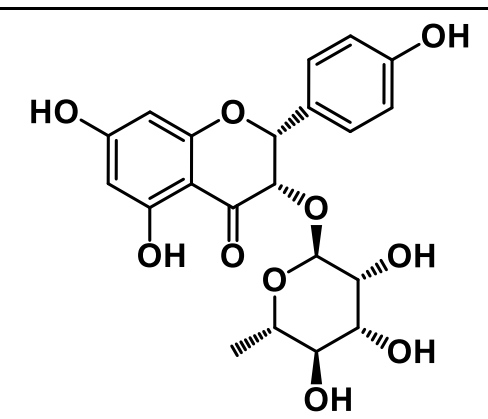	Longhao	*Artemisia dracunculus*[227]	*Artemisia dracunculus L.*
			Sophoraisoflavanone A<br>kenusanone H	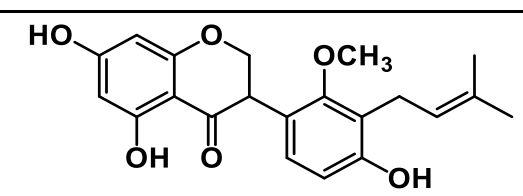 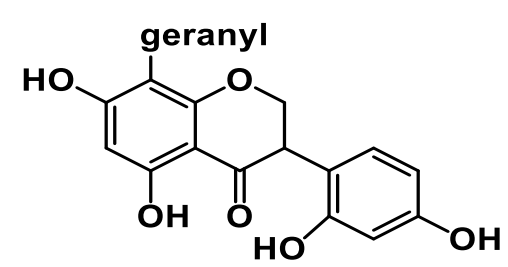	Goukushen	*Echinosophora koreensis*	*Echinosophora koreensis* [228]

			Sophoraisoflavanone A<br>kenusanone H<br>Panasenoside	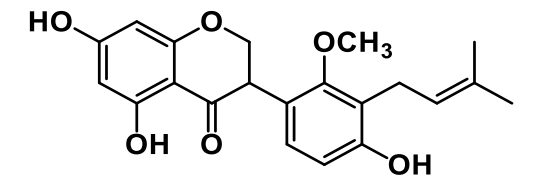<br>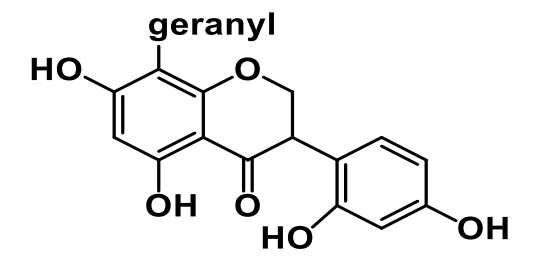<br>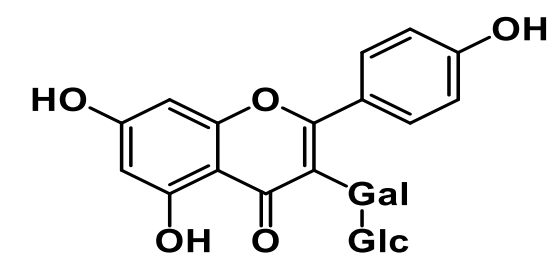	Goukushen<br>Renshen	*Echinosophora koreensis*<br>Ginseng	*Echinosophora koreensis*[228]<br>*Panax ginseng C. A. Mey.* [229]
		Dihydroisoflavone	Isoquercitrin	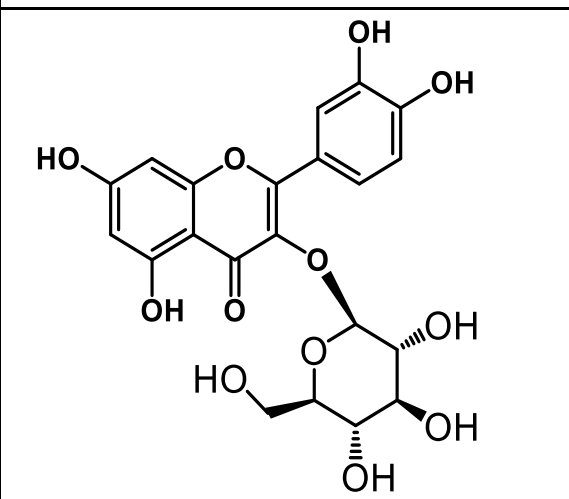	Sangzhi [230]	Mori ramutus	*Morus alba L.*
			Panasenoside	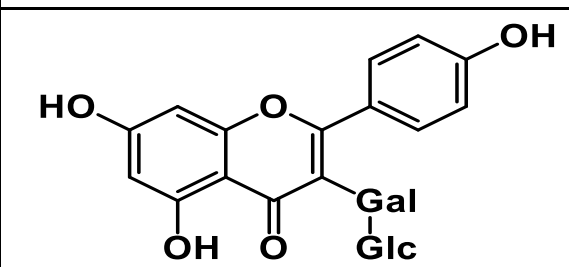	Sangshen [230]	Mulberry Fruits	*Morus alba L.*
		Flavonoid glucoside	Hyperoside	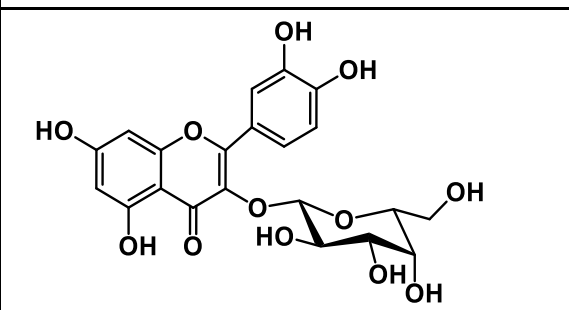	Huajiaoye	*Zanthoxylum bungeanum Maxim leaves*[231]	*Zanthoxylum bungeanum Maxim*
			Hyperoside<br>Isorhamnetin-3-O-galactoside<br>Quercitrin	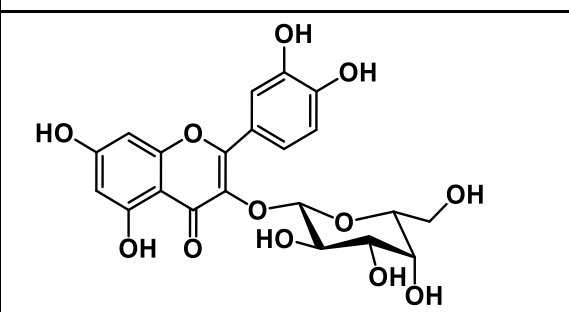	Shuiqin<br>Shuiqin	Oenanthe javanica[227]<br>Oenanthe javanica[227]	*Oenanthe javanica (Bl.) DC.*<br>*Oenanthe javanica (Bl.) DC.*

				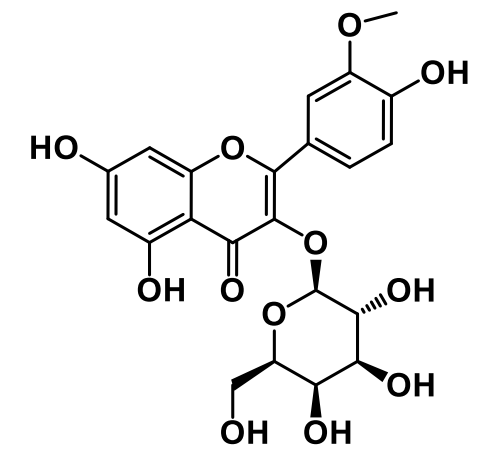 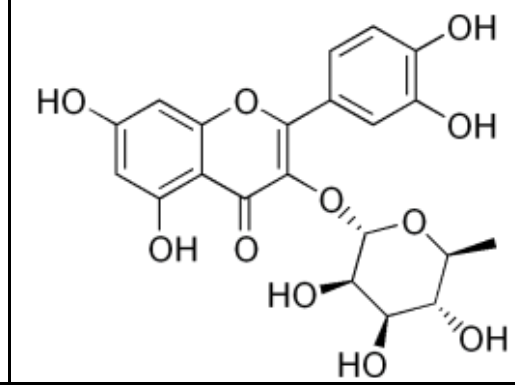			
			Isorhamnetin-3-O-galactoside<br>Quercitrin<br>Isorhamnetin-3-O-galactoside	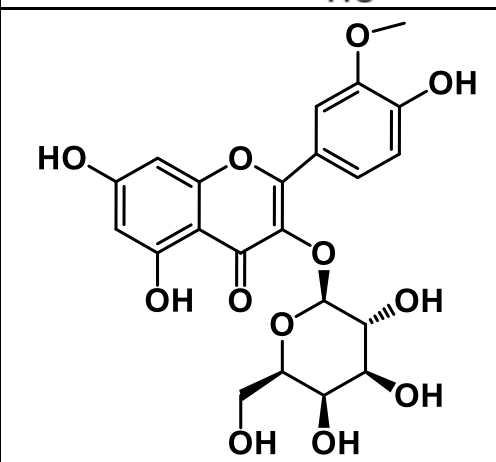 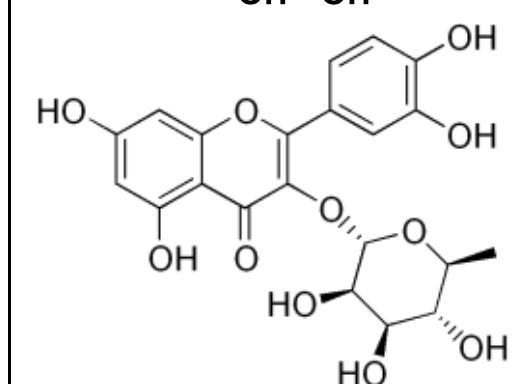 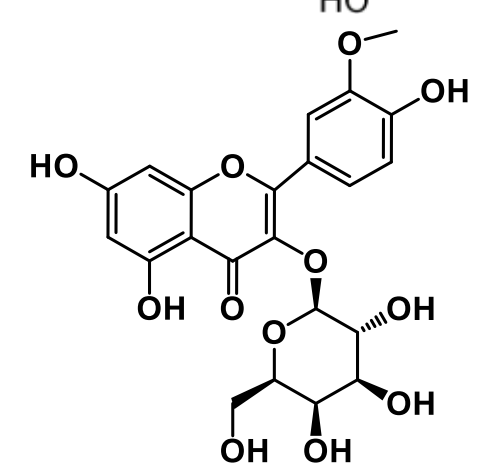	Shuiliao	Polygonum hydropiper	*Polygonum hydropiper L.*[232]
					Di'ercao	*Hypericum japonicum*	*Hypericum japonicum Thunb.*[232]

			Myricitrin<br>Quercetin 3-O-β-D-xylopyranoside	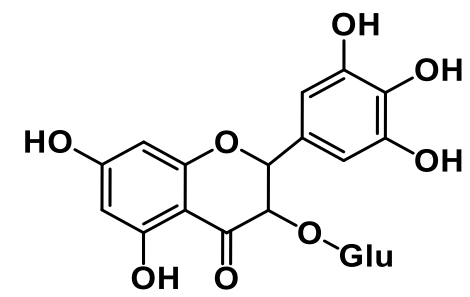<br>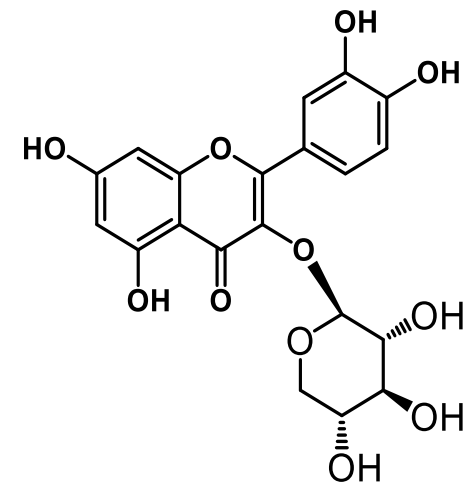	Hongguozai	Eugenia uniflora[233]	*Eugenia uniflora Linn.*
			Myricitrin<br>Quercetin 3-O-β-D-xylopyranoside<br>Quercetin 3-O-β-D-glucuronide	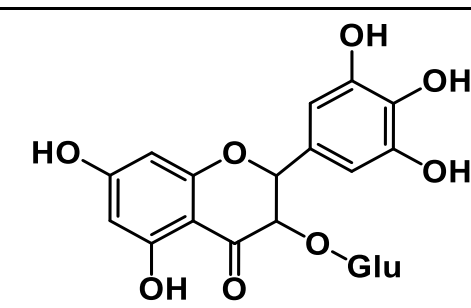<br>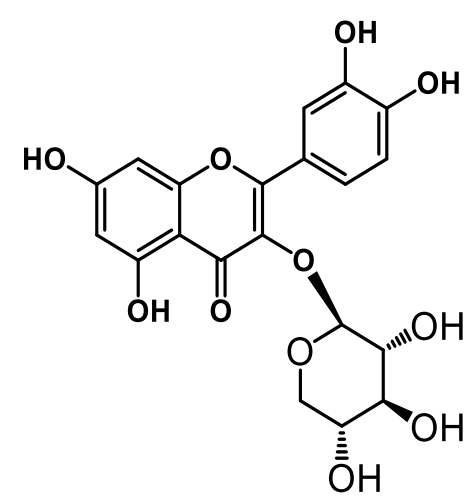<br>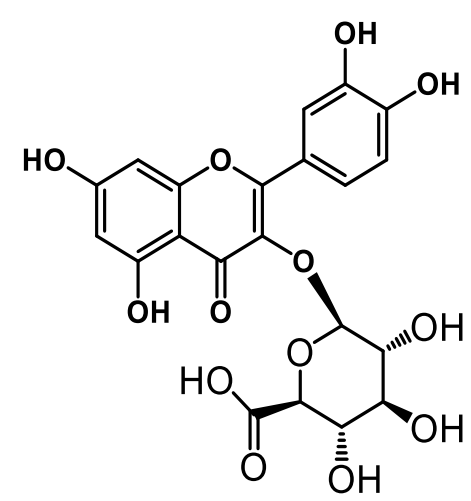	Huanghuacai	Hemerocallis fulva[234]	*Hemerocallis citrina Baroni*
					Gangbangui	Perforate fleeceflower	*Polygonum Perfoliatum L.*[235]

			Embeliaflavosides A	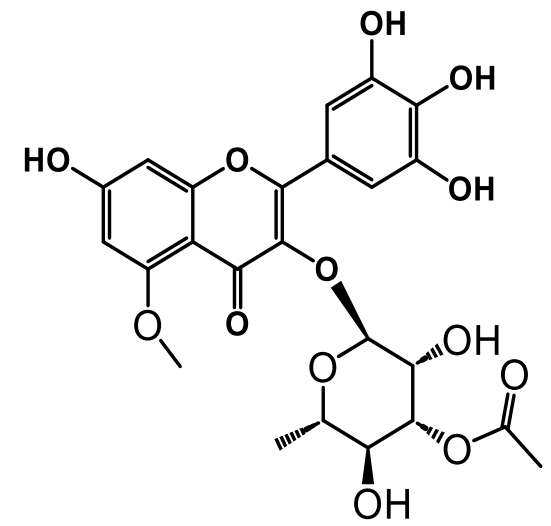	Suantentzi	Embelia ribes	*Embelia ribes*[236]
			Quercetin 3-gentiobioside	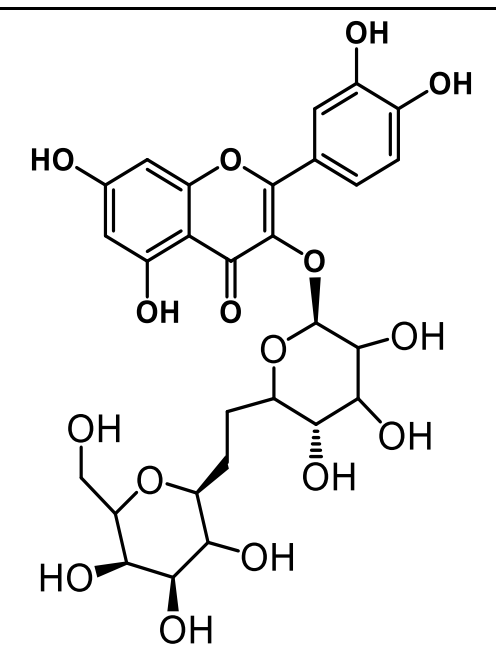	Changchunhua	Catharanthus roseus[237]	*Catharanthus roseus* （*L.*） *G. Don*
			luteolin-7-O-β-D-glucoside(cynaroside)	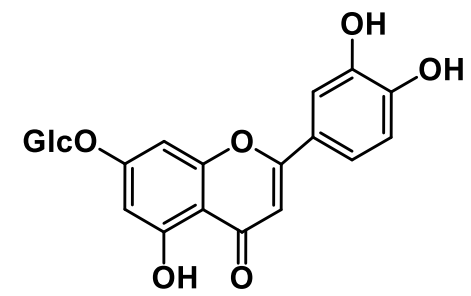	Suoyang	*Cynomorium songaricum Rupr.*	*Cynomorium songaricum Rupr.*[41]
			Scillavone A	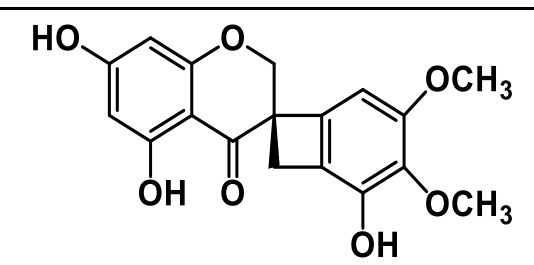	Mianzao’er	*Scilla scilloides*[238]	*Scilla scilloides*（*Lindl.*） *Druce*
			Aureusidin	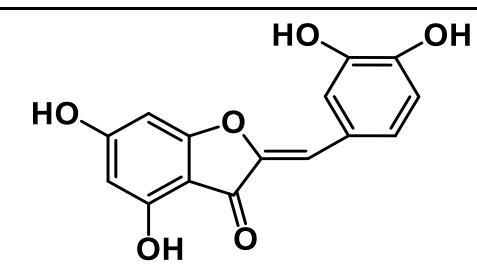	Sashenglaju	Helichrysum arenarium.[239]	Helichrysum arenarium

			carpusin	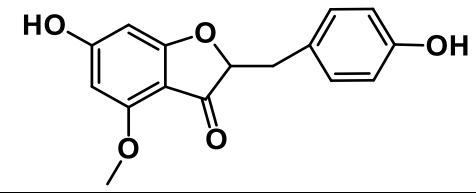	zangbiandahuang	*Rheum emodi*[240]	*Rheum australe D. Don*
		Homoisoflavone	Sulpuretin	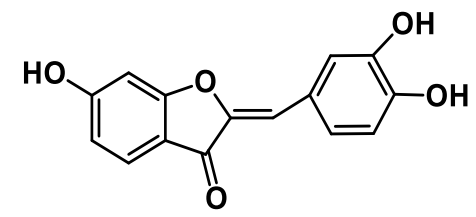	Qishu	*rhus verniciflua*[241]	*shaanxiense J. Z. Zhang et Z. Y. Shang*
		Aurone	Moracin C	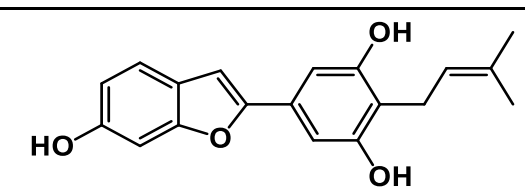	Sanbaipi	*Mori Cortex*	*Morus alba L.* [242]
			Iso-Moracin C	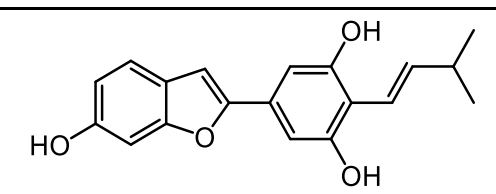	Sanbaipi	*Mori Cortex*	*Morus alba L.* [242]
			artoindonesianin O	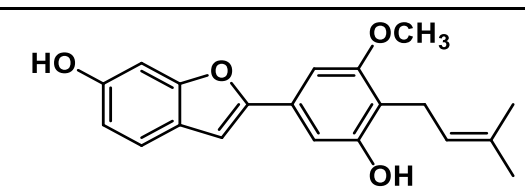	Sangshen	*mulberry;*	*Morus alba L.* [141]
5	2-Phenyl-benzofuran		lespedezavirgatol	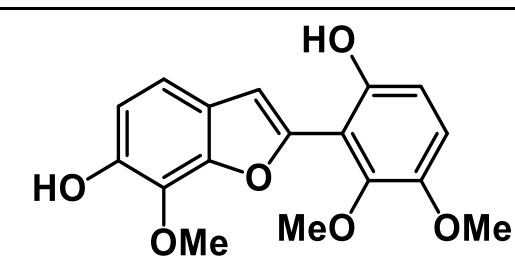	Xigenghuzhizi	*Lespedeza Virgata*	*Lespedeza Virgata*[243-245]
			lespedezavirgatal	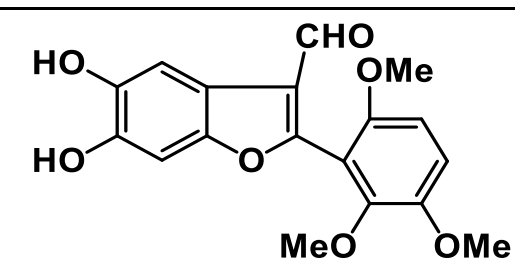	Xigenghuzhizi	*Lespedeza Virgata*	*Lespedeza Virgata,*[243, 244]
			Moracin M	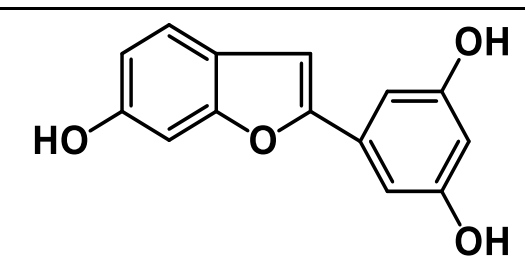	Sangbaipi	*Morus alba*	*Morus alba L.* [246-249]
			Moracin O	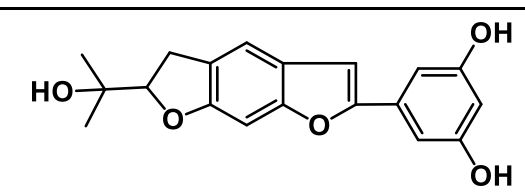	Sangbaipi	*Morus alba*	*Morus alba L*[246, 249]
			Moracin R	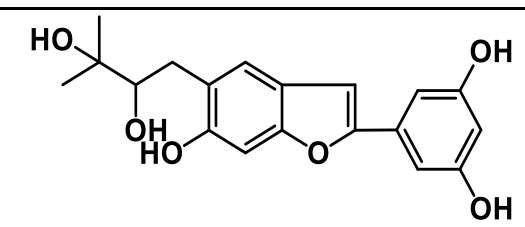	Sangbaipi	*Morus alba*	*Morus alba L.* [246, 248]

			Moracin S	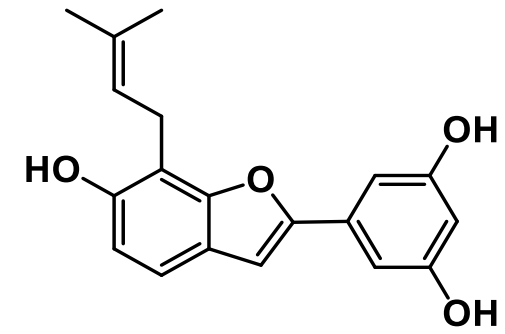	Sangbaipi	*Morus alba*	*Morus alba L.*[248, 249]
			Moracin T	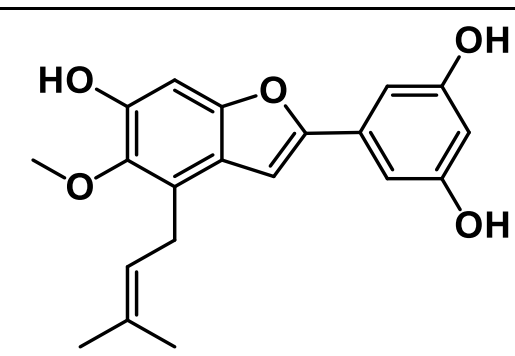	Sangbaipi	*Morus alba*	*Morus alba L.* [248]
			Moracin U	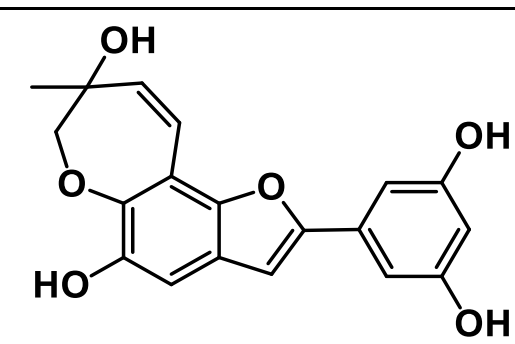	Sangbaipi	*Morus alba*	*Morus alba L.* [248]
			Moracin P	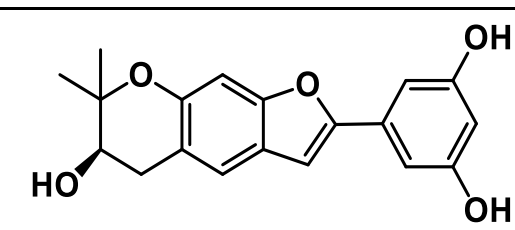	Sangbaipi	*Morus alba*	*Morus alba L.* [249]
			Moracin D	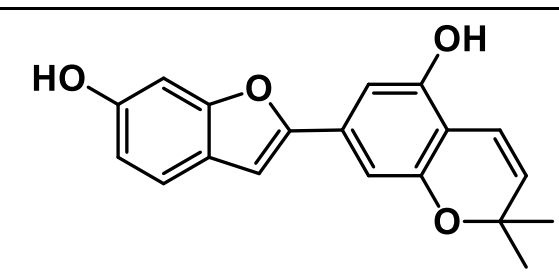	Sangbaipi	*Morus alba*	*Morus alba L.* [250]
			Oryzafuran	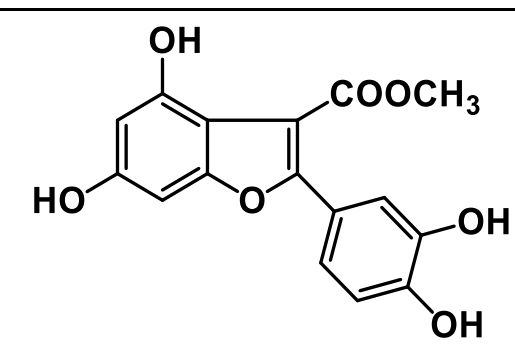	Heimi	*Black colored rice*	*Oryza sativa L.* [251]
			morunigrol C	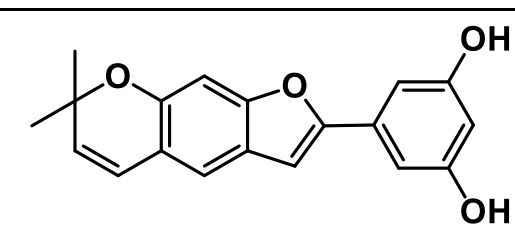	Heisangpi	*Dry bark of black mulberry*	*Morus nigra L*[98]

			albafurans A	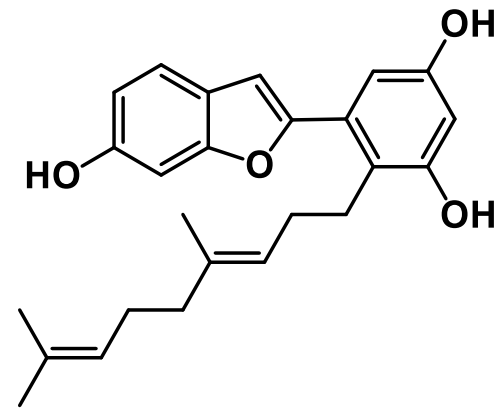	Heisangpi	*Dry bark of black mulberry*	*Morus nigra L*[98, 99]
			albafurans B	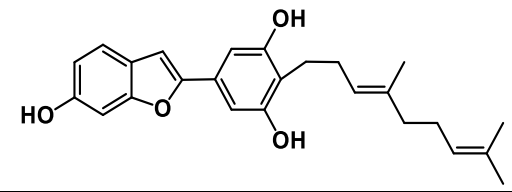	Heisangpi	*Dry bark of black mulberry*	*Morus nigra L.* [98, 99]
			mulberrofuran	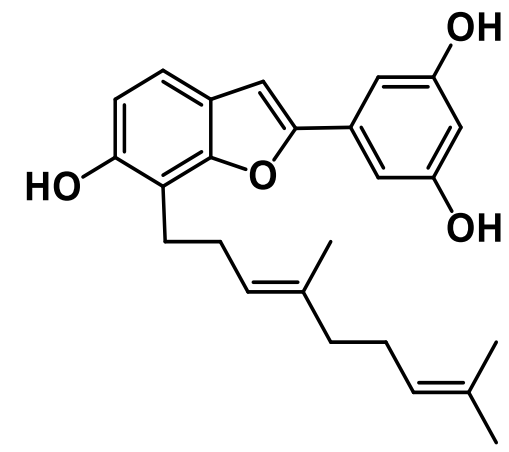	Heisangpi	*Dry bark of black mulberry*	*Morus nigra L.* [98]
			MoracinLM	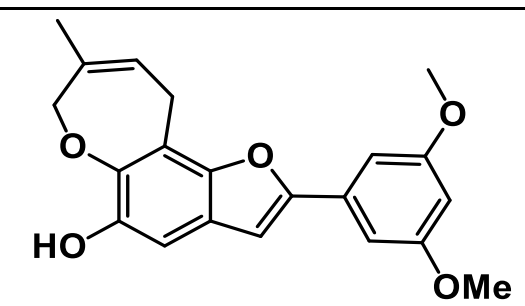	Shuweicao	*sage*	*the Twigs of Morus mesozygia*[252]
			moracin I	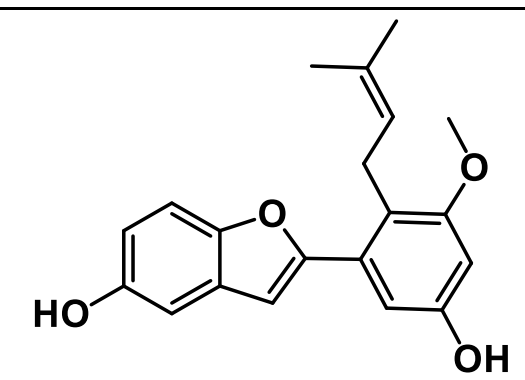	Shuweicao	*sage*	*the Twigs of Morus mesozygia*[252]
			Regiafuran A	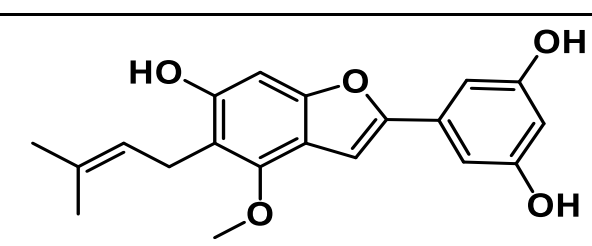	Dalvbingsang	*Odum*	*Chlorophora regiaA.Chev* [253]

			Isomangiferin	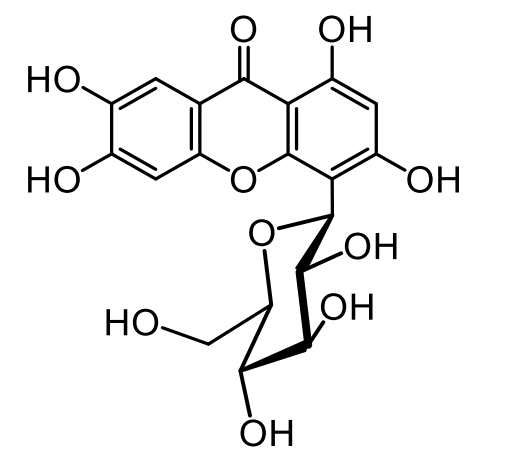	Zhimu	*Anemarrhenae Rhizoma*	Anemarrhena asphodeloides Bge. [254]
			Bellidifolin	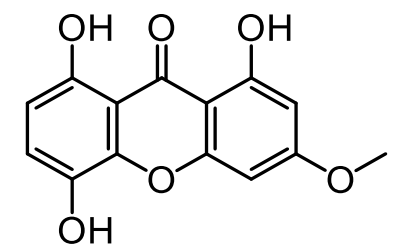	Zhangyacai	*Swertia*	*Swertia chirayita (Roxb.) H. Karsten* [255, 256]
			Swertianolin	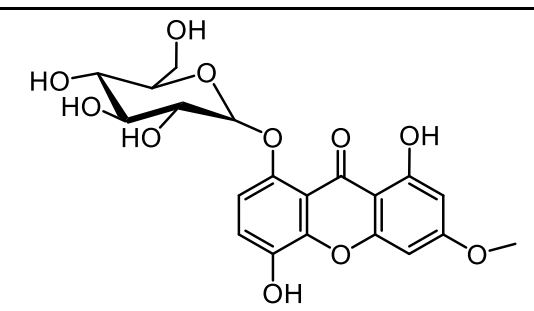	Longdancao	*Gentianae Radix et Rhizoma*	*Gentianella amarella ssp. acuta*[257]
6	Xanthone	Xanthone	Mangiferin	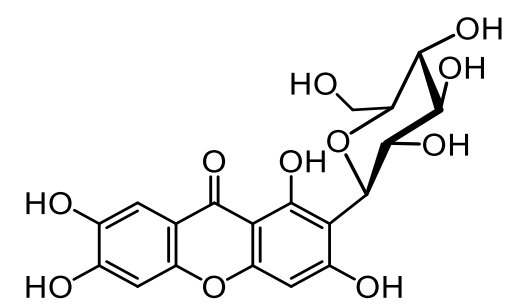	Mangguo	*Mango*	*Mangifera indica L.* [258],[259]
			1,3,5,8-Tetrahydroxyxanthone	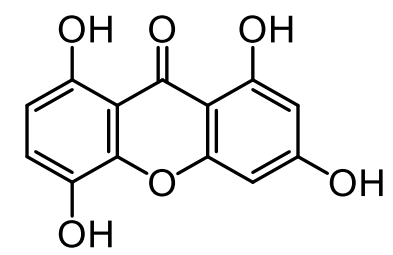	Zhangyacai	*Swertia bimaculata*	*Swertia bimaculata (Sieb. et Zucc.) Hook. Thoms.ex Clarke*[260, 261]
			Garcinone C	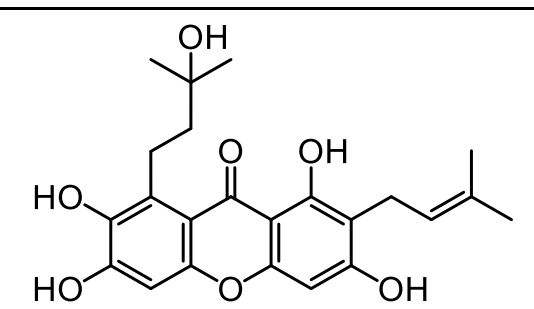	Linnanshanzhuzi	*Garcinia oblongifolia*	*Garcinia oblongifolia Champ. ex Benth.* [258, 262, 263]
			Norathyriol	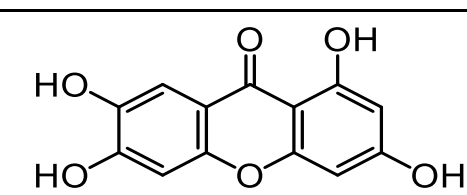	Zhimu	*Rhizoma anemarrhenae*	*Anemarrhena asphodeloides Bge.* [264, 265]

			Gartanin	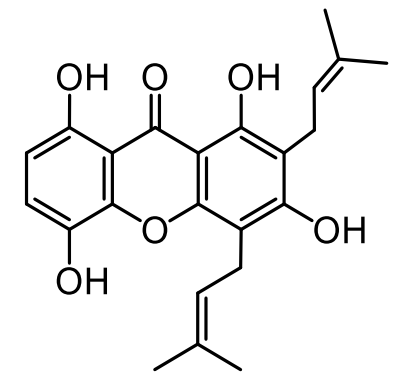	Shanzhu	*Mangosteen*	*Garcinia mangostana L.* [266-269]
			1,3,5,6-Tetrahydroxyxanthone	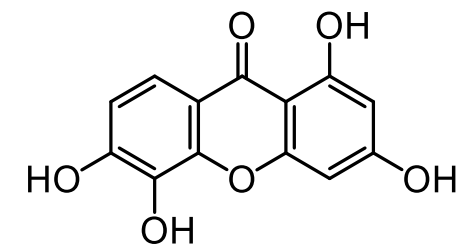	Tuoshuguo	*Cudrania*	*Cudrania tricuspidata (Carr.) Bur. ex Lavallee*[270-272]
			1,2,5-Trihydroxyxanthone	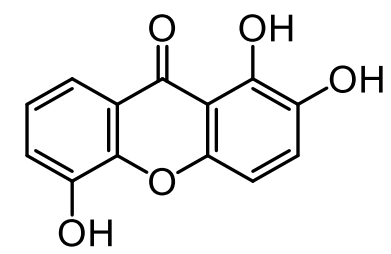	Tianjihuang	*Hypericum japonicum*	*Hypericum japonicum Thunb. ex Murray*[258, 273]
			Subelliptenone G	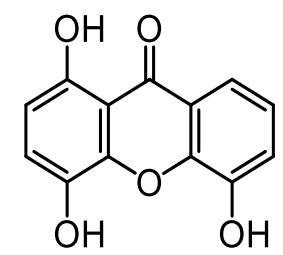	Tenghuang	*Garcinia hanburyi*	*Garcinia hanburyi Hook.f.* [258, 274, 275]
			1,5,6-Trihydroxyxanthone	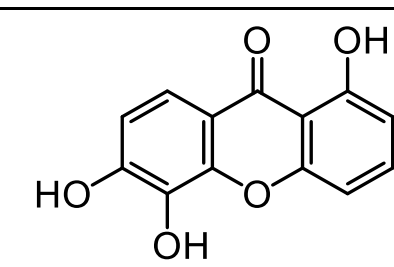	Tianjihuang	*Hypericum japonicum*	*Hypericum japonicum Thunb. ex Murray*[258, 276]
			Isojacareubin	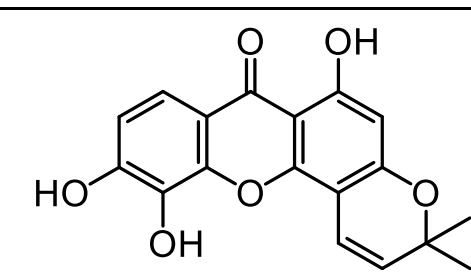	Tianjihuang	*Hypericum japonicum*	*Hypericum japonicum Thunb. ex Murray*[277]
			γ-Mangostin	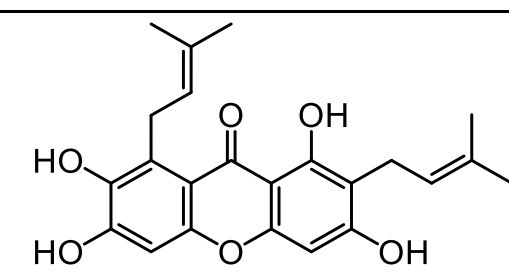	Shanzhu	*Mangosteen*	*Garcinia mangostana L.*[260, 278]
			Euxanthone	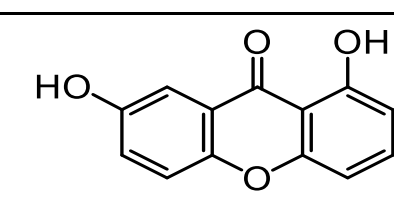	Yuanzhi	*Polygala*	*Polygala tenuifolia Willd*[279, 280]

			1,5-Dihydroxyxanthone	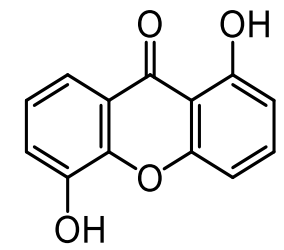	Honghouke	*Calophyllum*	*Calophyllum inophyllum L.*[260, 281, 282]
			α-Mangostin	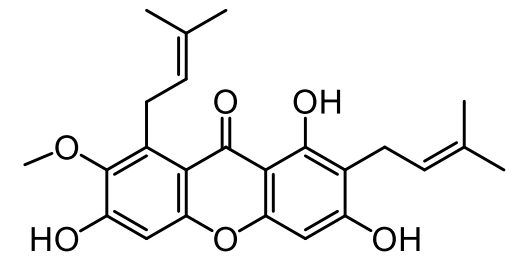	Shanzhu	*Mangosteen*	*Garcinia mangostana L.*[283, 284]
			Cowaxanthone B	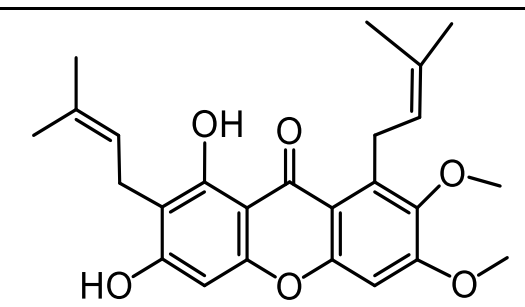	Yunnanshanzhuzi	*Garcinia cowa*	*Garcinia cowa Roxb.*[285, 286]
			2-Hydroxyxanthone	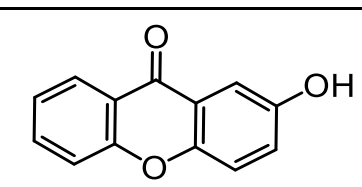	Honghouke	*Calophyllum*	*Calophyllum inophyllum L.*[258, 287].
			Garcinone D	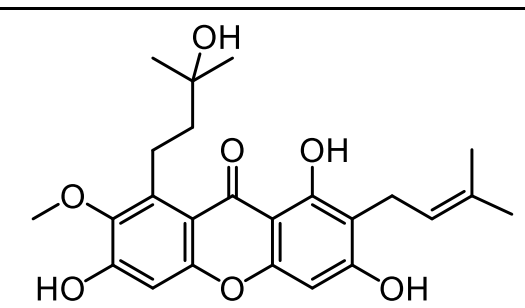	Shanzhu	*Mangosteen*	*Garcinia mangostana L.*[269, 288]
			Neomangiferin	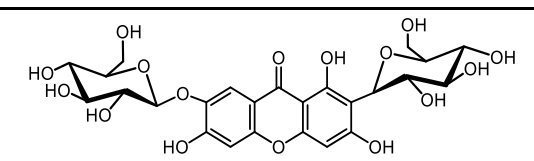	Zhimu	*Rhizoma anemarrhenae*	*Anemarrhena asphodeloides Bunge*[258, 289]
			8-Desoxygartanin	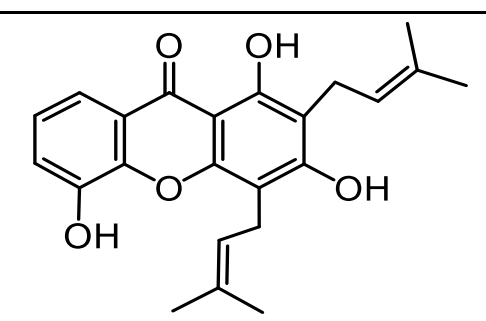	Shanzhu	*Mangosteen*	*Garcinia mangostana L.*[290, 291]
			7-O-methylmangiferin	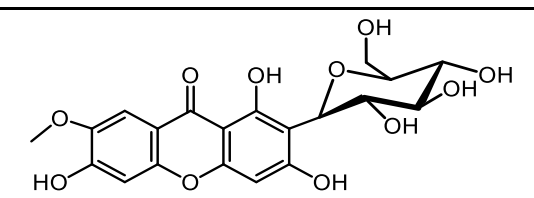	Shegan	*Rhizoma Belamcandae*	*Belamcanda chinensis L.*[258, 292]

			Cratoxylone	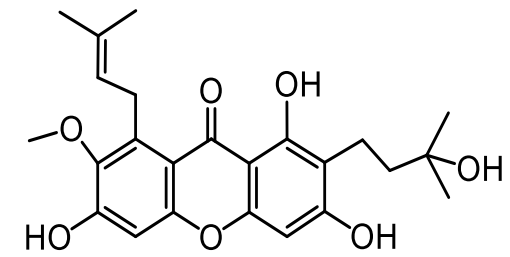	Huangniumu	*Cratoxylum cochinchinense*	*Cratoxylum cochinchinense (Lour.) Bl.*[260, 293]
			β-Mangostin	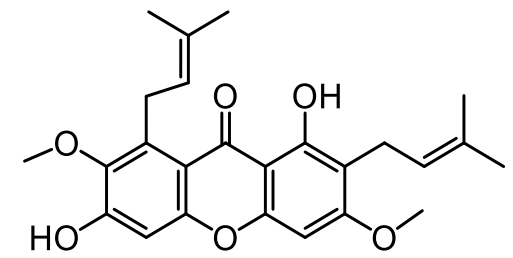	Yunnanshanzhuzi	*Garcinia cowa*	*Garcinia cowa Roxb.*[258, 284, 294]
			Lancerin	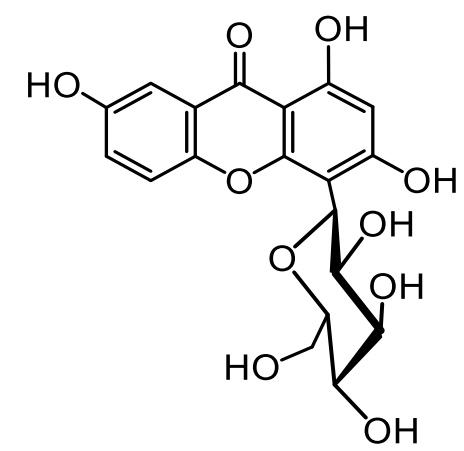	Luanyeyuanzhi	*Polygala sibirica*	*Polygala sibirica L.*[295, 296]
			1,2,3,7-Tetramethoxyxanthone	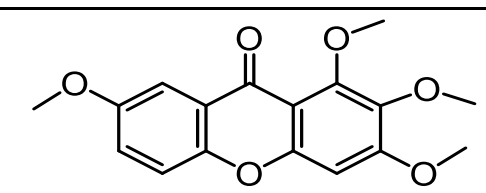	Luanyeyuanzhi	*Polygala sibirica*	*Polygala sibirica L.*[296, 297]
			Gentisin	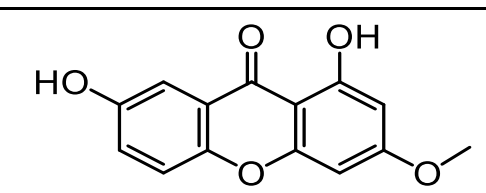	Qinjiao	*Gentiana macrophylla*	*Gentiana macrophμlla Pall.*[298, 299]
			1,3,7-trihydroxy-4-(1,1-dimethyl-2-propenyl)-5,6-(2,2-dimethylchromeno)-xanthone	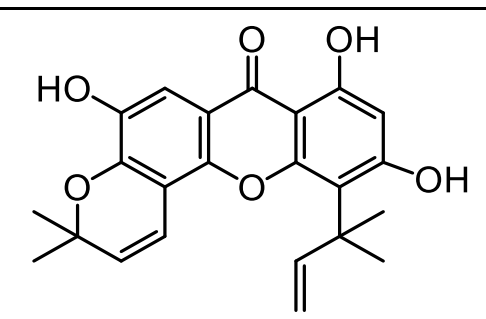	Tuomu	*Cudrania tricuspidata*	*Cudrania tricuspidata (Carr.) Bur. ex Lavallee*[300]
			Cudraxanthone C	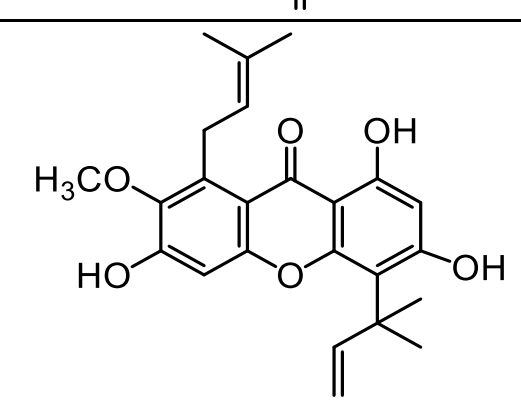	Tuomu	*Cudrania tricuspidata*	*Cudrania tricuspidata (Carr.) Bur. ex Lavallee*[300]

			Cudraxanthone D	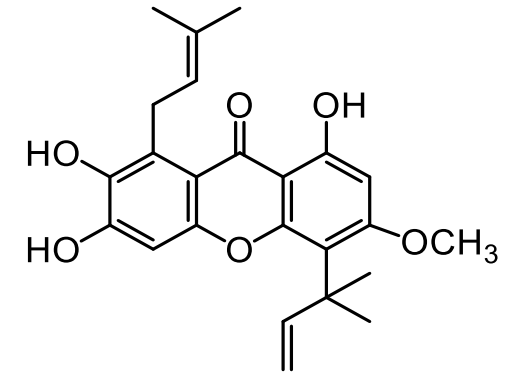	Tuomu	*Cudrania tricuspidata*	*Cudrania tricuspidata (Carr.) Bur. ex Lavallee*[300]
			Isocudraniaxanthone	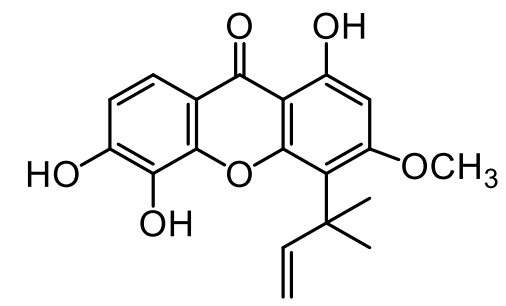	Tuomu	*Cudrania tricuspidata*	*Cudrania tricuspidata (Carr.) Bur. ex Lavallee*[300]
			1,3,6,7-tetrahydroxy-4-(1,1-dimethyl-2-propenyl)-8-prenylxanthone	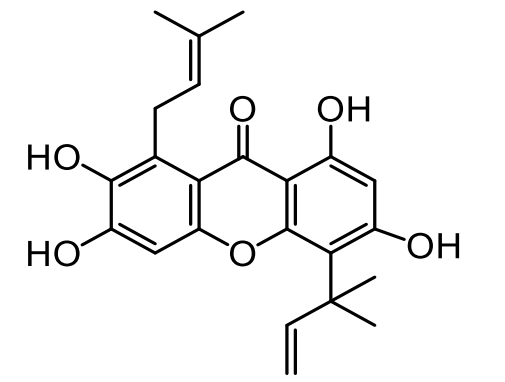	Tuomu	*Cudrania tricuspidata*	*Cudrania tricuspidata (Carr.) Bur. ex Lavallee*[300]
			Cudraxanthone L	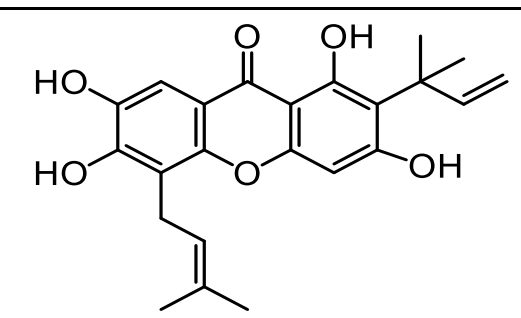	Tuomu	*Cudrania tricuspidata*	*Cudrania tricuspidata (Carr.) Bur. ex Lavallee*[300]
			Cudraxanthonec M	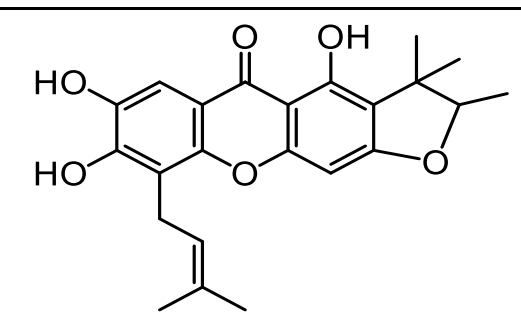	Tuomu	*Cudrania tricuspidata*	*Cudrania tricuspidata (Carr.) Bur. ex Lavallee*[300]
			Macluraxanthone B	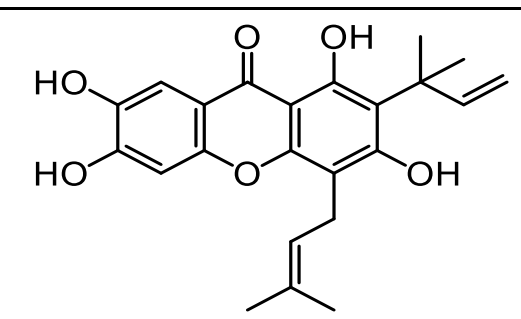	Tuomu	*Cudrania tricuspidata*	*Cudrania tricuspidata (Carr.) Bur. ex Lavallee*[300]
			1,7-Dihydroxy-2,3-methylenedioxyxanthone	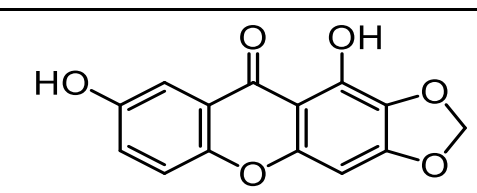	Huadaoshuilian	*Polygala fallax*	*Polygala fallax Hemsl.*[301, 302]

			Methylswertianin	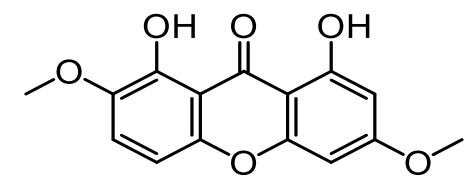	Shanpiaoercao	*Swertia punicea*	*Swertia punicea Hemsl.*[303-305]
			Flemiphilippinin A	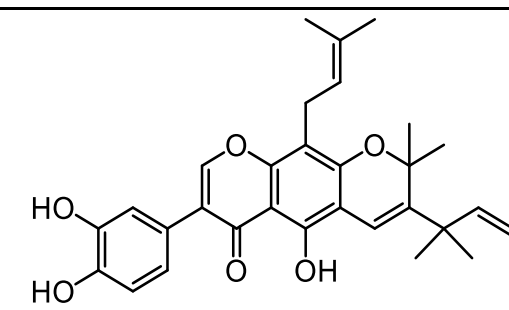	Qianjinba	*Flemingia philippinensis*	*Flemingia philippinensis Merr. et Rolfe*[306]
			Polygalaxanthone III	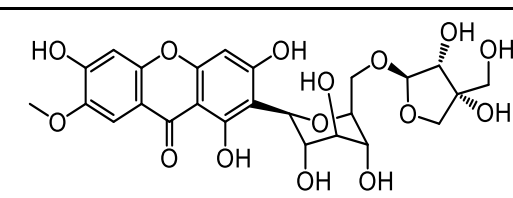	Yuanzhi	*Polygala tenuifolia*	*Polygala tenuifolia Willd.* [307, 308]
			Sibiricaxanthone B	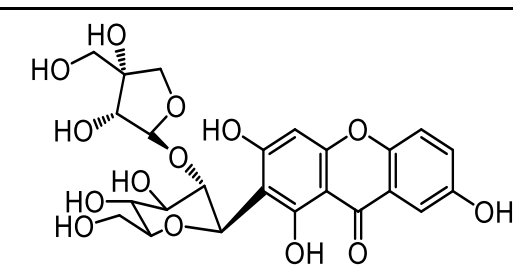	Yuanzhi	*Polygala tenuifolia*	*Polygala tenuifolia Willd.* [307, 308]
			Osthol	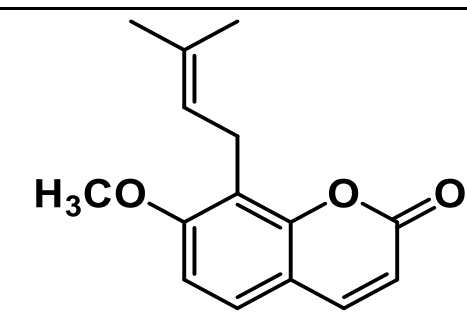	Putaoyou	*Citrus paradisi*	*Citrus paradisi* [309, 310]
			esculetin (6,7-dihydroxycoumarin)	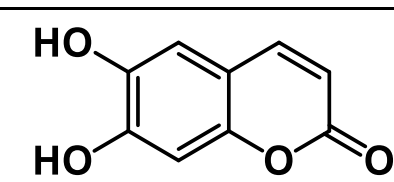	Qinpi	*Cortex Fraxini*	*Cortex Fraxini*[311, 312]
			Medicagol	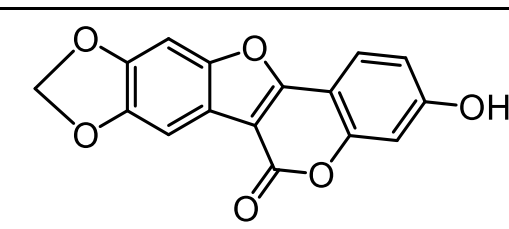	Huaimi	*Sophora japonica*	*Sophora japonica L.*[313, 314]

7	Coumarin		Pimpinellin	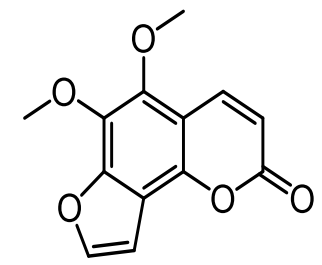	Baizhi	*Angelica dahurica*	*Angelica dahurica （Fisch. ex Hoffm. ）Benth. et Hook. f. ex Franch. et Sav* [315, 316]
			Wedelolactone	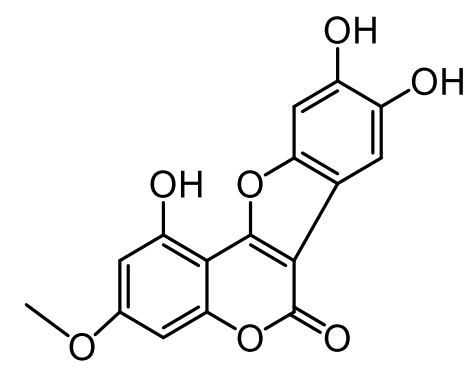	Pengqiju	*Wedelia calendulacea*	*Wedelia calendulacea (L.) Less.* [317-320]
			4-Hydroxycoumarin	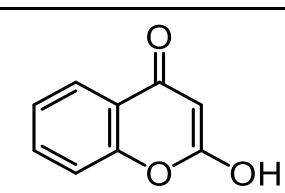	Yunxiang	*Ruta graveolens*	*Ruta graveolens L.*[321, 322]
			6-Methylcoumarin	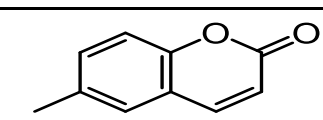	Yunxiang	*Ruta graveolens*	*Ruta graveolens L.* [321, 322]
			7-Hydroxycoumarin	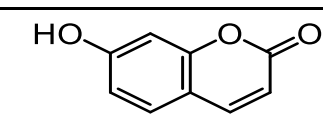	Yunxiang	*Ruta graveolens*	*Ruta graveolens L.* [321, 322]
			Peucedanol	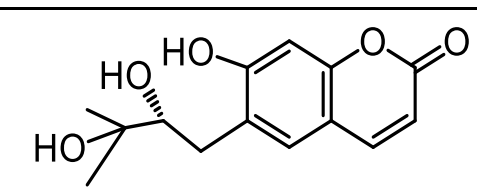	Baizhi	*Angelica dahurica*	*Angelica dahurica （Fisch. ex Hoffm. ）Benth. et Hook. f. ex Franch. et Sav* [323, 324]
			Scoparone	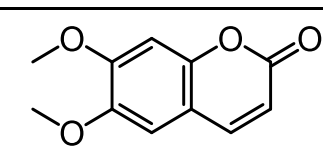	Yinchenhao	*Artemisiacapillaris*	*ArtemisiacapillarisThunb.*[310, 325]
			Psoralen	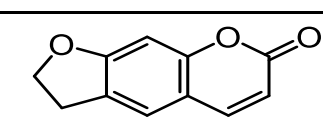	Buguzhi	*Psoralea corylifolia*	*Psoralea corylifolia L.* [326-328]
			Angelicin	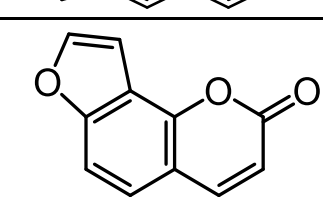	Buguzhi	*Psoralea corylifolia*	*Psoralea corylifolia L.*[329, 330]
			Bergaptol	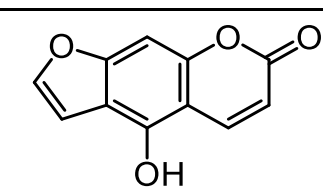	Baizhi	*Angelica dahurica*	*Angelica dahurica （Fisch. ex Hoffm. ）Benth. et Hook. f. ex Franch. et Sav*[331]

			Glycyrol	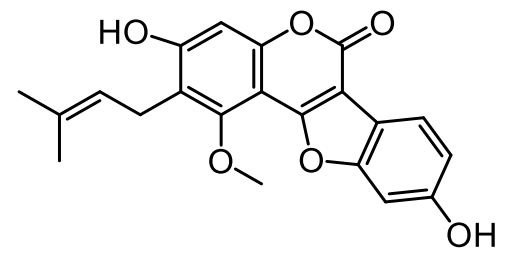	Gancao	*Glycyrrhiza uralensis*	*Glycyrrhiza uralensis Fisch*[331]
			Fraxetin	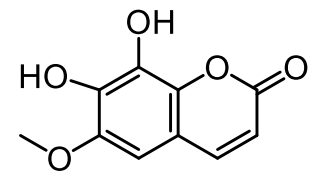	Duxiang	*Ledum palustre*	*Ledum palustre L.* [332, 333]
			Osthole	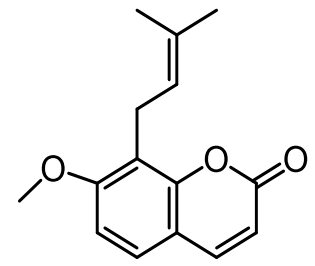	Duhuo	*Angelica pubescens*	*Angelica pubescens Maxim.f. biserrata Shan et Yuan* [334, 335]
			Esculetin	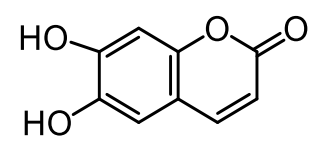	Qinpi	*Fraxinus rhynchophylla*	*Fraxinus rhynchophylla Hance* [336-338]
			Heraclenol	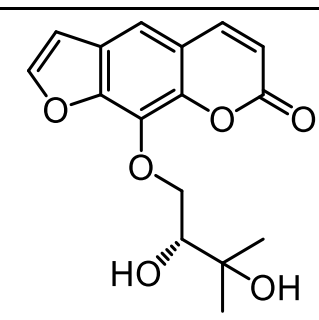	Baizhi	*Angelica dahurica*	*Angelica dahurica （Fisch. ex Hoffm. ）Benth. et Hook. f. ex Franch. et Sav* [338, 339]
			Scopoletin	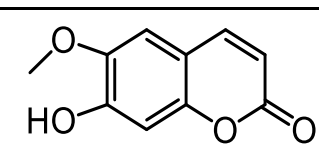	Bajitian	*Morinda citrifolia*	*Morinda citrifolia L.*[340-342]
			Scopolin	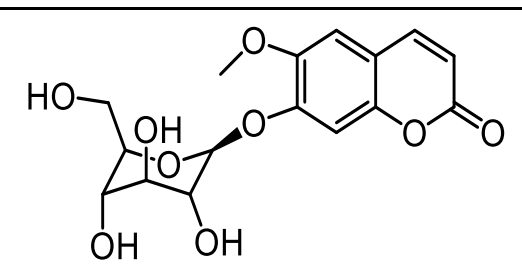	Yinchenhao	*Artemisia capillaris*	*Artemisia capillaris Thunb.* [343, 344]
			Columbianadin	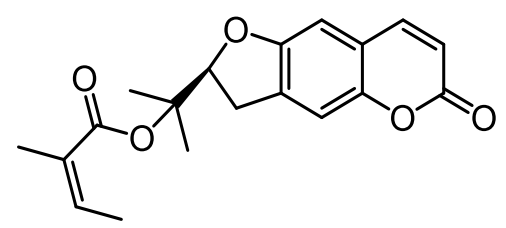	Duhuo	*Angelicae Pubescentis*	*Angelicae Pubescentis Radix*[345]
			Columbianetin	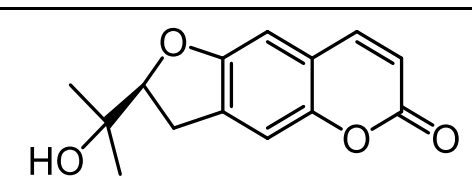	Duhuo	*Angelicae Pubescentis*	*Angelicae Pubescentis Radix*[346, 347]

			Bergamottin	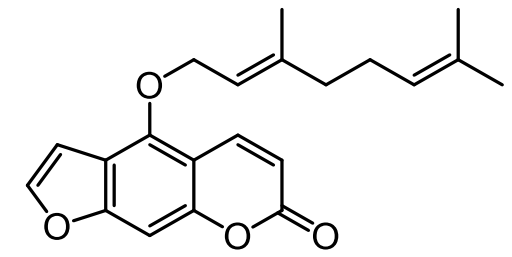	Ningmeng	*Lemon*	*Citrus limon (L.) Burm. f.*[348, 349]
			Auraptene	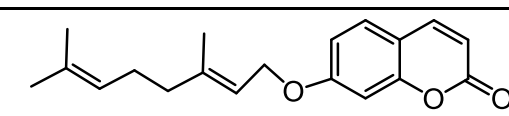	Zhishi	*Citrus aurantium*	*Citrus aurantium L.* [350, 351]
			Xanthotoxin	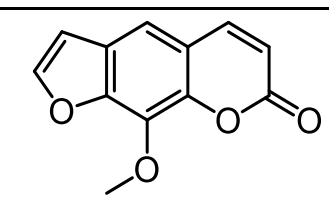	Baizhi	*Angelica dahurica*	*Angelica dahurica （Fisch. ex Hoffm. ）Benth. et Hook. f. ex Franch. et Sav* [352, 353]
			Xanthotoxol	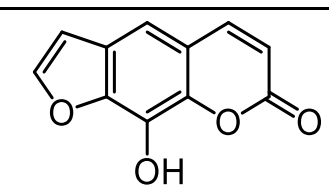	Baizhi	*Angelica dahurica*	*Angelica dahurica （Fisch. ex Hoffm. ）Benth. et Hook. f. ex Franch. et Sav*[331]
			Esculin	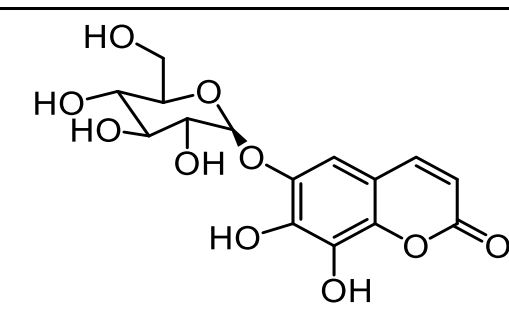	Qinpi	*Fraxinus rhynchophylla*	*Fraxinus rhynchophylla Hance*[323, 354]
			Isofraxidin	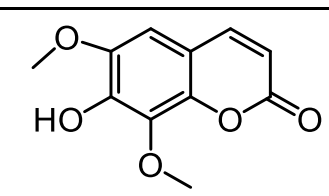	Zhongjiefeng	*Sarcandra glabra*	*Sarcandra glabra （Thunb. ）Nakai* [355-357]
			Skimmin	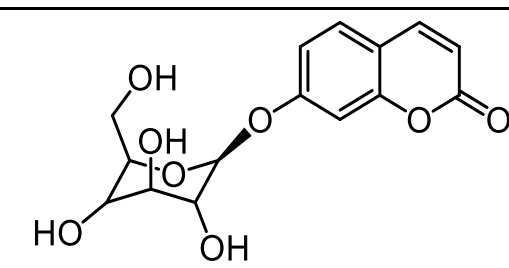	Baizhi	*Angelica dahurica*	*Angelica dahurica （Fisch. ex Hoffm. ）Benth. et Hook. f. ex Franch. et Sav* [358-360]
			Acteoside	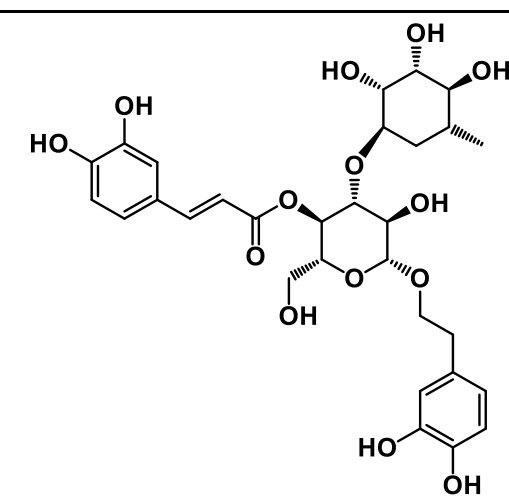	Xuanshen	*Scrophulariae Radix*	*Scrophularia ningpoensis*[361]

			Nothoapiole	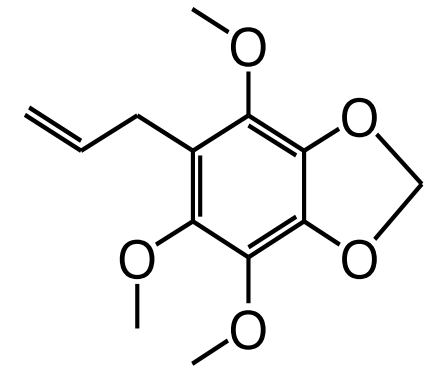	Heixiangcai	Carum bulbocastanum[362, 363]	Carum bulbocastanum
			3-(4-hydroxy-3-methoxy-phenyl)propane-1,2-diol	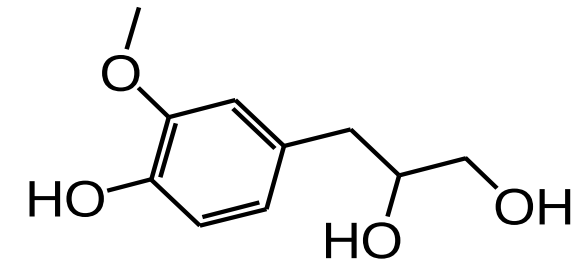	Duoxiangguo	Pimenta dioica[364]	Pimenta dioica
8	Phenylpropanoids	Phenylpropanoid	Taraxafolin B	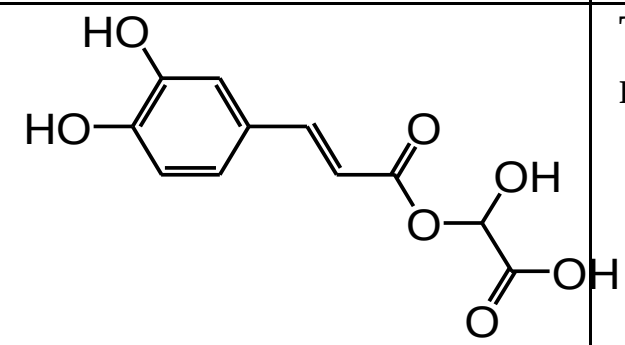	Tanwanpugongyin	*Taraxacum formosanum*[365]	*Taraxacum formosanum*
			Boropinic acid	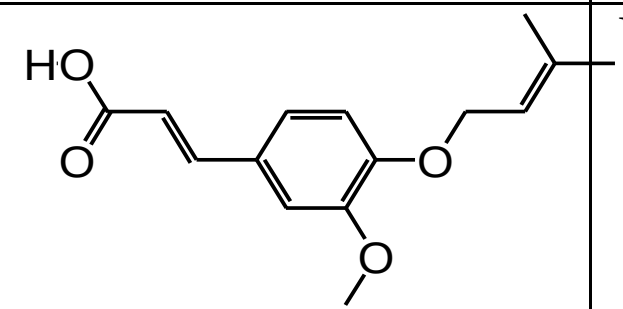	Yuyepengsha	*Boronia pinnata*[366]	*Boronia pinnata*
			Erythro-1'-(4-methoxyphenyl)-propan-1',2'-diol	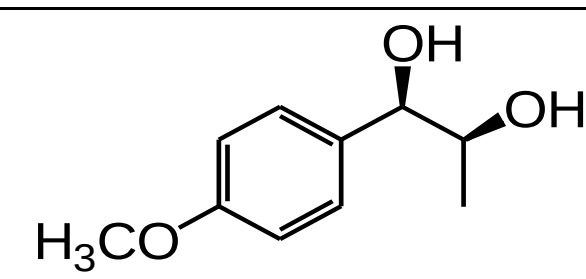	Huiqin	Pimpinella aurea[367]	*Pimpinella anisum L.*
			Erythro-1'-[4-(sec-butyl)-phenyl]-propan-1',2'-diol	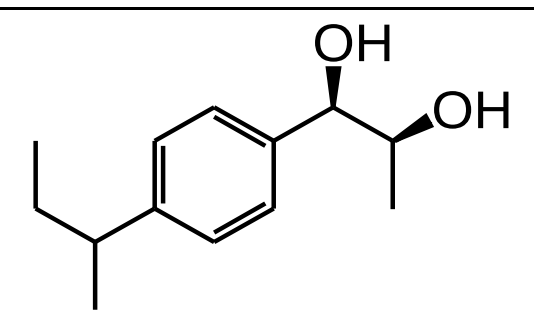	Huiqin	Pimpinella aurea	*Pimpinella anisum L.*[367]
			Guaiacylglycerol	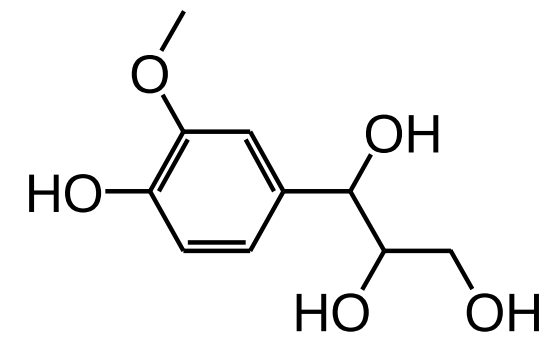	Bajiao	Musa basjoo	*Musa basjoo*[368]

			Clinopodic acid A	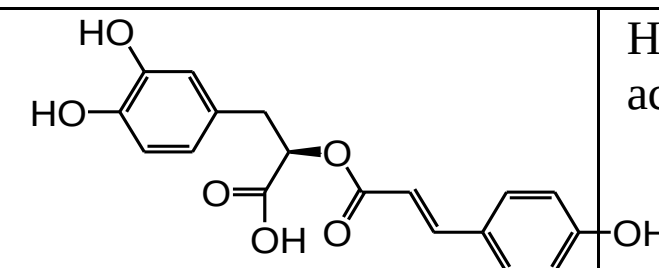	Huanghuaxiangchacai	Isodon lophanthoides var. graciliflorus[369]	*Isodon lophanthoides var. graciliflorus*
			Clinopodic acid B	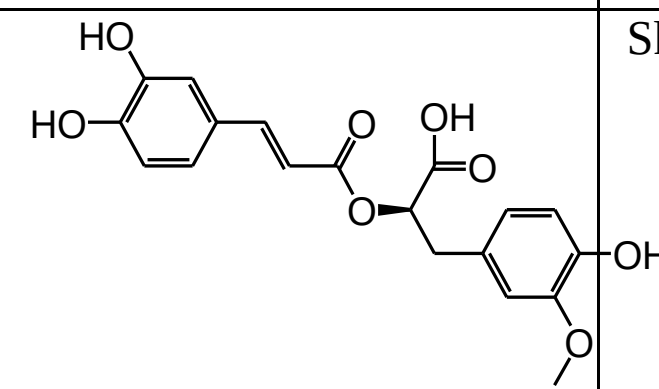	Shuweicao	Salvia palaestina	*Salvia palaestina L.*[370]
			Ballotetroside	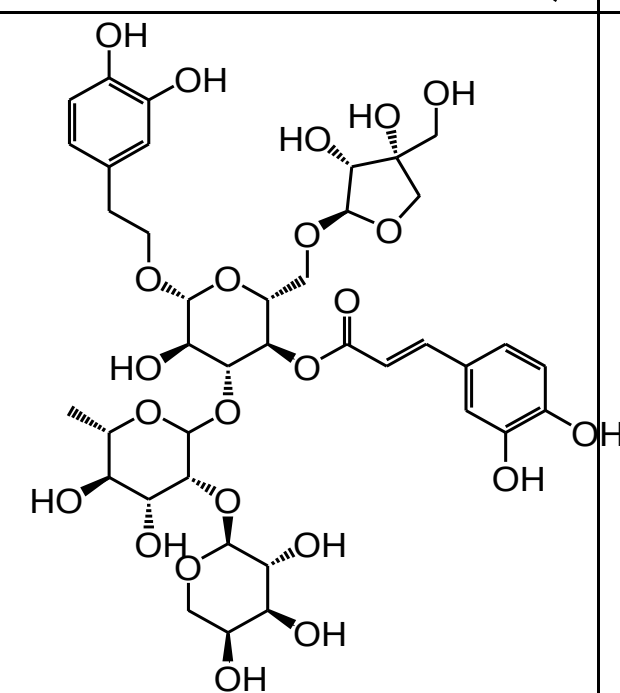	Ouxiazhicao	*Marrubium vulgare*	*Marrubium vulgare L.* [371, 372]
			L-O-Caffeoylhomoserine	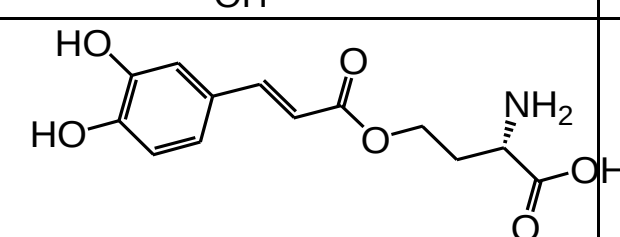	Jiaguojue	Matteuccia struthiopteris	*Matteuccia struthiopteris (L.) Todaro*[373]
			Caffeoyl malic acid	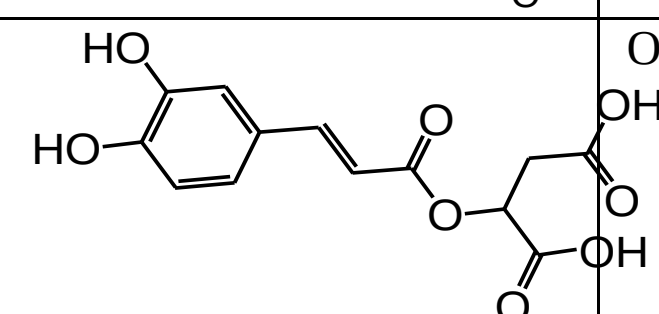	Ouxiazhicao	*Marrubium vulgare*	*Marrubium vulgare L.*[372, 374]
		Phenylallyl	Cistanoside F	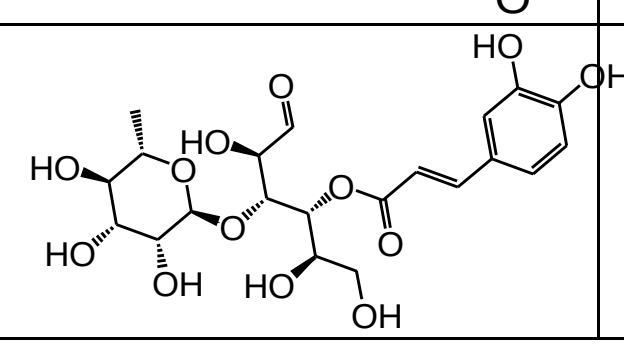	Shengdihuang	Rehmannia glutinosa	*Rehmannia glutinosa (Gaetn.) Libosch. ex Fisch. et Mey.*[375]

			crenatoside	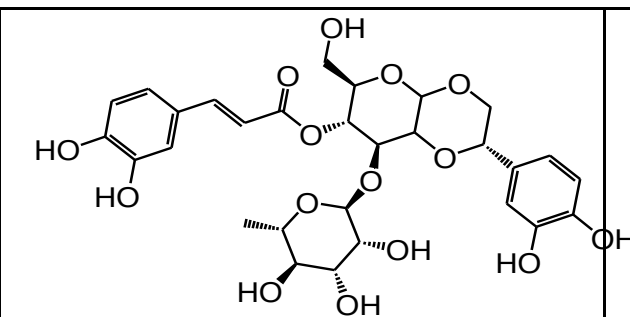	Liedan	Orobanche coerulescens	*Orobanche coerulescens Steph.*[376]
			Ehrenoside	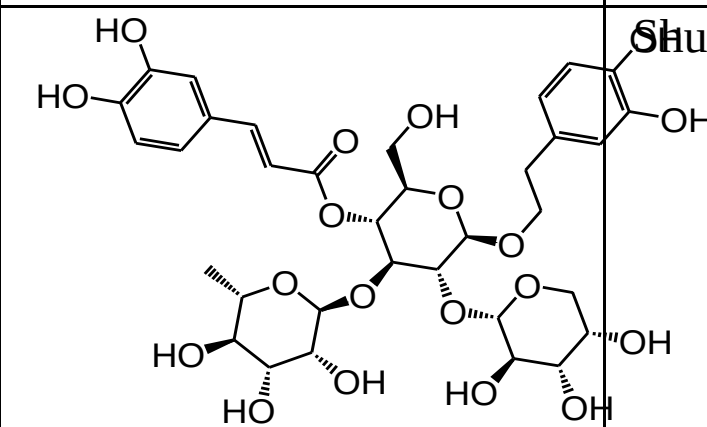	Shuchipopona	Veronica pectinate var. glandulosa[377]	*Veronica pectinate var. glandulosa*
			Ethyl rosmarinate	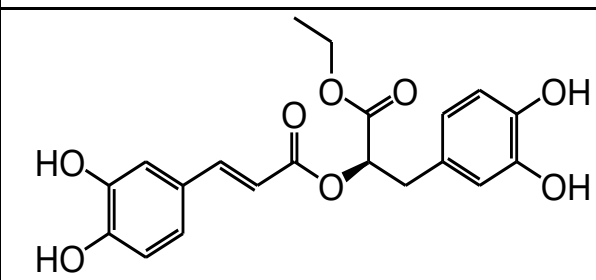	Changzhuliulicao	Lindelofia stylosa	*Lindelofia stylosa (Kar. et Kir.) Brand*[378]
			Forsythoside B	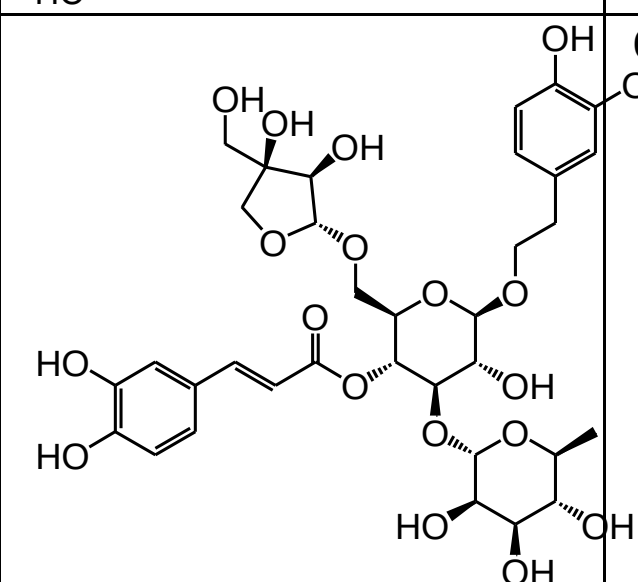	Ouxiazhicao	*Marrubium vulgare*	*Marrubium vulgare L.*[374]
			Isoacteoside	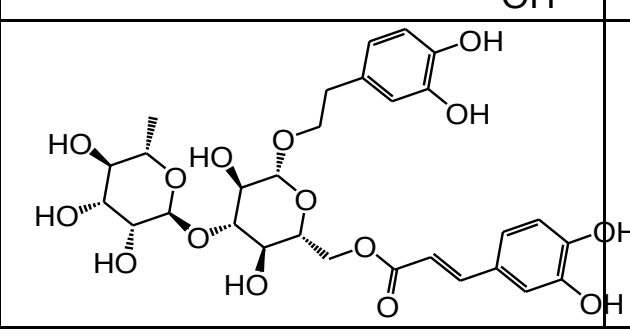	Shengdihuang	Rehmannia glutinosa	*Rehmannia glutinosa (Gaetn.) Libosch. ex Fisch. et Mey.*[379]

			Ligupurpuroside A	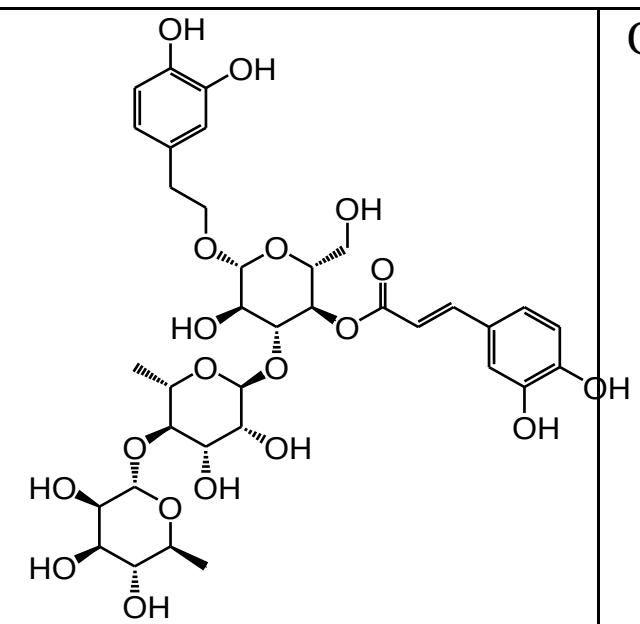	Cuzhuangnvzhen	Ligustrum robustum	*Ligustrum robustum (Roxb.) Blume*[380]
			Ligupurpuroside B	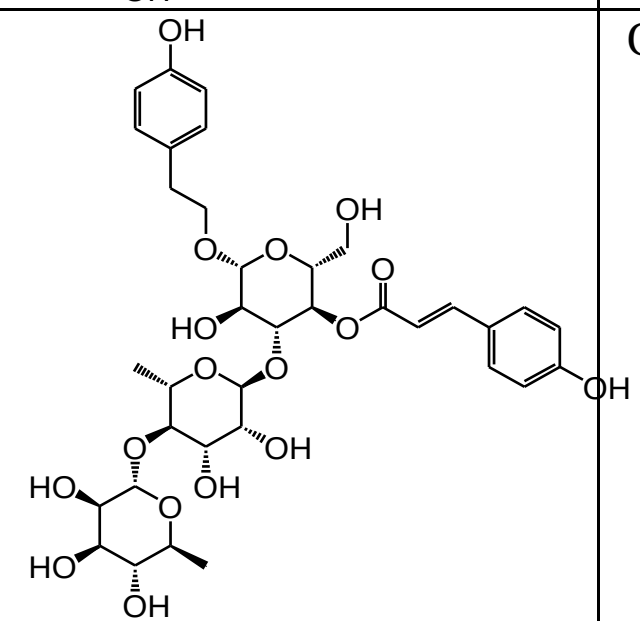	Cuzhuangnvzhen	Ligustrum robustum	*Ligustrum robustum (Roxb.) Blume*[380]
			Ligurobustoside I	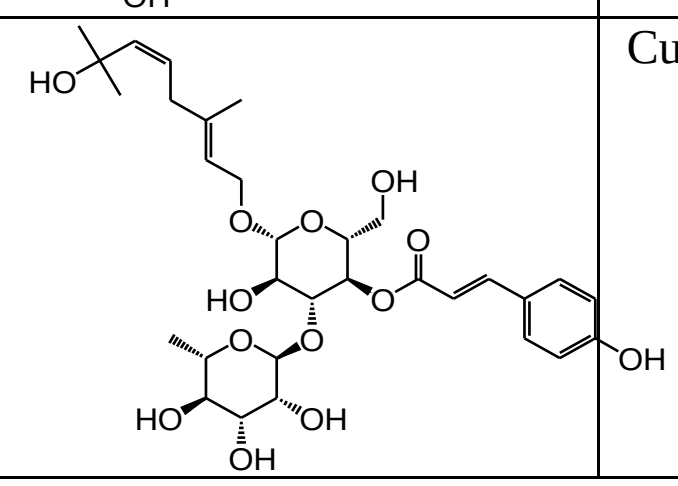	Cuzhuangnvzhen	Ligustrum robustum	*Ligustrum robustum (Roxb.) Blume*[380]

			Ligurobustoside N	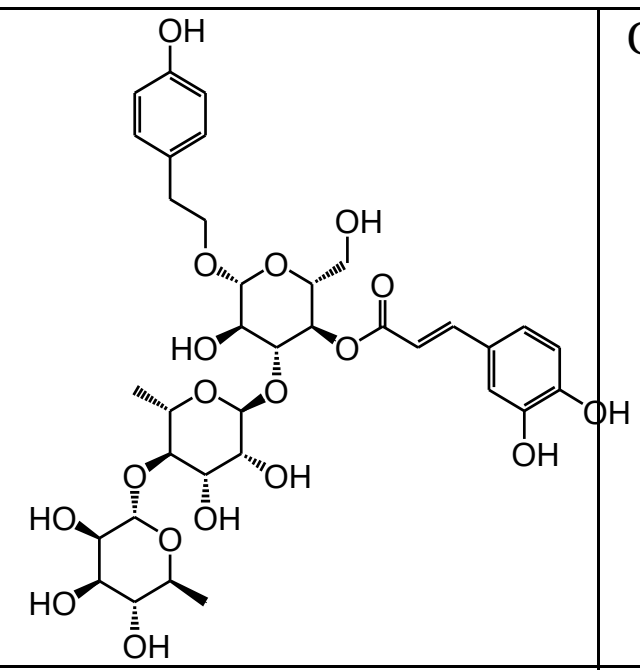	Cuzhuangnvzhen	Ligustrum robustum	*Ligustrum robustum (Roxb.) Blume*[380]
			Lithospermic acid	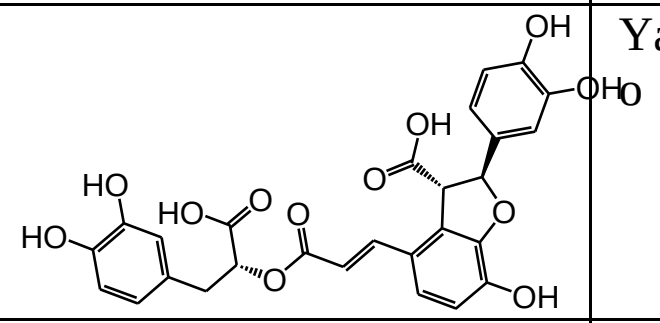	Yaoyongniusheca o	Anchusa officinalis	*Anchusa officinalis L.*[381]
			Osmanthuside B	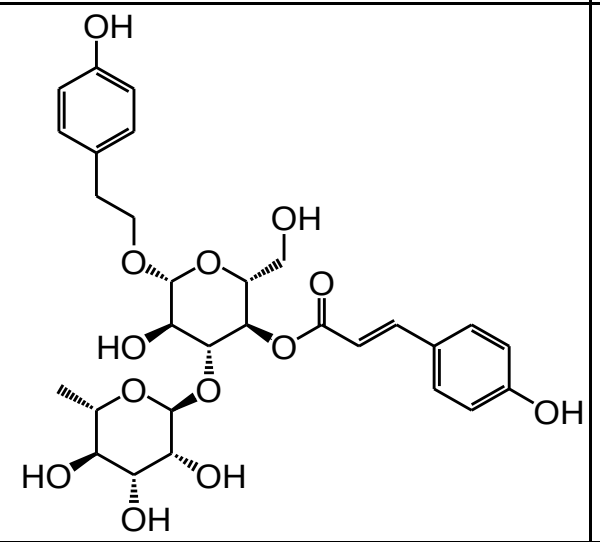	Cuzhuangnvzhen	Ligustrum robustum	*Ligustrum robustum (Roxb.) Blume*[381]
			Osmanthuside B6	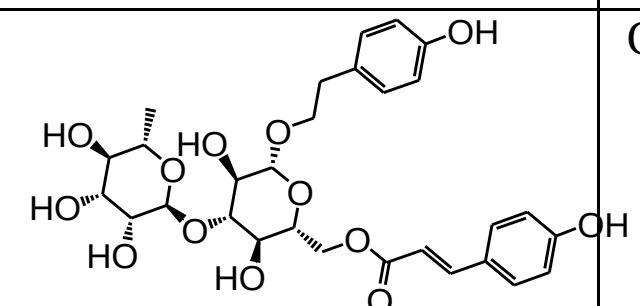	Cuzhuangnvzhen	Ligustrum robustum	*Ligustrum robustum (Roxb.) Blume*[381]
			Plantainoside A	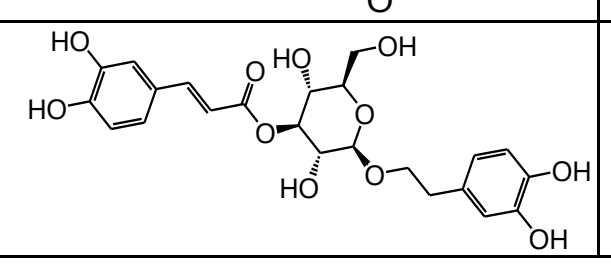	Cheqian	Plantago asiatica	*Plantago asiatica L.*[382]

			Plantainoside B	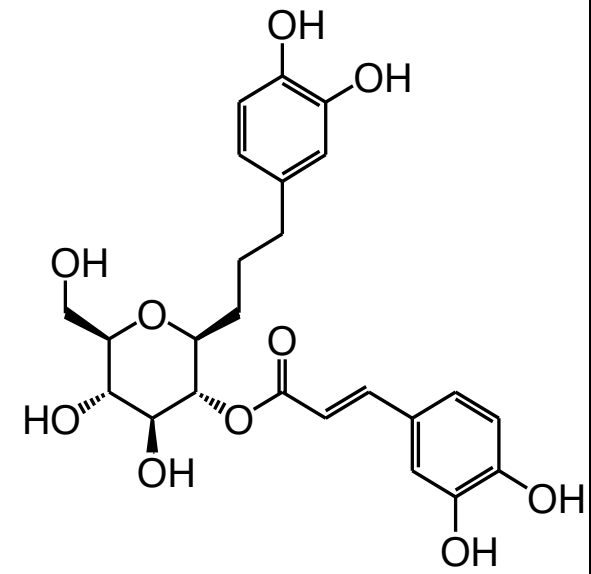	Cheqian	Plantago asiatica	*Plantago asiatica L.*[382]
			Plantainoside D	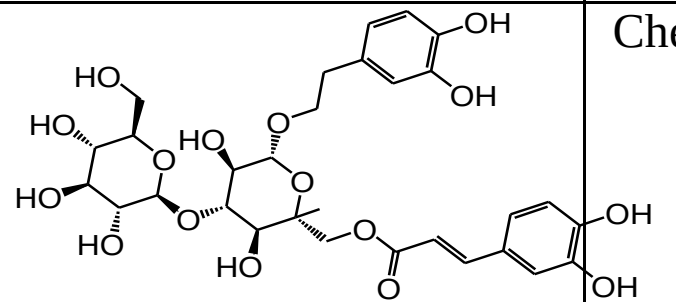	Cheqian	Plantago asiatica	*Plantago asiatica L.*[382]
			Scroside A	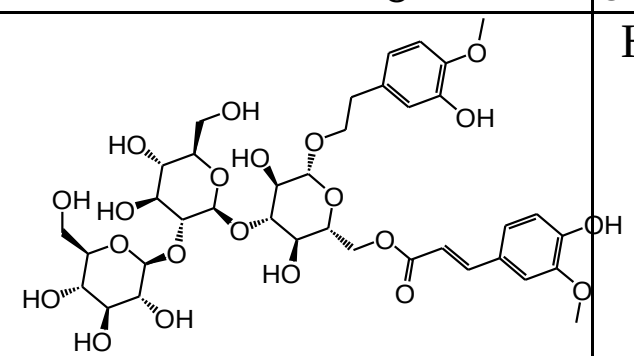	Huahuangliang	Picrorhiza scrophulariiflora	*Picrorhiza scrophulariiflora Pennell*[383]
			Verpectoside A	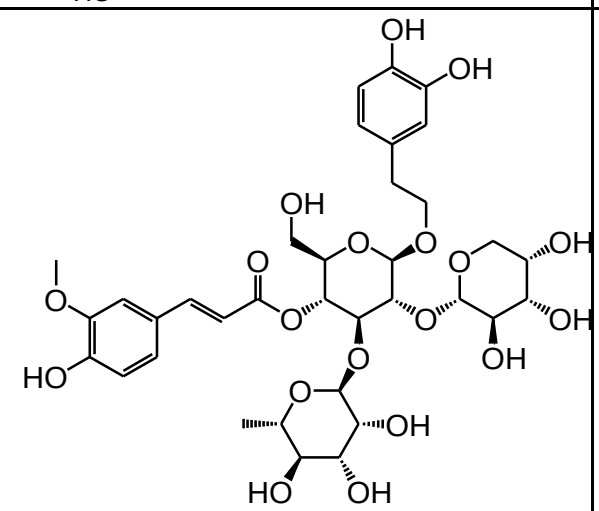	Shuchipopona	*Veronica pectinate var.glandulosa*[377]	*Veronica pectinate var.glandulosa*
			Verpectoside B	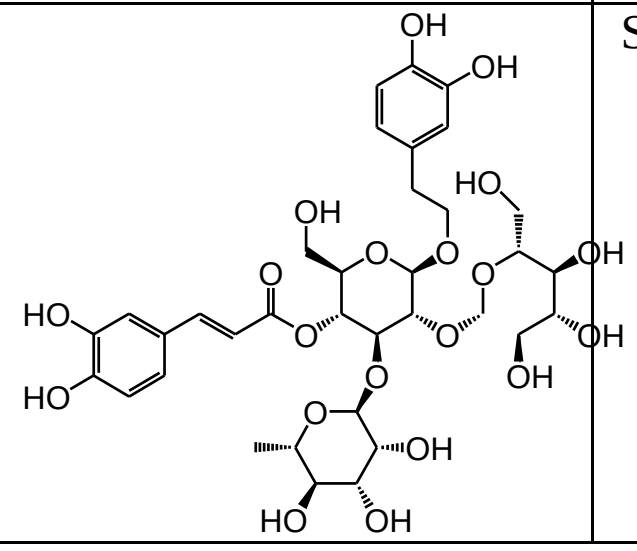	Shuchipopona	*Veronica pectinate var.glandulosa*[377]	*Veronica pectinate var.glandulosa*

			Verpectoside C	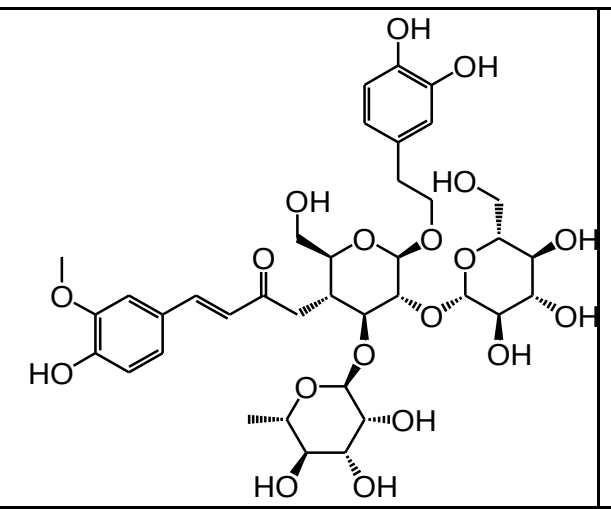	Shuchipopona	*Veronica pectinate var.glandulosa*[377]	*Veronica pectinate var.glandulosa*
			3-O-caffeoylquinic acid	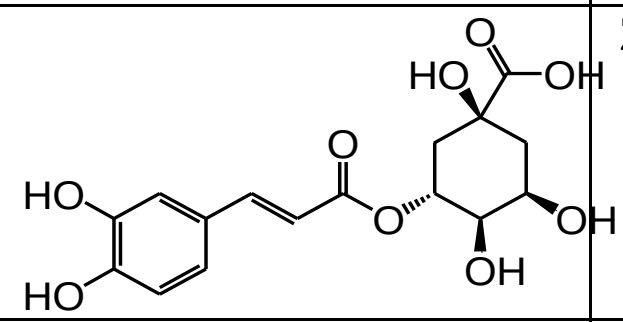	Zhimuchai	Loropetalum chinense	*Loropetalum chinense (R. Brown) Oliv.*[384, 385]
			4-O-caffeoylquinic acid	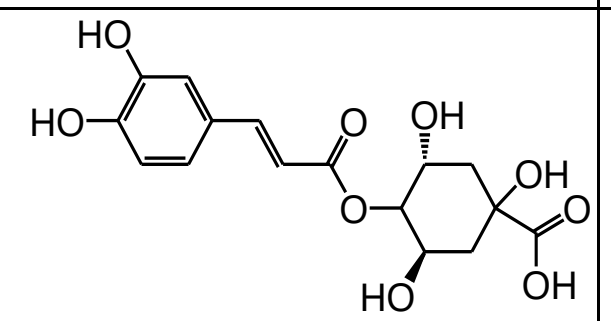	Sanshu	Morus alba	*Morus alba L.*[385, 386]
			5-O-caffeoylquinic acid	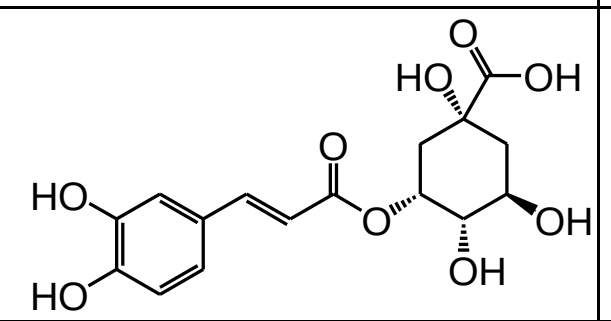	Sanshu	Morus alba	*Morus alba L.*[385, 386]
			3-caffeoylquinic acid	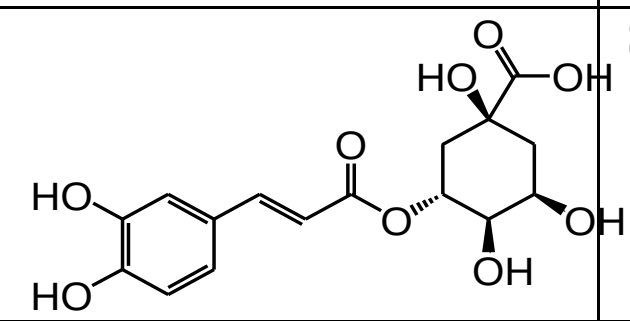	Sanyeyapateng	Tetrastigma hemsleyanum	*Tetrastigma hemsleyanum Diels et Gilg*[387-389]
			5-caffeoylquinic acid	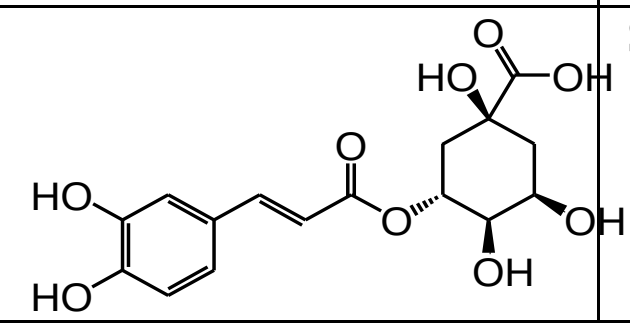	Sanyeyapateng	Tetrastigma hemsleyanum	*Tetrastigma hemsleyanum Diels et Gilg*[387, 388, 390]

			4-caffeoylquinic acid	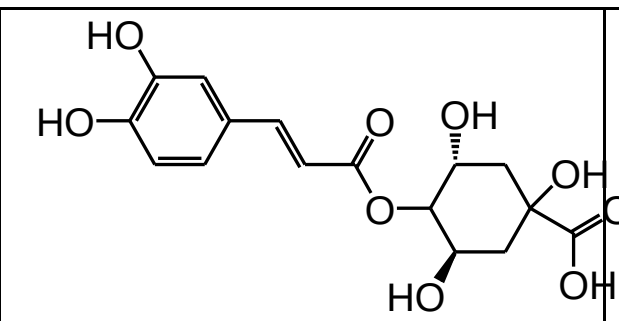	Jinyinhua	Lonicera japonica	*Lonicera japonica Thunb.*[389]
9	Caffeoylquinic acids		1-caffeoylquinic acid	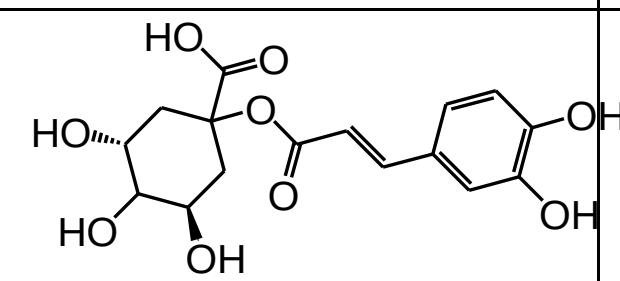	Sanyeyapateng	Tetrastigma hemsleyanum	*Tetrastigma hemsleyanum Diels et Gilg*[388]
			1,3-di-O-caffeoylquinic acid	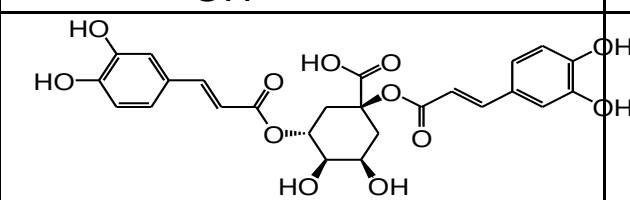	Jinyinhua	Lonicera japonica	*Lonicera japonica Thunb.*[391]
			3,5-di-O-caffeoylquinic acid	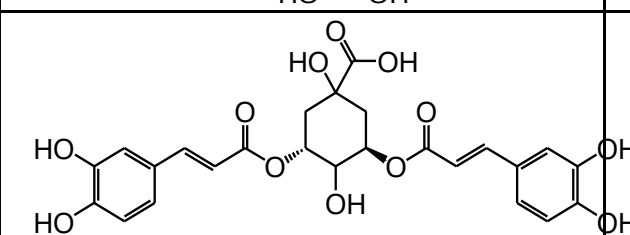	Jinyinhua	Lonicera japonica	*Lonicera japonica Thunb.*[391, 392]
			3,4-di-O-caffeoylquinic acid	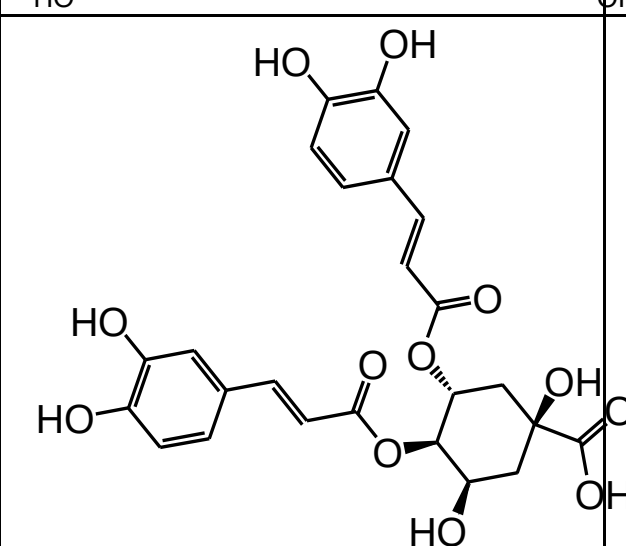	Jinyinhua	Lonicera japonica	*Lonicera japonica Thunb.*[391, 392]
			4,5-Di-O-caffeoylquinic acid	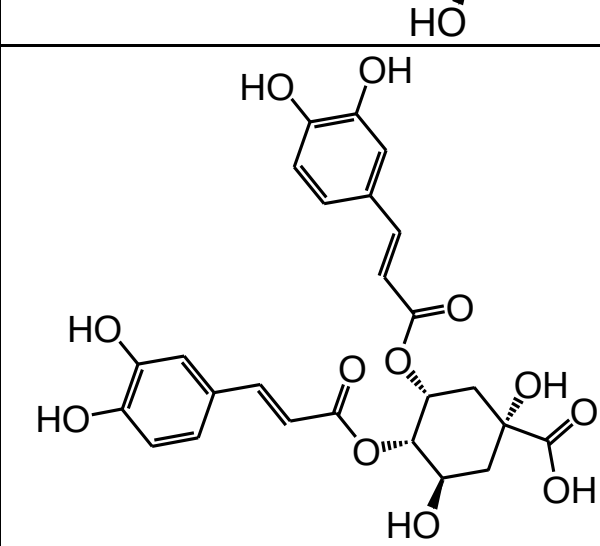	Guizhengcao	Bidens pilosa	*Bidens pilosa L.*[392]
			3,5-di-caffeoylquinic acid	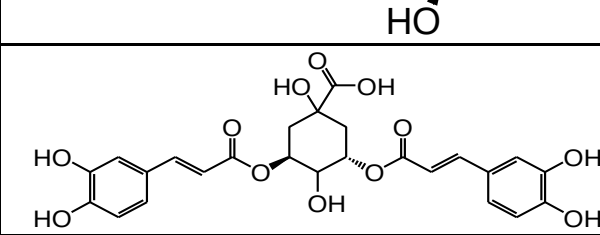	Kudincha	Leaf of Chinese Holly	*Ilex kudingcha C.J. Tseng*[393, 394]

			1,5-Dicaffeoylquinic acid	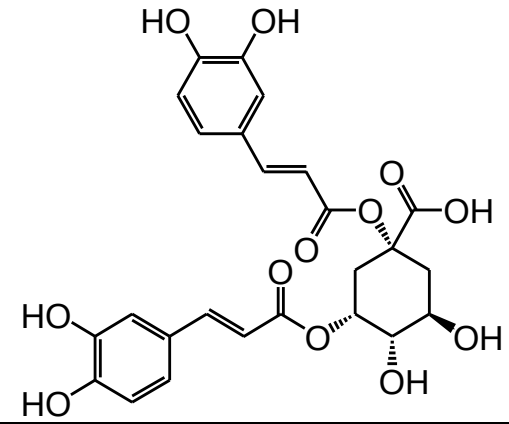	Huixiang	Foeniculum vulgare	*Foeniculum vulgare Mill.*[395-397]
			1-O-Caffeoylquinic acid	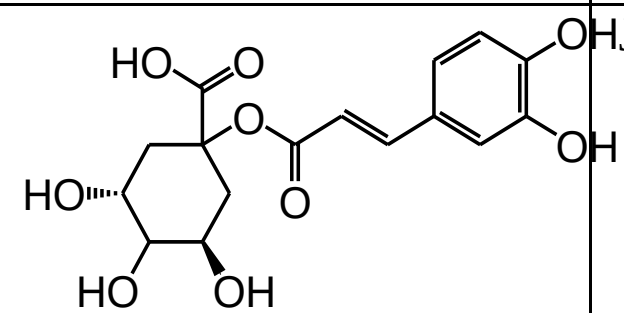	Jinyinhua	Lonicera japonica	*Lonicera japonica Thunb.*[391]
			1,4-Dicaffeoylquinic acid	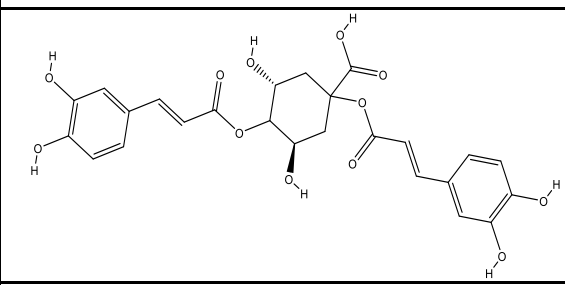	Baihao	white wormwood[398]	*Herba Artimisiae Sieversianae*
			Astaxanthin	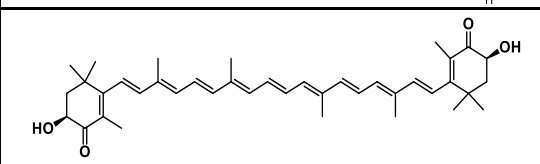	Yushenghuoqiuzao	*Haematococcus pluvialis*	*Haematococcus pluvialis*[399, 400]
			Dehydrohirsutanonol	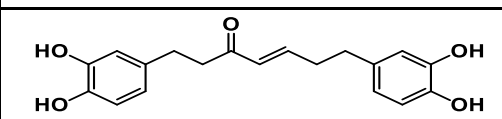	Chiyang	*Alnus japonica*	*Alnus japonica (Thunb.) Steud.*[401]
			Hexahydrocurcumin	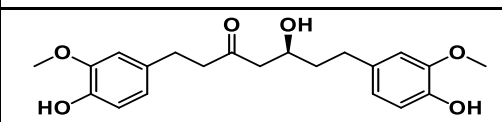	Ganjiang	*Zingiber officinale*	*Zingiber officinale Rosco*[402]
			Dehydrohirsutanonol	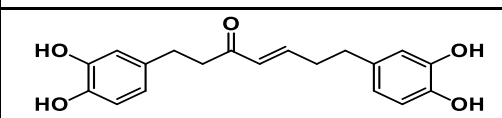	Chiyang	*Alnus japonica*	*Alnus japonica (Thunb.) Steud.*[401]
			Hexahydrocurcumin	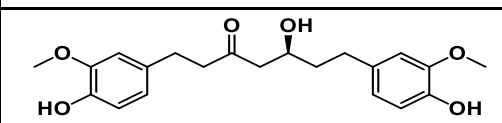	Ganjiang	*Zingiber officinale*	*Zingiber officinale Rosco*[402]
			Curcumin	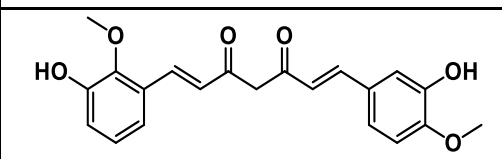	Jianghuang	*Curcuma longa L*	*Curcuma longa L*[403]
10	Curcumins and carotenoids	Curcumins	Tetrahydrocurcuminoids	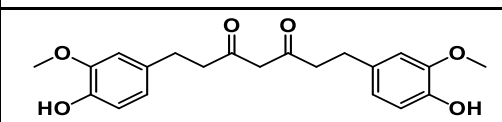	jianghuang	*Curcuma longa L*	*Curcuma longa L*[404]
			Dihydrocurcumin	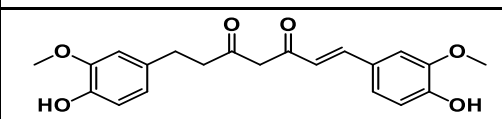	Jianghuang	*Curcuma longa L*	*Curcuma longa L* [405]
			Demethoxycurcumin	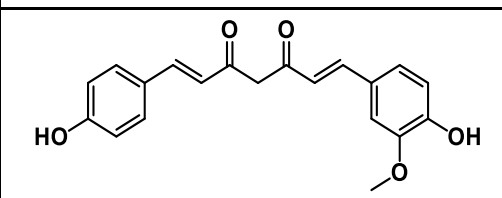	Ezhu	*Curcuma zedoaria (Christm.) Rosc*	*Curcuma zedoaria (Christm.) Rosc* [406]

			Bisdemethoxycurcumin	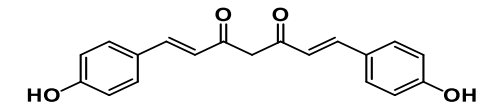	Ezhu	*Curcuma zedoaria (Christm.) Rosc*	*Curcuma zedoaria (Christm.) Rosc* [407]
			crocin	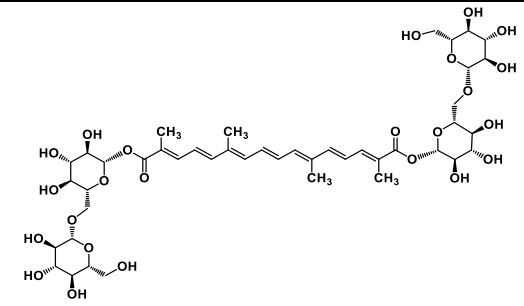	Zhizi	*Cardeniae Fructus*	*Gardenia jasminoides Ellis* [408]
			Crocetin	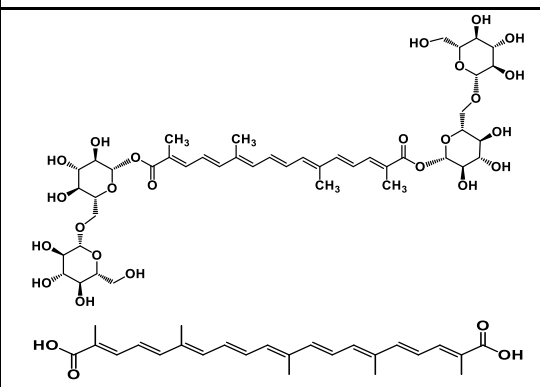	Xihonghua	*Croci stigma*	*Crocus sativus L*[408].
			Neurosporene	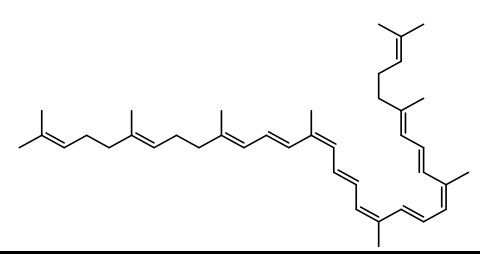	Fanqie	*tomato*	*Lycopersicon esculentum Mill.* [409]
			Carotene	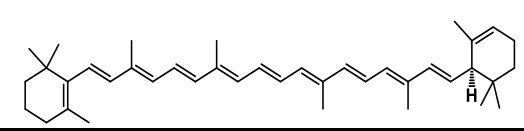	Nangua	*pumpkin*	*Cucurbita moschata (Duch. ex Lam.) Duch. ex Poiret*[410]
			Sinapine	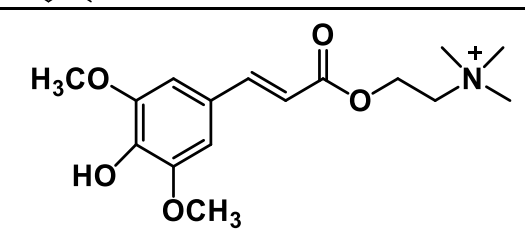	Laifuzi	*Semen Raphani*[411]	*Raphanus sativus L.*
			Higenamine	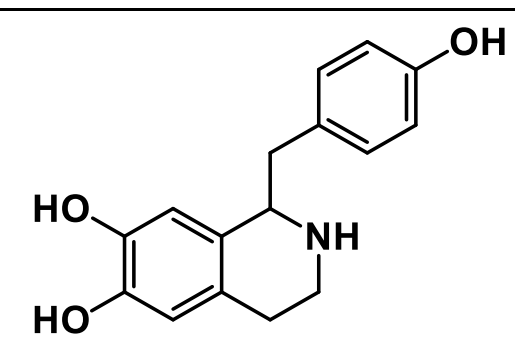	Wuyao	*Linderae Radix*	*Lindera aggregata (Sims) Kosterm.*[412]
		Carotenoids	Kukoamine A	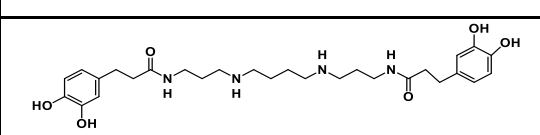	Digupi	*Lycii Cortex*	*Lycium chinense Mill.* [413]

			Kukoamine B	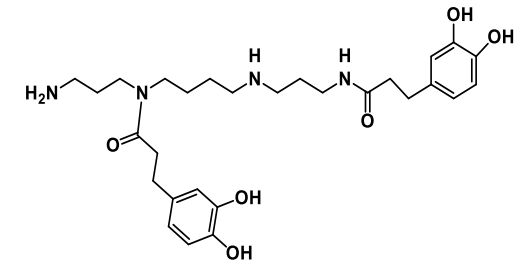	Digupi	*Lycii Cortex*	*Lycium chinense Mill.* [413]
			Magnocurarine	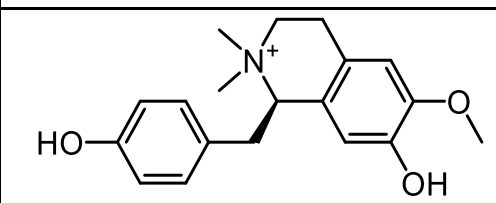	Chengqiezi	*Litsea cubeba*	*Litsea cubeba(Lour.)Pers*。 [414]
			Reticulin	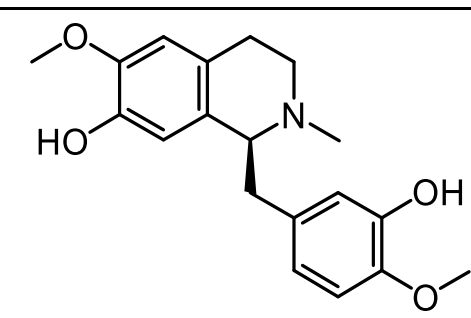	Fanlizhi	*Annona squamosa*	*Annona squamosa Linn.*[415]
11	Phenolic alkaloids		Aromoline	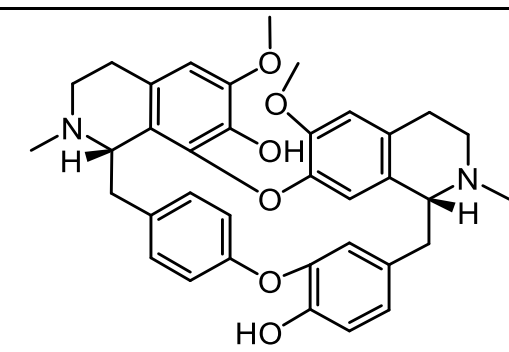	Tangsongcao	*Thalictrum aquilegifolium*	*Thalictrum aquilegifolium.*[416]
			Berbamine	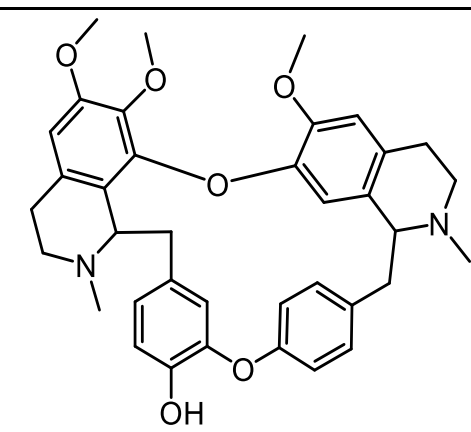	Baiyaozi	*Stephania cepharantha*	*Stephania cepharantha.*[417, 418]
			Neferine	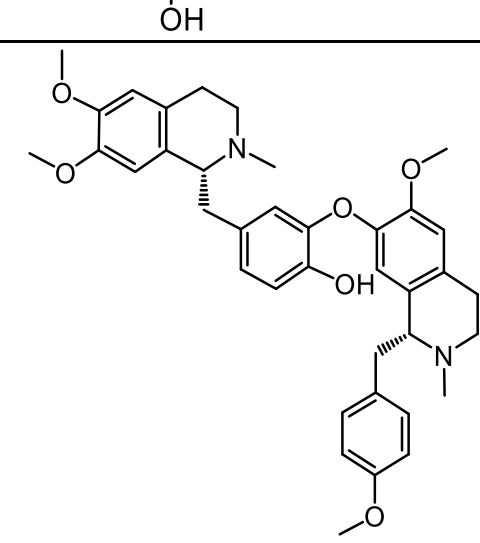	Lianzixin	*Nelumbo nucifera*	*Nelumbo nucifera Gaertn.*[419, 420]

			Fangchinoline	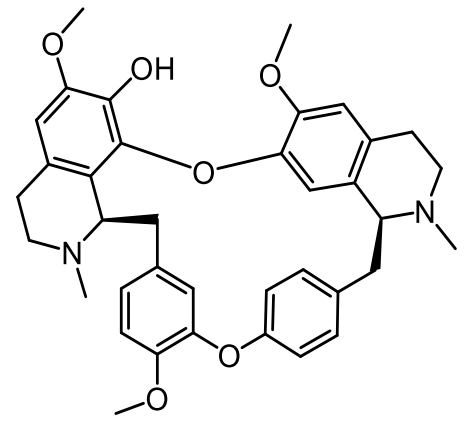	Fangji	*Stephania tetrandra*	*Stephania tetrandra S.Moore.*[421]
			Boldine	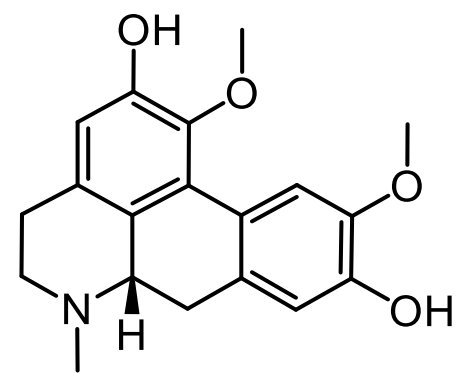	Boludushu	*Peumus boldus*	*Peumus boldus.*[422, 423]
			Laurotetanine	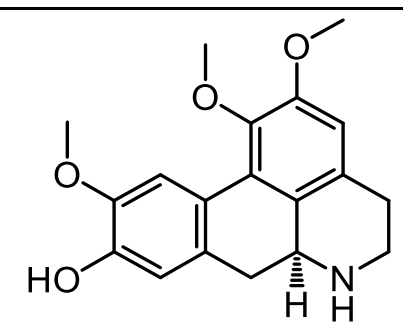	Biba	*Piper longum*	*Piper longum L.*[424]
			Magnoflorine	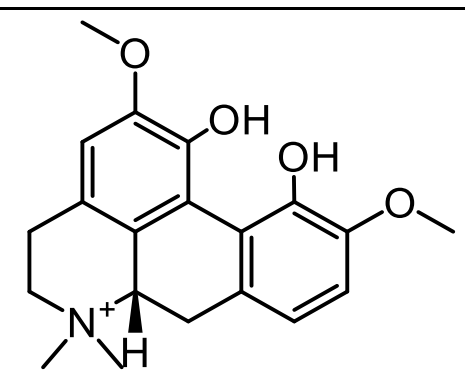	Beimadouling	*Aristolochia contorta*	*Aristolochia contorta Bunge.*[425]
			Jatrorrhizine	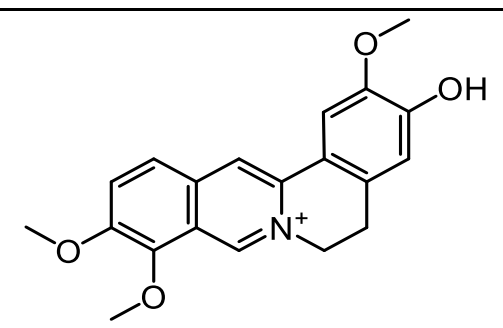	Shidagonglao	*Mahonia fortunei*	*Mahonia fortune (LindL)Fedde.*[426]
			Stepholidine	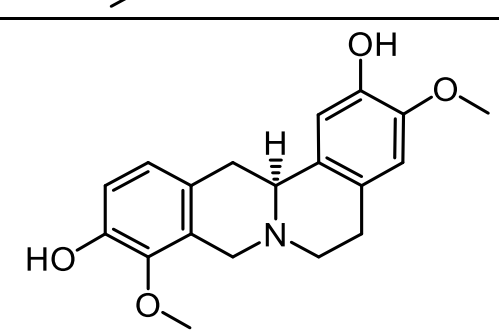	Bianfugegen	*Menispermum dauricum*	*Menispermum dauricum.*[427]

			Sinomenine	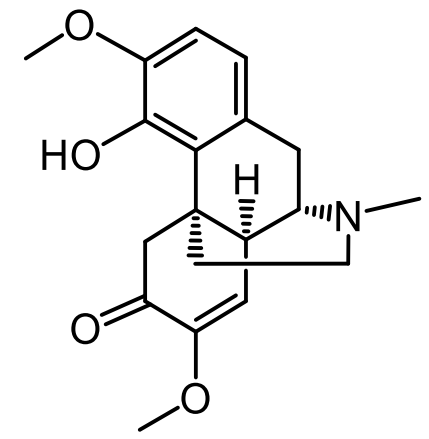	Bianfugegen	*Menispermum dauricum*	*Menispermum dauricum.*[428, 429]
			Haplopine	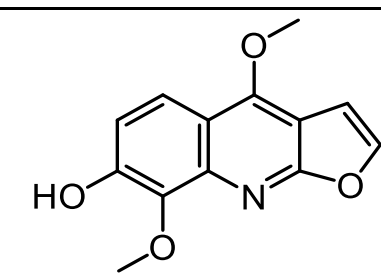	Huajiaole	*Zanthoxylum scandens*	*Zanthoxylum scandens Bl.*[430]
			Harmalol	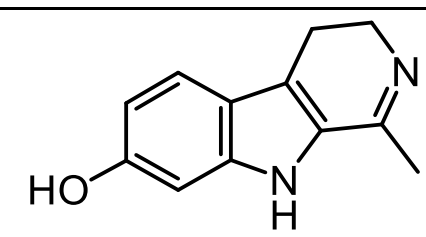	Luotuopeng	*Peganum harmala*	*Peganum harmala L.*[431]
			Hordenine	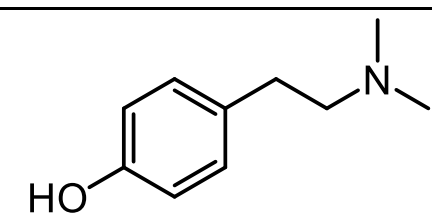	Duanyeshanmahu ang	*Desmodium tiliaefolium*	*Desmodium tiliaefolium.*[432]
			Sanggenon C	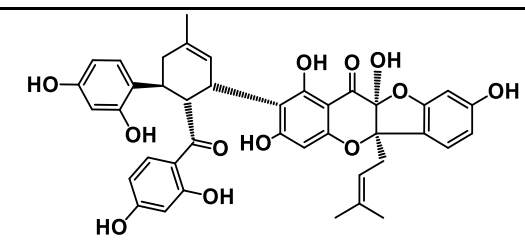	Sangbaipi	*Morus alba*	*Morus alba L.*[433, 434]
			cathayanons D	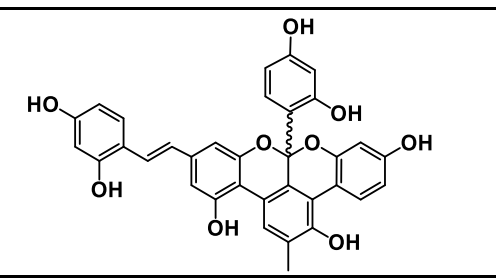	Huasang.	*cathayana*	*Morus. cathayana.* [435]
			Mulberrofuran K	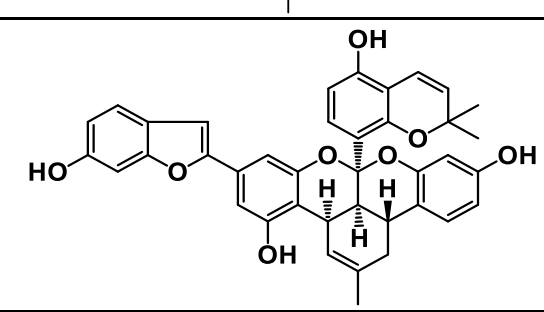	Changsuisang	*Morus wittiorum*	*Morus wittiorum*[436, 437]
12	Diels-Alder-type adduct		Mulberrofuran G	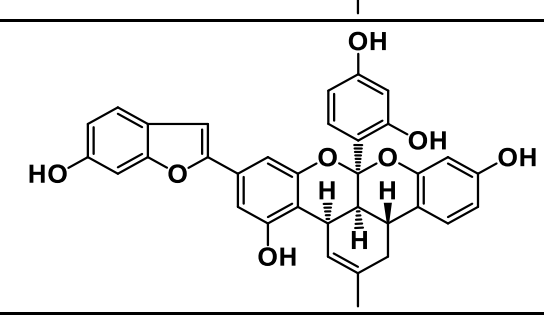	Changsuisang	*Morus wittiorum*	*Morus wittiorum.*[438, 439]

			Kuwanon J	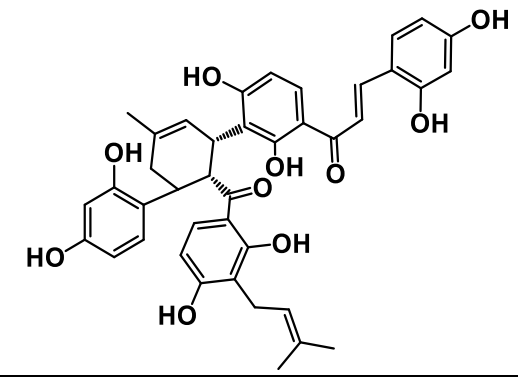	Changsuisang	*Morus wittiorum*	*Morus wittiorum*[440]
			Mongolicin F	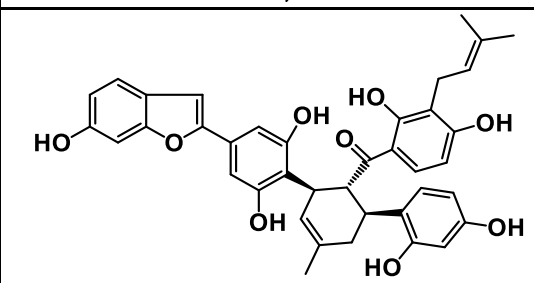	Changsuisang	*Morus wittiorum*	*Morus wittiorum.*[441]
			Salvianolic acid B	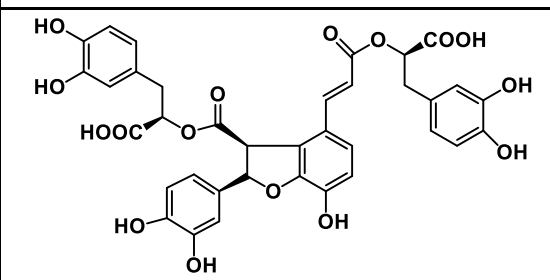	Dansen	*Salviae miltiorrhizae Radix et Rhizoma*	*Salvia Miltiorrhiza Bge*[442, 443]
			Bilobol	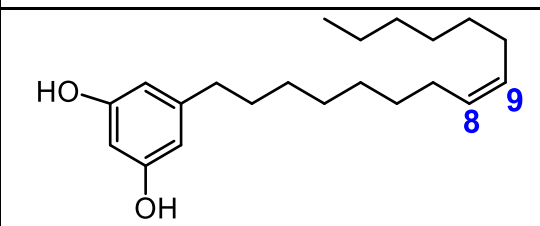	Baiguo	*Ginkgo biloba*	*Ginkgo biloba L*[444]
			Eckol	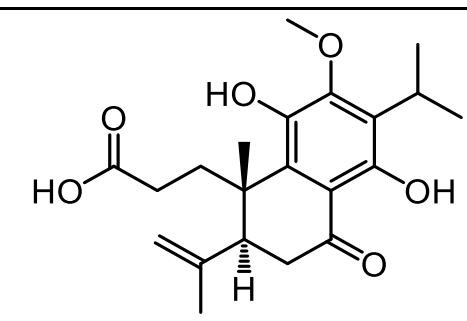	Hezao	*Ecklonia cava*	[445 446, 447]
			Embelin	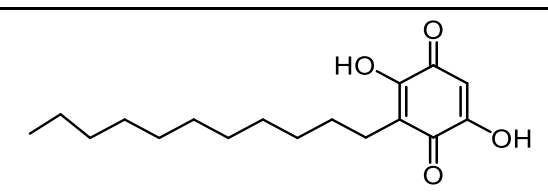	Weilingxian	*Clematis chinensis*	*Clematis chinensis Osbeck.*[448, 449]
		Phenyl-benzofuran/catechol hybrid	Embelin Phlorofucofuroeckol A	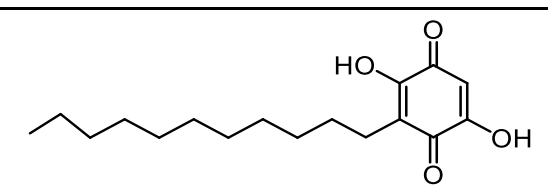			

				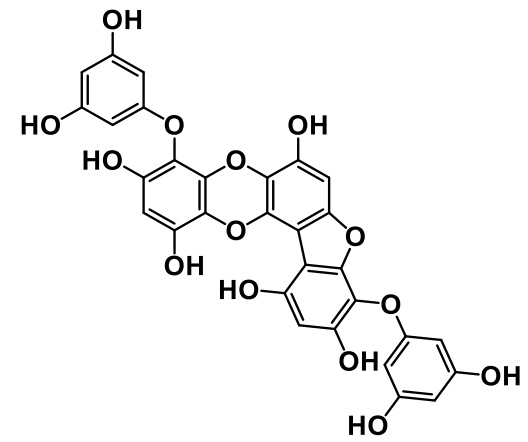			
			Hamamelitannin	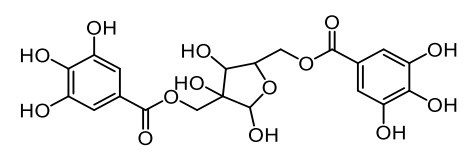	Kunbu	*brown alga; Ecklonia ; stolonifera*	*Ecklonia.*[450]
13	Phenolic hybrids		Oleuropein	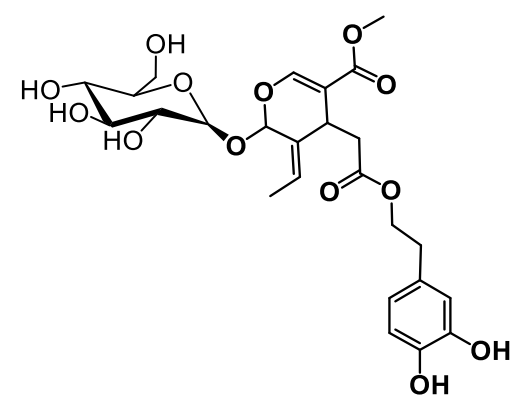	Jinlvmei	*Hamamelis virginiana*	*Hamamelis virginiana.*[451, 452]
			Hamamelitannin	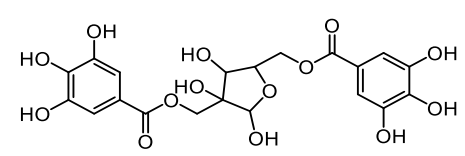	Ganlanye	*Olive leaf*	*Olea europaea L.*[453, 454]
			Carnosic acid<br>Rubiadin	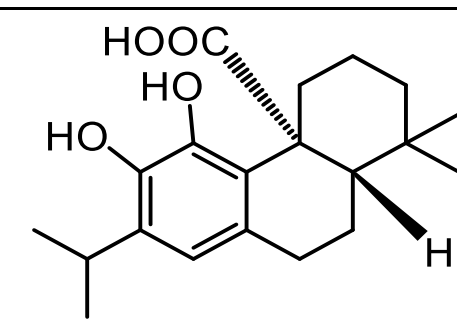 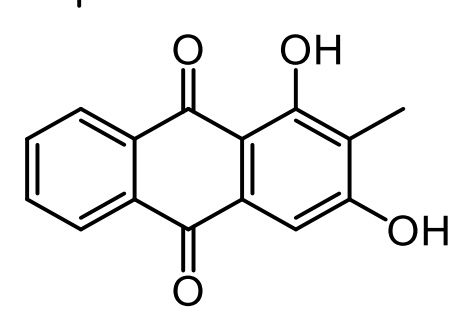	Midiexiang	*Rosmarinus officinalis*	*Rosmarinus officinalis L.*[455, 456]
					Bajitian	*Morinda citrifolia*	*Morinda citrifolia.*[457, 458]

			Carnosic acid<br>Rubiadin<br>Deoxyshikonin	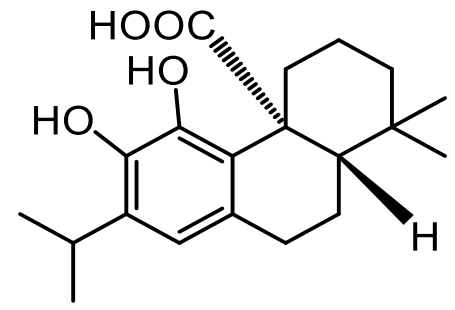<br>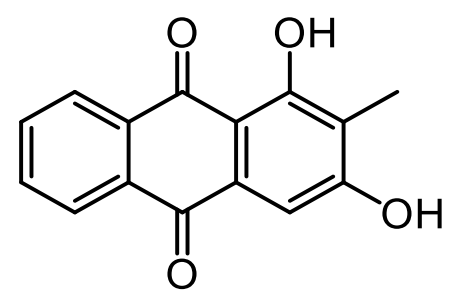<br>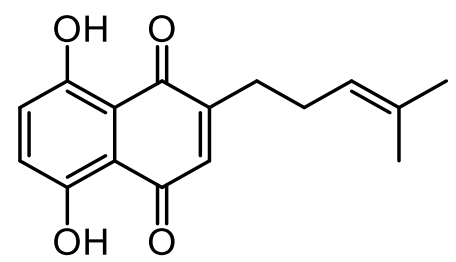	Zicao	*RadixArnebiae*	*RadixArnebiae.*[459]
			12-O-methylcandesalvone	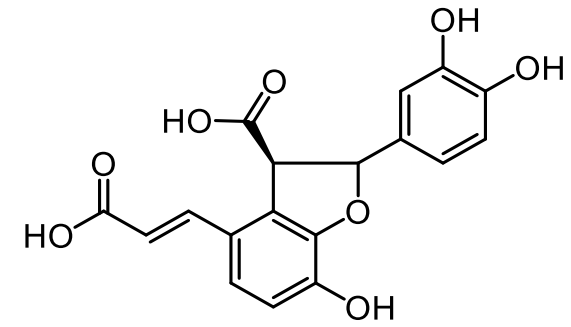	Zhutaishuweicao	*Salvia candelabrum*	*Salvia candelabrum.*[460, 461]
			Przewalskinic acid A	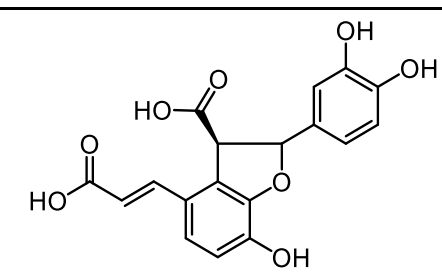	Danshen	*S. przewalskii*	*S. przewalskii.*[462]
			Tribuloside	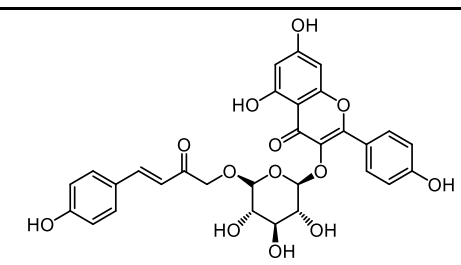	Jili	*Tribulus terrestris*	*Tribulus terrestris L.*[463]
			Punicalagin	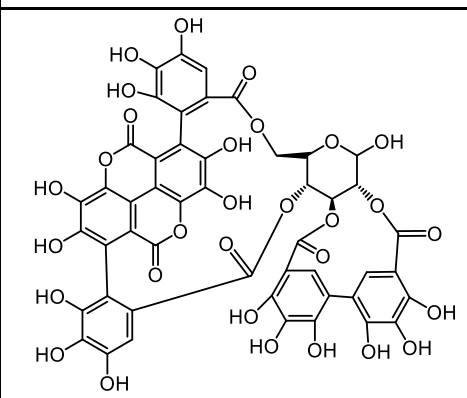	Shiliu	*Punica granatum*	*Punica granatum.*[464, 465]

			Aristolochic acid C	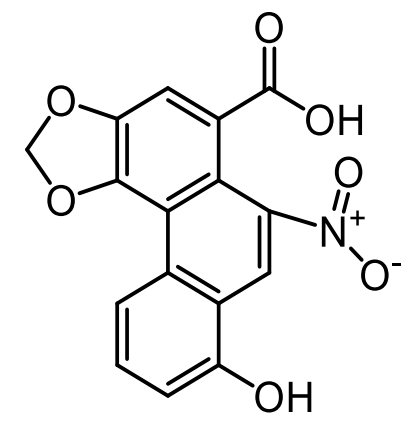	Guanmutong	*Aristolochia manshuriensis*	*Aristolochia manshuriensis.*[466]
			(+)-Gallocatechin	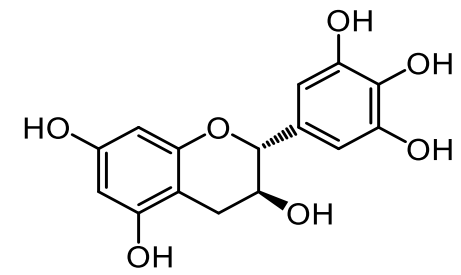	Chaye	*Acacia catechu*	*Acacia catechu (L.F.) Willd.*[467, 468]
			Amarogentin	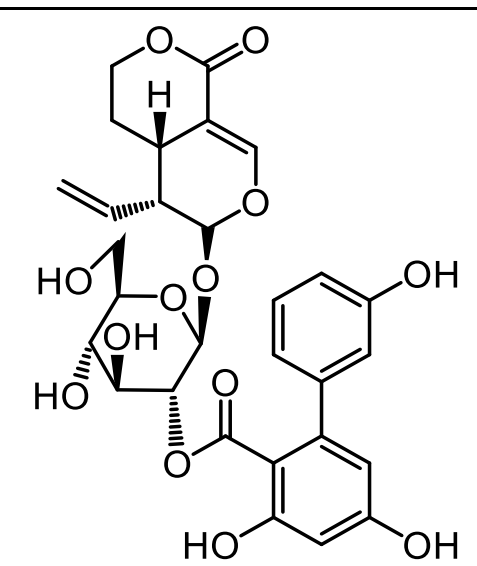	Huanglongdan	*Gentiana lutea*	*Gentiana lutea L.*[256, 469]
			Lucidumoside B	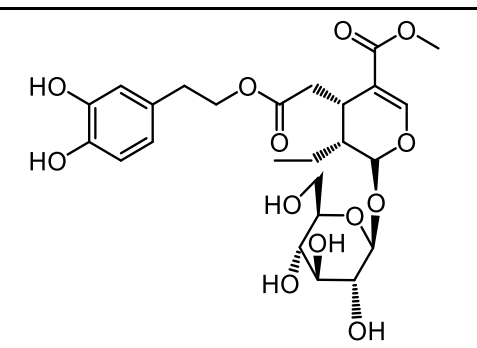	Nvzhenzi	*Ligustri Lucidi*	*Ligustri Lucidi.*[470]
			Lucidumoside C	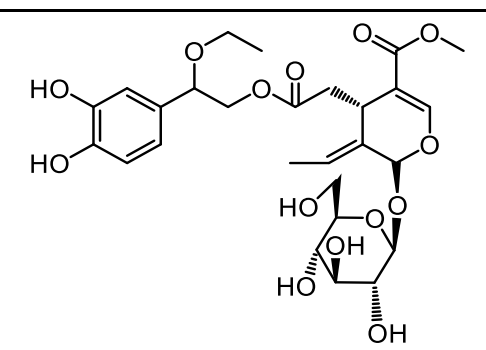	Nvzhenzi	*Ligustri Lucidi*	*Ligustri Lucidi.*[470]

			Neonuezhenide	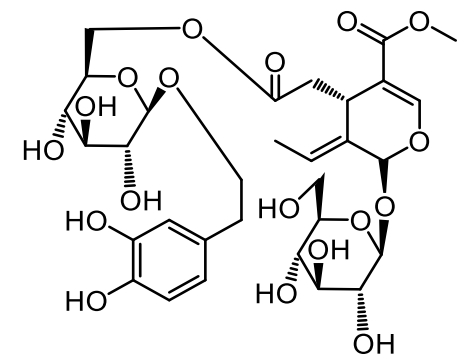	Nvzhenzi	*Ligustri Lucidi*	*Ligustri Lucidi.*[471]
			Pisiferic acid	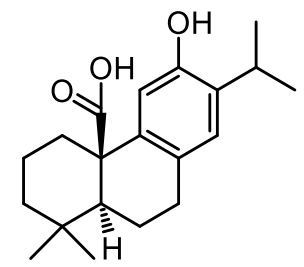	Ribenhuabo	*Chamaecyparis pisifera*	*Chamaecyparis pisifera (Sieb. et Zucc.) Endl.*[472, 473]
			Plectranthol A	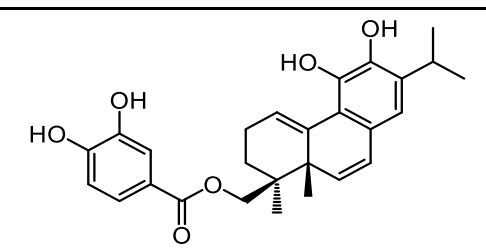	Yuanbanxiangcha cai	*Plectranthus nummularius*	*Plectranthus nummularius.*[474]
			Plectranthol B	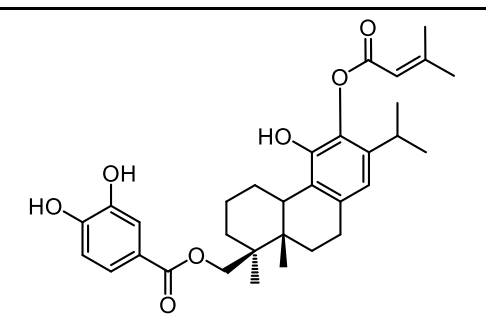	Yuanbanxiangcha cai	*Plectranthus nummularius*	*Plectranthus nummularius.*[474]
			α-Tocopherol	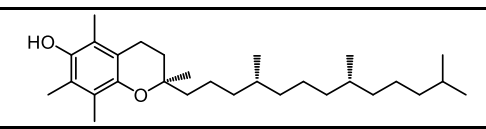	Bocai	*Spinacia oleracea*	*Spinacia oleracea L.*[475, 476]
			Castalagin	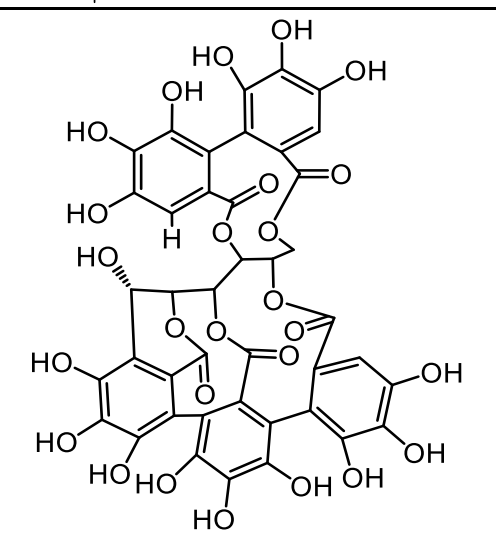	Shaji	*Hippophae rhamnoides*	*Hippophae rhamnoides Linn.*[477-480]

			Casuarictin	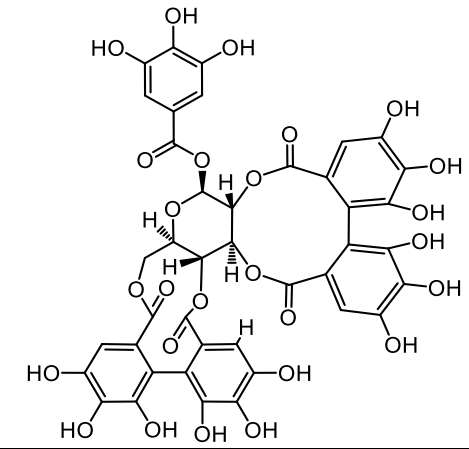	Baishao	*Paeonia albiflora*	*Paeonia albiflora[Syn. Paeonia lactiflora].*[481, 482]
			Chebulagic acid	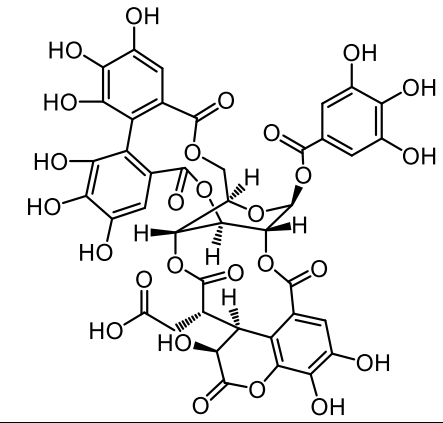	Anmole	*Phyllanthus emblica*	*Phyllanthus emblica Linn.*[483, 484]
			Chebulinic acid	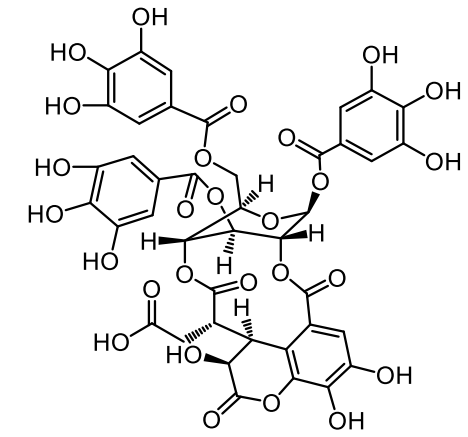	Anmole	*Phyllanthus emblica*	*Phyllanthus emblica Linn.*[485, 486]
			Corilagin	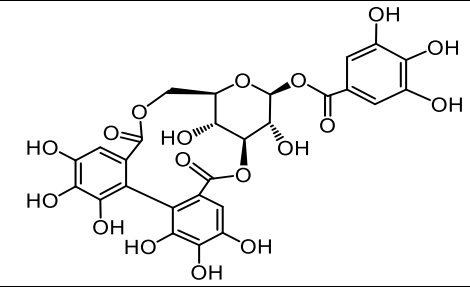	Anmole	*Phyllanthus emblica*	*Phyllanthus emblica Linn.*[487, 488]
			Geraniin	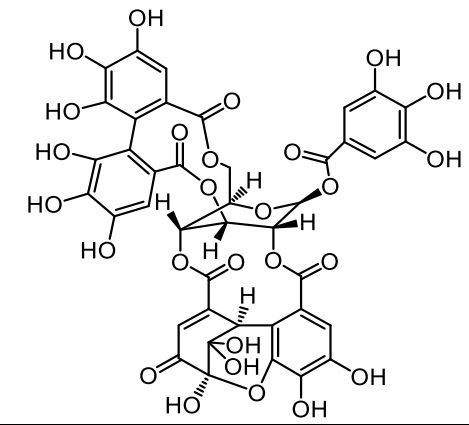	Anmole	*Phyllanthus emblica*	*Phyllanthus emblica Linn.*[489, 490]

			Mallotusinic acid	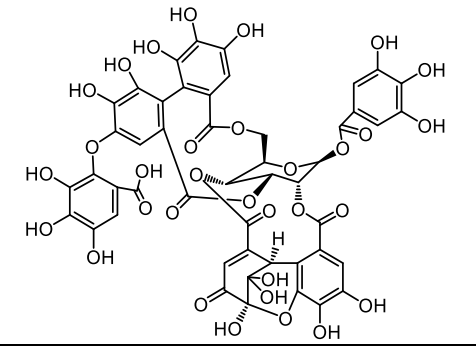	Hongbeishan magan	*Alchornea trewioides*	*Alchornea trewioides (Benth.) Muell. Arg.*[491]
			Strictinin	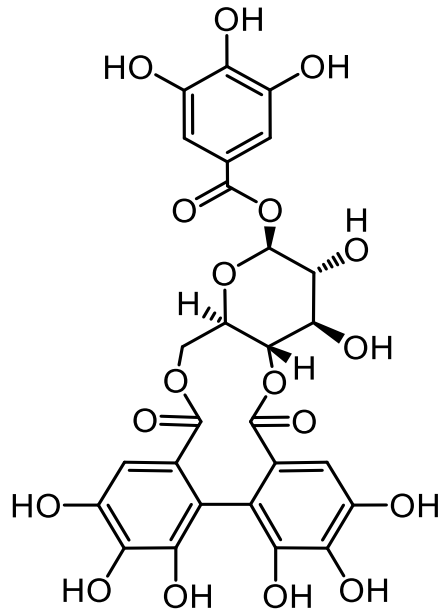	Baishao	*Paeonia albiflora*	*Paeonia albiflora[Syn. Paeonia lactiflora].*[492, 493]
			Pedunculagin	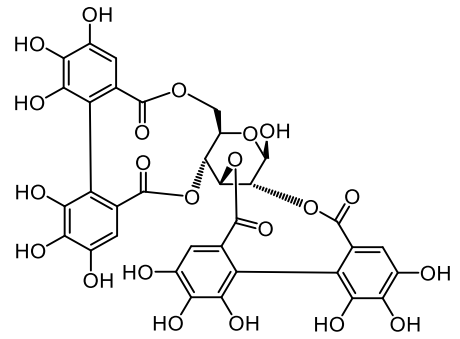	Baishao	*Paeonia albiflora*	*Paeonia albiflora[Syn. Paeonia lactiflora].*[494, 495]
			Tellimagrandin I	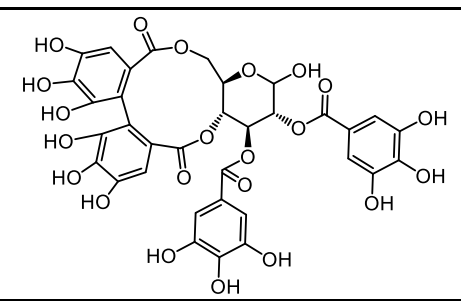	Baishao	*Paeonia albiflora*	*Paeonia albiflora[Syn. Paeonia lactiflora].*[496, 497]
			Dieckol	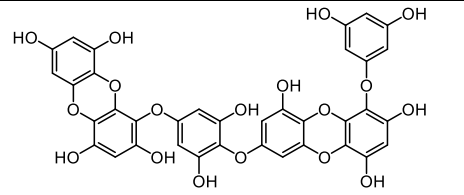	Zongzao	*Ecklonia stolonifera*	*Ecklonia stolonifera.*[498, 499]
			Eckstolonol	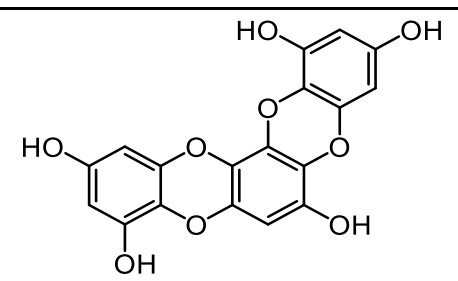	Zongzao	*Ecklonia stolonifera*	*Ecklonia stolonifera.*[500-502]

			Albibrissinoside B	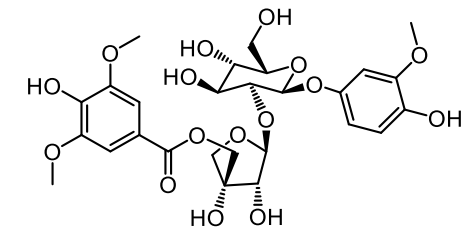	Hehuanpi	*Albizia julibrissin*	*Albizia julibrissin*[18]
			Curculigoside	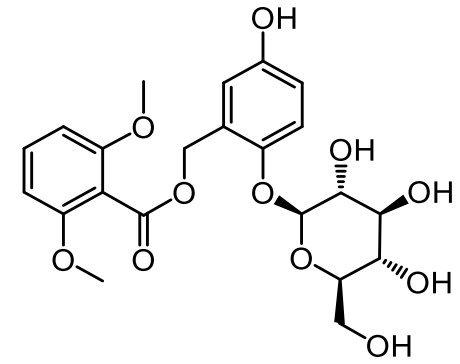	Xianmao	*Curculigo orchioides*	*Curculigo orchioides Gaertn.*[503, 504].
			Vaccihein A	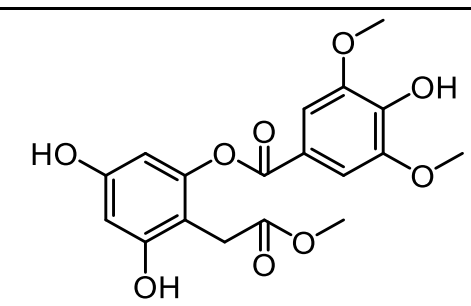	Yueju	*Vaccinium ashei*	*Vaccinium ashe.*[505]
			Dauricine	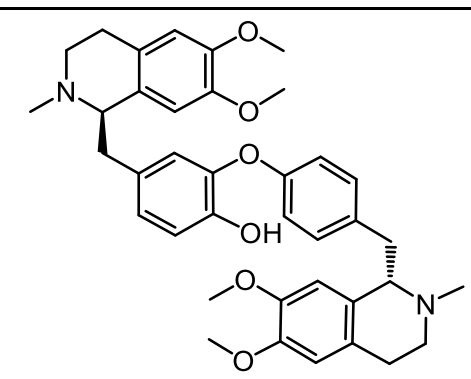	Bianfugegen	*Menispermum canadense*	*Menispermum canadense.*[506]
			Liensinine Piperlactam S	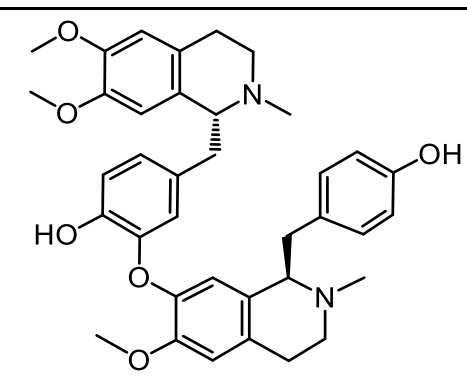	Lianzi	*Nelumbo nucifera*	*Nelumbo nucifera Gaertn.*[507]
			Piperlactam S	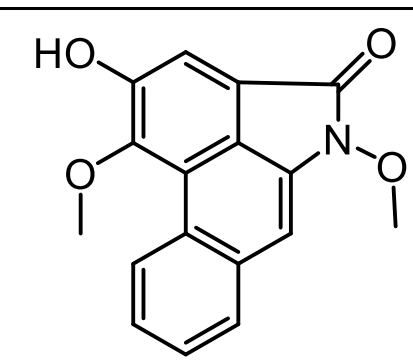	Haifengteng	*Piper kadsura*	*Piper kadsura [Syn.piper futokadsura].*[508]

			Paeonin C	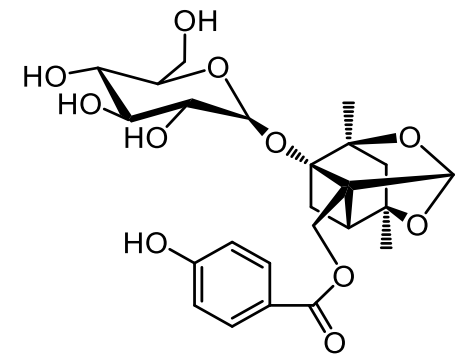	Douhuashaoyao	*Paeonia emodi*	*Paeonia emodi*[509]
			Protosappanin B	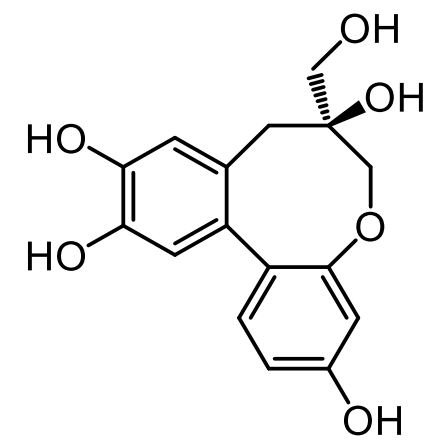	Sumu	*Caesalpinia sappan*	*Caesalpinia sappan Linn.*[510, 511]
			Usnic acid	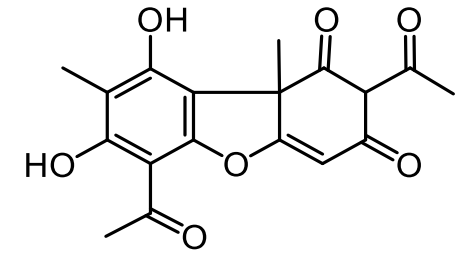	Songluo	*Usnea diffracta*	*Usnea diffracta Vai.*[512, 513]
			Gambogic acid	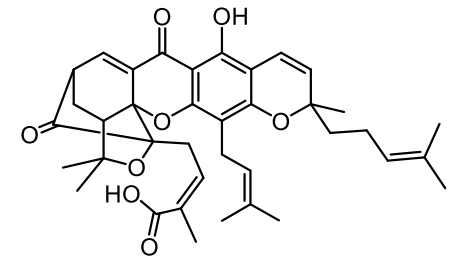	Tenghuang	*Garcinia hanburyi*	*Garcinia hanburyi Hook.f.*[514, 515]
			Procyanidin A2	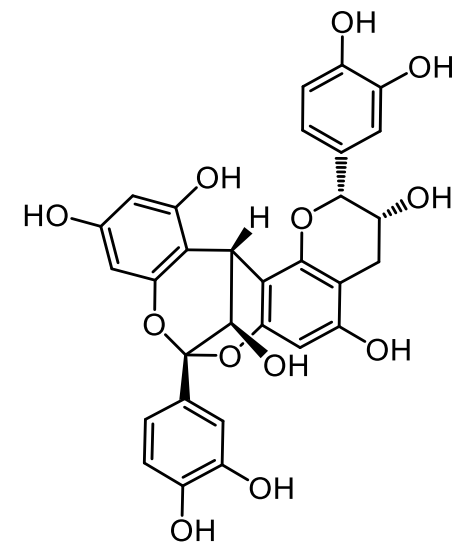	Putaozi	*Vitis vinifera*	*Vitis vinifera L.*[516, 517]
			Procyanidin B3	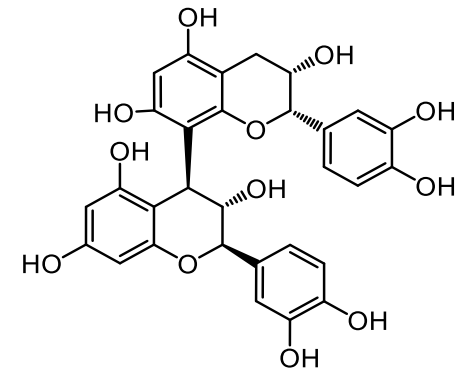	Putaozi	*Vitis vinifera*	*Vitis vinifera L.*[518, 519]

			Procyanidin C1	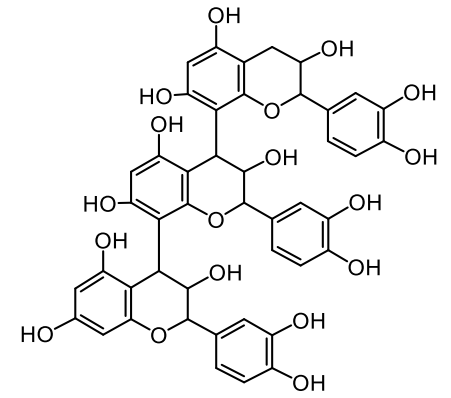	Putaozi	*Vitis vinifera*	*Vitis vinifera L.*[520, 521]
			Procyanidin B2	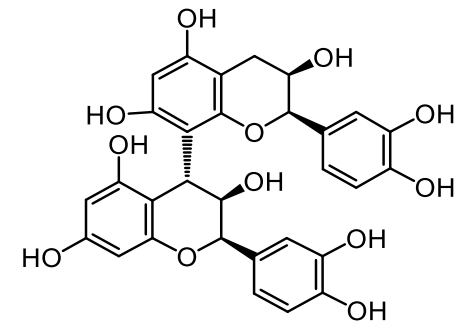	Putaozi<br>Mihoutao	*Vitis vinifera*<br>*Kiwifruit*	*Vitis vinifera L.*[522]<br>*Kiwifruit Cultivars*[523]
			Theaflavin	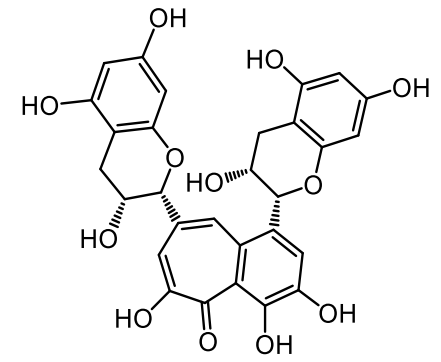	Hongcha	*Black tea*	*Black tea.*[524, 525]
			Theaflavin digallate	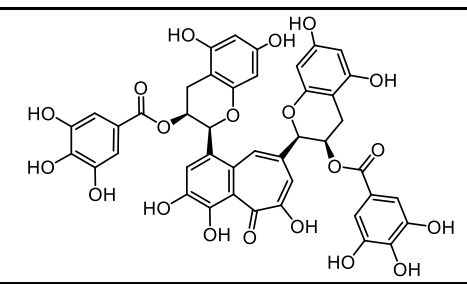	Hongcha	*Black tea*	*Black tea.*[526, 527]
			Geraniin	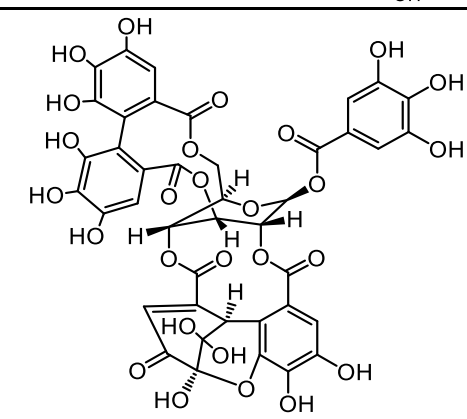	Yexiazhu	*Phyllanthus urinaria*	*Phyllanthus urinaria L.*[489, 490]
			Cyanidin 3-Arabinoside	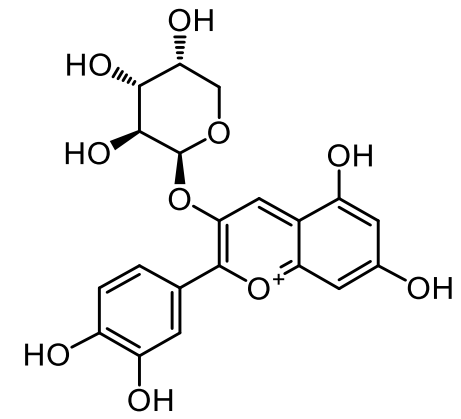	Heidou	*Glycinemax*	*Glycinemax（L.）merr.*[528]

			10-Gingerol	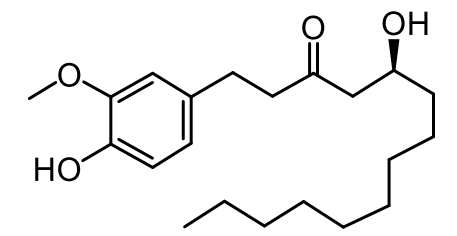	Jiang	*Zingiber officinale*	*Zingiber officinale Rosc.*[529]
			Gossypol	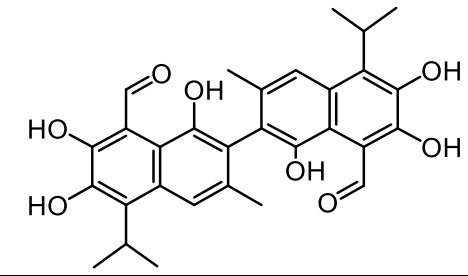	Mianhua	*Gossypium spp*	*Gossypium spp.*[530, 531]
			Echinacoside	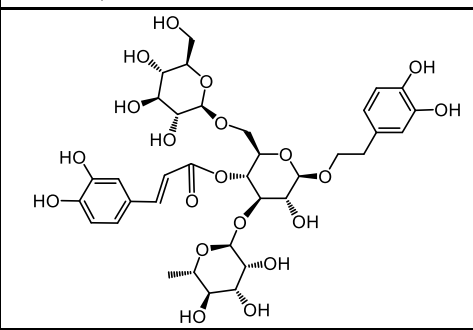	Roucongrong	*Cistanche deserticola*	*Cistanche deserticola Ma.*[532, 533]
			Leonurine	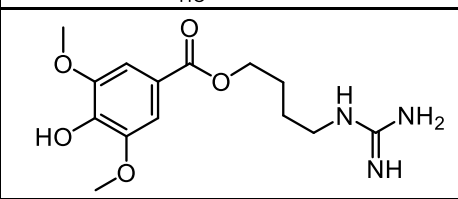	Yimucao	*Leonurus sibiricus*	*Leonurus artemisia (Laur.) S. Y. Hu F.*[534, 535]
			Phyllostadimer A	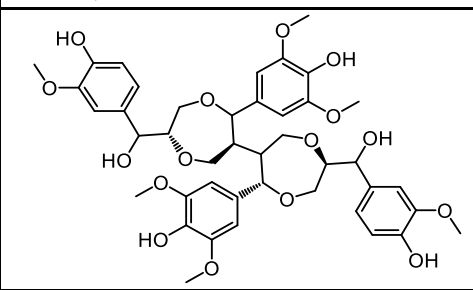	Mengzongzhu	*Phyllostachys edulis*	*Phyllostachys edulis.*[536]
			Maclurin	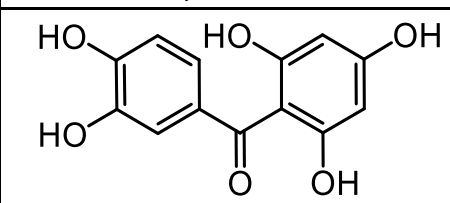	Daonianzi	*Garcinia mangostana*	*Garcinia mangostana.*[537, 538]
			Harunganol B	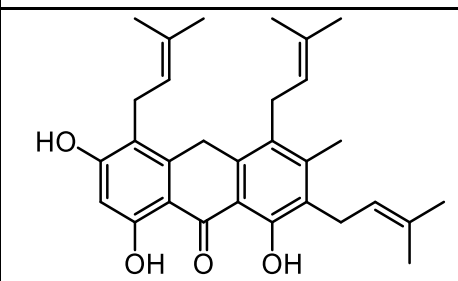	Madaohanimu	*Harungana madagascariensis*	*Harungana madagascariensis.*[539]
			Harungin anthrone	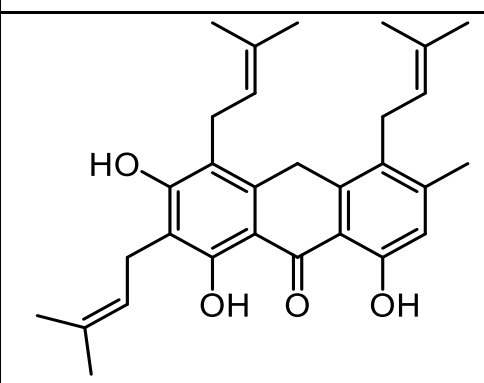	Madaohanimu	*Harungana madagascariensis*	*Harungana madagascariensis.*[539]

			Hypericin	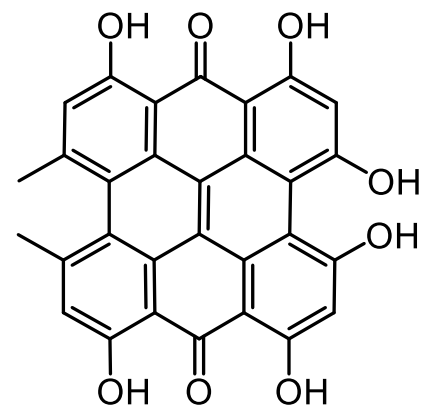	Biandijin	*Hypericum wightianum*	*Hypericum wightianum Wall. ex Wight et Arn.*[540, 541]

References

(1) Essa, H. A.; El-Metwally, A. E. 6-Gingerol ameliorates high-fat, high-sucrose diet-induced metabolic dysfunction and depressive-like behaviors by attenuating neuroinflammation and oxidative stress. *Metabolic Brain Disease* **2026**, *41* (1). DOI: 10.1007/s11011-025-01782-9.

(2) Zhang, F.; Zhang, J. G.; Yang, W.; Xu, P.; Xiao, Y. L.; Zhang, H. T. 6-Gingerol attenuates LPS-induced neuroinflammation and cognitive impairment partially via suppressing astrocyte overactivation. *Biomed. Pharmacother.* **2018**, *107*, 1523-1529. DOI: 10.1016/j.biopha.2018.08.136.

(3) Zuo, A. R.; Dong, H. H.; Yu, Y. Y.; Shu, Q. L.; Zheng, L. X.; Yu, X. Y.; Cao, S. W. The antityrosinase and antioxidant activities of flavonoids dominated by the number and location of phenolic hydroxyl groups. *Chin. Med.* **2018**, *13*, 12. DOI: 10.1186/s13020-018-0206-9.

(4) Marteau, C.; Guitard, R.; Penverne, C.; Favier, D.; Nardello-Rataj, V.; Aubry, J. M. Boosting effect of ortho-propenyl substituent on the antioxidant activity of natural phenols. *Food Chem.* **2016**, *196*, 418-427. DOI: 10.1016/j.foodchem.2015.09.007.

(5) Mateen, S.; Rehman, M. T.; Shahzad, S.; Naeem, S. S.; Faizy, A. F.; Khan, A. Q.; Khan, M. S.; Husain, F. M.; Moin, S. Anti-oxidant and anti-inflammatory effects of cinnamaldehyde and eugenol on mononuclear cells of rheumatoid arthritis patients. *European journal of pharmacology* **2019**, *852*, 14-24. DOI: 10.1016/j.ejphar.2019.02.031.

(6) Sharma, U. K.; Kumar, R.; Gupta, A.; Ganguly, R.; Singh, A. K.; Ojha, A. K.; Pandey, A. K. Ameliorating efficacy of eugenol against metanil yellow induced toxicity in albino Wistar rats. *Food and chemical toxicology : an international journal published for the British Industrial Biological Research Association* **2019**, *126*, 34-40. DOI: 10.1016/j.fct.2019.01.032.

(7) Cheng, J.; Dong, S. Q.; Yi, L. T.; Geng, D.; Liu, Q. Magnolol abrogates chronic mild stress-induced depressive-like behaviors by inhibiting neuroinflammation and oxidative stress in the prefrontal cortex of mice. *International Immunopharmacology* **2018**, *59*, 61-67. DOI: 10.1016/j.intimp.2018.03.031.

(8) Gursul, S.; Karabulut, I.; Durmaz, G. Antioxidant efficacy of thymol and carvacrol in microencapsulated walnut oil triacylglycerols. *Food Chem.* **2019**, *278*, 805-810. DOI: 10.1016/j.foodchem.2018.11.134.

(9) Dairi, N.; Ferfera-Harrar, H.; Ramos, M.; Garrigos, M. C. Cellulose acetate/AgNPs-organoclay and/or thymol nano-biocomposite films with combined antimicrobial/antioxidant properties for active food packaging use. *International journal of biological macromolecules* **2018**, *121*, 508-523. DOI: 10.1016/j.ijbiomac.2018.10.042.

(10) Hernandez, D. A.; Tenorio, F. J. Reactivity indexes of antioxidant molecules from Rosmarinus officinalis. *Structural Chemistry* **2018**, *29* (3), 741-751. DOI: 10.1007/s11224-017-1066-0.

(11) Loussouarn, M.; Krieger-Liszkay, A.; Svilar, L.; Bily, A.; Birtic, S.; Havaux, M. Carnosic Acid and Carnosol, Two Major Antioxidants of Rosemary, Act through Different Mechanisms. *Plant Physiology* **2017**, *175* (3), 1381-1394. DOI: 10.1104/pp.17.01183.

(12) Ghasemi, S.; Koohi, D. E.; Emmamzadehhashemi, M. S. B.; Khamas, S. S.; Moazen, M.; Hashemi, A. K.; Amin, G.; Golfakhrabadi, F.; Yousefi, Z.; Yousefbeyk, F. Investigation of phenolic compounds and antioxidant activity of leaves extracts from seventeen cultivars of Iranian olive (Olea europaea L.). *Journal of Food Science and Technology-Mysore* **2018**, *55* (11), 4600-4607. DOI: 10.1007/s13197-018-3398-1.

(13) Yildiz, Z. I.; Celebioglu, A.; Kilic, M. E.; Durgun, E.; Uyar, T. Fast-dissolving carvacrol/cyclodextrin inclusion complex electrospun fibers with enhanced thermal stability, water solubility, and antioxidant activity. *Journal of Materials Science* **2018**, *53* (23), 15837-15849. DOI: 10.1007/s10853-018-2750-1.

(14) Adeyemi, O. S.; Atolani, O.; Banerjee, P.; Arolasafe, G.; Preissner, R.; Etukudoh, P.; Ibraheem, O. Computational and experimental validation of antioxidant properties of synthesized bioactive ferulic acid derivatives. *International Journal of Food Properties* **2018**, *21* (1), 101-113. DOI: 10.1080/10942912.2018.1439958.

(15) Shirazi, M. T.; Gholami, H.; Kavoosi, G.; Rowshan, V.; Tafsiry, A. Chemical composition, antioxidant, antimicrobial and cytotoxic activities of Tagetes minuta and Ocimum basilicum essential oils. *Food science & nutrition* **2014**, *2* (2), 146-155. DOI: 10.1002/fsn3.85.

(16) Teissedre, P. L.; Waterhouse, A. L. Inhibition of oxidation of human low-density lipoproteins by phenolic substances in different essential oils varieties. *Journal of Agricultural and Food Chemistry* **2000**, *48* (9), 3801-3805. DOI: 10.1021/jf990921x.
(17) Zhang, R. F.; Khan, S. A.; Lin, Y. S.; Guo, D. L.; Pan, X. W.; Liu, L.; Wei, Z. C.; Zhang, Y.; Deng, Y. Y.; Zhang, M. W. Phenolic profiles and cellular antioxidant activity of longan pulp of 24 representative Chinese cultivars. *International Journal of Food Properties* **2018**, *21* (1), 746-759. DOI: 10.1080/10942912.2018.1425705.
(18) Jung, M. J.; Kang, S. S.; Jung, Y. J.; Choi, J. S. Phenolic glycosides from the stem bark of Albizzia julibrissin. *Chemical & pharmaceutical bulletin* **2004**, *52* (12), 1501-1503. DOI: 10.1248/cpb.52.1501.
(19) Wu, Q.; Fu, D. X.; Hou, A. J.; Lei, G. Q.; Llu, Z. J.; Chen, J. K.; Zhou, T. S. Antioxidative phenols and phenolic glycosides from Curculigo orchioides. *Chemical & pharmaceutical bulletin* **2005**, *53* (8), 1065-1067. DOI: 10.1248/cpb.53.1065.
(20) Chen, L. H.; Chen, C.; Zheng, Z. Z.; Liu, S. S.; Tong, Q. X.; Xiao, J. B.; Lin, H. T.; Ming, Y. L. Cytotoxic and antioxidant activities of Macfadyena unguis-cati L. aerial parts and bioguided isolation of the antitumor active components. *Industrial Crops and Products* **2017**, *107*, 531-538, Article; Proceedings Paper. DOI: 10.1016/j.indcrop.2017.05.018.
(21) Aminah, N. S.; Yulvia, A.; Tanjung, M. Methyl-3,4-Dihydroxybenzoate and 9-10-Dihydrophenanthrene-2,4,7-Triol Two Phenolic Compounds from Dioscorea alata L. and Their Antioxidant Activity. In *6th International Conference and Workshops on Basic and Applies Sciences*, Abdullah, H. Y. Ed.; AIP Conference Proceedings, Vol. 1888; Amer Inst Physics, 2017.
(22) Demirci, M. A.; Ipek, Y.; Gul, F.; Ozen, T.; Demirtas, I. Extraction, isolation of heat-resistance phenolic compounds, antioxidant properties, characterization and purification of 5-hydroxymaltol from Turkish apple pulps. *Food Chemistry* **2018**, *269*, 111-117. DOI: 10.1016/j.foodchem.2018.06.147.
(23) He, Z. D.; Lau, K. M.; But, P. P. H.; Jiang, R. W.; Dong, H.; Ma, S. C.; Fung, K. P.; Ye, W. C.; Sun, H. D. Antioxidative glycosides from the leaves of Ligustrum robustum. *Journal of Natural Products* **2003**, *66* (6), 851-854. DOI: 10.1021/np020568g.
(24) Qiong, W., et al. Antioxidative Phenols and Phenolic Glycosides from Curculigo orchioides. *Chem. Pharm. Bull* **2005**.
(25) Liu, C.-M.; Yang, H.-X.; Ma, J.-Q.; Yang, W.; Feng, Z.-J.; Sun, J.-M.; Cheng, C.; Li, J.; Jiang, H. Role of AMPK pathway in lead-induced endoplasmic reticulum stress in kidney and in paeonol-induced protection in mice. *Food and chemical toxicology : an international journal published for the British Industrial Biological Research Association* **2018**, *122*, 87-94. DOI: 10.1016/j.fct.2018.10.024.
(26) Wang, H.; Sun, Y.; Ye, W. C.; Xiong, F.; Wu, H. J.; Yang, C. H.; Zhao, S. X. Antioxidative phenylethanoid and phenolic glycosides from Picrorhiza scrophulariiflora. *Chem. Pharm. Bull.* **2004**, *52* (5), 615-617. DOI: 10.1248/cpb.52.615.
(27) Zhang, H. Y.; Ge, N.; Zhang, Z. Y. Theoretical elucidation of activity differences of five phenolic antioxidants. *Acta Pharmacologica Sinica* **1999**, *20* (4), 363-366, Article.
(28) Schwartner, C.; Bors, W.; Michel, C.; Franck, U.; MullerJakic, B.; Nenninger, A.; Asakawa, Y.; Wagner, H. Effect of Marchantins and related compounds on 5-lipoxygenase and cyclooxygenase and their antioxidant properties: A structure activity relationship study. *Phytomedicine* **1995**, *2* (2), 113-117. DOI: 10.1016/s0944-7113(11)80055-3.
(29) Xie, S. Y.; Yang, X. Y.; Ding, Z. G.; Li, M. G.; Zhao, J. Y. Chemical Constituents and Pharmacological Effects of Taraxacum mongolicum Hand.-Mazz. *Natural Product Research and Development* **2012**, *24* (S1), 141-151.
(30) Zhou, J.; Ma, Y.; Jia, Y.; Pang, M.; Cheng, G.; Cai, S. Phenolic profiles, antioxidant activities and cytoprotective effects of different phenolic fractions from oil palm (Elaeis guineensis Jacq.) fruits treated by ultra-high pressure. *Food Chem.* **2019**, *288*, 68-77. DOI: 10.1016/j.foodchem.2019.03.002.
(31) Li, X. C.; Lin, J.; Gao, Y. X.; Han, W. J.; Chen, D. F. Antioxidant activity and mechanism of Rhizoma Cimicifugae. *Chem. Cent. J.* **2012**, *6* (1). DOI: 10.1186/1752-153X-6-140.
(32) Wang, X. Z.; Li, X. C.; Chen, D. F. Evaluation of antioxidant activity of isoferulic acid in vitro. *Nat. Prod. Commun.* **2011**, *6* (9), 1285-1288.
(33) Alsanie, W. F.; El-Hallous, E. I.; Dessoky, E. S.; Ismail, I. A. Viper's Bugloss (Echium vulgare L) Extract as A Natural Antioxidant and Its Effect on Hyperlipidemia. *Int. J. Pharm. Phytopharm. Res.* **2018**, *8* (1), 81-89, Article.
(34) Li, X. C.; Hu, Q. P.; Jiang, S. X.; Li, F.; Lin, J.; Han, L.; Hong, Y. L.; Lu, W. B.; Gao, Y. X.; Chen, D. F. Flos Chrysanthemi Indici protects against hydroxyl-induced damages to DNA and MSCs via antioxidant mechanism. *J. Saudi Chem. Soc.* **2015**, *19* (4), 454-460. DOI: 10.1016/j.jscs.2014.06.004.
(35) Wang, Z. Y.; Xia, Q.; Liu, X.; Liu, W. X.; Huang, W. Z.; Mei, X.; Luo, J.; Shon, M. X.; Lin, R. C.; Zou, D. X.; et al. Phytochemistry, pharmacology, quality control and future research of Forsythia suspensa (Thunb.) Vahl: A review. *Journal of Ethnopharmacology* **2018**, *210*, 318-339. DOI: 10.1016/j.jep.2017.08.040.
(36) Jiang, Q.; Li, X. C.; Tian, Y. G.; Lin, Q. Q.; Xie, H.; Lu, W. B. A.; Chi, Y. G.; Chen, D. F. Lyophilized aqueous extracts of Mori Fructus and Mori Ramulus protect Mesenchymal stem cells from center dot OH-treated damage: bioassay and antioxidant mechanism. *Bmc Complementary and Alternative Medicine* **2017**, *17*. DOI: 10.1186/s12906-017-1730-3.

(37) Shin, H. Y.; Kim, S. M.; Lee, J. H.; Lim, S. T. Solid-state fermentation of black rice bran with Aspergillus awamori and Aspergillus oryzae: Effects on phenolic acid composition and antioxidant activity of bran extracts. *Food Chem.* **2019**, *272*, 235-241. DOI: 10.1016/j.foodchem.2018.07.174.
(38) Li, X. C.; Wang, X. Z.; Chen, D. F.; Chen, S. Z. Antioxidant of Protocatechuic Acid. *Functional Foods in Health and Disease* **2011**, *7*, 232-244.
(39) Kawabata, J.; Okamoto, Y.; Kodama, A.; Makimoto, T.; Kasai, T. Oxidative dimers produced from protocatechuic and gallic esters in the DPPH radical scavenging reaction. *J Agric Food Chem* **2002**, *50* (19), 5468-5471.
(40) Samsonowicz, M.; Regulska, E.; Karpowicz, D.; Lesniewska, B. Antioxidant properties of coffee substitutes rich in polyphenols and minerals. *Food Chem.* **2019**, *278*, 101-109. DOI: 10.1016/j.foodchem.2018.11.057.
(41) Xie, Y.; Li, X.; Xu, J.; Jiang, Q.; Xie, H.; He, J.; Chen, D. Two phenolic antioxidants in Suoyang enhance viability of *OH-damaged mesenchymal stem cells: comparison and mechanistic chemistry. *Chem. Cent. J.* **2017**, *11* (1). DOI: 10.1186/s13065-017-0313-1.
(42) Zhang, J. H.; Tian, H. L.; Zhan, P.; Du, F. L.; Zong, A. Z.; Xu, T. C. Isolation and identification of phenolic compounds in Chinese purple yam and evaluation of antioxidant activity. *Lwt-Food Science and Technology* **2018**, *96*, 161-165. DOI: 10.1016/j.lwt.2018.05.019.
(43) Nakilcioglu-Tas, E. The Effects of Sugar Addition and Degree of Roast on the Bioactive Compounds and Antioxidant Activity of Turkish-Style Coffee Brews. *Indian Journal of Pharmaceutical Education and Research* **2018**, *52* (3), 456-466. DOI: 10.5530/ijper.52.3.53.
(44) Cheng, F. F.; Chen, W. J.; Huang, Y. L.; Zhang, C. M.; Shen, Y.; Hai, H.; Zheng, Y. J.; Tang, M. M.; Zhao, S. L.; Liu, J. K. Protective effect of areca inflorescence extract on hydrogen peroxide-induced oxidative damage to human serum albumin. *Food Res. Int.* **2011**, *44* (1), 98-102. DOI: 10.1016/j.foodres.2010.11.005.
(45) Rao, Y. Y.; Zhao, X. X.; Li, Z. L.; Huang, J. H. Phenolic acids induced growth of 3D ordered gold nanoshell composite array as sensitive SERS nanosensor for antioxidant capacity assay. *Talanta* **2018**, *190*, 174-181, DOI: 10.1016/j.talanta.2018.07.069.
(46) Irakli, M.; Chatzopoulou, P.; Ekateriniadou, L. Optimization of ultrasound-assisted extraction of phenolic compounds: Oleuropein, phenolic acids, phenolic alcohols and flavonoids from olive leaves and evaluation of its antioxidant activities. *Industrial Crops and Products* **2018**, *124*, 382-388. DOI: 10.1016/j.indcrop.2018.07.070.
(47) Denk, B.; Kırıkkulak, M.; Güngör, Ş.; Gülhan, M. F.; İnanç, M. E.; Avdatek, F.; Yeni, D.; Taşdemir, U. Can Vanillic Acid, a Phenolic Substance, Be a Useful Tool to Prevent Oxidative Stress Induced by Freezing Ram Sperm? *Reproduction in Domestic Animals* **2025**, *60* (12). DOI: 10.1111/rda.70152.
(48) Al Omairi, N. E.; Radwan, O. K.; Alzahrani, Y. A.; Kassab, R. B. Neuroprotective efficiency of Mangifera indica leaves extract on cadmium-induced cortical damage in rats. *Metabolic Brain Disease* **2018**, *33* (4), 1121-1130. DOI: 10.1007/s11011-018-0222-6.
(49) Rouphael, Y.; Colla, G.; Graziani, G.; Ritieni, A.; Cardarelli, M.; De Pascale, S. Phenolic composition, antioxidant activity and mineral profile in two seed-propagated artichoke cultivars as affected by microbial inoculants and planting time. *Food Chemistry* **2017**, *234*, 10-19. DOI: 10.1016/j.foodchem.2017.04.175.
(50) Anupama, N.; Rani, M. R. P.; Shyni, G. L.; Raghu, K. G. Glucotoxicity results in apoptosis in H9c2 cells via alteration in redox homeostasis linked mitochondrial dynamics and polyol pathway and possible reversal with cinnamic acid. *Toxicology in Vitro* **2018**, *53*, 178-192. DOI: 10.1016/j.tiv.2018.08.010.
(51) Suriano, S.; Iannucci, A.; Codianni, P.; Fares, C.; Russo, M.; Pecchioni, N.; Marciello, U.; Savino, M. Phenolic acids profile, nutritional and phytochemical compounds, antioxidant properties in colored barley grown in southern Italy. *Food Research International* **2018**, *113*, 221-233. DOI: 10.1016/j.foodres.2018.06.072.
(52) Marquez, K.; Contreras, D.; Salgado, P.; Mardones, C. Production of hydroxyl radicals and their relationship with phenolic compounds in white wines. *Food Chemistry* **2019**, *271*, 80-86. DOI: 10.1016/j.foodchem.2018.07.165.
(53) Landete, J. M. Ellagitannins, ellagic acid and their derived metabolites: A review about source, metabolism, functions and health. *Food Research International* **2011**, *44* (5), 1150-1160, Review. DOI: 10.1016/j.foodres.2011.04.027.
(54) Yang, T.; Wang, L.; Zhu, M.; Zhang, L.; Yan, L. Properties and molecular mechanisms of resveratrol: a review. *Pharmazie* **2015**, *70* (8), 501-506.
(55) Li, X. K.; LI, H. Y.; Li, S.; Song, N. N.; Hou, X. D.; Zhou, W. H.; Cui, Y. H.; Ma, L. Advances in study on resveratrol. *Chinese Traditional and Herbal Drugs* **2016**, *47* (14), 2568-2577.
(56) Jaa, A.; de Moura, P. H. B.; Ruiz-Larrea, M. B.; Ruiz Sanz, J. I.; Richard, T. Potential Transformation of Food Resveratrol: Mechanisms and Biological Impact. *Molecules* **2025**, *30* (3). DOI: 10.3390/molecules30030536.
(57) Tang, C. C.; Zhang, W. W.; Cai, H. B.; Ye, Z. Y.; Zhang, X.; Tan, W. S. Resveratrol improves ex vivo expansion of CB-CD34(+) cells via downregulating intracellular reactive oxygen species level. *J. Cell. Biochem.* **2019**, *120* (5), 7778-7787. DOI: 10.1002/jcb.28052.

(58) Morikawa, T.; Xu, F. M.; Matsuda, H.; Yoshikawa, M. Structures of Novel Norstilbene Dimer, Longusone A, and Three New Stilbene Dimers, Longusols A, B, and C, with Antiallergic and Radical Scavenging Activities from Egyptian Natural Medicine Cyperus longus. *Chemical & pharmaceutical bulletin* **2010**, *58* (10), 1379-1385. DOI: 10.1248/cpb.58.1379.
(59) Xiang, T.; Uno, T.; Ogino, F.; Ai, C. Q.; Duo, J.; Sankawa, U. Antioxidant constituents of Caragana tibetica. *Chemical & pharmaceutical bulletin* **2005**, *53* (9), 1204-1206. DOI: 10.1248/cpb.53.1204.
(60) Lu, Y.; Wang, A. H.; Shi, P.; Zhang, H. A Theoretical Study on the Antioxidant Activity of Piceatannol and Isorhapontigenin Scavenging Nitric Oxide and Nitrogen Dioxide Radicals. *PloS one* **2017**, *12* (1), 16. DOI: 10.1371/journal.pone.0169773.
(61) Liu, J.; Li, W. J.; Jiang, S. J.; Guo, Y.; Wang, G. L.; Lin, R. C. Phenolic Acids and Antioxidant Activities of Danshen Injection. *Chemistry of Natural Compounds* **2014**, *50* (1), 83-87. DOI: 10.1007/s10600-014-0872-5.
(62) Perez-Gonzalez, A.; Rebollar-Zepeda, A. M.; Leon-Carmona, J. R.; Galano, A. Reactivity Indexes and O-H Bond Dissociation Energies of a Large Series of Polyphenols: Implications for their Free Radical Scavenging Activity. *Journal of the Mexican Chemical Society* **2012**, *56* (3), 241-249,
(63) Lam, F.; Bradshaw, T. D.; Mao, H.; Roberts, S.; Pan, Y. J.; Wang, S. D. ZJU-6, a novel derivative of Erianin, shows potent anti-tubulin polymerisation and anti-angiogenic activities. *Investigational New Drugs* **2012**, *30* (5), 1899-1907, DOI: 10.1007/s10637-011-9755-9.
(64) Zhang, X. D.; Yu, Y. E.; Cen, Y. S.; Yang, D. F.; Qi, Z. C.; Hou, Z. N.; Han, S. L.; Cai, Z. X.; Liu, K. C. Bivariate Correlation Analysis of the Chemometric Profiles of Chinese Wild Salvia miltiorrhiza Based on UPLC-Qqq-MS and Antioxidant Activities. *Molecules* **2018**, *23* (3), 13. DOI: 10.3390/molecules23030538.
(65) Wang, Y. Q.; Zhuang, G.; Li, S. J. Multiple on-line screening and identification methods for hydroxyl radical scavengers in Yudanshen. *Journal of Pharmaceutical and Biomedical Analysis* **2018**, *156*, 278-283. DOI: 10.1016/j.jpba.2018.04.043.
(66) Saez, V.; Pastene, E.; Vergara, C.; Mardones, C.; Hermosin-Gutierrez, I.; Gomez-Alonso, S.; Gomez, M. V.; Theoduloz, C.; Riquelme, S.; von Baer, D. Oligostilbenoids in Vitis vinifera L. Pinot Noir grape cane extract: Isolation, characterization, in vitro antioxidant capacity and anti-proliferative effect on cancer cells. *Food Chemistry* **2018**, *265*, 101-110, Article; Proceedings Paper. DOI: 10.1016/j.foodchem.2018.05.050.
(67) Sano, S.; Sugiyama, K.; Ito, T.; Katano, Y.; Ishihata, A. Identification of the Strong Vasorelaxing Substance Scirpusin B, a Dimer of Piceatannol, from Passion Fruit (Passiflora edulis) Seeds. *Journal of Agricultural and Food Chemistry* **2011**, *59* (11), 6209-6213. DOI: 10.1021/jf104959t.
(68) Lin, Y. L.; Chang, Y. Y.; Kuo, Y. H.; Shiao, M. S. Anti-lipid-peroxidative principles from Tournefortia sarmentosa. *Journal of Natural Products* **2002**, *65* (5), 745-747. DOI: 10.1021/np010538y.
(69) Chen, C. C.; Huang, Y. L.; Yeh, P. Y.; Ou, J. C. Cyclized geranyl stilbenes from the rhizomes of Helminthostachys zeylanica. *Planta medica* **2003**, *69* (10), 964-967, Article.
(70) Zhang, R.; Kang, K. A.; Piao, M. J.; Lee, K. H.; Jang, H. S.; Park, M. J.; Kim, B. J.; Kim, J. S.; Kim, Y. S.; Ryu, S. Y.; et al. Rhapontigenin from Rheum undulatum protects against oxidative-stress-induced cell damage through antioxidant activity. *Journal of Toxicology and Environmental Health-Part a-Current Issues* **2007**, *70* (13-14), 1155-1166. DOI: 10.1080/15287390701252766.
(71) Yu, W. Z.; Tao, M. R.; Zhao, Y. L.; Hu, X. Q.; Wang, M. F. 4 '- Methoxyresveratrol Alleviated AGE-Induced Inflammation via RAGE-Mediated NF-kappa B and NLRP3 Inflammasome Pathway. *Molecules* **2018**, *23* (6), 13. DOI: 10.3390/molecules23061447.
(72) Tung, N. H.; Kwon, H. J.; Kim, J. H.; Ra, J. C.; Ding, Y.; Kim, J. A.; Kim, Y. H. Anti-influenza diarylheptanoids from the bark of Alnus japonica. *Bioorganic & Medicinal Chemistry Letters* **2010**, *20* (3), 1000-1003. DOI: 10.1016/j.bmcl.2009.12.057.
(73) Huang, K. S.; Zhou, S.; Lin, M.; Wang, Y. H. An isorhapontigenin tetramer and a novel stilbene dimer from Gnetum hainanense. *Planta Med* **2002**, *68* (10), 916-920. DOI: 10.1055/s-2002-34951.
(74) Iliya, I.; Ali, Z.; Tanaka, T.; Iinuma, M.; Furasawa, M.; Nakaya, K.; Shirataki, Y.; Murata, J.; Darnaedi, D.; Matsuura, N.; et al. Three new trimeric stilbenes from Gnetum gnemon. *Chemical & pharmaceutical bulletin* **2003**, *51* (1), 85-88. DOI: 10.1248/cpb.51.85.
(75) Yao, C. S.; Lin, M.; Wang, L. Isolation and biomimetic synthesis of anti-inflammatory stilbenolignans from Gnetum cleistostachyum. *Chem. Pharm. Bull.* **2006**, *54* (7), 1053-1057. DOI: 10.1248/cpb.54.1053.
(76) Dai, S. J.; Mi, Z. M.; Ma, Z. B.; Li, S.; Chen, R. Y.; Yu, D. Q. Bioactive Diels-Alder type adducts from the stem bark of Morus macroura. *Planta medica* **2004**, *70* (8), 758-763. DOI: 10.1055/s-2004-827208.

(77) Cheng, K. J.; Liu, X.; Chen, L.; Lv, J. M.; Qu, F. J.; Pan, X. W.; Li, L.; Cui, X. G.; Gao, Y.; Xu, D. F. alpha-Viniferin activates autophagic apoptosis and cell death by reducing glucocorticoid receptor expression in castration-resistant prostate cancer cells. *Medical Oncology* **2018**, *35* (7), 11. DOI: 10.1007/s12032-018-1163-y.
(78) Ren, X. Y.; Yu, J.; Yin, X. F.; Tu, L. D.; Kong, Q. J. Isolation and purification of five phenolic compounds from the Xinjiang wine grape (Vitis Vinifera) and determination of their antioxidant mechanism at cellular level. *European Food Research and Technology* **2018**, *244* (9), 1569-1579. DOI: 10.1007/s00217-018-3070-z.
(79) He, C.-N.; Bi, W.; Shen, J.; Peng, Y.; Xiao, P.-G. Determination of ten stilbenes and their antioxidant activity of peony seed coat, seed kernel and seed coat extracts. *Zhongguo Zhong yao za zhi = Zhongguo zhongyao zazhi = China journal of Chinese materia medica* **2016**, *41* (6), 1081-1086. DOI: 10.4268/cjcmm20160618.
(80) Ge, H. M.; Yang, W. H.; Zhang, J.; Tan, R. X. Antioxidant Oligostilbenoids from the Stem Wood of Hopea hainanensis. *Journal of Agricultural and Food Chemistry* **2009**, *57* (13), 5756-5761. DOI: 10.1021/jf900756d.
(81) Dai, J. R.; Hallock, Y. F.; Cardellina, J. H.; Boyd, M. R. HIV-inhibitory and cytotoxic oligostilbenes from the leaves of Hopea malibato. *J. Nat. Prod.* **1998**, *61* (3), 351-353. DOI: 10.1021/np970519h.
(82) Li, X. C.; Xie, Y. L.; Xie, H.; Yang, J.; Chen, D. F. pi-pi Conjugation Enhances Oligostilbene's Antioxidant Capacity: Evidence from alpha-Viniferin and Caraphenol A. *Molecules* **2018**, *23* (3), 10. DOI: 10.3390/molecules23030694.
(83) Jin, Q.; Han, X. H.; Hong, S. S.; Lee, C.; Choe, S.; Lee, D.; Kim, Y.; Hong, J. T.; Lee, M. K.; Hwang, B. Y. Antioxidative oligostilbenes from Caragana sinica. *Bioorg Med Chem Lett* **2012**, *22* (2), 973-976. DOI: 10.1016/j.bmcl.2011.12.012.
(84) Wang, G. W.; Wang, H. L.; Capretto, D. A.; Han, Q.; Hu, R. B.; Yang, S. D. Tf2O-catalyzed Friedel-Crafts alkylation to synthesize dibenzo a,d cycloheptene cores and application in the total synthesis of Diptoindonesin D, Pauciflorial F, and (+/-)-Ampelopsin B. *Tetrahedron* **2012**, *68* (26), 5216-5222. DOI: 10.1016/j.tet.2012.03.073.
(85) Matsuura, B. S.; Keylor, M. H.; Li, B.; Lin, Y. X.; Allison, S.; Pratt, D. A.; Stephenson, C. R. J. A Scalable Biomimetic Synthesis of Resveratrol Dimers and Systematic Evaluation of their Antioxidant Activities. *Angewandte Chemie-International Edition* **2015**, *54* (12), 3754-3757. DOI: 10.1002/anie.201409773.
(86) Li, C.; Xu, X. F.; Tab, Z. H.; Wang, X. J.; Pan, Y. J. Resveratrol dimers, nutritional components in grape wine, are selective ROS scavengers and weak Nrf2 activators. *Food Chemistry* **2015**, *173*, 218-223. DOI: 10.1016/j.foodchem.2014.09.165.
(87) Chen, C. J.; Jiang, R.; Wang, G.; Jiao, R. H.; Tancharoen, C.; Sudto, K.; Vajarothai, S.; Hannongbua, S.; Ge, H. M.; Tan, R. X. Oligostilbenoids with Acetylcholinesterase Inhibitory Activity from Dipterocarpus alatus. *Planta medica* **2014**, *80* (17), 1641-1646. DOI: 10.1055/s-0034-1383194.
(88) Chen, J.; Ma, M.; Lu, Y.; Wang, L.; Wu, C.; Duan, H. Rhaponticin from rhubarb rhizomes alleviates liver steatosis and improves blood glucose and lipid profiles in KK/Ay diabetic mice. *Planta Med* **2009**, *75* (5), 472-477. DOI: 10.1055/s-0029-1185304.
(89) Yao, C. S.; Lin, M.; Yang, Q. Y. A new resveratrol trimer derivative from Gnetum brunonianum. *Journal of Asian Natural Products Research* **2012**, *14* (9), 918-922. DOI: 10.1080/10286020.2012.695350.
(90) Erasalo, H.; Hamalainen, M.; Leppanen, T.; Maki-Opas, I.; Laavola, M.; Haavikko, R.; Yli-Kauhaluoma, J.; Moilanen, E. Natural Stilbenoids Have Anti-Inflammatory Properties in Vivo and Down-Regulate the Production of Inflammatory Mediators NO, IL6, and MCP1 Possibly in a PI3K/Akt-Dependent Manner. *Journal of Natural Products* **2018**, *81* (5), 1131-1142. DOI: 10.1021/acs.jnatprod.7b00384.
(91) Lai, Y.; Zhou, C.; Huang, P.; Dong, Z.; Mo, C.; Xie, L.; Lin, H.; Zhou, Z.; Deng, G.; Liu, Y.; et al. Polydatin alleviated alcoholic liver injury in zebrafish larvae through ameliorating lipid metabolism and oxidative stress. *Journal of pharmacological sciences* **2018**, *138* (1), 46-53. DOI: 10.1016/j.jphs.2018.08.007.
(92) Lai, Y. L.; Zhou, C. Y.; Huang, P.; Dong, Z. Y.; Mo, C.; Xie, L. P.; Lin, H. Y.; Zhou, Z. T.; Deng, G. H.; Liu, Y.; et al. Polydatin alleviated alcoholic liver injury in zebrafish larvae through ameliorating lipid metabolism and oxidative stress. *Journal of Pharmacological Sciences* **2018**, *138* (1), 46-53. DOI: 10.1016/j.jphs.2018.08.007.
(93) Park, S. Y.; Jin, M. L.; Wang, Z.; Park, G.; Choi, Y. W. 2,3,4 ',5-tetrahydroxystilbene-2-O-β-D-glucoside exerts anti-inflammatory effects on lipopolysaccharide-stimulated microglia by inhibiting NF-kappa B and activating AMPK/Nrf2 pathways. *Food and Chemical Toxicology* **2016**, *97*, 159-167. DOI: 10.1016/j.fct.2016.09.010.
(94) Xu, S. F.; Liu, J.; Shi, J. S.; Wang, Z. T.; Ji, L. L. 2,3,4 ',5-tetrahydroxystilbene-2-O-β-D-glucoside exacerbates acetaminophen-induced hepatotoxicity by inducing hepatic expression of CYP2E1, CYP3A4 and CYP1A2. *Scientific Reports* **2017**, *7*, 13. DOI: 10.1038/s41598-017-16688-5.
(95) Lee, J. P.; Min, B. S.; An, R. B.; Na, M. K.; Lee, S. M.; Lee, H. K.; Kim, J. G.; Bae, K. H.; Kang, S. S. Stilbenes from the roots of Pleuropterus ciliinervis and their antioxidant activities. *Phytochemistry* **2003**, *64* (3), 759-763. DOI: 10.1016/s0031-9422(03)00417-5.
(96) Park, D.; Kim, H. J.; Jung, S. Y.; Yook, C. S.; Jin, C.; Lee, Y. S. A New Diarylheptanoid Glycoside from the Stem Bark of Alnus hirsuta and Protective Effects of Diarylheptanoid Derivatives in Human HepG2 Cells. *Chemical & pharmaceutical bulletin* **2010**, *58* (2), 238-241. DOI: 10.1248/cpb.58.238.

(97) Lee, M.; Song, J. Y.; Chin, Y. W.; Sung, S. H. Anti-adipogenic diarylheptanoids from Alnus hirsuta f. sibirica on 3T3-L1 cells. *Bioorganic & Medicinal Chemistry Letters* **2013**, *23* (7), 2069-2073. DOI: 10.1016/j.bmcl.2013.01.127.
(98) Wang, L.; Cui, X. Q.; Gong, T.; Yan, R. Y.; Tan, Y. X.; Chen, R. Y. Three new compounds from the barks of Morus nigra. *Journal of Asian Natural Products Research* **2008**, *10* (9), 897-902.
(99) Takasugi, M.; Ishikawa, S. I.; Masamune, T. Albafurans A and B, geranyl 2-phenylbenzofurans from mulberry. *Chemistry Letters* **1982**, (8), 1221-1222.
(100) Rong, Y. Z.; Wang, Z. W.; Zhao, B. DFT-Based Quantum Chemical Studies on Conformational, Electronic and Antioxidant Properties of Isobavachalcone and 4-Hydroxyderricin. *Food Biophys.* **2013**, *8* (4), 250-255. DOI: 10.1007/s11483-013-9296-1.
(101) Jung, H. A.; Jeong, D. M.; Chung, H. Y.; Lim, H. A.; Kim, J. Y.; Yoon, N. Y.; Choi, J. S. Re-evaluation of the antioxidant prenylated flavonoids from the roots of Sophora flavescens. *Biol Pharm Bull* **2008**, *31* (5), 908-915.
(102) Gaur, R.; Yadav, K. S.; Verma, R. K.; Yadav, N. P.; Bhakuni, R. S. In vivo anti-diabetic activity of derivatives of isoliquiritigenin and liquiritigenin. *Phytomedicine* **2014**, *21* (4), 415-422.
(103) Kim, Y. M.; Kim, T. H.; Kim, Y. W.; Yang, Y. M.; Ryu, D. H.; Hwang, S. J.; Lee, J. R.; Kim, S. C.; Kim, S. G. Inhibition of liver X receptor- Î±-dependent hepatic steatosis by isoliquiritigenin, a licorice antioxidant flavonoid, as mediated by JNK1 inhibition. *Free Radic Biol Med* **2010**, *49* (11), 1722-1734.
(104) Zhan, C.; Yang, J. Protective effects of isoliquiritigenin in transient middle cerebral artery occlusion-induced focal cerebral ischemia in rats. *Pharmacological Research* **2006**, *53* (3), 303-309.
(105) Zhou, Y. Z.; Li, X.; Gong, W. X.; Tian, J. S.; Gao, X. X.; Gao, L.; Zhang, X.; Du, G. H.; Qin, X. M. Protective effect of isoliquiritin against corticosterone-induced neurotoxicity in PC12 cells. *Food & Function* **2017**, *8* (3), 1235.
(106) Chen, J. J.; Cheng, M. J.; Shu, C. W.; Sung, P. J.; Lim, Y. P.; Cheng, L. Y.; Wang, S. L.; Chen, L. C. A New Chalcone and Antioxidant Constituents of Glycyrrhiza glabra. *Chemistry of Natural Compounds* **2017**, *53* (4), 632-634.
(107) Cheng, Z. J.; Kuo, S. C.; Chan, S. C.; Ko, F. N.; Teng, C. M. Antioxidant properties of butein isolated from Dalbergia odorifera. *Biochim Biophys Acta* **1998**, *1392* (2–3), 291-299.
(108) Nakanishi, N.; Fukuoh, A.; Kang, D.; Iwai, S.; Kuraoka, I. Effects of DNA lesions on the transcription reaction of mitochondrial RNA polymerase: implications for bypass RNA synthesis on oxidative DNA lesions. *Mutagenesis* **2013**, *28* (1), 117-123. DOI: 10.1093/mutage/ges060.
(109) Tian, X.; Liu, C.; Jiang, H. L.; Yan, Z.; Han, J.; Ju, L.; Meng, C. Cardioprotection provided by Echinatin against ischemia/reperfusion in isolated rat hearts. *Bmc Cardiovasc Disord* **2016**, *16* (1), 119.
(110) Lv, H.; Xiao, Q.; Zhou, J.; Feng, H.; Liu, G.; Ci, X. Licochalcone A Upregulates Nrf2 Antioxidant Pathway and Thereby Alleviates Acetaminophen-Induced Hepatotoxicity. *Frontiers in Pharmacology* **2018**, *9*, 147.
(111) Han, J.; Wang, D.; Yu, B.; Wang, Y.; Ren, H.; Zhang, B.; Wang, Y.; Zheng, Q. Cardioprotection against ischemia/reperfusion by licochalcone B in isolated rat hearts. *Oxidative Medicine & Cellular Longevity* **2014**, *2014*, 134862.
(112) Zhou, M.; Liu, L.; Wang, W.; Han, J.; Ren, H.; Zheng, Q.; Wang, D. Role of licochalcone C in cardioprotection against ischemia/reperfusion injury of isolated rat heart via antioxidant, anti-inflammatory, and anti-apoptotic activities. *Life Sciences* **2015**, *132*, 27-33.
(113) Yuan, X.; Niu, H.; Wang, P.; Lu, J.; Zhao, H.; Liu, S.; Zheng, Q.; Li, C. Cardioprotective Effect of Licochalcone D against Myocardial Ischemia/Reperfusion Injury in Langendorff-Perfused Rat Hearts. *PloS one* **2015**, *10* (6), e0128375.
(114) Kim, S. S.; Lim, J.; Bang, Y.; Gal, J.; Lee, S. U.; Cho, Y. C.; Yoon, G.; Kang, B. Y.; Cheon, S. H.; Choi, H. J. Licochalcone E activates Nrf2/antioxidant response element signaling pathway in both neuronal and microglial cells: therapeutic relevance to neurodegenerative disease. *Journal of Nutritional Biochemistry* **2012**, *23* (10), 1314-1323.
(115) Victor; Kuete; Jaures; Noumedem; Frederic; Nana. Chemistry and Pharmacology of 4-Hydroxylonchocarpin: A Review. *Chinese Journal of Integrative Medicine* **2013**, *19* (6), 475-480.
(116) Adem, F. A.; Kuete, V.; Mbaveng, A. T.; Heydenreich, M.; Koch, A.; Ndakala, A.; Irungu, B.; Yenesew, A.; Efferth, T. Cytotoxic flavonoids from two Lonchocarpus species. *Natural product research* **2018**, 1-9.
(117) Dang, Y.; Ling, S.; Duan, J.; Ma, J.; Ni, R.; Xu, J. W. Bavachalcone-induced manganese superoxide dismutase expression through the AMP-activated protein kinase pathway in human endothelial cells. *Pharmacology* **2015**, *95* (3-4), 105.

(118) Shakya, R.; Ye, J.; Rommens, C. M. Altered leaf colour is associated with increased superoxide-scavenging activity in aureusidin-producing transgenic plants. *Plant Biotechnology Journal* **2012**, *10* (9), 1046-1055.
(119) Calis, I.; Kuruüzüm, A.; Demirezer, L. O.; Sticher, O.; Ganci, W.; Rüedi, P. Phenylvaleric acid and flavonoid glycosides from Polygonum salicifolium. *Journal of Natural Products* **1999**, *62* (8), 1101.
(120) Cioffi, G.; Escobar, L. A.; De, T. N. Antioxidant chalcone glycosides and flavanones from Maclura (Chlorophora) tinctoria. *Journal of Natural Products* **2003**, *66* (8), 1061-1064.
(121) Cheng, Z.; Lin, C.; Hwang, T.; Teng, C. Broussochalcone A, a potent antioxidant and effective suppressor of inducible nitric oxide synthase in lipopolysaccharide-activated macrophages. *Biochemical Pharmacology* **2001**, *61* (8), 939-946.
(122) Padhye, S.; Ahmad, A.; Oswal, N.; Sarkar, F. H. Emerging role of Garcinol, the antioxidant chalcone from Garcinia indica Choisy and its synthetic analogs. *Journal of Hematology & Oncology* **2009**, *2* (1), 38.
(123) Isa, N. M.; Abdelwahab, S. I.; Mohan, S.; Abdul, A. B.; Sukari, M. A.; Taha, M. M.; Syam, S.; Narrima, P.; Sch, C.; Ahmad, S. In vitro anti-inflammatory, cytotoxic and antioxidant activities of boesenbergin A, a chalcone isolated from Boesenbergia rotunda (L.) (fingerroot). *Brazilian Journal of Medical & Biological Research* **2012**, *45* (6), 524-530.
(124) Wang, C.; Ma, H.; Zhang, S.; Wang, Y.; Liu, J.; Xiao, X. Safflor yellow B suppresses pheochromocytoma cell (PC12) injury induced by oxidative stress via antioxidant system and Bcl-2 /Bax pathway. *Naunyn-Schmiedeberg's Archives of Pharmacology* **2009**, *380* (2), 135-142.
(125) Li, Y.; Piao, D.; Zhang, H.; Kim, T.; Lee, S. H.; Chang, H. W.; Woo, M. H.; Son, J. K. Quality evaluation of Carthami Flos by HPLC-UV. *Archives of Pharmacal Research* **2015**, *38* (5), 1-9.
(126) Yue, S.; Tang, Y.; Xu, C.; Li, S.; Zhu, Y.; Duan, J. A. Two New Quinochalcone C-Glycosides from the Florets of Carthamus tinctorius. *International Journal of Molecular Sciences* **2014**, *15* (9), 16760-16771.
(127) Yoon, H. R.; Paik, Y. S. Radical-Scavenging Activities of Four Quinochalcones of Safflower. *Journal of the Korean Society for Applied Biological Chemistry* **2008**, *51* (4), 346-348.
(128) Pinner, K. D.; Wales, C. T. K.; Gristock, R. A.; Vo, H. T.; So, N.; Jacobs, A. T. Flavokawains A and B from kava (Piper methysticum) activate heat shock and antioxidant responses and protect against hydrogen peroxide-induced cell death in HepG2 hepatocytes. *Pharmaceutical Biology* **2016**, *54* (9), 1.
(129) Li, N.; Liu, J. H.; Zhang, J.; Yu, B. Y. Comparative Evaluation of Cytotoxicity and Antioxidative Activity of 20 Flavonoids. *J Agric Food Chem* **2008**, *56* (10), 3876-3883.
(130) Voon, F.-L.; Sulaiman, M. R.; Akhtar, M. N.; Idris, M. F.; Akira, A.; Perimal, E. K.; Israf, D. A.; Ming-Tatt, L. Cardamonin (2′,4′-dihydroxy-6′-methoxychalcone) isolated from Boesenbergia rotunda (L.) Mansf. inhibits CFA-induced rheumatoid arthritis in rats. *European Journal of Pharmacology* **2017**, *794*, 127-134.
(131) De, S. S.; Eckers, A.; Wehrend, C.; Micoogullari, M.; Sies, H.; Stahl, W.; Steinbrenner, H. Interplay between the chalcone cardamonin and selenium in the biosynthesis of Nrf2-regulated antioxidant enzymes in intestinal Caco-2 cells. *Free radical biology & medicine* **2016**, *91* (1-2), 164-171.
(132) Wen, R.; Lv, H. N.; Jiang, Y.; Tu, P. F. Anti-inflammatory flavone and chalcone derivatives from the roots of Pongamia pinnata (L.) Pierre. *Phytochemistry* **2018**, *149*, 56.
(133) Lima, N. M.; Andrade, J. I.; Lima, K. C.; dos Santos, F. N.; Barison, A.; Salomé, K. S.; Matsuura, T.; Nunez, C. V. Chemical profile and biological activities of Deguelia duckeana A.M.G. Azevedo (Fabaceae). *Natural product research* **2013**, *27* (4-5), 425-432.
(134) Saha, M. M.; Mallik, U. K.; Mallik, A. K. A chromenoflavanone and two caffeic esters from Pongamia glabra. *Phytochemistry* **1991**, *30* (11), 3834-3836.
(135) Magalhães, A. F.; Tozzi, A. M. G. A.; Sales, B. H. L. N.; Magalhães, E. G. Twenty-three flavonoids from Lonchocarpus subglaucescens. *Phytochemistry* **1996**, *42* (5), 1459-1471.
(136) Sritularak, B.; Likhitwitayawuid, K. Flavonoids from the pods of Millettia erythrocalyx. *Phytochemistry* **2006**, *67* (8), 812-817.
(137) Chang, L. C.; Gerhäuser, C.; Song, L.; Farnsworth, N. R.; Pezzuto, J. M.; Kinghorn, A. D. Activity-guided isolation of constituents of Tephrosia purpurea with the potential to induce the phase II enzyme, quinone reductase. *Journal of Natural Products* **1997**, *60* (9), 869.
(138) Caamalfuentes, E. E.; Perazasánchez, S. R.; Torrestapia, L. W.; Moopuc, R. E. Isolation and Identification of Cytotoxic Compounds from Aeschynomene fascicularis, a Mayan Medicinal Plant. *Molecules* **2015**, *20* (8), 13563-13574.
(139) Kim, Y. E.; Choi, H. C.; Lee, I. C.; Yuk, D. Y.; Lee, H.; Choi, B. Y. 3-Deoxysappanchalcone Promotes Proliferation of Human Hair Follicle Dermal Papilla Cells and Hair Growth in C57BL/6 Mice by Modulating WNT/β-Catenin and STAT Signaling. *Biomolecules & Therapeutics* **2016**, *24* (6), 572-580.
(140) Seo, H.; Kim, S.; Mahmud, H. A.; Islam, M. I.; Nam, K. W.; Lee, B. E.; Lee, H.; Cho, M. L.; Shin, H. M.; Song, H. Y. In vitro Antitubercular Activity of 3-Deoxysappanchalcone Isolated From the Heartwood of Caesalpinia sappan Linn. *Phytotherapy Research Ptr* **2017**, *31* (1).
(141) Seo, K. H.; Lee, D. Y.; Jeong, R. H.; Lee, D. S.; Kim, Y. E.; Hong, E. K.; Kim, Y. C.; Baek, N. I. Neuroprotective effect of prenylated arylbenzofuran and flavonoids from morus alba fruits on glutamate-induced oxidative injury in HT22 hippocampal cells. *Journal of medicinal food* **2015**, *18* (4), 403.

(142) Jiang, Y.; Zhang, G.; Yan, D.; Yang, H.; Ye, Z.; Ma, T. Bioactivity-Guided Fractionation of the Traditional Chinese Medicine Resina Draconis Reveals Loureirin B as a PAI-1 Inhibitor. *Evid Based Complement Alternat Med* **2017**, *2017*, 9425963. DOI: 10.1155/2017/9425963.
(143) Wang, X. H. Production of dragon's blood in Dracaena cochinchinensis plants by inoculation of Fusarium proliferatum. *Plant Science* **2011**, *180* (2), 292-299.
(144) Tang, N.; Yan, W. Solubilities of Naringin Dihydrochalcone in Pure Solvents and Mixed Solvents at Different Temperatures. **2016**.
(145) Rezk, B. M.; Haenen, G. R.; Wj, V. D. V.; Bast, A. The antioxidant activity of phloretin: the disclosure of a new antioxidant pharmacophore in flavonoids. *Biochemical & Biophysical Research Communications* **2002**, *295* (1), 9-13.
(146) Zhao, Y. M.; Wang, J.; Song, G. M.; Song, X. B.; Liu, D. L.; Xie, W. L. Study on the Dynamic Changs of Phlordzin and Phloretin in Leave and Branches of Apple Tree. *Food Research & Development* **2013**.
(147) Shindo, K.; Kato, M.; Kinoshita, A.; Kobayashi, A.; Koike, Y. Analysis of Antioxidant Activities Contained in theBoesenbergia pandurataSchult. Rhizome. *Bioscience, Biotechnology, and Biochemistry* **2014**, *70* (9), 2281-2284. DOI: 10.1271/bbb.60086.
(148) Sun, Y.; Li, W.; Liu, Z. Preparative isolation, quantification and antioxidant activity of dihydrochalcones from Sweet Tea (Lithocarpus polystachyus Rehd.). *Journal of Chromatography B* **2015**, *1002*, 372-378. DOI: 10.1016/j.jchromb.2015.08.045.
(149) Tian, Y.; Li, X.; Xie, H.; Wang, X.; Xie, Y.; Chen, C.; Chen, D. Protective Mechanism of the Antioxidant Baicalein toward Hydroxyl Radical-Treated Bone Marrow-Derived Mesenchymal Stem Cells. *Molecules* **2018**, *23* (1). DOI: 10.3390/molecules23010223.
(150) Ahmed, S. I.; Hayat, M. Q.; Tahir, M.; Mansoor, Q.; Ismail, M.; Keck, K.; Bates, R. B. Pharmacologically active flavonoids from the anticancer, antioxidant and antimicrobial extracts of Cassia angustifolia Vahl. *BMC Complement. Altern. Med.* **2016**, *16*.
(151) Fonseca, S. F.; Lima, D. B.; Alves, D.; Jacob, R. G.; Perin, G.; Lenardao, E. J.; Savegnago, L. Synthesis, characterization and antioxidant activity of organoselenium and organotellurium compound derivatives of chrysin. *New Journal of Chemistry* **2015**, *39* (4), 3043-3050.
(152) Wang CZ; Yuan HH; Bao XL; MB., L. In vitro antioxidant and cytotoxic properties of ethanol extract of Alpinia oxyphylla fruits. *Pharm Biol* **2013**, *51* (11), 1419–1425.
(153) Shieh, D. E.; Liu, L. T.; Lin, C. C. Antioxidant and free radical scavenging effects of baicalein, baicalin and wogonin. *Anticancer Research* **2000**, *20* (5a), 2861-2865.
(154) Dong, L. L.; Fu, Y. J.; Zu, Y. G.; Luo, M.; Wang, W.; Li, C. Y.; Mu, P. S. An enhanced preparation and purification of the major antioxidants baicalein and wogonin from Scutellariae radix. *Food Chem.* **2012**, *133* (2), 430-436.
(155) Gao, Y.; Zhao, J. T.; Zu, Y. G.; Fu, Y. J.; Liang, L.; Luo, M.; Wang, W.; Efferth, T. Antioxidant properties, superoxide dismutase and glutathione reductase activities in HepG2 cells with a fungal endophyte producing apigenin from pigeon pea [Cajanus cajan (L.) Millsp.]. *Food Research International* **2012**, *49* (1), 147-152.
(156) Zheng, R. L.; Xing, G. X.; Ju, Y.; Jia, Z. J. Flavonoids from Saussurea stella Maxim as superoxide scavengers and antioxidants. *Indian Journal of Chemistry Section B-Organic Chemistry Including Medicinal Chemistry* **1997**, *36* (12), 1201-1203.
(157) Hu, J.; Ma, W.; Li, N.; Wang, K. J. Antioxidant and Anti-Inflammatory Flavonoids from the Flowers of Chuju, a Medical Cultivar of Chrysanthemum Morifolim Ramat. *Journal of the Mexican Chemical Society* **2017**, *61* (4), 282-289, Article.
(158) Chen, Z. J.; Kong, S. S.; Song, F. F.; Li, L. P.; Jiang, H. D. Pharmacokinetic study of luteolin, apigenin, chrysoeriol and diosmetin after oral administration of Flos Chrysanthemi extract in rats. *Fitoterapia* **2012**, *83* (8), 1616-1622.
(159) Panuccio, M. R.; Fazio, A.; Papalia, T.; Barreca, D. Antioxidant Properties and Flavonoid Profile in Leaves of CalabrianLavandula multifidaL., an Autochthon Plant of Mediterranean Southern Regions. *Chemistry & Biodiversity* **2016**, *13* (4), 416-421. DOI: 10.1002/cbdv.201500115.
(160) Liao, W. Z.; Ning, Z. X.; Chen, L. Y.; Wei, Q. Y.; Yuan, E. D.; Yang, J. G.; Ren, J. Y. Intracellular Antioxidant Detoxifying Effects of Diosmetin on 2,2-Azobis(2-amidinopropane) Dihydrochloride (AAPH)-Induced Oxidative Stress through Inhibition of Reactive Oxygen Species Generation. *Journal of Agricultural and Food Chemistry* **2014**, *62* (34), 8648-8654.
(161) Zhang, C.-F.; Zhang, S.-L.; He, X.; Yang, X.-L.; Wu, H.-T.; Lin, B.-Q.; Jiang, C.-P.; Wang, J.; Yu, C.-H.; Yang, Z.-L.; et al. Antioxidant effects of Genkwa flos flavonoids on Freund′s adjuvant-induced rheumatoid arthritis in rats. *Journal of Ethnopharmacology* **2014**, *153* (3), 793-800. DOI: 10.1016/j.jep.2014.03.046.
(162) Yang, J.; Kim, J. S.; Jeong, H. J.; Kang, H. H.; Cho, J. C.; Yeom, H. M.; Kim, M. J. Determination of antioxidant and alpha-glucosidase inhibitory activities and luteolin contents of Chrysanthemum morifolium Ramat extracts. *African Journal of Biotechnology* **2011**, *10* (82), 19197-19202.
(163) Gokbulut, A.; Satilmis, B.; Batcioglu, K.; Cetin, B.; Sarer, E. Antioxidant activity and luteolin content of Marchantia polymorpha L. *Turkish Journal of Biology* **2012**, *36* (4), 381-385.

(164) An, F.; Yang, G. D.; Tian, J. M.; Wang, S. H. Antioxidant effects of the orientin and vitexin in Trollius chinensis Bunge in D-galactose-aged mice. *Neural Regeneration Research* **2012**, *7* (33), 2565-2575.

(165) Gu, C. B.; Ma, H.; Ning, W. J.; Niu, L. L.; Han, H. Y.; Yuan, X. H.; Fu, Y. J. Characterization, culture medium optimization and antioxidant activity of an endophytic vitexin-producing fungus Dichotomopilus funicola Y3 from pigeon pea [Cajanus cajan (L.) Millsp.]. *Journal of Applied Microbiology* **2018**, *125* (4), 1054-1065.

(166) Yang, P.; Kong, X. Z.; Cheng, C. M.; Li, C. Y.; Yang, X. M.; Zhao, X. E. Synthesis and biological evaluation of 8-substituted and deglucuronidated scutellarin and baicalin analogues as antioxidant responsive element activators. *Science China-Chemistry* **2011**, *54* (10), 1565-1575.

(167) Rehecho, S.; Hidalgo, O.; de Cirano, M. G. I.; Navarro, I.; Astiasaran, I.; Ansorena, D.; Cavero, R. Y.; Calvo, M. I. Chemical composition, mineral content and antioxidant activity of Verbena officinalis L. *Lwt-Food Science and Technology* **2011**, *44* (4), 875-882.

(168) Tuzun, B. S.; Hajdu, Z.; Orban-Gyapai, O.; Zomborszki, Z. P.; Jedlinszki, N.; Forgo, P.; Kivcak, B.; Hohmann, J. Isolation of Chemical Constituents of Centaurea virgata Lam. and Xanthine Oxidase Inhibitory Activity of the Plant Extract and Compounds. *Medicinal Chemistry* **2017**, *13* (5), 498-502. DOI: 10.2174/1573406413666161219161946.

(169) Peng, Z. F.; Strack, D.; Baumert, A.; Subramaniam, R.; Goh, N. K.; Chia, T. F.; Tan, S. N.; Chia, L. S. Antioxidant flavonoids from leaves of Polygonum hydropiper L. *Phytochemistry* **2003**, *62* (2), 219-228. DOI: 10.1016/s0031-9422(02)00504-6.

(170) Venditti, A.; Frezza, C.; Bianco, A.; Serafini, M.; Cianfaglione, K.; Nagy, D. U.; Iannarelli, R.; Caprioli, G.; Maggi, F. Polar Constituents, Essential Oil and Antioxidant Activity of Marsh Woundwort (Stachys palustris L.). *Chemistry & Biodiversity* **2017**, *14* (3), 8. DOI: 10.1002/cbdv.201600401.

(171) Fu, M. Q.; Xu, Y. J.; Chen, Y. L.; Wu, J. J.; Yu, Y. S.; Zou, B.; An, K. J.; Xiao, G. S. Evaluation of bioactive flavonoids and antioxidant activity in Pericarpium Citri Reticulatae (Citrus reticulata 'Chachi') during storage. *Food Chemistry* **2017**, *230*, 649-656. DOI: 10.1016/j.foodchem.2017.03.098.

(172) Mehmood, T.; Khan, M. R.; Shabbir, M. A.; Zia, M. A. Phytochemical profiling and HPLC quantification of citrus peel from different varieties. *Progress in Nutrition* **2018**, *20*, 279-288. DOI: 10.23751/pn.v20i1-S.6357.

(173) Wu, C.; Zhao, J.; Chen, Y.; Li, T.; Zhu, R.; Zhu, B.; Zhang, Y. Tangeretin protects human brain microvascular endothelial cells against oxygen-glucose deprivation-induced injury. *Journal of cellular biochemistry* **2018**. DOI: 10.1002/jcb.27762.

(174) Debnath, T.; Park, P.-J.; Deb Nath, N. C.; Samad, N. B.; Park, H. W.; Lim, B. O. Antioxidant activity of Gardenia jasminoides Ellis fruit extracts. *Food Chemistry* **2011**, *128* (3), 697-703. DOI: 10.1016/j.foodchem.2011.03.090.

(175) Jian, S.; Xue-Mei, H.; Mou-Ming, Z.; Li, L.; Chang-Bao, L.; Yi, D. Antioxidant and nitrite-scavenging capacities of phenolic compounds from sugarcane (Saccharum officinarum L.) tops. *Molecules* **2014**, *19* (9), 13147-13160.

(176) Suksamrarn, A.; Poomsing, P.; Aroonrerk, N.; Punjanon, T.; Suksamrarn, S.; Kongkun, S. Antimycobacterial and antioxidant flavones fromLimnophila geoffrayi. *Archives of Pharmacal Research* **2003**, *26* (10), 816-820. DOI: 10.1007/bf02980026.

(177) Cypriani, B.; Limasset, B.; Carrie, M. L.; Doucen, C. L.; Roussie, M.; Paulet, A. D.; Damon, M. Antioxidant activity of micronized diosmin on oxygen species from stimulated human neutrophils. *Biochemical Pharmacology* **1993**, *45* (7), 1531-1535.

(178) Dwiecki, K.; Neunert, G.; Polewski, P.; Polewski, K. Antioxidant activity of daidzein, a natural antioxidant, and its spectroscopic properties in organic solvents and phosphatidylcholine liposomes. *J Photoch Photobio B* **2009**, *96* (3), 242-248.

(179) Zhang, D. Y.; Zu, Y. G.; Fu, Y. J.; Luo, M.; Gu, C. B.; Wang, W.; Yao, X. H. Negative pressure cavitation extraction and antioxidant activity of biochanin A and genistein from the leaves of Dalbergia odorifera T. Chen. *Sep Purif Technol* **2011**, *83*, 91-99.

(180) Chen, C. Y.; Zu, Y. G.; Fu, Y. J.; Luo, M.; Zhao, C. J.; Wang, W.; Zhao, B. S.; Li, J.; Efferth, T. Preparation and antioxidant activity of Radix Astragali residues extracts rich in calycosin and formononetin. *Biochem Eng J* **2011**, *56* (1-2), 84-93.

(181) Chen, Y.; Chang, S. K. C. Macronutrients, Phytochemicals, and Antioxidant Activity of Soybean Sprout Germinated with or without Light Exposure. *Journal of Food Science* **2015**, *80* (6), S1391-S1398. DOI: 10.1111/1750-3841.12868.

(182) Jung, H. A.; Kim, A. R.; Chung, H. Y.; Choi, J. S. In vitro antioxidant activity of some selectedprunus species in Korea. *Archives of Pharmacal Research* **2002**, *25* (6), 865-872. DOI: 10.1007/bf02977006.

(183) Shi, S. Y.; Ma, Y. J.; Zhang, Y. P.; Liu, L. L.; Liu, Q.; Peng, M. J.; Xiong, X. Systematic separation and purification of 18 antioxidants from Pueraria lobata flower using HSCCC target-guided by DPPH-HPLC experiment. *Separation and Purification Technology* **2012**, *89*, 225-233. DOI: 10.1016/j.seppur.2012.01.041.

(184) Xu, H. Y.; Zheng, H. C.; Zhang, H. W.; Zhang, J. Y.; Ma, C. M. Comparison of Antioxidant Constituents of Agriophyllum squarrosum Seed with Conventional Crop Seeds. *Journal of Food Science* **2018**, *83* (7), 1823-1831. DOI: 10.1111/1750-3841.14159.

(185) Fu, Y.; Chen, J.; Li, Y. J.; Zheng, Y. F.; Li, P. Antioxidant and anti-inflammatory activities of six flavonoids separated from licorice. *Food Chem.* **2013**, *141* (2), 1063-1071.

(186) Ji, S.; Li, Z.; Song, W.; Wang, Y.; Liang, W.; Li, K.; Tang, S.; Wang, Q.; Qiao, X.; Zhou, D. Bioactive Constituents of Glycyrrhiza uralensis (Licorice): Discovery of the Effective Components of a Traditional Herbal Medicine. *Journal of Natural Products* **2017**, *79* (2), 281.

(187) Qiu, D.; Guo, J.; Yu, H.; Yan, J.; Yang, S.; Li, X.; Zhang, Y.; Sun, J.; Cong, J.; He, S.; et al. Antioxidant phenolic compounds isolated from wild Pyrus ussuriensis Maxim. fruit peels and leaves. *Food Chemistry* **2018**, *241*, 182-187. DOI: 10.1016/j.foodchem.2017.08.072.

(188) Meng, Z.; Zhao, J.; Duan, H.; Guan, Y.; Zhao, L. Green and efficient extraction of four bioactive flavonoids from Pollen Typhae by ultrasound-assisted deep eutectic solvents extraction. *J Pharm Biomed Anal* **2018**, *161*, 246-253. DOI: 10.1016/j.jpba.2018.08.048.

(189) Zhang, X. F.; Chen, J.; Yang, J. L.; Shi, Y. P. UPLC-MS/MS analysis for antioxidant components of Lycii Fructus based on spectrum-effect relationship. *Talanta* **2018**, *180*, 389-395. DOI: 10.1016/j.talanta.2017.12.078.

(190) Yuan, Q. X.; Zhao, L. Y. The Mulberry (Morus alba L.) Fruit-A Review of Characteristic Components and Health Benefits. *Journal of Agricultural and Food Chemistry* **2017**, *65* (48), 10383-10394. DOI: 10.1021/acs.jafc.7b03614.

(191) Mocan, A.; Schafberg, M.; Crișan, G.; Rohn, S. Determination of lignans and phenolic components of Schisandra chinensis (Turcz.) Baill. using HPLC-ESI-ToF-MS and HPLC-online TEAC: Contribution of individual components to overall antioxidant activity and comparison with traditional antioxidant assays. *Journal of Functional Foods* **2016**, *24*, 579-594. DOI: 10.1016/j.jff.2016.05.007.

(192) Quispe, C.; Villalobos, M.; Bórquez, J.; Simirgiotis, M. Chemical Composition and Antioxidant Activity of Aloe vera from the Pica Oasis (Tarapacá, Chile) by UHPLC-Q/Orbitrap/MS/MS. *Journal of Chemistry* **2018**, *2018*, 1-12. DOI: 10.1155/2018/6123850.

(193) Divakaran, S. A.; Hema, P. S.; Nair, M. S.; Nair, C. K. K. Antioxidant capacity and radioprotective properties of the flavonoids galangin and kaempferide isolated from Alpinia galanga L. (Zingiberaceae) against radiation induced cellular DNA damage. *International Journal of Radiation Research* **2013**, *11* (2), 81-89.

(194) Liu, P. F.; Deng, T. S.; Hou, X. L.; Wang, J. G. Antioxidant Properties of Isolated Isorhamnetin from the Sea Buckthorn Marc. *Plant Foods for Human Nutrition* **2009**, *64* (2), 141-145.

(195) Yao, Y. S.; Lin, G. B.; Xie, Y.; Ma, P.; Li, G. W.; Meng, Q. C.; Wu, T. Preformulation studies of myricetin: a natural antioxidant flavonoid. *Pharmazie* **2014**, *69* (1), 19-26.

(196) Yang, J. Y.; Lee, H. S. Evaluation of Antioxidant and Antibacterial Activities of Morin Isolated from Mulberry Fruits (Morus alba L.). *Journal of the Korean Society for Applied Biological Chemistry* **2012**, *55* (4), 485-489.

(197) Hatano, T.; Takagi, M.; Ito, H.; Yoshida, T. Phenolic constituents of liquorice .7. A new chalcone with a potent radical scavenging activity and accompanying phenolics from liquorice. *Chemical & Pharmaceutical Bulletin* **1997**, *45* (9), 1485-1492, Article.

(198) GuzmÃ n-GutiÃ©rrez, S. L.; Nieto-Camacho, A.; Castillo-Arellano, J. I.; Huerta-Salazar, E.; Hernã, n.-P. G.; Silva-Miranda, M.; Argã¼Ello-Nã, j. O.; Sepã¹lveda-Robles, O.; Espitia, C. I.; Reyes-Chilpa, R. Mexican Propolis: A Source of Antioxidants and Anti-Inflammatory Compounds, and Isolation of a Novel Chalcone and Îµ-Caprolactone Derivative. *Molecules* **2018**, *23* (2), 334.

(199) Bucar, F., .; Resch, M., .; Bauer, R., .; Burits, M., .; Knauder, E., .; Schubert-Zsilavecz, M.. 5-Methylflavasperone and rhamnetin from Guiera senegalensis and their antioxidative and 5-lipoxygenase inhibitory activity. *Pharmazie* **2010**, *30* (13), no-no.

(200) Zhao, L.-j.; Liu, W.; Xiong, S.-h.; Tang, J.; Lou, Z.-h.; Xie, M.-x.; Xia, B.-h.; Lin, L.-m.; Liao, D.-f. Determination of Total Flavonoids Contents and Antioxidant Activity of Ginkgo biloba Leaf by Near-Infrared Reflectance Method. *International Journal of Analytical Chemistry* **2018**, *2018*, 1-7. DOI: 10.1155/2018/8195784.

(201) Li, X. C.; Wang, L.; Han, W. J.; Mai, W. Q.; Han, L.; Chen, D. F. Amentoflavone protects against hydroxyl radical-induced DNA damage via antioxidant mechanism. *Turkish Journal of Biochemistry-Turk Biyokimya Dergisi* **2014**, *39* (1), 30-U58.

(202) Zheng, X.-k.; Wang, W.-w.; Zhang, L.; Su, C.-f.; Wu, Y.-y.; Ke, Y.-y.; Hou, Q.-w.; Liu, Z.-y.; Gao, A.-s.; Feng, W.-s. Antihyperlipidaemic and antioxidant effect of the total flavonoids inSelaginella tamariscina(Beauv.) Spring in diabetic mice. *Journal of Pharmacy and Pharmacology* **2013**, *65* (5), 757-766. DOI: 10.1111/jphp.12035.

(203) Li, X.; Huang, Y.; Chen, D. Protective Effect against Hydroxyl-induced DNA Damage and Antioxidant Activity of Citri reticulatae Pericarpium. *Adv Pharm Bull* **2013**, *3* (1), 175-181. DOI: 10.5681/apb.2013.029.

(204) Barreca, D.; Bisignano, C.; Ginestra, G.; Bisignano, G.; Bellocco, E.; Leuzzi, U.; Gattuso, G. Polymethoxylated, C-and O-glycosyl flavonoids in tangelo (Citrus reticulate Citrus paradisi) juice and their influence on antioxidant properties. *Food Chem* **2013**, *141*, 1481-1488.
(205) Huang, Y.; Zhou, L. S.; Yan, L.; Ren, J.; Zhou, D. X.; Li, S. S. Alpinetin inhibits lipopolysaccharide-induced acute kidney injury in mice. *International Immunopharmacology* **2015**, *28* (2), 1003-1008.
(206) Miler, M.; Zivanovic, J.; Ajdzanovic, V.; Orescanin-Dusic, Z.; Milenkovic, D.; Konic-Ristic, A.; Blagojevic, D.; Milosevic, V.; Sosic-Jurjevic, B. Citrus flavanones naringenin and hesperetin improve antioxidant status and membrane lipid compositions in the liver of old-aged Wistar rats. *Experimental Gerontology* **2016**, *84*, 49-60.
(207) Liu, R. X.; Wang, Q.; Guo, H. Z.; Li, L.; Bi, K. S.; Guo, D. A. Simultaneous determination of 10 major flavonoids in Dalbergia odorifera by high performance liquid chromatography. *Journal of Pharmaceutical and Biomedical Analysis* **2005**, *39* (3-4), 469-476.
(208) Johnson, J.; Maher, P.; Hanneken, A. The Flavonoid, Eriodictyol, Induces Long-term Protection in ARPE-19 Cells through Its Effects on Nrf2 Activation and Phase 2 Gene Expression. *Investigative Ophthalmology & Visual Science* **2009**, *50* (5), 2398-2406.
(209) Martins, N.; Barros, L.; Dueñas, M.; Santos-Buelga, C.; Ferreira, I. C. F. R. Characterization of phenolic compounds and antioxidant properties of Glycyrrhiza glabra L. rhizomes and roots. *RSC Advances* **2015**, *5* (34), 26991-26997. DOI: 10.1039/c5ra03963k.
(210) Aiello, F.; Armentano, B.; Polera, N.; Carullo, G.; Loizzo, M. R.; Bonesi, M.; Cappello, M. S.; Capobianco, L.; Tundis, R. From Vegetable Waste to New Agents for Potential Health Applications: Antioxidant Properties and Effects of Extracts, Fractions and Pinocembrin from Glycyrrhiza glabra L. Aerial Parts on Viability of Five Human Cancer Cell Lines. *Journal of Agricultural and Food Chemistry* **2017**, *65* (36), 7944-7954.
(211) Zhang, H.; Yang, Y.-f.; Zhou, Z.-q. Phenolic and flavonoid contents of mandarin (Citrus reticulata Blanco) fruit tissues and their antioxidant capacity as evaluated by DPPH and ABTS methods. *Journal of Integrative Agriculture* **2018**, *17* (1), 256-263. DOI: 10.1016/s2095-3119(17)61664-2.
(212) Patel, N. K.; Bhutani, K. K. Pinostrobin and Cajanus lactone isolated from Cajanus cajan (L.) leaves inhibits TNF-α and IL-1β production: in vitro and in vivo experimentation. *Phytomedicine* **2014**, *21* (7), 946-953.
(213) Dongmei, W.; Liang, Z.; Dengwu, L.; Wenjun, P. Antioxidant activities of different extracts and homoisoflavanones isolated from the Polygonatum odoratum. *Natural product research* **2013**, *27* (12), 1111-1114.
(214) Dieudonné, N.; Joseph Tanyi, M.; Zacharias Tanee, F.; Albert, K.; Jean-Claude, M.; M Carmen, R.; Giner, R. M.; Salvador, M. E.; José Luis, R. Anti-inflammatory activities of two flavanones, sigmoidin A and sigmoidin B, from Erythrina sigmoidea. *Planta medica* **2004**, *70* (02), 104-107.
(215) Igal, E.; M Paula, I.; Casanova, M. B.; Celentano, A. M.; Cristina, P.; José Luis, C.; Diez, R. A.; Marta, D. Mitochondrial toxicity and antioxidant activity of a prenylated flavonoid isolated from Dalea elegans. *Chemico-biological interactions* **2008**, *171* (3), 294-305.
(216) Lu, C.-l.; Zhu, W.; Wang, M.; Xu, X.-j.; Lu, C.-j. Antioxidant and Anti-Inflammatory Activities of Phenolic-Enriched Extracts ofSmilax glabra. *Evidence-Based Complementary and Alternative Medicine* **2014**, *2014*, 1-8. DOI: 10.1155/2014/910438.
(217) Valenzuela A; Guerra R; LA., V. Antioxidant properties of the flavonoids silybin and (+)-cyanidanol-3: comparison with butylated hydroxyanisole and butylated hydroxytoluene. *Planta Med* **1986**, *6*, 438-440.
(218) Lucini, L.; Kane, D.; Pellizzoni, M.; Ferrari, A.; Trevisi, E.; Ruzickova, G.; Arslan, D. Phenolic profile and in vitro antioxidant power of different milk thistle [Silybum marianum (L.) Gaertn.] cultivars. *Industrial Crops and Products* **2016**, *83*, 11-16. DOI: 10.1016/j.indcrop.2015.12.023.
(219) Ouyang, H.; Hou, K.; Peng, W.; Liu, Z.; Deng, H. Antioxidant and xanthine oxidase inhibitory activities of total polyphenols from onion. *Saudi Journal of Biological Sciences* **2018**, *25* (7), 1509-1513. DOI: 10.1016/j.sjbs.2017.08.005.
(220) Chang, S. K.; Alasalvar, C.; Shahidi, F. Review of dried fruits: Phytochemicals, antioxidant efficacies, and health benefits. *Journal of Functional Foods* **2016**, *21*, 113-132. DOI: 10.1016/j.jff.2015.11.034.
(221) Li, X.; Liu, J.; Lin, J.; Wang, T.; Huang, J.; Lin, Y.; Chen, D. Protective Effects of Dihydromyricetin against *OH-Induced Mesenchymal Stem Cells Damage and Mechanistic Chemistry. *Molecules* **2016**, *21* (5), 604. DOI: 10.3390/molecules21050604.
(222) Lu, C. L.; Zhu, W.; Wang, M.; Xu, X. J.; Lu, C. J. Antioxidant and Anti-Inflammatory Activities of Phenolic-Enriched Extracts of Smilax glabra. *Evidence-Based Complementary and Alternative Medicine* **2014**.
(223) Liu, J. J.; Li, X. C.; Lin, J.; Li, Y. R.; Wang, T. T.; Jiang, Q.; Chen, D. F. Sarcandra glabra (Caoshanhu) protects mesenchymal stem cells from oxidative stress: a bioevaluation and mechanistic chemistry. *BMC Complement. Altern. Med.* **2016**, *16* (1), 423. DOI: 10.1186/s12906-016-1383-7.

(224) Zhao, Z. W.; Dong, Z. F.; Ming, J.; Liu, Y. Cedrin identified from Cedrus deodara (Roxb.) G. Don protects PC12 cells against neurotoxicity induced by A beta(1-42). *Natural Product Research* **2018**, *32* (12), 1455-1458.
(225) Shao, B.; Guo, H. Z.; Cui, Y. J.; Liu, A. H.; Yu, H. L.; Guo, H.; Xu, M.; Guo, D. A. Simultaneous determination of six major stilbenes and flavonoids in Smilax china by high performance liquid chromatography. *Journal of Pharmaceutical and Biomedical Analysis* **2007**, *44* (3), 737-742.
(226) Chen, S. D.; Lu, C. J.; Zhao, R. Z. Qualitative and Quantitative Analysis of Rhizoma Smilacis glabrae by Ultra High Performance Liquid Chromatography Coupled with LTQ Orbitrap(XL) Hybrid Mass Spectrometry. *Molecules* **2014**, *19* (7), 10427-10439.
(227) Kwon, D.; Yoon, S.; Carter, O.; Bailey, G. S.; Dashwood, R. H. Antioxidant and antigenotoxic activities ofAngelica keiskei, Oenanthe javanicaandBrassica oleraceain the Salmonella mutagenicity assay and in HCT116 human colon cancer cells. *BioFactors* **2006**, *26* (4), 231-244. DOI: 10.1002/biof.5520260402.
(228) Choi, E. J.; Kwon, H. C.; Sohn, Y. C.; Nam, C. W.; Park, H. B.; Kim, C. Y.; Yang, H. O. Four flavonoids from Echinosophora koreensis and their effects on alcohol metabolizing enzymes. *Arch Pharm Res* **2009**, *32* (6), 851-855. DOI: 10.1007/s12272-009-1606-2.
(229) Qian, Z. M.; Lu, J.; Gao, Q. P.; Li, S. P. Rapid method for simultaneous determination of flavonoid, saponins and polyacetylenes in folium ginseng and radix ginseng by pressurized liquid extraction and high-performance liquid chromatography coupled with diode array detection and mass spectrometry. *J Chromatogr A* **2009**, *1216* (18), 3825-3830. DOI: 10.1016/j.chroma.2009.02.065.
(230) Jiang, Q.; Li, X. C.; Tian, Y. G.; Lin, Q. Q.; Xie, H.; Lu, W. B.; Chi, Y. G.; Chen, D. F. Lyophilized aqueous extracts of Mori Fructus and Mori Ramulus protect Mesenchymal stem cells from *OH-treated damage: bioassay and antioxidant mechanism. *BMC Complement. Altern. Med.* **2017**, *16* (1), 423. DOI: 10.1186/s12906-017-1730-3.
(231) He, F. Y.; Li, D. W.; Wang, D. M.; Deng, M. Extraction and Purification of Quercitrin, Hyperoside, Rutin, and Afzelin from Zanthoxylum Bungeanum Maxim Leaves Using an Aqueous Two-Phase System. *J. Food Sci.* **2016**, *81* (7), C1593-C1602.
(232) Li, X. C.; Jiang, Q.; Wang, T. T.; Liu, J. J.; Chen, D. F. Comparison of the Antioxidant Effects of Quercitrin and Isoquercitrin: Understanding the Role of the 6"-OH Group. *Molecules* **2016**, *21* (9), 1246. DOI: doi:10.3390/molecules21091246.
(233) Bezerra, I. C. F.; Ramos, R. T. D.; Ferreira, M. R. A.; Soares, L. A. L. Chromatographic profiles of extractives from leaves of Eugenia uniflora. *Revista Brasileira De Farmacognosia-Brazilian Journal of Pharmacognosy* **2018**, *28* (1), 92-101.
(234) Cichewicz, R. H.; Nair, M. G. Isolation and Characterization of Stelladerol, a New Antioxidant Naphthalene Glycoside, and Other Antioxidant Glycosides from Edible Daylily (Hemerocallis) Flowers. *Journal of Agricultural and Food Chemistry* **2002**, *50* (1), 87-91. DOI: 10.1021/jf010914k.
(235) Granica, S.; Czerwińska, M. E.; Żyżyńska-Granica, B.; Kiss, A. K. Antioxidant and anti-inflammatory flavonol glucuronides from Polygonum aviculare L. *Fitoterapia* **2013**, *91*, 180-188. DOI: 10.1016/j.fitote.2013.08.026.
(236) Qin, Y.; Chen, J.-P.; Li, C.-Y.; Zhu, L.-J.; Zhang, X.; Wang, J.-H.; Yao, X.-S. Flavonoid glycosides from the fruits of Embelia ribes and their anti-oxidant and α-glucosidase inhibitory activities. *Journal of Asian Natural Products Research* **2020**, *23* (8), 724-730. DOI: 10.1080/10286020.2020.1776266.
(237) Ferreres, F.; Pereira, D. M.; Valentão, P. c.; Andrade, P. B.; Seabra, R. M.; Sottomayor, M. New Phenolic Compounds and Antioxidant Potential ofCatharanthus roseus. *Journal of Agricultural and Food Chemistry* **2008**, *56* (21), 9967-9974. DOI: 10.1021/jf8022723.
(238) Nishida, Y.; Wada, K.; Toyohisa, D.; Tanaka, T.; Ono, M.; Yasuda, S. Homoisoflavones as the antioxidants responsible from bulbs of Scilla scilloides. *Nat Prod Res* **2013**, *27* (24), 2360-2362. DOI: 10.1080/14786419.2013.830218.
(239) Tepe, B. In vitro antioxidant activities of the methanol extracts of four Helichrysum species from Turkey. *Food Chemistry* **2005**, *90* (4), 685-689. DOI: 10.1016/j.foodchem.2004.04.030.
(240) Krenn, L.; Presser, A.; Pradhan, R.; Bahr, B.; Paper, D. H.; Mayer, K. K.; Kopp, B. Sulfemodin 8-O-β-D-glucoside, a new sulfated anthraquinone glycoside, and antioxidant phenolic compounds from Rheum emodi. *J Nat Prod* **2003**, *66* (8), 1107-1109. DOI: 10.1021/np0301442.
(241) Chen, H.; Wang, C.; Ye, J.; Zhou, H.; Yuan, J. Phenolic extracts fromRhus verniciflua Stokes bark by decompressing inner ebullition and their antioxidant activities. *Natural Product Research* **2014**, *28* (7), 496-499. DOI: 10.1080/14786419.2013.872105.
(242) Li, X. C.; Xie, H.; Zhan, R. C.; Chen, D. F. Effect of Double Bond Position on 2-Phenyl-benzofuran Antioxidants: A Comparative Study of Moracin C and Iso-Moracin C. *Molecules* **2018**, *23* (4). DOI: ARTN 754
10.3390/molecules23040754.
(243) Chen, Y.; Wei, X. H.; Deng, H. Antioxidant 2-phenylbenzofurans and a coumestan from Lespedeza virgata. *Journal of Natural Products* **2008**, *71* (6), 929.

(244) Li, M.; Zhang, L.; Liu, W.; Lu, W. DFT Study on Molecular Structures and ROS Scavenging Mechanisms of Novel Antioxidants from Lespedeza Virgata. *Chinese Journal of Chemical Physics* **2011**, *24* (2), 173-180.
(245) Qi, J.; Deng, H.; Cao, D. Total synthesis of lespedezavirgatol. *Science China Chemistry* **2010**, *53* (12), 2547-2550.
(246) Ju, H. L.; Ko, H. J.; Woo, E. R.; Sang, K. L.; Moon, B. S.; Chan, W. L.; Mandava, S.; Samala, M.; Lee, J.; Kim, H. P. Moracin M inhibits airway inflammation by interrupting the JNK/c-Jun and NF-κB pathways in vitro and in vivo. *European Journal of Pharmacology* **2016**, *783*, 64-72.
(247) Sun, J.; He, X. M.; Zhao, M. M.; Li, L.; Li, C. B.; Dong, Y. Antioxidant and nitrite-scavenging capacities of phenolic compounds from sugarcane (Saccharum officinarum L.) tops. *Molecules* **2014**, *19* (9), 13147-13160.
(248) Kapche, G. D. W. F.; Fozing, C. D.; Donfack, J. H.; Fotso, G. W.; Amadou, D.; Tchana, A. N.; Bezabih, M.; Moundipa, P. F.; Ngadjui, B. T.; Abegaz, B. M. Prenylated arylbenzofuran derivatives from Morus mesozygia with antioxidant activity. *Phytochemistry* **2009**, *70* (2), 216-221.
(249) Seong, S. H.; Ha, M. T.; Min, B. S.; Jung, H. A.; Choi, J. S. Moracin derivatives from Morus Radix as dual BACE1 and cholinesterase inhibitors with antioxidant and anti-glycation capacities. *Life Sciences* **2018**.
(250) Hwang, S. M.; Lee, H. J.; Jung, J. H.; Sim, D. Y.; Hwang, J.; Park, J. E.; Shim, B. S.; Kim, S. H. Inhibition of Wnt3a/FOXM1/β-Catenin Axis and Activation of GSK3beta and Caspases are Critically Involved in Apoptotic Effect of Moracin D in Breast Cancers. *Int J Mol Sci* **2018**, *19* (9). DOI: 10.3390/ijms19092681 From NLM.
(251) Han, S. J.; Ryu, S. N.; Kang, S. S. A new 2-arylbenzofuran with antioxidant activity from the black colored rice (Oryza sativa L.) bran. *Cheminform* **2005**, *36* (18), 1365.
(252) Kapche, G. D.; Amadou, D.; Waffo-Teguo, P.; Donfack, J. H.; Fozing, C. D.; Harakat, D.; Tchana, A. N.; Mérillon, J. M.; Moundipa, P. F.; Ngadjui, B. T. Hepatoprotective and antioxidant arylbenzofurans and flavonoids from the twigs of Morus mesozygia. *Planta medica* **2011**, *77* (10), 1044-1047.
(253) Kyekyeku, J. O.; Kusari, S.; Adosraku, R. K.; Zühlke, S.; Spiteller, M. Prenylated 2-arylbenzofuran derivatives with potent antioxidant properties from Chlorophora regia (Moraceae). *Fitoterapia* **2016**, *108* (1), 41-47.
(254) Meneses, M. A.; Caputo, G.; Scognamiglio, M.; Reverchon, E.; Adami, R. Antioxidant phenolic compounds recovery from Mangifera indica L. by-products by supercritical antisolvent extraction. *Journal of Food Engineering* **2015**, *163*, 45-53. DOI: 10.1016/j.jfoodeng.2015.04.025.
(255) Olennikov, D. N.; Chirikova, N. K.; Vennos, C. Chemical Composition, Antioxidant and Anticholinesterase Activities of Gentianella azurea from Russian Federation. *Nat. Prod. Commun.* **2017**, *12* (1), 55-56.
(256) Singh, P. P.; Ambika; Chauhan, S. M. S. Activity-guided isolation of antioxidant xanthones from Swertia chirayita (Roxb.) H. Karsten (Gentianaceae). *Natural product research* **2012**, *26* (18), 1682-1686. DOI: 10.1080/14786419.2011.592836.
(257) Urbain, A.; Marston, A.; Grilo, L. S.; Bravo, J.; Purev, O.; Purevsuren, B.; Batsuren, D.; Reist, M.; Carrupt, P. A.; Hostettmann, K. Xanthones from Gentianella amarella ssp. acuta with acetylcholinesterase and monoamine oxidase inhibitory activities. *J Nat Prod* **2008**, *71* (5), 895-897. DOI: 10.1021/np070690l.
(258) Li, X.; Chen, B.; Zhao, X.; Chen, D. 2-Phenyl-4,4,5,5-tetramethylimidazoline-1-oxyl 3-oxide Radical (PTIO center dot) Trapping Activity and Mechanisms of 16 Phenolic Xanthones. *Molecules* **2018**, *23* (7). DOI: 10.3390/molecules23071692.
(259) Matkowski, A.; Kus, P.; Goralska, E.; Wozniak, D. Mangiferin - a Bioactive Xanthonoid, not only from Mango and not just Antioxidant. *Mini-Reviews in Medicinal Chemistry* **2013**, *13* (3), 439-455, Review.
(260) Li, X.; Jiang, Q.; Chen, B.; Luo, X.; Chen, D. Structure-Activity Relationship and Prediction of the Electron-Transfer Potential of the Xanthones Series. *Chemistryopen* **2018**, *7* (9), 730-736. DOI: 10.1002/open.201800108.
(261) Zheng, H.-H.; Luo, C.-T.; Chen, H.; Lin, J.-N.; Ye, C.-L.; Mao, S.-S.; Li, Y.-L. Xanthones from Swertia mussotii as Multitarget-Directed Antidiabetic Agents. *Chemmedchem* **2014**, *9* (7), 1374-1377. DOI: 10.1002/cmdc.201300507.
(262) Xia, Y.; Liu, X.; Zou, C.; Feng, S.; Guo, H.; Yang, Y.; Lei, Y.; Zhang, J.; Lu, Y. Garcinone C exerts antitumor activity by modulating the expression of ATR/Stat3/4E-BP1 in nasopharyngeal carcinoma cells. *Oncology reports* **2018**, *39* (3), 1485-1493. DOI: 10.3892/or.2018.6218.
(263) Wang, S. N.; Li, Q.; Jing, M. H.; Alba, E.; Yang, X. H.; Sabate, R.; Han, Y. F.; Pi, R. B.; Lan, W. J.; Yang, X. B.; et al. Natural Xanthones from Garcinia mangostana with Multifunctional Activities for the Therapy of Alzheimer's Disease. *Neurochem. Res.* **2016**, *41* (7), 1806-1817. DOI: 10.1007/s11064-016-1896-y.
(264) Gu, C.; Yang, M.; Zhou, Z.; Khan, A.; Cao, J.; Cheng, G. Purification and characterization of four benzophenone derivatives from Mangifera indica L. leaves and their antioxidant, immunosuppressive and alpha-glucosidase inhibitory activities. *Journal of Functional Foods* **2019**, *52*, 709-714. DOI: 10.1016/j.jff.2018.11.045.

(265) Hou, C. Theoretical study of antioxidative ability and antioxidative mechanism of norathyriol in solution. *Computational and Theoretical Chemistry* **2014**, *1028*, 87-91. DOI: 10.1016/j.comptc.2013.11.023.
(266) Gao, X.-y.; Wang, S.-n.; Yang, X.-h.; Lan, W.-j.; Chen, Z.-w.; Chen, J.-k.; Xie, J.-h.; Han, Y.-f.; Pi, R.-b.; Yang, X.-b. Gartanin Protects Neurons against Glutamate-Induced Cell Death in HT22 Cells: Independence of Nrf-2 but Involvement of HO-1 and AMPK. *Neurochemical Research* **2016**, *41* (9), 2267-2277. DOI: 10.1007/s11064-016-1941-x.
(267) Luo, M.; Liu, Q.; He, M.; Yu, Z.; Pi, R.; Li, M.; Yang, X.; Wang, S.; Liu, A. Gartanin induces cell cycle arrest and autophagy and suppresses migration involving PI3K/Akt/mTOR and MAPK signalling pathway in human glioma cells. *Journal of Cellular and Molecular Medicine* **2017**, *21* (1), 46-57. DOI: 10.1111/jcmm.12937.
(268) Kim, M.-O.; Lee, H.-S.; Chin, Y.-W.; Moon, D.-O.; Ahn, J.-S. Gartanin induces autophagy through JNK activation which extenuates caspase-dependent apoptosis. *Oncology Reports* **2015**, *34* (1), 139-146. DOI: 10.3892/or.2015.3948.
(269) Wang, S.-n.; Li, Q.; Jing, M.-h.; Alba, E.; Yang, X.-h.; Sabate, R.; Han, Y.-f.; Pi, R.-b.; Lan, W.-j.; Yang, X.-b.; et al. Natural Xanthones from Garcinia mangostana with Multifunctional Activities for the Therapy of Alzheimer's Disease. *Neurochemical Research* **2016**, *41* (7), 1806-1817. DOI: 10.1007/s11064-016-1896-y.
(270) Blanco-Ayala, T.; Lugo-Huitron, R.; Serrano-Lopez, E. M.; Reyes-Chilpa, R.; Rangel-Lopez, E.; Pineda, B.; Noel Medina-Campos, O.; Sanchez-Chapul, L.; Pinzon, E.; Cristina, T.-S.; et al. Antioxidant properties of xanthones from Calophyllum brasiliense: prevention of oxidative damage induced by FeSO4. *Bmc Complementary and Alternative Medicine* **2013**, *13*. DOI: 10.1186/1472-6882-13-262.
(271) Chen, L.; Zhou, Q.; Li, B.; Liu, S.-J.; Dong, J.-X. A new flavonoid from Cudrania cochinchinensis. *Natural product research* **2015**, *29* (13), 1217-1221. DOI: 10.1080/14786419.2014.997234.
(272) Li, W.; Sun, Y. N.; Yan, X. T.; Yang, S. Y.; Choi, C. W.; Hyun, J. W.; Kang, H. K.; Paek, K. Y.; Kim, Y. H. Isolation of xanthones from adventitious roots of St. John's Wort (Hypericum perforatum L.) and their antioxidant and cytotoxic activities. *Food Science and Biotechnology* **2013**, *22* (4), 945-949. DOI: 10.1007/s10068-013-0168-8.
(273) Minami, H.; Kinoshita, M.; Fukuyama, Y.; Kodama, M.; Yoshizawa, T.; Sugiura, M.; Nakagawa, K.; Tago, H. Antioxidant Xanthones from Garcinia-Subelliptica. *Phytochemistry* **1994**, *36* (2), 501-506. DOI: 10.1016/s0031-9422(00)97103-6.
(274) Wang, L.-L.; Li, Z.-L.; Hua, H.-M.; Liu, X.-Q. Chemical constituents from barks of Garcinia tetralata. *Zhongguo Zhong yao za zhi = Zhongguo zhongyao zazhi = China journal of Chinese materia medica* **2008**, *33* (20), 2350-2352.
(275) Iinuma, M.; Tosa, H.; Tanaka, T.; Asai, F.; Shimano, R. Xanthones from Root Bark Of Garcinia-Subelliptica.*Phytochemistry* **1995**, *38* (1), 247-249. DOI: 10.1016/0031-9422(94)00595-k.
(276) Chukaew, A.; Saithong, S.; Chusri, S.; Limsuwan, S.; Watanapokasin, R.; Voravuthikunchai, S. P.; Chakthong, S. Cytotoxic xanthones from the roots of Mesua ferrea L. *Phytochemistry* **2019**, *157*, 64-70. DOI: 10.1016/j.phytochem.2018.10.008.
(277) Zuo, G.-Y.; An, J.; Han, J.; Zhang, Y.-L.; Wang, G.-C.; Hao, X.-Y.; Bian, Z.-Q. Isojacareubin from the Chinese Herb Hypericum japonicum: Potent Antibacterial and Synergistic Effects on Clinical Methicillin-Resistant Staphylococcus aureus (MRSA). *International Journal of Molecular Sciences* **2012**, *13* (7), 8210-8218. DOI: 10.3390/ijms13078210.
(278) Lee, E. J. fun609α, g-Mangostins Induce Autophagy and Show Synergistic Effect with Gemcitabine in Pancreatic Cancer Cell Lines. *Biomolecules & Therapeutics* **2017**, *25* (6), 609-617.
(279) Stark, T. D.; Salger, M.; Frank, O.; Balemba, O. B.; Wakamatsu, J.; Hofmann, T. Antioxidative Compounds from Garcinia buchananii Stem Bark. *Journal of Natural Products* **2015**, *78* (2), 234-240. DOI: 10.1021/np5007873.
(280) Yuan, H.; Jiang, C.; Zhao, J.; Zhao, Y.; Zhang, Y.; Xu, Y.; Gao, X.; Guo, L.; Liu, Y.; Liu, K.; et al. Euxanthone Attenuates A(1-42)-Induced Oxidative Stress and Apoptosis by Triggering Autophagy. *Journal of Molecular Neuroscience* **2018**, *66* (4), 512-523. DOI: 10.1007/s12031-018-1175-2.
(281) Li, Y.-z.; Li, Z.-l.; Hua, H.-m.; Li, Z.-g.; Liu, M.-s. Studies on flavonoids from stems and leaves of Calophyllum inophyllum. *Zhongguo Zhong yao za zhi = Zhongguo zhongyao zazhi = China journal of Chinese materia medica* **2007**, *32* (8), 692-694.
(282) Lannang, A. M.; Komguem, J.; Ngninzeko, F. N.; Tangmouo, J. G.; Lontsi, D.; Ajaz, A.; Choudhary, M. I.; Ranjit, R.; Devkota, K. P.; Sondengam, B. L. Bangangxanthone A and B, two xanthones from the stem bark of Garcinia polyantha Oliv. *Phytochemistry* **2005**, *66* (19), 2351-2355. DOI: 10.1016/j.phytochem.2005.06.016.
(283) Buelna-Chontal, M.; Correa, F.; Hernandez-Resendiz, S.; Zazueta, C.; Pedraza-Chaverri, J. Protective Effect of alpha-Mangostin on Cardiac Reperfusion Damage by Attenuation of Oxidative Stress. *Journal of medicinal food* **2011**, *14* (11), 1370-1374. DOI: 10.1089/jmf.2010.0238.
(284) Trisuwan, K.; Ritthiwigrom, T. Benzophenone and xanthone derivatives from the inflorescences of Garcinia cowa. *Archives of Pharmacal Research* **2012**, *35* (10), 1733-1738. DOI: 10.1007/s12272-012-1004-z.

(285) Panthong, K.; Hutadilok-Towatana, N.; Panthong, A. Cowaxanthone F, a new tetraoxygenated xanthone, and other anti-inflammatory and antioxidant compounds from Garcinia cowa. *Canadian Journal of Chemistry-Revue Canadienne De Chimie* **2009**, *87* (11), 1636-1640. DOI: 10.1139/v09-123.
(286) Panthong, K.; Pongcharoen, W.; Phongpaichit, S.; Taylor, W. C. Tetraoxygenated xanthones from the fruits of Garcinia cowa. *Phytochemistry* **2006**, *67* (10), 999-1004. DOI: 10.1016/j.phytochem.2006.02.027.
(287) Ponguschariyagul, S.; Sichaem, J.; Khumkratok, S.; Siripong, P.; Lugsanangarm, K.; Tip-pyang, S. Caloinophyllin A, a new chromanone derivative from Calophyllum inophyllum roots. *Natural product research* **2018**, *32* (21), 2535-2541. DOI: 10.1080/14786419.2018.1425845.
(288) Jung, H. A.; Su, B. N.; Keller, W. J.; Mehta, R. G.; Kinghorn, A. D. Antioxidant xanthones from the pericarp of Garcinia mangostana (mangosteen). *Journal of Agricultural and Food Chemistry* **2006**, *54* (6), 2077-2082. DOI: 10.1021/jf052649z.
(289) Li, J.; Chen, L.; Wu, H.; Lu, Y.; Hu, Z.; Lu, B.; Zhang, L.; Chai, Y.; Zhang, J. The Mixture of Salvianolic Acids from Salvia miltiorrhiza and Total Flavonoids from Anemarrhena asphodeloides Attenuate Sulfur Mustard-Induced Injury. *International Journal of Molecular Sciences* **2015**, *16* (10), 24555-24573. DOI: 10.3390/ijms161024555.
(290) Ji, X.; Avula, B.; Khan, I. A. Quantitative and qualitative determination of six xanthones in Garcinia mangostana L. by LC-PDA and LC-ESI-MS. *Journal of Pharmaceutical and Biomedical Analysis* **2007**, *43* (4), 1270-1276. DOI: 10.1016/j.jpba.2006.10.018.
(291) Suvarnakuta, P.; Chaweerungrat, C.; Devahastin, S. Effects of drying methods on assay and antioxidant activity of xanthones in mangosteen rind. *Food Chemistry* **2011**, *125* (1), 240-247. DOI: 10.1016/j.foodchem.2010.09.015.
(292) Li, J.; Li, W. Z. M.; Huang, W.; Cheung, A. W. H.; Bi, C. W. C.; Duan, R.; Guo, A. J. Y.; Dong, T. T. X.; Tsim, K. W. K. Quality evaluation of Rhizoma Belamcandae (Belamcanda chinensis (L.) DC.) by using high-performance liquid chromatography coupled with diode array detector and mass spectrometry. *Journal of Chromatography A* **2009**, *1216* (11), 2071-2078. DOI: 10.1016/j.chroma.2008.05.082.
(293) Yu, H. Y.; Jin, S. L.; Zhang, X.; Liu, Y.; Ou, Y. F.; Wang, N. L.; Yao, X. S. Two new benzophenone glucosides from Cratoxylon cochinchinensis. *Chinese Chemical Letters* **2009**, *20* (4), 459-461. DOI: 10.1016/j.cclet.2008.12.038.
(294) Taher, M.; Susanti, D.; Rezali, M. F.; Zohri, F. S. A.; Ichwan, S. J. A.; Alkhamaiseh, S. I.; Ahmad, F. Apoptosis, antimicrobial and antioxidant activities phytochemicals from Garcinia malaccensis Hk.f. *Asian Pacific Journal of Tropical Medicine* **2012**, *5* (2), 136-141. DOI: 10.1016/s1995-7645(12)60012-1.
(295) Chang, C. H.; Lin, C. C.; Hattori, M.; Namba, T. Effects on Anti-Lipid Peroxidation of Cudrania-Cochinchinensis Var Gerontogea. *Journal of Ethnopharmacology* **1994**, *44* (2), 79-85.
(296) Zhou, Y.-H.; Zhang, S.-Y.; Guo, Q.; Chai, X.-Y.; Jiang, Y.; Tu, P.-F. Chemical investigation of the roots of Polygala sibirica L. *Chinese journal of natural medicines* **2014**, *12* (3), 225-228. DOI: 10.3724/sp.j.1009.2014.00225.
(297) Wang, J.; Jiang, Z.; Wei, G.; Wang, Y.; Liu, Y. Protective Effects of Chemical Constituents from the Roots of Polygala sibirica L. on PC12 Cells Damage Induced by H2O2. *Latin American Journal of Pharmacy* **2014**, *33* (7), 1120-1124.
(298) Yin, C.; Xie, L.; Guo, Y. Phytochemical analysis and antibacterial activity of Gentiana macrophylla extract against bacteria isolated from burn wound infections. *Microbial Pathogenesis* **2018**, *114*, 25-28. DOI: 10.1016/j.micpath.2017.10.049.
(299) Wani, B. A.; Ramamoorthy, D.; Rather, M. A.; Arumugam, N.; Qazi, A. K.; Majeed, R.; Hamid, A.; Ganie, S. A.; Ganai, B. A.; Anand, R.; et al. Induction of apoptosis in human pancreatic MiaPaCa-2 cells through the loss of mitochondrial membrane potential (Delta psi(m)) by Gentiana kurroo root extract and LC-ESI-MS analysis of its principal constituents. *Phytomedicine* **2013**, *20* (8-9), 723-733. DOI: 10.1016/j.phymed.2013.01.011.
(300) Lee, B. W.; Lee, J. H.; Lee, S. T.; Lee, H. S.; Lee, W. S.; Jeong, T. S.; Park, K. H. Antioxidant and cytotoxic activities of xanthones from Cudrania tricuspidata. *Bioorganic & Medicinal Chemistry Letters* **2005**, *15* (24), 5548-5552. DOI: 10.1016/j.bmcl.2005.08.099.
(301) Lin, L.-L.; Huang, F.; Chen, S.-B.; Yang, D.-J.; Chen, S.-L.; Yang, J.-S.; Xiao, P.-G. Chemical constituents in roots of Polygala fallax and their anti-oxidation activities in vitro. *Zhongguo Zhong yao za zhi = Zhongguo zhongyao zazhi = China journal of Chinese materia medica* **2005**, *30* (11), 827-830.
(302) Wu, J.-f.; Tu, P.-f.; Zhan, H.-t.; Gao, J.-c. Dioxyxanthones from Polygala hongkongensis and Their Cytotoxicity. *Chemical Research in Chinese Universities* **2011**, *27* (5), 777-779.
(303) Ashida, S.; Noguchi, S. F.; Suzuki, T. Antioxidative Components, Xanthone Derivatives in Swertia-Japonica Makino. *Journal of the American Oil Chemists Society* **1994**, *71* (10), 1095-1099. DOI: 10.1007/bf02675902.
(304) Tian, L. Y.; Bai, X.; Chen, X. H.; Fang, J. B.; Liu, S. H.; Chen, J. C. Anti-diabetic effect of methylswertianin and bellidifolin from Swertia punicea Hemsl. and its potential mechanism. *Phytomedicine* **2010**, *17* (7), 533-539. DOI: 10.1016/j.phymed.2009.10.007.

(305) Mahendran, G.; Bai, V. N. Plant regeneration through direct somatic embryogenesis, antioxidant properties, and metabolite profiles of Swertia corymbosa (Griseb.) Wight ex CB Clarke. *Plant Biosystems* **2017**, *151* (1), 39-49. DOI: 10.1080/11263504.2015.1064043.
(306) Li, H.; Lu, Q.; Bian, K. Ultrasonic-Assisted Extraction and Evaluation of Biological Activities of Flavonoids from Flemingia philippinensis Merr et Rolfe. *Tropical Journal of Pharmaceutical Research* **2015**, *14* (8), 1365-U1213. DOI: 10.4314/tjpr.v14i8.7.
(307) Shi, Q.; Chen, J.; Zhou, Q.; Lei, H.; Luan, L.; Liu, X.; Wu, Y. Indirect identification of antioxidants in Polygalae Radix through their reaction with 2,2-dipheny1-1-picrylhydrazyl and subsequent HPLC-ESI-Q-TOF-MS/MS. *Talanta* **2015**, *144*, 830-835. DOI: 10.1016/j.talanta.2015.07.032.
(308) Xu, R.; Mao, F.; Zhao, Y.; Wang, W.; Fan, L.; Gao, X.; Zhao, J.; Tian, H. UPLC Quantitative Analysis of Multi-Components by Single Marker and Quality Evaluation of Polygala tenuifolia Wild. Extracts. *Molecules* **2017**, *22* (12). DOI: 10.3390/molecules22122276.
(309) Azad, F. R.; Shafaghat, A.; Salimi, F. Chemical Constituents, Antioxidant and Antimicrobial Activities of Essential Oil and Hexane Extract from Arnebia linearifolia DC. Seeds. *Journal of Essential Oil Bearing Plants* **2014**, *17* (1), 87-94. DOI: 10.1080/0972060x.2014.884813.
(310) Mohamed, T. K. Chemical constituents and antioxidant activity of Citrus paradisi (star-ruby red grapefruit) and Citrus sinensis (blood sweet orange) Egyptian cultivars. *Asian Journal of Chemistry* **2004**, *16* (3-4), 1753-1764.
(311) Marques, E. d. S.; Salles, D. B.; Maistro, E. L. Assessment of the genotoxic/clastogenic potential of coumarin derivative 6,7-dihydroxycoumarin (aesculetin) in multiple mouse organs. *Toxicology reports* **2015**, *2*, 268-274. DOI: 10.1016/j.toxrep.2015.01.005.
(312) Wang, K.; Zhang, Y.; Ekunwe, S. I. N.; Yi, X.; Liu, X.; Wang, H.; Pan, Y. Antioxidant activity and inhibition effect on the growth of human colon carcinoma (HT-29) cells of esculetin from Cortex Fraxini. *Medicinal Chemistry Research* **2011**, *20* (7), 968-974. DOI: 10.1007/s00044-010-9426-y.
(313) Tang, Y. P.; Hu, J.; Wang, J. H.; Lou, F. C. A new coumaronochromone from Sophora japonica. *Journal of Asian Natural Products Research* **2002**, *4* (1), 1-5. DOI: 10.1080/10286020290019622.
(314) Wadhwa, D.; Arora, V.; Arora, L.; Arora, P.; Parkash, O. Synthesis of Naphthalene Functionalized Trans-2,3-dihydrofuro 3,2-c coumarins as Antioxidant and Anthelmintic Agents. *Journal of Heterocyclic Chemistry* **2016**, *53* (4), 1030-1035. DOI: 10.1002/jhet.2431.
(315) Dehghan, H.; Sarrafi, Y.; Salehi, P.; Ebrahimi, S. N. alpha-Glucosidase inhibitory and antioxidant activity of furanocoumarins from Heracleum persicum. *Medicinal Chemistry Research* **2017**, *26* (4), 849-855. DOI: 10.1007/s00044-017-1796-y.
(316) Li, D.; Wu, L. Coumarins from the roots of Angelica dahurica cause anti-allergic inflammation. *Experimental and Therapeutic Medicine* **2017**, *14* (1), 874-880. DOI: 10.3892/etm.2017.4569.
(317) Kumar, V.; Sharma, K.; Ahmed, B.; Al-Abbasi, F. A.; Anwar, F.; Verma, A. Deconvoluting the dual hypoglycemic effect of wedelolactone isolated from Wedelia calendulacea: investigation via experimental validation and molecular docking. *Rsc Advances* **2018**, *8* (32), 18180-18196. DOI: 10.1039/c7ra12568b.
(318) Nie, Y.; Yao, W. A Comprehensive Quality Evaluation Method Based on C-30-HPLC and an Analytic Hierarchy Process for the Chinese Herbal Formula, Erzhiwan. *Molecules* **2018**, *23* (8). DOI: 10.3390/molecules23082045.
(319) Ding, H.; Wang, Y.; Gao, Y.; Han, X.; Liu, S.; Tang, G.; Li, J.; Zhao, D. Purification of wedelolactone from Eclipta alba and evaluation of antioxidant activity. *Separation Science and Technology* **2017**, *52* (17), 2732-2741. DOI: 10.1080/01496395.2017.1374973.
(320) Han, L.; Sun, C.; Lin, J.; Li, F.; Li, X.; Wu, Z.; Liu, C.; Bai, Y.; Mai, W.; Chen, D. Herba Ecliptae Protects against Hydroxyl Radical-induced Damages to DNA and Mesenchymal Stem Cells via Antioxidant Mechanism. *Journal of the Chinese Chemical Society* **2014**, *61* (10), 1161-1167. DOI: 10.1002/jccs.201400082.
(321) Kadhum, A. A. H.; Al-Amiery, A. A.; Musa, A. Y.; Mohamad, A. B. The Antioxidant Activity of New Coumarin Derivatives. *International Journal of Molecular Sciences* **2011**, *12* (9), 5747-5761. DOI: 10.3390/ijms12095747.
(322) Oliva, A.; Meepagala, K. M.; Wedge, D. E.; Harries, D.; Hale, A. L.; Aliotta, G.; Duke, S. O. Natural fungicides from Ruta graveolens L. leaves, including a new quinolone alkaloid. *Journal of Agricultural and Food Chemistry* **2003**, *51* (4), 890-896. DOI: 10.1021/jf0259361.
(323) Hisamoto, M.; Kikuzaki, H.; Ohigashi, H.; Nakatani, N. Antioxidant compounds from the leaves of Peucedanum japonicum Thunb. *Journal of Agricultural and Food Chemistry* **2003**, *51* (18), 5255-5261. DOI: 10.1021/jf0262458.
(324) Yang, Y.-F.; Xu, W.; Song, W.; Ye, M.; Yang, X.-W. Transport of Twelve Coumarins from Angelicae Pubescentis Radix across a MDCK-pHaMDR Cell Monolayer-An in Vitro Model for Blood-Brain Barrier Permeability. *Molecules* **2015**, *20* (7), 11719-11732. DOI: 10.3390/molecules200711719.

(325) Sourivong, P.; Schronerova, K.; Babincova, M. Scoparone inhibits ultraviolet radiation-induced lipid peroxidation. *Zeitschrift Fur Naturforschung Section C-a Journal of Biosciences* **2007**, *62* (1-2), 61-64.
(326) Doppalapudi, S.; Mahira, S.; Khan, W. Development and in vitro assessment of psoralen and resveratrol co-loaded ultradeformable liposomes for the treatment of vitiligo. *Journal of Photochemistry and Photobiology B-Biology* **2017**, *174*, 44-57. DOI: 10.1016/j.jphotobiol.2017.07.007.
(327) Jan, S.; Parween, T.; Siddiqi, T. O.; Mahmooduzzafar. Anti-oxidant modulation in response to gamma radiation induced oxidative stress in developing seedlings of Psoralea corylifolia L. *Journal of Environmental Radioactivity* **2012**, *113*, 142-149. DOI: 10.1016/j.jenvrad.2012.05.019.
(328) Guo, J. M.; Weng, X. C.; Wu, H.; Li, Q. H.; Bi, K. S. Antioxidants from a Chinese medicinal herb - Psoralea corylifolia L. *Food Chemistry* **2005**, *91* (2), 287-292. DOI: 10.1016/j.foodchem.2004.04.029.
(329) Chen, C.-H.; Hwang, T.-L.; Chen, L.-C.; Chang, T.-H.; Wei, C.-S.; Chen, J.-J. Isoflavones and anti-inflammatory constituents from the fruits of Psoralea corylifolia. *Phytochemistry* **2017**, *143*, 186-193. DOI: 10.1016/j.phytochem.2017.08.004.
(330) Orhan, I. E.; Tosun, F.; Skalicka-Wozniak, K. Cholinesterase and tyrosinase inhibitory, and antioxidant potential of randomly selected Umbelliferous plant species and the chromatographic profile of Heracleum platytaenium Boiss. and Angelica sylvestris L. var. sylvestris. *Journal of the Serbian Chemical Society* **2016**, *81* (4), 357-368. DOI: 10.2298/jsc150902017o.
(331) Bai, Y.; Li, D.; Zhou, T.; Qin, N.; Li, Z.; Yu, Z.; Hua, H. Coumarins from the roots of Angelica dahurica with antioxidant and antiproliferative activities. *Journal of Functional Foods* **2016**, *20*, 453-462. DOI: 10.1016/j.jff.2015.11.018.
(332) Wang, Z.; Zhu, W.; Liu, H.; Wu, G.; Song, M.; Yang, B.; Yang, D.; Wang, Q.; Kuang, H. Simultaneous Determination of Aesculin, Aesculetin, Fraxetin, Fraxin and Polydatin in Beagle Dog Plasma by UPLC-ESI-MS/MS and Its Application in a Pharmacokinetic Study after Oral Administration Extracts of Ledum palustre L. *Molecules* **2018**, *23* (9). DOI: 10.3390/molecules23092285.
(333) Yao, Y.; Zhao, X.; Xin, J.; Wu, Y.; Li, H. Coumarins improved type 2 diabetes induced by high-fat diet and streptozotocin in mice via antioxidation. *Canadian Journal of Physiology and Pharmacology* **2018**, *96* (8), 765-771. DOI: 10.1139/cjpp-2017-0612.
(334) Naseri, M.; Monsef-Esfehani, H. R.; Saeidnia, S.; Dastan, D.; Gohari, A. R. Antioxidative Coumarins from the Roots of Ferulago subvelutina. *Asian Journal of Chemistry* **2013**, *25* (4), 1875-1878. DOI: 10.14233/ajchem.2013.13208.
(335) Wu, C.-R.; Huang, M.-Y.; Lin, Y.-T.; Ju, H.-Y.; Ching, H. Antioxidant properties of Cortex Fraxini and its simple coumarins. *Food Chemistry* **2007**, *104* (4), 1464-1471. DOI: 10.1016/j.foodchem.2007.02.023.
(336) Chen, Y.; Zhang, Q.; Liu, H.; Lu, C.; Liang, C.-L.; Qiu, F.; Han, L.; Dai, Z. Esculetin Ameliorates Psoriasis-Like Skin Disease in Mice by Inducing CD4(+) Foxp3(+) Regulatory T Cells. *Frontiers in Immunology* **2018**, *9*. DOI: 10.3389/fimmu.2018.02092.
(337) He, Y.; Li, C.; Ma, Q.; Chen, S. Esculetin inhibits oxidative stress and apoptosis in H9c2 cardiomyocytes following hypoxiaireoxygenation injury. *Biochemical and Biophysical Research Communications* **2018**, *501* (1), 139-144. DOI: 10.1016/j.bbrc.2018.04.195.
(338) Kim, N. M.; Kim, J.; Chung, H. Y.; Choi, J. S. Isolation of luteolin 7-O-rutinoside and esculetin with potential antioxidant activity from the aerial parts of Artemisia montana. *Archives of Pharmacal Research* **2000**, *23* (3), 237-239. DOI: 10.1007/bf02976451.
(339) Pokharel, Y. R.; Jeong, J.-E.; Oh, S. J.; Kim, S. K.; Woo, E.-R.; Kang, K. W. Screening of potential chemopreventive compounds from Poncirus trifoliata Raf. *Pharmazie* **2006**, *61* (9), 796-798.
(340) Al-Rifai, A. Identification and evaluation of in-vitro antioxidant phenolic compounds from the Calendula tripterocarpa Rupr. *South African Journal of Botany* **2018**, *116*, 238-244. DOI: 10.1016/j.sajb.2018.04.007.
(341) Osman, W. N. W.; Mohamed, S. Standardized Morinda citrifolia L. and Morinda elliptica L. leaf extracts alleviated fatigue by improving glycogen storage and lipid/carbohydrate metabolism. *Phytotherapy Research* **2018**, *32* (10), 2078-2085. DOI: 10.1002/ptr.6151.
(342) Shaw, C. Y.; Chen, C. H.; Hsu, C. C.; Chen, C. C.; Tsai, Y. C. Antioxidant properties of scopoletin isolated from Sinomonium acutum. *Phytotherapy Research* **2003**, *17* (7), 823-825. DOI: 10.1002/ptr.1170.
(343) Dakah, A.; Zaid, S.; Suleiman, M.; Abbas, S.; Wink, M. In vitro propagation of the medicinal plant Ziziphora tenuior L. and evaluation of its antioxidant activity. *Saudi Journal of Biological Sciences* **2014**, *21* (4), 317-323. DOI: 10.1016/j.sjbs.2013.12.002.

(344) Jung, H. A.; Park, J. J.; Islam, M. N.; Jin, S. E.; Min, B.-S.; Lee, J.-H.; Sohn, H. S.; Choi, J. S. Inhibitory Activity of Coumarins from Artemisia capillaris against Advanced Glycation Endproduct Formation. *Archives of Pharmacal Research* **2012**, *35* (6), 1021-1035. DOI: 10.1007/s12272-012-0610-0.
(345) Kang, J. I.; Hong, J.-Y.; Choi, J. S.; Lee, S. K. Columbianadin Inhibits Cell Proliferation by Inducing Apoptosis and Necroptosis in HCT116 Colon Cancer Cells. *Biomolecules & Therapeutics* **2016**, *24* (3), 320-327. DOI: 10.4062/biomolther.2015.145.
(346) Ng, T. B.; Liu, F.; Wang, Z. T. Antioxidative activity of natural products from plants. *Life Sciences* **2000**, *66* (8), 709-723. DOI: 10.1016/s0024-3205(99)00642-6.
(347) Yang, Y.-F.; Zhang, L.; Yang, X.-W. Distribution Assessments of Coumarins from Angelicae Pubescentis Radix in Rat Cerebrospinal Fluid and Brain by Liquid Chromatography Tandem Mass Spectrometry Analysis. *Molecules (Basel, Switzerland)* **2018**, *23* (1). DOI: 10.3390/molecules23010225.
(348) Girennavar, B.; Poulose, S. M.; Jayaprakasha, G. K.; Bhat, N. G.; Patil, B. S. Furocoumarins from grapefruit juice and their effect on human CYP 3A4 and CYP1B1 isoenzymes. *Bioorganic & Medicinal Chemistry* **2006**, *14* (8), 2606-2612. DOI: 10.1016/j.bmc.2005.11.039.
(349) Miyake, Y.; Murakami, A.; Sugiyama, Y.; Isobe, M.; Koskimizu, K.; Ohigashi, H. Identification of coumarins from lemon fruit (Citrus limon) as inhibitors of in vitro tumor promotion and superoxide and nitric oxide generation. *Journal of Agricultural and Food Chemistry* **1999**, *47* (8), 3151-3157. DOI: 10.1021/jf980999y.
(350) Okuyama, S.; Nakashima, T.; Nakamura, K.; Shinoka, W.; Kotani, M.; Sawamoto, A.; Nakajima, M.; Furukawa, Y. Inhibitory Effects of Auraptene and Naringin on Astroglial Activation, Tau Hyperphosphorylation, and Suppression of Neurogenesis in the Hippocampus of Streptozotocin-Induced Hyperglycemic Mice. *Antioxidants* **2018**, *7* (8). DOI: 10.3390/antiox7080109.
(351) Soltani, F.; Mosaffa, F.; Iranshahi, M.; Karimi, G.; Malekaneh, M.; Haghighi, F.; Behravan, J. Auraptene from Ferula szowitsiana Protects Human Peripheral Lymphocytes Against Oxidative Stress. *Phytotherapy Research* **2010**, *24* (1), 85-89. DOI: 10.1002/ptr.2874.
(352) Karakaya, S.; Koca, M.; Kilic, C. S.; Coskun, M. Antioxidant and anticholinesterase activities of Ferulago syriaca Boiss. and F-isaurica Pesmen growing in Turkey. *Medicinal Chemistry Research* **2018**, *27* (7), 1843-1850. DOI: 10.1007/s00044-018-2196-7.
(353) Liang, W.-H.; Chang, T.-W.; Charng, Y.-C. Effects of drying methods on contents of bioactive compounds and antioxidant activities of Angelica dahurica. *Food Science and Biotechnology* **2018**, *27* (4), 1085-1092. DOI: 10.1007/s10068-018-0359-4.
(354) Biljali, S.; Hadjimitova, V. A.; Topashka-Ancheva, M. N.; Momekova, D. B.; Traykov, T. T.; Karaivanova, M. H. Antioxidant and antiradical properties of esculin, and its effect in a model of epirubicin-induced bone marrow toxicity. *Folia medica* **2012**, *54* (3), 42-49.
(355) Hu, H.-B.; Zheng, X.-D.; Jian, Y.-F.; Liu, J.-X.; Zhu, J.-H. Constituents of the root of Anemone tomentosa. *Archives of Pharmacal Research* **2011**, *34* (7), 1097-1105. DOI: 10.1007/s12272-011-0707-x.
(356) Zhang, B.; Chen, J.; Jiang, Q.; Wang, X.; Lu, Y.; Gong, L.; Chen, D. Simultaneous determination of three active components in rat plasma by UPLC-MS/MS: Application to pharmacokinetic study after oral administration of Herba Sarcandrae extract. *Biomedical Chromatography* **2017**, *31* (4). DOI: 10.1002/bmc.3834.
(357) Huang, H.-Y.; Ko, H.-H.; Jin, Y.-J.; Yang, S.-Z.; Shih, Y.-A.; Chen, I.-S. Dihydrochalcone glucosides and antioxidant activity from the roots of Anneslea fragrans var. lanceolata. *Phytochemistry* **2012**, *78*, 120-125. DOI: 10.1016/j.phytochem.2012.02.023.
(358) Deng, G.-g.; Gui, Z.-j.; Yang, X.-w. Chemical constituents from polarity part in roots of Angelica dahurica var. formosana cv. Chuanbaizhi. *China journal of Chinese materia medica* **2015**, *40* (19), 3805-3810.
(359) Phan Thi Anh, D.; Tran Le, Q.; Nguyen Thi Thanh, M. Constituents of the Stem of Nauclea orientalis. *Natural Product Communications* **2015**, *10* (11), 1901-1903.
(360) Doi, K.; Kojima, T.; Makino, M.; Kimura, Y.; Fujimoto, Y. Studies on the constituents of the leaves of Morus alba L. *Chemical & Pharmaceutical Bulletin* **2001**, *49* (2), 151-153. DOI: 10.1248/cpb.49.151.
(361) Huawu, Z.; Yiming, L.; Xiang, H.; Yuanying, J.; Shanhao, J.; Dayuan, Antiinflammatory and antioxidant effects of the extracts from Scrophularia ningpoensis Hemsl.[J]. **1999**, *9*.
(362) Kapoor, I. P. S.; Singh, B.; Singh, G.; De Heluani, C. S.; De Lampasona, M. P.; Catalan, C. A. N. Chemistry and antioxidant activity of essential oil and oleoresins of black caraway (Carum bulbocastanum) fruits: Part 69. *Journal of the Science of Food and Agriculture* **2010**, *90* (3), 385-390. DOI: 10.1002/jsfa.3824.
(363) Singh, G.; Marimuthu, P.; De Heluani, C. S.; Catalan, C. A. N. Antioxidant and biocidal activities of Carum nigrum (seed) essential oil, oleoresin, and their selected components. *Journal of Agricultural and Food Chemistry* **2006**, *54* (1), 174-181. DOI: 10.1021/jf0518610.
(364) Kikuzaki, H.; Hara, S.; Kawai, Y.; Nakatani, N. Antioxidative phenylpropanoids from berries of Pimenta dioica. *Phytochemistry* **1999**, *52* (7), 1307-1312. DOI: 10.1016/s0031-9422(99)00406-9.

(365) Leu, Y. L.; Wang, Y. L.; Huang, S. C.; Shi, L. S. Chemical constituents from roots of Taraxacum formosanum. *Chemical & pharmaceutical bulletin* **2005**, *53* (7), 853-855. DOI: 10.1248/cpb.53.853.
(366) Genovese, S.; Curini, M.; Epifano, F.; Menghini, L.; Ricci, D.; Fraternale, D.; Giamperi, L.; Bucchini, A.; Bellacchio, E. Antioxidant and lipoxygenase inhibitory activities of boropinic acid, active principle of Boronia pinnata. *Planta medica* **2006**, *72* (11), 987-987.
(367) Delazar, A.; Biglari, F.; Esnaashari, S.; Nazemiyeh, H.; Talebpour, A.-H.; Nahar, L.; Sarker, S. D. GC-MS analysis of the essential oils, and the isolation of phenylpropanoid derivatives from the aerial parts of Pimpinella aurea. *Phytochemistry* **2006**, *67* (19), 2176-2181. DOI: 10.1016/j.phytochem.2006.06.012.
(368) Tai, Z.; Chen, A.; Qin, B.; Cai, L.; Xu, Y. Chemical constituents and antioxidant activity of the Musa basjoo flower. *European Food Research and Technology* **2014**, *239* (3), 501-508. DOI: 10.1007/s00217-014-2244-6.
(369) Zhou, W.; Xie, H.; Xu, X.; Liang, Y.; Wei, X. Phenolic constituents from Isodon lophanthoides var. graciliflorus and their antioxidant and antibacterial activities. *Journal of Functional Foods* **2014**, *6*, 492-498. DOI: 10.1016/j.jff.2013.11.015.
(370) Al-Qudah, M. A.; Al-Jaber, H. I.; Abu Zarga, M. H.; Abu Orabi, S. T. Flavonoid and phenolic compounds from Salvia palaestina L. growing wild in Jordan and their antioxidant activities. *Phytochemistry* **2014**, *99*, 115-120. DOI: 10.1016/j.phytochem.2014.01.001.
(371) Yousuf, S.; Choudhary, M. I.; Rahman, A. U. Separation of phenylpropanoids and evaluation of their antioxidant activity. In *Advanced Protocols in Oxidative Stress II*, Springer, 2010; pp 357-377.
(372) Nićiforović, N.; Mihailović, V.; Mašković, P.; Solujić, S.; Stojković, A.; Muratspahić, D. P. J. F.; Toxicology, C. Antioxidant activity of selected plant species; potential new sources of natural antioxidants. **2010**, *48* (11), 3125-3130.
(373) Shin, S.-L.; Lee, C.-H. J. J. o. L. S. Antioxidant activities of ostrich fern by different extraction methods and solvents. **2011**, *21* (1), 56-61.
(374) Daels-Rakotoarison, D. A.; Seidel, V.; Gressier, B.; Brunet, C.; Tillequin, F.; Bailleul, F.; Luyckx, M.; Dine, T.; Cazin, M.; Cazin, J.-C. J. A. Neurosedative and antioxidant activities of phenylpropanoids from Ballota nigra. **2000**, *50* (01), 16-23.
(375) Hennebelle, T.; Sahpaz, S.; Gressier, B.; Joseph, H.; Bailleul, F. J. P. R. A. I. J. D. t. P.; Derivatives, T. E. o. N. P. Antioxidant and neurosedative properties of polyphenols and iridoids from Lippia alba. **2008**, *22* (2), 256-258.
(376) da Silva, F. R.; Rodrigues, F. E.; Gomes, A. R.; Arriaga, A.; Mafezoli, J.; Lemos, T. L.; Almeida, M.; Santiago, G. M.; Braz-Filho, R.; da Costa, J. G. J. Q. N. Phytochemical study, antioxidant and antibacterial activities of Stemodia maritima. **2014**, *37* (9), 1474-1478.
(377) Saracoglu, I.; Harput, U. S.; Inoue, M.; Ogihara, Y. New phenylethanoid glycosides from Veronica pectinata var. glandulosa and their free radical scavenging activities. *Chemical and pharmaceutical bulletin* **2002**, *50* (5), 665-668.
(378) Choudhary, M. I.; Begum, A.; Abbaskhan, A.; Musharraf, S. G.; Ejaz, A. J. C.; biodiversity. Two new antioxidant phenylpropanoids from Lindelofia stylosa. **2008**, *5* (12), 2676-2683.
(379) Waisundara, V. Y.; Huang, M.; Hsu, A.; Huang, D.; Tan, B. K.-H. J. T. A. j. o. C. m. Characterization of the anti-diabetic and antioxidant effects of Rehmannia glutinosa in streptozotocin-induced diabetic Wistar rats. **2008**, *36* (06), 1083-1104.
(380) He, Z.-D.; Lau, K.-M.; But, P. P.-H.; Jiang, R.-W.; Dong, H.; Ma, S.-C.; Fung, K.-P.; Ye, W.-C.; Sun, H.-D. Antioxidative Glycosides from the Leaves of Ligustrum r obustum. *Journal of natural products* **2003**, *66* (6), 851-854.
(381) Lin, Y.-L.; Chang, Y.-Y.; Kuo, Y.-H.; Shiao, M.-S. Anti-Lipid-Peroxidative Principles from Tournefortia s armentosa. *Journal of natural products* **2002**, *65* (5), 745-747.
(382) WANG, J.; LI, L.; LIU, C.-m.; LIU, Z.-j.; BAI, H.-l. J. L. J. o. T. C. M. Studies on the determination and antioxidant activities of total phenolic from different Chirita longgangensis var. hongyao extract [J]. **2011**, *3*.
(383) Wang, H.; Sun, Y.; Ye, W.-C.; Xiong, F.; Wu, J.-J.; Yang, C.-H.; Zhao, S.-X. Antioxidative phenylethanoid and phenolic glycosides from Picrorhiza scrophulariiflora. *Chemical and pharmaceutical bulletin* **2004**, *52* (5), 615-617.
(384) Chen, H.; Li, M.; Zhang, C.; Du, W.; Shao, H.; Feng, Y.; Zhang, W.; Yang, S. Isolation and Identification of the Anti-Oxidant Constituents from Loropetalum chinense (R-Brown) Oliv. Based on UHPLC-Q-TOF-MS/MS. *Molecules* **2018**, *23* (7). DOI: 10.3390/molecules23071720.
(385) Shi, J.; Gong, J.; Liu, J. e.; Wu, X.; Zhang, Y. Antioxidant capacity of extract from edible flowers of Prunus mume in China and its active components. *Lwt-Food Science and Technology* **2009**, *42* (2), 477-482. DOI: 10.1016/j.lwt.2008.09.008.

(386) Ganzon, J. G.; Chen, L. G.; Wang, C. C. 4-O-Caffeoylquinic acid as an antioxidant marker for mulberry leaves rich in phenolic compounds. *Journal of Food and Drug Analysis* **2018**, *26* (3), 985-993. DOI: 10.1016/j.jfda.2017.11.011.
(387) Hamauzu, Y.; Yasui, H.; Inno, T.; Kume, C.; Omanyuda, M. Phenolic profile, antioxidant property, and anti-influenza viral activity of Chinese quince (Pseudocydonia sinensis Schneid.), quince (Cydonia oblonga Mill.), and apple (Malus domestica Mill.) fruits. *Journal of Agricultural and Food Chemistry* **2005**, *53* (4), 928-934. DOI: 10.1021/jf0494635.
(388) Sun, Y.; Li, H.; Hu, J.; Li, J.; Fan, Y.-w.; Liu, X.-r.; Deng, Z.-y. Qualitative and Quantitative Analysis of Phenolics in Tetrastigma hemsleyanum and Their Antioxidant and Antiproliferative Activities. *Journal of Agricultural and Food Chemistry* **2013**, *61* (44), 10507-10515. DOI: 10.1021/jf4037547.
(389) Xiang, Z.; Ning, Z. Scavenging and antioxidant properties of compound derived from chlorogenic acid in South-China honeysuckle. *Lwt-Food Science and Technology* **2008**, *41* (7), 1189-1203. DOI: 10.1016/j.lwt.2007.08.006.
(390) Oliveira, I.; Valentao, P.; Lopes, R.; Andrade, P. B.; Bento, A.; Pereira, J. A. Phytochemical characterization and radical scavenging activity of Portulaca oleraceae L. leaves and stems. *Microchemical Journal* **2009**, *92* (2), 129-134. DOI: 10.1016/j.microc.2009.02.006.
(391) Dan, T.; Hui-Jun, L.; Jun, C.; Chao-Wei, G.; Ping, L. Rapid and simple method for screening of natural antioxidants from Chinese herb Flos Lonicerae Japonicae by DPPH-HPLC-DAD-TOF/MS. *Journal of separation science* **2015**, *31* (20), 3519-3526.
(392) Lee, W.-C.; Peng, C.-C.; Chang, C.-H.; Huang, S.-H.; Chyau, C.-C. Extraction of Antioxidant Components from Bidens pilosa Flowers and Their Uptake by Human Intestinal Caco-2 Cells. *Molecules* **2013**, *18* (2), 1582-1601. DOI: 10.3390/molecules18021582.
(393) Liu, L.; Sun, Y.; Laura, T.; Liang, X.; Ye, H.; Zeng, X. Determination of polyphenolic content and antioxidant activity of kudingcha made from Ilex kudingcha C.J. Tseng. *Food Chemistry* **2009**, *112* (1), 35-41. DOI: 10.1016/j.foodchem.2008.05.038.
(394) Zheleva-Dimitrova, D.; Gevrenova, R.; Zaharieva, M. M.; Najdenski, H.; Ruseva, S.; Lozanov, V.; Balabanova, V.; Yagi, S.; Momekov, G.; Mitev, V. HPLC-UV and LC–MS Analyses of Acylquinic Acids in Geigeria alata (DC) Oliv. & Hiern. and their Contribution to Antioxidant and Antimicrobial Capacity. *Phytochemical Analysis Pca* **2016**, *28*.
(395) da Silva, J. B.; Mendes, R. d. F.; Tomasco, V.; Campos Pinto, N. d. C.; de Oliveira, L. G.; Rodrigues, M. N.; de Oliveira Aragdoa, D. M.; Kopke de Aguiar, J. A.; Alves, M. S.; Nogueira Marques Castanon, M. C.; et al. New aspects on the hepatoprotective potential associated with the antioxidant, hypocholesterolemic and anti-inflammatory activities of Vernonia condensata Baker. *Journal of Ethnopharmacology* **2017**, *198*, 399-406. DOI: 10.1016/j.jep.2017.01.039.
(396) Pagano, I.; Piccinelli, A. L.; Celano, R.; Campone, L.; Gazzerro, P.; De Falco, E.; Rastrelli, L. Chemical profile and cellular antioxidant activity of artichoke by-products. *Food & Function* **2016**, *7* (12), 4841-4850. DOI: 10.1039/c6fo01443g.
(397) Salami, M.; Rahimmalek, M.; Ehtemam, M. H. Inhibitory effect of different fennel (Foeniculum vulgare) samples and their phenolic compounds on formation of advanced glycation products and comparison of antimicrobial and antioxidant activities. *Food Chemistry* **2016**, *213*, 196-205. DOI: 10.1016/j.foodchem.2016.06.070.
(398) Younsi, F.; Trimech, R.; Boulila, A.; Ezzine, O.; Dhahri, S.; Boussaid, M.; Messaoud, C. Essential Oil and Phenolic Compounds of Artemisia herba-alba (Asso.): Composition, Antioxidant, Antiacetylcholinesterase, and Antibacterial Activities. *International Journal of Food Properties* **2016**, *19* (7), 1425-1438. DOI: 10.1080/10942912.2015.1079789.
(399) Kobayashi, M.; Kakizono, T.; Nishio, N.; Nagai, S.; Kurimura, Y.; Tsuji, Y. J. A. M.; Biotechnology. Antioxidant role of astaxanthin in the green alga Haematococcus pluvialis. **1997**, *48* (3), 351-356.
(400) Kobayashi, M. J. A. m.; biotechnology. In vivo antioxidant role of astaxanthin under oxidative stress in the green alga Haematococcus pluvialis. **2000**, *54* (4), 550-555.
(401) Kuroyanagi, M.; Shimomae, M.; Nagashima, Y.; Muto, N.; Okuda, T.; Kawahara, N.; Nakane, T.; Sano, T. New diarylheptanoids from Alnus japonica and their antioxidative activity. *Chemical & pharmaceutical bulletin* **2005**, *53* (12), 1519-1523. DOI: 10.1248/cpb.53.1519.
(402) Wicha, P.; Tocharus, J.; Janyou, A.; Jittiwat, J.; Changtam, C.; Suksamrarn, A.; Tocharus, C. Hexahydrocurcumin protects against cerebral ischemia/reperfusion injury, attenuates inflammation, and improves antioxidant defenses in a rat stroke model. *PloS one* **2017**, *12* (12). DOI: 10.1371/journal.pone.0189211.
(403) Guo, X.; Li, W. F.; Wang, H. P.; Fan, Y. Y.; Wang, H. F.; Gao, X. H.; Niu, B. L.; Gong, X. C. Preparation, characterization, release and antioxidant activity of curcumin-loaded amorphous calcium phosphate nanoparticles. *Journal of Non-Crystalline Solids* **2018**, *500*, 317-325. DOI: 10.1016/j.jnoncrysol.2018.08.015.
(404) Serqueira, D. S.; Dornellas, R. M.; Silva, L. G.; de Melo, P. G.; Castellan, A.; Ruggiero, R.; Richter, E. M.; Munoz, R. A. A. Tetrahydrocurcuminoids as potential antioxidants for biodiesels. *Fuel* **2015**, *160*, 490-494. DOI: 10.1016/j.fuel.2015.07.104.
(405) Yu, Q. Q.; Liu, Y. Y.; Wu, Y. F.; Chen, Y. Dihydrocurcumin ameliorates the lipid accumulation, oxidative stress and insulin resistance in oleic acid-induced L02 and HepG2 cells. *Biomedicine & Pharmacotherapy* **2018**, *103*, 1327-1336. DOI: 10.1016/j.biopha.2018.04.143.

(406) Akter, J.; Hossain, M. A.; Takara, K.; Islam, M. Z.; Hou, D.-X. Antioxidant activity of different species and varieties of turmeric (Curcuma spp): Isolation of active compounds. *Comparative biochemistry and physiology. Toxicology & pharmacology : CBP* **2018**, *215*, 9-17. DOI: 10.1016/j.cbpc.2018.09.002.
(407) dos Santos, P. D. F.; Francisco, C. R. L.; Coqueiro, A.; Leimann, F. V.; Pinela, J.; Calhelha, R. C.; Ineu, R. P.; Ferreira, I.; Bona, E.; Goncalves, O. H. The nanoencapsulation of curcuminoids extracted from Curcuma longa L. and an evaluation of their cytotoxic, enzymatic, antioxidant and anti-inflammatory activities. *Food & Function* **2019**, *10* (2), 573-582. DOI: 10.1039/c8fo02431f.
(408) Puglia, C.; Santonocito, D.; Musumeci, T.; Cardile, V.; Graziano, A. C. E.; Salerno, L.; Raciti, G.; Crasci, L.; Panico, A. M.; Puglisi, G. Nanotechnological Approach to Increase the Antioxidant and Cytotoxic Efficacy of Crocin and Crocetin. *Planta Medica* **2019**, *85* (3), 258-265. DOI: 10.1055/a-0732-5757.
(409) Watanabe, M.; Ayugase, J. Improvement in Lipid Metabolism by Carotenoid Extracts from Pink Colored and Orange Colored Tomatoes in Mice Subjected to Restraint Stress. *Journal of the Japanese Society for Food Science and Technology-Nippon Shokuhin Kagaku Kogaku Kaishi* **2013**, *60* (7), 332-338, Article.
(410) Can-Cauich, C. A.; Sauri-Duch, E.; Moo-Huchin, V. M.; Betancur-Ancona, D.; Cuevas-Glory, L. F. Effect of extraction method and specie on the content of bioactive compounds and antioxidant activity of pumpkin oil from Yucatan, Mexico. *Food Chemistry* **2019**, *285*, 186-193. DOI: 10.1016/j.foodchem.2019.01.153.
(411) Galano, A.; Francisco-Marquez, M.; Alvarez-Idaboy, J. R. Mechanism and kinetics studies on the antioxidant activity of sinapinic acid. *Phys Chem Chem Phys* **2011**, *13* (23), 11199-11205. DOI: 10.1039/c1cp20722a.
(412) Zhang, N.; Lian, Z.; Peng, X.; Li, Z.; Zhu, H. Applications of Higenamine in pharmacology and medicine. *J Ethnopharmacol* **2017**, *196*, 242-252. DOI: 10.1016/j.jep.2016.12.033.
(413) Li, X.; Lin, J.; Chen, B.; Xie, H.; Chen, D. Antioxidant and Cytoprotective Effects of Kukoamines A and B: Comparison and Positional Isomeric Effect. *Molecules* **2018**, *23* (4). DOI: 10.3390/molecules23040973.
(414) Zhao, Q.; Zhao, Y.; Wang, K. Antinociceptive and free radical scavenging activities of alkaloids isolated from Lindera angustifolia Chen. *Journal of Ethnopharmacology* **2006**, *106* (3), 408-413. DOI: 10.1016/j.jep.2006.01.019.
(415) Costa, E. V.; Oliveira da Cruz, P. E.; de Lourenco, C. C.; de Souza Moraes, V. R.; de Lima Nogueira, P. C.; Salvador, M. J. Antioxidant and antimicrobial activities of aporphinoids and other alkaloids from the bark of Annona salzmannii A. DC. (Annonaceae). *Natural product research* **2013**, *27* (11), 1002-1006. DOI: 10.1080/14786419.2012.688044.
(416) Bezakova, L.; Misik, V.; Malekova, L.; Svajdlenka, E.; Kostalova, D. Lipoxygenase inhibition and antioxidant properties of bisbenzylisoquinoline alkaloids isolated from Mahonia aquifolium. *Pharmazie* **1996**, *51* (10), 758-761.
(417) Jia, F.; Ruan, S.; Liu, N.; Fu, L. Synergistic Antitumor Effects of Berbamine and Paclitaxel through ROS/Akt Pathway in Glioma Cells. *Evidence-based complementary and alternative medicine : eCAM* **2017**, *2017*, 8152526-8152526. DOI: 10.1155/2017/8152526.
(418) Zarei, A.; Changizi-Ashtiyani, S.; Taheri, S.; Ramezani, M. A quick overview on some aspects of endocrinological and therapeutic effects of Berberis vulgaris L. *Avicenna journal of phytomedicine* **2015**, *5* (6), 485-497.
(419) Khan, A.; Bai, H.; Liu, E.; Chen, M.; Yu, C.; Wang, R.; Khan, A.; Bai, Z. Protective effect of neferine against UV-B-mediated oxidative damage in human epidermal keratinocytes. *Journal of Dermatological Treatment* **2018**, *29* (7), 733-741. DOI: 10.1080/09546634.2018.1441490.
(420) Xie, Y.; Zhang, Y.; Guo, Z.; Zeng, H.; Zheng, B. Effects of Neferine from Nelumbinis Plumula on Oleic Acid-Induced Hepatic Steatosis in Vitro via Its Antioxidant Activity. *Current Topics in Nutraceutical Research* **2017**, *15* (3-4), 195-204.
(421) Gulcin, I.; Elias, R.; Gepdiremen, A.; Chea, A.; Topal, F. Antioxidant activity of bisbenzylisoquinoline alkaloids from Stephania rotunda: cepharanthine and fangchinoline. *Journal of Enzyme Inhibition and Medicinal Chemistry* **2010**, *25* (1), 44-53. DOI: 10.3109/14756360902932792.
(422) Gomez, G. I.; Velarde, V. Boldine Improves Kidney Damage in the Goldblatt 2K1C Model Avoiding the Increase in TGF-beta. *International Journal of Molecular Sciences* **2018**, *19* (7). DOI: 10.3390/ijms19071864.
(423) Moezi, L.; Yahosseini, S.; Jamshizadeh, A.; Pirsalami, F. Acute Boldine Treatment Induces Anti-convulsant Effects in Mice through its Antioxidant Activity. *Drug research* **2018**. DOI: 10.1055/a-0659-2478.
(424) Zahari, A.; Ablat, A.; Siyasothy, Y.; Mohamad, J.; Choudhary, M. I.; Awang, K. In vitro antiplasmodial and antioxidant activities of bisbenzylisoquinoline alkaloids from Alseodaphne corneri Kosterm. *Asian Pacific Journal of Tropical Medicine* **2016**, *9* (4), 320-324. DOI: 10.1016/j.apjtm.2016.03.008.
(425) Li, C.; Wang, M.-H. Potential Biological Activities of Magnoflorine: A Compound from Aristolochia debilis Sieb. et Zucc. *Korean Journal of Plant Reources.* **2014**, *27* (3), 223-228. DOI: 10.7732/kjpr.2014.27.3.223.

(426) Luo, T.; Shen, X.-Y.; Li, S.; Ouyang, T.; Mai, Q.-A.; Wang, H.-Q. The Protective Effect of Jatrorrhizine Against Oxidative Stress in Primary Rat Cortical Neurons. *Cns & Neurological Disorders-Drug Targets* **2017**, *16* (5), 617-623. DOI: 10.2174/1871527315666160711101210.
(427) Ubeda, A.; Montesinos, C.; Paya, M.; Terencio, C.; Alcaraz, M. J. Antioxidant action of benzylisoquinoline alkaloids. *Free radical research communications* **1993**, *18* (3), 167-175. DOI: 10.3109/10715769309147490.
(428) Fan, H.; Shu, Q.; Guan, X.; Zhao, J.; Yan, J.; Li, X.; Liu, J.; Jia, Z.; Shi, J.; Li, J. Sinomenine Protects PC12 Neuronal Cells against H2O2-induced Cytotoxicity and Oxidative Stress via a ROS-dependent Up-regulation of Endogenous Antioxidant System. *Cellular and Molecular Neurobiology* **2017**, *37* (8), 1387-1398. DOI: 10.1007/s10571-017-0469-1.
(429) Zhou, Y.; Liu, H.; Song, J.; Cao, L.; Tang, L.; Qi, C. Sinomenine alleviates dextran sulfate sodium-induced colitis via the Nrf2/NQO-1 signaling pathway. *Molecular Medicine Reports* **2018**, *18* (4), 3691-3698. DOI: 10.3892/mmr.2018.9378.
(430) Kiplimo, J. J.; Islam, M. S.; Koorbanally, N. A. A Novel Flavonoid and Furoquinoline Alkaloids from Vepris glomerata and their Antioxidant Activity. *Natural Product Communications* **2011**, *6* (12), 1847-1850.
(431) Kim, D. H.; Jang, Y. Y.; Han, E. S.; Lee, C. S. Protective effect of harmaline and harmalol against dopamine- and 6-hydroxydopamine-induced oxidative damage of brain mitochondria and synaptosomes, and viability loss of PC12 cells. *European Journal of Neuroscience* **2001**, *13* (10), 1861-1872. DOI: 10.1046/j.0953-816x.2001.01563.x.
(432) Su, S.; Cao, M.; Wu, G.; Long, Z.; Cheng, X.; Fan, J.; Xu, Z.; Su, H.; Hao, Y.; Li, G.; et al. Hordenine protects against hyperglycemia-associated renal complications in streptozotocin-induced diabetic mice. *Biomedicine & Pharmacotherapy* **2018**, *104*, 315-324. DOI: 10.1016/j.biopha.2018.05.036.
(433) Li, X.; Ren, Z.; Wu, Z.; Fu, Z.; Xie, H.; Deng, L.; Jiang, X.; Chen, D. Steric Effect of Antioxidant Diels-Alder-Type Adducts: A Comparison of Sanggenon C with Sanggenon D. *Molecules (Basel, Switzerland)* **2018**, *23* (10). DOI: 10.3390/molecules23102610.
(434) Chen, L.-D.; Liu, Z.-H.; Zhang, L.-F.; Yao, J.-N.; Wang, C.-F. Sanggenon C induces apoptosis of colon cancer cells via inhibition of NO production, iNOS expression and ROS activation of the mitochondrial pathway. *Oncology reports* **2017**, *38* (4), 2123-2131. DOI: 10.3892/or.2017.5912.
(435) Zhang, Q.-J.; Ni, G.; Wang, Y.-H.; Chen, R.-Y.; Yu, D.-Q. Three new Diels-Alder type adducts from the stem bark of Morus cathayana. *Journal of Asian Natural Products Research* **2009**, *11* (3), 267-273. DOI: 10.1080/10286020902767690.
(436) Cui, X.-Q.; Wang, H.-Q.; Liu, C.; Chen, R.-Y. Study of anti-oxidant phenolic compounds from stem barks of Morus yunanensis. *Zhongguo Zhong yao za zhi = Zhongguo zhongyao zazhi = China journal of Chinese materia medica* **2008**, *33* (13), 1569-1572.
(437) Dai, S. J.; Wu, Y.; Wang, Y. H.; He, W. Y.; Chen, R. Y.; Yu, D. Q. New Diels-Alder type adducts from Morus macroura and their anti-oxidant activities. *Chemical & pharmaceutical bulletin* **2004**, *52* (10), 1190-1193. DOI: 10.1248/cpb.52.1190.
(438) Hong, S.; Kwon, J.; Kim, D.-W.; Lee, H. J.; Lee, D.; Mar, W. Mulberrofuran G Protects Ischemic Injury-induced Cell Death via Inhibition of NOX4-mediated ROS Generation and ER Stress. *Phytotherapy Research* **2017**, *31* (2), 321-329. DOI: 10.1002/ptr.5754.
(439) Abbas, G. M.; Bar, F. M. A.; Baraka, H. N.; Gohar, A. A.; Lahloub, M.-F. A new antioxidant stilbene and other constituents from the stem bark of Morus nigra L. *Natural product research* **2014**, *28* (13), 952-959. DOI: 10.1080/14786419.2014.900770.
(440) Dai, S. J.; Ma, Z. B.; Wu, Y.; Chen, R. Y.; Yu, D. Q. Guangsangons F-J, anti-oxidant and anti-inflammatory Diels-Alder type adducts, from Morus macroura Miq. *Phytochemistry* **2004**, *65* (23), 3135-3141. DOI: 10.1016/j.phytochem.2004.07.010.
(441) Tan, Y.-X.; Yan, R.-Y.; Wang, H.-Q.; Chen, R.-Y.; Yu, D.-Q. Wittiorumins A-F, Antioxidant Diels-Alder-Type Adducts from Morus wittiorum. *Planta medica* **2009**, *75* (3), 249-255. DOI: 10.1055/s-0028-1112202.
(442) Wang, T.; Li, L.; Li, Y.; Wu, J.; Huang, Y.; Wei, G.; Chen, D. Mechanistic Chemistry of Extraordinary Capacity of Salvianolic Acid B on Oxidativelydamaged Mesenchymal Stem Cells. *J. Chin. Chem. Soc.* **2016**, *63*, 924–929. DOI: 10.1002/jccs.201600112
(443) Zhou, R. F.; Long, H. S.; Zhang, B.; Lao, Z. Z.; Zheng, Q. Y.; Wang, T. C.; Zhang, Y. X.; Wu, Q. G.; Lai, X. P.; Li, G.; et al. Salvianolic acid B, an antioxidant derived from Salvia militarize, protects mice against -radiation-induced damage through Nrf2/Bach1. *Molecular Medicine Reports* **2019**, *19* (2), 1309-1317. DOI: 10.3892/mmr.2018.9718.
(444) Chan, P. C.; Xia, Q. S.; Fu, P. P. Ginkgo biloba leave extract: Biological, medicinal, and toxicological effects. *Journal of Environmental Science and Health Part C-Environmental Carcinogenesis & Ecotoxicology Reviews* **2007**, *25* (3), 211-244, Review. DOI: 10.1080/10590500701569414.
(445) Nenadis, N.; Lazaridou, O.; Tsimidou, M. Z. Use of reference compounds in antioxidant activity assessment. *J Agric Food Chem* **2007**, *55* (14), 5452-5460. DOI: 10.1021/jf070473q.
(446) Lee, M. S.; Shin, T.; Utsuki, T.; Choi, J. S.; Byun, D. S.; Kim, H. R. Isolation and Identification of Phlorotannins from Ecklonia stolonifera with Antioxidant and Hepatoprotective Properties in Tacrine-Treated HepG2 Cells. *Journal of Agricultural and Food Chemistry* **2012**, *60* (21), 5340-5349. DOI: 10.1021/jf300157w.

(447) Shibata, T.; Ishimaru, K.; Kawaguchi, S.; Yoshikawa, H.; Hama, Y. Antioxidant activities of phlorotannins isolated from Japanese Laminariaceae. *Journal of Applied Phycology* **2008**, *20* (5), 705-711. DOI: 10.1007/s10811-007-9254-8.
(448) Gupta, R.; Sharma, A. K.; Sharma, M. C.; Gupta, R. S. Antioxidant activity and protection of pancreatic ss-cells by embelin in streptozotocin-induced diabetes. *Journal of Diabetes* **2012**, *4* (3), 248-256. DOI: 10.1111/j.1753-0407.2012.00187.x.
(449) Mahendran, S.; Badami, S.; Ravi, S.; Thippeswamy, B. S.; Veerapur, V. P. Antioxidant, Analgesic and Anti-Inflammatory Properties Of New Ninhydrin Adduct of Embelin. *Pharmaceutical Chemistry Journal* **2011**, *45* (9), 547-551. DOI: 10.1007/s11094-011-0676-x.
(450) Lee, M.-S.; Kim, J.-I.; Utsuki, T.; Park, N.-G.; Kim, H.-R. Cytoprotective effects of phlorofucofuroeckol A isolated from Ecklonia stolonifera against tacrine-treated HepG2 cells. *Fitoterapia* **2012**, *83* (6), 1060-1067. DOI: 10.1016/j.fitote.2012.05.007.
(451) Masaki, H.; Atsumi, T.; Sakurai, H. Hamamelitannin As a New Potent Active Oxygen Scavenger. *Phytochemistry* **1994**, *37* (2), 337-343. DOI: 10.1016/0031-9422(94)85057-7.
(452) Sanchez-Tena, S.; Fernandez-Cachon, M. L.; Carreras, A.; Luisa Mateos-Martin, M.; Costoya, N.; Moyer, M. P.; Nunez, M. J.; Torres, J. L.; Cascante, M. Hamamelitannin from Witch Hazel (Hamamelis virginiana) Displays Specific Cytotoxic Activity against Colon Cancer Cells. *Journal of Natural Products* **2012**, *75* (1), 26-33. DOI: 10.1021/np200426k.
(453) Aree, T.; Jongrungruangchok, S. Structure-antioxidant activity relationship of β-cyclodextrin inclusion complexes with olive tyrosol, hydroxytyrosol and oleuropein: Deep insights from X-ray analysis, DFT calculation and DPPH assay. *Carbohydrate polymers* **2018**, *199*, 661-669. DOI: 10.1016/j.carbpol.2018.07.019.
(454) Liu, M.; Yong, Q.; Yu, S. Efficient bioconversion of oleuropein from olive leaf extract to antioxidant hydroxytyrosol by enzymatic hydrolysis and high-temperature degradation. *Biotechnology and Applied Biochemistry* **2018**, *65* (5), 680-689. DOI: 10.1002/bab.1651.
(455) Andrade, M. A.; Ribeiro-Santos, R.; Conceicao Costa Bonito, M.; Saraiva, M.; Sanches-Silva, A. Characterization of rosemary and thyme extracts for incorporation into a whey protein based film. *Lwt-Food Science and Technology* **2018**, *92*, 497-508. DOI: 10.1016/j.lwt.2018.02.041.
(456) Alejandro Hernandez, D.; Tenorio, F. J. Reactivity indexes of antioxidant molecules from Rosmarinus officinalis. *Structural Chemistry* **2018**, *29* (3), 741-751. DOI: 10.1007/s11224-017-1066-0.
(457) Tripathi, Y. B.; Sharma, M. Comparison of the antioxidant action of the alcoholic extract of Rubia cordifolia with rubiadin. *Indian Journal of Biochemistry & Biophysics* **1998**, *35* (5), 313-316.
(458) Tripathi, Y. B.; Sharma, M.; Manickam, M. Rubiadin, a new antioxidant from Rubia cordifolia. *Indian Journal of Biochemistry & Biophysics* **1997**, *34* (3), 302-306.
(459) Han, H.; Weng, X.; Bi, K. Antioxidants from a Chinese medicinal herb - Lithospermum erythrorhizon. *Food Chemistry* **2008**, *106* (1), 2-10. DOI: 10.1016/j.foodchem.2007.01.031.
(460) Janicsak, G.; Hohmann, J.; Zupko, I.; Forgo, P.; Redei, D.; Falkay, G.; Mathe, I. Diterpenes from the aerial parts of Salvia candelabrum and their protective effects against lipid peroxidation. *Planta medica* **2003**, *69* (12), 1156-1159.
(461) Mathe, I.; Hohmann, J.; Janicsak, G.; Nagy, G.; Dora, R. Chemical diversity of the biological active ingredients of salvia officinalis and some closely related species. *Acta pharmaceutica Hungarica* **2007**, *77* (1), 37-45.
(462) Ozarowski, M.; Piasecka, A.; Gryszczynska, A.; Sawikowska, A.; Pietrowiak, A.; Opala, B.; Mikolajczak, P. L.; Kujawski, R.; Kachlicki, P.; Buchwald, W.; et al. Determination of phenolic compounds and diterpenes in roots of Salvia miltiorrhiza and Salvia przewalskii by two LC-MS tools: Multi-stage and high resolution tandem mass spectrometry with assessment of antioxidant capacity. *Phytochemistry Letters* **2017**, *20*, 331-338. DOI: 10.1016/j.phytol.2016.12.001.
(463) Christopher, R.; Nyandoro, S. S.; Chacha, M.; de Koning, C. B. A new cinnamoylglycoflavonoid, antimycobacterial and antioxidant constituents from Heritiera littoralis leaf extracts. *Natural product research* **2014**, *28* (6), 351-358. DOI: 10.1080/14786419.2013.863202.
(464) Khalil, A. A.; Khan, M. R.; Shabbir, M. A.; Khalil ur, R. In Vitro Antioxidant Activity and Punicalagin Content Quantification Of Pomegranate Peel Obtained As Agro-Waste After Juice Extraction. *Pakistan Journal of Agricultural Sciences* **2018**, *55* (1), 197-201. DOI: 10.21162/pakjas/18.5663.
(465) Oudane, B.; Boudemagh, D.; Bounekhel, M.; Sobhi, W.; Vidal, M.; Broussy, S. Isolation, characterization, antioxidant activity, and protein-precipitating capacity of the hydrolyzable tannin punicalagin from pomegranate yellow peel (Punica granatum). *Journal of Molecular Structure* **2018**, *1156*, 390-396. DOI: 10.1016/j.molstruc.2017.11.129.
(466) Bhattacharjee, P.; Bera, I.; Chakraborty, S.; Ghoshal, N.; Bhattacharyya, D. Aristolochic acid and its derivatives as inhibitors of snake venom L-amino acid oxidase. *Toxicon* **2017**, *138*, 1-17. DOI: 10.1016/j.toxicon.2017.08.003.
(467) Aree, T.; Jongrungruangchok, S. β-Cyclodextrin encapsulation elevates antioxidant capacity of tea: A closing chapter on non-epicatechins, atomistic insights from X-ray analysis, DFT calculation and DPPH assay. *Carbohydrate polymers* **2018**, *194*, 24-33. DOI: 10.1016/j.carbpol.2018.04.016.

(468) Martini, S.; Conte, A.; Tagliazucchi, D. Bioaccessibility, bioactivity and cell metabolism of dark chocolate phenolic compounds after in vitro gastro-intestinal digestion. *Journal of Functional Foods* **2018**, *49*, 424-436. DOI: 10.1016/j.jff.2018.09.005.
(469) Lad, H.; Bhatnagar, D. Amelioration of oxidative and inflammatory changes by Swertia chirayita leaves in experimental arthritis. *Inflammopharmacology* **2016**, *24* (6), 363-375. DOI: 10.1007/s10787-016-0290-3.
(470) He, Z. D.; But, P. P. H.; Chan, T. W. D.; Dong, H.; Xu, H. X.; Lau, C. P.; Sun, H. D. Antioxidative glucosides from the fruits of Ligustrum lucidum. *Chemical & pharmaceutical bulletin* **2001**, *49* (6), 780-784. DOI: 10.1248/cpb.49.780.
(471) Ma, S. C.; He, Z. D.; Deng, X. L.; But, P. P. H.; Ooi, V. E. C.; Xu, H. X.; Lee, S. H. S.; Lee, S. F. In vitro evaluation of secoiridoid glucosides from the fruits of Ligustrum lucidum as antiviral agents. *Chemical & pharmaceutical bulletin* **2001**, *49* (11), 1471-1473. DOI: 10.1248/cpb.49.1471.
(472) Tada, M.; Ohkanda, T.; Kurabe, J. Syntheses of Carnosic Acid and Carnosol, Anti-oxidants in Rosemary, from Pisiferic Acid, the Major Constituent of Sawara. *Chemical & pharmaceutical bulletin* **2010**, *58* (1), 27-29. DOI: 10.1248/cpb.58.27.
(473) Yatagai, M.; Nakatani, N. Antimite, Antifly, Antioxidative, And Antibacterial Activities Of Pisiferic Acid And Its Congeners. *Mokuzai Gakkaishi* **1994**, *40* (12), 1355-1362.
(474) Narukawa, Y.; Shimizu, N.; Shimotohno, K.; Takeda, T. Two new diterpenoids from Plectranthus nummularius BRIQ. *Chemical & pharmaceutical bulletin* **2001**, *49* (9), 1182-1184. DOI: 10.1248/cpb.49.1182.
(475) Quintero, C.; Vera, R.; Dario Perez, L. alpha-Tocopherol loaded thermosensitive polymer nanoparticles: preparation, in vitro release and antioxidant properties. *Polimeros-Ciencia E Tecnologia* **2016**, *26* (4), 304-312. DOI: 10.1590/0104-1428.2324.
(476) Pantoja-Chamorro, A. L.; Hurtado-Benavides, A. M.; Martínez-Correa, H. A. Evaluación del Rendimiento, Composición y Actividad Antioxidante de Aceite de Semillas de Mora (Rubus glaucus) Extraído con CO2 Supercrítico. *Información tecnológica* **2017**, *28* (1), 35-46.
(477) Iwatake, M.; Okamoto, K.; Tanaka, T.; Tsukuba, T. Castalagin Exerts Inhibitory Effects on Osteoclastogenesis Through Blocking a Broad Range of Signaling Pathways with Low Cytotoxicity. *Phytotherapy Research* **2015**, *29* (6), 917-924. DOI: 10.1002/ptr.5333.
(478) Kaneshima, T.; Myoda, T.; Nakata, M.; Fujimori, T.; Toeda, K.; Nishizawa, M. Antioxidant activity of C-Glycosidic ellagitannins from the seeds and peel of camu-camu (Myrciaria dubia). *Lwt-Food Science and Technology* **2016**, *69*, 76-81. DOI: 10.1016/j.lwt.2016.01.024.
(479) Aroso, I. M.; Araujo, A. R.; Fernandes, J. P.; Santos, T.; Batista, M. T.; Pires, R. A.; Mano, J. F.; Reis, R. L. Hydroalcoholic extracts from the bark of Quercus suber L. (Cork): optimization of extraction conditions, chemical composition and antioxidant potential. *Wood Science and Technology* **2017**, *51* (4), 855-872. DOI: 10.1007/s00226-017-0904-y.
(480) Muccilli, V.; Cardullo, N.; Spatafora, C.; Cunsolo, V.; Tringali, C. alpha-Glucosidase inhibition and antioxidant activity of an oenological commercial tannin. Extraction, fractionation and analysis by HPLC/ESI-MS/MS and H-1 NMR. *Food Chemistry* **2017**, *215*, 50-60. DOI: 10.1016/j.foodchem.2016.07.136.
(481) Kahkonen, M.; Kylli, P.; Ollilainen, V.; Salminen, J.-P.; Heinonen, M. Antioxidant Activity of Isolated Ellagitannins from Red Raspberries and Cloudberries. *Journal of Agricultural and Food Chemistry* **2012**, *60* (5), 1167-1174. DOI: 10.1021/jf203431g.
(482) Zamurrad, S.; Parrino, S.; Daccueil, F.; Roemer, E. J.; Simon, S. R. In vitro investigation of antioxidant and antiproteolytic properties of the clove extracts: Tellimagrandin I and Casuarictin. *In Vitro Cellular & Developmental Biology-Animal* **2008**, *44*, S47-S47.
(483) Luo, W.; Zhao, M.; Yang, B.; Ren, J.; Shen, G.; Rao, G. Antioxidant and antiproliferative capacities of phenolics purified from Phyllanthus emblica L. fruit. *Food Chemistry* **2011**, *126* (1), 277-282. DOI: 10.1016/j.foodchem.2010.11.018.
(484) Reddy, D. B.; Reddanna, P. Chebulagic acid (CA) attenuates LPS-induced inflammation by suppressing NF-kappa B and MAPK activation in RAW 264.7 macrophages. *Biochemical and Biophysical Research Communications* **2009**, *381* (1), 112-117. DOI: 10.1016/j.bbrc.2009.02.022.
(485) Klika, K. D.; Saleem, A.; Sinkkonen, J.; Kahkonen, M.; Loponen, J.; Tahtinen, P.; Pihlaja, K. The structural and conformational analyses and antioxidant activities of chebulinic acid and its thrice-hydrolyzed derivative, 2,4-chebuloyl-β-D-glucopyranoside, isolated from the fruit of Terminalia chebula. *Arkivoc* **2004**, 83-105.
(486) Upadhyay, A.; Agrahari, P.; Singh, D. K. A Review on the Pharmacological Aspects of Terminalia chebula. *International Journal of Pharmacology* **2014**, *10* (6), 289-298. DOI: 10.3923/ijp.2014.289.298.
(487) Ding, Y.; Ren, D.; Xu, H.; Liu, W.; Liu, T.; Li, L.; Li, J.; Li, Y.; Wen, A. Antioxidant and pro-angiogenic effects of corilagin in rat cerebral ischemia via Nrf2 activation. *Oncotarget* **2017**, *8* (70), 114816-114828. DOI: 10.18632/oncotarget.22023.
(488) Reddy, B. U.; Mullick, R.; Kumar, A.; Sharma, G.; Bag, P.; Roy, C. L.; Sudha, G.; Tandon, H.; Dave, P.; Shukla, A.; et al. A natural small molecule inhibitor corilagin blocks HCV replication and modulates oxidative stress to reduce liver damage. *Antiviral Research* **2018**, *150*, 47-59. DOI: 10.1016/j.antiviral.2017.12.004.

(489) Aayadi, H.; Mittal, S. P. K.; Deshpande, A.; Gore, M.; Ghaskadbi, S. S. Cytoprotective effect exerted by geraniin in HepG2 cells is through microRNA mediated regulation of BACH-1 and HO-1. *Bmb Reports* **2017**, *50* (11), 560-565. DOI: 10.5483/BMBRep.2017.50.11.060.

(490) Chung, A. P. Y. S.; Gurtu, S.; Chakravarthi, S.; Moorthy, M.; Palanisamy, U. D. Geraniin Protects High-Fat Diet-Induced Oxidative Stress in Sprague Dawley Rats. *Frontiers in nutrition* **2018**, *5*, 17-17. DOI: 10.3389/fnut.2018.00017.

(491) Tabata, H.; Katsube, T.; Tsuma, T.; Ohta, Y.; Imawaka, N.; Utsumi, T. Isolation and evaluation of the radical-scavenging activity of the antioxidants in the leaves of an edible plant, Mallotus japonicus. *Food Chemistry* **2008**, *109* (1), 64-71. DOI: 10.1016/j.foodchem.2007.12.017.

(492) Yang, C.; Li, F.; Zhang, X.; Wang, L.; Zhou, Z.; Wang, M. Phenolic antioxidants from Rosa soulieana flowers. *Natural product research* **2013**, *27* (21), 2055-2058. DOI: 10.1080/14786419.2013.811660.

(493) Zhou, B.; Yang, L.; Liu, Z. L. Strictinin as an efficient antioxidant in lipid peroxidation. *Chemistry and Physics of Lipids* **2004**, *131* (1), 15-25. DOI: 10.1016/j.chemphyslip.2004.03.007.

(494) Yin, T.-P.; Cai, L.; Chen, Y.; Li, Y.; Wang, Y.-R.; Liu, C.-S.; Ding, Z.-T. Tannins and Antioxidant Activities of the Walnut (Juglans regia) Pellicle. *Natural Product Communications* **2015**, *10* (12), 2141-2144.

(495) Eyles, A.; Davies, N. W.; Mitsunaga, T.; Mihara, R.; Mohammed, C. Role of Eucalyptus globulus wound wood extractives: evidence of superoxide dismutase-like activity. *Forest Pathology* **2004**, *34* (4), 225-232. DOI: 10.1111/j.1439-0329.2004.00361.x.

(496) Chen, Y.; Wang, J.; Ou, Y.; Chen, H.; Xiao, S.; Liu, G.; Cao, Y.; Huang, Q. Cellular antioxidant activities of polyphenols isolated from Eucalyptus leaves (Eucalyptus grandis x Eucalyptus urophylla GL9). *Journal of Functional Foods* **2014**, *7*, 737-745. DOI: 10.1016/j.jff.2013.12.003.

(497) Ganesan, D.; Al-Sayed, E.; Albert, A.; Paul, E.; Singab, A. N. B.; Sadasivam, S. G.; Saso, L. Antioxidant activity of phenolic compounds from extracts of Eucalyptus globulus and Melaleuca styphelioides and their protective role on D-glucose-induced hyperglycemic stress and oxalate stress in NRK-49Fcells. *Natural product research* **2018**, *32* (11), 1274-1280. DOI: 10.1080/14786419.2017.1343324.

(498) Chang, M. Y.; Byon, S.-H.; Shin, H.-C.; Han, S. E.; Kim, J. Y.; Byun, J. Y.; Lee, J. D.; Park, M. K. Protective effects of the seaweed phlorotannin polyphenolic compound dieckol on gentamicin-induced damage in auditory hair cells. *International Journal of Pediatric Otorhinolaryngology* **2016**, *83* (4), 31-36. DOI: 10.1016/j.ijporl.2016.01.014.

(499) Lee, M.-S.; Lee, B.; Park, K.-E.; Utsuki, T.; Shin, T.; Oh, C. W.; Kim, H.-R. Dieckol enhances the expression of antioxidant and detoxifying enzymes by the activation of Nrf2-MAPK signaling pathway in HepG2 cells (vol 174, pg 538, 2015). *Food Chemistry* **2016**, *202*, 521-521. DOI: 10.1016/j.foodchem.2016.02.084.

(500) Kang, H. S.; Chung, H. Y.; Jung, J. H.; Son, B. W.; Choi, J. S. A new phlorotannin from the brown alga Ecklonia stolonifera. *Chemical & pharmaceutical bulletin* **2003**, *51* (8), 1012-1014.

(501) Jang, J.; Ye, B.-R.; Heo, S.-J.; Oh, C.; Kang, D.-H.; Kim, J. H.; Affan, A.; Yoon, K.-T.; Choi, Y.-U.; Park, S. C.; et al. Photo-oxidative stress by ultraviolet-B radiation and antioxidative defense of eckstolonol in human keratinocytes. *Environmental Toxicology and Pharmacology* **2012**, *34* (3), 926-934. DOI: 10.1016/j.etap.2012.08.003.

(502) Kang, H. S.; Chung, H. Y.; Kim, J. Y.; Son, B. W.; Jung, H. A.; Choi, J. S. Inhibitory phlorotannins from the edible brown alga Ecklonia stolonifera on total reactive oxygen species (ROS) generation. *Archives of Pharmacal Research* **2004**, *27* (2), 194-198. DOI: 10.1007/bf02980106.

(503) Ooi, D. J.; Chan, K. W.; Sarega, N.; Alitheen, N. B.; Ithnin, H.; Ismail, M. Bioprospecting the Curculigoside-Cinnamic Acid-Rich Fraction from Molineria latifolia Rhizome as a Potential Antioxidant Therapeutic Agent. *Molecules* **2016**, *21* (6). DOI: 10.3390/molecules21060682.

(504) Wang, Y.; Zhao, L.; Wang, Y.; Xu, J.; Nie, Y.; Guo, Y.; Tong, Y.; Qin, L.; Zhang, Q. Curculigoside isolated from Curculigo orchioides prevents hydrogen peroxide-induced dysfunction and oxidative damage in calvarial osteoblasts. *Acta Biochimica Et Biophysica Sinica* **2012**, *44* (5), 431-441. DOI: 10.1093/abbs/gms014.

(505) Ono, M.; Masuoka, C.; Koto, M.; Tateishi, M.; Komatsu, H.; Kobayashi, H.; Igoshi, K.; Ito, Y.; Okawa, M.; Nohara, T. Antioxidant ortho-benzoyloxyphenyl acetic acid ester, vaccihein A, from the fruit of rabbiteye blueberry (Vaccinium ashei). *Chemical & pharmaceutical bulletin* **2002**, *50* (10), 1416-1417. DOI: 10.1248/cpb.50.1416.

(506) Ye, L.; Gong, J.; Wang, Y.; Sun, X. Pharmacological Activities of Sijunzi Decoction Which Are Related to Its Antioxidant Properties. *Journal of Chemistry* **2014**. DOI: 10.1155/2014/278318.

(507) Pang, L.; Zhu, K.; Feng, X.; Liu, W.; Peng, D.; Qiu, L.; Gao, X.; Deng, J.; Li, Y.; Zhao, X. Protective effect of liensinine on periodontitis through its antioxidant effect in mice. *Journal of the Korean Society for Applied Biological Chemistry* **2015**, *58* (6), 927-936. DOI: 10.1007/s13765-015-0122-y.

(508) Tsai, J. Y.; Chou, C. J.; Chen, C. F.; Chiou, W. F. Antioxidant activity of piperlactam S: Prevention of copper-induced LDL peroxidation and amelioration of free radical-induced oxidative stress of endothelial cells. *Planta medica* **2003**, *69* (1), 3-8. DOI: 10.1055/s-2003-37040.

(509) Riaz, N.; Malik, A.; Rehman, A.; Ahmed, Z.; Muhammad, P.; Nawaz, S. A.; Siddiqui, J.; Choudhary, M. I. Lipoxygenase inhibiting and antioxidant oligostilbene and monoterpene galactoside from Paeonia emodi. *Phytochemistry* **2004**, *65* (8), 1129-1135. DOI: 10.1016/j.phytochem.2003.10.012.
(510) Jun, H. U.; Yan, X.; Wei, W.; Hao, W. U.; Lei, H.; Lijun, D. U. Antioxidant Activity In Vitro of Three Constituents from Caesalpinia sappan L. *Tsinghua Science & Technology* **2008**, *13* (4), 474-479.
(511) Sasaki, Y.; Hosokawa, T.; Nagai, M.; Nagumo, S. In vitro study for inhibition of NO production about constituents of Sappan Lignum. *Biological & Pharmaceutical Bulletin* **2007**, *30* (1), 193-196. DOI: 10.1248/bpb.30.193.
(512) Singh, S.; Khatoon, S.; Joshi, Y.; Prgyadeep, S.; Upreti, D. K.; Rawat, A. K. S. A Validated HPTLC Densitometric Method for Simultaneous Determination of Evernic and Usnic Acids in Four Usnea Species and Comparison of Their Antioxidant Potential. *Journal of chromatographic science* **2016**, *54* (9), 1670-1677. DOI: 10.1093/chromsci/bmw118.
(513) Popovici, V.; Bucur, L.; Popescu, A.; Caraiane, A.; Badea, V. Determination Of The Content In Usnic Acid And Polyphenols From The Extracts Of Usnea Barbata L. And The Evaluation Of Their Antioxidant Activity. *Farmacia* **2018**, *66* (2), 337-341.
(514) Youns, M.; ElKhoely, A.; Kamel, R. The growth inhibitory effect of gambogic acid on pancreatic cancer cells. *Naunyn-Schmiedebergs Archives of Pharmacology* **2018**, *391* (5), 551-560. DOI: 10.1007/s00210-018-1485-5.
(515) Nabi, F.; Li, K.; Shahzad, M.; Han, Z.; Zhang, D.; Liu, J.; Li, J. Gambogic Acid Inhibits Hsp90 Expressions in Thiram-Induced Tibial Dyschondroplasia. *Pakistan Veterinary Journal* **2016**, *36* (2), 224-226.
(516) da Silva Padilha, C. V.; Telles Biasoto, A. C.; Correa, L. C.; Lima, M. d. S.; Pereira, G. E. Phenolic compounds profile and antioxidant activity of commercial tropical red wines (Vitis vinifera L.) from Sao Francisco Valley, Brazil. *Journal of Food Biochemistry* **2017**, *41* (3). DOI: 10.1111/jfbc.12346.
(517) Li, S.; Yang, Y.; Li, J.; Zhu, Z.; Lorenzo, J. M.; Barba, F. J. Increasing Yield and Antioxidative Performance of Litchi Pericarp Procyanidins in Baked Food by Ultrasound-Assisted Extraction Coupled with Enzymatic Treatment. *Molecules* **2018**, *23* (9). DOI: 10.3390/molecules23092089.
(518) Mizuno, M.; Nakanishi, I.; Matsubayashi, S.; Imai, K.; Arai, T.; Matsumoto, K.-i.; Fukuhara, K. Synthesis and antioxidant activity of a procyanidin B3 analogue. *Bioorganic & Medicinal Chemistry Letters* **2017**, *27* (4), 1041-1044. DOI: 10.1016/j.bmcl.2016.12.067.
(519) Mizuno, M.; Nakanishi, I.; Matsumoto, K.-i.; Fukuhara, K. Enhanced radical scavenging activity of a procyanidin B3 analogue comprised of a dimer of planar catechin. *Bioorganic & Medicinal Chemistry Letters* **2017**, *27* (22), 5010-5013. DOI: 10.1016/j.bmcl.2017.10.007.
(520) Raudone, L.; Raudonis, R.; Liaudanskas, M.; Viskelis, J.; Pukalskas, A.; Janulis, V. Phenolic Profiles and Contribution of Individual Compounds to Antioxidant Activity of Apple Powders. *Journal of Food Science* **2016**, *81* (5), C1055-C1061. DOI: 10.1111/1750-3841.13277.
(521) Justino, A. B.; Miranda, N. C.; Franco, R. R.; Martins, M. M.; da Silva, N. M.; Espindola, F. S. Annona muricata Linn. leaf as a source of antioxidant compounds with in vitro antidiabetic and inhibitory potential against alpha-amylase, alpha-glucosidase, lipase, non-enzymatic glycation and lipid peroxidation. *Biomedicine & Pharmacotherapy* **2018**, *100*, 83-92. DOI: 10.1016/j.biopha.2018.01.172.
(522) Xiao, Y.; Dong, J.; Yin, Z.; Wu, Q.; Zhou, Y.; Zhou, X. Procyanidin B2 protects against D-galactose-induced mimetic aging in mice: Metabolites and microbiome analysis. *Food and Chemical Toxicology* **2018**, *119*, 141-149. DOI: 10.1016/j.fct.2018.05.017.
(523) Yang, Q.-N.; Deng, W.; Wu, D.-T.; Li, J.; Liu, H.-Y.; Yan, H.-L.; Du, K.; Hu, Y.-C.; Zou, L.; Huang, J.-W. Characterization, Antioxidant Capacity, and Anti-Inflammatory Activity of Polyphenol-Enriched Extracts Obtained from Unripe, Mature, and Overripe Fruits of Red-Fleshed Kiwifruit Cultivars. *Foods* **2024**, *13* (18). DOI: 10.3390/foods13182860.
(524) Fatima, M.; Rizvi, S. I. Anti Oxidative Effect of Black Tea Theaflavin on Erythrocytes Subjected to Oxidative Stress. *National Academy Science Letters-India* **2015**, *38* (1), 25-28. DOI: 10.1007/s40009-014-0285-9.
(525) Bhuyan, L. P.; Sabhapondit, S.; Baruah, B. D.; Bordoloi, C.; Gogoi, R.; Bhattacharyya, P. Polyphenolic compounds and antioxidant activity of CTC black tea of North-East India. *Food Chemistry* **2013**, *141* (4), 3744-3751. DOI: 10.1016/j.foodchem.2013.06.086.
(526) Gao, Y.; Li, W.; Jia, L.; Li, B.; Chen, Y. C.; Tu, Y. Enhancement of (-)-epigallocatechin-3-gallate and theaflavin-3-3 '-digallate induced apoptosis by ascorbic acid in human lung adenocarcinoma SPC-A-1 cells and esophageal carcinoma Eca-109 cells via MAPK pathways. *Biochemical and Biophysical Research Communications* **2013**, *438* (2), 370-374. DOI: 10.1016/j.bbrc.2013.07.078.
(527) Sang, S. M.; Tian, S. Y.; Jhoo, J. W.; Wang, H.; Stark, R. E.; Rosen, R. T.; Yang, C. S.; Ho, C. T. Chemical studies of the antioxidant mechanism of theaflavins: radical reaction products of theaflavin 3,3 '-digallate with hydrogen peroxide. *Tetrahedron Letters* **2003**, *44* (30), 5583-5587. DOI: 10.1016/s0040-4039(03)01382-0.

(528) Blaszczak, W.; Amarowicz, R.; Gorecki, A. R. Antioxidant capacity, phenolic composition and microbial stability of aronia juice subjected to high hydrostatic pressure processing. *Innovative Food Science & Emerging Technologies* **2017**, *39*, 141-147. DOI: 10.1016/j.ifset.2016.12.005.

(529) Dugasani, S.; Pichika, M. R.; Nadarajah, V. D.; Balijepalli, M. K.; Tandra, S.; Korlakunta, J. N. Comparative antioxidant and anti-inflammatory effects of 6 -gingerol, 8 -gingerol, 10 -gingerol and 6 -shogaol. *Journal of Ethnopharmacology* **2010**, *127* (2), 515-520. DOI: 10.1016/j.jep.2009.10.004.

(530) Ionov, M.; Gordiyenko, N. V.; Zukowska, I.; Tokhtaeva, E.; Mareninova, O. A.; Baram, N.; Ziyaev, K.; Rezhepov, K.; Zamaraeva, M. Stability and antioxidant activity of gossypol derivative immobilized on N-polyvinylpyrrolidone. *International journal of biological macromolecules* **2012**, *51* (5), 908-914. DOI: 10.1016/j.ijbiomac.2012.08.005.

(531) Moser, B. R. Efficacy of gossypol as an antioxidant additive in biodiesel. *Renewable Energy* **2012**, *40* (1), 65-70. DOI: 10.1016/j.renene.2011.09.022.

(532) Zhang, D.; Lu, C.; Yu, Z.; Wang, X.; Yan, L.; Zhang, J.; Li, H.; Wang, J.; Wen, A. Echinacoside Alleviates UVB Irradiation-Mediated Skin Damage via Inhibition of Oxidative Stress, DNA Damage, and Apoptosis. *Oxidative Medicine and Cellular Longevity* **2017**. DOI: 10.1155/2017/6851464.

(533) Jia, C.; Shi, H.; Jin, W.; Zhang, K.; Jiang, Y.; Zhao, M.; Tu, P. Metabolism of Echinacoside, a Good Antioxidant, in Rats: Isolation and Identification of Its Biliary Metabolites. *Drug Metabolism and Disposition* **2009**, *37* (2), 431-438. DOI: 10.1124/dmd.108.023697.

(534) Liu, X.; Pan, L.; Wang, X.; Gong, Q.; Zhu, Y. Z. Leonurine protects against tumor necrosis factor-alpha-mediated inflammation in human umbilical vein endothelial cells. *Atherosclerosis* **2012**, *222* (1), 34-42. DOI: 10.1016/j.atherosclerosis.2011.04.027.

(535) Qi, J.; Hong, Z. Y.; Xin, H.; Zhu, Y. Z. Neuroprotective Effects of Leonurine on Ischemia/Reperfusion-Induced Mitochondrial Dysfunctions in Rat Cerebral Cortex. *Biological & pharmaceutical bulletin* **2010**, *33* (12), 1958-1964. DOI: 10.1248/bpb.33.1958.

(536) Suga, A.; Takaishi, Y.; Goto, S.; Munakata, T.; Yamauchi, I.; Kogure, K. Two lignan dimers from bamboo stems (Phyllostachys edulis). *Phytochemistry* **2003**, *64* (5), 991-996. DOI: 10.1016/50031-9422(03)00422-9.

(537) Lee, Y. J.; Jung, O.; Lee, J.; Son, J.; Cho, J. Y.; Ryou, C.; Lee, S. Y. Maclurin exerts anti-cancer effects on PC3 human prostate cancer cells via activation of p38 and inhibitions of JNK, FAK, AKT, and c-Myc signaling pathways. *Nutrition research (New York, N.Y.)* **2018**, *58*, 62-71. DOI: 10.1016/j.nutres.2018.07.003.

(538) Li, X.; Gao, Y.; Li, F.; Liang, A.; Xu, Z.; Bai, Y.; Mai, W.; Han, L.; Chen, D. Maclurin protects against hydroxyl radical-induced damages to mesenchymal stem cells: Antioxidant evaluation and mechanistic insight. *Chemico-biological interactions* **2014**, *219*, 221-228. DOI: 10.1016/j.cbi.2014.06.014.

(539) Kouam, S. F.; Ngadjui, B. T.; Krohn, K.; Wafo, P.; Ajaz, A.; Choudhary, M. I. Prenylated anthronold antioxidants from the stem bark of Harungana madagascariensis. *Phytochemistry* **2005**, *66* (10), 1174-1179. DOI: 10.1016/j.phytochem.2005.03.022.

(540) Cakir, A.; Mavi, A.; Yildirim, A.; Duru, M. E.; Harmandar, H.; Kazaz, C. Isolation and characterization of antioxidant phenolic compounds from the aerial parts of Hypericum hyssopifolium L. by activity-guided fractionation. *Journal of Ethnopharmacology* **2003**, *87* (1), 73-83. DOI: 10.1016/s0378-8741(03)00112-0.

(541) Conforti, F.; Statti, G. A.; Tundis, R.; Menichini, F.; Houghton, P. Antioxidant activity of methanolic extract of Hypericum triquetrifolium Turra aerial part. *Fitoterapia* **2002**, *73* (6), 479-483. DOI: 10.1016/s0367-326x(02)00162-4.
